# Comparative genomic analysis of *Mycobacterium iranicum* UM_TJL against representative mycobacterial species suggests its environmental origin

**DOI:** 10.1038/srep07169

**Published:** 2014-11-24

**Authors:** Joon Liang Tan, Yun Fong Ngeow, Wei Yee Wee, Guat Jah Wong, Hien Fuh Ng, Siew Woh Choo

**Affiliations:** 1 Department of Medical Microbiology, Faculty of Medicine, University of Malaya, Kuala Lumpur, Malaysia; 2 Department of Oral Biology and Biomedical Sciences, Faculty of Dentistry, University of Malaya, Kuala Lumpur, Malaysia; 3 Genome Informatics Research Laboratory, High Impact Research (HIR) Building, University of Malaya, Kuala Lumpur, Malaysia

## Abstract

*Mycobacterium iranicum* is a newly reported mycobacterial species. We present the first comparative study of *M. iranicum* UM_TJL and other mycobacteria. We found *M. iranicum* to have a close genetic association with environmental mycobacteria infrequently associated with human infections. Nonetheless, UM_TJL is also equipped with many virulence genes (some of which appear to be the consequence of transduction-related gene transfer) that have been identified in established human pathogens. Taken all together, our data suggest that *M. iranicum* is an environmental bacterium adapted for pathogenicity in the human host. This comparative study provides important clues and forms the basis for future functional studies on this mycobacterium.

The nontuberculous mycobacteria (NTM) have been long considered as environmental organisms with low virulence that are only occasionally associated with infections, mostly in immunocompromised hosts. This perception, however, has been challenged in the last few decades, as advancements in medical diagnostics led to the recognition of increasing numbers of NTM species associated with human pathology^1^,^2^. It is now known that the prevalence of pulmonary NTM infections can exceed that of pulmonary tuberculosis in tuberculosis non-endemic areas^3^,^4^ and potentially fatal infections can occur in susceptible individuals^5^.

*Mycobacterium iranicum* is a new species of NTM first described by Shojaei and his colleagues who studied eight strains isolated between 2005 and 2011 from patients in six different countries^6^. Little is known about this novel species that has been implicated so far in respiratory and skin wound infections but has also been recovered from the cerebrospinal fluid of at least one patient^6^. Its role in the development of bronchiectasis was discussed by Balakrishnan et al^7^. In 2013, we published the draft genome of *M. iranicum* strain UM_TJL (hitherto referred to as UM_TJL), an isolate from the sputum of a Malaysian patient with suspected pulmonary tuberculosis complicating his underlying diabetes mellitus and ischaemic heart disease^8^. He was treated with ceftriaxone for a week and appeared to have responded with clinical improvement and a change in the smear microscopy from acid-fast bacillus positive to acid-fast bacillus negative. In the current study, we examined the UM_TJL genome to determine its genetic relationship with other *M. iranicum* strains and 29 other mycobacterial spp. with genome information lodged in public databases. We looked particularly for putative virulence determinants and evidence of horizontal transfer of genetic material from other microbial sources.

## Results

### Overview of *M. iranicum* and Phylogenies of Housekeeping Genes

**Figure 1:**
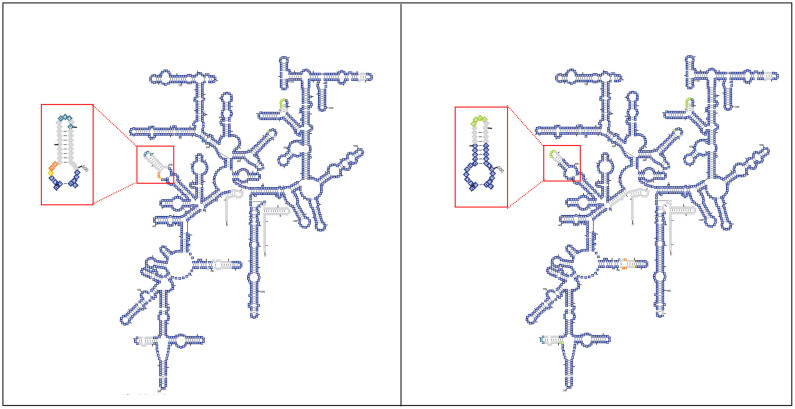
Comparison of the secondary structures of 16S rRNA between the rapidly and slowly growing mycobacteria. (A) 16S rRNA of *M. iranicum* UM_TJL (rapid grower) (B) 16S rRNA of *M. tuberculosis* (slow grower). The gaps (deletions) are illustrated by grey boxes and the aligned nucleotides are illustrated by different colours. The signature that can differentiate the rapid and slow growing mycobacteria is located at position 471 to 502 (red box). The 16S rRNA of *M. iranicum* has a shorter helix (indicated by more gaps) compared to *M. tuberculosis*.

UM_TJL has biological and genetic features of a rapid-growing mycobacterium. It is an acid-fast bacillus that formed visible colonies within 2 days on Middlebrooke 7H10 agar and it shows the genetic signature (short helix 18 in the 16S rRNA secondary structure) of rapid-growers^9^ (Figure 1).

The draft genome of UM_TJL showed general features (genome size, G+C content, number of putative coding sequences [CDSs] and RNAs) that are within the range of other annotated mycobacterial species. These features have been previously reported in brief^8^ and the genome sequence was deposited in GenBank under the accession no. AUWT00000000. The genomic organization and functional annotation are illustrated in Supplementary Figure S1 and Supplementary Figure S2 respectively.

In the *hsp65*, *rpoB* and 16S rRNA phylogenetic trees (Supplementary Figures S3, S4, S5), we observed UM_TJL, as well as other *M. iranicum* strains, to be closest to mycobacteria of low virulence for humans, such as *M. vaccae, M. aurum, M. komossense, M. gilvum and M. poriferae*.

To infer the evolutionary relationships of *M. iranicum* strains, we constructed a supermatrix tree by concatenating the three marker genes: *hsp65*, 16S rRNA and *rpoB* from UM_TJL and the strains described by Shojaei et al.^6^ (Supplementary Figure 6). Our supermatrix analysis showed that divergent evolution separated *M. iranicum* strains from northern and southern Europe. Our strain, UM_TJL clustered together with the strains from southern Europe, suggesting that it might have evolved from the southern European clade. There is only a 10 bp nucleotide difference between UM_TJL and the Italian strain FI-05198 from the southern European clade.

**Figure 2:**
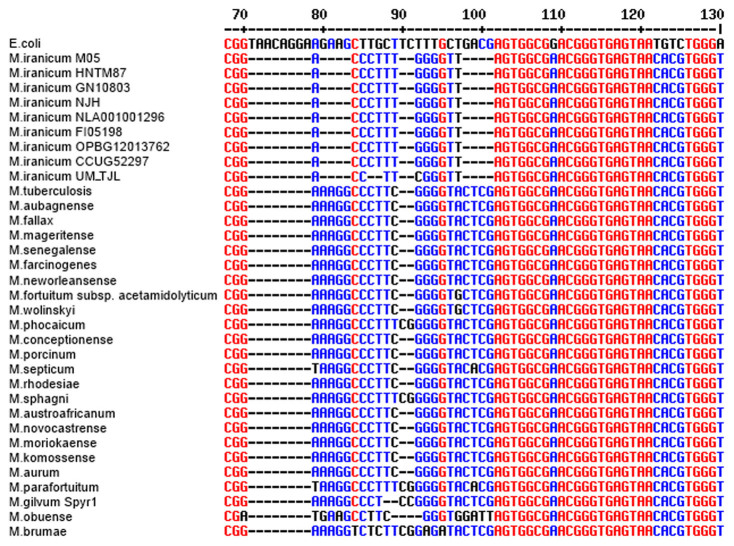
Multiple sequence alignment of 16S rRNA gene from mycobacterial species (*E.coli* numbering system). Deletions in nt 80–83 and nt 98–101 distinguish *M.iranicum* from other mycobacterial species.

Shojaei and his colleagues^6^ observed a pair of 4 bp deletions in the 16S rRNA gene sequence (from the nucleotide positions 80^th^ to 83^rd^ and 98^th^ to 101^st^ (*E. coli* numbering system)) that is believed to be unique to *M. iranicum* (Figure 2). To examine whether UM_TJL has this unique signature, we aligned its 16S rRNA gene sequence with the gene sequences from other *M. iranicum* strains and mycobacterial species. Multiple alignments of these 16S rRNA gene sequences showed that *M. iranicum* strains have the same signature. The presence of this signature in different strains from different geographical regions of the world reinforces the belief that it is *M. iranicum*-specific and not the result of random mutations.

### Comparative Genomics with Average Amino Acid Identity (AAI)

**Figure 3:**
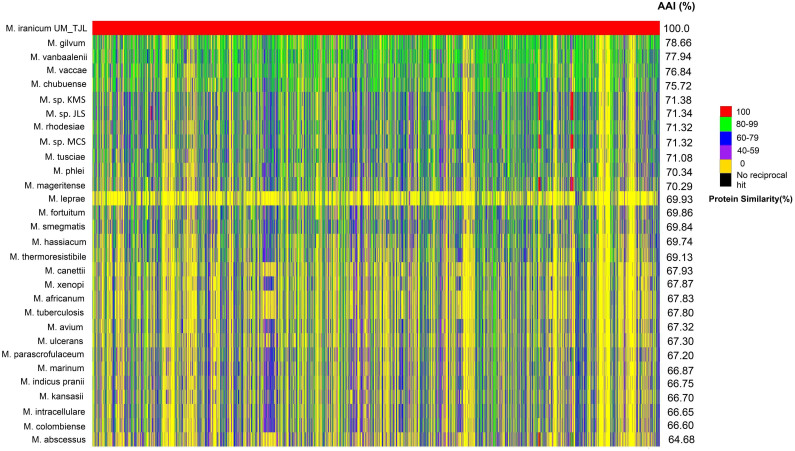
Protein similarity matrix and AAI values in descending order. The identity percentages of 5,995 CDS from UM_TJL to each homolog found in the other 29 mycobacterial species are illustrated using different colours.

The AAI has been reported to be a reliable alternative to the use of classical housekeeping genes for the evaluation of bacterial genetic relatedness^10^. It is based on the calculation of the average percentage amino acid similarity between all conserved genes in a pair of different genomes. Using UM_TJL as the reference genome, we calculated the AAI for the other 29 mycobacterial species. The results showed AAI values ranging from approximately 64% to 79% (Figure 3), with *M. gilvum* (78.66%) being the closest neighbor of UM_TJL, followed by *M. vanbaalenii* (77.94%) and *M. vaccae* (76.84%). These results are consistent with the findings of Shojaei and colleagues who studied the evolutionary relationships of these species using specific marker genes^6^.

Eleven species (*M. gilvum* to *M. mageritense* in Figure 3) showed AAI values higher than 70%. These are species with relatively low virulence that are rarely associated with opportunistic human infections. In contrast, the two best-known human pathogens *M. tuberculosis* and *M. leprae* as well as other species often isolated from human and animal material such as *M. kansasii, M. avium-intracellulare, M. marinum*, *M. ulcerans* and *M.* *abscessus* all have AAI values less than 70%. The most striking difference is seen with *M. leprae* which appeared to have only about 22% of the proteins in UM_TJL. This could be related to the reductive evolution in this highly specialized human pathogen^11^. Overall, the higher degree of genetic relatedness with free-living mycobacteria as indicated by the higher AAI values, suggests an environmental origin and habitat for UM_TJL.

### Mycobacterial Core Genes

**Figure 4:**
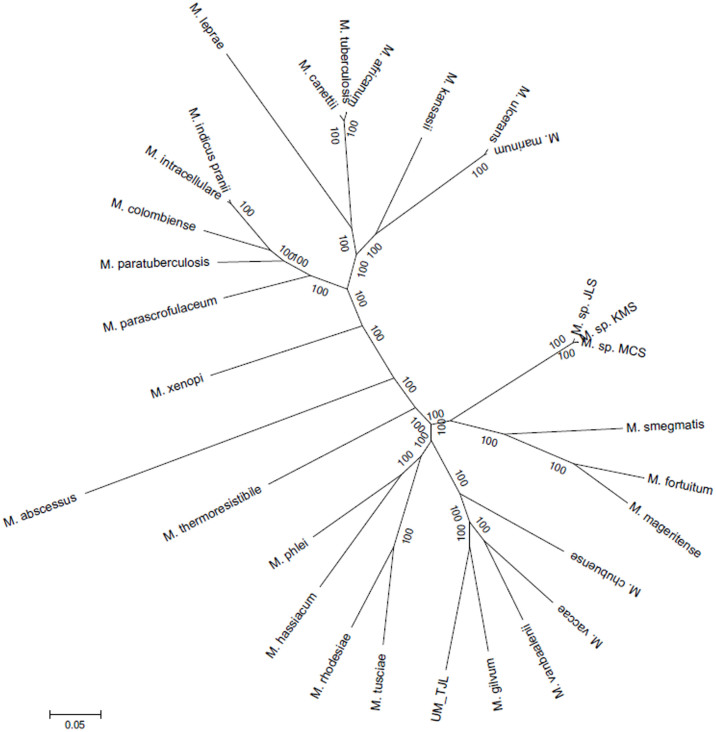
Phylogenomic tree constructed using supersequence from single-copy core genes.

As phylogenomic studies are less likely to be affected by genetic events such as horizontal gene transfer (HGT), we used the 899 core gene families identified in our 30 mycobacterial spp. to construct phylogenetic trees. We constructed a maximum likelihood tree for each of 727 single-copy core genes (data not shown) as well as one phylogenomic tree based on the concatenation of all 727 single-copy core genes (Figure 4). Both the phylogenomic and single-gene trees show a clear separation between rapid and slow growing mycobacteria, except in the case of *M. tusciae*, a slow growing mycobacterium that is clustered with the rapid growers. This conflicting genotypic-phenotypic feature of *M. tusciae* has been previously reported by Tortoli et al.^12^. The divergence between established pathogens, opportunistic pathogens and soil bacteria of low virulence (*M*. sp. JLS, *M*. sp. KMS and *M*. sp. MCS) is also discernible, and in all the trees, UM_TJL is consistently clustered in the same clade with the four environmental mycobacteria *M. chubuense*, *M. vaccae*, *M. gilvum* and *M. vanbaalenii*, supporting our view on its environmental origin.

### Genomic Islands

Genomic islands (GIs) are relatively large segments of DNA with evidence of a horizontal origin. They play important roles in microbial evolution, virulence, drug resistance and adaptation to different environments^13^. We identified 13 putative GIs in the genome of UM_TJL using the IslandViewer^14^ to make predictions based on unique features in codon usage, dinucleotide sequence composition and the presence of mobile elements. These 13 GIs were subsequently reduced to seven (Table 1) after we excluded GIs that are located within 20 Kb of the gap/border of two different concatenated contigs. Of the seven GIs, only one (GI5) is found in the other 29 spp. compared with UM_TJL. They are the environmental mycobacteria, *M. vanbaalenii*, *M. gilvum*, *M*. sp. JLS, *M*. sp. KMS and *M*. sp. MCS.

The GIs carry putative genes for toxic substances and drug resistances that are likely to confer a selective advantage in a human host environment. One of these genes is *EmrE* which encodes an antiporter that exchanges H^+^ with toxic cations such as ethidium and methyl viologen. This gene has been reported to be responsible for resistance to ethidium, acriflavine, reserpine and tetracycline^15^,^16^. Two other genes encode beta-lactamase and macrolide glycosyltransferase enzymes that are able to breakdown and inactivate beta-lactam and macrolide antibiotics respectively^17^,^18^. The product of the ErfK/YbiS/YcfS/YnhG family protein encoding gene in GI5 is involved in peptidoglycan cross-linking and causes resistance to beta-lactam antibiotics by replacing penicillin-binding proteins^19^. The lycopene beta cyclase gene in GI2 is involved in the production of proteins with a wide range of functions in different kingdoms^20^,^21^. Carotenoids are an example of the proteins synthesised^20^. As pigment production allows bacteria to tolerate oxidative stress, the production of carotenoids is indirectly associated with virulence^22^. The presence of these acquired virulence-associated and antibiotic resistance genes suggests that UM_TJL is well equipped for pathogenicity in human hosts and is likely to acquire further virulence factors via future HGT events. Phage-mediated HGT is indicated by the prediction of phage components and a recombinase in GI1. On the other hand, components of the Type II restriction-modification system predicted in GI3 indicate the possible presence of phage resistance mechanisms in UM_TJL.

### Horizontal Gene Transfer

In UM_TJL, we found 300 homologs of genes (excluding genes found in GIs) from sources of mobile elements - 5 from viruses, 10 prophages and 285 plasmids - distributed throughout the genome (Supplementary Table 1). Of these genes, 292 are found in at least one other mycobacterium sp. The remaining eight are found in *Nocardia, Rhodococcus*, *Corynebacterium* or *Achromobacter piechaudii*. Other than genes known to function in mobility (insertion sequence, transposase, recombinase and integrase), the mobile elements carry key genes that are important in biological systems of prokaryotic organisms, for instance, those involved in defense systems (Restriction Modification, abortive infection protein), drug resistance (EmrB/QacA subfamily, tetracycline resistance protein, cytochrome P450, cyclase family protein) and response to environmental stress (cold shock protein, heat shock protein). Surprisingly, despite finding many gene homologs from prophages, we were unable to identify intact prophages. The reason for this may be related to the presence of Restriction- Modification (R-M) systems. The R-M system is a phage defense mechanism in bacteria that functions with a methyl- transferase (MTase) for the modification of self DNA, and a restriction endonuclease (REase) for the cleaving of un-methylated foreign DNA. There are four types of R-M systems classified by their subunit composition, ATP(GTP) requirement and cleavage mechanism^23^. The type II R-M system is found in GI3 together with methylase subunits and the methylated cytosine restriction (mcrC) gene^24^. The enzymes in this system cleave viral DNA at specific sites^25^. We did not find any genes associated with other phage defense systems such as the clustered regularly interspaced short palindromic repeats (CRISPR) and Phage Growth Limitation (PgL) systems.

The toxin-antitoxin (TA) system was once considered a structurally unique entity that does not have specific physiological roles in the host. However, rigorous analysis revealed its significance in a wide range of molecular functions, including bacterial fitness and pathogenicity. In mycobacteria, TA has been reported to be associated with survival in *M. smegmatis*^26^ and with persistence, drug tolerance and adaptability in *M. tuberculosis* during infection^27^. We observed 28 TA homologs in UM_TJL (Supplementary Table 2) which are also present in some of the other 29 mycobacterial spp.

### Comparative Pathogenomics Analysis

The number of virulence factors in UM_TJL is comparable to that in other pathogenic mycobacteria. We predicted 225 virulence genes in the genome of UM_TJL by BLAST searching the RAST-predicted ORFs against the Virulence Factors Database (VFDB)^28^ (Supplementary Figure 7). About 25% are core virulence factors conserved in the mycobacterium genomes used in the comparative analysis. These are mainly proteins for cell wall biosynthesis (*fbpA, fbpB*, *fbpC*), translocation of cell wall components (*lprG*), interaction with macrophages (*fbpA, fbpC*), replication within macrophages (*PrrA-PrrB*), blocking lysosomal delivery (*pknG*), nitrogen metabolism (*glnA*), regulation of secretory systems (*MprA-Mpr B*) and other adaptations to the human host environment (sigma factors and two-component systems). About 40% of non-core factors are shared with the four closest species (*M. chubuense*, *M. vaccae*, *M. gilvum* and *M. vanbaalenii*). Again, these are proteins involved with two-component systems (*phoPR*), liproprotein antigens (*lpqH*), membrane transportation (*mmpL3*), synthesis of cell wall saccharides (*rmlBA*) and mammalian cell entry (*mce*). Only the homolog of *irp1* encoding a siderophore for ferrous ion uptake appear specific to UM_TJL. Although the highest number of virulence factors is found among the *M. tuberculosis* complex (*M. africanum* and *M. tuberculosis*), *M. canettii* and its close relative, *M. marinum*^29^, 164 to 230 of the virulence factors listed in the VFDB that are normally described in pathogenic mycobacteria are also identified in mycobacteria of low virulence from environmental sources *(M. gilvum, M. vanbaalenii, M. vaccae, M. chubuense, M*. sp. KMS, *M*. sp. JLS, *M*. sp. MCS and *M. indicus pranii*).

## Discussion

In this study, we compared the genome of *M. iranicum* UM_TJL to those of other mycobacterial spp. to gain a better understanding of the bacterium's genetic relatedness to other mycobacterial spp, as well as its attributes for invasion and establishing disease in a human host. The taxonomic position of UM_TJL is suggested by inter-species phylogenetic comparisons that cluster it together with other *M iranicum* strains. These comparisons are based on house-keeping genes, single-copy core genes and the concatenation of 727 single-copy core genes from 30 mycobacterial spp. representing different degrees of clinical virulence. The analysis of core genes with the exclusion of paralogs enabled us to compare the mycobacterial genomes with less chance of interference from in-paralogs and genes with ambiguous identity caused by horizontal gene transfer. Our phylogenomic and single gene phylogenetic tree topologies are all in agreement with respect to the position of UM_TJL among the mycobacterial spp. used in the comparison. An interesting observation from this study is that, although *M. iranicum* strains reported in literature so far have all been recovered from human clinical specimens, our AAI and core gene analyses show UM_TJL (a strain of *M. iranicum*) to be genetically more closely related to saprophytic species and occasional opportunistic pathogens of low virulence.

It has been hypothesized that the origin of bacterial virulence determinants is most likely to be in the environmental microbiota, and that these virulence factors are the main drivers in the evolution of environmental bacteria into pathogens^30^. Many virulence factors are acquired by bacteria via HGT. The three processes of genetic transfer among bacteria, transduction, transformation and conjugation, have all been reported in mycobacteria^31^. Conjugative transfer and transformation have been observed for both plasmid-borne and chromosomal DNA and a possible role in virulence has been suggested for the large number of bacteriophages identified in mycobacteria. In UM_TJL, there is evidence of virus-, phage- and plasmid-mediated HGT resulting in a large number of GIs, TAs and virulence factors. These HGT events appear to have occurred not only among mycobacteria but also between mycobacteria and closely-related gram-positive bacteria (*Rhodococcus, Corynebacterium* and *Nocardia*) and in at least one gram-negative bacterium (Achromobacter). Furthermore, there are more putative virulence genes in the backbone of the UM_TJL genome than in more established human pathogens like *M. abscessus*. The fact that most of these genes are also found in other environmental mycobacteria, some of which have never been isolated before from human material, furthers the theory that virulence factors are likely to be part of the primitive essentials for bacterial survival in the natural environment. When bacteria stray into clinical settings, however, these same virulence determinants provide the ammunition for cell invasion, overcoming host resistance, multiplication and patho-adaptability in human hosts.

### Conclusion

The genome of *M. iranicum* is described for the first time as strain UM_TJL, a clinical isolate from a Malaysian patient. The genomic comparison of *M. iranicum* with other mycobacterial species is also reported for the first time. UM_TJL shows the closest genomic relatedness to environmental mycobacterial species, and, like them, harbours an abundance of virulence factors, many of which are associated with mobile genetic elements. These features suggest that *M. iranicum* strains are environmental bacteria that might have evolved into consequential human pathogens with the aid of HGT.

## Methods

### Genomic Assessment

As previously described^8^, the genome of UM_TJL was recovered from reads obtained with Illumina HiSeq 2000 whole genome sequencing at approximately 618-fold coverage (assuming a genome size of 6 Mb). Prior to assembly, the quality of sequencing reads were visualized using FastQC and quality trimmed at the standard threshold of Q20. After assembly, the genome quality was assessed based on sequence continuity (length-weighted median size of 116,366 bp) and contamination screening against common contaminant databases. Only contigs larger than 200 bp were used in downstream analyses. Genome annotation was done with The Rapid Annotation using Subsystem Technology (RAST)^32^ (Supplementary Table 4.).

Similarly, the genomes of 29 other mycobacterial species were retrieved from NCBI Genbank and submitted to RAST for annotation. The resulted annotations were used for comparative genomic analysis with the UM_TJL genome (Supplementary Table 3).

### Phylogenetic Trees Reconstruction

We first used standard markers (*hsp65*, *rpoB* and 16S rRNA) to infer the phylogenetic relationship of UM_TJL with another 30 mycobacteria identified through an updated version of Bioinformatics Bacteria Identification Tool - leBIBI V5^33^, available at http://umr5558-sud-str1.univ-lyon1.fr/lebibi/lebibi.cgi. Subsequently, we constructed phylogenetic trees for single-copy core genes of UM_TJL and the 29 selected genomes listed in Supplementary Table 3. All gene sets were aligned using MAFFT^34^, and phylogenetic trees were inferred using the FastTree2 program^35^.

### AAI Calculation

The average amino acid identity (AAI) was calculated using the method described by Konstantinidis & Tiedje^10^. The UM_TJL genome was used as the reference for comparison against other mycobacterial genomes. Conserved proteins were determined by whole-genome pairwise sequence comparisons using the BLAST algorithm^36^. A two-way BLAST was used and only forward and reverse-matched orthologs were used in AAI calculations. To minimize errors in the inference of homology, thresholds were set above the twilight zone^37^ so that all conserved proteins have a BLAST match of at least 40% identity at the amino acid level and a sequence coverage of at least 70%^38^. The genetic relatedness between a pair of genomes was measured by the AAI of all conserved proteins between the two genomes as computed by the BLAST algorithm.

### Prediction of Horizontal Gene Transfer

#### Genomic islands

The draft genome of UM_TJL was submitted to IslandViewer^18^ for genomic island prediction. This software implemented sequence composition base approaches from SIGI-HMM^39^ and IslandPath-DIMOB^40^. The two programs had previously shown specificity up to approximately 86% to 92% and accuracy of 86%^41^. In addition, IslandViewer also included the comparative genomics approach from IslandPick^18^. The results generated from IslandViewer were further filtered by eliminating the islands situated within 20 Kb of the gap/border of two different concatenated contigs and having a size less than 10 Kb.

#### Other horizontally transferred genes

We investigated the presence of other putative horizontally transferred genes by performing BLAST searches against A CLAssification of Mobile genetic Elements (ACLAME)^42^ and Toxin-Antitoxin Database (TADB)^43^.

### Comparative Pathogenomics Analysis

The genome sequences annotated through RAST yielded predicted protein functions derived from the subsystems in FIGfams^9^. Protein sequences were downloaded from the RAST server and a homology search was performed on these sequences against Virulence Factor Database (VFDB)^23^ through the use of BLASTP^14^. The resulted hits were filtered using an in-house Perl script by accepting orthologs at the threshold of 60% identity and 60% completeness between queries and subjects. Instead of using the tabular comparison for pathogenomic composition, we harnessed the data collected from VFDB to construct a graphical representation to allow for the spontaneous comparison of pathogenomic profiles between genomes.

## Author Contributions

S.W.C., Y.F.N. and J.L.T. designed the experiments. J.L.T., W.Y.W., G.J.W. and S.W.C. performed the bioinformatics analysis. H.F.N. and Y.F.N. performed the experiments. J.L.T., Y.F.N. and S.W.C. wrote the paper.

## Supplementary Material

Supplementary Information Supplementary

## Figures and Tables

**Table 1:** Genomic Islands and key genes

GIs	Length (bp)	No. of CDS	GC content (%)	Key Genes and gene products
GI1	17,601	22	67.1	*EmrE* (multidrug resistance efflux gene in *E. coli*)
				Metallo-beta-lactamase superfamily proteins
				Phage Terminase
				Phage capsid and scaffold
				Recombinase
GI2	21,736	28	63.6	Macrolide glycosyltransferase
				Lycopene beta cyclase
				ATPase AAA
				Transposase
GI3	34,418	22	62.5	*mcrC* (a gene from the methylated cytosine restriction system)
				Type II restriction enzyme, methylase subunits
				Luciferase family protein
				DNA-invertase
GI4	11,698	14	66.9	Transcriptional regulators
				Beta-glucosidase
GI5	13,381	9	67.7	Transposase
				ErfK/YbiS/YcfS/YnhG family protein
GI6	12,375	10	59.4	Cell division protein DivIC (FtsB)
GI7	15,558	7	54.6	Methyl-accepting chemotaxis protein
