# Supplementary material for: Comparative genomic analysis of Mycobacterium iranicum UM_TJL against representative mycobacterial species suggests its environmental origin
Source: Sci Rep. 2014 Nov 24;4:7169. doi: 10.1038/srep07169 (PMC4241510; doi:10.1038/srep07169)
Supplement: Supplementary Information — Supplementary [file srep07169-s1.pdf]

Comparative genomic analysis of *Mycobacterium iranicum* UM_TJL against representative mycobacterial species suggest its environmental origin

Joon Liang Tan[1,3], Yun Fong Ngeow[1*], Wei Yee Wee[2,3], Guat Jah Wong[2,3], Hien Fuh Ng[1], Siew Woh Choo[2,3*]

Department of Medical Microbiology, Faculty of Medicine, University of Malaya, Kuala Lumpur, Malaysia[1]

Department of Oral Biology and Biomedical Sciences, Faculty of Dentistry, University of Malaya, Kuala Lumpur, Malaysia[2]

Genome Informatics Research Laboratory, High Impact Research (HIR) Building, University of Malaya, Kuala Lumpur, Malaysia[3]

Corresponding authors:

Professor Dr. Yun Fong Ngeow

Department of Medical Microbiology,

Faculty of Medicine,

University of Malaya, 50603 Kuala Lumpur,

Malaysia

Email address: yunngeow@um.edu.my

Dr. Siew Woh Choo

Department of Oral Biology & Biomedical Sciences,

Faculty of Dentistry,

University of Malaya, 50603 Kuala Lumpur,

Malaysia. Email address: lchoo@um.edu.my

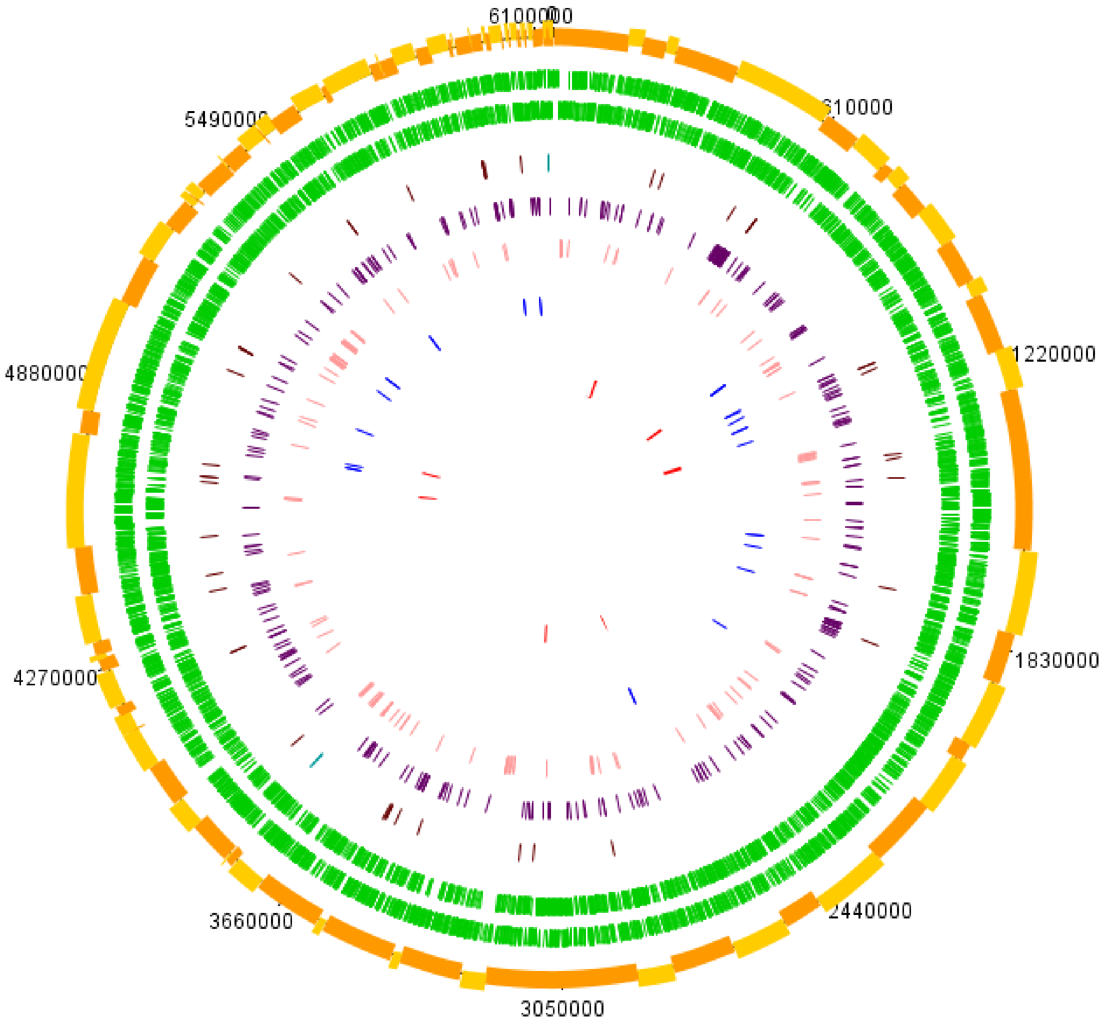

Figure S1. Genomic content of *M. iranicum* UM_TJL. From the outermost ring: 1) Alternate bright and dark orange – contigs; 2) Green – forward and reverse cds; 3) Brown and greenish – tRNA and rRNA; 4) purple ring - putative horizontally transferred genes 5) pink – virulence factors 6) blue – toxin-antitoxin 7) red – genomic islands

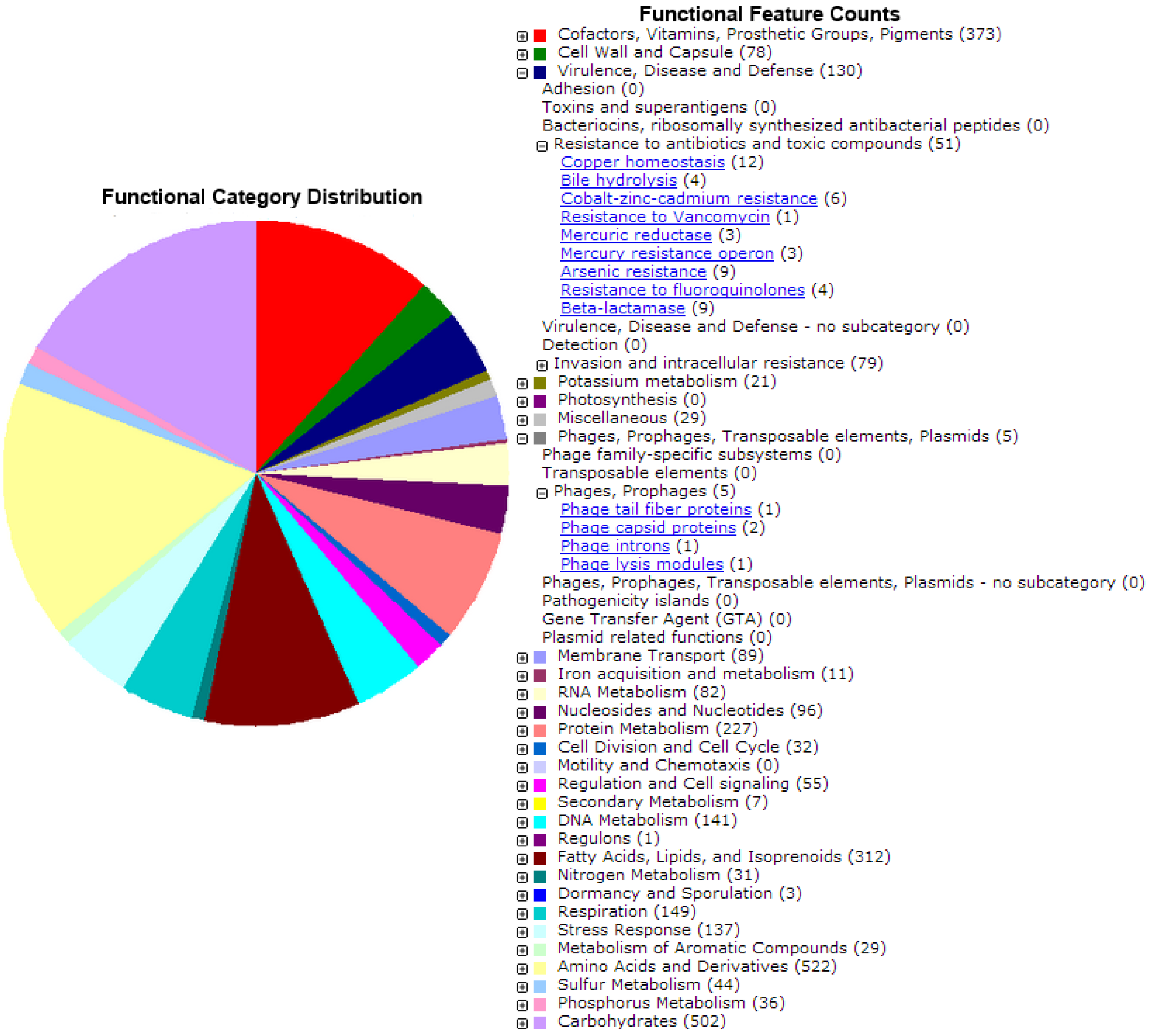

Figure S2. Functional categories of 1,968 protein-coding genes predicted in the genome of UM_TJL.

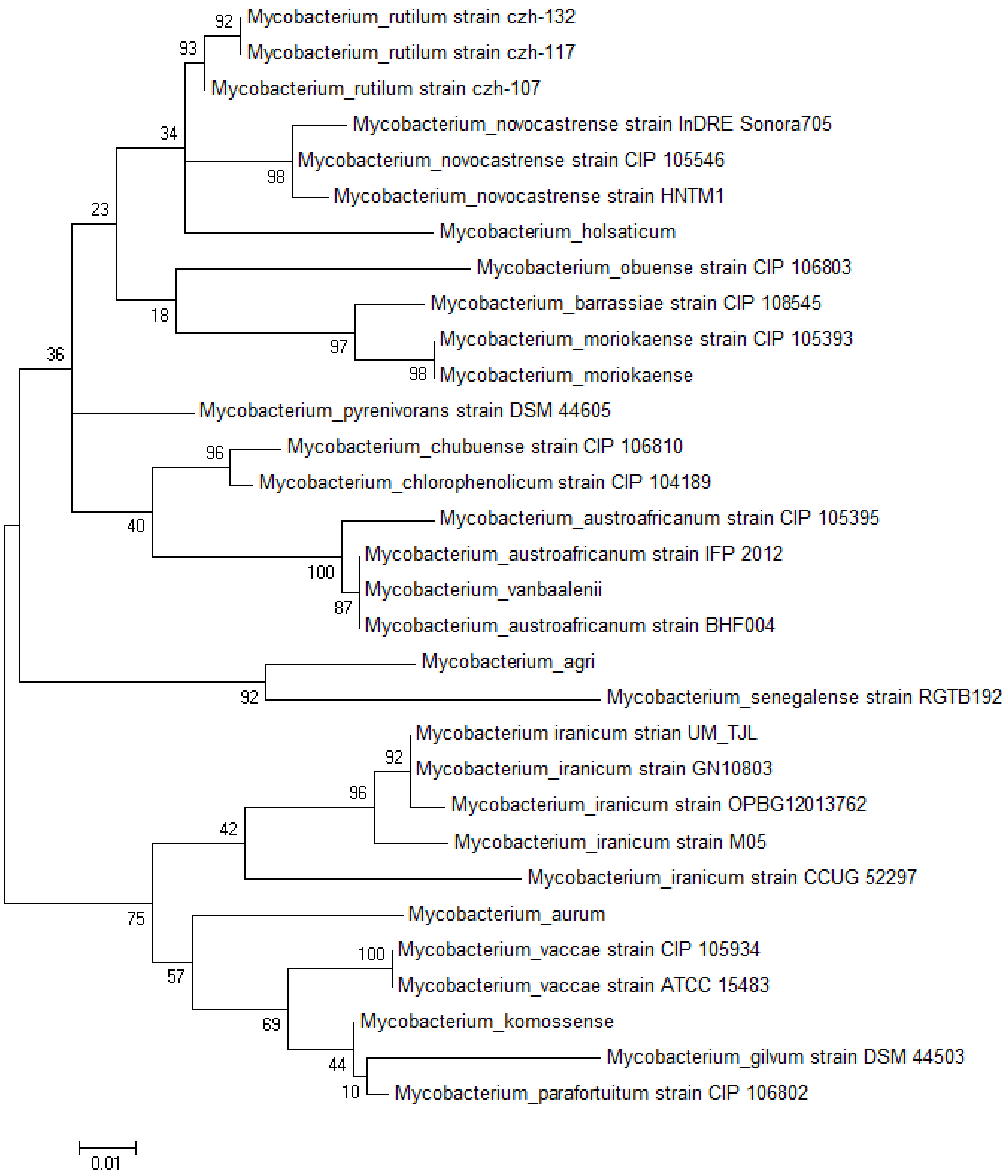

Figure S3. *hsp65*-based maximum likelihood inference. The *hsp65* phylogenetic tree supports the identity of UM_TJL as a new member of *M. iranicum*.

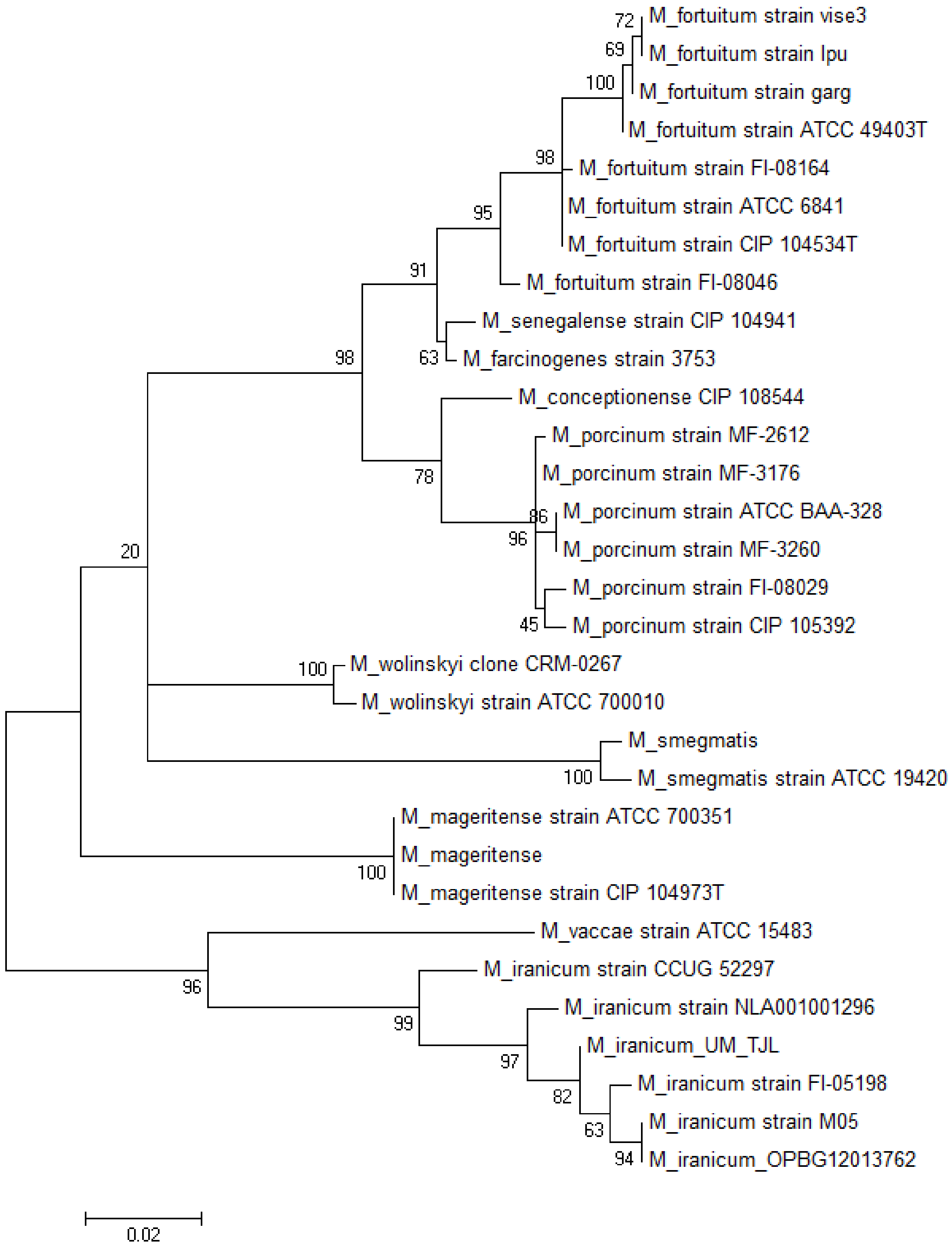

Figure S4. *rpoB*-based maximum likelihood inference. The *rpoB*-based phylogenetic tree clearly distinguishes UM_TJL and other *M. iranicum* strains from other mycobacterium species.

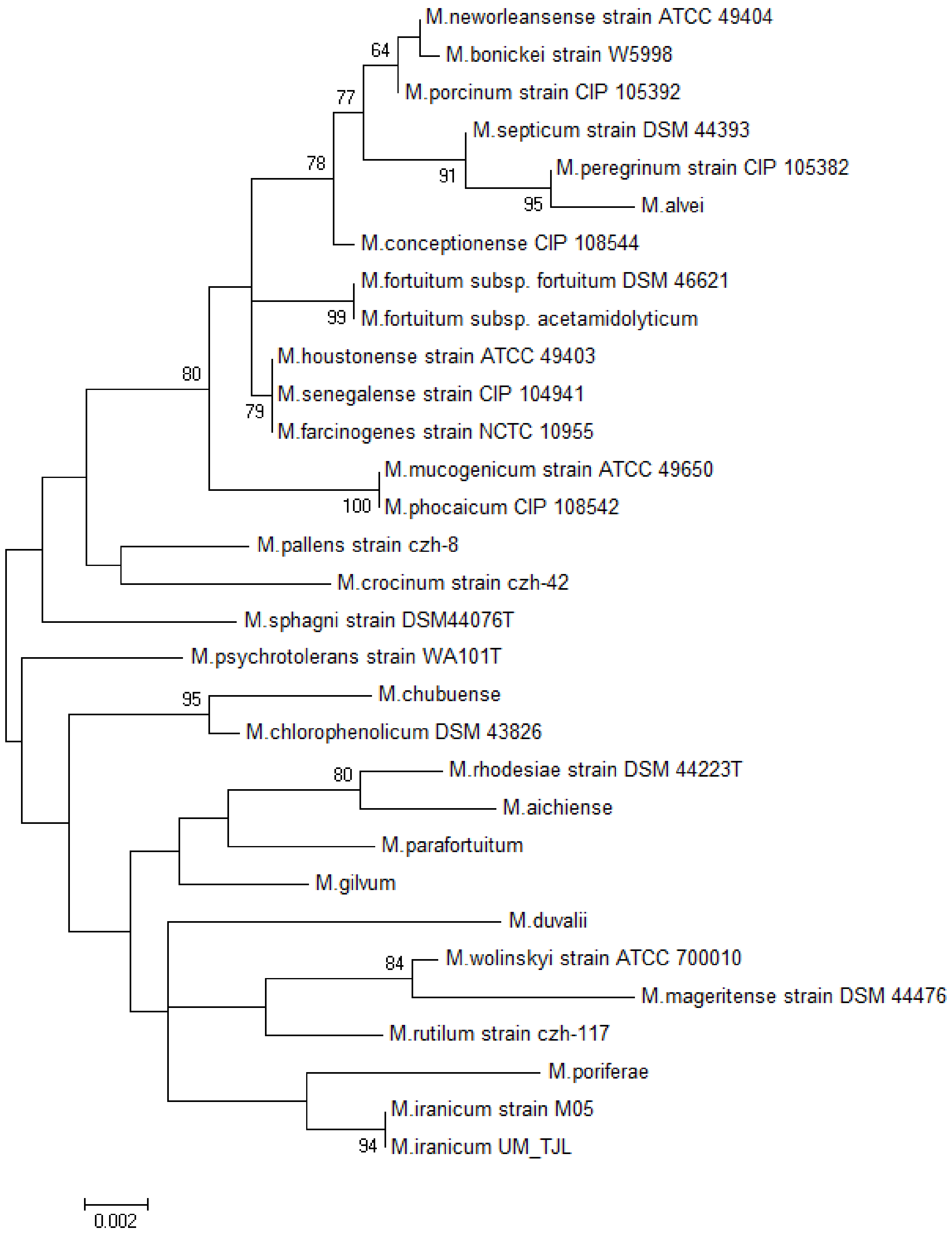

Figure S5. ***16s rRNA*-based maximum likelihood inference.** UM_TJL is positioned within the same clade as M. iranicum M05.

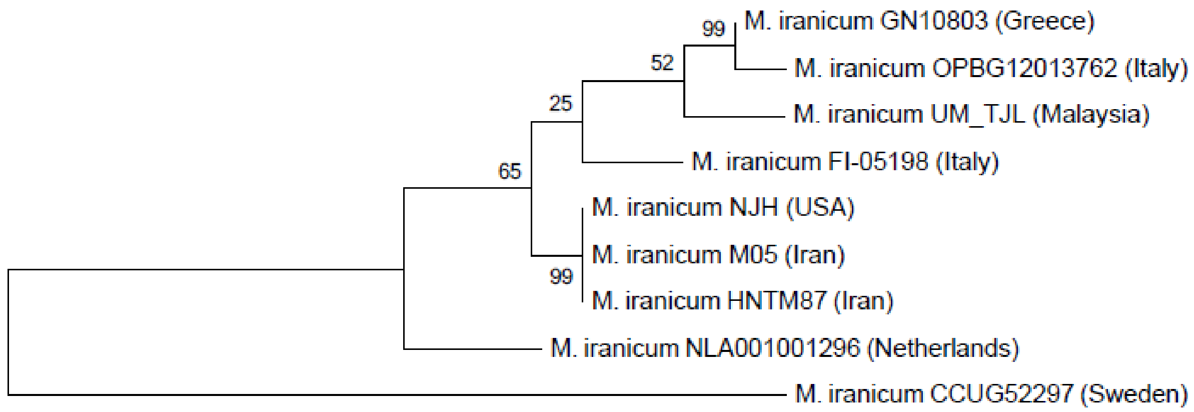

Figure S6. Supermatrix tree for *M. iranicum* strains reconstructed with the concatenation of *16S rRNA*, *rpoB* and *hsp65*. Strains described by Shojaei et al. (2013) were used to infer the evolutionary relationship among the *M. iranicum* strains.

Supplementary Table 1. All UM_TJL protein encoding sequences with homologs in ACLAME database. The highlighted proteins are the sequences present in GIs.

RAST CDS	Source	Annotation
fig|6666666.28487.peg.39	plasmid	abortive infection protein
fig|6666666.28487.peg.81	plasmid	methionine sulfoxide reductase B
fig|6666666.28487.peg.82	plasmid	peptide methionine sulfoxide reductase msrA
fig|6666666.28487.peg.98	plasmid	hypothetical protein
fig|6666666.28487.peg.160	plasmid	class III aminotransferase
fig|6666666.28487.peg.166	plasmid	protein of unknown function DUF344
fig|6666666.28487.peg.173	plasmid	hypothetical protein
fig|6666666.28487.peg.190	plasmid	probable transposase
fig|6666666.28487.peg.191	plasmid	integrase, catalytic region
fig|6666666.28487.peg.194	plasmid	urease gamma subunit
fig|6666666.28487.peg.200	plasmid	NADH dehydrogenase
fig|6666666.28487.peg.207	plasmid	fructose-6-phosphate phosphoketolase
fig|6666666.28487.peg.306	plasmid	hypothetical protein
fig|6666666.28487.peg.313	plasmid	IS sequence
fig|6666666.28487.peg.317	plasmid	integrase, catalytic region
fig|6666666.28487.peg.318	plasmid	transposase IS3/IS911 family protein
fig|6666666.28487.peg.320	plasmid	hypothetical protein
fig|6666666.28487.peg.327	plasmid	50S ribosomal protein L21
fig|6666666.28487.peg.428	plasmid	dihydroxy-acid dehydratase
fig|6666666.28487.peg.486	plasmid	putative thioredoxin reductase (TrxB)
fig|6666666.28487.peg.487	prophage	thioredoxin I
fig|6666666.28487.peg.501	plasmid	phosphogluconate dehydrogenase (decarboxylating)
fig|6666666.28487.peg.505	plasmid	DNA topoisomerase IV, A subunit
fig|6666666.28487.peg.509	plasmid	L-serine dehydratase 1
fig|6666666.28487.peg.525	prophage	type I restriction-modification system, M subunit
fig|6666666.28487.peg.533	plasmid	hypothetical protein
fig|6666666.28487.peg.539	plasmid	type II methyltransferase
fig|6666666.28487.peg.550	plasmid	type I restriction-modification system methyltransferase subunit
fig|6666666.28487.peg.552	plasmid	type I site-specific modification system restriction subunit
fig|6666666.28487.peg.582	virus	peptidoglycan binding domain-containing protein
fig|6666666.28487.peg.605	plasmid	ferredoxin
fig|6666666.28487.peg.617	plasmid	helicase domain protein
fig|6666666.28487.peg.618	plasmid	type II restriction enzyme methylase subunit
fig|6666666.28487.peg.619	plasmid	hypothetical protein
fig|6666666.28487.peg.621	plasmid	helicase domain protein
fig|6666666.28487.peg.623	plasmid	hypothetical protein
fig|6666666.28487.peg.679	plasmid	probable serine/threonine protein kinase
fig|6666666.28487.peg.712	plasmid	protein of unknown function DUF302

fig|6666666.28487.peg.713	plasmid	protein of unknown function DUF156
fig|6666666.28487.peg.734	plasmid	hypothetical protein
fig|6666666.28487.peg.735	plasmid	DSBA oxidoreductase
fig|6666666.28487.peg.785	plasmid	2-oxo-acid dehydrogenase E1 subunit, homodimeric type
fig|6666666.28487.peg.806	plasmid	phage Gp37Gp68
fig|6666666.28487.peg.851	plasmid	cytochrome c oxidase, subunit III
fig|6666666.28487.peg.855	plasmid	NLP/P60 protein
fig|6666666.28487.peg.905	plasmid	protein of unknown function DUF112 transmembrane
fig|6666666.28487.peg.921	plasmid	ferredoxin reductase
fig|6666666.28487.peg.922	plasmid	CYP153 protein
fig|6666666.28487.peg.923	plasmid	2Fe-2S ferredoxin
fig|6666666.28487.peg.953	plasmid	thiamine biosynthesis protein
fig|6666666.28487.peg.981	plasmid	possible F420-dependent glucose-6-phosphate dehydrogenase
fig|6666666.28487.peg.984	plasmid	nitrilotriacetate monooxygenase
fig|6666666.28487.peg.990	plasmid	type II restriction modification
fig|6666666.28487.peg.1055	plasmid	heat shock protein Hsp70
fig|6666666.28487.peg.1057	plasmid	putative enzyme, similar to uricase protein
fig|6666666.28487.peg.1079	plasmid	aminotransferase AlaT
fig|6666666.28487.peg.1180	plasmid	epoxide hydrolase domain protein
fig|6666666.28487.peg.1199	plasmid	transcriptional regulator, MarR family
fig|6666666.28487.peg.1200	plasmid	drug resistance transporter, EmrB/QacA subfamily
fig|6666666.28487.peg.1204	plasmid	citrate (Si)-synthase
fig|6666666.28487.peg.1235	plasmid	transposase, mutator type
fig|6666666.28487.peg.1236	plasmid	transposase, mutator type
fig|6666666.28487.peg.1240	virus	single-stranded DNA-binding protein
fig|6666666.28487.peg.1248	plasmid	inositol-3-phosphate synthase
fig|6666666.28487.peg.1255	plasmid	putative ATP-binding component of ABC transporter
fig|6666666.28487.peg.1263	plasmid	fatty-acid--CoA ligase
fig|6666666.28487.peg.1264	plasmid	acyl-CoA dehydrogenase
fig|6666666.28487.peg.1266	plasmid	sterol carrier protein
fig|6666666.28487.peg.1267	plasmid	putative zinc-containing alcohol dehydrogenase
fig|6666666.28487.peg.1270	plasmid	transposase IS3/IS911 family protein
fig|6666666.28487.peg.1271	plasmid	integrase, catalytic region
fig|6666666.28487.peg.1278	plasmid	metal dependent phosphohydrolase
fig|6666666.28487.peg.1279	plasmid	NUDIX hydrolase
fig|6666666.28487.peg.1280	plasmid	transcriptional regulator, XRE family
fig|6666666.28487.peg.1281	plasmid	regulatory protein
fig|6666666.28487.peg.1282	plasmid	cell divisionFtsK/SpoIIIE
fig|6666666.28487.peg.1290	plasmid	PyrD
fig|6666666.28487.peg.1294	plasmid	BacA
fig|6666666.28487.peg.1300	plasmid	hypothetical protein

fig|6666666.28487.peg.1302	plasmid	B12-dependent methionine synthase
fig|6666666.28487.peg.1308	plasmid	FAD-dependent pyridine nucleotide-disulphide oxidoreductase
fig|6666666.28487.peg.1380	plasmid	protein of unknown function UPF0060
fig|6666666.28487.peg.1431	plasmid	hypothetical protein
fig|6666666.28487.peg.1450	plasmid	probable 2-hydroxy-6-oxo-6-phenylhexa-2,4-dienoate hydrolase
fig|6666666.28487.peg.1468	plasmid	Probable amino-acid ATP-binding ABC transporter protein
fig|6666666.28487.peg.1475	prophage	recombinase A
fig|6666666.28487.peg.1502	virus	putative thymidylate synthase
fig|6666666.28487.peg.1561	plasmid	cytochrome c assembly protein
fig|6666666.28487.peg.1562	plasmid	putative cytochrome c biogenesis protein ResB
fig|6666666.28487.peg.1563	plasmid	cytochrome c biogenesis protein, transmembrane region
fig|6666666.28487.peg.1564	plasmid	alkyl hydroperoxide reductase/ Thiol specific antioxidant/ Mal allergen
fig|6666666.28487.peg.1654	plasmid	putative monooxygenase protein
fig|6666666.28487.peg.1680	prophage	chaperonin GroEL
fig|6666666.28487.peg.1719	plasmid	transcriptional regulator, TetR family
fig|6666666.28487.peg.1720	plasmid	ABC-1 domain protein
fig|6666666.28487.peg.1721	plasmid	cytochrome P450
fig|6666666.28487.peg.1784	plasmid	acyl-CoA dehydrogenase domain protein
fig|6666666.28487.peg.1785	plasmid	methylmalonate-semialdehyde dehydrogenase
fig|6666666.28487.peg.1793	plasmid	translation initiation factor IF-1
fig|6666666.28487.peg.1815	plasmid	aldehyde dehydrogenase (NAD+)
fig|6666666.28487.peg.1848	plasmid	phospho-2-dehydro-3-deoxyheptonate aldolase
fig|6666666.28487.peg.1911	plasmid	acyltransferase 3
fig|6666666.28487.peg.1933	plasmid	methylmalonyl-CoA mutase protein
fig|6666666.28487.peg.1946	plasmid	NLP/P60 protein
fig|6666666.28487.peg.1949	plasmid	aconitate hydratase
fig|6666666.28487.peg.1961	plasmid	hypothetical protein
fig|6666666.28487.peg.1963	plasmid	putative cobalt-containing nitrile hydratase subunit alpha
fig|6666666.28487.peg.1978	plasmid	cyclase family protein
fig|6666666.28487.peg.2115	plasmid	possible carnitinyl-CoA dehydratase
fig|6666666.28487.peg.2144	plasmid	3-hydroxyacyl-CoA dehydrogenase
fig|6666666.28487.peg.2159	plasmid	putative glutaredoxin
fig|6666666.28487.peg.2162	plasmid	type II restriction modification
fig|6666666.28487.peg.2178	plasmid	ferredoxin
fig|6666666.28487.peg.2195	plasmid	cytochrome-c oxidase
fig|6666666.28487.peg.2233	plasmid	4Fe-4S ferredoxin, iron-sulfur binding domain protein
fig|6666666.28487.peg.2289	plasmid	putative lipoprotein
fig|6666666.28487.peg.2290	plasmid	transcriptional repressor, CopY family
fig|6666666.28487.peg.2302	plasmid	possible enoyl-CoA hydratase
fig|6666666.28487.peg.2348	plasmid	acetaldehyde dehydrogenase
fig|6666666.28487.peg.2349	plasmid	2-hydroxypenta-2,4-dienoate hydratase

fig|6666666.28487.peg.2377	plasmid	putative acetyl-CoA acyltransferase
fig|6666666.28487.peg.2396	plasmid	short-chain dehydrogenase/reductase SDR
fig|6666666.28487.peg.2403	plasmid	possible tetracycline resistance protein
fig|6666666.28487.peg.2456	plasmid	carbonate dehydratase
fig|6666666.28487.peg.2464	plasmid	cobalt transporter
fig|6666666.28487.peg.2480	plasmid	GTP cyclohydrolase I
fig|6666666.28487.peg.2507	plasmid	cold-shock protein, CspA family
fig|6666666.28487.peg.2510	prophage	serine/threonine protein kinase
fig|6666666.28487.peg.2520	plasmid	acetate--CoA ligase
fig|6666666.28487.peg.2532	plasmid	LysR family transcriptional regulator
fig|6666666.28487.peg.2539	plasmid	pyridoxal-5'-phosphate-dependent enzyme subunit beta
fig|6666666.28487.peg.2603	plasmid	catalase protein
fig|6666666.28487.peg.2626	plasmid	hypothetical protein
fig|6666666.28487.peg.2627	plasmid	domain of unknown function DUF1801
fig|6666666.28487.peg.2686	plasmid	AMP-dependent synthetase and ligase
fig|6666666.28487.peg.2756	plasmid	putative asparagine synthetase (glutamine amidotransferase) protein
fig|6666666.28487.peg.2771	plasmid	putative dehydrogenase protein
fig|6666666.28487.peg.2803	plasmid	putative non-heme chloroperoxidase
fig|6666666.28487.peg.2819	plasmid	catalase
fig|6666666.28487.peg.2827	prophage	choloylglycine hydrolase
fig|6666666.28487.peg.2831	plasmid	formyl-methanofuran dehydrogenase domain 3 protein
fig|6666666.28487.peg.2832	plasmid	glutamate synthase large subunit 2 protein
fig|6666666.28487.peg.2882	plasmid	microcompartments protein
fig|6666666.28487.peg.2885	plasmid	microcompartments protein
fig|6666666.28487.peg.2886	plasmid	microcompartments protein
fig|6666666.28487.peg.2893	plasmid	probable thioredoxin
fig|6666666.28487.peg.2897	plasmid	protein of unknown function DUF81
fig|6666666.28487.peg.2898	plasmid	hypothetical protein
fig|6666666.28487.peg.2899	plasmid	beta-lactamase domain-containing protein
fig|6666666.28487.peg.2901	plasmid	putative short chain oxidoreductase protein
fig|6666666.28487.peg.2913	plasmid	hydrolase
fig|6666666.28487.peg.2960	plasmid	cytochrome-c oxidase
fig|6666666.28487.peg.2978	plasmid	divalent cation-transport integral membrane protein
fig|6666666.28487.peg.3050	plasmid	vitamin B6 biosynthesis protein
fig|6666666.28487.peg.3075	plasmid	sn-glycerol-3-phosphate ABC transporter, ATP-binding protein
fig|6666666.28487.peg.3098	plasmid	beta-lactamase superfamily metal-dependent hydrolase
fig|6666666.28487.peg.3101	plasmid	probable nitroreductase protein
fig|6666666.28487.peg.3108	plasmid	hypothetical protein
fig|6666666.28487.peg.3109	plasmid	putative arsenate reductase (ArsC)
fig|6666666.28487.peg.3110	plasmid	putative arsenate reductase
fig|6666666.28487.peg.3111	plasmid	putative arsenite export protein

fig|6666666.28487.peg.3112	plasmid	putative transcriptional regulator
fig|6666666.28487.peg.3132	plasmid	putative NAD(P) transhydrogenase subunit A
fig|6666666.28487.peg.3307	plasmid	Probable glucarate dehydratase protein
fig|6666666.28487.peg.3352	plasmid	probable S-adenosylmethionine synthetase
fig|6666666.28487.peg.3419	plasmid	cold-shock protein, CspA family
fig|6666666.28487.peg.3423	plasmid	cold-shock protein, CspA family
fig|6666666.28487.peg.3431	plasmid	aldehyde dehydrogenase
fig|6666666.28487.peg.3449	plasmid	alcohol dehydrogenase
fig|6666666.28487.peg.3459	plasmid	possible forkhead associated domain (FHA)
fig|6666666.28487.peg.3488	plasmid	putative mandelate racemase
fig|6666666.28487.peg.3512	plasmid	amino acid transport protein
fig|6666666.28487.peg.3513	plasmid	ethanolamine ammonia lyase large subunit
fig|6666666.28487.peg.3517	plasmid	acetolactate synthase
fig|6666666.28487.peg.3521	plasmid	catalase-peroxidase
fig|6666666.28487.peg.3525	prophage	isocitrate dehydrogenase, NADP-dependent, monomeric type
fig|6666666.28487.peg.3541	plasmid	putative glucose-6-phosphate dehydrogenase
fig|6666666.28487.peg.3553	plasmid	probable 4-coumarate--CoA ligase
fig|6666666.28487.peg.3582	plasmid	pyruvate carboxyltransferase
fig|6666666.28487.peg.3583	plasmid	acetaldehyde dehydrogenase
fig|6666666.28487.peg.3585	virus	gp65
fig|6666666.28487.peg.3602	prophage	ATP-dependent protease ATP-binding subunit
fig|6666666.28487.peg.3636	plasmid	type II methyltransferase
fig|6666666.28487.peg.3661	plasmid	NAD+ synthase
fig|6666666.28487.peg.3670	plasmid	malate synthase G
fig|6666666.28487.peg.3729	plasmid	putative ATP-binding component of ABC transporter
fig|6666666.28487.peg.3775	prophage	hypothetical protein
fig|6666666.28487.peg.3776	prophage	hypothetical protein
fig|6666666.28487.peg.3806	plasmid	possible glutamate synthase (ferredoxin)
fig|6666666.28487.peg.3811	plasmid	putative short-chain dehydrogenase
fig|6666666.28487.peg.3844	plasmid	integrase, catalytic region
fig|6666666.28487.peg.3852	plasmid	hypothetical protein
fig|6666666.28487.peg.3863	plasmid	K+-transporting ATPase, B subunit
fig|6666666.28487.peg.3873	plasmid	propionate--CoA ligase
fig|6666666.28487.peg.3879	plasmid	putative conserved lipoprotein LpqU
fig|6666666.28487.peg.3986	plasmid	putative ABC transporter protein
fig|6666666.28487.peg.3993	plasmid	Probable peptide synthetase protein
fig|6666666.28487.peg.4006	plasmid	4Fe-4S ferredoxin, iron-sulfur binding domain protein
fig|6666666.28487.peg.4011	plasmid	FO synthase
fig|6666666.28487.peg.4048	plasmid	short chain dehydrogenase
fig|6666666.28487.peg.4133	plasmid	acetyl-CoA C-acyltransferase
fig|6666666.28487.peg.4219	plasmid	ErfK/YbiS/YcfS/YnhG family protein

fig|6666666.28487.peg.4230	plasmid	putative methylcrotonoyl-CoA carboxylase non-biotinylated subunit protein
fig|6666666.28487.peg.4291	plasmid	peptidase M23B
fig|6666666.28487.peg.4302	plasmid	glucose-6-phosphate isomerase
fig|6666666.28487.peg.4312	plasmid	putative phytoene dehydrogenase
fig|6666666.28487.peg.4352	plasmid	aldehyde dehydrogenase
fig|6666666.28487.peg.4367	plasmid	biotin carboxylase
fig|6666666.28487.peg.4377	plasmid	propionyl-CoA carboxylase beta subunit
fig|6666666.28487.peg.4425	plasmid	rubredoxin
fig|6666666.28487.peg.4429	plasmid	probable adenosylhomocysteinase
fig|6666666.28487.peg.4446	plasmid	protein of unknown function DUF344
fig|6666666.28487.peg.4469	plasmid	putative regulatory protein
fig|6666666.28487.peg.4510	plasmid	putative relaxase
fig|6666666.28487.peg.4537	plasmid	lankamycin synthase, modules 5 and 6
fig|6666666.28487.peg.4577	plasmid	phosphate transport system ATP-binding protein
fig|6666666.28487.peg.4579	plasmid	phosphate transport system permease protein
fig|6666666.28487.peg.4603	virus	putative phosphoribosyl formylglycinamidine (FGAM) synthase II
fig|6666666.28487.peg.4689	plasmid	ATPase involved in chromosome partitioning-like protein
fig|6666666.28487.peg.4706	plasmid	type II methyltransferase
fig|6666666.28487.peg.4737	plasmid	D-cysteine desulfhydrase
fig|6666666.28487.peg.4889	plasmid	probable serine/threonine protein kinase
fig|6666666.28487.peg.4975	plasmid	AAA ATPase, central domain protein
fig|6666666.28487.peg.4978	plasmid	copper-translocating P-type ATPase
fig|6666666.28487.peg.4979	plasmid	putative protein of unknown function
fig|6666666.28487.peg.4980	plasmid	YHS domain protein
fig|6666666.28487.peg.4982	plasmid	copper-translocating P-type ATPase
fig|6666666.28487.peg.4985	plasmid	heavy metal-associated domain protein
fig|6666666.28487.peg.4987	plasmid	helix-turn-helix- domain containing protein, AraC type
fig|6666666.28487.peg.4989	plasmid	regulatory protein, MerR
fig|6666666.28487.peg.4990	plasmid	mercuric reductase
fig|6666666.28487.peg.4996	plasmid	hypothetical protein
fig|6666666.28487.peg.4997	plasmid	ErfK/YbiS/YcfS/YnhG family protein
fig|6666666.28487.peg.4998	plasmid	NLP/P60 protein
fig|6666666.28487.peg.5000	plasmid	putative transporter
fig|6666666.28487.peg.5001	plasmid	putative pyridoxal-phosphate dependent enzyme
fig|6666666.28487.peg.5003	plasmid	peptidase M48, Ste24p
fig|6666666.28487.peg.5004	plasmid	transcriptional repressor, CopY family
fig|6666666.28487.peg.5005	plasmid	NLP/P60 protein
fig|6666666.28487.peg.5007	plasmid	two component transcriptional regulator, winged helix family
fig|6666666.28487.peg.5008	plasmid	integral membrane sensor signal transduction histidine kinase
fig|6666666.28487.peg.5009	plasmid	protein of unknown function DUF305
fig|6666666.28487.peg.5011	plasmid	DSBA oxidoreductase

fig|6666666.28487.peg.5012	plasmid	hypothetical protein
fig|6666666.28487.peg.5014	plasmid	putative cytochrome c biogenesis protein ResB
fig|6666666.28487.peg.5015	plasmid	cytochrome c biogenesis protein, transmembrane region
fig|6666666.28487.peg.5016	plasmid	copper resistance D domain-containing protein
fig|6666666.28487.peg.5017	plasmid	regulatory protein, ArsR
fig|6666666.28487.peg.5018	plasmid	cadA protein
fig|6666666.28487.peg.5019	plasmid	hypothetical protein
fig|6666666.28487.peg.5020	plasmid	putative lipoprotein LppW
fig|6666666.28487.peg.5022	plasmid	Cytochrome-c oxidase
fig|6666666.28487.peg.5023	plasmid	redoxin domain protein
fig|6666666.28487.peg.5025	plasmid	cytochrome c oxidase, subunit III
fig|6666666.28487.peg.5027	plasmid	transcriptional repressor, CopY family
fig|6666666.28487.peg.5037	plasmid	membrane protein
fig|6666666.28487.peg.5045	plasmid	phosphoglucomutase, alpha-D-glucose phosphate-specific
fig|6666666.28487.peg.5071	plasmid	cytidine deaminase
fig|6666666.28487.peg.5112	plasmid	oxidoreductase
fig|6666666.28487.peg.5174	plasmid	hypothetical protein
fig|6666666.28487.peg.5177	plasmid	pyruvate, phosphate dikinase
fig|6666666.28487.peg.5183	plasmid	IS sequence
fig|6666666.28487.peg.5184	plasmid	IS sequence
fig|6666666.28487.peg.5186	plasmid	carbamate kinase
fig|6666666.28487.peg.5187	plasmid	ornithine carbamoyltransferase
fig|6666666.28487.peg.5212	plasmid	probable pyruvate dehydrogenase, E1 component, alpha subunit
fig|6666666.28487.peg.5213	plasmid	probable pyruvate dehydrogenase, E1 component, beta subunit
fig|6666666.28487.peg.5240	plasmid	putative relaxase
fig|6666666.28487.peg.5247	plasmid	hypothetical protein
fig|6666666.28487.peg.5248	plasmid	Cobyrinic acid a,c-diamide synthase
fig|6666666.28487.peg.5259	plasmid	putative serine recombinase
fig|6666666.28487.peg.5261	prophage	plasmid maintenance system killer
fig|6666666.28487.peg.5298	plasmid	pyruvate carboxylase
fig|6666666.28487.peg.5315	plasmid	malate dehydrogenase (oxaloacetate decarboxylating)
fig|6666666.28487.peg.5376	plasmid	thioesterase superfamily protein
fig|6666666.28487.peg.5377	plasmid	putative transcriptional regulator, TetR family
fig|6666666.28487.peg.5409	plasmid	NADH ubiquinone oxidoreductase, 20 kDa subunit
fig|6666666.28487.peg.5428	plasmid	DNA-directed RNA polymerase, beta' subunit
fig|6666666.28487.peg.5439	plasmid	enoyl-CoA hydratase
fig|6666666.28487.peg.5445	plasmid	ribosomal protein S12
fig|6666666.28487.peg.5448	plasmid	translation elongation factor Tu
fig|6666666.28487.peg.5453	plasmid	ornithine aminotransferase
fig|6666666.28487.peg.5458	plasmid	copper-translocating P-type ATPase
fig|6666666.28487.peg.5557	plasmid	probable thioredoxin

fig|6666666.28487.peg.5561	plasmid	transposase IS3/IS911 family protein
fig|6666666.28487.peg.5562	plasmid	integrase, catalytic region
fig|6666666.28487.peg.5567	plasmid	hypothetical protein
fig|6666666.28487.peg.5613	plasmid	phosphoenolpyruvate carboxykinase (GTP)
fig|6666666.28487.peg.5618	plasmid	membrane protein, MmpL family
fig|6666666.28487.peg.5645	plasmid	chlorite dismutase
fig|6666666.28487.peg.5663	prophage	phage portal protein, SPP1 Gp6-like
fig|6666666.28487.peg.5724	plasmid	protein of unknown function DUF305
fig|6666666.28487.peg.5725	plasmid	histidine kinase
fig|6666666.28487.peg.5726	plasmid	two component transcriptional regulator, winged helix family
fig|6666666.28487.peg.5728	plasmid	NLP/P60 protein
fig|6666666.28487.peg.5729	plasmid	heavy metal-associated domain protein
fig|6666666.28487.peg.5731	plasmid	copper-translocating P-type ATPase
fig|6666666.28487.peg.5800	plasmid	putative transcriptional regulator, ArsR family
fig|6666666.28487.peg.5813	plasmid	probable transposase
fig|6666666.28487.peg.5837	plasmid	hypothetical protein
fig|6666666.28487.peg.5838	plasmid	putative transcriptional regulatory protein
fig|6666666.28487.peg.5839	plasmid	hypothetical protein
fig|6666666.28487.peg.5840	plasmid	fatty acid hydroxylase
fig|6666666.28487.peg.5842	plasmid	hypothetical protein
fig|6666666.28487.peg.5843	plasmid	hypothetical protein
fig|6666666.28487.peg.5908	plasmid	ErfK/YbiS/YcfS/YnhG family protein
fig|6666666.28487.peg.5912	plasmid	protein of unknown function DUF156
fig|6666666.28487.peg.5942	plasmid	transposase IS3/IS911 family protein
fig|6666666.28487.peg.5951	plasmid	hypothetical protein
fig|6666666.28487.peg.5952	plasmid	NLP/P60 protein
fig|6666666.28487.peg.5953	plasmid	ErfK/YbiS/YcfS/YnhG family protein
fig|6666666.28487.peg.5954	plasmid	NLP/P60 protein
fig|6666666.28487.peg.5995	plasmid	IS21 family element, transposase istB

Supplementary Table 2. List of putative Toxin antitoxin loci in UM_TJL.

RAST Predicted CDS	TADB ID	NCBI Acc.	Gene	Organism
fig|6666666.28487.peg.318	TADB_10135064	NC_013124	Afer_2015	Acidimicrobium ferrooxidans DSM 10331
fig|6666666.28487.peg.319	TADB_5491310	NC_008726	Mvan_2930	Mycobacterium vanbaalenii PYR-1
fig|6666666.28487.peg.320	TADB_5289351	NC_008596	MSMEG_4447	Mycobacterium smegmatis str. MC2 155
fig|6666666.28487.peg.459	TADB_5490498	NC_008726	Mvan_2637	Mycobacterium vanbaalenii PYR-1
fig|6666666.28487.peg.460	TADB_5490496	NC_008726	Mvan_2636	Mycobacterium vanbaalenii PYR-1
fig|6666666.28487.peg.487	TADB_11459764	NC_013946	Mrub_0888	Meiothermus ruber DSM 1279
fig|6666666.28487.peg.535	TADB_6050637	NC_009338	Mflv_0787	Mycobacterium gilvum PYR-GCK
fig|6666666.28487.peg.536	TADB_6050635	NC_009338	Mflv_0786	Mycobacterium gilvum PYR-GCK
fig|6666666.28487.peg.598	TADB_7765790	NC_010397	MAB_3069c	Mycobacterium abscessus
fig|6666666.28487.peg.988	TADB_5485142	NC_008726	Mvan_0694	Mycobacterium vanbaalenii PYR-1
fig|6666666.28487.peg.989	TADB_5485140	NC_008726	Mvan_0693	Mycobacterium vanbaalenii PYR-1
fig|6666666.28487.peg.1052	TADB_10438169	NC_014158	Tpau_0489	Tsukamurella paurometabola DSM 20162
fig|6666666.28487.peg.1171	TADB_110937	NC_000962	Rv0910	Mycobacterium tuberculosis H37Rv
fig|6666666.28487.peg.1483	TADB_7765788	NC_010397	MAB_3068c	Mycobacterium abscessus
fig|6666666.28487.peg.2066	TADB_5491335	NC_008726	Mvan_2940	Mycobacterium vanbaalenii PYR-1
fig|6666666.28487.peg.2067	TADB_5491333	NC_008726	Mvan_2939	Mycobacterium vanbaalenii PYR-1
fig|6666666.28487.peg.4303	TADB_9001736	NC_012522	ROP_50820	Rhodococcus opacus B4
fig|6666666.28487.peg.4304	TADB_4741901	NC_008268	RHA1_ro05020	Rhodococcus jostii RHA1
fig|6666666.28487.peg.4326	TADB_7764005	NC_010397	MAB_2386	Mycobacterium abscessus
fig|6666666.28487.peg.4495	TADB_6050626	NC_009338	Mflv_0783	Mycobacterium gilvum PYR-GCK
fig|6666666.28487.peg.4684	TADB_6050623	NC_009338	Mflv_0782	Mycobacterium gilvum PYR-GCK
fig|6666666.28487.peg.4751	TADB_5854354	NC_009077	Mjls_0384	Mycobacterium sp. JLS
fig|6666666.28487.peg.4752	TADB_5854352	NC_009077	Mjls_0383	Mycobacterium sp. JLS
fig|6666666.28487.peg.5260	TADB_6345443	NC_009483	Gura_1216	Geobacter uraniireducens Rf4
fig|6666666.28487.peg.5261	TADB_7642000	NC_010296	MAE_38920	Microcystis aeruginosa NIES-843
fig|6666666.28487.peg.5838	TADB_5865734	NC_009077	Mjls_4572	Mycobacterium sp. JLS
fig|6666666.28487.peg.5839	TADB_5865644	NC_009077	Mjls_4540	Mycobacterium sp. JLS
fig|6666666.28487.peg.5942	TADB_10135064	NC_013124	Afer_2015	Acidimicrobium ferrooxidans DSM 10331

Supplementary Table 3. Information on mycobacterial strains used for comparisons

Strain	Accession. No	No. RAST Predicted CDS	Reported Virulence Status
M. iranicum UM_TJL	NZ_AUWT00000000	5995	+
M. gilvum	NC_009338	5410	-
M. vanbaalenii	NC_008726	6239	-
M. vaccae	ALQA00000000	5911	-
M. chubuense	NC_018027	5924	-
M. sp. KMS	NC_008705	5553	-
M. sp. JLS	NC_009077	5881	-
M. rhodesiae	NC_016604	6374	+
M. sp. MCS	NC_008146	5506	-
M. tusciae	AGJJ00000000	7174	+
M. phlei	AJFJ00000000	5568	+
M. mageritense	AGSZ00000000	6238	+
M. leprae	NC_002677	5693	++
M. fortuitum	ALQB00000000	6077	+
M. smegmatis	NC_008596	6775	+
M. hassiacum	AMRA00000000	4799	+
M. thermoresistibile	AGVE00000000	4634	+
M. canettii	NC_019952	4375	++
M. xenopi	AJFI00000000	4375	+
M. africanum	NC_015758	4321	++
M. tuberculosis	NC_000962	4368	++
M. avium	NC_002944	4594	+

M. ulcerans	NC_008611	5518	+
M. parascrofulaceum	ADNV00000000	6044	+
M. marinum	NC_010612	5777	+
M. indicus pranii	NC_018612	5278	-
M. kansasii	NC_022663	5958	+
M. intracellulare	NC_016946	5099	+
M. colombiense	AFVW00000000	5291	+
M. abscessus	NC_010397	5024	+

++, primary pathogen of high virulence;

+ opportunistic pathogen of intermediate virulence;

- mycobacteria of low virulence, infrequently associated with human infections

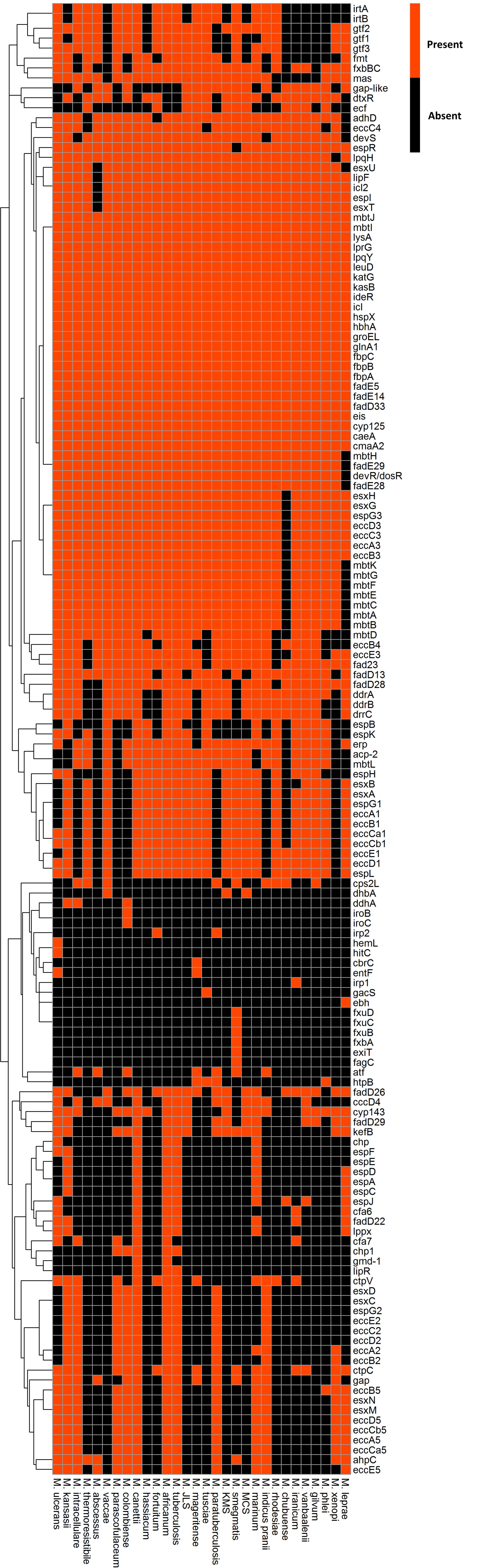
Present
Absent
irtA
irtB
gtf2
gtf1
gtf3
fmt
fxbBC
mas
gap-like
dtxR
ecf
adhD
eccC4
devS
espR
lpqH
esxU
lipF
icl2
espI
esxT
mbtJ
mbtI
lysA
lprG
lpqY
leuD
katG
kasB
ideR
icl
hspX
hbhA
groEL
glnA1
fbpC
fbpB
fbpA
fadE5
fadE14
fadD33
eis
cyp125
caeA
cmaA2
mbtH
fadE29
devR/dosR
fadE28
esxH
esxG
espG3
eccD3
eccC3
eccA3
eccB3
mbtK
mbtG
mbtF
mbtE
mbtC
mbtA
mbtB
mbtD
eccB4
eccE3
fad23
fadD13
fadD28
ddrA
ddrB
drrC
espB
espK
erp
acp-2
mbtL
espH
esxB
esxA
espG1
eccA1
eccB1
eccCa1
eccCb1
eccE1
eccD1
espL
cps2L
dhbA
ddhA
iroB
iroC
irp2
hemL
hitC
cbrC
entF
irp1
gacS
ebh
fxuD
fxuC
fxuB
fxbA
exiT
fagC
atf
htpB
fadD26
cccD4
cyp143
fadD29
kefB
chp
espF
espE
espD
espA
espC
espJ
cfa6
fadD22
lppx
cfa7
chp1
gmd-1
lipR
ctpV
esxD
esxC
espG2
eccE2
eccC2
eccD2
eccA2
eccB2
ctpC
gap
eccB5
esxN
esxM
eccD5
eccCb5
eccA5
eccCa5
ahpC
eccE5
M. ulcerans
M. kansasii
M. intracellulare
M. thermoresistibile
M. abscessus
M. vaccae
M. parascrofulaceum
M. colombiense
M. canettii
M. hassiacum
M. fortuitum
M. africanum
M. tuberculosis
M. JLS
M. magaritense
M. tusciae
M. paratuberculosis
M. KMS
M. smegmatis
M. MCS
M. marinum
M. indicus pranii
M. rhodesiae
M. chubuense
M. iranicum
M. vanbaalenii
M. gilvum
M. phlei
M. xenopi
M. leprae

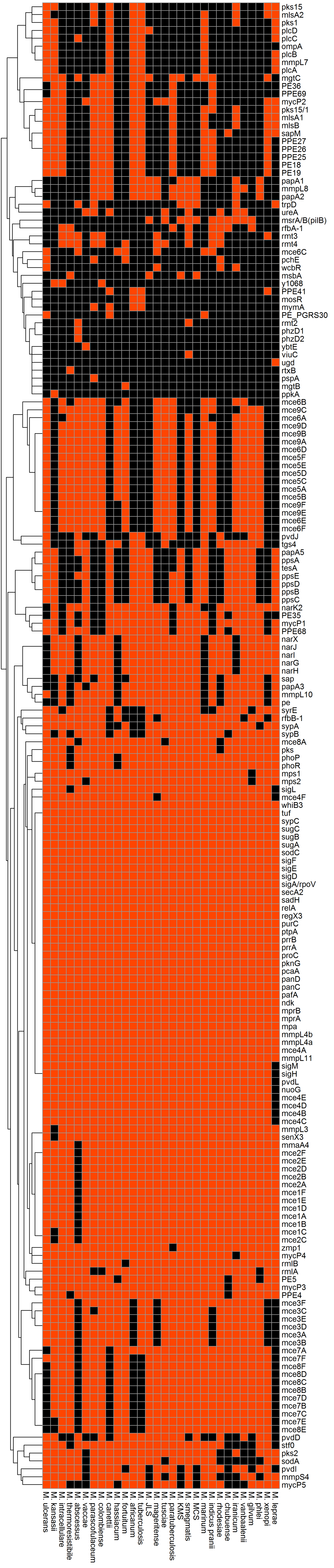

Figure S7. Illustration of pathogenomics for 30 mycobacteria. The presence of individual virulence factors is indicated with orange color. Based on the figure, most virulence factors appear as accessory genes in the mycobacterium genus.

Supplementary Table 4. RAST annotated ORFs information

Contig_ID	Feature_ID	Type	Start	Stop	Strand	Function
contig_1	fig|6666666.28487.peg.1	Protein	92	490	+	Possible membrane protein
contig_1	fig|6666666.28487.peg.2	Protein	505	1893	+	Oxidoreductase, FAD-binding protein
contig_1	fig|6666666.28487.peg.3	Protein	1894	2676	+	3-oxoacyl-[acyl-carrier protein] reductase paralog (EC 1.1.1.100)
contig_1	fig|6666666.28487.peg.4	Protein	2706	4538	+	putative membrane protein
contig_1	fig|6666666.28487.peg.5	Protein	4543	7770	+	INTEGRAL MEMBRANE INDOLYLACETYLINOSITOL ARABINOSYLTRANSFERASE EMBC (ARABINOSYLINDOLYLACETYLINOSITOL SYNTHASE)
contig_1	fig|6666666.28487.peg.6	Protein	7823	11089	+	Probable arabinosyltransferase A (EC 2.4.2.-)
contig_1	fig|6666666.28487.peg.7	Protein	11122	14331	+	INTEGRAL MEMBRANE INDOLYLACETYLINOSITOL ARABINOSYLTRANSFERASE EMBC (ARABINOSYLINDOLYLACETYLINOSITOL SYNTHASE)
contig_1	fig|6666666.28487.peg.8	Protein	14328	15206	+	putative beta-lactamase
contig_1	fig|6666666.28487.peg.9	Protein	16606	15203	-	Serine/threonine protein kinase (EC 2.7.11.1)
contig_1	fig|6666666.28487.peg.10	Protein	17510	16608	-	FIG00825558: hypothetical protein
contig_1	fig|6666666.28487.peg.11	Protein	19130	17556	-	Propionyl-CoA carboxylase beta chain (EC 6.4.1.3)
contig_1	fig|6666666.28487.peg.12	Protein	24541	19127	-	Probable polyketide synthase, similar to many. e.g. gp|M63676|SERERYAA_1 S.erythraea first ORF of eryA gene, involved in complex polyketide formation in erythromycin biosynthesis.
contig_1	fig|6666666.28487.peg.13	Protein	26486	24597	-	Long-chain-fatty-acid--CoA ligase (EC 6.2.1.3)
contig_1	fig|6666666.28487.peg.14	Protein	27633	26623	-	hypothetical protein Rv3802c

contig_1	fig|6666666.28487.peg.15	Protein	28107	27652	-	FIG00820592: hypothetical protein
contig_1	fig|6666666.28487.peg.16	Protein	29057	28176	-	Antigen 85-C precursor (85C) (Antigen 85 complex C) (Ag85C) (Mycolyl transferase 85C) (EC 2.3.1.-)
contig_1	fig|6666666.28487.peg.17	Protein	30250	29201	-	Antigen 85-A precursor (85A) (Antigen 85 complex A) (Ag85A) (Mycolyl transferase 85A) (EC 2.3.1.-)
contig_1	fig|6666666.28487.peg.18	Protein	32476	30503	-	hypothetical protein Rv3805c
contig_1	fig|6666666.28487.peg.19	Protein	33407	32454	-	POSSIBLE CONSERVED INTEGRAL MEMBRANE PROTEIN
contig_1	fig|6666666.28487.peg.20	Protein	33951	33412	-	FIG008913: Membrane-associated phospholipid phosphatase
contig_1	fig|6666666.28487.peg.21	Protein	35884	33944	-	Galactofuranosyl transferase (EC 2.-.-.-)
contig_1	fig|6666666.28487.peg.22	Protein	37095	35881	-	UDP-galactopyranose mutase (EC 5.4.99.9)
contig_1	fig|6666666.28487.peg.23	Protein	37353	38294	+	Exported repetitive protein precursor (Cell surface protein pirG) (EXP53)
contig_1	fig|6666666.28487.peg.24	Protein	38426	40039	+	Putative uncharacterized protein BCG_3873
contig_1	fig|6666666.28487.peg.25	Protein	42735	40036	-	FIG00826446: hypothetical protein
contig_1	fig|6666666.28487.peg.26	Protein	43670	42858	-	Cof family hydrolase
contig_1	fig|6666666.28487.peg.27	Protein	44404	43667	-	1-acyl-sn-glycerol-3-phosphate acyltransferase (EC 2.3.1.51)
contig_1	fig|6666666.28487.peg.28	Protein	45164	44409	-	1-acyl-sn-glycerol-3-phosphate acyltransferase (EC 2.3.1.51)
contig_1	fig|6666666.28487.peg.29	Protein	45228	45920	+	aminoglycoside 3'-phosphotransferase
contig_1	fig|6666666.28487.peg.30	Protein	45971	47524	+	Rieske (2Fe-2S) domain protein
contig_1	fig|6666666.28487.peg.31	Protein	47526	47876	+	Putative uncharacterized protein BCG_3881

contig_1	fig|6666666.28487.peg.32	Protein	49132	47873	-	Seryl-tRNA synthetase (EC 6.1.1.11)
contig_1	fig|6666666.28487.peg.33	Protein	49254	50642	+	PROBABLE CONSERVED MEMBRANE PROTEIN
contig_1	fig|6666666.28487.peg.34	Protein	50647	50997	+	FIG00820873: hypothetical protein
contig_1	fig|6666666.28487.peg.35	Protein	51057	51266	+	FIG00824423: hypothetical protein
contig_1	fig|6666666.28487.peg.36	Protein	51938	51246	-	putative phosphoglycerate mutase
contig_1	fig|6666666.28487.peg.37	Protein	52855	51935	-	Prephenate dehydratase (EC 4.2.1.51)
contig_1	fig|6666666.28487.peg.38	Protein	52968	53768	+	FIG00998479: hypothetical protein
contig_1	fig|6666666.28487.peg.39	Protein	53806	54588	+	Putative conserved integral membrane protein
contig_1	fig|6666666.28487.peg.40	Protein	54922	55878	+	Cell envelope-associated transcriptional attenuator LytR-CpsA-Psr, subfamily A1 (as in PMID19099556)
contig_1	fig|6666666.28487.peg.41	Protein	55957	56538	+	Ferritin
contig_1	fig|6666666.28487.peg.42	Protein	57374	56553	-	Glycerophosphoryl diester phosphodiesterase (EC 3.1.4.46)
contig_1	fig|6666666.28487.peg.43	Protein	57984	57379	-	PROBABLE CONSERVED TRANSMEMBRANE PROTEIN
contig_1	fig|6666666.28487.peg.44	Protein	58783	58424	-	Rhodanese-related sulfurtransferase
contig_1	fig|6666666.28487.peg.45	Protein	59173	59331	+	FIG00822287: hypothetical protein
contig_1	fig|6666666.28487.peg.46	Protein	59617	59315	-	putative phage repressor
contig_1	fig|6666666.28487.peg.47	Protein	59686	60120	+	Nickel-dependent superoxide dismutase (EC 1.15.1.1)
contig_1	fig|6666666.28487.peg.48	Protein	60302	60835	+	FIG017534: hypothetical protein
contig_1	fig|6666666.28487.peg.49	Protein	61277	61690	+	ESX-1 secreted protein regulator EspR
contig_1	fig|6666666.28487.peg.50	Protein	62895	61756	-	FIG00822573: hypothetical protein

contig_1	fig|6666666.28487.peg.51	Protein	63383	62892	-	FIG00820679: hypothetical protein
contig_1	fig|6666666.28487.peg.52	Protein	64371	63445	-	hypothetical protein
contig_1	fig|6666666.28487.peg.53	Protein	64588	65244	+	FIG022958: hypothetical protein
contig_1	fig|6666666.28487.peg.54	Protein	65286	65759	+	hypothetical protein
contig_1	fig|6666666.28487.peg.55	Protein	65796	66353	+	Copper resistance protein CopC precursor
contig_1	fig|6666666.28487.peg.56	Protein	66350	67270	+	Copper resistance protein D
contig_1	fig|6666666.28487.peg.57	Protein	67313	67768	+	conserved hypothetical protein
contig_1	fig|6666666.28487.peg.58	Protein	67758	68252	+	Ribonuclease E inhibitor RraA
contig_1	fig|6666666.28487.peg.59	Protein	69734	68265	-	monooxygenase, flavin-binding family
contig_1	fig|6666666.28487.peg.60	Protein	69820	70452	+	Transcriptional repressor EthR, TetR family
contig_1	fig|6666666.28487.peg.61	Protein	70477	71685	+	DNA polymerase IV (EC 2.7.7.7)
contig_1	fig|6666666.28487.peg.62	Protein	71728	72543	+	hypothetical protein
contig_1	fig|6666666.28487.peg.63	Protein	73547	72540	-	DNA-dependent DNA polymerase beta chain
contig_1	fig|6666666.28487.peg.64	Protein	73643	74773	+	hypothetical protein
contig_1	fig|6666666.28487.peg.65	Protein	75083	75547	+	hypothetical protein
contig_1	fig|6666666.28487.peg.66	Protein	75544	76974	+	hypothetical protein
contig_1	fig|6666666.28487.peg.67	Protein	77536	76967	-	FIG00823427: hypothetical protein
contig_1	fig|6666666.28487.peg.68	Protein	77551	78468	+	Coenzyme F420-dependent oxidoreductase
contig_1	fig|6666666.28487.peg.69	Protein	78688	78548	-	FIG00820244: hypothetical protein

contig_1	fig|6666666.28487.peg.70	Protein	80293	78968	-	Glutamate synthase [NADPH] small chain (EC 1.4.1.13)
contig_1	fig|6666666.28487.peg.71	Protein	80267	80449	+	hypothetical protein
contig_1	fig|6666666.28487.peg.72	Protein	85028	80427	-	Glutamate synthase [NADPH] large chain (EC 1.4.1.13)
contig_1	fig|6666666.28487.peg.73	Protein	85542	86396	+	FIG00821742: hypothetical protein
contig_1	fig|6666666.28487.peg.74	Protein	87003	86458	-	Non-specific DNA-binding protein Dps / Iron-binding ferritin-like antioxidant protein / Ferroxidase (EC 1.16.3.1)
contig_1	fig|6666666.28487.peg.75	Protein	87994	87083	-	FIG00822261: hypothetical protein
contig_1	fig|6666666.28487.peg.76	Protein	88378	88004	-	FIG00822464: hypothetical protein
contig_1	fig|6666666.28487.peg.77	Protein	89782	88400	-	Glycine/D-amino acid oxidases (deaminating)
contig_1	fig|6666666.28487.peg.78	Protein	90890	89847	-	Dihydroflavonol-4-reductase (EC 1.1.1.219)
contig_1	fig|6666666.28487.peg.79	Protein	91393	90887	-	FIG00821695: hypothetical protein
contig_1	fig|6666666.28487.peg.80	Protein	91806	91438	-	putative large secreted protein
contig_1	fig|6666666.28487.peg.81	Protein	91918	92394	+	Peptide methionine sulfoxide reductase MsrB (EC 1.8.4.12)
contig_1	fig|6666666.28487.peg.82	Protein	92391	92903	+	Peptide methionine sulfoxide reductase MsrA (EC 1.8.4.11)
contig_1	fig|6666666.28487.peg.83	Protein	94198	92900	-	Cytochrome P450 138
contig_1	fig|6666666.28487.peg.84	Protein	94303	94899	+	Transcriptional regulator, TetR family
contig_1	fig|6666666.28487.peg.85	Protein	95480	94911	-	Transcriptional regulator, TetR family
contig_1	fig|6666666.28487.peg.86	Protein	95555	96223	+	putative secreted protein
contig_1	fig|6666666.28487.peg.87	Protein	97134	96220	-	FIG00821966: hypothetical protein
contig_1	fig|6666666.28487.peg.88	Protein	98002	97277	-	putative methyltransferase

contig_1	fig|6666666.28487.peg.89	Protein	99231	97996	-	Glycosyltransferase
contig_1	fig|6666666.28487.peg.90	Protein	99872	99417	-	Pyridoxine 5'-phosphate oxidase, Rv1155
contig_1	fig|6666666.28487.peg.91	Protein	99920	100216	+	Partial REP13E12 repeat protein
contig_1	fig|6666666.28487.peg.92	Protein	100815	100225	-	hypothetical protein
contig_1	fig|6666666.28487.peg.93	Protein	100806	102212	+	Acyl-CoA dehydrogenase
contig_1	fig|6666666.28487.peg.94	Protein	104683	102215	-	Putative membrane protein
contig_1	fig|6666666.28487.peg.95	Protein	104815	106671	+	FIG00821413: hypothetical protein
contig_1	fig|6666666.28487.peg.96	Protein	106686	106817	+	FIG00822048: hypothetical protein
contig_1	fig|6666666.28487.peg.97	Protein	106943	107233	+	FIG00826438: hypothetical protein
contig_1	fig|6666666.28487.peg.98	Protein	107230	108516	+	FIG00821438: hypothetical protein
contig_1	fig|6666666.28487.peg.99	Protein	108549	109208	+	FIG00824785: hypothetical protein
contig_1	fig|6666666.28487.peg.100	Protein	109205	109759	+	Nicotinamidase (EC 3.5.1.19)
contig_1	fig|6666666.28487.peg.101	Protein	109828	111975	+	Glycogen debranching enzyme (EC 3.2.1.-)
contig_1	fig|6666666.28487.peg.102	Protein	113500	112199	-	uncharacterized protein probably involved in trehalose biosynthesis
contig_1	fig|6666666.28487.peg.103	Protein	114596	113535	-	FIG00825702: hypothetical protein
contig_1	fig|6666666.28487.peg.104	Protein	114753	115565	+	FIG00821808: hypothetical protein
contig_1	fig|6666666.28487.peg.105	Protein	115579	116148	+	Orotate phosphoribosyltransferase (EC 2.4.2.10)
contig_1	fig|6666666.28487.peg.106	Protein	117268	116237	-	Sulfate and thiosulfate import ATP-binding protein CysA (EC 3.6.3.25)
contig_1	fig|6666666.28487.peg.107	Protein	118044	117265	-	binding-protein-dependent transport systems inner membrane

						component
contig_1	fig|6666666.28487.peg.108	Protein	118903	118058	-	ABC transporter permease protein
contig_1	fig|6666666.28487.peg.109	Protein	120038	118911	-	Ferric iron ABC transporter, iron-binding protein
contig_1	fig|6666666.28487.peg.110	Protein	120147	120584	+	pyridoxamine 5'-phosphate oxidase-related, FMN-binding
contig_1	fig|6666666.28487.peg.111	Protein	120670	121236	+	RNA polymerase sigma-E factor
contig_1	fig|6666666.28487.peg.112	Protein	122120	121212	-	MCE-family protein Mce1A
contig_1	fig|6666666.28487.peg.113	Protein	122432	122166	-	FIG00830672: hypothetical protein
contig_1	fig|6666666.28487.peg.114	Protein	123733	122429	-	L-Proline/Glycine betaine transporter ProP
contig_1	fig|6666666.28487.peg.115	Protein	124608	123820	-	Transcription termination factor Rho
contig_1	fig|6666666.28487.peg.116	Protein	124629	126557	+	Kup system potassium uptake protein
contig_1	fig|6666666.28487.peg.117	Protein	128909	126540	-	Maltose phosphorylase (EC 2.4.1.8) / Trehalose phosphorylase (EC 2.4.1.64)
contig_1	fig|6666666.28487.peg.118	Protein	129619	128906	-	Beta-phosphoglucomutase (EC 5.4.2.6)
contig_1	fig|6666666.28487.peg.119	Protein	129662	130528	+	FIG00826463: hypothetical protein
contig_1	fig|6666666.28487.peg.120	Protein	131385	130525	-	FIG056164: rhomboid family serine protease
contig_1	fig|6666666.28487.peg.121	Protein	131794	131387	-	Glyoxalase family protein
contig_1	fig|6666666.28487.peg.122	Protein	132459	131791	-	nitroreductase family protein
contig_1	fig|6666666.28487.peg.123	Protein	132440	132559	+	hypothetical protein
contig_1	fig|6666666.28487.peg.124	Protein	132799	133200	+	POSSIBLE EXPORTED PROTEIN
contig_1	fig|6666666.28487.peg.125	Protein	133343	133780	+	FIG00822299: hypothetical protein

contig_1	fig|6666666.28487.peg.126	Protein	133879	134973	+	Beta-lactamase
contig_1	fig|6666666.28487.peg.127	Protein	136072	134936	-	FAD-dependent pyridine nucleotide-disulphide oxidoreductase
contig_1	fig|6666666.28487.peg.128	Protein	136941	136069	-	FIG005069: Hypothetical protein
contig_1	fig|6666666.28487.peg.129	Protein	137692	136934	-	glutamine cyclotransferase
contig_1	fig|6666666.28487.peg.130	Protein	137756	138916	+	Sorbitol dehydrogenase (EC 1.1.1.14)
contig_1	fig|6666666.28487.peg.131	Protein	139347	138913	-	FIG00822892: hypothetical protein
contig_1	fig|6666666.28487.peg.132	Protein	140552	139362	-	2-polyprenyl-6-methoxyphenol hydroxylase and related FAD-dependent oxidoreductases
contig_1	fig|6666666.28487.peg.133	Protein	141531	140545	-	2-amino-3-carboxymuconate-6-semialdehyde decarboxylase (EC 4.1.1.45)
contig_1	fig|6666666.28487.peg.134	Protein	142274	141528	-	ABC transporter related
contig_1	fig|6666666.28487.peg.135	Protein	143064	142288	-	binding-protein-dependent transport systems inner membrane component
contig_1	fig|6666666.28487.peg.136	Protein	143888	143061	-	taurine ABC transporter, permease protein, putative
contig_1	fig|6666666.28487.peg.137	Protein	144922	143885	-	hypothetical protein
contig_1	fig|6666666.28487.peg.138	Protein	150558	146656	-	ATP-dependent helicase hrpA (hrpA)
contig_1	fig|6666666.28487.peg.139	Protein	150933	150583	-	FIG00822548: hypothetical protein
contig_1	fig|6666666.28487.peg.140	Protein	151025	151669	+	hypothetical protein
contig_1	fig|6666666.28487.peg.141	Protein	151743	152219	+	hypothetical protein
contig_1	fig|6666666.28487.peg.142	Protein	153114	152221	-	FIG00830858: hypothetical protein

contig_1	fig|6666666.28487.peg.143	Protein	153264	154418	+	FIG00829057: hypothetical protein
contig_1	fig|6666666.28487.peg.144	Protein	154429	155886	+	Selenoprotein O and cysteine-containing homologs
contig_1	fig|6666666.28487.peg.145	Protein	155909	157333	+	Wax ester synthase/acyl-CoA:diacylglycerol acyltransferase
contig_1	fig|6666666.28487.peg.146	Protein	157625	157344	-	protein of unknown function DUF222
contig_10	fig|6666666.28487.peg.147	Protein	135	935	+	Short chain dehydrogenase
contig_10	fig|6666666.28487.peg.148	Protein	935	1774	+	hypothetical protein
contig_10	fig|6666666.28487.peg.149	Protein	1767	2399	+	Transcriptional regulator, TetR family
contig_10	fig|6666666.28487.peg.150	Protein	2399	3694	+	Alcohol dehydrogenase (EC 1.1.1.1)
contig_10	fig|6666666.28487.peg.151	Protein	3701	4147	+	putative 4-hydroxybenzoyl-CoA thioesterase
contig_10	fig|6666666.28487.peg.152	Protein	4144	4647	+	Transcriptional regulator, MarR family
contig_10	fig|6666666.28487.peg.153	Protein	4804	5109	+	conserved hypothetical protein
contig_10	fig|6666666.28487.peg.154	Protein	5770	5093	-	hypothetical protein
contig_10	fig|6666666.28487.peg.155	Protein	6120	5770	-	anti-sigma-factor antagonist
contig_10	fig|6666666.28487.peg.156	Protein	7184	6117	-	5-methyltetrahydrofolate--homocysteine methyltransferase (EC 2.1.1.13)
contig_10	fig|6666666.28487.peg.157	Protein	8767	7181	-	Stage II sporulation E family protein
contig_10	fig|6666666.28487.peg.158	Protein	9878	8895	-	Glycerophosphoryl diester phosphodiesterase (EC 3.1.4.46)
contig_10	fig|6666666.28487.peg.159	Protein	11014	9887	-	Putative uncharacterized protein BCG_3858
contig_10	fig|6666666.28487.peg.160	Protein	11125	12426	+	Siderophore biosynthesis diaminobutyrate--2-oxoglutarate aminotransferase (EC 2.6.1.76)

contig_10	fig|6666666.28487.peg.161	Protein	12647	13798	+	hypothetical protein
contig_10	fig|6666666.28487.peg.162	Protein	14985	13795	-	transporter, putative
contig_10	fig|6666666.28487.peg.163	Protein	15452	15036	-	Transcriptional regulator, MarR family
contig_10	fig|6666666.28487.peg.164	Protein	15956	15504	-	hypothetical protein
contig_10	fig|6666666.28487.peg.165	Protein	16584	16027	-	ThiJ/PfpI family protein
contig_10	fig|6666666.28487.peg.166	Protein	17518	16640	-	Polyphosphate kinase 2 (EC 2.7.4.1)
contig_10	fig|6666666.28487.peg.167	Protein	17554	17838	+	hypothetical protein
contig_10	fig|6666666.28487.peg.168	Protein	17825	18184	+	hypothetical protein
contig_10	fig|6666666.28487.peg.169	Protein	18228	18917	+	serine esterase, cutinase family
contig_10	fig|6666666.28487.peg.170	Protein	18996	19688	+	putative methyltransferase
contig_10	fig|6666666.28487.peg.171	Protein	19751	19882	+	zinc finger, CDGSH-type domain protein
contig_10	fig|6666666.28487.peg.172	Protein	20921	19887	-	FIG00827770: hypothetical protein
contig_10	fig|6666666.28487.peg.173	Protein	21111	22106	+	FIG00829233: hypothetical protein
contig_10	fig|6666666.28487.peg.174	Protein	22225	22383	+	hypothetical protein
contig_10	fig|6666666.28487.peg.175	Protein	22417	23133	+	Hypothetical nudix hydrolase YeaB
contig_10	fig|6666666.28487.peg.176	Protein	23308	23631	+	FIG00820518: hypothetical protein
contig_10	fig|6666666.28487.peg.177	Protein	24539	23628	-	FIG00829574: hypothetical protein
contig_10	fig|6666666.28487.peg.178	Protein	25817	24540	-	Potassium efflux system KefA protein / Small-conductance mechanosensitive channel
contig_10	fig|6666666.28487.peg.179	Protein	26540	25881	-	Uncharacterized protein conserved in bacteria

contig_10	fig|6666666.28487.peg.180	Protein	27076	26564	-	FIG00820318: hypothetical protein
contig_10	fig|6666666.28487.peg.181	Protein	27371	27111	-	FIG00823570: hypothetical protein
contig_10	fig|6666666.28487.peg.182	Protein	27527	28081	+	FIG00830811: hypothetical protein
contig_10	fig|6666666.28487.peg.183	Protein	29083	28082	-	Putative metal chaperone, involved in Zn homeostasis, GTPase of COG0523 family
contig_10	fig|6666666.28487.peg.184	Protein	29181	30806	+	hypothetical protein
contig_10	fig|6666666.28487.peg.185	Protein	31551	30772	-	Oxidoreductase, short chain dehydrogenase/reductase family
contig_10	fig|6666666.28487.peg.186	Protein	32030	31638	-	Phage tail fiber protein
contig_10	fig|6666666.28487.peg.187	Protein	32155	32766	+	conserved hypothetical protein
contig_10	fig|6666666.28487.peg.188	Protein	34157	32763	-	Adenosylmethionine-8-amino-7-oxononanoate aminotransferase (EC 2.6.1.62)
contig_100	fig|6666666.28487.peg.189	Protein	975	10	-	Mobile element protein
contig_101	fig|6666666.28487.rna.1	RNA	1530	12	-	Small Subunit Ribosomal RNA; ssuRNA; SSU rRNA
contig_104	fig|6666666.28487.peg.190	Protein	68	364	+	Mobile element protein
contig_104	fig|6666666.28487.peg.191	Protein	361	1227	+	Mobile element protein
contig_105	fig|6666666.28487.peg.192	Protein	53	607	+	Probable serine/threonine-protein kinase pknH (EC 2.7.11.1)
contig_106	fig|6666666.28487.peg.193	Protein	33	479	+	Partial REP13E12 repeat protein
contig_11	fig|6666666.28487.peg.194	Protein	70	372	+	Urease gamma subunit (EC 3.5.1.5)
contig_11	fig|6666666.28487.peg.195	Protein	382	717	+	Urease beta subunit (EC 3.5.1.5)
contig_11	fig|6666666.28487.peg.196	Protein	719	2470	+	Urease alpha subunit (EC 3.5.1.5)

contig_11	fig|6666666.28487.peg.197	Protein	2488	3132	+	Urease accessory protein UreF
contig_11	fig|6666666.28487.peg.198	Protein	3213	3893	+	Urease accessory protein UreG
contig_11	fig|6666666.28487.peg.199	Protein	3894	4520	+	Urease accessory protein UreD
contig_11	fig|6666666.28487.peg.200	Protein	5890	4517	-	NADH dehydrogenase (EC 1.6.99.3)
contig_11	fig|6666666.28487.peg.201	Protein	6975	6046	-	Oxidoreductase
contig_11	fig|6666666.28487.peg.202	Protein	7747	7076	-	short-chain dehydrogenase/reductase SDR
contig_11	fig|6666666.28487.peg.203	Protein	8149	9198	+	Alanine and proline-rich secreted protein Apa, fibronectin attachment protein
contig_11	fig|6666666.28487.peg.204	Protein	9336	9644	+	Transglycosylase-associated protein
contig_11	fig|6666666.28487.peg.205	Protein	9656	10663	+	Alcohol dehydrogenase (EC 1.1.1.1)
contig_11	fig|6666666.28487.peg.206	Protein	11441	10680	-	Possible membrane protein
contig_11	fig|6666666.28487.peg.207	Protein	13983	11479	-	Xylulose-5-phosphate phosphoketolase (EC 4.1.2.9); Fructose-6-phosphate phosphoketolase (EC 4.1.2.22)
contig_11	fig|6666666.28487.peg.208	Protein	14054	16405	+	hypothetical protein
contig_11	fig|6666666.28487.peg.209	Protein	16420	17895	+	Acetyl-CoA acetyltransferase
contig_11	fig|6666666.28487.peg.210	Protein	18885	17896	-	Malate dehydrogenase (EC 1.1.1.37)
contig_11	fig|6666666.28487.peg.211	Protein	19496	18900	-	FIG00824938: hypothetical protein
contig_11	fig|6666666.28487.peg.212	Protein	20009	19593	-	hypothetical protein
contig_11	fig|6666666.28487.peg.213	Protein	20378	20046	-	FIG00825063: hypothetical protein
contig_11	fig|6666666.28487.peg.214	Protein	22834	20483	-	ATP-dependent protease La (EC 3.4.21.53) Type I

contig_11	fig|6666666.28487.peg.215	Protein	22964	23704	+	Oxidoreductase
contig_11	fig|6666666.28487.peg.216	Protein	25084	24083	-	Antigen 85-C precursor (85C) (Antigen 85 complex C) (Ag85C) (Mycolyl transferase 85C) (EC 2.3.1.-)
contig_11	fig|6666666.28487.peg.217	Protein	25379	25816	+	hypothetical protein
contig_11	fig|6666666.28487.peg.218	Protein	27122	25821	-	Neopullulanase (EC 3.2.1.135)
contig_11	fig|6666666.28487.peg.219	Protein	27598	27119	-	Cytosine/adenosine deaminases
contig_11	fig|6666666.28487.peg.220	Protein	27632	28399	+	hypothetical protein
contig_11	fig|6666666.28487.peg.221	Protein	30027	28402	-	TPR domain protein
contig_11	fig|6666666.28487.peg.222	Protein	31093	30050	-	membrane protein, putative
contig_11	fig|6666666.28487.peg.223	Protein	31673	31086	-	Transcriptional regulator, TetR family
contig_11	fig|6666666.28487.peg.224	Protein	32038	32181	+	hypothetical protein
contig_11	fig|6666666.28487.peg.225	Protein	33815	32211	-	FIG00821727: hypothetical protein
contig_11	fig|6666666.28487.peg.226	Protein	34518	33826	-	Isochorismatase (EC 3.3.2.1)
contig_11	fig|6666666.28487.peg.227	Protein	35936	34515	-	Allophanate hydrolase (EC 3.5.1.54)
contig_11	fig|6666666.28487.peg.228	Protein	36643	36014	-	Transcriptional regulator, GntR family
contig_11	fig|6666666.28487.peg.229	Protein	38207	36795	-	Partial REP13E12 repeat protein
contig_11	fig|6666666.28487.peg.230	Protein	39738	38245	-	monooxygenase, FAD-binding
contig_11	fig|6666666.28487.peg.231	Protein	39881	40507	+	FIG00821978: hypothetical protein
contig_11	fig|6666666.28487.peg.232	Protein	42491	40479	-	Possible membrane protein
contig_11	fig|6666666.28487.peg.233	Protein	42523	43233	+	FIG00820028: hypothetical protein

contig_11	fig|6666666.28487.peg.234	Protein	43230	43982	+	Enoyl-CoA hydratase (EC 4.2.1.17)
contig_11	fig|6666666.28487.peg.235	Protein	44160	44002	-	FIG00820183: hypothetical protein
contig_11	fig|6666666.28487.peg.236	Protein	44831	44199	-	Long-chain fatty-acid-CoA ligase (EC 6.2.1.3), Mycobacterial subgroup FadD16
contig_11	fig|6666666.28487.peg.237	Protein	45070	45549	+	Bacterioferritin (BFR)
contig_11	fig|6666666.28487.peg.238	Protein	45664	47730	+	Multidrug resistance protein B
contig_11	fig|6666666.28487.peg.239	Protein	47752	48234	+	FIG00824327: hypothetical protein
contig_11	fig|6666666.28487.peg.240	Protein	48256	49617	+	Glutamine synthetase (EC 6.3.1.2), putative
contig_11	fig|6666666.28487.peg.241	Protein	49654	50757	+	Amidohydrolase
contig_11	fig|6666666.28487.peg.242	Protein	52600	50831	-	D-aminoacylase (EC 3.5.1.81)
contig_11	fig|6666666.28487.peg.243	Protein	53081	52611	-	FIG00826330: hypothetical protein
contig_11	fig|6666666.28487.peg.244	Protein	54873	53071	-	FIG00828677: hypothetical protein
contig_11	fig|6666666.28487.peg.245	Protein	54983	55912	+	Dienelactone hydrolase family
contig_11	fig|6666666.28487.peg.246	Protein	56016	57122	+	Dolichol-phosphate mannosyltransferase (EC 2.4.1.83) in lipid-linked oligosaccharide synthesis cluster
contig_11	fig|6666666.28487.peg.247	Protein	57173	57583	+	FIG00833118: hypothetical protein
contig_11	fig|6666666.28487.peg.248	Protein	57673	58140	+	FIG00823503: hypothetical protein
contig_11	fig|6666666.28487.peg.249	Protein	59432	58137	-	putative cytochrome P450 hydroxylase
contig_11	fig|6666666.28487.peg.250	Protein	59822	59451	-	FIG00823221: hypothetical protein
contig_11	fig|6666666.28487.peg.251	Protein	60398	59922	-	FIG00820653: hypothetical protein

contig_11	fig|6666666.28487.peg.252	Protein	60644	60432	-	hypothetical protein
contig_11	fig|6666666.28487.peg.253	Protein	60896	61777	+	Cyclopropane-fatty-acyl-phospholipid synthase (EC 2.1.1.79)
contig_11	fig|6666666.28487.peg.254	Protein	62783	61761	-	Agmatinase (EC 3.5.3.11)
contig_11	fig|6666666.28487.peg.255	Protein	63278	62844	-	4-hydroxybenzoyl-CoA thioesterase family active site
contig_11	fig|6666666.28487.peg.256	Protein	63348	64295	+	Threonine dehydratase, catabolic (EC 4.3.1.19)
contig_11	fig|6666666.28487.peg.257	Protein	64567	64331	-	hypothetical protein
contig_112	fig|6666666.28487.peg.258	Protein	162	779	+	Mobile element protein
contig_113	fig|6666666.28487.peg.259	Protein	350	463	+	hypothetical protein
contig_12	fig|6666666.28487.peg.260	Protein	11	190	+	phage integrase family protein
contig_12	fig|6666666.28487.peg.261	Protein	284	580	+	phage-related integrase/recombinase
contig_12	fig|6666666.28487.peg.262	Protein	577	867	+	hypothetical protein
contig_12	fig|6666666.28487.peg.263	Protein	1531	1394	-	hypothetical protein
contig_12	fig|6666666.28487.peg.264	Protein	2068	1667	-	hypothetical protein
contig_12	fig|6666666.28487.peg.265	Protein	4100	2751	-	MmgE/PrpD family protein
contig_12	fig|6666666.28487.peg.266	Protein	4671	5189	+	MaoC-like dehydratase
contig_12	fig|6666666.28487.peg.267	Protein	5191	5814	+	L-carnitine dehydratase/bile acid-inducible protein F
contig_12	fig|6666666.28487.peg.268	Protein	5865	6020	+	hypothetical protein
contig_12	fig|6666666.28487.peg.269	Protein	6022	7185	+	Butyryl-CoA dehydrogenase (EC 1.3.99.2)
contig_12	fig|6666666.28487.peg.270	Protein	8907	7639	-	Ethanolamine permease

contig_12	fig|6666666.28487.peg.271	Protein	9171	10100	+	hypothetical protein
contig_12	fig|6666666.28487.peg.272	Protein	10310	11734	+	Glycerol-3-phosphate dehydrogenase (EC 1.1.5.3)
contig_12	fig|6666666.28487.peg.273	Protein	11727	12512	+	oxidoreductase
contig_12	fig|6666666.28487.peg.274	Protein	12648	12770	+	hypothetical protein
contig_12	fig|6666666.28487.peg.275	Protein	12824	13621	+	Tagatose 1,6-bisphosphate aldolase (EC 4.1.2.40)
contig_12	fig|6666666.28487.peg.276	Protein	13618	14409	+	hypothetical protein
contig_12	fig|6666666.28487.peg.277	Protein	14406	15377	+	hypothetical protein
contig_12	fig|6666666.28487.peg.278	Protein	15374	16708	+	hypothetical protein
contig_12	fig|6666666.28487.peg.279	Protein	17045	17371	+	Probable F420-dependent oxidoreductase family protein
contig_12	fig|6666666.28487.peg.280	Protein	17400	17552	+	hypothetical protein
contig_12	fig|6666666.28487.peg.281	Protein	17561	17689	+	hypothetical protein
contig_12	fig|6666666.28487.peg.282	Protein	17936	17757	-	hypothetical protein
contig_12	fig|6666666.28487.peg.283	Protein	18033	18158	+	hypothetical protein
contig_12	fig|6666666.28487.peg.284	Protein	21999	18400	-	hypothetical protein
contig_12	fig|6666666.28487.peg.285	Protein	22300	23343	+	FIG00823379: hypothetical protein
contig_12	fig|6666666.28487.peg.286	Protein	23679	23446	-	hypothetical protein
contig_12	fig|6666666.28487.peg.287	Protein	25003	23951	-	transposase
contig_12	fig|6666666.28487.peg.288	Protein	25316	25702	+	FIG00824223: hypothetical protein
contig_12	fig|6666666.28487.peg.289	Protein	25740	26492	+	FIG027960: hypothetical protein

contig_12	fig|6666666.28487.peg.290	Protein	29155	28040	-	hypothetical protein
contig_12	fig|6666666.28487.peg.291	Protein	29261	29485	+	hypothetical protein
contig_12	fig|6666666.28487.peg.292	Protein	29943	29542	-	hypothetical protein
contig_12	fig|6666666.28487.peg.293	Protein	30587	29940	-	hypothetical protein
contig_12	fig|6666666.28487.peg.294	Protein	31203	30694	-	hypothetical protein
contig_12	fig|6666666.28487.peg.295	Protein	31309	31464	+	hypothetical protein
contig_12	fig|6666666.28487.peg.296	Protein	32185	31733	-	hypothetical protein
contig_12	fig|6666666.28487.peg.297	Protein	33599	32628	-	hypothetical protein
contig_12	fig|6666666.28487.peg.298	Protein	35992	33614	-	hypothetical protein
contig_12	fig|6666666.28487.peg.299	Protein	36852	35989	-	AAA ATPase, central region
contig_12	fig|6666666.28487.peg.300	Protein	37270	38286	+	hypothetical protein
contig_12	fig|6666666.28487.peg.301	Protein	38307	38804	+	hypothetical protein
contig_12	fig|6666666.28487.peg.302	Protein	38987	39226	+	hypothetical protein
contig_12	fig|6666666.28487.peg.303	Protein	39475	40428	+	Tetratricopeptide TPR_4
contig_12	fig|6666666.28487.peg.304	Protein	40492	41388	+	hypothetical protein
contig_12	fig|6666666.28487.peg.305	Protein	41635	42654	+	hypothetical protein
contig_12	fig|6666666.28487.peg.306	Protein	43893	44735	+	hypothetical protein
contig_12	fig|6666666.28487.peg.307	Protein	44748	44894	+	hypothetical protein
contig_12	fig|6666666.28487.peg.308	Protein	45055	45186	+	hypothetical protein

contig_12	fig|6666666.28487.peg.309	Protein	45276	45443	+	hypothetical protein
contig_12	fig|6666666.28487.peg.310	Protein	45888	46364	+	hypothetical protein
contig_12	fig|6666666.28487.peg.311	Protein	46442	46843	+	hypothetical protein
contig_12	fig|6666666.28487.peg.312	Protein	47098	47319	+	hypothetical protein
contig_12	fig|6666666.28487.peg.313	Protein	48356	47565	-	Mobile element protein
contig_12	fig|6666666.28487.peg.314	Protein	49968	48376	-	Mobile element protein
contig_12	fig|6666666.28487.peg.315	Protein	50437	49970	-	Transcriptional regulator, MarR family
contig_12	fig|6666666.28487.peg.316	Protein	50508	51680	+	antibiotic resistance macrolide glycosyltransferase
contig_12	fig|6666666.28487.peg.317	Protein	52778	51957	-	Mobile element protein
contig_12	fig|6666666.28487.peg.318	Protein	53143	52808	-	Mobile element protein
contig_12	fig|6666666.28487.peg.319	Protein	53704	53561	-	hypothetical protein
contig_12	fig|6666666.28487.peg.320	Protein	53952	53809	-	hypothetical protein
contig_12	fig|6666666.28487.peg.321	Protein	54853	54212	-	hypothetical protein
contig_12	fig|6666666.28487.peg.322	Protein	54917	55864	+	Transcriptional regulator, DeoR family
contig_12	fig|6666666.28487.peg.323	Protein	55982	56140	+	hypothetical protein
contig_12	fig|6666666.28487.peg.324	Protein	56262	57257	+	hypothetical protein
contig_12	fig|6666666.28487.peg.325	Protein	58593	57385	-	Lycopene beta cyclase (EC 1.14.-.-)
contig_12	fig|6666666.28487.peg.326	Protein	59423	58827	-	Transcriptional regulator, TetR family
contig_12	fig|6666666.28487.peg.327	Protein	59531	60019	+	COGs COG3558

contig_12	fig|6666666.28487.peg.328	Protein	61353	60055	-	hypothetical protein
contig_12	fig|6666666.28487.peg.329	Protein	61486	61644	+	hypothetical protein
contig_12	fig|6666666.28487.peg.330	Protein	61846	63531	+	Choline dehydrogenase (EC 1.1.99.1)
contig_12	fig|6666666.28487.peg.331	Protein	64285	63590	-	Short-chain dehydrogenase/reductase SDR
contig_12	fig|6666666.28487.peg.332	Protein	64480	64331	-	hypothetical protein
contig_12	fig|6666666.28487.peg.333	Protein	64637	65446	+	POSSIBLE OXIDOREDUCTASE (EC 1.-.-.-)
contig_12	fig|6666666.28487.peg.334	Protein	65929	65465	-	FIG00691228: hypothetical protein
contig_12	fig|6666666.28487.peg.335	Protein	66007	66579	+	Dihydrofolate reductase (EC 1.5.1.3)
contig_12	fig|6666666.28487.peg.336	Protein	67026	66634	-	Lipoprotein LpqJ
contig_12	fig|6666666.28487.peg.337	Protein	67404	67787	+	peptidylprolyl isomerase, FKBP-type
contig_12	fig|6666666.28487.peg.338	Protein	68179	69045	+	Integral membrane protein
contig_12	fig|6666666.28487.peg.339	Protein	69302	69048	-	hypothetical protein
contig_12	fig|6666666.28487.peg.340	Protein	69421	69582	+	hypothetical protein
contig_12	fig|6666666.28487.peg.341	Protein	69888	69595	-	hypothetical protein
contig_12	fig|6666666.28487.peg.342	Protein	70040	71155	+	Esterase/lipase/thioesterase
contig_12	fig|6666666.28487.peg.343	Protein	71167	71532	+	Ribosome-associated heat shock protein implicated in the recycling of the 50S subunit (S4 paralog)
contig_12	fig|6666666.28487.peg.344	Protein	71543	72316	+	FIG00821135: hypothetical protein
contig_12	fig|6666666.28487.peg.345	Protein	73659	72322	-	NADH dehydrogenase (EC 1.6.99.3)
contig_12	fig|6666666.28487.peg.346	Protein	73822	74781	+	Antigen 85-C precursor (85C) (Antigen 85 complex C)

						(Ag85C) (Mycolyl transferase 85C) (EC 2.3.1.-)
contig_12	fig|6666666.28487.peg.347	Protein	75102	74836	-	FIG00826893: hypothetical protein
contig_12	fig|6666666.28487.peg.348	Protein	75243	75581	+	hypothetical protein
contig_12	fig|6666666.28487.peg.349	Protein	75796	76140	+	hypothetical protein
contig_12	fig|6666666.28487.peg.350	Protein	76527	76120	-	hypothetical protein
contig_12	fig|6666666.28487.peg.351	Protein	77090	76578	-	FIG00820297: hypothetical protein
contig_12	fig|6666666.28487.peg.352	Protein	77152	78693	+	Long-chain-fatty-acid--CoA ligase (EC 6.2.1.3)
contig_12	fig|6666666.28487.peg.353	Protein	78835	78665	-	hypothetical protein
contig_12	fig|6666666.28487.peg.354	Protein	78819	79571	+	OsmC-like family protein
contig_12	fig|6666666.28487.peg.355	Protein	80913	79606	-	hypothetical protein
contig_12	fig|6666666.28487.peg.356	Protein	81196	81630	+	MOSC domain protein
contig_12	fig|6666666.28487.peg.357	Protein	81998	81633	-	hypothetical protein
contig_13	fig|6666666.28487.peg.358	Protein	46	747	+	Enoyl-CoA hydratase
contig_13	fig|6666666.28487.peg.359	Protein	850	1842	+	Magnesium and cobalt transport protein CorA
contig_13	fig|6666666.28487.peg.360	Protein	3028	1820	-	Anthranilate dioxygenase reductase
contig_13	fig|6666666.28487.peg.361	Protein	3057	3803	+	putative conserved membrane protein
contig_13	fig|6666666.28487.peg.362	Protein	3905	7192	+	Exodeoxyribonuclease V gamma chain (EC 3.1.11.5)
contig_13	fig|6666666.28487.peg.363	Protein	7261	10509	+	Exodeoxyribonuclease V beta chain (EC 3.1.11.5)
contig_13	fig|6666666.28487.peg.364	Protein	10506	12230	+	Exodeoxyribonuclease V alpha chain (EC 3.1.11.5)

contig_13	fig|6666666.28487.peg.365	Protein	12403	12687	+	hypothetical protein
contig_13	fig|6666666.28487.peg.366	Protein	12838	13146	+	hypothetical protein
contig_13	fig|6666666.28487.peg.367	Protein	13199	13336	+	hypothetical protein
contig_13	fig|6666666.28487.peg.368	Protein	13282	15660	+	Possible membrane protein
contig_13	fig|6666666.28487.peg.369	Protein	15800	17023	+	Alkanesulfonate monooxygenase (EC 1.14.14.5)
contig_13	fig|6666666.28487.peg.370	Protein	18255	17029	-	putative cytochrome P450 hydroxylase
contig_13	fig|6666666.28487.peg.371	Protein	18455	19645	+	MCE-family protein Mce1A
contig_13	fig|6666666.28487.peg.372	Protein	19642	20682	+	MCE-family protein Mce1B
contig_13	fig|6666666.28487.peg.373	Protein	20679	22118	+	MCE-family protein Mce1C
contig_13	fig|6666666.28487.peg.374	Protein	22120	23415	+	MCE-family protein Mce1D
contig_13	fig|6666666.28487.peg.375	Protein	23446	23649	+	MCE-family protein Mce1D
contig_13	fig|6666666.28487.peg.376	Protein	23646	24770	+	MCE-family lipoprotein LprK (MCE-family lipoprotein Mce1e)
contig_13	fig|6666666.28487.peg.377	Protein	24773	26320	+	MCE-family protein Mce1F
contig_13	fig|6666666.28487.peg.378	Protein	26394	26960	+	Transcriptional regulator, TetR family
contig_13	fig|6666666.28487.peg.379	Protein	27199	26978	-	FIG00831874: hypothetical protein
contig_13	fig|6666666.28487.peg.380	Protein	27360	28085	+	FIG00826787: hypothetical protein
contig_13	fig|6666666.28487.peg.381	Protein	28184	28050	-	hypothetical protein
contig_13	fig|6666666.28487.peg.382	Protein	28209	30431	+	Transmembrane transport protein MmpL13b
contig_13	fig|6666666.28487.peg.383	Protein	30443	31519	+	Chalcone synthase (EC 2.3.1.74)

contig_13	fig|6666666.28487.peg.384	Protein	31531	32046	+	Isoprenylcysteine carboxyl methyltransferase
contig_13	fig|6666666.28487.peg.385	Protein	34981	33008	-	FIG00824246: hypothetical protein
contig_13	fig|6666666.28487.peg.386	Protein	35060	37585	+	Glycogen phosphorylase (EC 2.4.1.1)
contig_13	fig|6666666.28487.peg.387	Protein	38102	37659	-	Transcriptional regulator, MarR family
contig_13	fig|6666666.28487.peg.388	Protein	38169	39383	+	Drug resistance transporter Bcr/CflA subfamily
contig_13	fig|6666666.28487.peg.389	Protein	39796	39380	-	hypothetical protein
contig_13	fig|6666666.28487.peg.390	Protein	39843	40592	+	hypothetical protein
contig_13	fig|6666666.28487.peg.391	Protein	40691	41737	+	SECRETED ANTIGEN 85-B FBPB (85B) (ANTIGEN 85 COMPLEX B) (MYCOLYL TRANSFERASE 85B) (FIBRONECTIN-BINDING PROTEIN B) (EXTRACELLULAR ALPHA-ANTIGEN)
contig_13	fig|6666666.28487.peg.392	Protein	42619	41738	-	hypothetical protein
contig_13	fig|6666666.28487.peg.393	Protein	43098	42682	-	hypothetical protein
contig_13	fig|6666666.28487.peg.394	Protein	43567	43136	-	hypothetical protein
contig_13	fig|6666666.28487.peg.395	Protein	43692	44705	+	AMIDASE
contig_13	fig|6666666.28487.peg.396	Protein	44732	45550	+	Short-chain dehydrogenase/reductase SDR
contig_13	fig|6666666.28487.peg.397	Protein	46151	45552	-	Transcriptional regulator, TetR family
contig_13	fig|6666666.28487.peg.398	Protein	46224	47204	+	N-carbamoylputrescine amidase (3.5.1.53)
contig_13	fig|6666666.28487.peg.399	Protein	47201	48805	+	Exoenzymes regulatory protein AepA in lipid-linked oligosaccharide synthesis cluster
contig_13	fig|6666666.28487.peg.400	Protein	48815	50164	+	Putrescine importer

contig_13	fig|6666666.28487.peg.401	Protein	51280	50246	-	2-dehydropantoate 2-reductase (EC 1.1.1.169)
contig_13	fig|6666666.28487.peg.402	Protein	54431	51333	-	FIG00825314: hypothetical protein
contig_13	fig|6666666.28487.peg.403	Protein	55036	54431	-	FIG00826528: hypothetical protein
contig_13	fig|6666666.28487.peg.404	Protein	55082	55765	+	FIG00825989: hypothetical protein
contig_13	fig|6666666.28487.peg.405	Protein	55870	58212	+	putative helicase
contig_13	fig|6666666.28487.peg.406	Protein	58278	59297	+	Low-specificity L-threonine aldolase (EC 4.1.2.5)
contig_13	fig|6666666.28487.peg.407	Protein	59658	61022	+	Amine oxidase [flavin-containing] A (EC 1.4.3.4)
contig_13	fig|6666666.28487.peg.408	Protein	61063	61197	+	hypothetical protein
contig_13	fig|6666666.28487.peg.409	Protein	61253	62395	+	Ferredoxin reductase
contig_13	fig|6666666.28487.peg.410	Protein	62413	63276	+	hypothetical protein
contig_13	fig|6666666.28487.peg.411	Protein	63336	64250	+	hypothetical protein
contig_13	fig|6666666.28487.peg.412	Protein	64407	64778	+	hypothetical protein
contig_13	fig|6666666.28487.peg.413	Protein	65550	64804	-	FIG00825022: hypothetical protein
contig_13	fig|6666666.28487.peg.414	Protein	65584	66540	+	Enoyl-[acyl-carrier-protein] reductase [FMN] (EC 1.3.1.9)
contig_13	fig|6666666.28487.peg.415	Protein	66732	68174	+	CBSS-498211.3.peg.1514: hypothetical protein
contig_13	fig|6666666.28487.peg.416	Protein	68171	68875	+	FIG039767: hypothetical protein
contig_13	fig|6666666.28487.peg.417	Protein	68868	72230	+	FIG007317: hypothetical protein
contig_13	fig|6666666.28487.peg.418	Protein	72253	73272	+	FIG00828378: hypothetical protein
contig_13	fig|6666666.28487.peg.419	Protein	73269	74852	+	MFS permease

contig_13	fig|6666666.28487.peg.420	Protein	74993	75532	+	RNA polymerase sigma-54 factor RpoN
contig_13	fig|6666666.28487.peg.421	Protein	75523	75963	+	hypothetical protein
contig_13	fig|6666666.28487.peg.422	Protein	76066	76236	+	hypothetical protein
contig_13	fig|6666666.28487.peg.423	Protein	76401	77411	+	Integral membrane protein TerC
contig_13	fig|6666666.28487.peg.424	Protein	78631	77405	-	Na+/H+ antiporter NhaA type
contig_13	fig|6666666.28487.peg.425	Protein	78632	79003	+	hypothetical protein
contig_13	fig|6666666.28487.peg.426	Protein	79000	79560	+	Transcriptional regulator, TetR family
contig_13	fig|6666666.28487.peg.427	Protein	80366	79539	-	Putative DNA-binding protein
contig_13	fig|6666666.28487.peg.428	Protein	80487	82289	+	Dihydroxy-acid dehydratase (EC 4.2.1.9)
contig_13	fig|6666666.28487.peg.429	Protein	84017	82311	-	Flavohemoprotein (Hemoglobin-like protein) (Flavohemoglobin) (Nitric oxide dioxygenase) (EC 1.14.12.17)
contig_13	fig|6666666.28487.peg.430	Protein	84096	84758	+	haloacid dehalogenase, type II
contig_13	fig|6666666.28487.peg.431	Protein	85030	85635	+	Transcriptional regulator, TetR family
contig_13	fig|6666666.28487.peg.432	Protein	85735	86286	+	Transcriptional regulator, TetR family
contig_13	fig|6666666.28487.peg.433	Protein	86410	86742	+	hypothetical protein
contig_13	fig|6666666.28487.peg.434	Protein	87559	86786	-	hypothetical protein
contig_13	fig|6666666.28487.peg.435	Protein	87758	87570	-	hypothetical protein
contig_13	fig|6666666.28487.peg.436	Protein	87869	89104	+	L-lactate dehydrogenase (EC 1.1.2.3)
contig_13	fig|6666666.28487.peg.437	Protein	91346	89193	-	diguanylate cyclase/phosphodiesterase (GGDEF & EAL domains) with PAS/PAC sensor(s)

contig_13	fig|6666666.28487.peg.438	Protein	91976	91554	-	Organic hydroperoxide resistance protein
contig_13	fig|6666666.28487.peg.439	Protein	92418	91987	-	Organic hydroperoxide resistance transcriptional regulator
contig_13	fig|6666666.28487.peg.440	Protein	92524	93501	+	FIG00830619: hypothetical protein
contig_14	fig|6666666.28487.peg.441	Protein	221	66	-	hypothetical protein
contig_14	fig|6666666.28487.peg.442	Protein	490	281	-	hypothetical protein
contig_14	fig|6666666.28487.peg.443	Protein	765	487	-	hypothetical protein
contig_14	fig|6666666.28487.peg.444	Protein	2054	969	-	Aminopeptidase YpdF (MP-, MA-, MS-, AP-, NP- specific)
contig_14	fig|6666666.28487.peg.445	Protein	2088	2696	+	PROBABLE CONSERVED TRANSMEMBRANE PROTEIN
contig_14	fig|6666666.28487.peg.446	Protein	3810	2725	-	3-dehydroquinate synthase (EC 4.2.3.4)
contig_14	fig|6666666.28487.peg.447	Protein	4469	3807	-	Shikimate kinase I (EC 2.7.1.71)
contig_14	fig|6666666.28487.peg.448	Protein	5692	4469	-	Chorismate synthase (EC 4.2.3.5)
contig_14	fig|6666666.28487.peg.449	Protein	6466	5723	-	hypothetical protein
contig_14	fig|6666666.28487.peg.450	Protein	6934	6503	-	peptidase A24A, prepilin type IV
contig_14	fig|6666666.28487.peg.451	Protein	7744	6980	-	Shikimate 5-dehydrogenase I alpha (EC 1.1.1.25)
contig_14	fig|6666666.28487.peg.452	Protein	9018	7771	-	FIG004453: protein YceG like
contig_14	fig|6666666.28487.peg.453	Protein	9538	9011	-	Putative Holliday junction resolvase YggF
contig_14	fig|6666666.28487.peg.454	Protein	12245	9543	-	Alanyl-tRNA synthetase (EC 6.1.1.7)
contig_14	fig|6666666.28487.peg.455	Protein	12710	12309	-	FIG027887: hypothetical protein
contig_14	fig|6666666.28487.peg.456	Protein	12849	13637	+	Potassium voltage-gated channel subfamily KQT; possible potassium channel, VIC family

contig_14	fig|6666666.28487.peg.457	Protein	13705	14010	+	Possible membrane protein
contig_14	fig|6666666.28487.peg.458	Protein	14257	14559	+	hypothetical protein
contig_14	fig|6666666.28487.peg.459	Protein	15049	14576	-	Probable glutathione S-transferase-related transmembrane protein (EC 2.5.1.18)
contig_14	fig|6666666.28487.peg.460	Protein	15399	15046	-	Transcriptional regulator, ArsR family
contig_14	fig|6666666.28487.peg.461	Protein	16826	15483	-	ATPase, AAA family
contig_14	fig|6666666.28487.peg.462	Protein	17675	16836	-	FIG00826016: hypothetical protein
contig_14	fig|6666666.28487.peg.463	Protein	17725	18300	+	hypothetical protein
contig_14	fig|6666666.28487.peg.464	Protein	18341	19126	+	Short chain oxidoreductase
contig_14	fig|6666666.28487.peg.465	Protein	19123	19602	+	hypothetical protein
contig_14	fig|6666666.28487.peg.466	Protein	19599	20375	+	3-oxoacyl-[acyl-carrier protein] reductase (EC 1.1.1.100)
contig_14	fig|6666666.28487.peg.467	Protein	20434	23076	+	Phosphoenolpyruvate synthase (EC 2.7.9.2)
contig_14	fig|6666666.28487.peg.468	Protein	25016	24084	-	Protein containing transglutaminase-like domain, putative cysteine protease
contig_14	fig|6666666.28487.peg.469	Protein	27712	25013	-	Protein containing domains DUF404, DUF407, DUF403
contig_14	fig|6666666.28487.peg.470	Protein	31047	27712	-	Large protein containing transglutaminase-like domain
contig_14	fig|6666666.28487.peg.471	Protein	32080	31091	-	putative integral membrane protein
contig_14	fig|6666666.28487.peg.472	Protein	32160	32975	+	Glycerol-3-phosphate regulon repressor, DeoR family
contig_15	fig|6666666.28487.peg.473	Protein	30	713	+	Proline-rich 28 kDa antigen
contig_15	fig|6666666.28487.peg.474	Protein	787	1149	+	hypothetical protein

contig_15	fig|6666666.28487.peg.475	Protein	1760	1146	-	FIG00996838: hypothetical protein
contig_15	fig|6666666.28487.peg.476	Protein	1887	1768	-	hypothetical protein
contig_15	fig|6666666.28487.peg.477	Protein	1912	3195	+	Possible membrane protein
contig_15	fig|6666666.28487.peg.478	Protein	3224	3991	+	FIG00822256: hypothetical protein
contig_15	fig|6666666.28487.peg.479	Protein	4570	4076	-	FIG00820046: hypothetical protein
contig_15	fig|6666666.28487.peg.480	Protein	6022	4577	-	tRNA nucleotidyltransferase (EC 2.7.7.21) (EC 2.7.7.25)
contig_15	fig|6666666.28487.peg.481	Protein	6399	6917	+	MutT/nudix family protein
contig_15	fig|6666666.28487.peg.482	Protein	6938	9289	+	hypothetical protein
contig_15	fig|6666666.28487.peg.483	Protein	9324	12875	+	Proposed peptidoglycan lipid II flippase MurJ
contig_15	fig|6666666.28487.peg.484	Protein	12932	13510	+	RNA polymerase sigma-70 factor, ECF subfamily
contig_15	fig|6666666.28487.peg.485	Protein	13569	14135	+	hypothetical protein
contig_15	fig|6666666.28487.peg.486	Protein	14248	15243	+	Thioredoxin reductase (EC 1.8.1.9)
contig_15	fig|6666666.28487.peg.487	Protein	15240	15569	+	Thioredoxin reductase (EC 1.8.1.9)
contig_15	fig|6666666.28487.peg.488	Protein	15645	16841	+	N-acetylmuramoyl-L-alanine amidase (EC 3.5.1.28)
contig_15	fig|6666666.28487.peg.489	Protein	17595	16846	-	FIG007808: hypothetical protein
contig_15	fig|6666666.28487.peg.490	Protein	18808	17813	-	Chromosome (plasmid) partitioning protein ParB / Stage 0 sporulation protein J
contig_15	fig|6666666.28487.peg.491	Protein	19784	18852	-	Chromosome (plasmid) partitioning protein ParA / Sporulation initiation inhibitor protein Soj
contig_15	fig|6666666.28487.peg.492	Protein	20467	19784	-	rRNA small subunit 7-methylguanosine (m7G) methyltransferase GidB

contig_15	fig|6666666.28487.peg.493	Protein	21126	20548	-	RNA-binding protein Jag
contig_15	fig|6666666.28487.peg.494	Protein	22294	21161	-	Inner membrane protein translocase component YidC, long form
contig_15	fig|6666666.28487.peg.495	Protein	22870	22610	-	Ribonuclease P protein component (EC 3.1.26.5)
contig_15	fig|6666666.28487.peg.496	Protein	22884	23003	+	hypothetical protein
contig_15	fig|6666666.28487.peg.497	Protein	23132	22989	-	LSU ribosomal protein L34p
contig_15	fig|6666666.28487.peg.498	Protein	23622	23491	-	hypothetical protein
contig_15	fig|6666666.28487.peg.499	Protein	23725	25206	+	Chromosomal replication initiator protein DnaA
contig_15	fig|6666666.28487.peg.500	Protein	25738	26934	+	DNA polymerase III beta subunit (EC 2.7.7.7)
contig_15	fig|6666666.28487.peg.501	Protein	26968	27861	+	6-phosphogluconate dehydrogenase, decarboxylating (EC 1.1.1.44)
contig_15	fig|6666666.28487.peg.502	Protein	27869	29029	+	DNA recombination and repair protein RecF
contig_15	fig|6666666.28487.peg.503	Protein	29026	29583	+	Zn-ribbon-containing, possibly RNA-binding protein and truncated derivatives
contig_15	fig|6666666.28487.peg.504	Protein	29795	31816	+	DNA gyrase subunit B (EC 5.99.1.3)
contig_15	fig|6666666.28487.peg.505	Protein	31836	35639	+	DNA gyrase subunit A (EC 5.99.1.3)
contig_15	fig|6666666.28487.peg.506	Protein	35871	36584	+	FIG187021: hypothetical protein
contig_15	fig|6666666.28487.rna.2	RNA	36655	36728	+	tRNA-Ile-GAT
contig_15	fig|6666666.28487.peg.507	Protein	36746	36871	+	hypothetical protein
contig_15	fig|6666666.28487.rna.3	RNA	36862	36934	+	tRNA-Ala-TGC
contig_15	fig|6666666.28487.peg.508	Protein	37029	37202	+	hypothetical protein

contig_15	fig|6666666.28487.peg.509	Protein	38552	37206	-	L-serine dehydratase (EC 4.3.1.17)
contig_15	fig|6666666.28487.peg.510	Protein	39001	38567	-	FIG024317: hypothetical protein
contig_15	fig|6666666.28487.peg.511	Protein	39120	39647	+	Peptidyl-prolyl cis-trans isomerase (EC 5.2.1.8)
contig_15	fig|6666666.28487.peg.512	Protein	40132	39701	-	FIG020554: membrane protein
contig_15	fig|6666666.28487.peg.513	Protein	40560	40279	-	FIG018426: putative septation inhibitor protein
contig_15	fig|6666666.28487.peg.514	Protein	40693	41454	+	FIG215594: Membrane spanning protein
contig_15	fig|6666666.28487.peg.515	Protein	41695	42831	+	PE-PPE, C-terminal domain protein
contig_15	fig|6666666.28487.peg.516	Protein	43108	44259	+	Uncharacterized protein ImpB
contig_15	fig|6666666.28487.peg.517	Protein	44298	44972	+	Anthranilate synthase, amidotransferase component (EC 4.1.3.27) @ Para-aminobenzoate synthase, amidotransferase component (EC 2.6.1.85)
contig_15	fig|6666666.28487.peg.518	Protein	46827	44950	-	Serine/threonine-protein kinase PknB (EC 2.7.11.1)
contig_15	fig|6666666.28487.peg.519	Protein	48125	46824	-	Serine/threonine-protein kinase PknA (EC 2.7.11.1)
contig_15	fig|6666666.28487.peg.520	Protein	49664	48189	-	Cell division protein FtsI [Peptidoglycan synthetase] (EC 2.4.1.129)
contig_15	fig|6666666.28487.peg.521	Protein	51073	49661	-	Cell division protein FtsW
contig_15	fig|6666666.28487.peg.522	Protein	52638	51070	-	Serine/threonine phosphatase PPP (EC 3.1.3.16)
contig_15	fig|6666666.28487.peg.523	Protein	53128	52661	-	FIG00818182: hypothetical protein
contig_15	fig|6666666.28487.peg.524	Protein	54559	53192	-	FIG025441: hypothetical protein
contig_15	fig|6666666.28487.rna.4	RNA	54811	54893	+	tRNA-Leu-CAG
contig_15	fig|6666666.28487.peg.525	Protein	55158	56612	+	Type I restriction-modification system, DNA-methyltransferase

						subunit M (EC 2.1.1.72)
contig_15	fig|6666666.28487.peg.526	Protein	57773	61186	+	Type I restriction-modification system, restriction subunit R (EC 3.1.21.3)
contig_15	fig|6666666.28487.peg.527	Protein	61209	61877	+	hypothetical protein
contig_15	fig|6666666.28487.peg.528	Protein	62516	62082	-	hypothetical protein
contig_15	fig|6666666.28487.peg.529	Protein	63720	62536	-	COG0476: Dinucleotide-utilizing enzymes involved in molybdopterin and thiamine biosynthesis family 2
contig_15	fig|6666666.28487.peg.530	Protein	64348	63695	-	hypothetical protein
contig_15	fig|6666666.28487.peg.531	Protein	64478	64834	+	hypothetical protein
contig_15	fig|6666666.28487.peg.532	Protein	64834	65982	+	DNA-binding protein
contig_15	fig|6666666.28487.peg.533	Protein	66920	66081	-	hypothetical protein
contig_15	fig|6666666.28487.peg.534	Protein	67549	66917	-	hypothetical protein
contig_15	fig|6666666.28487.peg.535	Protein	68066	67557	-	FIG001353: Acetyltransferase
contig_15	fig|6666666.28487.peg.536	Protein	68341	68063	-	FIG032766: hypothetical protein
contig_15	fig|6666666.28487.peg.537	Protein	68506	68718	+	hypothetical protein
contig_15	fig|6666666.28487.peg.538	Protein	69622	68699	-	hypothetical protein
contig_15	fig|6666666.28487.peg.539	Protein	69807	69619	-	hypothetical protein
contig_15	fig|6666666.28487.peg.540	Protein	70449	69973	-	hypothetical protein
contig_15	fig|6666666.28487.peg.541	Protein	71502	70498	-	hypothetical protein
contig_15	fig|6666666.28487.peg.542	Protein	71922	72314	+	FIG00833009: hypothetical protein

contig_15	fig|6666666.28487.peg.543	Protein	72684	73811	+	Appr-1-p processing domain protein
contig_15	fig|6666666.28487.peg.544	Protein	74601	74837	+	hypothetical protein
contig_15	fig|6666666.28487.peg.545	Protein	74889	75131	+	hypothetical protein
contig_15	fig|6666666.28487.peg.546	Protein	75287	75946	+	FIG00823833: hypothetical protein
contig_15	fig|6666666.28487.peg.547	Protein	76348	76533	+	hypothetical protein
contig_15	fig|6666666.28487.peg.548	Protein	76807	77862	+	hypothetical protein
contig_15	fig|6666666.28487.peg.549	Protein	77866	79644	+	hypothetical protein
contig_15	fig|6666666.28487.peg.550	Protein	80364	82100	+	Type I restriction-modification system, DNA-methyltransferase subunit M (EC 2.1.1.72)
contig_15	fig|6666666.28487.peg.551	Protein	82097	83305	+	Type I restriction-modification system, specificity subunit S (EC 3.1.21.3)
contig_15	fig|6666666.28487.peg.552	Protein	83308	86430	+	Type I restriction-modification system, restriction subunit R (EC 3.1.21.3)
contig_15	fig|6666666.28487.peg.553	Protein	86737	88152	+	hypothetical protein
contig_15	fig|6666666.28487.peg.554	Protein	88251	89819	+	hypothetical protein
contig_15	fig|6666666.28487.peg.555	Protein	91998	91024	-	putative transcriptional regulator
contig_15	fig|6666666.28487.peg.556	Protein	92606	91995	-	hypothetical protein
contig_15	fig|6666666.28487.peg.557	Protein	93266	93439	+	hypothetical protein
contig_15	fig|6666666.28487.peg.558	Protein	94317	95399	+	hypothetical protein
contig_15	fig|6666666.28487.peg.559	Protein	95396	97189	+	hypothetical protein
contig_15	fig|6666666.28487.peg.560	Protein	98210	97614	-	hypothetical protein

contig_15	fig|6666666.28487.peg.561	Protein	98468	98325	-	hypothetical protein
contig_15	fig|6666666.28487.peg.562	Protein	98773	98465	-	hypothetical protein
contig_15	fig|6666666.28487.peg.563	Protein	100218	98797	-	Streptococcal hemagglutinin protein
contig_15	fig|6666666.28487.peg.564	Protein	100385	102247	+	FIG00842086: hypothetical protein
contig_15	fig|6666666.28487.peg.565	Protein	102300	103883	+	Type I restriction-modification system, DNA-methyltransferase subunit M (EC 2.1.1.72)
contig_15	fig|6666666.28487.peg.566	Protein	104353	105057	+	Type I restriction-modification system, specificity subunit S (EC 3.1.21.3)
contig_15	fig|6666666.28487.peg.567	Protein	105058	106161	+	5'-nucleotidase (EC 3.1.3.5)
contig_15	fig|6666666.28487.peg.568	Protein	106158	109403	+	Type I restriction-modification system, restriction subunit R (EC 3.1.21.3)
contig_15	fig|6666666.28487.peg.569	Protein	111047	109539	-	hypothetical protein
contig_15	fig|6666666.28487.peg.570	Protein	111738	111472	-	hypothetical protein
contig_15	fig|6666666.28487.peg.571	Protein	112516	112364	-	hypothetical protein
contig_15	fig|6666666.28487.peg.572	Protein	113243	114352	+	hypothetical protein
contig_15	fig|6666666.28487.peg.573	Protein	114349	116133	+	hypothetical protein
contig_15	fig|6666666.28487.peg.574	Protein	116601	116458	-	hypothetical protein
contig_15	fig|6666666.28487.peg.575	Protein	117574	116552	-	hypothetical protein
contig_16	fig|6666666.28487.peg.576	Protein	176	1483	+	PE family protein
contig_16	fig|6666666.28487.peg.577	Protein	1890	1492	-	FIG00821641: hypothetical protein
contig_16	fig|6666666.28487.peg.578	Protein	2186	2662	+	integral membrane protein

contig_16	fig|6666666.28487.peg.579	Protein	2782	3090	+	hypothetical protein
contig_16	fig|6666666.28487.peg.580	Protein	3131	3442	+	hypothetical protein
contig_16	fig|6666666.28487.peg.581	Protein	3974	3489	-	hypothetical protein
contig_16	fig|6666666.28487.peg.582	Protein	4069	4914	+	Phage endolysin
contig_16	fig|6666666.28487.peg.583	Protein	7169	4911	-	Formate dehydrogenase-O, major subunit (EC 1.2.1.2)
contig_16	fig|6666666.28487.peg.584	Protein	7254	7856	+	Transcriptional regulator, TetR family
contig_16	fig|6666666.28487.peg.585	Protein	8842	7853	-	Dihydroflavonol-4-reductase (EC 1.1.1.219)
contig_16	fig|6666666.28487.peg.586	Protein	8981	10978	+	FIG00832845: hypothetical protein
contig_16	fig|6666666.28487.peg.587	Protein	11346	11570	+	hypothetical protein
contig_16	fig|6666666.28487.peg.588	Protein	11756	11616	-	Methylmalonate-semialdehyde dehydrogenase (EC 1.2.1.27)
contig_16	fig|6666666.28487.peg.589	Protein	11765	12169	+	Long-chain-fatty-acid--CoA ligase (EC 6.2.1.3)
contig_16	fig|6666666.28487.peg.590	Protein	12420	12644	+	hypothetical protein
contig_16	fig|6666666.28487.peg.591	Protein	13009	13377	+	hypothetical protein
contig_16	fig|6666666.28487.peg.592	Protein	13430	14899	+	ATP-binding region, ATPase-like:Histidine kinase, HAMP region:Histidine kinase A, N-terminal
contig_16	fig|6666666.28487.peg.593	Protein	14884	15549	+	two component system response regulator
contig_16	fig|6666666.28487.peg.594	Protein	15844	15602	-	hypothetical protein
contig_16	fig|6666666.28487.peg.595	Protein	16239	16361	+	hypothetical protein
contig_16	fig|6666666.28487.peg.596	Protein	16463	17383	+	putative DNA-binding protein
contig_16	fig|6666666.28487.peg.597	Protein	18015	17422	-	putative DNA-binding protein

contig_16	fig|6666666.28487.peg.598	Protein	18156	18326	+	hypothetical protein
contig_16	fig|6666666.28487.peg.599	Protein	18476	19417	+	short chain dehydrogenase(EC:1.1.1.-)
contig_16	fig|6666666.28487.peg.600	Protein	19543	19668	+	hypothetical protein
contig_16	fig|6666666.28487.peg.601	Protein	21962	19767	-	Periplasmic aromatic aldehyde oxidoreductase, molybdenum binding subunit YagR
contig_16	fig|6666666.28487.peg.602	Protein	22330	21959	-	Periplasmic aromatic aldehyde oxidoreductase, FAD binding subunit YagS
contig_16	fig|6666666.28487.peg.603	Protein	22935	22327	-	Periplasmic aromatic aldehyde oxidoreductase, FAD binding subunit YagS
contig_16	fig|6666666.28487.peg.604	Protein	23386	22937	-	Periplasmic aromatic aldehyde oxidoreductase, iron-sulfur subunit YagT
contig_16	fig|6666666.28487.peg.605	Protein	23249	23446	+	hypothetical protein
contig_16	fig|6666666.28487.peg.606	Protein	24934	24251	-	Pyridoxamine 5'-phosphate oxidase-related, FMN-binding
contig_16	fig|6666666.28487.peg.607	Protein	25350	25201	-	hypothetical protein
contig_16	fig|6666666.28487.peg.608	Protein	26338	25445	-	luciferase family protein
contig_16	fig|6666666.28487.peg.609	Protein	27284	27165	-	hypothetical protein
contig_16	fig|6666666.28487.peg.610	Protein	28458	27361	-	possible mcrC protein
contig_16	fig|6666666.28487.peg.611	Protein	30674	28461	-	ATPase associated with various cellular activities, AAA_5
contig_16	fig|6666666.28487.peg.612	Protein	32409	30787	-	hypothetical protein
contig_16	fig|6666666.28487.peg.613	Protein	36173	32406	-	hypothetical protein
contig_16	fig|6666666.28487.peg.614	Protein	37471	36281	-	predicted protein

contig_16	fig|6666666.28487.peg.615	Protein	37541	39544	+	hypothetical protein
contig_16	fig|6666666.28487.peg.616	Protein	39774	39652	-	hypothetical protein
contig_16	fig|6666666.28487.peg.617	Protein	39872	43420	+	ATP-dependent RNA helicase
contig_16	fig|6666666.28487.peg.618	Protein	43420	47475	+	Type II restriction enzyme, methylase subunits
contig_16	fig|6666666.28487.peg.619	Protein	48924	47596	-	Type II restriction enzyme, methylase subunits
contig_16	fig|6666666.28487.peg.620	Protein	48878	50245	+	DNA-invertase
contig_16	fig|6666666.28487.peg.621	Protein	50263	53961	+	helicase domain protein
contig_16	fig|6666666.28487.peg.622	Protein	53961	56012	+	FIG00732917: hypothetical protein
contig_16	fig|6666666.28487.peg.623	Protein	56068	56706	+	Phosphatidylserine/phosphatidylglycerophosphate/ cardiolipin synthases and related enzymes-like
contig_16	fig|6666666.28487.peg.624	Protein	58020	57007	-	hypothetical protein
contig_16	fig|6666666.28487.peg.625	Protein	58903	58025	-	hypothetical protein
contig_16	fig|6666666.28487.peg.626	Protein	59321	58953	-	hypothetical protein
contig_16	fig|6666666.28487.peg.627	Protein	60256	59402	-	hypothetical protein
contig_16	fig|6666666.28487.peg.628	Protein	60824	60297	-	hypothetical protein
contig_16	fig|6666666.28487.peg.629	Protein	61197	61039	-	hypothetical protein
contig_16	fig|6666666.28487.peg.630	Protein	61481	61206	-	hypothetical protein
contig_16	fig|6666666.28487.peg.631	Protein	63502	62504	-	ATP/GTP-binding protein
contig_16	fig|6666666.28487.peg.632	Protein	65569	63506	-	Integrase, catalytic region
contig_16	fig|6666666.28487.peg.633	Protein	66096	65566	-	hypothetical protein

contig_16	fig|6666666.28487.peg.634	Protein	67213	68271	+	hypothetical protein
contig_16	fig|6666666.28487.peg.635	Protein	68423	68542	+	hypothetical protein
contig_16	fig|6666666.28487.peg.636	Protein	69745	68660	-	Integrase
contig_16	fig|6666666.28487.peg.637	Protein	70609	71151	+	hypothetical protein
contig_16	fig|6666666.28487.peg.638	Protein	71148	73199	+	hypothetical protein
contig_16	fig|6666666.28487.peg.639	Protein	73202	74239	+	hypothetical protein
contig_16	fig|6666666.28487.peg.640	Protein	74568	74431	-	hypothetical protein
contig_16	fig|6666666.28487.peg.641	Protein	74899	75081	+	hypothetical protein
contig_16	fig|6666666.28487.peg.642	Protein	75205	77799	+	Type I restriction-modification system, restriction subunit R (EC 3.1.21.3)
contig_16	fig|6666666.28487.peg.643	Protein	77796	79229	+	Type I restriction-modification system, DNA-methyltransferase subunit M (EC 2.1.1.72)
contig_16	fig|6666666.28487.peg.644	Protein	79226	80605	+	Type I restriction-modification system, specificity subunit S (EC 3.1.21.3)
contig_16	fig|6666666.28487.peg.645	Protein	80743	83961	+	hypothetical protein
contig_16	fig|6666666.28487.peg.646	Protein	84582	84740	+	hypothetical protein
contig_17	fig|6666666.28487.peg.647	Protein	265	1365	+	Sulfate and thiosulfate binding protein CysP
contig_17	fig|6666666.28487.peg.648	Protein	1479	2234	+	Sulfate transport system permease protein CysT
contig_17	fig|6666666.28487.peg.649	Protein	2231	3049	+	Sulfate transport system permease protein CysW
contig_17	fig|6666666.28487.peg.650	Protein	3067	4092	+	Sulfate and thiosulfate import ATP-binding protein CysA (EC 3.6.3.25)

contig_17	fig|6666666.28487.peg.651	Protein	5092	4094	-	Magnesium and cobalt efflux protein CorC
contig_17	fig|6666666.28487.peg.652	Protein	6452	5085	-	Magnesium and cobalt efflux protein CorC
contig_17	fig|6666666.28487.peg.653	Protein	8011	6545	-	6-aminohexanoate-cyclic-dimer hydrolase
contig_17	fig|6666666.28487.peg.654	Protein	8100	9581	+	Carbohydrate kinase, FGGY
contig_17	fig|6666666.28487.peg.655	Protein	10374	9583	-	3-oxoacyl-[acyl-carrier protein] reductase (EC 1.1.1.100)
contig_17	fig|6666666.28487.peg.656	Protein	11396	10386	-	transcriptional regulator, putative
contig_17	fig|6666666.28487.peg.657	Protein	11544	11362	-	hypothetical protein
contig_17	fig|6666666.28487.peg.658	Protein	11578	12666	+	Sorbitol dehydrogenase (EC 1.1.1.14)
contig_17	fig|6666666.28487.peg.659	Protein	12731	14110	+	Various polyols ABC transporter, periplasmic substrate-binding protein
contig_17	fig|6666666.28487.peg.660	Protein	14125	15081	+	Maltose/maltodextrin ABC transporter, permease protein MalF
contig_17	fig|6666666.28487.peg.661	Protein	15086	15967	+	Maltose/maltodextrin ABC transporter, permease protein MalG
contig_17	fig|6666666.28487.peg.662	Protein	15967	17091	+	Glycerol-3-phosphate ABC transporter, ATP-binding protein UgpC (TC 3.A.1.1.3)
contig_17	fig|6666666.28487.peg.663	Protein	17088	18230	+	Maltose/maltodextrin transport ATP-binding protein MalK (EC 3.6.3.19)
contig_17	fig|6666666.28487.peg.664	Protein	18227	19972	+	Dihydroxyacetone kinase, ATP-dependent (EC 2.7.1.29)
contig_17	fig|6666666.28487.peg.665	Protein	19974	20462	+	Ribose 5-phosphate isomerase B (EC 5.3.1.6)
contig_17	fig|6666666.28487.peg.666	Protein	20459	22012	+	Inositol transport system ATP-binding protein
contig_17	fig|6666666.28487.peg.667	Protein	22009	23043	+	Inositol transport system permease protein

contig_17	fig|6666666.28487.peg.668	Protein	23087	24136	+	Inositol transport system sugar-binding protein
contig_17	fig|6666666.28487.peg.669	Protein	24133	24915	+	short chain dehydrogenase
contig_17	fig|6666666.28487.peg.670	Protein	25642	24938	-	Sirohydrochlorin ferrochelatase (EC 4.99.1.4)
contig_17	fig|6666666.28487.peg.671	Protein	26349	25639	-	Phosphoadenylyl-sulfate reductase [thioredoxin] (EC 1.8.4.8) / Adenylyl-sulfate reductase [thioredoxin] (EC 1.8.4.10)
contig_17	fig|6666666.28487.peg.672	Protein	28027	26354	-	Ferredoxin--sulfite reductase, actinobacterial type (EC 1.8.7.1)
contig_17	fig|6666666.28487.peg.673	Protein	28338	29513	+	Hypothetical radical SAM family enzyme in heat shock gene cluster, similarity with CPO of BS HemN-type
contig_17	fig|6666666.28487.peg.674	Protein	30377	29517	-	FIG00821822: hypothetical protein
contig_17	fig|6666666.28487.peg.675	Protein	30426	30803	+	FIG00827933: hypothetical protein
contig_17	fig|6666666.28487.peg.676	Protein	30933	36113	+	Malonyl CoA-acyl carrier protein transacylase (EC 2.3.1.39)
contig_17	fig|6666666.28487.peg.677	Protein	36237	37694	+	hypothetical protein
contig_17	fig|6666666.28487.peg.678	Protein	37792	38934	+	Transcriptional regulatory protein EmbR
contig_17	fig|6666666.28487.peg.679	Protein	38979	40478	+	Serine/threonine protein kinase (EC 2.7.11.1)
contig_17	fig|6666666.28487.peg.680	Protein	40583	41266	+	conserved hypothetical secreted protein
contig_17	fig|6666666.28487.peg.681	Protein	42970	41276	-	2,3-dihydroxybenzoate-AMP ligase (EC 2.7.7.58)
contig_17	fig|6666666.28487.peg.682	Protein	43079	46546	+	Phenyloxazoline synthase MbtB (EC 6.3.2.-)
contig_17	fig|6666666.28487.peg.683	Protein	46543	47286	+	Phenyloxazoline synthase MbtB (EC 6.3.2.-)
contig_17	fig|6666666.28487.peg.684	Protein	47439	48722	+	Malonyl CoA-acyl carrier protein transacylase (EC 2.3.1.39)
contig_17	fig|6666666.28487.peg.685	Protein	48722	51643	+	Polyketide synthetase MbtD

contig_17	fig|6666666.28487.peg.686	Protein	51636	56741	+	Long-chain-fatty-acid--CoA ligase (EC 6.2.1.3)
contig_17	fig|6666666.28487.peg.687	Protein	56752	61239	+	Long-chain-fatty-acid--CoA ligase (EC 6.2.1.3)
contig_17	fig|6666666.28487.peg.688	Protein	61236	62555	+	L-lysine 6-monooxygenase MbtG (EC 1.14.13.59)
contig_17	fig|6666666.28487.peg.689	Protein	62533	62742	+	MbtH-like protein
contig_17	fig|6666666.28487.peg.690	Protein	63085	62765	-	FIG00996129: hypothetical protein
contig_17	fig|6666666.28487.peg.691	Protein	63224	64255	+	Heat-inducible transcription repressor HrcA
contig_17	fig|6666666.28487.peg.692	Protein	64307	65455	+	Chaperone protein DnaJ
contig_17	fig|6666666.28487.peg.693	Protein	65479	66219	+	Ribosomal RNA small subunit methyltransferase E (EC 2.1.1.-)
contig_17	fig|6666666.28487.peg.694	Protein	66399	66788	+	membrane protein, MmpS family
contig_17	fig|6666666.28487.peg.695	Protein	66842	69646	+	Putative membrane protein mmpL4
contig_17	fig|6666666.28487.peg.696	Protein	69814	70776	+	Phosphate starvation-inducible protein PhoH, predicted ATPase
contig_17	fig|6666666.28487.peg.697	Protein	70791	71345	+	FIG000233: metal-dependent hydrolase
contig_17	fig|6666666.28487.peg.698	Protein	71342	72640	+	Magnesium and cobalt efflux protein CorC
contig_17	fig|6666666.28487.peg.699	Protein	72637	72963	+	FIG00821235: hypothetical protein
contig_17	fig|6666666.28487.peg.700	Protein	72960	73868	+	GTP-binding protein Era
contig_17	fig|6666666.28487.peg.701	Protein	74354	73869	-	FIG00825175: hypothetical protein
contig_17	fig|6666666.28487.peg.702	Protein	75858	74365	-	Asp-tRNAAsn/Glu-tRNAGln amidotransferase A subunit and related amidases
contig_17	fig|6666666.28487.peg.703	Protein	75970	76800	+	DNA recombination and repair protein RecO

contig_17	fig|6666666.28487.peg.704	Protein	76838	77629	+	Undecaprenyl pyrophosphate synthetase (EC 2.5.1.31)
contig_17	fig|6666666.28487.peg.705	Protein	77638	78057	+	FIG00820411: hypothetical protein
contig_17	fig|6666666.28487.peg.706	Protein	78068	78259	+	FIG00830288: hypothetical protein
contig_17	fig|6666666.28487.peg.707	Protein	78569	80047	+	possible sensory histidine kinase
contig_17	fig|6666666.28487.peg.708	Protein	80051	80302	+	Hemolysins and related proteins containing CBS domains
contig_17	fig|6666666.28487.peg.709	Protein	80289	81005	+	Hemolysins and related proteins containing CBS domains
contig_17	fig|6666666.28487.peg.710	Protein	81010	81312	+	Hemolysins and related proteins containing CBS domains
contig_17	fig|6666666.28487.peg.711	Protein	82969	81320	-	Glutathione-regulated potassium-efflux system protein KefB
contig_17	fig|6666666.28487.peg.712	Protein	83650	83258	-	protein of unknown function DUF302
contig_17	fig|6666666.28487.peg.713	Protein	83929	83660	-	FIG00995588: hypothetical protein
contig_17	fig|6666666.28487.peg.714	Protein	84166	85149	+	Universal stress protein family
contig_17	fig|6666666.28487.peg.715	Protein	85550	85146	-	Zinc uptake regulation protein ZUR
contig_17	fig|6666666.28487.peg.716	Protein	85909	85550	-	Transcriptional regulator, ArsR family
contig_17	fig|6666666.28487.peg.717	Protein	85996	87387	+	Glycyl-tRNA synthetase (EC 6.1.1.14)
contig_17	fig|6666666.28487.peg.718	Protein	89584	87548	-	protein of unknown function DUF477
contig_17	fig|6666666.28487.peg.719	Protein	89650	90924	+	Deoxyguanosinetriphosphate triphosphohydrolase (EC 3.1.5.1)
contig_17	fig|6666666.28487.peg.720	Protein	90956	92908	+	DNA primase (EC 2.7.7.-)
contig_17	fig|6666666.28487.peg.721	Protein	92969	93274	+	FIG00823654: hypothetical protein
contig_17	fig|6666666.28487.peg.722	Protein	93527	93276	-	FIG00822443: hypothetical protein

contig_17	fig|6666666.28487.rna.5	RNA	93683	93755	+	tRNA-Asn-GTT
contig_17	fig|6666666.28487.peg.723	Protein	93869	94369	+	Lipoprotein LppP
contig_17	fig|6666666.28487.peg.724	Protein	96337	94376	-	Acyl-coenzyme A oxidase 3, peroxisomal (EC 1.3.3.6)
contig_17	fig|6666666.28487.peg.725	Protein	96440	97174	+	nodulin 21-related protein
contig_17	fig|6666666.28487.peg.726	Protein	98446	97238	-	multidrug-efflux transporter, putative
contig_17	fig|6666666.28487.peg.727	Protein	98992	98552	-	Transcriptional regulator, MarR family
contig_17	fig|6666666.28487.peg.728	Protein	99107	101155	+	Candidate substrate-specific domain of ECF transporters in Mycobacteria / Duplicated ATPase component of energizing module of predicted ECF transporter in Mycobacteria
contig_17	fig|6666666.28487.peg.729	Protein	101152	102000	+	Transmembrane component of energizing module of ECF transporters in Mycobacteria
contig_17	fig|6666666.28487.peg.730	Protein	102017	103531	+	TldD family protein, Actinobacterial subgroup
contig_17	fig|6666666.28487.peg.731	Protein	103528	104901	+	TldE/PmbA family protein, Actinobacterial subgroup
contig_17	fig|6666666.28487.peg.732	Protein	104909	105748	+	FIG00824926: hypothetical protein
contig_17	fig|6666666.28487.rna.6	RNA	105787	105860	+	tRNA-Met-CAT
contig_17	fig|6666666.28487.peg.733	Protein	106367	105954	-	hypothetical protein
contig_17	fig|6666666.28487.peg.734	Protein	106628	106969	+	hypothetical protein
contig_17	fig|6666666.28487.peg.735	Protein	107087	107740	+	Membrane protein
contig_17	fig|6666666.28487.peg.736	Protein	108686	107793	-	Membrane protein, putative
contig_17	fig|6666666.28487.peg.737	Protein	108927	108673	-	oxidoreductase, short-chain dehydrogenase/reductase family
contig_17	fig|6666666.28487.peg.738	Protein	109487	108924	-	oxidoreductase, short-chain dehydrogenase/reductase family

contig_17	fig|6666666.28487.peg.739	Protein	109852	111222	+	Serine phosphatase RsbU, regulator of sigma subunit
contig_17	fig|6666666.28487.peg.740	Protein	111290	112255	+	Putative tricarboxylic transport TctC
contig_17	fig|6666666.28487.peg.741	Protein	112920	112252	-	FIG00823974: hypothetical protein
contig_17	fig|6666666.28487.peg.742	Protein	113037	113984	+	Potassium efflux system KefA protein / Small-conductance mechanosensitive channel
contig_17	fig|6666666.28487.peg.743	Protein	114439	113987	-	FIG00829901: hypothetical protein
contig_17	fig|6666666.28487.peg.744	Protein	114532	115980	+	Di-/tripeptide transporter
contig_17	fig|6666666.28487.peg.745	Protein	117590	115977	-	Phosphodiesterase/alkaline phosphatase D
contig_17	fig|6666666.28487.peg.746	Protein	118112	117609	-	Dihydroneopterin triphosphate pyrophosphohydolase, putative, Actinobacterial type, NudB-like
contig_17	fig|6666666.28487.peg.747	Protein	118195	118521	+	Uncharacterized conserved protein
contig_17	fig|6666666.28487.peg.748	Protein	118590	119513	+	hypothetical protein
contig_17	fig|6666666.28487.peg.749	Protein	119765	122344	+	Thermolysin precursor (EC 3.4.24.27) (Thermostable neutral proteinase)
contig_17	fig|6666666.28487.peg.750	Protein	123004	122327	-	FIG00829219: hypothetical protein
contig_17	fig|6666666.28487.peg.751	Protein	123315	123022	-	alpha/beta hydrolase fold
contig_17	fig|6666666.28487.peg.752	Protein	123481	124077	+	FIG00824381: hypothetical protein
contig_17	fig|6666666.28487.peg.753	Protein	124080	124406	+	branched-chain amino acid transport
contig_17	fig|6666666.28487.peg.754	Protein	124963	124376	-	Transcriptional regulator, TetR family
contig_17	fig|6666666.28487.peg.755	Protein	125097	126125	+	FIG00833095: hypothetical protein
contig_17	fig|6666666.28487.peg.756	Protein	126145	127218	+	hypothetical protein

contig_17	fig|6666666.28487.peg.757	Protein	128503	127208	-	hypothetical protein
contig_17	fig|6666666.28487.peg.758	Protein	129466	128720	-	short chain dehydrogenase
contig_17	fig|6666666.28487.peg.759	Protein	131087	129489	-	Dipeptide transport ATP-binding protein DppD (TC 3.A.1.5.2)
contig_17	fig|6666666.28487.peg.760	Protein	132028	131084	-	Dipeptide transport system permease protein DppC (TC 3.A.1.5.2)
contig_17	fig|6666666.28487.peg.761	Protein	132972	132025	-	Dipeptide transport system permease protein DppB (TC 3.A.1.5.2)
contig_17	fig|6666666.28487.peg.762	Protein	134513	132975	-	Dipeptide-binding ABC transporter, periplasmic substrate-binding component (TC 3.A.1.5.2)
contig_17	fig|6666666.28487.peg.763	Protein	134645	135160	+	FIG00820951: hypothetical protein
contig_17	fig|6666666.28487.peg.764	Protein	135129	136181	+	FIG00827002: hypothetical protein
contig_17	fig|6666666.28487.peg.765	Protein	137339	136260	-	S-adenosyl-L-methionine:salicylic acid carboxyl methyltransferase-like protein
contig_17	fig|6666666.28487.peg.766	Protein	137405	138142	+	FIG00820673: hypothetical protein
contig_17	fig|6666666.28487.peg.767	Protein	138762	138139	-	Putative hydrolase in cluster with formaldehyde/S-nitrosomycothiol reductase MscR
contig_17	fig|6666666.28487.peg.768	Protein	139847	138762	-	Formaldehyde dehydrogenase MscR, NAD/mycothiol-dependent (EC 1.2.1.66) / S-nitrosomycothiol reductase MscR
contig_17	fig|6666666.28487.peg.769	Protein	139992	141620	+	Tetratricopeptide repeat-containing protein / Transcriptional regulator, LuxR family
contig_17	fig|6666666.28487.peg.770	Protein	141717	142790	+	Possible transcriptional regulatory protein
contig_17	fig|6666666.28487.peg.771	Protein	142818	143648	+	Beta-lactamase class C and other penicillin binding proteins

contig_17	fig|6666666.28487.peg.772	Protein	143836	143687	-	hypothetical protein
contig_17	fig|6666666.28487.peg.773	Protein	143787	144299	+	FIG00820627: hypothetical protein
contig_17	fig|6666666.28487.peg.774	Protein	144583	144296	-	hypothetical protein
contig_17	fig|6666666.28487.peg.775	Protein	145484	144594	-	Diacylglycerol kinase-related protein
contig_17	fig|6666666.28487.peg.776	Protein	147123	145507	-	Flavoprotein
contig_17	fig|6666666.28487.peg.777	Protein	147141	147719	+	Transcriptional regulator, TetR family
contig_17	fig|6666666.28487.peg.778	Protein	147716	149290	+	Glycerol-3-phosphate dehydrogenase (EC 1.1.5.3)
contig_17	fig|6666666.28487.peg.779	Protein	150752	149322	-	Propionyl-CoA carboxylase beta chain (EC 6.4.1.3)
contig_17	fig|6666666.28487.peg.780	Protein	152020	150767	-	3-oxoacyl-[acyl-carrier-protein] synthase, KASII (EC 2.3.1.41)
contig_17	fig|6666666.28487.peg.781	Protein	153271	152063	-	3-oxoacyl-[acyl-carrier-protein] synthase, KASII (EC 2.3.1.41)
contig_17	fig|6666666.28487.peg.782	Protein	153609	153310	-	Acyl carrier protein
contig_17	fig|6666666.28487.peg.783	Protein	154620	153685	-	Malonyl CoA-acyl carrier protein transacylase (EC 2.3.1.39)
contig_17	fig|6666666.28487.peg.784	Protein	156038	154755	-	Regulator of polyketide synthase expression
contig_17	fig|6666666.28487.peg.785	Protein	158935	156146	-	Pyruvate dehydrogenase E1 component (EC 1.2.4.1)
contig_17	fig|6666666.28487.peg.786	Protein	159122	159697	+	FIG00820323: hypothetical protein
contig_17	fig|6666666.28487.peg.787	Protein	159796	160221	+	FIG00819990: hypothetical protein
contig_17	fig|6666666.28487.peg.788	Protein	160218	160682	+	Alkyl hydroperoxide reductase subunit C-like protein
contig_17	fig|6666666.28487.rna.7	RNA	160729	160800	+	tRNA-Val-TAC
contig_17	fig|6666666.28487.peg.789	Protein	161440	160820	-	expressed protein

contig_17	fig|6666666.28487.peg.790	Protein	163173	161458	-	FIG00825306: hypothetical protein
contig_17	fig|6666666.28487.peg.791	Protein	163231	164184	+	Adenosylcobinamide-phosphate synthase
contig_17	fig|6666666.28487.peg.792	Protein	164974	164138	-	Cytochrome oxidase biogenesis protein Surf1, facilitates heme A insertion
contig_17	fig|6666666.28487.peg.793	Protein	165470	164976	-	Low molecular weight protein tyrosine phosphatase (EC 3.1.3.48)
contig_17	fig|6666666.28487.peg.794	Protein	166140	165463	-	Phosphoglycolate phosphatase (EC 3.1.3.18)
contig_17	fig|6666666.28487.peg.795	Protein	166215	167351	+	FIG042796: Hypothetical protein
contig_17	fig|6666666.28487.peg.796	Protein	167341	168078	+	FIG137478: Hypothetical protein
contig_17	fig|6666666.28487.peg.797	Protein	168075	169151	+	FIG006762: Phosphoglycerate mutase family
contig_17	fig|6666666.28487.peg.798	Protein	169729	169142	-	transcriptional regulator, Cro-CI family, putative
contig_17	fig|6666666.28487.peg.799	Protein	169777	170697	+	Thioredoxin reductase (EC 1.8.1.9)
contig_17	fig|6666666.28487.peg.800	Protein	170690	171313	+	Thioredoxin reductase (EC 1.8.1.9)
contig_17	fig|6666666.28487.peg.801	Protein	171344	172627	+	CONSERVED 13E12 REPEAT FAMILY PROTEIN
contig_17	fig|6666666.28487.peg.802	Protein	173071	172637	-	putative lyase
contig_17	fig|6666666.28487.peg.803	Protein	173513	173097	-	Transcriptional regulator, AraC family
contig_17	fig|6666666.28487.peg.804	Protein	174091	174468	+	hypothetical protein
contig_17	fig|6666666.28487.peg.805	Protein	175122	175316	+	hypothetical protein
contig_17	fig|6666666.28487.peg.806	Protein	175473	176219	+	Phage Gp37Gp68 protein
contig_17	fig|6666666.28487.peg.807	Protein	177327	176212	-	hypothetical protein

contig_17	fig|6666666.28487.peg.808	Protein	177552	178754	+	HNH homing endonuclease
contig_17	fig|6666666.28487.peg.809	Protein	180491	178983	-	Chaperonin GroEL (HSP60 family)
contig_17	fig|6666666.28487.peg.810	Protein	180656	182425	+	Long-chain-fatty-acid--CoA ligase (EC 6.2.1.3)
contig_17	fig|6666666.28487.peg.811	Protein	182450	183229	+	Enoyl-CoA hydratase (EC 4.2.1.17)
contig_17	fig|6666666.28487.peg.812	Protein	184080	183238	-	3-methyl-2-oxobutanoate hydroxymethyltransferase (EC 2.1.2.11)
contig_17	fig|6666666.28487.peg.813	Protein	184240	185634	+	Wax ester synthase/acyl-CoA:diacylglycerol acyltransferase
contig_17	fig|6666666.28487.peg.814	Protein	185699	187231	+	Probable exported protease [EC:3.4.-.-]
contig_17	fig|6666666.28487.peg.815	Protein	187329	188837	+	Putative hydrolase
contig_17	fig|6666666.28487.peg.816	Protein	188883	190223	+	Glutamine synthetase type I (EC 6.3.1.2)
contig_17	fig|6666666.28487.peg.817	Protein	190254	193232	+	Glutamate-ammonia-ligase adenylyltransferase (EC 2.7.7.42)
contig_17	fig|6666666.28487.peg.818	Protein	193238	193867	+	FIG00822716: hypothetical protein
contig_17	fig|6666666.28487.peg.819	Protein	193864	194724	+	FIG00820321: hypothetical protein
contig_17	fig|6666666.28487.peg.820	Protein	195236	194721	-	hypothetical protein
contig_17	fig|6666666.28487.peg.821	Protein	196840	195404	-	Glutamine synthetase type I (EC 6.3.1.2)
contig_17	fig|6666666.28487.peg.822	Protein	196988	197455	+	PROBABLE MEMBRANE PROTEIN
contig_17	fig|6666666.28487.peg.823	Protein	199282	197456	-	PROBABLE SIGNAL PEPTIDE PROTEIN
contig_17	fig|6666666.28487.peg.824	Protein	200041	199289	-	Transmembrane protein MT2276, clustered with lipoate gene
contig_17	fig|6666666.28487.peg.825	Protein	200987	200025	-	Lipoate synthase
contig_17	fig|6666666.28487.peg.826	Protein	201682	200984	-	Octanoate-[acyl-carrier-protein]-protein-N-octanoyltransferase

contig_17	fig|6666666.28487.peg.827	Protein	202610	201693	-	Cell division inhibitor
contig_17	fig|6666666.28487.peg.828	Protein	204413	202617	-	Dihydrolipoamide acyltransferase component of branched-chain alpha-keto acid dehydrogenase complex (EC 2.3.1.168)
contig_17	fig|6666666.28487.peg.829	Protein	204574	204927	+	Putative oxidoreductase
contig_17	fig|6666666.28487.peg.830	Protein	204949	206379	+	Oxidoreductase, short-chain dehydrogenase/reductase family (EC 1.1.1.-)
contig_17	fig|6666666.28487.peg.831	Protein	207939	206419	-	Cytosol aminopeptidase PepA (EC 3.4.11.1)
contig_17	fig|6666666.28487.peg.832	Protein	207981	209078	+	Aminomethyltransferase (glycine cleavage system T protein) (EC 2.1.2.10)
contig_17	fig|6666666.28487.peg.833	Protein	209111	210226	+	Branched-chain amino acid aminotransferase (EC 2.6.1.42)
contig_17	fig|6666666.28487.peg.834	Protein	210262	210459	+	hypothetical protein
contig_17	fig|6666666.28487.peg.835	Protein	211205	210477	-	Cobalamin synthase
contig_17	fig|6666666.28487.peg.836	Protein	212276	211221	-	Nicotinate-nucleotide--dimethylbenzimidazole phosphoribosyltransferase (EC 2.4.2.21)
contig_17	fig|6666666.28487.peg.837	Protein	212959	212273	-	CblZ, a non-orthologous displasment for Alpha-ribazole-5'-phosphate phosphatase
contig_17	fig|6666666.28487.peg.838	Protein	213018	214100	+	Glycerate kinase (EC 2.7.1.31)
contig_17	fig|6666666.28487.peg.839	Protein	214237	214590	+	probable iron binding protein from the HesB_IscA_SufA family
contig_17	fig|6666666.28487.peg.840	Protein	215209	214592	-	putative conserved membrane protein
contig_17	fig|6666666.28487.peg.841	Protein	215437	216408	+	Adenosine kinase (EC 2.7.1.20)
contig_17	fig|6666666.28487.peg.842	Protein	218292	216427	-	Asparagine synthetase [glutamine-hydrolyzing] (EC 6.3.5.4)

contig_17	fig|6666666.28487.peg.843	Protein	218263	218514	+	hypothetical protein
contig_17	fig|6666666.28487.peg.844	Protein	218553	219587	+	Cytochrome c oxidase polypeptide II (EC 1.9.3.1)
contig_17	fig|6666666.28487.peg.845	Protein	219597	220019	+	Probable cytochrome c oxidase polypeptide 4 (EC 1.9.3.1)
contig_17	fig|6666666.28487.peg.846	Protein	220173	221063	+	Putative membrane protein MmpS3
contig_17	fig|6666666.28487.peg.847	Protein	221079	221759	+	hypothetical protein
contig_17	fig|6666666.28487.peg.848	Protein	223626	221977	-	Ubiquinol--cytochrome c reductase, cytochrome B subunit (EC 1.10.2.2)
contig_17	fig|6666666.28487.peg.849	Protein	224831	223623	-	Ubiquinol-cytochrome C reductase iron-sulfur subunit (EC 1.10.2.2)
contig_17	fig|6666666.28487.peg.850	Protein	225676	224828	-	ubiquinol cytochrome C oxidoreductase, cytochrome C1 subunit
contig_17	fig|6666666.28487.peg.851	Protein	226230	225688	-	Cytochrome c oxidase polypeptide III (EC 1.9.3.1)
contig_17	fig|6666666.28487.peg.852	Protein	227306	226296	-	Cytochrome c oxidase polypeptide III (EC 1.9.3.1)
contig_17	fig|6666666.28487.peg.853	Protein	229169	227313	-	DNA polymerase III epsilon subunit (EC 2.7.7.7)
contig_17	fig|6666666.28487.peg.854	Protein	229308	229550	+	FIG00824215: hypothetical protein
contig_17	fig|6666666.28487.peg.855	Protein	229769	230869	+	Probable endopeptidase
contig_17	fig|6666666.28487.peg.856	Protein	231042	231674	+	FIG00821136: hypothetical protein
contig_17	fig|6666666.28487.peg.857	Protein	231671	232807	+	Poly(glycerol-phosphate) alpha-glucosyltransferase (EC 2.4.1.52)
contig_17	fig|6666666.28487.peg.858	Protein	234612	232804	-	Long-chain-fatty-acid--CoA ligase (EC 6.2.1.3)
contig_17	fig|6666666.28487.peg.859	Protein	234706	235101	+	FIG00995034: hypothetical protein

contig_17	fig|6666666.28487.peg.860	Protein	235174	235611	+	FIG00821549: hypothetical protein
contig_17	fig|6666666.28487.peg.861	Protein	235712	236863	+	Arsenical pump-driving ATPase (EC 3.6.3.16)
contig_17	fig|6666666.28487.peg.862	Protein	236860	237258	+	FIG00821592: hypothetical protein
contig_17	fig|6666666.28487.peg.863	Protein	237375	238097	+	1-acyl-sn-glycerol-3-phosphate acyltransferase (EC 2.3.1.51)
contig_17	fig|6666666.28487.peg.864	Protein	239320	238094	-	Possible membrane protein
contig_17	fig|6666666.28487.peg.865	Protein	239504	240328	+	Probable conserved integral membrane protein
contig_17	fig|6666666.28487.peg.866	Protein	240329	240808	+	FIG00820624: hypothetical protein
contig_17	fig|6666666.28487.peg.867	Protein	240859	242271	+	2-keto-3-deoxy-D-arabino-heptulosonate-7-phosphate synthase II (EC 2.5.1.54)
contig_17	fig|6666666.28487.peg.868	Protein	243830	242295	-	Probable serine/threonine-protein kinase pknL (EC 2.7.11.1)
contig_17	fig|6666666.28487.peg.869	Protein	245517	243925	-	Carotene biosynthesis associated membrane protein
contig_17	fig|6666666.28487.peg.870	Protein	246613	245534	-	Geranylgeranyl diphosphate synthase (EC 2.5.1.29)
contig_17	fig|6666666.28487.peg.871	Protein	247391	246702	-	Lipoprotein LppM
contig_17	fig|6666666.28487.peg.872	Protein	248017	247427	-	Acetyltransferase (EC 2.3.1.-)
contig_17	fig|6666666.28487.peg.873	Protein	248231	248632	+	putative conserved transmembrane protein
contig_17	fig|6666666.28487.peg.874	Protein	248928	249362	+	Cell division protein MraZ
contig_17	fig|6666666.28487.peg.875	Protein	249499	250461	+	rRNA small subunit methyltransferase H
contig_17	fig|6666666.28487.peg.876	Protein	250464	251546	+	FIG034299: proline rich membrane protein
contig_17	fig|6666666.28487.peg.877	Protein	251543	253486	+	Cell division protein FtsI [Peptidoglycan synthetase] (EC 2.4.1.129)
contig_17	fig|6666666.28487.peg.878	Protein	253550	255070	+	UDP-N-acetylmuramoylalanyl-D-glutamate--2,6-

						diaminopimelate ligase (EC 6.3.2.13)
contig_17	fig|6666666.28487.peg.879	Protein	255067	256560	+	UDP-N-acetylmuramoylalanyl-D-glutamyl-2,6-diaminopimelate--D-alanyl-D-alanine ligase (EC 6.3.2.10)
contig_17	fig|6666666.28487.peg.880	Protein	256557	257636	+	Phospho-N-acetylmuramoyl-pentapeptide-transferase (EC 2.7.8.13)
contig_17	fig|6666666.28487.peg.881	Protein	257782	259083	+	UDP-N-acetylmuramoylalanine--D-glutamate ligase (EC 6.3.2.9)
contig_17	fig|6666666.28487.peg.882	Protein	259088	260623	+	Cell division protein FtsW
contig_17	fig|6666666.28487.peg.883	Protein	260745	260614	-	hypothetical protein
contig_17	fig|6666666.28487.peg.884	Protein	260620	261795	+	UDP-N-acetylglucosamine--N-acetylmuramyl-(pentapeptide) pyrophosphoryl-undecaprenol N-acetylglucosamine transferase (EC 2.4.1.227)
contig_17	fig|6666666.28487.peg.885	Protein	261792	263201	+	UDP-N-acetylmuramate--alanine ligase (EC 6.3.2.8)
contig_17	fig|6666666.28487.peg.886	Protein	263222	264127	+	Cell division protein FtsQ
contig_17	fig|6666666.28487.peg.887	Protein	264314	265489	+	Cell division protein FtsZ (EC 3.4.24.-)
contig_17	fig|6666666.28487.peg.888	Protein	265527	266219	+	COG1496: Uncharacterized conserved protein
contig_17	fig|6666666.28487.peg.889	Protein	266216	267043	+	Hypothetical protein YggS, proline synthase co-transcribed bacterial homolog PROSC
contig_17	fig|6666666.28487.peg.890	Protein	267040	267684	+	FIG021292: hypothetical protein
contig_17	fig|6666666.28487.peg.891	Protein	267794	268084	+	FIG021764: Possible membrane protein
contig_17	fig|6666666.28487.peg.892	Protein	268330	269124	+	FIG055075: Possibly a cell division protein, antigen 84 in Mycobacteria

contig_17	fig|6666666.28487.peg.893	Protein	269155	269502	+	Probable transmembrane protein
contig_17	fig|6666666.28487.peg.894	Protein	270174	269512	-	Phosphoribosyl transferase domain protein
contig_17	fig|6666666.28487.peg.895	Protein	270242	271591	+	Cell division inhibitor
contig_17	fig|6666666.28487.peg.896	Protein	272304	271573	-	Glutamine amidotransferase class-I (EC 6.3.5.2)
contig_17	fig|6666666.28487.peg.897	Protein	272361	272768	+	hypothetical protein
contig_17	fig|6666666.28487.peg.898	Protein	273153	272854	-	hypothetical protein
contig_17	fig|6666666.28487.peg.899	Protein	273487	273308	-	hypothetical protein
contig_17	fig|6666666.28487.peg.900	Protein	273999	281279	+	Endoglucanase E1 precursor (EC 3.2.1.4) (Endo-1,4-beta-glucanase E1) (Cellulase E1) (Endocellulase E1)
contig_17	fig|6666666.28487.peg.901	Protein	281957	281286	-	Response regulator CitB of citrate metabolism
contig_17	fig|6666666.28487.peg.902	Protein	283596	281950	-	Signal transduction histidine kinase CitA regulating citrate metabolism
contig_17	fig|6666666.28487.peg.903	Protein	283643	284632	+	TctC citrate transporter
contig_17	fig|6666666.28487.peg.904	Protein	284629	285165	+	TctB citrate transporter
contig_17	fig|6666666.28487.peg.905	Protein	285166	286707	+	TctA citrate transporter
contig_17	fig|6666666.28487.peg.906	Protein	286736	287128	+	UspA
contig_17	fig|6666666.28487.peg.907	Protein	287152	288117	+	hydrolase
contig_17	fig|6666666.28487.peg.908	Protein	288500	288210	-	Nitrite reductase [NAD(P)H] small subunit (EC 1.7.1.4)
contig_17	fig|6666666.28487.peg.909	Protein	290965	288497	-	Nitrite reductase [NAD(P)H] large subunit (EC 1.7.1.4)
contig_17	fig|6666666.28487.peg.910	Protein	291321	290968	-	hypothetical protein

contig_17	fig|6666666.28487.peg.911	Protein	291498	292907	+	Nitrate/nitrite transporter
contig_17	fig|6666666.28487.peg.912	Protein	292959	296858	+	Assimilatory nitrate reductase large subunit (EC:1.7.99.4)
contig_17	fig|6666666.28487.peg.913	Protein	298153	296837	-	FIG00828488: hypothetical protein
contig_17	fig|6666666.28487.peg.914	Protein	298846	298292	-	FIG00823976: hypothetical protein
contig_17	fig|6666666.28487.peg.915	Protein	300006	298906	-	Alcohol dehydrogenase (EC 1.1.1.1)
contig_17	fig|6666666.28487.peg.916	Protein	301502	300003	-	Acetyl-CoA acetyltransferase
contig_17	fig|6666666.28487.peg.917	Protein	301725	302234	+	Transcriptional regulator, TetR family
contig_17	fig|6666666.28487.peg.918	Protein	302280	302717	+	Phenylacetic acid degradation protein PaaI
contig_17	fig|6666666.28487.peg.919	Protein	302717	303628	+	BpoB
contig_17	fig|6666666.28487.peg.920	Protein	303701	304300	+	Transcriptional regulator, TetR family
contig_17	fig|6666666.28487.peg.921	Protein	305498	304317	-	Ferredoxin reductase
contig_17	fig|6666666.28487.peg.922	Protein	306901	305513	-	putative cytochrome P450 hydroxylase
contig_17	fig|6666666.28487.peg.923	Protein	307252	306932	-	Ferredoxin, 2Fe-2S
contig_17	fig|6666666.28487.peg.924	Protein	307367	308371	+	Transcriptional regulator, AraC family
contig_17	fig|6666666.28487.peg.925	Protein	308652	309818	+	FIG00832116: hypothetical protein
contig_17	fig|6666666.28487.peg.926	Protein	309815	310252	+	FIG309211: hypothetical protein
contig_17	fig|6666666.28487.peg.927	Protein	310270	311292	+	F420-dependent N(5),N(10)-methylenetetrahydromethanopterin reductase (EC 1.5.99.11)
contig_17	fig|6666666.28487.peg.928	Protein	311285	311743	+	FIG00820994: hypothetical protein
contig_17	fig|6666666.28487.peg.929	Protein	311740	312165	+	MaoC family protein

contig_17	fig|6666666.28487.peg.930	Protein	312162	312689	+	FIG00829314: hypothetical protein
contig_17	fig|6666666.28487.peg.931	Protein	312686	313156	+	FIG00822566: hypothetical protein
contig_17	fig|6666666.28487.peg.932	Protein	313200	313967	+	Enoyl-CoA hydratase (EC 4.2.1.17)
contig_17	fig|6666666.28487.peg.933	Protein	313969	314781	+	FIG00825734: hypothetical protein
contig_17	fig|6666666.28487.peg.934	Protein	314778	316502	+	FIG00820360: hypothetical protein
contig_17	fig|6666666.28487.peg.935	Protein	317092	316499	-	Transcriptional regulator, TetR family
contig_17	fig|6666666.28487.peg.936	Protein	318865	317312	-	Long-chain-fatty-acid--CoA ligase (EC 6.2.1.3)
contig_17	fig|6666666.28487.peg.937	Protein	320046	318892	-	Isovaleryl-CoA dehydrogenase (EC 1.3.99.10)
contig_17	fig|6666666.28487.peg.938	Protein	322134	320101	-	Methylcrotonyl-CoA carboxylase biotin-containing subunit (EC 6.4.1.4)
contig_17	fig|6666666.28487.peg.939	Protein	323741	322140	-	Methylcrotonyl-CoA carboxylase carboxyl transferase subunit (EC 6.4.1.4)
contig_17	fig|6666666.28487.peg.940	Protein	324699	324085	-	Transcriptional regulator, TetR family
contig_17	fig|6666666.28487.peg.941	Protein	324829	325698	+	Metal-dependent hydrolase
contig_17	fig|6666666.28487.peg.942	Protein	326389	325880	-	Transcriptional regulator, TetR family
contig_17	fig|6666666.28487.peg.943	Protein	326764	327495	+	Oxidoreductase, short chain dehydrogenase/reductase family
contig_17	fig|6666666.28487.peg.944	Protein	327509	328432	+	FIG00823655: hypothetical protein
contig_17	fig|6666666.28487.peg.945	Protein	328429	329922	+	Cyclohexanone monooxygenase (EC 1.14.13.22)
contig_17	fig|6666666.28487.peg.946	Protein	329967	331484	+	Cyclohexanone monooxygenase (EC 1.14.13.22)
contig_17	fig|6666666.28487.peg.947	Protein	332308	332144	-	hypothetical protein

contig_17	fig|6666666.28487.peg.948	Protein	332277	334052	+	Cyclohexanone monooxygenase (EC 1.14.13.22)
contig_18	fig|6666666.28487.peg.949	Protein	53	382	+	protein of unknown function DUF222
contig_18	fig|6666666.28487.peg.950	Protein	394	774	+	FIG00831791: hypothetical protein
contig_18	fig|6666666.28487.peg.951	Protein	1796	771	-	drug transport protein, putative
contig_18	fig|6666666.28487.peg.952	Protein	2471	1818	-	drug transport protein, putative
contig_18	fig|6666666.28487.peg.953	Protein	2657	4186	+	Hydroxymethylpyrimidine phosphate synthase ThiC
contig_18	fig|6666666.28487.peg.954	Protein	4183	5019	+	Hydroxymethylpyrimidine phosphate kinase ThiD (EC 2.7.4.7)
contig_18	fig|6666666.28487.peg.955	Protein	5016	5630	+	FIG022825: hypothetical protein
contig_18	fig|6666666.28487.peg.956	Protein	5645	6355	+	FIG01256597: hypothetical protein
contig_18	fig|6666666.28487.peg.957	Protein	6392	7879	+	Cyclohexanone monooxygenase (EC 1.14.13.22)
contig_18	fig|6666666.28487.peg.958	Protein	7889	8155	+	Putative membrane protein
contig_18	fig|6666666.28487.peg.959	Protein	8168	9418	+	amino acid permease family protein
contig_18	fig|6666666.28487.peg.960	Protein	9426	10703	+	Uracil permease
contig_18	fig|6666666.28487.peg.961	Protein	12123	10657	-	Aminopeptidase Y (Arg, Lys, Leu preference) (EC 3.4.11.15)
contig_18	fig|6666666.28487.peg.962	Protein	13619	12120	-	Aminopeptidase Y (Arg, Lys, Leu preference) (EC 3.4.11.15)
contig_18	fig|6666666.28487.peg.963	Protein	14550	13630	-	EphC
contig_18	fig|6666666.28487.peg.964	Protein	15301	14552	-	Putative ABC transport system membrane protein
contig_18	fig|6666666.28487.peg.965	Protein	16215	15298	-	ABC transporter, ATP-binding protein
contig_18	fig|6666666.28487.peg.966	Protein	16958	16212	-	putative secreted hydrolase

contig_18	fig|6666666.28487.peg.967	Protein	17757	16975	-	Thiazole biosynthesis protein ThiG
contig_18	fig|6666666.28487.peg.968	Protein	17935	17750	-	Sulfur carrier protein ThiS
contig_18	fig|6666666.28487.peg.969	Protein	18960	17944	-	Glycine oxidase ThiO (EC 1.4.3.19)
contig_18	fig|6666666.28487.peg.970	Protein	19097	19744	+	Thiamin-phosphate pyrophosphorylase (EC 2.5.1.3)
contig_18	fig|6666666.28487.peg.971	Protein	20006	19728	-	mutT3
contig_18	fig|6666666.28487.peg.972	Protein	20356	21675	+	Possible membrane protein
contig_18	fig|6666666.28487.peg.973	Protein	21675	22670	+	"Glutamine ABC transporter, periplasmic glutamine-binding protein (TC 3.A.1.3.2)"
contig_18	fig|6666666.28487.peg.974	Protein	22670	24988	+	serine/threonine protein kinase
contig_18	fig|6666666.28487.peg.975	Protein	25589	24978	-	transcriptional regulator, TetR family
contig_18	fig|6666666.28487.peg.976	Protein	26669	25599	-	hypothetical protein
contig_18	fig|6666666.28487.peg.977	Protein	27549	26683	-	FIG00822389: hypothetical protein
contig_18	fig|6666666.28487.peg.978	Protein	29434	28280	-	Acetate kinase (EC 2.7.2.1)
contig_18	fig|6666666.28487.peg.979	Protein	31521	29431	-	Phosphate acetyltransferase (EC 2.3.1.8)
contig_18	fig|6666666.28487.peg.980	Protein	32515	31544	-	Integral membrane protein
contig_18	fig|6666666.28487.peg.981	Protein	33538	32528	-	F420-dependent glucose-6-phosphate dehydrogenase
contig_18	fig|6666666.28487.peg.982	Protein	33562	34296	+	Beta-lactamase-like protein
contig_18	fig|6666666.28487.peg.983	Protein	34563	34288	-	FIG00821848: hypothetical protein
contig_18	fig|6666666.28487.peg.984	Protein	35236	34589	-	Nitrilotriacetate monooxygenase component A (EC 1.14.13.-)
contig_18	fig|6666666.28487.peg.985	Protein	36339	35218	-	luciferase family protein

contig_18	fig|6666666.28487.peg.986	Protein	37256	36336	-	fmnh2-utilizing oxygenase
contig_18	fig|6666666.28487.peg.987	Protein	37732	37253	-	GCN5-related N-acetyltransferase
contig_18	fig|6666666.28487.peg.988	Protein	38148	37729	-	Probable glutathione S-transferase-related transmembrane protein (EC 2.5.1.18)
contig_18	fig|6666666.28487.peg.989	Protein	38480	38163	-	Transcriptional regulator, ArsR family
contig_18	fig|6666666.28487.peg.990	Protein	38573	39280	+	FIG172199: hypothetical thioredoxin family protein
contig_18	fig|6666666.28487.peg.991	Protein	39267	39911	+	FIG00827796: hypothetical protein
contig_18	fig|6666666.28487.peg.992	Protein	39908	40645	+	hypothetical protein
contig_18	fig|6666666.28487.peg.993	Protein	40666	41529	+	short chain dehydrogenase family protein [imported]
contig_18	fig|6666666.28487.peg.994	Protein	41930	41526	-	FIG00821188: hypothetical protein
contig_18	fig|6666666.28487.peg.995	Protein	43145	41931	-	O-acetylhomoserine sulfhydrylase (EC 2.5.1.49) / O-succinylhomoserine sulfhydrylase (EC 2.5.1.48)
contig_18	fig|6666666.28487.peg.996	Protein	43555	43142	-	Rhodanese-related sulfurtransferase
contig_18	fig|6666666.28487.peg.997	Protein	44027	43575	-	FIG00826202: hypothetical protein
contig_18	fig|6666666.28487.peg.998	Protein	45139	44024	-	Phosphoribosylglycinamide formyltransferase 2 (EC 2.1.2.-)
contig_18	fig|6666666.28487.peg.999	Protein	45836	45396	-	Transcriptional regulator, MarR family
contig_18	fig|6666666.28487.peg.1000	Protein	45928	48024	+	FIG00821761: hypothetical protein
contig_18	fig|6666666.28487.peg.1001	Protein	48060	49268	+	putative cytochrome P450 hydroxylase
contig_18	fig|6666666.28487.peg.1002	Protein	49265	49462	+	putative ferredoxin
contig_18	fig|6666666.28487.peg.1003	Protein	50099	49419	-	FIG00825411: hypothetical protein

contig_18	fig|6666666.28487.peg.1004	Protein	51207	50104	-	Putative membrane protein
contig_18	fig|6666666.28487.peg.1005	Protein	51866	51228	-	FIG00822066: hypothetical protein
contig_18	fig|6666666.28487.peg.1006	Protein	53152	51863	-	Adenylosuccinate synthetase (EC 6.3.4.4)
contig_18	fig|6666666.28487.peg.1007	Protein	53307	53194	-	hypothetical protein
contig_18	fig|6666666.28487.peg.1008	Protein	53275	53859	+	FIG00820475: hypothetical protein
contig_18	fig|6666666.28487.peg.1009	Protein	53870	54649	+	Possible membrane protein
contig_18	fig|6666666.28487.peg.1010	Protein	54741	55589	+	Cobalt-zinc-cadmium resistance protein CzcD
contig_18	fig|6666666.28487.peg.1011	Protein	55990	55586	-	hypothetical protein
contig_18	fig|6666666.28487.peg.1012	Protein	56110	56829	+	PROBABLE CONSERVED MEMBRANE PROTEIN
contig_18	fig|6666666.28487.peg.1013	Protein	58300	56936	-	Fructose-bisphosphate aldolase class II (EC 4.1.2.13)
contig_18	fig|6666666.28487.peg.1014	Protein	58716	58408	-	hypothetical protein
contig_18	fig|6666666.28487.peg.1015	Protein	59099	58932	-	hypothetical protein
contig_18	fig|6666666.28487.peg.1016	Protein	59338	62166	+	hypothetical protein
contig_18	fig|6666666.28487.peg.1017	Protein	62312	63079	+	hypothetical protein
contig_18	fig|6666666.28487.peg.1018	Protein	63615	63085	-	hypothetical protein
contig_18	fig|6666666.28487.peg.1019	Protein	64776	63763	-	Alcohol dehydrogenase (EC 1.1.1.1)
contig_18	fig|6666666.28487.peg.1020	Protein	64810	65520	+	DedA protein
contig_18	fig|6666666.28487.peg.1021	Protein	65636	65517	-	hypothetical protein
contig_18	fig|6666666.28487.peg.1022	Protein	65657	66142	+	FIG00982399: hypothetical protein

contig_18	fig|6666666.28487.peg.1023	Protein	67978	66131	-	Na+/H+ antiporter NhaA type
contig_18	fig|6666666.28487.peg.1024	Protein	69111	67975	-	fructose-bisphosphate aldolase family protein
contig_18	fig|6666666.28487.peg.1025	Protein	69185	69724	+	Transcriptional regulator PqrA, TetR family
contig_18	fig|6666666.28487.peg.1026	Protein	69721	71361	+	Multidrug resistance efflux pump
contig_18	fig|6666666.28487.peg.1027	Protein	72785	71886	-	hypothetical protein Rv0381c
contig_18	fig|6666666.28487.peg.1028	Protein	73568	72792	-	Probable short-chain dehydrogenase
contig_18	fig|6666666.28487.peg.1029	Protein	74386	73583	-	Possible membrane protein
contig_18	fig|6666666.28487.peg.1030	Protein	74496	75170	+	Short-chain dehydrogenase/reductase SDR
contig_18	fig|6666666.28487.peg.1031	Protein	77862	75316	-	ClpB protein
contig_18	fig|6666666.28487.peg.1032	Protein	78042	78659	+	FIG00832988: hypothetical protein
contig_18	fig|6666666.28487.peg.1033	Protein	79111	78656	-	Membrane spanning protein, GtrA-family
contig_18	fig|6666666.28487.peg.1034	Protein	79082	79222	+	hypothetical protein
contig_18	fig|6666666.28487.peg.1035	Protein	79541	79188	-	Glyoxalase family protein
contig_18	fig|6666666.28487.peg.1036	Protein	80678	79602	-	regulatory protein
contig_18	fig|6666666.28487.peg.1037	Protein	81234	80803	-	Transcriptional regulator, MarR family
contig_18	fig|6666666.28487.peg.1038	Protein	81828	81307	-	hypothetical protein
contig_18	fig|6666666.28487.peg.1039	Protein	82215	81838	-	hypothetical protein
contig_18	fig|6666666.28487.peg.1040	Protein	82814	82212	-	Cobalt-containing nitrile hydratase subunit alpha (EC 4.2.1.84)
contig_18	fig|6666666.28487.peg.1041	Protein	83521	82823	-	Cobalt-containing nitrile hydratase subunit beta (EC 4.2.1.84)

contig_18	fig|6666666.28487.peg.1042	Protein	83736	83948	+	hypothetical protein
contig_18	fig|6666666.28487.peg.1043	Protein	83969	84778	+	Predicted cobalt transporter CbtA
contig_18	fig|6666666.28487.peg.1044	Protein	84765	85778	+	Acetamidase/Formamidase
contig_18	fig|6666666.28487.peg.1045	Protein	86716	85766	-	Uncharacterized protein Synpcc7942_2318 (ORF 2)
contig_18	fig|6666666.28487.peg.1046	Protein	86783	87460	+	membrane protein
contig_18	fig|6666666.28487.peg.1047	Protein	88021	87449	-	Transcriptional regulator, TetR family
contig_18	fig|6666666.28487.peg.1048	Protein	88173	89183	+	putative membrane protein
contig_18	fig|6666666.28487.peg.1049	Protein	89195	90361	+	oxidoreductase, putative
contig_18	fig|6666666.28487.peg.1050	Protein	90386	91288	+	Aldose 1-epimerase
contig_18	fig|6666666.28487.peg.1051	Protein	92610	91357	-	hypothetical protein
contig_18	fig|6666666.28487.peg.1052	Protein	93112	92744	-	HspR, transcriptional repressor of DnaK operon
contig_18	fig|6666666.28487.peg.1053	Protein	94293	93109	-	Chaperone protein DnaJ
contig_18	fig|6666666.28487.peg.1054	Protein	94961	94335	-	Heat shock protein GrpE
contig_18	fig|6666666.28487.peg.1055	Protein	96838	94958	-	Chaperone protein DnaK
contig_18	fig|6666666.28487.peg.1056	Protein	97036	97998	+	Allantoicase (EC 3.5.3.4)
contig_18	fig|6666666.28487.peg.1057	Protein	97995	98900	+	Chitooligosaccharide deacetylase (EC 3.5.1.-); putative uricase
contig_18	fig|6666666.28487.peg.1058	Protein	99625	98885	-	Hydantoin racemase (EC 5.1.99.-)
contig_18	fig|6666666.28487.peg.1059	Protein	101175	99622	-	Hydantoin permease
contig_18	fig|6666666.28487.peg.1060	Protein	101948	101229	-	Transcriptional regulator, GntR family

contig_18	fig|6666666.28487.peg.1061	Protein	102139	102666	+	Lipoprotein LpqJ
contig_18	fig|6666666.28487.peg.1062	Protein	102734	104110	+	NADPH-dependent mycothiol reductase Mtr
contig_18	fig|6666666.28487.peg.1063	Protein	104107	105510	+	Glucose-6-phosphate 1-dehydrogenase (EC 1.1.1.49)
contig_18	fig|6666666.28487.peg.1064	Protein	105503	107299	+	Glucoamylase (EC 3.2.1.3)
contig_18	fig|6666666.28487.peg.1065	Protein	107315	107938	+	FIG00821383: hypothetical protein
contig_18	fig|6666666.28487.peg.1066	Protein	107956	108906	+	FIG00822435: hypothetical protein
contig_18	fig|6666666.28487.peg.1067	Protein	108947	109396	+	FIG01267895: hypothetical protein
contig_18	fig|6666666.28487.peg.1068	Protein	109423	109929	+	Glycoprotein gp2
contig_18	fig|6666666.28487.peg.1069	Protein	111727	109943	-	hypothetical protein
contig_18	fig|6666666.28487.peg.1070	Protein	113249	111765	-	Isoniazid inductible protein IniC
contig_18	fig|6666666.28487.peg.1071	Protein	115241	113349	-	conserved hypothetical proline rich protein
contig_18	fig|6666666.28487.peg.1072	Protein	117342	115495	-	Isoniazid inductible protein IniA
contig_18	fig|6666666.28487.peg.1073	Protein	118531	117434	-	Flagellar hook-length control protein FliK
contig_18	fig|6666666.28487.peg.1074	Protein	119374	118859	-	FIG00820657: hypothetical protein
contig_18	fig|6666666.28487.peg.1075	Protein	119582	121378	+	FIG00821990: hypothetical protein
contig_18	fig|6666666.28487.peg.1076	Protein	121389	123884	+	POSSIBLE TRANSCRIPTIONAL REGULATORY PROTEIN
contig_18	fig|6666666.28487.peg.1077	Protein	124039	127227	+	Iron-sulphur-binding reductase
contig_18	fig|6666666.28487.peg.1078	Protein	127243	128247	+	hypothetical protein
contig_18	fig|6666666.28487.peg.1079	Protein	128302	129570	+	Aspartate aminotransferase (EC 2.6.1.1)

contig_18	fig|6666666.28487.peg.1080	Protein	130159	129554	-	hypothetical protein
contig_18	fig|6666666.28487.peg.1081	Protein	130367	130254	-	hypothetical protein
contig_18	fig|6666666.28487.peg.1082	Protein	130380	131609	+	FIG00821472: hypothetical protein
contig_18	fig|6666666.28487.peg.1083	Protein	132504	131629	-	Glucose-1-phosphate thymidylyltransferase (EC 2.7.7.24)
contig_18	fig|6666666.28487.peg.1084	Protein	132872	132504	-	FIG00821425: hypothetical protein
contig_18	fig|6666666.28487.peg.1085	Protein	133628	132882	-	FIG00822836: hypothetical protein
contig_18	fig|6666666.28487.peg.1086	Protein	134929	133631	-	putative cytochrome P450 hydroxylase
contig_18	fig|6666666.28487.peg.1087	Protein	135288	134965	-	FIG00827470: hypothetical protein
contig_18	fig|6666666.28487.peg.1088	Protein	136638	135298	-	UDP-glucose dehydrogenase (EC 1.1.1.22)
contig_18	fig|6666666.28487.peg.1089	Protein	138332	136716	-	FIG00824053: hypothetical protein
contig_18	fig|6666666.28487.peg.1090	Protein	139134	138562	-	Deoxycytidine triphosphate deaminase (EC 3.5.4.13)
contig_18	fig|6666666.28487.peg.1091	Protein	140249	139155	-	hypothetical protein
contig_18	fig|6666666.28487.rna.8	RNA	140387	140457	+	tRNA-Gly-CCC
contig_18	fig|6666666.28487.peg.1092	Protein	141476	140529	-	site-specific recombinase, phage integrase family protein
contig_18	fig|6666666.28487.peg.1093	Protein	141921	141712	-	hypothetical protein
contig_18	fig|6666666.28487.peg.1094	Protein	141937	142098	+	hypothetical protein
contig_18	fig|6666666.28487.peg.1095	Protein	142423	142590	+	hypothetical protein
contig_18	fig|6666666.28487.peg.1096	Protein	142760	143053	+	hypothetical protein
contig_18	fig|6666666.28487.peg.1097	Protein	143053	143238	+	hypothetical protein

contig_18	fig|6666666.28487.peg.1098	Protein	143235	143615	+	hypothetical protein
contig_18	fig|6666666.28487.peg.1099	Protein	143837	144391	+	hypothetical protein
contig_18	fig|6666666.28487.peg.1100	Protein	144388	146058	+	hypothetical protein
contig_18	fig|6666666.28487.peg.1101	Protein	146055	146318	+	hypothetical protein
contig_18	fig|6666666.28487.peg.1102	Protein	146311	146631	+	hypothetical protein
contig_18	fig|6666666.28487.peg.1103	Protein	146628	147089	+	hypothetical protein
contig_18	fig|6666666.28487.peg.1104	Protein	147204	147091	-	hypothetical protein
contig_18	fig|6666666.28487.peg.1105	Protein	147231	148070	+	hypothetical protein
contig_18	fig|6666666.28487.peg.1106	Protein	148070	148255	+	hypothetical protein
contig_18	fig|6666666.28487.peg.1107	Protein	149100	149396	+	hypothetical protein
contig_18	fig|6666666.28487.peg.1108	Protein	149471	149797	+	hypothetical protein
contig_18	fig|6666666.28487.peg.1109	Protein	150098	151093	+	esterase, PHB depolymerase family
contig_18	fig|6666666.28487.peg.1110	Protein	151157	151393	+	hypothetical protein
contig_18	fig|6666666.28487.peg.1111	Protein	152037	151462	-	Transcriptional regulator, TetR family
contig_18	fig|6666666.28487.peg.1112	Protein	152543	152295	-	hypothetical protein
contig_18	fig|6666666.28487.peg.1113	Protein	153943	152732	-	putative cytochrome P450 hydroxylase
contig_18	fig|6666666.28487.peg.1114	Protein	154503	153940	-	transcriptional regulator, TetR family
contig_18	fig|6666666.28487.peg.1115	Protein	156066	154669	-	Low-affinity gluconate/H+ symporter GntU
contig_18	fig|6666666.28487.peg.1116	Protein	157060	156098	-	3-hydroxyacyl-CoA dehydrogenase (EC 1.1.1.35)

contig_18	fig|6666666.28487.peg.1117	Protein	158008	157085	-	AP endonuclease, family protein 2
contig_18	fig|6666666.28487.peg.1118	Protein	158077	158922	+	Transcriptional regulator, GntR family
contig_18	fig|6666666.28487.peg.1119	Protein	159010	160725	+	Dihydroxyacetone kinase, ATP-dependent (EC 2.7.1.29)
contig_18	fig|6666666.28487.peg.1120	Protein	160748	161206	+	Ribose 5-phosphate isomerase B (EC 5.3.1.6)
contig_18	fig|6666666.28487.peg.1121	Protein	161193	161999	+	Triosephosphate isomerase (EC 5.3.1.1)
contig_18	fig|6666666.28487.peg.1122	Protein	162221	162511	+	hypothetical protein
contig_18	fig|6666666.28487.peg.1123	Protein	162517	163236	+	hypothetical protein
contig_18	fig|6666666.28487.peg.1124	Protein	163223	163750	+	hypothetical protein
contig_18	fig|6666666.28487.peg.1125	Protein	163830	165056	+	FIG00831158: hypothetical protein
contig_18	fig|6666666.28487.peg.1126	Protein	166057	167859	+	hypothetical protein
contig_18	fig|6666666.28487.peg.1127	Protein	168858	167875	-	glycoside hydrolase, family 16
contig_18	fig|6666666.28487.peg.1128	Protein	168982	169767	+	Endonuclease/exonuclease/phosphatase
contig_19	fig|6666666.28487.peg.1129	Protein	430	14	-	PROBABLE CONSERVED TRANSMEMBRANE PROTEIN
contig_19	fig|6666666.28487.peg.1130	Protein	457	903	+	FIG00824284: hypothetical protein
contig_19	fig|6666666.28487.peg.1131	Protein	1639	893	-	FIG172214: hypothetical protein
contig_19	fig|6666666.28487.peg.1132	Protein	1865	2797	+	Cyclopropane-fatty-acyl-phospholipid synthase 2, CmaA2 (EC 2.1.1.79)
contig_19	fig|6666666.28487.peg.1133	Protein	2871	3755	+	METHOXY MYCOLIC ACID SYNTHASE 3 MMAA3 (METHYL MYCOLIC ACID SYNTHASE 3) (MMA3) (HYDROXY MYCOLIC ACID SYNTHASE)
contig_19	fig|6666666.28487.peg.1134	Protein	4980	3763	-	3-oxoacyl-[acyl-carrier-protein] synthase, KASII (EC 2.3.1.41)

contig_19	fig|6666666.28487.peg.1135	Protein	5115	5330	+	FIG234577: hypothetical protein
contig_19	fig|6666666.28487.peg.1136	Protein	6867	5443	-	Aldehyde dehydrogenase (EC 1.2.1.3)
contig_19	fig|6666666.28487.peg.1137	Protein	8608	6896	-	COG0028: Thiamine pyrophosphate-requiring enzymes
contig_19	fig|6666666.28487.peg.1138	Protein	9585	8620	-	Acyl-CoA dehydrogenase, short-chain specific (EC 1.3.99.2)
contig_19	fig|6666666.28487.peg.1139	Protein	10778	9582	-	FadE30
contig_19	fig|6666666.28487.peg.1140	Protein	11692	10775	-	acyl-CoA dehydrogenase, putative
contig_19	fig|6666666.28487.peg.1141	Protein	12840	11689	-	Acyl-CoA dehydrogenase (EC 1.3.99.-)
contig_19	fig|6666666.28487.peg.1142	Protein	13997	12840	-	L-carnitine dehydratase/bile acid-inducible protein F (EC 2.8.3.16)
contig_19	fig|6666666.28487.peg.1143	Protein	14961	13990	-	Short-chain dehydrogenase/reductase SDR
contig_19	fig|6666666.28487.peg.1144	Protein	16889	15039	-	Cyclohexanone monooxygenase (EC 1.14.13.22)
contig_19	fig|6666666.28487.peg.1145	Protein	18369	16957	-	Phenylpropionate dioxygenase and related ring-hydroxylating dioxygenases, large terminal subunit
contig_19	fig|6666666.28487.peg.1146	Protein	19220	18369	-	3-oxoacyl-[acyl-carrier protein] reductase (EC 1.1.1.100)
contig_19	fig|6666666.28487.peg.1147	Protein	19507	20967	+	FIG00821196: hypothetical protein
contig_19	fig|6666666.28487.peg.1148	Protein	20964	21707	+	Transcriptional regulator, GntR family
contig_19	fig|6666666.28487.peg.1149	Protein	21704	23194	+	Long-chain-fatty-acid--CoA ligase (EC 6.2.1.3)
contig_19	fig|6666666.28487.peg.1150	Protein	23196	23963	+	Oxidoreductase, short-chain dehydrogenase/reductase family
contig_19	fig|6666666.28487.peg.1151	Protein	24067	25557	+	Long-chain-fatty-acid--CoA ligase (EC 6.2.1.3)
contig_19	fig|6666666.28487.peg.1152	Protein	25582	27396	+	hypothetical protein

contig_19	fig|6666666.28487.peg.1153	Protein	28561	27404	-	putative integral membrane protein
contig_19	fig|6666666.28487.peg.1154	Protein	29402	28650	-	hypothetical protein
contig_19	fig|6666666.28487.peg.1155	Protein	30856	29399	-	hypothetical protein
contig_19	fig|6666666.28487.peg.1156	Protein	31020	31379	+	conserved hypothetical secreted protein
contig_19	fig|6666666.28487.peg.1157	Protein	32604	31543	-	FIG00821560: hypothetical protein
contig_19	fig|6666666.28487.peg.1158	Protein	32783	32565	-	hypothetical protein
contig_19	fig|6666666.28487.peg.1159	Protein	33065	33712	+	FIG00820766: hypothetical protein
contig_19	fig|6666666.28487.peg.1160	Protein	33747	34349	+	Transcriptional regulator, TetR family
contig_19	fig|6666666.28487.peg.1161	Protein	34651	34406	-	hypothetical protein
contig_19	fig|6666666.28487.peg.1162	Protein	34664	36031	+	MorD
contig_19	fig|6666666.28487.peg.1163	Protein	36028	36834	+	ATPase
contig_19	fig|6666666.28487.peg.1164	Protein	36850	36981	+	FIG00820854: hypothetical protein
contig_19	fig|6666666.28487.peg.1165	Protein	36978	37703	+	FIG00822652: hypothetical protein
contig_19	fig|6666666.28487.peg.1166	Protein	37719	38135	+	FIG00822010: hypothetical protein
contig_19	fig|6666666.28487.peg.1167	Protein	38132	39352	+	putative cytochrome P450 hydroxylase
contig_19	fig|6666666.28487.peg.1168	Protein	39408	40445	+	FIG00825548: hypothetical protein
contig_19	fig|6666666.28487.peg.1169	Protein	40554	41117	+	Sigma 1-type opioid receptor
contig_19	fig|6666666.28487.peg.1170	Protein	41123	41785	+	oxidoreductase ylbE
contig_19	fig|6666666.28487.peg.1171	Protein	42298	41867	-	FIG00820677: hypothetical protein

contig_19	fig|6666666.28487.peg.1172	Protein	42607	42302	-	FIG01278058: hypothetical protein
contig_19	fig|6666666.28487.peg.1173	Protein	45005	42618	-	Cation-transporting ATPase, E1-E2 family
contig_19	fig|6666666.28487.peg.1174	Protein	46597	45002	-	Beta-lactamase (EC 3.5.2.6)
contig_19	fig|6666666.28487.peg.1175	Protein	47747	46626	-	Outer membrane protein romA
contig_19	fig|6666666.28487.peg.1176	Protein	48469	47744	-	Enoyl-CoA hydratase (EC 4.2.1.17)
contig_19	fig|6666666.28487.peg.1177	Protein	48505	49992	+	Acetyl-coenzyme A carboxyl transferase alpha chain (EC 6.4.1.2) / Acetyl-coenzyme A carboxyl transferase beta chain (EC 6.4.1.2)
contig_19	fig|6666666.28487.peg.1178	Protein	50060	52825	+	hypothetical protein
contig_19	fig|6666666.28487.peg.1179	Protein	53268	52822	-	Transposase and inactivated derivatives
contig_19	fig|6666666.28487.peg.1180	Protein	53301	54404	+	Epoxide hydrolase (EC 3.3.2.9)
contig_19	fig|6666666.28487.peg.1181	Protein	56889	54412	-	hypothetical protein
contig_19	fig|6666666.28487.peg.1182	Protein	58863	57046	-	ABC-type multidrug/protein/lipid transport system, ATPase component
contig_19	fig|6666666.28487.peg.1183	Protein	60706	58892	-	ABC transporter, ATP-binding/permease protein
contig_19	fig|6666666.28487.peg.1184	Protein	62732	60789	-	ABC transporter, ATP-binding/permease protein
contig_19	fig|6666666.28487.peg.1185	Protein	63352	62804	-	Chromate transporter
contig_19	fig|6666666.28487.peg.1186	Protein	63932	63357	-	chromate transport protein
contig_19	fig|6666666.28487.peg.1187	Protein	64905	63925	-	Cardiolipin synthetase (EC 2.7.8.-)
contig_19	fig|6666666.28487.peg.1188	Protein	64894	65025	+	hypothetical protein
contig_19	fig|6666666.28487.peg.1189	Protein	65090	65791	+	DNA-binding response regulator

contig_19	fig|6666666.28487.peg.1190	Protein	65804	67147	+	Sensor-type histidine kinase prrB (EC 2.7.13.3)
contig_19	fig|6666666.28487.peg.1191	Protein	67578	67216	-	FKBP-type peptidyl-prolyl cis-trans isomerase
contig_19	fig|6666666.28487.peg.1192	Protein	67631	67879	+	FIG00821893: hypothetical protein
contig_19	fig|6666666.28487.peg.1193	Protein	68521	67967	-	hypothetical protein
contig_19	fig|6666666.28487.peg.1194	Protein	69271	68558	-	hypothetical protein
contig_19	fig|6666666.28487.peg.1195	Protein	69974	69318	-	hypothetical protein
contig_19	fig|6666666.28487.peg.1196	Protein	70230	70739	+	hypothetical protein
contig_19	fig|6666666.28487.peg.1197	Protein	70732	72024	+	Beta-carotene ketolase (EC 1.14.-.-)
contig_19	fig|6666666.28487.peg.1198	Protein	72091	72291	+	Beta-carotene ketolase (EC 1.14.-.-)
contig_19	fig|6666666.28487.peg.1199	Protein	72732	72313	-	Transcriptional regulator, MarR family
contig_19	fig|6666666.28487.peg.1200	Protein	74170	72722	-	PROBABLE CONSERVED INTEGRAL MEMBRANE TRANSPORT PROTEIN
contig_19	fig|6666666.28487.peg.1201	Protein	75645	74344	-	Citrate synthase (si) (EC 2.3.3.1)
contig_19	fig|6666666.28487.peg.1202	Protein	76400	75756	-	Transcriptional regulator, TetR family
contig_19	fig|6666666.28487.peg.1203	Protein	77168	76545	-	Pyridoxamine 5'-phosphate oxidase (EC 1.4.3.5)
contig_19	fig|6666666.28487.peg.1204	Protein	77255	78364	+	Citrate synthase (si) (EC 2.3.3.1)
contig_19	fig|6666666.28487.peg.1205	Protein	78372	78986	+	FIG00822982: hypothetical protein
contig_19	fig|6666666.28487.peg.1206	Protein	80703	79000	-	Undecaprenyl-phosphate galactosephosphotransferase (EC 2.7.8.6)
contig_19	fig|6666666.28487.peg.1207	Protein	80965	81417	+	PhnB protein; putative DNA binding 3-demethylubiquinone-9 3-methyltransferase domain protein

contig_19	fig|6666666.28487.peg.1208	Protein	81741	82517	+	hypothetical protein
contig_19	fig|6666666.28487.peg.1209	Protein	84181	82520	-	Ferredoxin / Ferredoxin--NADP(+) reductase, actinobacterial (eukaryote-like) type (EC 1.18.1.2)
contig_19	fig|6666666.28487.peg.1210	Protein	85218	84190	-	possible transmembrane protein
contig_19	fig|6666666.28487.peg.1211	Protein	85308	86528	+	Phosphoserine aminotransferase (EC 2.6.1.52)
contig_19	fig|6666666.28487.peg.1212	Protein	86618	87409	+	FIG00996668: hypothetical protein
contig_19	fig|6666666.28487.peg.1213	Protein	87716	87441	-	PROBABLE TRANSMEMBRANE PROTEIN
contig_19	fig|6666666.28487.peg.1214	Protein	88552	87713	-	putative rRNA methylase
contig_19	fig|6666666.28487.peg.1215	Protein	88633	88893	+	POSSIBLE CONSERVED TRANSMEMBRANE PROTEIN
contig_19	fig|6666666.28487.peg.1216	Protein	88903	89355	+	FIG00820282: hypothetical protein
contig_19	fig|6666666.28487.peg.1217	Protein	90188	89352	-	FIG00821400: hypothetical protein
contig_19	fig|6666666.28487.peg.1218	Protein	90361	91950	+	possible membrane protein
contig_19	fig|6666666.28487.peg.1219	Protein	91947	92462	+	POSSIBLE CONSERVED EXPORTED PROTEIN
contig_19	fig|6666666.28487.peg.1220	Protein	93464	92451	-	FIG00820534: hypothetical protein
contig_19	fig|6666666.28487.peg.1221	Protein	93985	93575	-	Cold shock protein CspC
contig_19	fig|6666666.28487.peg.1222	Protein	94135	94542	+	FIG020413: transmembrane protein
contig_19	fig|6666666.28487.peg.1223	Protein	94608	95600	+	Molybdenum cofactor biosynthesis protein MoaA
contig_19	fig|6666666.28487.peg.1224	Protein	95604	95870	+	Molybdenum cofactor biosynthesis protein MoaD
contig_19	fig|6666666.28487.peg.1225	Protein	96340	97782	+	FIG00820555: hypothetical protein
contig_19	fig|6666666.28487.peg.1226	Protein	98215	97790	-	Molybdenum cofactor biosynthesis protein MoaE

contig_19	fig|6666666.28487.peg.1227	Protein	98691	98212	-	Molybdenum cofactor biosynthesis protein MoaB
contig_19	fig|6666666.28487.peg.1228	Protein	99170	98688	-	Molybdenum cofactor biosynthesis protein MoaC
contig_19	fig|6666666.28487.peg.1229	Protein	99358	99173	-	FIG057355: hypothetical protein
contig_19	fig|6666666.28487.peg.1230	Protein	99451	101697	+	probable DNA-binding protein
contig_19	fig|6666666.28487.peg.1231	Protein	104643	101704	-	FIG00830370: hypothetical protein
contig_19	fig|6666666.28487.peg.1232	Protein	105168	106796	+	DNA repair helicase
contig_19	fig|6666666.28487.peg.1233	Protein	108884	106803	-	hypothetical protein
contig_2	fig|6666666.28487.peg.1234	Protein	877	329	-	hypothetical protein
contig_2	fig|6666666.28487.peg.1235	Protein	1052	1690	+	Mobile element protein
contig_2	fig|6666666.28487.peg.1236	Protein	1687	2286	+	Mobile element protein
contig_2	fig|6666666.28487.peg.1237	Protein	5284	2675	-	Replicative DNA helicase (EC 3.6.1.-) @ intein-containing
contig_2	fig|6666666.28487.peg.1238	Protein	6226	5777	-	LSU ribosomal protein L9p
contig_2	fig|6666666.28487.peg.1239	Protein	6495	6238	-	SSU ribosomal protein S18p @ SSU ribosomal protein S18p, zinc-dependent
contig_2	fig|6666666.28487.peg.1240	Protein	7045	6536	-	Single-stranded DNA-binding protein
contig_2	fig|6666666.28487.peg.1241	Protein	7435	7163	-	SSU ribosomal protein S6p
contig_2	fig|6666666.28487.peg.1242	Protein	7630	8160	+	hypothetical protein
contig_2	fig|6666666.28487.peg.1243	Protein	9676	8123	-	Multimodular transpeptidase-transglycosylase (EC 2.4.1.129) (EC 3.4.-.-)
contig_2	fig|6666666.28487.peg.1244	Protein	12177	9748	-	Multimodular transpeptidase-transglycosylase (EC 2.4.1.129) (EC 3.4.-.-)

contig_2	fig|6666666.28487.peg.1245	Protein	12736	12302	-	FIG00822203: hypothetical protein
contig_2	fig|6666666.28487.peg.1246	Protein	12871	13746	+	Possible membrane protein
contig_2	fig|6666666.28487.peg.1247	Protein	13865	14410	+	Transcriptional regulator, PadR family
contig_2	fig|6666666.28487.peg.1248	Protein	14481	15569	+	Inositol-1-phosphate synthase (EC 5.5.1.4)
contig_2	fig|6666666.28487.peg.1249	Protein	15728	16624	+	Hydrolase
contig_2	fig|6666666.28487.peg.1250	Protein	18586	16799	-	FIG00825201: hypothetical protein
contig_2	fig|6666666.28487.peg.1251	Protein	18813	20888	+	Putative phosphatase
contig_2	fig|6666666.28487.peg.1252	Protein	20973	21770	+	Possible oxidoreductase
contig_2	fig|6666666.28487.peg.1253	Protein	21823	22500	+	Transcriptional regulator, GntR family
contig_2	fig|6666666.28487.peg.1254	Protein	22510	24297	+	Amino acid ABC transporter, permease protein, 3-TM region, His/Glu/Gln/Arg/opine family protein
contig_2	fig|6666666.28487.peg.1255	Protein	24297	25028	+	Amino acid ABC transporter, ATP-binding protein
contig_2	fig|6666666.28487.peg.1256	Protein	25113	25550	+	Transcriptional regulator, MarR family
contig_2	fig|6666666.28487.peg.1257	Protein	26894	25554	-	Putative isomerase
contig_2	fig|6666666.28487.peg.1258	Protein	26951	27616	+	Short chain dehydrogenase
contig_2	fig|6666666.28487.peg.1259	Protein	28514	27657	-	hypothetical protein
contig_2	fig|6666666.28487.peg.1260	Protein	31300	28616	-	Leucyl-tRNA synthetase (EC 6.1.1.4)
contig_20	fig|6666666.28487.peg.1261	Protein	57	1100	+	Cytochrome P450
contig_20	fig|6666666.28487.peg.1262	Protein	1097	1750	+	Transglutaminase-like domain
contig_20	fig|6666666.28487.peg.1263	Protein	1777	3396	+	Long-chain-fatty-acid--CoA ligase (EC 6.2.1.3)

contig_20	fig|6666666.28487.peg.1264	Protein	3435	4580	+	Butyryl-CoA dehydrogenase (EC 1.3.99.2)
contig_20	fig|6666666.28487.peg.1265	Protein	4630	5688	+	FIG00826404: hypothetical protein
contig_20	fig|6666666.28487.peg.1266	Protein	5780	6190	+	FIG00822284: hypothetical protein
contig_20	fig|6666666.28487.peg.1267	Protein	6339	7445	+	Alcohol dehydrogenase (EC 1.1.1.1)
contig_20	fig|6666666.28487.peg.1268	Protein	7765	8805	+	Isovaleryl-CoA dehydrogenase (EC 1.3.99.10)
contig_20	fig|6666666.28487.peg.1269	Protein	9286	8846	-	Mobile element protein
contig_20	fig|6666666.28487.peg.1270	Protein	9358	9666	+	Mobile element protein
contig_20	fig|6666666.28487.peg.1271	Protein	9699	10520	+	Mobile element protein
contig_20	fig|6666666.28487.peg.1272	Protein	11276	12046	+	FIG00828501: hypothetical protein
contig_20	fig|6666666.28487.peg.1273	Protein	12043	12786	+	FIG00824390: hypothetical protein
contig_20	fig|6666666.28487.peg.1274	Protein	12964	14079	+	Patatin-like protein
contig_20	fig|6666666.28487.peg.1275	Protein	14066	15079	+	hypothetical protein
contig_20	fig|6666666.28487.peg.1276	Protein	15076	16851	+	Molybdopterin biosynthesis MoeB protein
contig_20	fig|6666666.28487.peg.1277	Protein	16938	17090	+	hypothetical protein
contig_20	fig|6666666.28487.peg.1278	Protein	17452	17976	+	FIG00821288: hypothetical protein
contig_20	fig|6666666.28487.peg.1279	Protein	18452	17985	-	MutT-like protein
contig_20	fig|6666666.28487.peg.1280	Protein	19259	18459	-	FIG00827398: hypothetical protein
contig_20	fig|6666666.28487.peg.1281	Protein	19513	19857	+	FIG00821607: hypothetical protein
contig_20	fig|6666666.28487.peg.1282	Protein	19950	21395	+	Transfer protein traSA

contig_20	fig|6666666.28487.peg.1283	Protein	21457	22899	+	Replication initiator protein
contig_20	fig|6666666.28487.peg.1284	Protein	23089	24735	+	Site-specific recombinase
contig_20	fig|6666666.28487.rna.9	RNA	24802	24717	-	tRNA-Leu-GAG
contig_20	fig|6666666.28487.peg.1285	Protein	24881	26215	+	FIG016551: Putative peptidase
contig_20	fig|6666666.28487.peg.1286	Protein	26239	26775	+	Phospholipid-binding protein
contig_20	fig|6666666.28487.peg.1287	Protein	28093	26750	-	FIG00870036: hypothetical protein
contig_20	fig|6666666.28487.peg.1288	Protein	29172	28090	-	oxidoreductase
contig_20	fig|6666666.28487.peg.1289	Protein	29245	29841	+	hypothetical protein
contig_20	fig|6666666.28487.peg.1290	Protein	30902	29838	-	Dihydroorotate dehydrogenase (EC 1.3.3.1)
contig_20	fig|6666666.28487.peg.1291	Protein	31138	30899	-	FIG00821060: hypothetical protein
contig_20	fig|6666666.28487.peg.1292	Protein	32127	31153	-	Lipoprotein LppL
contig_20	fig|6666666.28487.peg.1293	Protein	32313	32624	+	FIG00821698: hypothetical protein
contig_20	fig|6666666.28487.peg.1294	Protein	32661	33503	+	Undecaprenyl-diphosphatase (EC 3.6.1.27)
contig_20	fig|6666666.28487.peg.1295	Protein	33500	34204	+	Phosphoglycerate mutase (EC 5.4.2.1)
contig_20	fig|6666666.28487.peg.1296	Protein	34242	34826	+	FIG00820819: hypothetical protein
contig_20	fig|6666666.28487.peg.1297	Protein	34852	35619	+	Phosphatidylinositol 3-and 4-kinase family protein
contig_20	fig|6666666.28487.peg.1298	Protein	35604	36365	+	Protein cysQ homolog
contig_20	fig|6666666.28487.peg.1299	Protein	36408	37646	+	L-cysteine:1D-myo-inosityl 2-amino-2-deoxy-alpha-D-glucopyranoside ligase MshC
contig_20	fig|6666666.28487.peg.1300	Protein	37674	39155	+	putative exported protein

contig_20	fig|6666666.28487.peg.1301	Protein	40030	39152	-	FIG00823557: hypothetical protein
contig_20	fig|6666666.28487.peg.1302	Protein	40343	44137	+	5-methyltetrahydrofolate--homocysteine methyltransferase (EC 2.1.1.13)
contig_20	fig|6666666.28487.peg.1303	Protein	44259	44921	+	Phosphoribosyl-ATP pyrophosphatase (EC 3.6.1.31)
contig_20	fig|6666666.28487.peg.1304	Protein	45025	46497	+	Sugar transporter
contig_20	fig|6666666.28487.peg.1305	Protein	46552	46815	+	Phosphoribosyl-ATP pyrophosphatase (EC 3.6.1.31)
contig_20	fig|6666666.28487.peg.1306	Protein	46817	47662	+	ATP phosphoribosyltransferase (EC 2.4.2.17)
contig_20	fig|6666666.28487.peg.1307	Protein	47731	48213	+	Hypothetical membrane protein Rv2120c
contig_20	fig|6666666.28487.peg.1308	Protein	48221	49591	+	PF00070 family, FAD-dependent NAD(P)-disulphide oxidoreductase
contig_20	fig|6666666.28487.peg.1309	Protein	49662	50348	+	Transcriptional regulator, TetR family
contig_20	fig|6666666.28487.peg.1310	Protein	50345	51277	+	NrdB
contig_20	fig|6666666.28487.peg.1311	Protein	51784	51281	-	FIG00820071: hypothetical protein
contig_20	fig|6666666.28487.peg.1312	Protein	52575	51784	-	TesB-like acyl-CoA thioesterase 5
contig_20	fig|6666666.28487.peg.1313	Protein	53435	52572	-	RecB family exonuclease
contig_20	fig|6666666.28487.peg.1314	Protein	53511	54341	+	RNA methyltransferase
contig_20	fig|6666666.28487.peg.1315	Protein	54634	54338	-	YlxP-like protein
contig_20	fig|6666666.28487.peg.1316	Protein	55148	54639	-	Probable conserved lipoprotein lppK
contig_20	fig|6666666.28487.peg.1317	Protein	55720	55217	-	Low molecular weight antigen MTB12 precursor
contig_20	fig|6666666.28487.peg.1318	Protein	55868	57715	+	Bacterial proteasome-activating AAA-ATPase (PAN)

contig_20	fig|6666666.28487.peg.1319	Protein	57843	58160	+	FIG00821667: hypothetical protein
contig_20	fig|6666666.28487.peg.1320	Protein	58763	58161	-	FIG00821521: hypothetical protein
contig_20	fig|6666666.28487.peg.1321	Protein	59932	58760	-	FIG00830267: hypothetical protein
contig_20	fig|6666666.28487.peg.1322	Protein	61080	59929	-	hypothetical protein
contig_20	fig|6666666.28487.peg.1323	Protein	61139	62647	+	Pup ligase PafA' paralog, possible component of postulated heterodimer PafA-PafA'
contig_20	fig|6666666.28487.peg.1324	Protein	62753	62947	+	Prokaryotic ubiquitin-like protein Pup
contig_20	fig|6666666.28487.peg.1325	Protein	62944	63852	+	Proteasome subunit beta (EC 3.4.25.1), bacterial
contig_20	fig|6666666.28487.peg.1326	Protein	63849	64607	+	Proteasome subunit alpha (EC 3.4.25.1), bacterial
contig_20	fig|6666666.28487.peg.1327	Protein	64852	67296	+	Putative Dimethylglycine oxidase
contig_20	fig|6666666.28487.peg.1328	Protein	67301	68437	+	sarcosine oxidase
contig_20	fig|6666666.28487.peg.1329	Protein	68434	69573	+	Phenylpropionate dioxygenase and related ring-hydroxylating dioxygenases, large terminal subunit
contig_20	fig|6666666.28487.peg.1330	Protein	69597	71099	+	Betaine aldehyde dehydrogenase (EC 1.2.1.8)
contig_20	fig|6666666.28487.peg.1331	Protein	71108	72667	+	Urea carboxylase-related amino acid permease
contig_20	fig|6666666.28487.peg.1332	Protein	72660	74180	+	Choline dehydrogenase (EC 1.1.99.1)
contig_20	fig|6666666.28487.peg.1333	Protein	74155	76002	+	Predicted signal-transduction protein containing cAMP-binding and CBS domains
contig_20	fig|6666666.28487.peg.1334	Protein	76015	76809	+	Transcriptional regulator, IclR family
contig_20	fig|6666666.28487.peg.1335	Protein	78488	76866	-	Probable secreted protein
contig_20	fig|6666666.28487.peg.1336	Protein	78745	78485	-	hypothetical protein

contig_20	fig|6666666.28487.peg.1337	Protein	78883	80226	+	Pup ligase PafA, possible component of postulated heterodimer PafA-PafA'
contig_20	fig|6666666.28487.peg.1338	Protein	80849	80253	-	Methyltransferase type 12
contig_20	fig|6666666.28487.peg.1339	Protein	80924	81934	+	FIG005453: Putative DeoR-family transcriptional regulator
contig_20	fig|6666666.28487.peg.1340	Protein	81931	82917	+	FIG019733: possible DNA-binding protein
contig_20	fig|6666666.28487.peg.1341	Protein	82948	83259	+	Twin-arginine translocation protein TatA
contig_20	fig|6666666.28487.peg.1342	Protein	83332	84282	+	Twin-arginine translocation protein TatC
contig_20	fig|6666666.28487.peg.1343	Protein	84266	87046	+	FIG005666: putative helicase
contig_20	fig|6666666.28487.peg.1344	Protein	87094	87858	+	Possible membrane protein
contig_20	fig|6666666.28487.peg.1345	Protein	88837	87872	-	DNA polymerase I (EC 2.7.7.7)
contig_20	fig|6666666.28487.peg.1346	Protein	88959	90089	+	Probable dipeptidase PepE (EC 3.4.13.-)
contig_20	fig|6666666.28487.peg.1347	Protein	90513	90100	-	FIG01000472: hypothetical protein
contig_20	fig|6666666.28487.peg.1348	Protein	90549	91304	+	Oxidoreductase, short-chain dehydrogenase/reductase family
contig_20	fig|6666666.28487.peg.1349	Protein	91301	92491	+	Cobalt-precorrin-6y C5-methyltransferase (EC 2.1.1.-) / Cobalt-precorrin-6y C15-methyltransferase [decarboxylating] (EC 2.1.1.-)
contig_20	fig|6666666.28487.peg.1350	Protein	93542	92454	-	FIG00823195: hypothetical protein
contig_20	fig|6666666.28487.peg.1351	Protein	94170	93553	-	Transcriptional regulator, TetR family
contig_20	fig|6666666.28487.peg.1352	Protein	95736	94249	-	Cobalt-precorrin-2 C20-methyltransferase (EC 2.1.1.130) / Cobalt-precorrin-3b C17-methyltransferase
contig_20	fig|6666666.28487.peg.1353	Protein	96359	95733	-	Cobalt-precorrin-8x methylmutase (EC 5.4.1.2)

contig_20	fig|6666666.28487.peg.1354	Protein	97487	96369	-	Cobalamin biosynthesis protein CobG
contig_20	fig|6666666.28487.peg.1355	Protein	97800	98102	+	hypothetical protein
contig_20	fig|6666666.28487.peg.1356	Protein	99028	98108	-	Alpha-ketoglutarate-dependent taurine dioxygenase (EC 1.14.11.17)
contig_20	fig|6666666.28487.peg.1357	Protein	99211	99858	+	Glycine betaine/carnitine/choline ABC transporter, permease protein
contig_20	fig|6666666.28487.peg.1358	Protein	99855	101054	+	L-proline glycine betaine ABC transport system permease protein ProV (TC 3.A.1.12.1)
contig_20	fig|6666666.28487.peg.1359	Protein	101051	101836	+	putative ABC transporter permease
contig_20	fig|6666666.28487.peg.1360	Protein	101833	102801	+	L-proline glycine betaine binding ABC transporter protein ProX (TC 3.A.1.12.1) / Osmotic adaptation
contig_20	fig|6666666.28487.peg.1361	Protein	103416	103039	-	hypothetical protein
contig_20	fig|6666666.28487.peg.1362	Protein	104287	103466	-	Transcriptional regulator, AraC family
contig_20	fig|6666666.28487.peg.1363	Protein	104316	107921	+	CobN component of cobalt chelatase involved in B12 biosynthesis
contig_20	fig|6666666.28487.peg.1364	Protein	107947	108324	+	FIG00995176: hypothetical protein
contig_20	fig|6666666.28487.peg.1365	Protein	108332	108739	+	FIG00820604: hypothetical protein
contig_20	fig|6666666.28487.peg.1366	Protein	108773	109282	+	Cytoplasmic membrane protein FsxA
contig_20	fig|6666666.28487.peg.1367	Protein	109279	110862	+	Exoenzymes regulatory protein AepA in lipid-linked oligosaccharide synthesis cluster
contig_20	fig|6666666.28487.peg.1368	Protein	110855	112528	+	Apolipoprotein N-acyltransferase (EC 2.3.1.-) in lipid-linked oligosaccharide synthesis cluster
contig_20	fig|6666666.28487.peg.1369	Protein	112525	113310	+	Dolichol-phosphate mannosyltransferase (EC 2.4.1.83) in

						lipid-linked oligosaccharide synthesis cluster
contig_20	fig|6666666.28487.peg.1370	Protein	113691	113395	-	FIG00820327: hypothetical protein
contig_20	fig|6666666.28487.peg.1371	Protein	113949	114230	+	FIG00822025: hypothetical protein
contig_20	fig|6666666.28487.peg.1372	Protein	114269	115498	+	Transcriptional regulator, CadC
contig_20	fig|6666666.28487.peg.1373	Protein	115710	115540	-	hypothetical protein
contig_20	fig|6666666.28487.peg.1374	Protein	115742	116290	+	FIG00827741: hypothetical protein
contig_20	fig|6666666.28487.peg.1375	Protein	116459	117040	+	FIG00822076: hypothetical protein
contig_20	fig|6666666.28487.peg.1376	Protein	117589	117047	-	Probable lipoprotein lppI
contig_20	fig|6666666.28487.peg.1377	Protein	117639	117755	+	hypothetical protein
contig_20	fig|6666666.28487.peg.1378	Protein	117837	119333	+	Probable carboxylesterase LipT (EC 3.1.1.-)
contig_20	fig|6666666.28487.peg.1379	Protein	119393	121396	+	Monoamine oxidase (1.4.3.4)
contig_20	fig|6666666.28487.peg.1380	Protein	121481	121807	+	Protein of unknown function UPF0060
contig_20	fig|6666666.28487.peg.1381	Protein	122695	121811	-	FIG00820237: hypothetical protein
contig_20	fig|6666666.28487.peg.1382	Protein	122772	123509	+	FIG00826599: hypothetical protein
contig_20	fig|6666666.28487.peg.1383	Protein	124509	123562	-	FIG00823227: hypothetical protein
contig_20	fig|6666666.28487.peg.1384	Protein	124535	124672	+	hypothetical protein
contig_20	fig|6666666.28487.peg.1385	Protein	125556	124642	-	transcriptional regulator, LysR family
contig_20	fig|6666666.28487.peg.1386	Protein	125605	126420	+	Enoyl-CoA hydratase (EC 4.2.1.17)
contig_20	fig|6666666.28487.peg.1387	Protein	126472	127407	+	Integral membrane protein

contig_20	fig|6666666.28487.peg.1388	Protein	127467	127787	+	FIG00828063: hypothetical protein
contig_20	fig|6666666.28487.peg.1389	Protein	128718	127861	-	Putrescine transport system permease protein PotI (TC 3.A.1.11.2)
contig_20	fig|6666666.28487.peg.1390	Protein	129590	128715	-	Putrescine transport system permease protein PotH (TC 3.A.1.11.2)
contig_20	fig|6666666.28487.peg.1391	Protein	130802	129621	-	Putrescine ABC transporter putrescine-binding protein PotF (TC 3.A.1.11.2)
contig_20	fig|6666666.28487.peg.1392	Protein	131988	130876	-	Putrescine transport ATP-binding protein PotG (TC 3.A.1.11.2)
contig_20	fig|6666666.28487.peg.1393	Protein	132353	132784	+	FIG00822670: hypothetical protein
contig_20	fig|6666666.28487.peg.1394	Protein	134231	132792	-	putative membrane transport protein
contig_20	fig|6666666.28487.peg.1395	Protein	135460	134288	-	D-AMINOPEPTIDASE (EC 3.4.11.19)
contig_20	fig|6666666.28487.peg.1396	Protein	135471	136907	+	Cytosine/purine/uracil/thiamine/allantoin permease family protein
contig_20	fig|6666666.28487.peg.1397	Protein	136969	137451	+	TspO-MBR family protein
contig_20	fig|6666666.28487.peg.1398	Protein	137463	137981	+	FIG00828264: hypothetical protein
contig_20	fig|6666666.28487.peg.1399	Protein	138596	138024	-	probable serine/threonine-protein kinase PknH, putative(EC:2.7.11.1)
contig_21	fig|6666666.28487.peg.1400	Protein	129	623	+	Tryptophan-rich sensory protein
contig_21	fig|6666666.28487.peg.1401	Protein	654	1439	+	oxidoreductase, short-chain dehydrogenase/reductase family
contig_21	fig|6666666.28487.peg.1402	Protein	2524	1436	-	FIG00833520: hypothetical protein
contig_21	fig|6666666.28487.peg.1403	Protein	2762	4597	+	3-methylmercaptopropionyl-CoA dehydrogenase (DmdC)

contig_21	fig|6666666.28487.peg.1404	Protein	5096	5740	+	hypothetical protein
contig_21	fig|6666666.28487.peg.1405	Protein	6005	5730	-	hypothetical protein
contig_21	fig|6666666.28487.peg.1406	Protein	7170	6244	-	FIG00830838: hypothetical protein
contig_21	fig|6666666.28487.peg.1407	Protein	8695	7394	-	3-ketoacyl-CoA thiolase (EC 2.3.1.16) @ Acetyl-CoA acetyltransferase (EC 2.3.1.9)
contig_21	fig|6666666.28487.peg.1408	Protein	8855	10207	+	3-oxoacyl-[acyl-carrier protein] reductase (EC 1.1.1.100)
contig_21	fig|6666666.28487.peg.1409	Protein	10209	11057	+	Acyl dehydratase
contig_21	fig|6666666.28487.peg.1410	Protein	11664	11044	-	Transcriptional regulator, TetR family
contig_21	fig|6666666.28487.peg.1411	Protein	12749	11751	-	Beta-hexosaminidase (EC 3.2.1.52)
contig_21	fig|6666666.28487.peg.1412	Protein	13167	13655	+	Universal stress protein family
contig_21	fig|6666666.28487.peg.1413	Protein	13728	13886	+	SMALL SECRETED PROTEIN
contig_21	fig|6666666.28487.peg.1414	Protein	14074	18111	+	PROBABLE CONSERVED TRANSMEMBRANE PROTEIN
contig_21	fig|6666666.28487.peg.1415	Protein	18104	19597	+	Long-chain-fatty-acid--CoA ligase (EC 6.2.1.3)
contig_21	fig|6666666.28487.peg.1416	Protein	20864	19686	-	Lysophospholipid acyltransferase
contig_21	fig|6666666.28487.peg.1417	Protein	21046	22251	+	PROBABLE CONSERVED MEMBRANE PROTEIN
contig_21	fig|6666666.28487.peg.1418	Protein	22308	24014	+	Possible membrane protein
contig_21	fig|6666666.28487.peg.1419	Protein	25087	24227	-	FIG00832597: hypothetical protein
contig_21	fig|6666666.28487.peg.1420	Protein	25293	25084	-	hypothetical protein
contig_21	fig|6666666.28487.peg.1421	Protein	25431	26660	+	putative cytochrome P450 hydroxylase
contig_21	fig|6666666.28487.peg.1422	Protein	27810	26635	-	Glycosyltransferase (EC 2.4.1.-)

contig_21	fig|6666666.28487.peg.1423	Protein	27898	28665	+	POSSIBLE METHYLTRANSFERASE (METHYLASE)
contig_21	fig|6666666.28487.peg.1424	Protein	28711	30195	+	Aldehyde dehydrogenase (EC 1.2.1.3)
contig_21	fig|6666666.28487.peg.1425	Protein	30661	30266	-	conserved hypothetical protein
contig_21	fig|6666666.28487.peg.1426	Protein	32542	30707	-	3-methylmercaptopropionyl-CoA dehydrogenase (DmdC)
contig_21	fig|6666666.28487.peg.1427	Protein	33131	32610	-	Bifunctional deaminase-reductase domain protein
contig_21	fig|6666666.28487.peg.1428	Protein	34069	33143	-	Transcriptional regulator, AraC family
contig_22	fig|6666666.28487.peg.1429	Protein	1111	668	-	Deoxyuridine 5'-triphosphate nucleotidohydrolase (EC 3.6.1.23)
contig_22	fig|6666666.28487.peg.1430	Protein	1158	1646	+	PROBABLE CONSERVED ALANINE RICH TRANSMEMBRANE PROTEIN
contig_22	fig|6666666.28487.peg.1431	Protein	1952	1650	-	hypothetical protein
contig_22	fig|6666666.28487.peg.1432	Protein	2121	2786	+	Possible secreted alanine rich protein
contig_22	fig|6666666.28487.peg.1433	Protein	3640	2789	-	Inositol-1-monophosphatase (EC 3.1.3.25)
contig_22	fig|6666666.28487.peg.1434	Protein	3806	4603	+	Polyphosphate glucokinase (EC 2.7.1.63)
contig_22	fig|6666666.28487.peg.1435	Protein	4824	6272	+	RNA polymerase sigma factor RpoD
contig_22	fig|6666666.28487.peg.1436	Protein	6351	7445	+	O-methyltransferase
contig_22	fig|6666666.28487.peg.1437	Protein	9142	7460	-	PE-PGRS family protein
contig_22	fig|6666666.28487.peg.1438	Protein	10032	9250	-	FIG00821682: hypothetical protein
contig_22	fig|6666666.28487.peg.1439	Protein	10473	10108	-	Glutathione S-transferase, omega (EC 2.5.1.18)
contig_22	fig|6666666.28487.peg.1440	Protein	10607	10798	+	FIG00820633: hypothetical protein
contig_22	fig|6666666.28487.peg.1441	Protein	10867	11886	+	Inner membrane protein YihY, formerly thought to be RNase

						BN
contig_22	fig|6666666.28487.peg.1442	Protein	12112	11876	-	FIG00998432: hypothetical protein
contig_22	fig|6666666.28487.peg.1443	Protein	12190	12591	+	Possible membrane protein
contig_22	fig|6666666.28487.peg.1444	Protein	12739	13698	+	RNA polymerase sigma factor SigB
contig_22	fig|6666666.28487.peg.1445	Protein	13847	14539	+	Iron-dependent repressor IdeR/DtxR
contig_22	fig|6666666.28487.peg.1446	Protein	15782	14541	-	FIG00999418: hypothetical protein
contig_22	fig|6666666.28487.peg.1447	Protein	15781	17196	+	Soluble pyridine nucleotide transhydrogenase (EC 1.6.1.1)
contig_22	fig|6666666.28487.peg.1448	Protein	18003	17203	-	hypothetical protein
contig_22	fig|6666666.28487.peg.1449	Protein	18243	19235	+	FIG00994994: hypothetical protein
contig_22	fig|6666666.28487.peg.1450	Protein	19278	20300	+	alpha/beta hydrolase fold
contig_22	fig|6666666.28487.peg.1451	Protein	20332	21018	+	Phenazine biosynthesis protein PhzF like
contig_22	fig|6666666.28487.peg.1452	Protein	21489	21025	-	Ribonucleotide reductase transcriptional regulator NrdR
contig_22	fig|6666666.28487.peg.1453	Protein	21972	21652	-	Cell division protein DivIC (FtsB), stabilizes FtsL against RasP cleavage
contig_22	fig|6666666.28487.peg.1454	Protein	22317	22180	-	hypothetical protein
contig_22	fig|6666666.28487.peg.1455	Protein	22358	23050	+	SOS-response repressor and protease LexA (EC 3.4.21.88)
contig_22	fig|6666666.28487.peg.1456	Protein	25150	23072	-	Possible conserved transmembrane alanine and glycine rich protein
contig_22	fig|6666666.28487.peg.1457	Protein	26517	25348	-	Acyl-CoA dehydrogenase FadE20
contig_22	fig|6666666.28487.peg.1458	Protein	26667	28904	+	Biotin sulfoxide reductase (EC 1.-.-.-)

contig_22	fig|6666666.28487.peg.1459	Protein	30214	28877	-	ATP-dependent RNA helicase
contig_22	fig|6666666.28487.peg.1460	Protein	31838	30426	-	GTP-binding protein HflX
contig_22	fig|6666666.28487.peg.1461	Protein	32808	31939	-	Diaminopimelate epimerase (EC 5.1.1.7)
contig_22	fig|6666666.28487.peg.1462	Protein	33739	32813	-	tRNA delta(2)-isopentenylpyrophosphate transferase (EC 2.5.1.8)
contig_22	fig|6666666.28487.peg.1463	Protein	34422	33736	-	FIG01121091: hypothetical protein
contig_22	fig|6666666.28487.peg.1464	Protein	35323	34436	-	hypothetical protein
contig_22	fig|6666666.28487.peg.1465	Protein	35504	36889	+	ATPase involved in DNA repair
contig_22	fig|6666666.28487.peg.1466	Protein	37484	36894	-	putative conserved transmembrane protein
contig_22	fig|6666666.28487.peg.1467	Protein	39046	37481	-	tRNA-i(6)A37 methylthiotransferase
contig_22	fig|6666666.28487.peg.1468	Protein	39266	40000	+	amino acid ABC transporter, ATP-binding protein (glnQ)
contig_22	fig|6666666.28487.peg.1469	Protein	40036	40869	+	glnH, putative
contig_22	fig|6666666.28487.peg.1470	Protein	40896	41576	+	amino acid ABC transporter, permease protein
contig_22	fig|6666666.28487.peg.1471	Protein	41573	42451	+	glutamate permease
contig_22	fig|6666666.28487.peg.1472	Protein	42461	42883	+	Sll0939 protein
contig_22	fig|6666666.28487.peg.1473	Protein	42880	43140	+	hypothetical protein
contig_22	fig|6666666.28487.peg.1474	Protein	43703	43137	-	Regulatory protein RecX
contig_22	fig|6666666.28487.peg.1475	Protein	44724	43672	-	RecA protein
contig_22	fig|6666666.28487.peg.1476	Protein	44893	45786	+	dienelactone hydrolase
contig_22	fig|6666666.28487.peg.1477	Protein	46240	45761	-	OsmC/Ohr family protein

contig_22	fig|6666666.28487.peg.1478	Protein	46442	46248	-	FIG00820724: hypothetical protein
contig_22	fig|6666666.28487.peg.1479	Protein	47624	46452	-	Alanine rich transferase
contig_22	fig|6666666.28487.peg.1480	Protein	47668	48120	+	FIG00820841: hypothetical protein
contig_22	fig|6666666.28487.peg.1481	Protein	48961	48182	-	Possible conserved membrane alanine rich protein
contig_22	fig|6666666.28487.peg.1482	Protein	49841	49017	-	Phage shock protein A (IM30) , suppresses sigma54-dependent transcription
contig_22	fig|6666666.28487.peg.1483	Protein	50286	49966	-	Transcriptional regulator, XRE family
contig_22	fig|6666666.28487.peg.1484	Protein	50863	50375	-	C-terminal domain of CinA type S
contig_22	fig|6666666.28487.peg.1485	Protein	51392	50841	-	CDP-diacylglycerol--glycerol-3-phosphate 3-phosphatidyltransferase (EC 2.7.8.5)
contig_22	fig|6666666.28487.peg.1486	Protein	51480	52016	+	Acetyltransferase, GNAT family
contig_22	fig|6666666.28487.peg.1487	Protein	53075	52017	-	Chalcone synthase (EC 2.3.1.74)
contig_22	fig|6666666.28487.peg.1488	Protein	53952	53062	-	putative integral membrane protein
contig_22	fig|6666666.28487.peg.1489	Protein	54082	55083	+	Methyltransferase type 12
contig_22	fig|6666666.28487.peg.1490	Protein	57449	55080	-	Cell division protein FtsK
contig_22	fig|6666666.28487.peg.1491	Protein	57792	58106	+	Antibiotic biosynthesis monooxygenase
contig_22	fig|6666666.28487.peg.1492	Protein	58119	58937	+	Dehydrogenase
contig_22	fig|6666666.28487.peg.1493	Protein	58976	59806	+	O-Methyltransferase involved in polyketide biosynthesis
contig_22	fig|6666666.28487.peg.1494	Protein	60083	60895	+	two component system response regulator
contig_22	fig|6666666.28487.peg.1495	Protein	62959	61283	-	Ribonuclease J2 (endoribonuclease in RNA processing)

contig_22	fig|6666666.28487.peg.1496	Protein	63878	62970	-	Dihydrodipicolinate synthase (EC 4.2.1.52)
contig_22	fig|6666666.28487.peg.1497	Protein	64676	63924	-	Thymidylate synthase thyX (EC 2.1.1.-)
contig_22	fig|6666666.28487.peg.1498	Protein	65840	64722	-	diguanylate cyclase (GGDEF domain) with PAS/PAC sensor
contig_22	fig|6666666.28487.peg.1499	Protein	66647	65880	-	tRNA-specific adenosine-34 deaminase (EC 3.5.4.-) / domain of unknown function
contig_22	fig|6666666.28487.peg.1500	Protein	67993	66779	-	FIG00822893: hypothetical protein
contig_22	fig|6666666.28487.peg.1501	Protein	68472	67990	-	Dihydrofolate reductase (EC 1.5.1.3)
contig_22	fig|6666666.28487.peg.1502	Protein	69269	68469	-	Thymidylate synthase (EC 2.1.1.45)
contig_22	fig|6666666.28487.peg.1503	Protein	69312	70049	+	Dienelactone hydrolase family
contig_22	fig|6666666.28487.peg.1504	Protein	70355	70071	-	hypothetical protein
contig_22	fig|6666666.28487.peg.1505	Protein	70848	71375	+	hypothetical protein
contig_22	fig|6666666.28487.peg.1506	Protein	72121	71387	-	3-oxoacyl-[acyl-carrier protein] reductase (EC 1.1.1.100)
contig_22	fig|6666666.28487.peg.1507	Protein	72691	72236	-	Multimeric flavodoxin WrbA
contig_22	fig|6666666.28487.peg.1508	Protein	73155	72688	-	FIG00823389: hypothetical protein
contig_22	fig|6666666.28487.peg.1509	Protein	73889	73152	-	Dihydrodipicolinate reductase (EC 1.3.1.26)
contig_22	fig|6666666.28487.peg.1510	Protein	74016	75515	+	Partial REP13E12 repeat protein
contig_22	fig|6666666.28487.peg.1511	Protein	75535	75918	+	FIG00821738: hypothetical protein
contig_22	fig|6666666.28487.peg.1512	Protein	76314	75919	-	Transcriptional regulator, AsnC family
contig_22	fig|6666666.28487.peg.1513	Protein	76528	77643	+	Alanine dehydrogenase (EC 1.4.1.1)
contig_22	fig|6666666.28487.peg.1514	Protein	78075	77701	-	UPF0225 protein YchJ

contig_22	fig|6666666.28487.peg.1515	Protein	78101	78691	+	FIG00830608: hypothetical protein
contig_22	fig|6666666.28487.peg.1516	Protein	79587	78688	-	Beta-lactamase (EC 3.5.2.6)
contig_22	fig|6666666.28487.peg.1517	Protein	80880	79606	-	FIG007959: peptidase, M16 family
contig_22	fig|6666666.28487.peg.1518	Protein	83164	80936	-	Polyribonucleotide nucleotidyltransferase (EC 2.7.7.8)
contig_22	fig|6666666.28487.peg.1519	Protein	83853	83584	-	SSU ribosomal protein S15p (S13e)
contig_22	fig|6666666.28487.peg.1520	Protein	84969	83995	-	Riboflavin kinase (EC 2.7.1.26) / FMN adenylyltransferase (EC 2.7.7.2)
contig_22	fig|6666666.28487.peg.1521	Protein	85062	85778	+	Mn-dependent transcriptional regulator MntR
contig_22	fig|6666666.28487.peg.1522	Protein	85788	86219	+	FIG00826705: hypothetical protein
contig_22	fig|6666666.28487.peg.1523	Protein	86865	86224	-	hypothetical protein
contig_22	fig|6666666.28487.peg.1524	Protein	87437	86862	-	Transcriptional regulator, TetR family
contig_22	fig|6666666.28487.peg.1525	Protein	87602	87982	+	conserved hypothetical protein
contig_22	fig|6666666.28487.peg.1526	Protein	89142	87976	-	integral membrane sensor signal transduction histidine kinase
contig_22	fig|6666666.28487.peg.1527	Protein	89801	89139	-	Two-component response regulator
contig_22	fig|6666666.28487.peg.1528	Protein	90044	89853	-	hypothetical protein
contig_22	fig|6666666.28487.peg.1529	Protein	90949	90068	-	tRNA pseudouridine synthase B (EC 4.2.1.70)
contig_22	fig|6666666.28487.peg.1530	Protein	91632	90946	-	4'-phosphopantetheinyl transferase EntD (EC 2.7.8.-)
contig_22	fig|6666666.28487.peg.1531	Protein	92576	91629	-	putative SimX4 homolog
contig_22	fig|6666666.28487.peg.1532	Protein	92690	93313	+	Possible membrane protein
contig_22	fig|6666666.28487.peg.1533	Protein	93393	94967	+	Glutaryl-7-ACA acylase

contig_22	fig|6666666.28487.peg.1534	Protein	95516	94956	-	FIG00820705: hypothetical protein
contig_22	fig|6666666.28487.peg.1535	Protein	95779	95513	-	COGs COG0840
contig_22	fig|6666666.28487.peg.1536	Protein	95836	96600	+	3-hydroxybutyryl-CoA dehydratase (EC 4.2.1.55)
contig_22	fig|6666666.28487.peg.1537	Protein	97418	96597	-	Sugar phosphate isomerases/epimerase
contig_22	fig|6666666.28487.peg.1538	Protein	98608	97418	-	Conserved hypothetical protein potentially related to ribose or hydroxymethylpyrimidine metabolism
contig_22	fig|6666666.28487.peg.1539	Protein	99726	98605	-	GLUCOSE-FRUCTOSE OXIDOREDUCTASE (EC 1.1.99.28)
contig_22	fig|6666666.28487.peg.1540	Protein	99725	99850	+	hypothetical protein
contig_22	fig|6666666.28487.peg.1541	Protein	100007	101032	+	Transcriptional regulator, LacI family
contig_22	fig|6666666.28487.peg.1542	Protein	101167	102048	+	Hydroxymethylpyrimidine ABC transporter, transmembrane component
contig_22	fig|6666666.28487.peg.1543	Protein	102074	103108	+	Hydroxymethylpyrimidine ABC transporter, substrate-binding component
contig_22	fig|6666666.28487.peg.1544	Protein	103233	103099	-	hypothetical protein
contig_22	fig|6666666.28487.peg.1545	Protein	103202	103942	+	Hydroxymethylpyrimidine ABC transporter, ATPase component
contig_22	fig|6666666.28487.peg.1546	Protein	104467	104036	-	Transcriptional regulator, MerR family
contig_22	fig|6666666.28487.peg.1547	Protein	104514	105125	+	SAM-dependent methyltransferase (EC 2.1.1.-)
contig_22	fig|6666666.28487.peg.1548	Protein	106470	105142	-	putative DNA-damage-inducible protein F
contig_22	fig|6666666.28487.peg.1549	Protein	107458	106454	-	FIG146085: 3'-to-5' oligoribonuclease A, Bacillus type
contig_22	fig|6666666.28487.peg.1550	Protein	107927	107433	-	Ribosome-binding factor A

contig_22	fig|6666666.28487.peg.1551	Protein	109760	107928	-	Translation initiation factor 2
contig_22	fig|6666666.28487.peg.1552	Protein	109722	110561	+	FIG00820868: hypothetical protein
contig_22	fig|6666666.28487.peg.1553	Protein	111173	110811	-	COG2740: Predicted nucleic-acid-binding protein implicated in transcription termination
contig_22	fig|6666666.28487.peg.1554	Protein	112301	111240	-	Transcription termination protein NusA
contig_22	fig|6666666.28487.peg.1555	Protein	112789	112298	-	COG0779: clustered with transcription termination protein NusA
contig_22	fig|6666666.28487.peg.1556	Protein	113079	112786	-	hypothetical protein
contig_22	fig|6666666.28487.peg.1557	Protein	113039	113515	+	PROBABLE CONSERVED TRANSMEMBRANE ALANINE RICH PROTEIN
contig_22	fig|6666666.28487.peg.1558	Protein	113512	113988	+	FIG00999285: hypothetical protein
contig_22	fig|6666666.28487.peg.1559	Protein	115753	113996	-	Prolyl-tRNA synthetase (EC 6.1.1.15)
contig_22	fig|6666666.28487.peg.1560	Protein	117263	115773	-	Putative transmembrane efflux protein
contig_23	fig|6666666.28487.peg.1561	Protein	1047	61	-	Cytochrome c-type biogenesis protein CcsA/ResC
contig_23	fig|6666666.28487.peg.1562	Protein	2693	1044	-	Ccs1/ResB-related putative cytochrome C-type biogenesis protein
contig_23	fig|6666666.28487.peg.1563	Protein	3472	2693	-	Cytochrome c-type biogenesis protein CcdA (DsbD analog)
contig_23	fig|6666666.28487.peg.1564	Protein	4059	3469	-	Thiol:disulfide oxidoreductase related to ResA
contig_23	fig|6666666.28487.peg.1565	Protein	4652	4068	-	Hypothetical, similarity to phosphoglycerate mutase
contig_23	fig|6666666.28487.peg.1566	Protein	5998	4700	-	Glutamate-1-semialdehyde aminotransferase (EC 5.4.3.8)
contig_23	fig|6666666.28487.peg.1567	Protein	7574	6051	-	S-adenosylhomocysteine deaminase (EC 3.5.4.28);

						Methylthioadenosine deaminase
contig_23	fig|6666666.28487.peg.1568	Protein	9070	7571	-	Cytosine/purine/uracil/thiamine/allantoin permease family protein
contig_23	fig|6666666.28487.peg.1569	Protein	9172	10665	+	putative regulatory protein
contig_23	fig|6666666.28487.peg.1570	Protein	11078	10698	-	FIG00821089: hypothetical protein
contig_23	fig|6666666.28487.peg.1571	Protein	12495	11131	-	putative cytochrome P450
contig_23	fig|6666666.28487.peg.1572	Protein	12979	12479	-	FIG00826102: hypothetical protein
contig_23	fig|6666666.28487.peg.1573	Protein	13356	13024	-	FIG00825655: hypothetical protein
contig_23	fig|6666666.28487.peg.1574	Protein	13989	13399	-	Transcriptional regulator, TetR family
contig_23	fig|6666666.28487.peg.1575	Protein	14887	14024	-	FIG00822021: hypothetical protein
contig_23	fig|6666666.28487.peg.1576	Protein	15225	15004	-	putative transmembrane protein
contig_23	fig|6666666.28487.peg.1577	Protein	15758	15267	-	Transmembrane transport protein MmpL2
contig_23	fig|6666666.28487.peg.1578	Protein	16147	15755	-	FIG00826307: hypothetical protein
contig_23	fig|6666666.28487.peg.1579	Protein	16578	16204	-	FIG00820800: hypothetical protein
contig_23	fig|6666666.28487.peg.1580	Protein	17510	16623	-	Porphobilinogen synthase (EC 4.2.1.24)
contig_23	fig|6666666.28487.peg.1581	Protein	19336	17636	-	Uroporphyrinogen-III methyltransferase (EC 2.1.1.107) / Uroporphyrinogen-III synthase (EC 4.2.1.75)
contig_23	fig|6666666.28487.peg.1582	Protein	20268	19333	-	Porphobilinogen deaminase (EC 2.5.1.61)
contig_23	fig|6666666.28487.peg.1583	Protein	21661	20294	-	Glutamyl-tRNA reductase (EC 1.2.1.70)
contig_23	fig|6666666.28487.peg.1584	Protein	21991	21749	-	FIG00822170: hypothetical protein

contig_23	fig|6666666.28487.peg.1585	Protein	22067	22954	+	Phosphoserine phosphatase (EC 3.1.3.3)
contig_23	fig|6666666.28487.peg.1586	Protein	22988	23527	+	(3R)-hydroxyacyl-ACP dehydratase subunit HadA
contig_23	fig|6666666.28487.peg.1587	Protein	24681	23611	-	Phospholipid/glycerol acyltransferase
contig_23	fig|6666666.28487.peg.1588	Protein	25766	24723	-	UDP-glucose 4-epimerase (EC 5.1.3.2)
contig_23	fig|6666666.28487.peg.1589	Protein	25848	25961	+	hypothetical protein
contig_23	fig|6666666.28487.peg.1590	Protein	26385	26122	-	"DNA binding domain protein, excisionase family"
contig_23	fig|6666666.28487.peg.1591	Protein	27419	26535	-	Pyrroline-5-carboxylate reductase (EC 1.5.1.2)
contig_23	fig|6666666.28487.peg.1592	Protein	28247	27429	-	TesB-like acyl-CoA thioesterase 3
contig_23	fig|6666666.28487.peg.1593	Protein	29109	28264	-	"AP endonuclease, family protein 2"
contig_23	fig|6666666.28487.peg.1594	Protein	30306	29113	-	hypothetical protein
contig_23	fig|6666666.28487.peg.1595	Protein	31399	30335	-	Exopolyphosphatase (EC 3.6.1.11)
contig_23	fig|6666666.28487.peg.1596	Protein	31445	32227	+	FIG00820527: hypothetical protein
contig_23	fig|6666666.28487.peg.1597	Protein	32224	32526	+	YCII-related domain-containing protein
contig_23	fig|6666666.28487.peg.1598	Protein	34907	32541	-	Glutamate-1-semialdehyde aminotransferase (EC 5.4.3.8)
contig_23	fig|6666666.28487.peg.1599	Protein	34982	35557	+	dTDP-4-dehydrorhamnose 3,5-epimerase (EC 5.1.3.13)
contig_23	fig|6666666.28487.peg.1600	Protein	36575	35541	-	UDP-glucose 4-epimerase (EC 5.1.3.2)
contig_23	fig|6666666.28487.peg.1601	Protein	37216	36572	-	FIG01131441: hypothetical protein
contig_23	fig|6666666.28487.peg.1602	Protein	38028	37228	-	Glucose-1-phosphate cytidylyltransferase (EC 2.7.7.33)
contig_23	fig|6666666.28487.peg.1603	Protein	39239	38025	-	methyltransferase, putative

contig_23	fig|6666666.28487.peg.1604	Protein	40585	39266	-	Oligosaccharide repeat unit transporter
contig_23	fig|6666666.28487.peg.1605	Protein	41480	40587	-	glycosyl transferase, family 2
contig_23	fig|6666666.28487.peg.1606	Protein	42669	41983	-	Phosphate regulon transcriptional regulatory protein PhoB (SphR); Sensory transduction protein regX3
contig_23	fig|6666666.28487.peg.1607	Protein	43882	42677	-	Phosphate regulon sensor protein PhoR (SphS) (EC 2.7.13.3)
contig_23	fig|6666666.28487.peg.1608	Protein	44776	44024	-	Phosphoglycerate mutase (EC 5.4.2.1)
contig_23	fig|6666666.28487.peg.1609	Protein	45347	44799	-	Uncharacterized protein Rv0487/MT0505 clustered with mycothiol biosynthesis gene
contig_23	fig|6666666.28487.peg.1610	Protein	46600	45344	-	Glycosyltransferase MshA involved in mycothiol biosynthesis (EC 2.4.1.-)
contig_23	fig|6666666.28487.peg.1611	Protein	48065	46728	-	Transcriptional regulatory protein
contig_23	fig|6666666.28487.peg.1612	Protein	48216	48971	+	Serine 3-dehydrogenase
contig_23	fig|6666666.28487.peg.1613	Protein	49098	49217	+	hypothetical protein
contig_23	fig|6666666.28487.peg.1614	Protein	50512	49214	-	Possible lipoprotein LprQ
contig_23	fig|6666666.28487.peg.1615	Protein	51577	50528	-	UDP-N-acetylenolpyruvoylglucosamine reductase (EC 1.1.1.158)
contig_23	fig|6666666.28487.peg.1616	Protein	51604	52125	+	FIG00996480: hypothetical protein
contig_23	fig|6666666.28487.peg.1617	Protein	52168	52944	+	Methylase of polypeptide chain release factors
contig_23	fig|6666666.28487.peg.1618	Protein	53968	52907	-	conserved hypothetical protein
contig_23	fig|6666666.28487.peg.1619	Protein	55516	53978	-	sodium-solute symporter, putative
contig_23	fig|6666666.28487.peg.1620	Protein	55860	57008	+	Agmatinase (EC 3.5.3.11)

contig_23	fig|6666666.28487.peg.1621	Protein	57098	57547	+	Nitrite-sensitive transcriptional repressor NsrR
contig_23	fig|6666666.28487.peg.1622	Protein	57544	58740	+	Flavohemoprotein (Hemoglobin-like protein) (Flavohemoglobin) (Nitric oxide dioxygenase) (EC 1.14.12.17)
contig_23	fig|6666666.28487.peg.1623	Protein	58758	59582	+	Carbon-nitrogen hydrolase
contig_23	fig|6666666.28487.peg.1624	Protein	59762	60436	+	hypothetical protein
contig_23	fig|6666666.28487.peg.1625	Protein	61113	60433	-	Deoxyribose-phosphate aldolase (EC 4.1.2.4)
contig_23	fig|6666666.28487.peg.1626	Protein	61514	61113	-	POSSIBLE CONSERVED SECRETED PROTEIN
contig_23	fig|6666666.28487.peg.1627	Protein	61738	61514	-	FIG00820770: hypothetical membrane protein
contig_23	fig|6666666.28487.peg.1628	Protein	62487	61852	-	Iron-regulated heparin binding hemagglutinin HbhA (Adhesin)
contig_23	fig|6666666.28487.peg.1629	Protein	62968	62480	-	"Transcriptional regulator, XRE family"
contig_23	fig|6666666.28487.peg.1630	Protein	64407	63067	-	Possible membrane protein
contig_23	fig|6666666.28487.peg.1631	Protein	64521	65225	+	"Transcriptional regulator, TetR family"
contig_23	fig|6666666.28487.peg.1632	Protein	65230	66114	+	UDP-galactose-lipid carrier transferase (EC 2.-.-.-)
contig_23	fig|6666666.28487.peg.1633	Protein	67074	66220	-	3-hydroxybutyryl-CoA dehydrogenase (EC 1.1.1.157); 3-hydroxyacyl-CoA dehydrogenase (EC 1.1.1.35)
contig_23	fig|6666666.28487.peg.1634	Protein	68504	67212	-	Isocitrate lyase (EC 4.1.3.1) / Methylisocitrate lyase (EC 4.1.3.30)
contig_23	fig|6666666.28487.peg.1635	Protein	69615	68785	-	Acyl-ACP thioesterase
contig_23	fig|6666666.28487.peg.1636	Protein	69705	71150	+	"Transcriptional regulator, XRE family"
contig_23	fig|6666666.28487.peg.1637	Protein	71147	71722	+	FIG00821074: hypothetical protein
contig_23	fig|6666666.28487.peg.1638	Protein	71790	72758	+	Hydrolase, alpha/beta fold family protein

contig_23	fig|6666666.28487.peg.1639	Protein	73856	72765	-	diguanylate cyclase
contig_23	fig|6666666.28487.peg.1640	Protein	74149	73871	-	hypothetical protein
contig_23	fig|6666666.28487.peg.1641	Protein	75556	74150	-	Dihydrolipoamide dehydrogenase (EC 1.8.1.4)
contig_23	fig|6666666.28487.peg.1642	Protein	76180	75563	-	Putative ABC transport system, ATP-binding subunit
contig_23	fig|6666666.28487.peg.1643	Protein	76935	76177	-	putative ABC transporter ATP-binding protein
contig_23	fig|6666666.28487.peg.1644	Protein	77732	76932	-	binding-protein-dependent transport systems inner membrane component
contig_23	fig|6666666.28487.peg.1645	Protein	78682	77729	-	binding-protein-dependent transport systems inner membrane component
contig_23	fig|6666666.28487.peg.1646	Protein	80328	78760	-	putative substrate-binding transport protein
contig_23	fig|6666666.28487.peg.1647	Protein	80839	80405	-	PROBABLE TRANSMEMBRANE PROTEIN
contig_23	fig|6666666.28487.peg.1648	Protein	81074	80832	-	hypothetical protein
contig_23	fig|6666666.28487.peg.1649	Protein	81736	81302	-	hypothetical protein
contig_23	fig|6666666.28487.peg.1650	Protein	82312	81836	-	FIG00821442: hypothetical protein
contig_23	fig|6666666.28487.peg.1651	Protein	83582	82314	-	Transcriptional activator of acetoin metabolism
contig_23	fig|6666666.28487.peg.1652	Protein	84415	83621	-	2,3-butanediol dehydrogenase, S-alcohol forming, (S)-acetoin-specific (EC 1.1.1.76)
contig_23	fig|6666666.28487.peg.1653	Protein	85455	84412	-	Threonine dehydrogenase and related Zn-dependent dehydrogenases
contig_23	fig|6666666.28487.peg.1654	Protein	85587	87407	+	Flavin-containing monooxygenase
contig_23	fig|6666666.28487.peg.1655	Protein	87780	87490	-	hypothetical protein

contig_23	fig|6666666.28487.peg.1656	Protein	88891	87824	-	hypothetical protein
contig_23	fig|6666666.28487.peg.1657	Protein	88980	91007	+	Prolyl endopeptidase (EC 3.4.21.26)
contig_23	fig|6666666.28487.peg.1658	Protein	91009	91662	+	hypothetical protein
contig_23	fig|6666666.28487.peg.1659	Protein	91659	91979	+	hypothetical protein
contig_23	fig|6666666.28487.peg.1660	Protein	92053	92535	+	protein of unknown function DUF417
contig_23	fig|6666666.28487.peg.1661	Protein	92537	93172	+	RNA polymerase sigma factor RpoE
contig_23	fig|6666666.28487.peg.1662	Protein	93169	93411	+	hypothetical protein
contig_23	fig|6666666.28487.peg.1663	Protein	93464	94375	+	Enoyl-CoA hydratase (EC 4.2.1.17)
contig_23	fig|6666666.28487.peg.1664	Protein	94425	95927	+	2-polyprenyl-6-methoxyphenol hydroxylase and related FAD-dependent oxidoreductases
contig_23	fig|6666666.28487.peg.1665	Protein	95924	96520	+	Transcriptional regulator, TetR family
contig_23	fig|6666666.28487.peg.1666	Protein	97166	96525	-	hypothetical protein
contig_23	fig|6666666.28487.peg.1667	Protein	98066	97212	-	FIG00819982: hypothetical protein
contig_23	fig|6666666.28487.peg.1668	Protein	98986	98102	-	Polyphosphate kinase 2 (EC 2.7.4.1)
contig_23	fig|6666666.28487.peg.1669	Protein	100576	99047	-	Aldehyde dehydrogenase (EC 1.2.1.3)
contig_23	fig|6666666.28487.peg.1670	Protein	101074	100586	-	FIG00826663: hypothetical protein
contig_23	fig|6666666.28487.peg.1671	Protein	101661	101158	-	FIG00820188: hypothetical protein
contig_23	fig|6666666.28487.peg.1672	Protein	102646	101672	-	Serine/threonine protein kinase (EC 2.7.11.1)
contig_23	fig|6666666.28487.peg.1673	Protein	103039	102650	-	PROBABLE LACTOYLGLUTATHIONE LYASE (EC 4.4.1.5)
contig_23	fig|6666666.28487.peg.1674	Protein	103062	104786	+	D-aminoacylase (EC 3.5.1.81)

contig_23	fig|6666666.28487.peg.1675	Protein	104792	105337	+	Carboxymuconolactone decarboxylase
contig_23	fig|6666666.28487.peg.1676	Protein	106141	105329	-	short-chain dehydrogenase/reductase SDR
contig_23	fig|6666666.28487.peg.1677	Protein	106174	106545	+	FIG00825576: hypothetical protein
contig_23	fig|6666666.28487.peg.1678	Protein	107560	106535	-	Bifunctional protein: zinc-containing alcohol dehydrogenase; quinone oxidoreductase (NADPH:quinone reductase) (EC 1.1.1.-); Similar to arginate lyase
contig_23	fig|6666666.28487.peg.1679	Protein	107640	108959	+	major facilitator family transporter
contig_23	fig|6666666.28487.peg.1680	Protein	110701	109076	-	Heat shock protein 60 family chaperone GroEL
contig_23	fig|6666666.28487.peg.1681	Protein	111517	110897	-	Transcriptional regulator, TetR family
contig_23	fig|6666666.28487.peg.1682	Protein	111620	112531	+	probable oxidoreductase/Short-chain dehydrogenase
contig_23	fig|6666666.28487.peg.1683	Protein	113675	112542	-	PPE family protein
contig_23	fig|6666666.28487.peg.1684	Protein	113777	114286	+	FIG00823588: hypothetical protein
contig_23	fig|6666666.28487.peg.1685	Protein	114378	116081	+	Type III restriction enzyme, res subunit
contig_23	fig|6666666.28487.peg.1686	Protein	116186	117265	+	regulatory protein, LuxR
contig_23	fig|6666666.28487.peg.1687	Protein	118610	117255	-	hypothetical protein
contig_23	fig|6666666.28487.peg.1688	Protein	119160	118669	-	FIG00829777: hypothetical protein
contig_23	fig|6666666.28487.peg.1689	Protein	120123	119191	-	POSSIBLE OXIDOREDUCTASE (EC 1.-.-.-)
contig_23	fig|6666666.28487.peg.1690	Protein	120942	120127	-	HAD-superfamily subfamily IIA hydrolase, hypothetical 2
contig_23	fig|6666666.28487.peg.1691	Protein	121025	121819	+	putative transcriptional regulator, MerR family
contig_23	fig|6666666.28487.peg.1692	Protein	123237	121801	-	transmembrane protein, distant homology with ydbT

contig_23	fig|6666666.28487.peg.1693	Protein	123626	123234	-	transmembrane protein, distant homology with ydbS
contig_23	fig|6666666.28487.peg.1694	Protein	123753	124670	+	probable oxidoreductase/Short-chain dehydrogenase
contig_23	fig|6666666.28487.peg.1695	Protein	124706	125875	+	Molybdopterin biosynthesis protein MoeA
contig_23	fig|6666666.28487.peg.1696	Protein	125967	126593	+	Phosphatidylserine decarboxylase (EC 4.1.1.65)
contig_23	fig|6666666.28487.peg.1697	Protein	126593	127459	+	CDP-diacylglycerol--serine O-phosphatidyltransferase (EC 2.7.8.8)
contig_23	fig|6666666.28487.peg.1698	Protein	127461	129629	+	Cell division protein FtsH (EC 3.4.24.-)
contig_23	fig|6666666.28487.peg.1699	Protein	129788	130231	+	FIG00821770: hypothetical protein
contig_23	fig|6666666.28487.peg.1700	Protein	131762	130266	-	Na(+)/H(+) antiporter
contig_23	fig|6666666.28487.peg.1701	Protein	131794	132489	+	Trk system potassium uptake protein TrkA
contig_23	fig|6666666.28487.peg.1702	Protein	132536	135406	+	Na(+) H(+) antiporter subunit A; Na(+) H(+) antiporter subunit B
contig_23	fig|6666666.28487.peg.1703	Protein	135403	135873	+	Na(+) H(+) antiporter subunit C
contig_23	fig|6666666.28487.peg.1704	Protein	135870	137477	+	Na(+) H(+) antiporter subunit D
contig_23	fig|6666666.28487.peg.1705	Protein	137634	137449	-	hypothetical protein
contig_23	fig|6666666.28487.peg.1706	Protein	137618	138100	+	Na(+) H(+) antiporter subunit E
contig_23	fig|6666666.28487.peg.1707	Protein	138097	138384	+	Na(+) H(+) antiporter subunit F
contig_23	fig|6666666.28487.peg.1708	Protein	138381	138749	+	Na(+) H(+) antiporter subunit G
contig_23	fig|6666666.28487.peg.1709	Protein	138739	139470	+	FIG00822978: hypothetical protein
contig_23	fig|6666666.28487.peg.1710	Protein	139552	139842	+	hypothetical protein
contig_23	fig|6666666.28487.peg.1711	Protein	140481	139849	-	Uncharacterized protein, similar to the N-terminal domain of

						Lon protease
contig_23	fig|6666666.28487.peg.1712	Protein	141615	140485	-	Carboxylate-amine ligase
contig_23	fig|6666666.28487.peg.1713	Protein	142294	141596	-	Superoxide dismutase [Cu-Zn] precursor (EC 1.15.1.1)
contig_23	fig|6666666.28487.peg.1714	Protein	142760	142305	-	Tuberculin related peptide
contig_23	fig|6666666.28487.peg.1715	Protein	143111	142803	-	FIG01000274: hypothetical protein
contig_23	fig|6666666.28487.peg.1716	Protein	143284	143853	+	Peptide deformylase (EC 3.5.1.88)
contig_23	fig|6666666.28487.peg.1717	Protein	143929	144756	+	Histone acetyltransferase HPA2 and related acetyltransferases
contig_23	fig|6666666.28487.peg.1718	Protein	144761	145585	+	Exodeoxyribonuclease III (EC 3.1.11.2)
contig_24	fig|6666666.28487.peg.1719	Protein	644	45	-	Transcriptional regulator, TetR family
contig_24	fig|6666666.28487.peg.1720	Protein	722	2131	+	Ubiquinone biosynthesis monooxygenase UbiB
contig_24	fig|6666666.28487.peg.1721	Protein	2118	3362	+	cytochrome P450
contig_24	fig|6666666.28487.peg.1722	Protein	3539	4054	+	Mycofactocin precursor
contig_24	fig|6666666.28487.peg.1723	Protein	4057	4350	+	Mycofactocin system small protein
contig_24	fig|6666666.28487.peg.1724	Protein	4347	5537	+	Mycofactocin radical SAM maturase
contig_24	fig|6666666.28487.peg.1725	Protein	5540	6715	+	Mycofactocin system heme/flavin dehydrogenase
contig_24	fig|6666666.28487.peg.1726	Protein	7719	6829	-	hypothetical protein
contig_24	fig|6666666.28487.peg.1727	Protein	7690	8970	+	Mycofactocin system glycosyltransferase
contig_24	fig|6666666.28487.peg.1728	Protein	8971	10395	+	Choline dehydrogenase (EC 1.1.99.1)
contig_24	fig|6666666.28487.peg.1729	Protein	11947	10364	-	Integral membrane protein

contig_24	fig|6666666.28487.peg.1730	Protein	12033	12671	+	transcriptional regulator, TetR family
contig_24	fig|6666666.28487.peg.1731	Protein	13887	12637	-	putative cytochrome P450 hydroxylase
contig_24	fig|6666666.28487.peg.1732	Protein	14005	15561	+	carboxylesterase, type B
contig_24	fig|6666666.28487.peg.1733	Protein	15892	15563	-	hypothetical protein
contig_24	fig|6666666.28487.peg.1734	Protein	15924	16142	+	hypothetical protein
contig_24	fig|6666666.28487.peg.1735	Protein	16415	16720	+	SSU ribosomal protein S10p (S20e)
contig_24	fig|6666666.28487.peg.1736	Protein	16736	17389	+	LSU ribosomal protein L3p (L3e)
contig_24	fig|6666666.28487.peg.1737	Protein	17389	18051	+	LSU ribosomal protein L4p (L1e)
contig_24	fig|6666666.28487.peg.1738	Protein	18051	18353	+	LSU ribosomal protein L23p (L23Ae)
contig_24	fig|6666666.28487.peg.1739	Protein	18375	19211	+	LSU ribosomal protein L2p (L8e)
contig_24	fig|6666666.28487.peg.1740	Protein	19223	19504	+	SSU ribosomal protein S19p (S15e)
contig_24	fig|6666666.28487.peg.1741	Protein	19536	20012	+	LSU ribosomal protein L22p (L17e)
contig_24	fig|6666666.28487.peg.1742	Protein	20012	20839	+	SSU ribosomal protein S3p (S3e)
contig_24	fig|6666666.28487.peg.1743	Protein	20843	21259	+	LSU ribosomal protein L16p (L10e)
contig_24	fig|6666666.28487.peg.1744	Protein	21259	21492	+	LSU ribosomal protein L29p (L35e)
contig_24	fig|6666666.28487.peg.1745	Protein	21492	21797	+	SSU ribosomal protein S17p (S11e)
contig_24	fig|6666666.28487.peg.1746	Protein	22511	21861	-	Transcriptional regulator, TetR family
contig_24	fig|6666666.28487.peg.1747	Protein	22565	23215	+	membrane protein, putative
contig_24	fig|6666666.28487.peg.1748	Protein	23225	25579	+	Arylsulfatase (EC 3.1.6.1)

contig_24	fig|6666666.28487.peg.1749	Protein	25585	26241	+	hypothetical protein
contig_24	fig|6666666.28487.peg.1750	Protein	26246	27136	+	Sulfatase modifying factor 1 precursor (C-alpha-formyglycine-generating enzyme 1)
contig_24	fig|6666666.28487.peg.1751	Protein	27321	27689	+	LSU ribosomal protein L14p (L23e)
contig_24	fig|6666666.28487.peg.1752	Protein	27690	28010	+	LSU ribosomal protein L24p (L26e)
contig_24	fig|6666666.28487.peg.1753	Protein	28010	28573	+	LSU ribosomal protein L5p (L11e)
contig_24	fig|6666666.28487.peg.1754	Protein	28578	28763	+	SSU ribosomal protein S14p (S29e) @ SSU ribosomal protein S14p (S29e), zinc-dependent
contig_24	fig|6666666.28487.peg.1755	Protein	28863	29261	+	SSU ribosomal protein S8p (S15Ae)
contig_24	fig|6666666.28487.peg.1756	Protein	29276	29815	+	LSU ribosomal protein L6p (L9e)
contig_24	fig|6666666.28487.peg.1757	Protein	29819	30226	+	LSU ribosomal protein L18p (L5e)
contig_24	fig|6666666.28487.peg.1758	Protein	30249	30920	+	SSU ribosomal protein S5p (S2e)
contig_24	fig|6666666.28487.peg.1759	Protein	30924	31103	+	LSU ribosomal protein L30p (L7e)
contig_24	fig|6666666.28487.peg.1760	Protein	31106	31549	+	LSU ribosomal protein L15p (L27Ae)
contig_24	fig|6666666.28487.peg.1761	Protein	31664	32014	+	protein of unknown function DUF732
contig_24	fig|6666666.28487.peg.1762	Protein	32046	33818	+	Possible protease IV SppA (endopeptidase IV) (signal peptide peptidase)
contig_24	fig|6666666.28487.peg.1763	Protein	34695	33784	-	hydrolase
contig_24	fig|6666666.28487.peg.1764	Protein	34705	35952	+	Transcriptional regulator, IclR family
contig_24	fig|6666666.28487.peg.1765	Protein	36701	35949	-	3-oxoacyl-[acyl-carrier protein] reductase (EC 1.1.1.100)
contig_24	fig|6666666.28487.peg.1766	Protein	37152	36751	-	putative oxidoreductase

contig_24	fig|6666666.28487.peg.1767	Protein	38356	37304	-	Tartrate dehydrogenase (EC 1.1.1.93) / Tartrate decarboxylase (EC 4.1.1.73) / D-malic enzyme (EC 1.1.1.83)
contig_24	fig|6666666.28487.peg.1768	Protein	39957	38353	-	Sodium-dependent transporter
contig_24	fig|6666666.28487.peg.1769	Protein	40996	40079	-	FIG00824343: hypothetical protein
contig_24	fig|6666666.28487.peg.1770	Protein	41900	41001	-	O-Methyltransferase involved in polyketide biosynthesis
contig_24	fig|6666666.28487.peg.1771	Protein	42825	41887	-	O-Methyltransferase involved in polyketide biosynthesis
contig_24	fig|6666666.28487.peg.1772	Protein	43781	42855	-	FIG00819993: hypothetical protein
contig_24	fig|6666666.28487.peg.1773	Protein	44033	45370	+	Preprotein translocase secY subunit (TC 3.A.5.1.1)
contig_24	fig|6666666.28487.peg.1774	Protein	45396	45932	+	Adenylate kinase (EC 2.7.4.3)
contig_24	fig|6666666.28487.peg.1775	Protein	45933	46733	+	Methionine aminopeptidase (EC 3.4.11.18)
contig_24	fig|6666666.28487.peg.1776	Protein	46779	47303	+	RNA polymerase sigma-70 factor, ECF subfamily
contig_24	fig|6666666.28487.peg.1777	Protein	47300	48016	+	putative transmembrane anti-sigma factor
contig_24	fig|6666666.28487.peg.1778	Protein	48048	48545	+	Transcriptional regulator, MarR family
contig_24	fig|6666666.28487.peg.1779	Protein	49767	48559	-	Sensor histidine protein kinase UhpB, glucose-6-phosphate specific (EC 2.7.13.3)
contig_24	fig|6666666.28487.peg.1780	Protein	50396	49764	-	Two-component response regulator
contig_24	fig|6666666.28487.peg.1781	Protein	50447	51235	+	CbbY family protein, putative
contig_24	fig|6666666.28487.peg.1782	Protein	51272	51889	+	Transcriptional regulator, TetR family
contig_24	fig|6666666.28487.peg.1783	Protein	52783	51911	-	3-hydroxyisobutyrate dehydrogenase (EC 1.1.1.31)
contig_24	fig|6666666.28487.peg.1784	Protein	53982	52798	-	Acyl-CoA dehydrogenase, short-chain specific (EC 1.3.99.2)

contig_24	fig|6666666.28487.peg.1785	Protein	55507	53987	-	Methylmalonate-semialdehyde dehydrogenase (EC 1.2.1.27)
contig_24	fig|6666666.28487.peg.1786	Protein	56215	55604	-	dTDP-4-dehydrorhamnose 3,5-epimerase (EC 5.1.3.13)
contig_24	fig|6666666.28487.peg.1787	Protein	57219	56212	-	dTDP-glucose 4,6-dehydratase (EC 4.2.1.46)
contig_24	fig|6666666.28487.peg.1788	Protein	57310	58005	+	PROBABLE CONSERVED LIPOPROTEIN LPQN
contig_24	fig|6666666.28487.peg.1789	Protein	58858	57995	-	Coenzyme F420-dependent N5,N10-methylene tetrahydromethanopterin reductase and related flavin-dependent oxidoreductases
contig_24	fig|6666666.28487.peg.1790	Protein	60494	59034	-	sensor histidine kinase
contig_24	fig|6666666.28487.peg.1791	Protein	62300	60636	-	Thioredoxin reductase (EC 1.8.1.9)
contig_24	fig|6666666.28487.peg.1792	Protein	62283	62606	+	hypothetical protein
contig_24	fig|6666666.28487.peg.1793	Protein	62582	62803	+	Translation initiation factor 1
contig_24	fig|6666666.28487.peg.1794	Protein	62844	62957	+	LSU ribosomal protein L36p
contig_24	fig|6666666.28487.peg.1795	Protein	63146	63520	+	SSU ribosomal protein S13p (S18e)
contig_24	fig|6666666.28487.peg.1796	Protein	63527	63940	+	SSU ribosomal protein S11p (S14e)
contig_24	fig|6666666.28487.peg.1797	Protein	63955	64560	+	SSU ribosomal protein S4p (S9e)
contig_24	fig|6666666.28487.peg.1798	Protein	64642	65694	+	DNA-directed RNA polymerase alpha subunit (EC 2.7.7.6)
contig_24	fig|6666666.28487.peg.1799	Protein	65768	66379	+	LSU ribosomal protein L17p
contig_24	fig|6666666.28487.peg.1800	Protein	66387	67277	+	tRNA pseudouridine synthase A (EC 4.2.1.70)
contig_24	fig|6666666.28487.peg.1801	Protein	67933	67274	-	serine esterase, cutinase family
contig_24	fig|6666666.28487.peg.1802	Protein	68639	67962	-	Cutinase

contig_24	fig|6666666.28487.peg.1803	Protein	69326	68664	-	serine esterase, cutinase family
contig_24	fig|6666666.28487.peg.1804	Protein	70371	69346	-	Permease of the drug/metabolite transporter (DMT) superfamily
contig_24	fig|6666666.28487.peg.1805	Protein	70451	71011	+	FIG00820767: hypothetical protein
contig_24	fig|6666666.28487.peg.1806	Protein	71069	72385	+	PROBABLE CONSERVED MEMBRANE PROTEIN
contig_24	fig|6666666.28487.peg.1807	Protein	74974	73664	-	hypothetical protein
contig_24	fig|6666666.28487.peg.1808	Protein	75087	78491	+	FtsK/SpoIIIE family protein
contig_24	fig|6666666.28487.peg.1809	Protein	78599	79606	+	conserved hypothetical alanine and valine rich protein
contig_24	fig|6666666.28487.peg.1810	Protein	79698	80030	+	ESAT-6 like protein EsxU
contig_24	fig|6666666.28487.peg.1811	Protein	80040	80336	+	probable secreted protein
contig_24	fig|6666666.28487.peg.1812	Protein	80492	81796	+	GAF domain-containing protein / Signal transduction response regulator
contig_24	fig|6666666.28487.peg.1813	Protein	81882	82799	+	Lysophospholipase (EC 3.1.1.5)
contig_24	fig|6666666.28487.peg.1814	Protein	83319	82771	-	Transcriptional regulator, TetR family
contig_24	fig|6666666.28487.peg.1815	Protein	83468	84991	+	Aldehyde dehydrogenase (EC 1.2.1.3)
contig_24	fig|6666666.28487.peg.1816	Protein	85469	85047	-	hypothetical protein
contig_24	fig|6666666.28487.peg.1817	Protein	85757	86200	+	LSU ribosomal protein L13p (L13Ae)
contig_24	fig|6666666.28487.peg.1818	Protein	86197	86712	+	SSU ribosomal protein S9p (S16e)
contig_24	fig|6666666.28487.peg.1819	Protein	87027	87527	+	FIG00821690: hypothetical protein
contig_24	fig|6666666.28487.peg.1820	Protein	87658	88995	+	Phosphoglucosamine mutase (EC 5.4.2.10)

contig_24	fig|6666666.28487.peg.1821	Protein	89049	89339	+	hypothetical protein
contig_24	fig|6666666.28487.peg.1822	Protein	89432	90763	+	conserved hypothetical alanine and proline rich protein
contig_24	fig|6666666.28487.peg.1823	Protein	90809	91399	+	FIG00820385: hypothetical protein
contig_24	fig|6666666.28487.peg.1824	Protein	92516	91374	-	acyl-CoA dehydrogenase domain protein
contig_24	fig|6666666.28487.peg.1825	Protein	92560	93135	+	Transcriptional regulator, TetR family
contig_24	fig|6666666.28487.peg.1826	Protein	93187	94362	+	Geranylgeranyl reductase (EC 1.3.1.83)
contig_24	fig|6666666.28487.peg.1827	Protein	94842	94420	-	hypothetical protein
contig_24	fig|6666666.28487.peg.1828	Protein	95891	94839	-	N5,N10-methylenetetrahydromethanopterin reductase-related protein
contig_24	fig|6666666.28487.peg.1829	Protein	96795	95950	-	FIG00996653: hypothetical protein
contig_24	fig|6666666.28487.peg.1830	Protein	96851	98716	+	Glucosamine--fructose-6-phosphate aminotransferase [isomerizing] (EC 2.6.1.16)
contig_24	fig|6666666.28487.peg.1831	Protein	98716	99606	+	carboxylesterase
contig_24	fig|6666666.28487.peg.1832	Protein	99828	100130	+	anti-sigma-factor antagonist
contig_24	fig|6666666.28487.peg.1833	Protein	100496	100131	-	hypothetical protein
contig_24	fig|6666666.28487.peg.1834	Protein	100528	101943	+	NAD(P)HX epimerase / NAD(P)HX dehydratase
contig_24	fig|6666666.28487.peg.1835	Protein	101954	103348	+	Glutamate decarboxylase (EC 4.1.1.15)
contig_24	fig|6666666.28487.peg.1836	Protein	103375	104508	+	Alanine racemase (EC 5.1.1.1)
contig_24	fig|6666666.28487.peg.1837	Protein	104505	105596	+	Hydrolase (HAD superfamily)
contig_24	fig|6666666.28487.peg.1838	Protein	105589	106071	+	ATPase YjeE, predicted to have essential role in cell wall biosynthesis

contig_24	fig|6666666.28487.peg.1839	Protein	106068	106715	+	Inactive homolog of metal-dependent proteases, putative molecular chaperone
contig_24	fig|6666666.28487.peg.1840	Protein	106712	107173	+	Ribosomal-protein-S18p-alanine acetyltransferase (EC 2.3.1.-)
contig_24	fig|6666666.28487.peg.1841	Protein	107170	108192	+	YgjD/Kae1/Qri7 family, required for threonylcarbamoyladenosine (t(6)A) formation in tRNA
contig_24	fig|6666666.28487.peg.1842	Protein	109733	108189	-	13E12 repeat family protein
contig_24	fig|6666666.28487.peg.1843	Protein	109958	110260	+	Heat shock protein 60 family co-chaperone GroES
contig_24	fig|6666666.28487.peg.1844	Protein	110349	111980	+	Heat shock protein 60 family chaperone GroEL
contig_24	fig|6666666.28487.peg.1845	Protein	112383	112712	+	hypothetical protein
contig_24	fig|6666666.28487.peg.1846	Protein	113728	113276	-	hypothetical protein
contig_24	fig|6666666.28487.peg.1847	Protein	113828	114181	+	FIG00832246: hypothetical protein
contig_24	fig|6666666.28487.peg.1848	Protein	114407	115477	+	2-keto-3-deoxy-D-arabino-heptulosonate-7-phosphate synthase I alpha (EC 2.5.1.54)
contig_24	fig|6666666.28487.peg.1849	Protein	115789	115445	-	hypothetical protein
contig_24	fig|6666666.28487.peg.1850	Protein	116484	115786	-	Cobalt-containing nitrile hydratase subunit alpha (EC 4.2.1.84)
contig_24	fig|6666666.28487.peg.1851	Protein	117425	116529	-	Thiocyanate hydrolase subunit beta (EC 3.5.5.8)
contig_24	fig|6666666.28487.peg.1852	Protein	117609	118517	+	FIG00827626: hypothetical protein
contig_24	fig|6666666.28487.peg.1853	Protein	118648	118529	-	Sporulation regulatory protein WhiD
contig_24	fig|6666666.28487.peg.1854	Protein	119078	118941	-	hypothetical protein

contig_24	fig|6666666.28487.peg.1855	Protein	119241	120011	+	FIG00996157: hypothetical protein
contig_24	fig|6666666.28487.peg.1856	Protein	120109	120729	+	RNA polymerase sigma-70 factor
contig_24	fig|6666666.28487.peg.1857	Protein	120722	121906	+	FIG00997706: hypothetical protein
contig_24	fig|6666666.28487.peg.1858	Protein	122333	121929	-	hypothetical protein
contig_24	fig|6666666.28487.peg.1859	Protein	122458	124011	+	Inosine-5'-monophosphate dehydrogenase (EC 1.1.1.205)
contig_24	fig|6666666.28487.peg.1860	Protein	124043	125179	+	Inosine-5'-monophosphate dehydrogenase (EC 1.1.1.205)
contig_24	fig|6666666.28487.peg.1861	Protein	125773	125183	-	Transcriptional regulator, TetR family
contig_24	fig|6666666.28487.peg.1862	Protein	125860	127596	+	Cholesterol oxidase (EC 1.1.3.6)
contig_24	fig|6666666.28487.peg.1863	Protein	129694	127580	-	Two-component system, sensor protein
contig_24	fig|6666666.28487.peg.1864	Protein	130013	130663	+	Two-component system, regulatory protein
contig_24	fig|6666666.28487.peg.1865	Protein	130879	132414	+	GMP synthase [glutamine-hydrolyzing] (EC 6.3.5.2)
contig_24	fig|6666666.28487.peg.1866	Protein	132548	133468	+	putative esterase
contig_24	fig|6666666.28487.peg.1867	Protein	134042	133479	-	Thiaminase II (EC 3.5.99.2)
contig_24	fig|6666666.28487.peg.1868	Protein	134885	134058	-	Probable transcription regulator protein
contig_24	fig|6666666.28487.peg.1869	Protein	136471	134942	-	Cyclohexanone monooxygenase (EC 1.14.13.22)
contig_24	fig|6666666.28487.peg.1870	Protein	137395	136475	-	FIG00830796: hypothetical protein
contig_24	fig|6666666.28487.peg.1871	Protein	138224	137406	-	Oxidoreductase, short chain dehydrogenase/reductase family
contig_24	fig|6666666.28487.peg.1872	Protein	139247	138228	-	Acetyl hydrolase MbtJ
contig_24	fig|6666666.28487.peg.1873	Protein	139426	140124	+	FIG00821108: hypothetical protein

contig_24	fig|6666666.28487.peg.1874	Protein	140156	141688	+	DNA polymerase-like protein PA0670
contig_24	fig|6666666.28487.peg.1875	Protein	142713	141679	-	Inosine-uridine preferring nucleoside hydrolase (EC 3.2.2.1)
contig_24	fig|6666666.28487.peg.1876	Protein	144707	142710	-	Oxidoreductase, short-chain dehydrogenase/reductase family
contig_24	fig|6666666.28487.peg.1877	Protein	144777	145643	+	Enoyl-CoA hydratase
contig_24	fig|6666666.28487.peg.1878	Protein	145774	146940	+	hypothetical protein
contig_24	fig|6666666.28487.peg.1879	Protein	147017	150271	+	DNA polymerase III alpha subunit (EC 2.7.7.7)
contig_24	fig|6666666.28487.peg.1880	Protein	150410	151069	+	putative oxidoreductase
contig_25	fig|6666666.28487.peg.1881	Protein	2306	1518	-	Tryptophan synthase alpha chain (EC 4.2.1.20)
contig_25	fig|6666666.28487.peg.1882	Protein	3565	2303	-	Tryptophan synthase beta chain (EC 4.2.1.20)
contig_25	fig|6666666.28487.peg.1883	Protein	4384	3566	-	Indole-3-glycerol phosphate synthase (EC 4.1.1.48)
contig_25	fig|6666666.28487.peg.1884	Protein	5050	4457	-	FIG00997095: hypothetical protein
contig_25	fig|6666666.28487.peg.1885	Protein	6573	5047	-	Anthranilate synthase, aminase component (EC 4.1.3.27)
contig_25	fig|6666666.28487.peg.1886	Protein	6606	7070	+	Alkyl hydroperoxide reductase subunit C-like protein
contig_25	fig|6666666.28487.peg.1887	Protein	7067	7861	+	ABC transporter, ATP-binding protein
contig_25	fig|6666666.28487.peg.1888	Protein	8205	7858	-	Phosphoribosyl-AMP cyclohydrolase (EC 3.5.4.19)
contig_25	fig|6666666.28487.peg.1889	Protein	8987	8202	-	Imidazole glycerol phosphate synthase cyclase subunit (EC 4.1.3.-)
contig_25	fig|6666666.28487.peg.1890	Protein	9802	8984	-	Histidinol-phosphatase [alternative form] (EC 3.1.3.15)
contig_25	fig|6666666.28487.peg.1891	Protein	10542	9799	-	Phosphoribosylformimino-5-aminoimidazole carboxamide ribotide isomerase (EC 5.3.1.16) / Acting phosphoribosylanthranilate isomerase (EC 5.3.1.24)

contig_25	fig|6666666.28487.peg.1892	Protein	11168	10548	-	Imidazole glycerol phosphate synthase amidotransferase subunit (EC 2.4.2.-)
contig_25	fig|6666666.28487.peg.1893	Protein	11755	11165	-	Imidazoleglycerol-phosphate dehydratase (EC 4.2.1.19)
contig_25	fig|6666666.28487.peg.1894	Protein	12894	11779	-	Histidinol-phosphate aminotransferase (EC 2.6.1.9)
contig_25	fig|6666666.28487.peg.1895	Protein	14222	12891	-	Histidinol dehydrogenase (EC 1.1.1.23)
contig_25	fig|6666666.28487.peg.1896	Protein	14268	14690	+	FIG00820654: hypothetical protein
contig_25	fig|6666666.28487.peg.1897	Protein	14714	15352	+	transporter, LysE family
contig_25	fig|6666666.28487.peg.1898	Protein	15829	15380	-	FIG00825470: hypothetical protein
contig_25	fig|6666666.28487.peg.1899	Protein	16690	15833	-	Quinolinate phosphoribosyltransferase [decarboxylating] (EC 2.4.2.19)
contig_25	fig|6666666.28487.peg.1900	Protein	18258	16687	-	L-aspartate oxidase (EC 1.4.3.16)
contig_25	fig|6666666.28487.peg.1901	Protein	19301	18255	-	Quinolinate synthetase (EC 2.5.1.72)
contig_25	fig|6666666.28487.peg.1902	Protein	19360	20037	+	Nudix-related transcriptional regulator NrtR
contig_25	fig|6666666.28487.peg.1903	Protein	20080	21435	+	Lipase 1 (EC 3.1.1.3)
contig_25	fig|6666666.28487.peg.1904	Protein	22275	22024	-	FIG00820826: hypothetical protein
contig_25	fig|6666666.28487.peg.1905	Protein	23295	22300	-	Biotin synthase (EC 2.8.1.6)
contig_25	fig|6666666.28487.peg.1906	Protein	23341	23943	+	Predicted biotin repressor from TetR family
contig_25	fig|6666666.28487.peg.1907	Protein	24460	23936	-	FIG00820026: hypothetical protein
contig_25	fig|6666666.28487.peg.1908	Protein	25140	24460	-	Dethiobiotin synthetase (EC 6.3.3.3)
contig_25	fig|6666666.28487.peg.1909	Protein	26279	25137	-	8-amino-7-oxononanoate synthase (EC 2.3.1.47)
contig_25	fig|6666666.28487.peg.1910	Protein	27611	26316	-	Adenosylmethionine-8-amino-7-oxononanoate

						aminotransferase (EC 2.6.1.62)
contig_25	fig|6666666.28487.peg.1911	Protein	27725	29836	+	O-antigen acetylase
contig_25	fig|6666666.28487.peg.1912	Protein	29836	31980	+	Glycogen debranching enzyme (EC 3.2.1.-)
contig_25	fig|6666666.28487.peg.1913	Protein	31982	34276	+	Malto-oligosyltrehalose synthase (EC 5.4.99.15)
contig_25	fig|6666666.28487.peg.1914	Protein	34269	36002	+	Malto-oligosyltrehalose trehalohydrolase (EC 3.2.1.141)
contig_25	fig|6666666.28487.peg.1915	Protein	36071	36499	+	hypothetical protein
contig_25	fig|6666666.28487.peg.1916	Protein	37794	36505	-	Threonine dehydratase (EC 4.3.1.19)
contig_25	fig|6666666.28487.peg.1917	Protein	37931	38161	+	hypothetical protein
contig_25	fig|6666666.28487.peg.1918	Protein	41713	38180	-	DNA polymerase III alpha subunit (EC 2.7.7.7)
contig_25	fig|6666666.28487.peg.1919	Protein	42474	41800	-	Transcriptional regulator, TetR family
contig_25	fig|6666666.28487.peg.1920	Protein	43418	42528	-	Protein rarD
contig_25	fig|6666666.28487.peg.1921	Protein	44329	43415	-	Ribosomal large subunit pseudouridine synthase D (EC 4.2.1.70)
contig_25	fig|6666666.28487.peg.1922	Protein	44931	44341	-	Lipoprotein signal peptidase (EC 3.4.23.36)
contig_25	fig|6666666.28487.peg.1923	Protein	44974	45876	+	L-asparaginase (EC 3.5.1.1)
contig_25	fig|6666666.28487.peg.1924	Protein	47236	45878	-	DNA polymerase IV (EC 2.7.7.7)
contig_25	fig|6666666.28487.peg.1925	Protein	47275	47508	+	FIG002958: hypothetical protein
contig_25	fig|6666666.28487.peg.1926	Protein	50641	47498	-	Isoleucyl-tRNA synthetase (EC 6.1.1.5)
contig_25	fig|6666666.28487.peg.1927	Protein	50642	50767	+	hypothetical protein
contig_25	fig|6666666.28487.peg.1928	Protein	51501	50893	-	Phenolpthiocerol synthesis type-I polyketide synthase PpsE

contig_25	fig|6666666.28487.peg.1929	Protein	51637	52011	+	hypothetical protein
contig_25	fig|6666666.28487.peg.1930	Protein	52065	52799	+	FIG00827374: hypothetical protein
contig_25	fig|6666666.28487.peg.1931	Protein	54114	52861	-	Esterase LipL
contig_25	fig|6666666.28487.peg.1932	Protein	55155	54166	-	putative periplasmic protein kinase ArgK and related GTPases of G3E family
contig_25	fig|6666666.28487.peg.1933	Protein	57400	55142	-	Methylmalonyl-CoA mutase (EC 5.4.99.2)
contig_25	fig|6666666.28487.peg.1934	Protein	59264	57402	-	Methylmalonyl-CoA mutase, small subunit (EC 5.4.99.2)
contig_25	fig|6666666.28487.peg.1935	Protein	59370	60077	+	DedA family protein paralog
contig_25	fig|6666666.28487.peg.1936	Protein	60426	60064	-	FIG00822897: hypothetical protein
contig_25	fig|6666666.28487.peg.1937	Protein	61646	60423	-	Putative stomatin/prohibitin-family membrane protease subunit YbbK
contig_25	fig|6666666.28487.peg.1938	Protein	62096	61662	-	Putative activity regulator of membrane protease YbbK
contig_25	fig|6666666.28487.peg.1939	Protein	62183	63025	+	FIG00821059: hypothetical protein
contig_25	fig|6666666.28487.peg.1940	Protein	64053	63022	-	Ferrochelatase, protoheme ferro-lyase (EC 4.99.1.1)
contig_25	fig|6666666.28487.peg.1941	Protein	64863	64054	-	Enoyl-[acyl-carrier-protein] reductase [NADH] (EC 1.3.1.9)
contig_25	fig|6666666.28487.peg.1942	Protein	65607	64885	-	3-oxoacyl-[acyl-carrier protein] reductase (EC 1.1.1.100)
contig_25	fig|6666666.28487.peg.1943	Protein	66643	65660	-	Possible membrane protein
contig_25	fig|6666666.28487.peg.1944	Protein	67600	66695	-	Possible membrane protein
contig_25	fig|6666666.28487.peg.1945	Protein	68779	67640	-	COG0714: MoxR-like ATPases
contig_25	fig|6666666.28487.peg.1946	Protein	69637	68936	-	Invasion protein

contig_25	fig|6666666.28487.peg.1947	Protein	71049	69634	-	NLP/P60 family protein
contig_25	fig|6666666.28487.peg.1948	Protein	71862	71269	-	hypothetical protein
contig_25	fig|6666666.28487.peg.1949	Protein	72027	74843	+	Aconitate hydratase (EC 4.2.1.3) @ 2-methylisocitrate dehydratase (EC 4.2.1.99)
contig_25	fig|6666666.28487.peg.1950	Protein	74853	75425	+	Transcriptional regulator, TetR family
contig_25	fig|6666666.28487.peg.1951	Protein	75626	75432	-	POSSIBLE TRANSCRIPTIONAL REGULATORY PROTEIN
contig_25	fig|6666666.28487.peg.1952	Protein	77355	75718	-	ABC transporter ATP-binding protein
contig_25	fig|6666666.28487.peg.1953	Protein	77501	79183	+	Long-chain-fatty-acid--CoA ligase (EC 6.2.1.3)
contig_25	fig|6666666.28487.peg.1954	Protein	79190	79909	+	glycosyl transferase, family 2
contig_25	fig|6666666.28487.peg.1955	Protein	79891	80838	+	daunorubicin resistance ABC transporter ATP-binding subunit
contig_25	fig|6666666.28487.peg.1956	Protein	80835	81575	+	ABC-2 type transporter
contig_25	fig|6666666.28487.peg.1957	Protein	81572	82351	+	ABC-2 type transporter
contig_25	fig|6666666.28487.peg.1958	Protein	82590	82315	-	protein of unknown function DUF222
contig_26	fig|6666666.28487.peg.1959	Protein	434	1984	+	FIG00814964: hypothetical protein
contig_26	fig|6666666.28487.peg.1960	Protein	2032	2589	+	FIG00823891: hypothetical protein
contig_26	fig|6666666.28487.peg.1961	Protein	3269	2592	-	Predicted cobalt transporter CbtC
contig_26	fig|6666666.28487.peg.1962	Protein	3588	3304	-	Putative metal chaperone, involved in Zn homeostasis, GTPase of COG0523 family
contig_26	fig|6666666.28487.peg.1963	Protein	4205	3585	-	Cobalt-containing nitrile hydratase subunit alpha (EC 4.2.1.84)
contig_26	fig|6666666.28487.peg.1964	Protein	4936	4202	-	Cobalt-containing nitrile hydratase subunit beta (EC 4.2.1.84)

contig_26	fig|6666666.28487.peg.1965	Protein	5457	4933	-	FIG00828737: hypothetical protein
contig_26	fig|6666666.28487.peg.1966	Protein	5471	5611	+	hypothetical protein
contig_26	fig|6666666.28487.peg.1967	Protein	7616	6582	-	Transcriptional regulator, AraC family
contig_26	fig|6666666.28487.peg.1968	Protein	8857	7706	-	transport protein
contig_26	fig|6666666.28487.peg.1969	Protein	8910	10091	+	hypothetical protein
contig_26	fig|6666666.28487.peg.1970	Protein	10150	10263	+	hypothetical protein
contig_26	fig|6666666.28487.peg.1971	Protein	10307	11506	+	FIG00821604: hypothetical protein
contig_26	fig|6666666.28487.peg.1972	Protein	11547	13535	+	4-alpha-glucanotransferase (amylomaltase) (EC 2.4.1.25)
contig_26	fig|6666666.28487.peg.1973	Protein	13591	13860	+	hypothetical protein
contig_26	fig|6666666.28487.peg.1974	Protein	15127	13862	-	Ammonium transporter
contig_26	fig|6666666.28487.peg.1975	Protein	16023	15208	-	hypothetical protein
contig_26	fig|6666666.28487.peg.1976	Protein	16463	16032	-	hypothetical protein
contig_26	fig|6666666.28487.peg.1977	Protein	16562	17161	+	Transcriptional regulator, TetR family
contig_26	fig|6666666.28487.peg.1978	Protein	17942	17151	-	blr6059; putative cyclase
contig_26	fig|6666666.28487.peg.1979	Protein	17995	19185	+	putative monooxygenase
contig_26	fig|6666666.28487.peg.1980	Protein	19393	19190	-	hypothetical protein
contig_26	fig|6666666.28487.peg.1981	Protein	19507	20643	+	Ribonuclease BN (EC 3.1.-.-)
contig_26	fig|6666666.28487.peg.1982	Protein	21644	20655	-	Endoglucanase A precursor (EC 3.2.1.4)
contig_26	fig|6666666.28487.peg.1983	Protein	22311	21691	-	CheB methylesterase(EC:3.1.1.61)

contig_26	fig|6666666.28487.peg.1984	Protein	23161	22313	-	Chemotaxis protein methyltransferase CheR (EC 2.1.1.80)
contig_26	fig|6666666.28487.peg.1985	Protein	26651	23166	-	hypothetical protein
contig_26	fig|6666666.28487.peg.1986	Protein	29258	26658	-	hypothetical protein
contig_26	fig|6666666.28487.peg.1987	Protein	29364	29816	+	FIG00823190: hypothetical protein
contig_26	fig|6666666.28487.peg.1988	Protein	29831	30253	+	FIG00821490: hypothetical protein
contig_26	fig|6666666.28487.peg.1989	Protein	30294	31325	+	MaoC domain protein dehydratase
contig_26	fig|6666666.28487.peg.1990	Protein	31332	32561	+	hypothetical protein
contig_26	fig|6666666.28487.peg.1991	Protein	33512	32514	-	Ribose operon repressor
contig_26	fig|6666666.28487.peg.1992	Protein	35771	33660	-	Isoquinoline 1-oxidoreductase beta subunit (EC 1.3.99.16)
contig_26	fig|6666666.28487.peg.1993	Protein	36238	35768	-	Isoquinoline 1-oxidoreductase alpha subunit (EC 1.3.99.16)
contig_26	fig|6666666.28487.peg.1994	Protein	36884	36240	-	hypothetical protein
contig_26	fig|6666666.28487.peg.1995	Protein	37888	36950	-	LysR-family protein transcriptional regulator
contig_26	fig|6666666.28487.peg.1996	Protein	38011	38724	+	3-oxoacyl-[acyl-carrier protein] reductase (EC 1.1.1.100)
contig_26	fig|6666666.28487.peg.1997	Protein	38726	39085	+	hypothetical protein
contig_26	fig|6666666.28487.peg.1998	Protein	39330	39094	-	FIG00821671: hypothetical protein
contig_26	fig|6666666.28487.peg.1999	Protein	39628	41661	+	Topoisomerase IV subunit B (EC 5.99.1.-)
contig_26	fig|6666666.28487.peg.2000	Protein	41671	43815	+	Topoisomerase IV subunit A (EC 5.99.1.-)
contig_26	fig|6666666.28487.peg.2001	Protein	44047	43826	-	hypothetical protein
contig_26	fig|6666666.28487.peg.2002	Protein	44071	45492	+	Beta-lactamase class C and other penicillin binding proteins

contig_26	fig|6666666.28487.peg.2003	Protein	48458	45489	-	COG3866 Pectate lyase
contig_26	fig|6666666.28487.peg.2004	Protein	49402	48689	-	Transcriptional regulator, GntR family
contig_26	fig|6666666.28487.peg.2005	Protein	49530	50021	+	Gluconokinase (EC 2.7.1.12)
contig_26	fig|6666666.28487.peg.2006	Protein	50058	51479	+	Low-affinity gluconate/H+ symporter GntU
contig_26	fig|6666666.28487.peg.2007	Protein	51543	52310	+	Putative secreted protein
contig_26	fig|6666666.28487.peg.2008	Protein	52345	53013	+	(AJ250023) putative polyketide synthase
contig_26	fig|6666666.28487.peg.2009	Protein	54080	53010	-	NLP/P60 family protein
contig_26	fig|6666666.28487.peg.2010	Protein	54365	54222	-	hypothetical protein
contig_26	fig|6666666.28487.peg.2011	Protein	54276	55181	+	Putative lipoprotein lppW precursor
contig_26	fig|6666666.28487.peg.2012	Protein	55374	56717	+	hypothetical protein
contig_26	fig|6666666.28487.peg.2013	Protein	56728	56928	+	hypothetical protein
contig_26	fig|6666666.28487.peg.2014	Protein	57193	58254	+	hypothetical protein
contig_26	fig|6666666.28487.peg.2015	Protein	58288	58776	+	hypothetical protein
contig_26	fig|6666666.28487.peg.2016	Protein	58791	59408	+	conserved hypothetical protein
contig_26	fig|6666666.28487.peg.2017	Protein	60333	59419	-	Aldo-keto reductase
contig_26	fig|6666666.28487.peg.2018	Protein	60540	61310	+	phospholipid/glycerol acyltransferase
contig_26	fig|6666666.28487.peg.2019	Protein	61921	61322	-	COG1309: Transcriptional regulator
contig_26	fig|6666666.28487.peg.2020	Protein	62036	62962	+	hypothetical protein
contig_26	fig|6666666.28487.peg.2021	Protein	64158	62968	-	putative cytochrome P450

contig_26	fig|6666666.28487.peg.2022	Protein	65746	64163	-	3-(3-hydroxy-phenyl)propionate hydroxylase (EC 1.14.13.-)
contig_26	fig|6666666.28487.peg.2023	Protein	67629	65743	-	Long-chain-fatty-acid--CoA ligase (EC 6.2.1.3)
contig_26	fig|6666666.28487.peg.2024	Protein	68564	67626	-	Fumarylacetoacetate hydrolase family protein
contig_26	fig|6666666.28487.peg.2025	Protein	69703	68561	-	2,3-dihydroxybiphenyl 1,2-dioxygenase
contig_26	fig|6666666.28487.peg.2026	Protein	69815	70519	+	Transcriptional regulator, TetR family
contig_26	fig|6666666.28487.peg.2027	Protein	71577	70639	-	dienelactone hydrolase domain protein
contig_26	fig|6666666.28487.peg.2028	Protein	71894	71673	-	hypothetical protein
contig_26	fig|6666666.28487.peg.2029	Protein	72236	72364	+	hypothetical protein
contig_26	fig|6666666.28487.peg.2030	Protein	72448	74025	+	hypothetical protein
contig_26	fig|6666666.28487.peg.2031	Protein	74453	74037	-	pyridoxamine 5'-phosphate oxidase-related, FMN-binding
contig_26	fig|6666666.28487.peg.2032	Protein	75286	74450	-	FIG01126584: hypothetical protein
contig_26	fig|6666666.28487.peg.2033	Protein	75367	76299	+	Transcriptional regulator, AraC family
contig_26	fig|6666666.28487.peg.2034	Protein	76738	76328	-	Endoribonuclease L-PSP
contig_26	fig|6666666.28487.peg.2035	Protein	77306	76866	-	integral membrane protein
contig_26	fig|6666666.28487.peg.2036	Protein	77873	77367	-	hypothetical protein
contig_26	fig|6666666.28487.peg.2037	Protein	79684	77885	-	hypothetical protein
contig_26	fig|6666666.28487.peg.2038	Protein	79760	80329	+	Transcriptional regulator, TetR family
contig_26	fig|6666666.28487.peg.2039	Protein	80942	80370	-	Alcohol dehydrogenase, zinc-binding domain protein
contig_26	fig|6666666.28487.peg.2040	Protein	81379	80978	-	Sorbitol dehydrogenase (EC 1.1.1.14)

contig_26	fig|6666666.28487.peg.2041	Protein	81710	81396	-	L-arabinose isomerase (EC 5.3.1.4)
contig_26	fig|6666666.28487.peg.2042	Protein	81894	82823	+	putative aldose-1-epimerase
contig_26	fig|6666666.28487.peg.2043	Protein	84119	82833	-	major facilitator family transporter
contig_26	fig|6666666.28487.peg.2044	Protein	86422	84179	-	Beta-glucosidase (EC 3.2.1.21)
contig_26	fig|6666666.28487.peg.2045	Protein	87486	86545	-	Transcriptional regulator, AraC family
contig_26	fig|6666666.28487.peg.2046	Protein	87578	88432	+	putative oxidoreductase
contig_26	fig|6666666.28487.peg.2047	Protein	88411	88605	+	putative oxidoreductase
contig_26	fig|6666666.28487.peg.2048	Protein	88703	89254	+	YaeQ protein
contig_26	fig|6666666.28487.peg.2049	Protein	90883	90056	-	Transcriptional regulator, MerR family
contig_26	fig|6666666.28487.peg.2050	Protein	91032	91382	+	FIG172065: hypothetical protein
contig_26	fig|6666666.28487.peg.2051	Protein	91379	92218	+	Short-chain dehydrogenase/reductase SDR precursor
contig_26	fig|6666666.28487.peg.2052	Protein	92364	93092	+	hypothetical protein
contig_26	fig|6666666.28487.peg.2053	Protein	93630	93115	-	integral membrane protein
contig_26	fig|6666666.28487.peg.2054	Protein	93915	93793	-	hypothetical protein
contig_26	fig|6666666.28487.peg.2055	Protein	93915	94682	+	oxidoreductase, aldo-keto reductase family
contig_26	fig|6666666.28487.peg.2056	Protein	96108	94693	-	PF00070 family, FAD-dependent NAD(P)-disulphide oxidoreductase
contig_26	fig|6666666.28487.peg.2057	Protein	96782	96144	-	Transcriptional regulator, TetR family
contig_26	fig|6666666.28487.peg.2058	Protein	96903	98069	+	PROBABLE CONSERVED TRANSMEMBRANE PROTEIN
contig_26	fig|6666666.28487.peg.2059	Protein	98066	98524	+	hypothetical protein

contig_26	fig|6666666.28487.peg.2060	Protein	99492	98542	-	Nitrilase (EC 3.5.5.7)
contig_26	fig|6666666.28487.peg.2061	Protein	100741	99506	-	Histidinol dehydrogenase (EC 1.1.1.23)
contig_26	fig|6666666.28487.peg.2062	Protein	100790	100936	+	hypothetical protein
contig_26	fig|6666666.28487.peg.2063	Protein	102493	101003	-	amino acid permease-associated region
contig_26	fig|6666666.28487.peg.2064	Protein	102597	103520	+	transcriptional regulator, LysR family
contig_26	fig|6666666.28487.peg.2065	Protein	103540	103674	+	hypothetical protein
contig_26	fig|6666666.28487.peg.2066	Protein	104085	103696	-	hypothetical protein
contig_26	fig|6666666.28487.peg.2067	Protein	104327	104082	-	hypothetical protein
contig_26	fig|6666666.28487.peg.2068	Protein	105389	104370	-	epoxide hydrolase
contig_26	fig|6666666.28487.peg.2069	Protein	106600	105383	-	hypothetical protein
contig_26	fig|6666666.28487.peg.2070	Protein	106740	107255	+	Transcriptional regulator, TetR family
contig_26	fig|6666666.28487.peg.2071	Protein	107395	107709	+	hypothetical protein
contig_26	fig|6666666.28487.peg.2072	Protein	107910	107716	-	hypothetical protein
contig_26	fig|6666666.28487.peg.2073	Protein	108020	108388	+	hypothetical protein
contig_26	fig|6666666.28487.peg.2074	Protein	108606	109016	+	Mobile element protein
contig_26	fig|6666666.28487.peg.2075	Protein	111012	109054	-	FIG00826512: hypothetical protein
contig_26	fig|6666666.28487.peg.2076	Protein	111728	111012	-	FIG00830223: hypothetical protein
contig_26	fig|6666666.28487.peg.2077	Protein	111869	112840	+	hypothetical protein
contig_26	fig|6666666.28487.peg.2078	Protein	112929	113603	+	TetR-family transcriptional regulator
contig_26	fig|6666666.28487.peg.2079	Protein	113638	114501	+	3-hydroxybutyryl-CoA dehydrogenase (EC 1.1.1.157); 3-

						hydroxyacyl-CoA dehydrogenase (EC 1.1.1.35)
contig_26	fig|6666666.28487.peg.2080	Protein	114528	116300	+	putative 67 kDa myosin-crossreactive streptococcal antigen
contig_27	fig|6666666.28487.peg.2081	Protein	1271	594	-	cAMP-binding proteins - catabolite gene activator and regulatory subunit of cAMP-dependent protein kinases
contig_27	fig|6666666.28487.peg.2082	Protein	1511	1356	-	hypothetical protein
contig_27	fig|6666666.28487.peg.2083	Protein	2288	1731	-	hypothetical protein
contig_27	fig|6666666.28487.peg.2084	Protein	2336	3109	+	probable secreted protein
contig_27	fig|6666666.28487.peg.2085	Protein	4346	3102	-	FIG00831221: hypothetical protein
contig_27	fig|6666666.28487.peg.2086	Protein	4375	4950	+	Transcriptional regulator, TetR family
contig_27	fig|6666666.28487.peg.2087	Protein	4947	6053	+	FIG00821768: hypothetical protein
contig_27	fig|6666666.28487.peg.2088	Protein	6277	7161	+	Universal stress protein family
contig_27	fig|6666666.28487.peg.2089	Protein	8318	7176	-	Acyl-CoA dehydrogenase family protein
contig_27	fig|6666666.28487.peg.2090	Protein	9551	8319	-	Butyryl-CoA dehydrogenase (EC 1.3.99.2)
contig_27	fig|6666666.28487.peg.2091	Protein	9893	10738	+	FIG00823491: hypothetical protein
contig_27	fig|6666666.28487.peg.2092	Protein	12557	10719	-	Sensory box/GGDEF family protein
contig_27	fig|6666666.28487.peg.2093	Protein	14685	12574	-	POSSIBLE MOLYBDOPTERIN BIOSYNTHESIS PROTEIN MOEY
contig_27	fig|6666666.28487.peg.2094	Protein	15434	14682	-	FIG00822292: hypothetical protein
contig_27	fig|6666666.28487.peg.2095	Protein	16225	16677	+	Acyl dehydratase
contig_27	fig|6666666.28487.peg.2096	Protein	16767	18104	+	Cyclohexanone monooxygenase (EC 1.14.13.22)

contig_27	fig|6666666.28487.peg.2097	Protein	18101	18994	+	Hydrolases of the alpha/beta superfamily
contig_27	fig|6666666.28487.peg.2098	Protein	19809	18988	-	short-chain dehydrogenase/reductase SDR
contig_27	fig|6666666.28487.peg.2099	Protein	21013	19838	-	Butyryl-CoA dehydrogenase (EC 1.3.99.2)
contig_27	fig|6666666.28487.peg.2100	Protein	21121	21861	+	3-oxoacyl-[acyl-carrier protein] reductase (EC 1.1.1.100)
contig_27	fig|6666666.28487.peg.2101	Protein	21858	23075	+	3-ketoacyl-CoA thiolase (EC 2.3.1.16) @ Acetyl-CoA acetyltransferase (EC 2.3.1.9)
contig_27	fig|6666666.28487.peg.2102	Protein	23072	24265	+	Butyryl-CoA dehydrogenase (EC 1.3.99.2)
contig_27	fig|6666666.28487.peg.2103	Protein	24270	24938	+	Predicted regulator PutR for proline utilization, GntR family
contig_27	fig|6666666.28487.peg.2104	Protein	24938	26122	+	L-carnitine dehydratase/bile acid-inducible protein F
contig_27	fig|6666666.28487.peg.2105	Protein	27619	26111	-	Long-chain-fatty-acid--CoA ligase (EC 6.2.1.3)
contig_27	fig|6666666.28487.peg.2106	Protein	29180	27612	-	Long-chain-fatty-acid--CoA ligase (EC 6.2.1.3)
contig_27	fig|6666666.28487.peg.2107	Protein	30619	29177	-	Long-chain-fatty-acid--CoA ligase (EC 6.2.1.3)
contig_27	fig|6666666.28487.peg.2108	Protein	31410	30619	-	Enoyl-CoA hydratase (EC 4.2.1.17)
contig_27	fig|6666666.28487.peg.2109	Protein	32232	31420	-	Enoyl-CoA hydratase (EC 4.2.1.17)
contig_27	fig|6666666.28487.peg.2110	Protein	33053	32247	-	Enoyl-CoA hydratase (EC 4.2.1.17)
contig_27	fig|6666666.28487.peg.2111	Protein	33162	33941	+	regulatory protein GntR, HTH
contig_27	fig|6666666.28487.peg.2112	Protein	33952	34881	+	Enoyl-CoA hydratase (EC 4.2.1.17)
contig_27	fig|6666666.28487.peg.2113	Protein	34960	36135	+	3-ketoacyl-CoA thiolase (EC 2.3.1.16) @ Acetyl-CoA acetyltransferase (EC 2.3.1.9)
contig_27	fig|6666666.28487.peg.2114	Protein	36969	36217	-	Transcriptional regulator, TetR family

contig_27	fig|6666666.28487.peg.2115	Protein	37190	37951	+	Enoyl-CoA hydratase (EC 4.2.1.17)
contig_27	fig|6666666.28487.peg.2116	Protein	37962	38759	+	3-oxoacyl-[acyl-carrier protein] reductase (EC 1.1.1.100)
contig_27	fig|6666666.28487.peg.2117	Protein	39000	40934	+	Enoyl-CoA hydratase (EC 4.2.1.17)
contig_27	fig|6666666.28487.peg.2118	Protein	40936	42567	+	Long-chain-fatty-acid--CoA ligase (EC 6.2.1.3)
contig_27	fig|6666666.28487.peg.2119	Protein	42564	43397	+	PROBABLE ANTIBIOTIC-RESISTANCE PROTEIN
contig_27	fig|6666666.28487.peg.2120	Protein	43397	44437	+	Butyryl-CoA dehydrogenase (EC 1.3.99.2)
contig_27	fig|6666666.28487.peg.2121	Protein	44441	45613	+	acyl-CoA dehydrogenase domain protein
contig_27	fig|6666666.28487.peg.2122	Protein	46755	45610	-	3-ketoacyl-CoA thiolase (acaB-12)
contig_27	fig|6666666.28487.peg.2123	Protein	47583	46774	-	TesB-like acyl-CoA thioesterase 5
contig_27	fig|6666666.28487.peg.2124	Protein	48057	47683	-	conserved hypothetical protein
contig_27	fig|6666666.28487.peg.2125	Protein	48102	50285	+	oxidoreductase, molybdopterin-binding
contig_27	fig|6666666.28487.peg.2126	Protein	51575	50361	-	L-carnitine dehydratase/bile acid-inducible protein F
contig_27	fig|6666666.28487.peg.2127	Protein	51708	53249	+	Aldehyde dehydrogenase (EC 1.2.1.3)
contig_27	fig|6666666.28487.peg.2128	Protein	54522	53311	-	putative cytochrome P450 hydroxylase
contig_27	fig|6666666.28487.peg.2129	Protein	54769	56214	+	Aldehyde dehydrogenase (EC 1.2.1.3)
contig_27	fig|6666666.28487.peg.2130	Protein	57853	56201	-	Long-chain-fatty-acid--CoA ligase (EC 6.2.1.3)
contig_27	fig|6666666.28487.peg.2131	Protein	58999	57983	-	Terminal oxygenase KshA
contig_27	fig|6666666.28487.peg.2132	Protein	59080	60708	+	Cyclohexanone monooxygenase (EC 1.14.13.22)
contig_27	fig|6666666.28487.peg.2133	Protein	60705	61826	+	FIG00823551: hypothetical protein

contig_27	fig|6666666.28487.peg.2134	Protein	61882	62256	+	FIG00822683: hypothetical protein
contig_27	fig|6666666.28487.peg.2135	Protein	62253	63293	+	N5,N10-methylenetetrahydromethanopterin reductase-related protein
contig_27	fig|6666666.28487.peg.2136	Protein	64848	63352	-	Propionyl-CoA carboxylase beta chain (EC 6.4.1.3)
contig_27	fig|6666666.28487.peg.2137	Protein	64926	65966	+	PROBABLE OXIDOREDUCTASE
contig_27	fig|6666666.28487.peg.2138	Protein	65963	67201	+	putative cytochrome P450 hydroxylase
contig_27	fig|6666666.28487.peg.2139	Protein	68556	67273	-	FIG00821608: hypothetical protein
contig_27	fig|6666666.28487.peg.2140	Protein	68748	68611	-	hypothetical protein
contig_27	fig|6666666.28487.peg.2141	Protein	71054	68748	-	Carbon starvation protein A
contig_27	fig|6666666.28487.peg.2142	Protein	72690	71155	-	ATP-dependent DNA ligase (EC 6.5.1.1)
contig_27	fig|6666666.28487.peg.2143	Protein	74238	72721	-	Sulfur oxidation molybdopterin C protein
contig_27	fig|6666666.28487.peg.2144	Protein	74356	75123	+	3-hydroxyacyl-CoA dehydrogenase type II (EC 1.1.1.35)
contig_27	fig|6666666.28487.peg.2145	Protein	75267	76313	+	probable phosphotransferase
contig_27	fig|6666666.28487.peg.2146	Protein	77083	76328	-	membrane protein, putative
contig_27	fig|6666666.28487.peg.2147	Protein	77317	77772	+	FIG00821186: hypothetical protein
contig_27	fig|6666666.28487.peg.2148	Protein	77993	79438	+	Amino acid permease-associated region
contig_27	fig|6666666.28487.peg.2149	Protein	79852	79475	-	hypothetical protein
contig_27	fig|6666666.28487.peg.2150	Protein	81338	79857	-	Cytochrome P450 136
contig_27	fig|6666666.28487.peg.2151	Protein	81393	81506	+	hypothetical protein
contig_27	fig|6666666.28487.peg.2152	Protein	81466	82086	+	Transcriptional regulator, TetR family

contig_27	fig|6666666.28487.peg.2153	Protein	82385	82083	-	hypothetical protein
contig_27	fig|6666666.28487.peg.2154	Protein	82462	83331	+	Short chain dehydrogenase
contig_27	fig|6666666.28487.peg.2155	Protein	84513	83335	-	FIG00826588: hypothetical protein
contig_27	fig|6666666.28487.peg.2156	Protein	85642	84524	-	DNA polymerase-like protein MT3142
contig_27	fig|6666666.28487.peg.2157	Protein	86214	85606	-	Transcriptional regulator, TetR family
contig_27	fig|6666666.28487.peg.2158	Protein	86310	86867	+	NADPH:quinone oxidoreductase
contig_27	fig|6666666.28487.peg.2159	Protein	87330	87569	+	Glutaredoxin-like protein NrdH, required for reduction of Ribonucleotide reductase class Ib
contig_27	fig|6666666.28487.peg.2160	Protein	87610	88056	+	Ribonucleotide reduction protein NrdI
contig_27	fig|6666666.28487.peg.2161	Protein	88023	90194	+	Ribonucleotide reductase of class Ib (aerobic), alpha subunit (EC 1.17.4.1)
contig_27	fig|6666666.28487.peg.2162	Protein	90690	90355	-	hypothetical protein
contig_27	fig|6666666.28487.peg.2163	Protein	91188	90805	-	FIG00828816: hypothetical protein
contig_27	fig|6666666.28487.peg.2164	Protein	91217	91930	+	Transcriptional regulator, LysR family
contig_27	fig|6666666.28487.peg.2165	Protein	92428	92006	-	Lactoylglutathione lyase (EC 4.4.1.5)
contig_27	fig|6666666.28487.peg.2166	Protein	92832	92425	-	Glyoxalase family protein
contig_27	fig|6666666.28487.peg.2167	Protein	93345	92842	-	FIG00829766: hypothetical protein
contig_27	fig|6666666.28487.peg.2168	Protein	94534	93353	-	Geranylgeranyl reductase (EC 1.3.1.83)
contig_27	fig|6666666.28487.peg.2169	Protein	94687	95427	+	Transcriptional regulator, TetR family
contig_27	fig|6666666.28487.peg.2170	Protein	95861	95493	-	hypothetical protein

contig_27	fig|6666666.28487.peg.2171	Protein	97057	95990	-	transcriptional regulator, SARP family
contig_27	fig|6666666.28487.peg.2172	Protein	97257	98219	+	Ribonucleotide reductase of class Ib (aerobic), beta subunit (EC 1.17.4.1)
contig_27	fig|6666666.28487.peg.2173	Protein	98428	99150	+	hypothetical protein
contig_27	fig|6666666.28487.peg.2174	Protein	101468	99213	-	FIG00832039: hypothetical protein
contig_27	fig|6666666.28487.peg.2175	Protein	101497	101850	+	nitrate/nitrite response regulator protein
contig_27	fig|6666666.28487.peg.2176	Protein	102521	101847	-	two-component system response regulator
contig_27	fig|6666666.28487.peg.2177	Protein	102575	104740	+	Periplasmic aromatic aldehyde oxidoreductase, molybdenum binding subunit YagR
contig_27	fig|6666666.28487.peg.2178	Protein	104737	105273	+	Periplasmic aromatic aldehyde oxidoreductase, iron-sulfur subunit YagT
contig_27	fig|6666666.28487.peg.2179	Protein	105267	106256	+	Periplasmic aromatic aldehyde oxidoreductase, FAD binding subunit YagS
contig_27	fig|6666666.28487.peg.2180	Protein	107845	106253	-	Probable amino acid permease
contig_27	fig|6666666.28487.peg.2181	Protein	108079	108420	+	FIG00822200: hypothetical protein
contig_27	fig|6666666.28487.peg.2182	Protein	109531	108485	-	Alcohol dehydrogenase (EC 1.1.1.1)
contig_27	fig|6666666.28487.peg.2183	Protein	110343	109642	-	Transcriptional regulator, TetR family
contig_27	fig|6666666.28487.peg.2184	Protein	110554	112578	+	Oligopeptide transport system permease protein OppB (TC 3.A.1.5.1)
contig_27	fig|6666666.28487.peg.2185	Protein	112578	113447	+	Dipeptide transport system permease protein DppC (TC 3.A.1.5.2)
contig_27	fig|6666666.28487.peg.2186	Protein	113453	114457	+	Dipeptide transport system permease protein DppB (TC

						3.A.1.5.2)
contig_27	fig|6666666.28487.peg.2187	Protein	114458	115105	+	Nicotinamidase/isochorismatase family protein
contig_27	fig|6666666.28487.peg.2188	Protein	115102	116679	+	Dipeptide-binding ABC transporter, periplasmic substrate-binding component (TC 3.A.1.5.2); Putative hemin-binding lipoprotein
contig_27	fig|6666666.28487.peg.2189	Protein	116679	117515	+	N-acyl homoserine lactone hydrolase
contig_27	fig|6666666.28487.peg.2190	Protein	117524	118840	+	Allantoinase (EC 3.5.2.5)
contig_27	fig|6666666.28487.peg.2191	Protein	118837	119859	+	Allantoicase (EC 3.5.3.4)
contig_27	fig|6666666.28487.peg.2192	Protein	119856	120377	+	FIG00823998: hypothetical protein
contig_27	fig|6666666.28487.peg.2193	Protein	120374	121429	+	2-Oxobutyrate oxidase, putative
contig_27	fig|6666666.28487.peg.2194	Protein	122621	121422	-	Iron(III) dicitrate transport system, periplasmic iron-binding protein FecB (TC 3.A.1.14.1)
contig_27	fig|6666666.28487.peg.2195	Protein	122760	124505	+	Cytochrome c oxidase polypeptide I (EC 1.9.3.1)
contig_27	fig|6666666.28487.peg.2196	Protein	124525	125754	+	Phosphoserine phosphatase (EC 3.1.3.3)
contig_27	fig|6666666.28487.peg.2197	Protein	127344	125830	-	Transcriptional regulator, GntR family domain / Aspartate aminotransferase (EC 2.6.1.1)
contig_27	fig|6666666.28487.peg.2198	Protein	127357	127488	+	FIG00830841: hypothetical protein
contig_27	fig|6666666.28487.peg.2199	Protein	127489	128166	+	putative integral membrane protein
contig_27	fig|6666666.28487.peg.2200	Protein	128129	128794	+	Pantothenate kinase (EC 2.7.1.33)
contig_27	fig|6666666.28487.peg.2201	Protein	128808	129650	+	ABC transporter ATP-binding protein
contig_28	fig|6666666.28487.peg.2202	Protein	1873	4362	+	hypothetical protein

contig_28	fig|6666666.28487.peg.2203	Protein	8006	9427	+	POSSIBLE CONSERVED POLYKETIDE SYNTHASE ASSOCIATED PROTEIN PAPA2
contig_28	fig|6666666.28487.peg.2204	Protein	9491	12499	+	membrane protein, MmpL family, putative
contig_28	fig|6666666.28487.peg.2205	Protein	12642	14348	+	Long-chain-fatty-acid--CoA ligase (EC 6.2.1.3)
contig_28	fig|6666666.28487.peg.2206	Protein	14364	15512	+	POSSIBLE EXPORTED PROTEIN
contig_28	fig|6666666.28487.peg.2207	Protein	16159	15509	-	Lepb1170_F2_64
contig_28	fig|6666666.28487.peg.2208	Protein	16277	16786	+	Nitrilotriacetate monooxygenase component B (EC 1.14.13.-)
contig_28	fig|6666666.28487.peg.2209	Protein	16978	16817	-	FIG00821372: hypothetical protein
contig_28	fig|6666666.28487.peg.2210	Protein	17481	17038	-	Rhodanese-related sulfurtransferase
contig_28	fig|6666666.28487.peg.2211	Protein	19306	17573	-	Acyl-CoA dehydrogenase (EC 1.3.99.3)
contig_28	fig|6666666.28487.peg.2212	Protein	19367	19825	+	Leucine-responsive regulatory protein, regulator for leucine (or lrp) regulon and high-affinity branched-chain amino acid transport system
contig_28	fig|6666666.28487.peg.2213	Protein	19993	20256	+	hypothetical protein
contig_28	fig|6666666.28487.peg.2214	Protein	21121	20369	-	Succinate dehydrogenase iron-sulfur protein (EC 1.3.99.1)
contig_28	fig|6666666.28487.peg.2215	Protein	23049	21124	-	Succinate dehydrogenase flavoprotein subunit (EC 1.3.99.1)
contig_28	fig|6666666.28487.peg.2216	Protein	23917	23099	-	putative succinate dehydrogenase [membrane anchor subunit] (succinic dehydrogenase)
contig_28	fig|6666666.28487.peg.2217	Protein	24293	23991	-	FIG00820825: hypothetical protein
contig_28	fig|6666666.28487.peg.2218	Protein	24413	25168	+	Probable carboxyvinyl-carboxyphosphonate phosphorylmutase (EC 2.7.8.23)

contig_28	fig|6666666.28487.peg.2219	Protein	25604	25173	-	hypothetical protein
contig_28	fig|6666666.28487.peg.2220	Protein	26193	25756	-	UspA domain protein
contig_28	fig|6666666.28487.peg.2221	Protein	26335	26799	+	Alkaline shock protein 23
contig_28	fig|6666666.28487.peg.2222	Protein	26799	27113	+	hypothetical protein
contig_28	fig|6666666.28487.peg.2223	Protein	27116	27379	+	hypothetical protein
contig_28	fig|6666666.28487.peg.2224	Protein	27376	27555	+	conserved hypothetical protein
contig_28	fig|6666666.28487.peg.2225	Protein	27548	27922	+	hypothetical protein
contig_28	fig|6666666.28487.peg.2226	Protein	27919	28455	+	hypothetical protein
contig_28	fig|6666666.28487.peg.2227	Protein	28458	29021	+	hypothetical protein
contig_28	fig|6666666.28487.peg.2228	Protein	30099	29035	-	hypothetical protein
contig_28	fig|6666666.28487.peg.2229	Protein	30600	30199	-	FIG00829066: hypothetical protein
contig_28	fig|6666666.28487.peg.2230	Protein	31130	30699	-	Heat shock protein hsp
contig_28	fig|6666666.28487.peg.2231	Protein	31330	33888	+	Nitrite reductase [NAD(P)H] large subunit (EC 1.7.1.4)
contig_28	fig|6666666.28487.peg.2232	Protein	33895	34254	+	Nitrite reductase [NAD(P)H] small subunit (EC 1.7.1.4)
contig_28	fig|6666666.28487.peg.2233	Protein	34380	34733	+	Ferredoxin
contig_28	fig|6666666.28487.peg.2234	Protein	35447	34713	-	Sirohydrochlorin ferrochelatase (EC 4.99.1.4)
contig_28	fig|6666666.28487.peg.2235	Protein	36592	35444	-	Bifunctional uroporphyrinogen-III synthetase/response regulator domain protein
contig_28	fig|6666666.28487.peg.2236	Protein	37249	36707	-	Aminoglycoside 2'-N-acetyltransferase AAC (AAC(2')-IC)
contig_28	fig|6666666.28487.peg.2237	Protein	38179	37283	-	Allophanate hydrolase 2 subunit 2 (EC 3.5.1.54)

contig_28	fig|6666666.28487.peg.2238	Protein	38859	38176	-	Allophanate hydrolase 2 subunit 1 (EC 3.5.1.54)
contig_28	fig|6666666.28487.peg.2239	Protein	39630	38944	-	Putative preQ0 transporter
contig_28	fig|6666666.28487.peg.2240	Protein	40598	39645	-	PROBABLE PERIPLASMIC IRON-TRANSPORT LIPOPROTEIN
contig_28	fig|6666666.28487.peg.2241	Protein	41127	40612	-	hypothetical protein
contig_28	fig|6666666.28487.peg.2242	Protein	41810	41202	-	Transporter, LysE family
contig_28	fig|6666666.28487.peg.2243	Protein	41945	42133	+	hypothetical protein
contig_28	fig|6666666.28487.peg.2244	Protein	42785	42246	-	hypothetical protein
contig_28	fig|6666666.28487.peg.2245	Protein	43420	42980	-	hypothetical protein
contig_28	fig|6666666.28487.peg.2246	Protein	44244	43417	-	Beta-ketoadipate enol-lactone hydrolase (EC 3.1.1.24)
contig_28	fig|6666666.28487.peg.2247	Protein	44827	44708	-	hypothetical protein
contig_28	fig|6666666.28487.peg.2248	Protein	45033	44821	-	hypothetical protein
contig_28	fig|6666666.28487.peg.2249	Protein	45460	45296	-	hypothetical protein
contig_28	fig|6666666.28487.peg.2250	Protein	46394	45585	-	6-phosphogluconate dehydrogenase, NAD-binding protein
contig_28	fig|6666666.28487.peg.2251	Protein	47020	46418	-	methyltransferase
contig_28	fig|6666666.28487.peg.2252	Protein	48027	47020	-	Tricarboxylate transport protein TctC
contig_28	fig|6666666.28487.peg.2253	Protein	49566	48067	-	Tricarboxylate transport membrane protein TctA
contig_28	fig|6666666.28487.peg.2254	Protein	50007	49570	-	hypothetical protein
contig_28	fig|6666666.28487.peg.2255	Protein	51062	50298	-	Transcriptional regulator, IclR family
contig_28	fig|6666666.28487.peg.2256	Protein	51344	51210	-	hypothetical protein

contig_28	fig|6666666.28487.peg.2257	Protein	51463	51585	+	hypothetical protein
contig_28	fig|6666666.28487.peg.2258	Protein	51707	52111	+	FIG00824120: hypothetical protein
contig_28	fig|6666666.28487.peg.2259	Protein	52095	52379	+	conserved hypothetical protein
contig_28	fig|6666666.28487.peg.2260	Protein	52515	52381	-	hypothetical protein
contig_28	fig|6666666.28487.peg.2261	Protein	52575	53375	+	FIG00823047: hypothetical protein
contig_28	fig|6666666.28487.peg.2262	Protein	54087	53419	-	Transcriptional regulator, TetR family
contig_28	fig|6666666.28487.peg.2263	Protein	54214	55848	+	FIG00830506: hypothetical protein
contig_28	fig|6666666.28487.peg.2264	Protein	55967	56680	+	serine esterase, cutinase family
contig_28	fig|6666666.28487.peg.2265	Protein	56738	58204	+	Dimethylaniline monooxygenase (N-oxide-forming)(EC:1.14.13.8)
contig_28	fig|6666666.28487.peg.2266	Protein	60039	58810	-	putative cytochrome P450 hydroxylase
contig_28	fig|6666666.28487.peg.2267	Protein	60235	61104	+	Transcriptional regulator
contig_28	fig|6666666.28487.peg.2268	Protein	61129	61908	+	Precorrin-6A synthase (EC 2.1.1.152)
contig_28	fig|6666666.28487.peg.2269	Protein	61905	63377	+	Cobyric acid synthase
contig_28	fig|6666666.28487.peg.2270	Protein	63374	64120	+	Cobalt-precorrin-6x reductase (EC 1.3.1.54)
contig_28	fig|6666666.28487.peg.2271	Protein	64117	64641	+	Adenosylcobinamide-phosphate guanylyltransferase (EC 2.7.7.62)
contig_28	fig|6666666.28487.peg.2272	Protein	64654	65466	+	Formamidopyrimidine-DNA glycosylase (EC 3.2.2.23)
contig_28	fig|6666666.28487.peg.2273	Protein	65738	65442	-	hypothetical protein
contig_28	fig|6666666.28487.peg.2274	Protein	66370	65735	-	Cobalamin biosynthesis protein BluB @ 5,6-dimethylbenzimidazole synthase, flavin destructase family

contig_28	fig|6666666.28487.peg.2275	Protein	67134	66367	-	Cobalt-precorrin-4 C11-methyltransferase (EC 2.1.1.133)
contig_28	fig|6666666.28487.peg.2276	Protein	67806	67228	-	Transcriptional regulator, TetR family
contig_28	fig|6666666.28487.peg.2277	Protein	69413	67989	-	Wax ester synthase/acyl-CoA:diacylglycerol acyltransferase
contig_28	fig|6666666.28487.peg.2278	Protein	70483	69602	-	COG0583: Transcriptional regulator
contig_28	fig|6666666.28487.peg.2279	Protein	70585	71730	+	hypothetical protein
contig_28	fig|6666666.28487.peg.2280	Protein	71849	72382	+	Transcriptional regulator, TetR family
contig_28	fig|6666666.28487.peg.2281	Protein	72471	74081	+	Sulfite reductase [NADPH] flavoprotein alpha-component (EC 1.8.1.2)
contig_28	fig|6666666.28487.peg.2282	Protein	75562	74156	-	Xanthine/uracil/thiamine/ascorbate permease family protein
contig_28	fig|6666666.28487.peg.2283	Protein	75827	75570	-	FIG00824833: hypothetical protein
contig_28	fig|6666666.28487.peg.2284	Protein	77015	75840	-	Acyl-CoA dehydrogenase, short-chain specific (EC 1.3.99.2)
contig_29	fig|6666666.28487.peg.2285	Protein	719	9	-	DNA-binding response regulator
contig_29	fig|6666666.28487.peg.2286	Protein	821	1555	+	FIG00825385: hypothetical protein
contig_29	fig|6666666.28487.peg.2287	Protein	1749	1552	-	FIG00822650: hypothetical protein
contig_29	fig|6666666.28487.rna.10	RNA	2084	2155	+	tRNA-Thr-TGT
contig_29	fig|6666666.28487.peg.2288	Protein	2951	5242	+	Trehalose synthase
contig_29	fig|6666666.28487.peg.2289	Protein	5331	6584	+	ErfK/YbiS/YcfS/YnhG family protein
contig_29	fig|6666666.28487.peg.2290	Protein	6598	6975	+	Transcriptional regulator, MecI family
contig_29	fig|6666666.28487.peg.2291	Protein	6972	7889	+	Peptidase M48, Ste24p precursor
contig_29	fig|6666666.28487.peg.2292	Protein	8293	10350	+	FIG00822442: hypothetical protein

contig_29	fig|6666666.28487.peg.2293	Protein	11027	10347	-	membrane protein
contig_29	fig|6666666.28487.peg.2294	Protein	11910	11245	-	D-alanyl-D-alanine dipeptidase
contig_29	fig|6666666.28487.peg.2295	Protein	11973	13094	+	Xanthine and CO dehydrogenases maturation factor, XdhC/CoxF family
contig_29	fig|6666666.28487.peg.2296	Protein	13181	15532	+	Carbon monoxide dehydrogenase large chain (EC 1.2.99.2)
contig_29	fig|6666666.28487.peg.2297	Protein	15535	16224	+	carbon monoxide dehydrogenase G protein
contig_29	fig|6666666.28487.peg.2298	Protein	16221	17114	+	Carbon monoxide dehydrogenase medium chain (EC 1.2.99.2)
contig_29	fig|6666666.28487.peg.2299	Protein	17107	17580	+	Carbon monoxide dehydrogenase small chain (EC 1.2.99.2)
contig_29	fig|6666666.28487.peg.2300	Protein	17670	18569	+	3-hydroxyisobutyrate dehydrogenase (EC 1.1.1.31)
contig_29	fig|6666666.28487.peg.2301	Protein	19478	18636	-	short-chain dehydrogenase/reductase SDR
contig_29	fig|6666666.28487.peg.2302	Protein	20426	19530	-	Enoyl-CoA hydratase (EC 4.2.1.17)
contig_29	fig|6666666.28487.peg.2303	Protein	20518	20685	+	FIG00820754: hypothetical protein
contig_29	fig|6666666.28487.peg.2304	Protein	20685	22253	+	Alpha,alpha-trehalose-phosphate synthase [UDP-forming] (EC 2.4.1.15)
contig_29	fig|6666666.28487.peg.2305	Protein	22734	22240	-	MCE-associated transmembrane protein
contig_29	fig|6666666.28487.peg.2306	Protein	23234	22734	-	MCE-associated alanine and valine rich protein
contig_29	fig|6666666.28487.peg.2307	Protein	25153	23447	-	MCE-family protein Mce1F
contig_29	fig|6666666.28487.peg.2308	Protein	26328	25153	-	MCE-family lipoprotein LprK (MCE-family lipoprotein Mce1e)
contig_29	fig|6666666.28487.peg.2309	Protein	26672	26325	-	MCE-family protein Mce1D
contig_29	fig|6666666.28487.peg.2310	Protein	27724	26642	-	MCE-family protein Mce1D

contig_29	fig|6666666.28487.peg.2311	Protein	28779	27721	-	MCE-family protein Mce1C
contig_29	fig|6666666.28487.peg.2312	Protein	29800	28772	-	MCE-family protein Mce1B
contig_29	fig|6666666.28487.peg.2313	Protein	31002	29800	-	MCE-family protein Mce1A
contig_29	fig|6666666.28487.peg.2314	Protein	31857	31015	-	Conserved hypothetical integral membrane protein YrbE1B
contig_29	fig|6666666.28487.peg.2315	Protein	32653	31889	-	Conserved hypothetical integral membrane protein YrbE1A
contig_29	fig|6666666.28487.peg.2316	Protein	33826	32918	-	Probable short-chain type dehydrogenase/reductase (EC 1.-.-.-)
contig_29	fig|6666666.28487.peg.2317	Protein	34031	33840	-	Ferredoxin
contig_29	fig|6666666.28487.peg.2318	Protein	34193	35374	+	FadE30
contig_29	fig|6666666.28487.peg.2319	Protein	35392	36489	+	Acyl-CoA dehydrogenase, short-chain specific (EC 1.3.99.2)
contig_29	fig|6666666.28487.peg.2320	Protein	36498	38009	+	Long-chain-fatty-acid--CoA ligase (EC 6.2.1.3)
contig_29	fig|6666666.28487.peg.2321	Protein	38039	39673	+	Long-chain-fatty-acid--CoA ligase (EC 6.2.1.3)
contig_29	fig|6666666.28487.peg.2322	Protein	39756	41249	+	3-ketosteroid-delta1-dehydrogenase
contig_29	fig|6666666.28487.peg.2323	Protein	41246	42394	+	Terminal oxygenase KshA
contig_29	fig|6666666.28487.peg.2324	Protein	42479	43222	+	PROBABLE CONSERVED LIPOPROTEIN LPQN
contig_29	fig|6666666.28487.peg.2325	Protein	44853	43297	-	COG0028: Thiamine pyrophosphate-requiring enzymes
contig_29	fig|6666666.28487.peg.2326	Protein	45995	44874	-	Enoyl-[acyl-carrier-protein] reductase [FMN] (EC 1.3.1.9)
contig_29	fig|6666666.28487.peg.2327	Protein	47647	45998	-	Long-chain-fatty-acid--CoA ligase (EC 6.2.1.3)
contig_29	fig|6666666.28487.peg.2328	Protein	47710	48516	+	Enoyl-CoA hydratase (EC 4.2.1.17)
contig_29	fig|6666666.28487.peg.2329	Protein	49188	48592	-	FMN-dependent NADH-azoreductase

contig_29	fig|6666666.28487.peg.2330	Protein	49272	49754	+	regulatory protein, MarR
contig_29	fig|6666666.28487.peg.2331	Protein	51065	49842	-	putative cytochrome P450 hydroxylase
contig_29	fig|6666666.28487.peg.2332	Protein	51811	51062	-	Acetoacetate decarboxylase family protein
contig_29	fig|6666666.28487.peg.2333	Protein	52896	51865	-	Coenzyme F420-dependent oxidoreductase
contig_29	fig|6666666.28487.peg.2334	Protein	52967	53959	+	FIG00996312: hypothetical protein
contig_29	fig|6666666.28487.peg.2335	Protein	54007	55062	+	Lipid-transfer protein
contig_29	fig|6666666.28487.peg.2336	Protein	55065	56252	+	3-ketoacyl-CoA thiolase
contig_29	fig|6666666.28487.peg.2337	Protein	56873	56325	-	Transcriptional regulator, TetR family
contig_29	fig|6666666.28487.peg.2338	Protein	58152	56929	-	FIG00824895: hypothetical protein
contig_29	fig|6666666.28487.peg.2339	Protein	58332	59513	+	Terminal oxygenase KshA
contig_29	fig|6666666.28487.peg.2340	Protein	59574	60032	+	FIG00820011: hypothetical protein
contig_29	fig|6666666.28487.peg.2341	Protein	61219	60074	-	FIG00994930: hypothetical protein
contig_29	fig|6666666.28487.peg.2342	Protein	62010	61219	-	3-oxoacyl-[acyl-carrier protein] reductase (EC 1.1.1.100)
contig_29	fig|6666666.28487.peg.2343	Protein	63190	62012	-	FIG00994788: hypothetical protein
contig_29	fig|6666666.28487.peg.2344	Protein	63229	64131	+	Transcriptional regulator, IclR family
contig_29	fig|6666666.28487.peg.2345	Protein	66232	64142	-	ATP-dependent DNA helicase RecQ
contig_29	fig|6666666.28487.peg.2346	Protein	66949	66278	-	COG1272: Predicted membrane protein hemolysin III homolog
contig_29	fig|6666666.28487.peg.2347	Protein	68140	67085	-	4-hydroxy-2-oxovalerate aldolase (EC 4.1.3.39)
contig_29	fig|6666666.28487.peg.2348	Protein	69087	68137	-	Acetaldehyde dehydrogenase, acetylating, (EC 1.2.1.10) in

						gene cluster for degradation of phenols, cresols, catechol
contig_29	fig|6666666.28487.peg.2349	Protein	69868	69098	-	2-keto-4-pentenoate hydratase (EC 4.2.1.-)
contig_29	fig|6666666.28487.peg.2350	Protein	69955	71637	+	3-oxosteroid 1-dehydrogenase (EC 1.3.99.4)
contig_29	fig|6666666.28487.peg.2351	Protein	71640	72500	+	Enoyl-CoA hydratase
contig_29	fig|6666666.28487.peg.2352	Protein	72992	72507	-	FIG00828503: hypothetical protein
contig_29	fig|6666666.28487.peg.2353	Protein	74505	73114	-	PE_PGRS family protein
contig_29	fig|6666666.28487.peg.2354	Protein	74738	74863	+	hypothetical protein
contig_29	fig|6666666.28487.peg.2355	Protein	75240	76151	+	Immunogenic protein MPT63/MPB63 precursor
contig_29	fig|6666666.28487.peg.2356	Protein	76913	76206	-	Serine/threonine protein kinase (EC 2.7.11.1)
contig_29	fig|6666666.28487.peg.2357	Protein	78153	76987	-	Lipid carrier protein IgrF
contig_29	fig|6666666.28487.peg.2358	Protein	78578	78150	-	Enoyl coenzyme A hydratase IgrE
contig_29	fig|6666666.28487.peg.2359	Protein	79513	78560	-	Conserved protein IgrD
contig_29	fig|6666666.28487.peg.2360	Protein	80673	79510	-	Probable acyl-CoA dehydrogenase FadE29 (EC 1.3.99.-); Acyl-CoA dehydrogenase IgrC
contig_29	fig|6666666.28487.peg.2361	Protein	81704	80703	-	Probable acyl-CoA dehydrogenase FadE28 (EC 1.3.99.-); Acyl-CoA dehydrogenase IgrB
contig_29	fig|6666666.28487.peg.2362	Protein	82993	81731	-	Putative cytochrome P450 125 (EC 1.14.-.-); Putative cytochrome P450 IgrA
contig_29	fig|6666666.28487.peg.2363	Protein	83115	84278	+	Probable acetyl-CoA acetyltransferase FadA5 (EC 2.3.1.9)
contig_29	fig|6666666.28487.peg.2364	Protein	84278	84664	+	CysQ, putative
contig_29	fig|6666666.28487.peg.2365	Protein	84673	85182	+	Putative uncharacterized protein BCG_3611

contig_29	fig|6666666.28487.peg.2366	Protein	85321	86016	+	putative GAF sensor protein
contig_29	fig|6666666.28487.peg.2367	Protein	86931	86026	-	Putative uncharacterized protein (Hypothetical short-chain type dehydrogenase/reductase)
contig_29	fig|6666666.28487.peg.2368	Protein	87732	86944	-	Probable short-chain type dehydrogenase/reductase (EC 1.-.-.-)
contig_29	fig|6666666.28487.peg.2369	Protein	87785	88540	+	Probable enoyl-CoA hydratase EchA20 (EC 4.2.1.17)
contig_29	fig|6666666.28487.peg.2370	Protein	88503	89450	+	Putative CoA-transferase subunit alpha Rv3551/MT3655 (EC 2.8.3.-)
contig_29	fig|6666666.28487.peg.2371	Protein	89447	90196	+	Putative CoA-transferase subunit beta Rv3552/MT3656 (EC 2.8.3.-)
contig_29	fig|6666666.28487.peg.2372	Protein	90193	91269	+	2-nitropropane dioxygenase (EC 1.13.11.32)
contig_29	fig|6666666.28487.peg.2373	Protein	91370	91963	+	Putative uncharacterized protein
contig_29	fig|6666666.28487.peg.2374	Protein	91960	92700	+	FIG00824227: hypothetical protein
contig_29	fig|6666666.28487.peg.2375	Protein	92751	93866	+	Sorbitol dehydrogenase (EC 1.1.1.14)
contig_29	fig|6666666.28487.peg.2376	Protein	94213	93947	-	Ectoine hydroxylase (EC 1.17.-.-)
contig_29	fig|6666666.28487.peg.2377	Protein	95913	94756	-	Probable acetyl-CoA acetyltransferase FadA6 (EC 2.3.1.9)
contig_29	fig|6666666.28487.peg.2378	Protein	96509	95913	-	Transcriptional factor in putative operon for degradation of branched-chain alkanes, nitroalkanes and may be also cyclic ketones, alkenoic acids
contig_29	fig|6666666.28487.peg.2379	Protein	97433	96537	-	NAD-dependent epimerase/dehydratase
contig_29	fig|6666666.28487.peg.2380	Protein	98707	97637	-	oxidoreductase domain protein
contig_29	fig|6666666.28487.peg.2381	Protein	100352	98814	-	hypothetical protein

contig_29	fig|6666666.28487.peg.2382	Protein	101365	100502	-	hypothetical protein
contig_29	fig|6666666.28487.peg.2383	Protein	101560	102843	+	hypothetical protein
contig_29	fig|6666666.28487.peg.2384	Protein	102840	103889	+	hypothetical protein
contig_29	fig|6666666.28487.peg.2385	Protein	103957	104826	+	conserved hypothetical protein
contig_29	fig|6666666.28487.peg.2386	Protein	105690	104842	-	glycosyl transferase, family 2
contig_29	fig|6666666.28487.peg.2387	Protein	106470	105703	-	glycosyl transferase, WecB/TagA/CpsF family
contig_29	fig|6666666.28487.peg.2388	Protein	107836	106538	-	Tyrosine-protein kinase transmembrane modulator EpsC
contig_29	fig|6666666.28487.peg.2389	Protein	108534	110522	+	FIG00830268: hypothetical protein
contig_29	fig|6666666.28487.peg.2390	Protein	110653	111531	+	FIG00826682: hypothetical protein
contig_29	fig|6666666.28487.peg.2391	Protein	111515	113218	+	Glucose-methanol-choline (GMC) oxidoreductase:NAD binding site
contig_29	fig|6666666.28487.peg.2392	Protein	113439	114410	+	UDP-glucose 4-epimerase (EC 5.1.3.2)
contig_29	fig|6666666.28487.peg.2393	Protein	115975	114422	-	hypothetical protein
contig_29	fig|6666666.28487.peg.2394	Protein	117003	115972	-	hypothetical protein
contig_29	fig|6666666.28487.peg.2395	Protein	117689	117000	-	putative two-component system response regulator
contig_29	fig|6666666.28487.peg.2396	Protein	118553	117762	-	Short-chain dehydrogenase/reductase SDR
contig_29	fig|6666666.28487.peg.2397	Protein	119736	118588	-	FadE30
contig_29	fig|6666666.28487.peg.2398	Protein	119796	121352	+	Long-chain-fatty-acid--CoA ligase (EC 6.2.1.3)
contig_29	fig|6666666.28487.peg.2399	Protein	121353	122477	+	Acyl-CoA dehydrogenase, short-chain specific (EC 1.3.99.2)
contig_29	fig|6666666.28487.peg.2400	Protein	122485	123438	+	Butyryl-CoA dehydrogenase (EC 1.3.99.2)

contig_29	fig|6666666.28487.peg.2401	Protein	123438	124379	+	Butyryl-CoA dehydrogenase (EC 1.3.99.2)
contig_29	fig|6666666.28487.peg.2402	Protein	124389	125543	+	Valine--pyruvate aminotransferase (EC 2.6.1.66)
contig_29	fig|6666666.28487.peg.2403	Protein	125557	126723	+	hypothetical protein
contig_29	fig|6666666.28487.peg.2404	Protein	126825	128108	+	CONSERVED 13E12 REPEAT FAMILY PROTEIN
contig_29	fig|6666666.28487.peg.2405	Protein	129385	128111	-	Xylose ABC transporter, permease protein XylH
contig_29	fig|6666666.28487.peg.2406	Protein	130185	129382	-	D-xylose transport ATP-binding protein XylG
contig_29	fig|6666666.28487.peg.2407	Protein	131265	130192	-	Xylose ABC transporter, periplasmic xylose-binding protein XylF
contig_29	fig|6666666.28487.peg.2408	Protein	132595	131372	-	Xylose isomerase (EC 5.3.1.5)
contig_29	fig|6666666.28487.peg.2409	Protein	132727	133926	+	Xylose-responsive transcription regulator, ROK family
contig_29	fig|6666666.28487.peg.2410	Protein	133929	135323	+	Xylulose kinase (EC 2.7.1.17)
contig_29	fig|6666666.28487.peg.2411	Protein	137226	135325	-	Acetoacetyl-CoA synthetase (EC 6.2.1.16)
contig_29	fig|6666666.28487.peg.2412	Protein	137969	137223	-	D-beta-hydroxybutyrate dehydrogenase (EC 1.1.1.30)
contig_29	fig|6666666.28487.peg.2413	Protein	139348	137966	-	metabolite-proton symporter
contig_29	fig|6666666.28487.peg.2414	Protein	139468	140367	+	Transcriptional regulator, LysR family
contig_29	fig|6666666.28487.peg.2415	Protein	140377	141141	+	Monoglyceride lipase (EC 3.1.1.23)
contig_29	fig|6666666.28487.peg.2416	Protein	141747	141196	-	Transcriptional regulator, TetR family
contig_29	fig|6666666.28487.peg.2417	Protein	141806	142261	+	hypothetical protein
contig_29	fig|6666666.28487.peg.2418	Protein	142824	142258	-	Nitrilotriacetate monooxygenase component B (EC 1.14.13.-)
contig_29	fig|6666666.28487.peg.2419	Protein	143726	142827	-	2,3-dihydroxybiphenyl 1,2-dioxygenase

contig_29	fig|6666666.28487.peg.2420	Protein	144633	143740	-	2-hydroxy-6-oxo-6-phenylhexa-2,4-dienoate hydrolase (EC 3.7.1.-)
contig_29	fig|6666666.28487.peg.2421	Protein	145817	144633	-	POSSIBLE OXIDOREDUCTASE
contig_29	fig|6666666.28487.peg.2422	Protein	145979	147058	+	Phenylacetate-CoA oxygenase/reductase, PaaK subunit
contig_29	fig|6666666.28487.peg.2423	Protein	147081	147662	+	FIG00821138: hypothetical protein
contig_29	fig|6666666.28487.peg.2424	Protein	149809	147659	-	Acyl-CoA dehydrogenase (EC 1.3.99.-)
contig_29	fig|6666666.28487.peg.2425	Protein	150078	150692	+	Transcriptional regulator kstR (Rv3574), TetR family
contig_29	fig|6666666.28487.peg.2426	Protein	150696	151454	+	Trehalose-6-phosphate phosphatase (EC 3.1.3.12)
contig_29	fig|6666666.28487.peg.2427	Protein	151518	152114	+	Thioredoxin reductase (EC 1.8.1.9)
contig_29	fig|6666666.28487.peg.2428	Protein	153211	152111	-	Transcriptional regulator, LacI family
contig_29	fig|6666666.28487.peg.2429	Protein	154122	153250	-	Zinc ABC transporter, inner membrane permease protein ZnuB
contig_29	fig|6666666.28487.peg.2430	Protein	154913	154119	-	Zinc ABC transporter, ATP-binding protein ZnuC
contig_29	fig|6666666.28487.peg.2431	Protein	155886	154900	-	Zinc ABC transporter, periplasmic-binding protein ZnuA
contig_29	fig|6666666.28487.peg.2432	Protein	156014	157138	+	diguanylate cyclase
contig_29	fig|6666666.28487.peg.2433	Protein	157988	157143	-	Thiosulfate sulfurtransferase, rhodanese (EC 2.8.1.1)
contig_29	fig|6666666.28487.peg.2434	Protein	159213	158011	-	putative secreted protein
contig_29	fig|6666666.28487.peg.2435	Protein	160160	159252	-	Zinc ABC transporter, periplasmic-binding protein ZnuA
contig_29	fig|6666666.28487.peg.2436	Protein	162148	160157	-	Zinc ABC transporter, inner membrane permease protein ZnuB
contig_29	fig|6666666.28487.peg.2437	Protein	162169	162819	+	Zinc ABC transporter, ATP-binding protein ZnuC

contig_29	fig|6666666.28487.peg.2438	Protein	162838	163596	+	Lactam utilization protein LamB
contig_29	fig|6666666.28487.peg.2439	Protein	163933	163589	-	Transcriptional regulator, ArsR family
contig_29	fig|6666666.28487.peg.2440	Protein	164036	165244	+	Permeases of the major facilitator superfamily
contig_29	fig|6666666.28487.peg.2441	Protein	167386	165206	-	Probable cation-transporting P-type ATPase C (EC 3.6.3.-) (Metal-transporting ATPase Mta72)
contig_29	fig|6666666.28487.peg.2442	Protein	167679	167392	-	hypothetical protein
contig_29	fig|6666666.28487.peg.2443	Protein	167742	168740	+	Membrane protein, putative
contig_29	fig|6666666.28487.peg.2444	Protein	168737	169450	+	Putative membrane protein
contig_29	fig|6666666.28487.peg.2445	Protein	169716	169444	-	LSU ribosomal protein L31p
contig_29	fig|6666666.28487.peg.2446	Protein	169824	170738	+	Zn-dependent hydrolases of the beta-lactamase fold
contig_29	fig|6666666.28487.peg.2447	Protein	170729	171967	+	Arsenic efflux pump protein
contig_29	fig|6666666.28487.peg.2448	Protein	172905	171964	-	23S rRNA (guanosine-2'-O-) -methyltransferase rlmB (EC 2.1.1.-)
contig_29	fig|6666666.28487.peg.2449	Protein	174321	172906	-	Cysteinyl-tRNA synthetase (EC 6.1.1.16)
contig_29	fig|6666666.28487.peg.2450	Protein	174762	174367	-	2-C-methyl-D-erythritol 2,4-cyclodiphosphate synthase (EC 4.6.1.12)
contig_29	fig|6666666.28487.peg.2451	Protein	175283	174846	-	CarD-like transcriptional regulator
contig_29	fig|6666666.28487.peg.2452	Protein	175654	176202	+	Lipoprotein LpqE
contig_29	fig|6666666.28487.peg.2453	Protein	176330	177655	+	DNA repair protein RadA
contig_29	fig|6666666.28487.peg.2454	Protein	177666	178787	+	DNA integrity scanning protein disA
contig_29	fig|6666666.28487.peg.2455	Protein	179580	178789	-	FIG00820636: hypothetical protein

contig_29	fig|6666666.28487.peg.2456	Protein	180233	179613	-	Carbonic anhydrase (EC 4.2.1.1)
contig_29	fig|6666666.28487.peg.2457	Protein	180232	181131	+	A/G-specific adenine glycosylase (EC 3.2.2.-)
contig_29	fig|6666666.28487.peg.2458	Protein	182115	181135	-	Transcriptional regulator, AraC family
contig_29	fig|6666666.28487.peg.2459	Protein	182188	182439	+	FIG00823222: hypothetical protein
contig_29	fig|6666666.28487.peg.2460	Protein	183189	182413	-	PUTATIVE HYDROLASE
contig_29	fig|6666666.28487.peg.2461	Protein	183229	183558	+	Antibiotic biosynthesis monooxygenase
contig_29	fig|6666666.28487.peg.2462	Protein	183527	184897	+	Beta-lactamase (EC 3.5.2.6)
contig_29	fig|6666666.28487.peg.2463	Protein	185444	184887	-	Probable phosphoglycerate mutase
contig_29	fig|6666666.28487.peg.2464	Protein	186251	185451	-	Predicted cobalt transporter CbtA
contig_29	fig|6666666.28487.peg.2465	Protein	186494	186279	-	FIG00823300: hypothetical protein
contig_29	fig|6666666.28487.peg.2466	Protein	190060	186656	-	hypothetical protein
contig_29	fig|6666666.28487.peg.2467	Protein	190303	190935	+	probable tetracycline repressor protein
contig_29	fig|6666666.28487.peg.2468	Protein	193504	190955	-	ATP-dependent Clp protease, ATP-binding subunit ClpC / Negative regulator of genetic competence clcC/mecB
contig_29	fig|6666666.28487.peg.2469	Protein	194145	193804	-	Histone protein Lsr2
contig_29	fig|6666666.28487.peg.2470	Protein	195769	194252	-	Lysyl-tRNA synthetase (class II) (EC 6.1.1.6)
contig_29	fig|6666666.28487.peg.2471	Protein	195799	197055	+	D-serine deaminase
contig_29	fig|6666666.28487.peg.2472	Protein	197874	197059	-	Pantothenate kinase type III, CoaX-like (EC 2.7.1.33)
contig_29	fig|6666666.28487.peg.2473	Protein	198826	197879	-	Pantoate--beta-alanine ligase (EC 6.3.2.1)
contig_29	fig|6666666.28487.peg.2474	Protein	199698	198823	-	FIG173306: hypothetical protein

contig_29	fig|6666666.28487.peg.2475	Protein	201019	199844	-	Possible membrane protein
contig_29	fig|6666666.28487.peg.2476	Protein	201636	201160	-	FIG027937: secreted protein
contig_29	fig|6666666.28487.peg.2477	Protein	202163	201636	-	2-amino-4-hydroxy-6-hydroxymethyldihydropteridine pyrophosphokinase (EC 2.7.6.3)
contig_29	fig|6666666.28487.peg.2478	Protein	202563	202183	-	Dihydroneopterin aldolase (EC 4.1.2.25)
contig_29	fig|6666666.28487.peg.2479	Protein	203371	202556	-	Dihydropteroate synthase (EC 2.5.1.15)
contig_29	fig|6666666.28487.peg.2480	Protein	204015	203407	-	GTP cyclohydrolase I (EC 3.5.4.16) type 1
contig_29	fig|6666666.28487.peg.2481	Protein	206411	204048	-	Cell division protein FtsH (EC 3.4.24.-)
contig_29	fig|6666666.28487.peg.2482	Protein	206708	206502	-	hypothetical protein
contig_29	fig|6666666.28487.peg.2483	Protein	206661	207557	+	Epoxide hydrolase (EC 3.3.2.9)
contig_29	fig|6666666.28487.peg.2484	Protein	207583	208323	+	Lipoprotein LpqG
contig_29	fig|6666666.28487.peg.2485	Protein	210563	208320	-	Formate dehydrogenase-O, major subunit (EC 1.2.1.2)
contig_29	fig|6666666.28487.peg.2486	Protein	211104	210574	-	Hypoxanthine-guanine phosphoribosyltransferase (EC 2.4.2.8)
contig_29	fig|6666666.28487.peg.2487	Protein	212140	211184	-	tRNA(Ile)-lysidine synthetase
contig_29	fig|6666666.28487.peg.2488	Protein	213219	212119	-	hypothetical protein
contig_29	fig|6666666.28487.peg.2489	Protein	214586	213216	-	D-alanyl-D-alanine carboxypeptidase (EC 3.4.16.4)
contig_29	fig|6666666.28487.peg.2490	Protein	214709	215194	+	Inorganic pyrophosphatase (EC 3.6.1.1)
contig_29	fig|6666666.28487.peg.2491	Protein	215274	215792	+	2-oxo-4-hydroxy-4-carboxy--5-ureidoimidazoline (OHCU) decarboxylase
contig_29	fig|6666666.28487.peg.2492	Protein	216175	215801	-	FIG00825404: hypothetical protein

contig_29	fig|6666666.28487.peg.2493	Protein	217310	216282	-	Integral membrane protein TerC
contig_29	fig|6666666.28487.peg.2494	Protein	217531	221490	+	Non-ribosomal peptide synthetase, terminal component
contig_29	fig|6666666.28487.peg.2495	Protein	221487	222833	+	Membrane alanine aminopeptidase (EC 3.4.11.2)
contig_29	fig|6666666.28487.peg.2496	Protein	222830	224101	+	putative conserved integral membrane protein
contig_29	fig|6666666.28487.peg.2497	Protein	224111	225082	+	UDP-glucose 4-epimerase (EC 5.1.3.2)
contig_29	fig|6666666.28487.peg.2498	Protein	225087	226853	+	PROBABLE CONSERVED TRANSMEMBRANE PROTEIN
contig_29	fig|6666666.28487.peg.2499	Protein	226923	227393	+	FIG00823426: hypothetical protein
contig_29	fig|6666666.28487.peg.2500	Protein	227616	228116	+	hypothetical protein
contig_29	fig|6666666.28487.peg.2501	Protein	229083	228253	-	ErfK/YbiS/YcfS/YnhG family protein
contig_29	fig|6666666.28487.peg.2502	Protein	229530	230693	+	Mobile element protein
contig_29	fig|6666666.28487.rna.11	RNA	230813	230741	-	tRNA-Thr-CGT
contig_29	fig|6666666.28487.peg.2503	Protein	232126	230912	-	DNA polymerase III delta prime subunit (EC 2.7.7.7)
contig_29	fig|6666666.28487.peg.2504	Protein	232209	233819	+	Adenylate cyclase (EC 4.6.1.1)
contig_29	fig|6666666.28487.peg.2505	Protein	236635	233816	-	DNA topoisomerase I (EC 5.99.1.2)
contig_29	fig|6666666.28487.peg.2506	Protein	237349	236771	-	FIG00995018: hypothetical protein
contig_29	fig|6666666.28487.peg.2507	Protein	237685	237482	-	Cold shock protein CspA
contig_29	fig|6666666.28487.peg.2508	Protein	237996	240347	+	ATP-dependent helicase
contig_29	fig|6666666.28487.peg.2509	Protein	240357	241427	+	FIG00821239: hypothetical protein
contig_29	fig|6666666.28487.peg.2510	Protein	242642	241446	-	serine/threonine protein kinase

contig_29	fig|6666666.28487.peg.2511	Protein	243050	242709	-	conserved hypothetical protein
contig_29	fig|6666666.28487.peg.2512	Protein	243327	243040	-	FIG00999563: hypothetical protein
contig_29	fig|6666666.28487.peg.2513	Protein	243572	243369	-	FIG043778: hypothetical protein
contig_29	fig|6666666.28487.peg.2514	Protein	244165	243593	-	type II secretion system protein
contig_29	fig|6666666.28487.peg.2515	Protein	244950	244162	-	FIG016317: Probable conserved transmembrane protein
contig_29	fig|6666666.28487.peg.2516	Protein	246125	244947	-	Flp pilus assembly protein, ATPase CpaF
contig_29	fig|6666666.28487.peg.2517	Protein	247216	246122	-	Putative morphological differentiation-related protein
contig_29	fig|6666666.28487.peg.2518	Protein	247656	248513	+	Phosphoserine phosphatase (EC 3.1.3.3)
contig_29	fig|6666666.28487.peg.2519	Protein	249715	248951	-	probable oxidoreductase
contig_29	fig|6666666.28487.peg.2520	Protein	249851	251797	+	Acetyl-coenzyme A synthetase (EC 6.2.1.1)
contig_29	fig|6666666.28487.peg.2521	Protein	251899	252159	+	hypothetical protein
contig_29	fig|6666666.28487.peg.2522	Protein	252838	252161	-	Protease
contig_29	fig|6666666.28487.peg.2523	Protein	253024	253530	+	FIG00820727: hypothetical protein
contig_29	fig|6666666.28487.peg.2524	Protein	253530	254498	+	Epoxide hydrolase (EC 3.3.2.9)
contig_29	fig|6666666.28487.peg.2525	Protein	255692	254499	-	putative serine protease
contig_29	fig|6666666.28487.peg.2526	Protein	256471	255689	-	Hypothetical nudix hydrolase YeaB
contig_29	fig|6666666.28487.peg.2527	Protein	257088	256468	-	Possible membrane-anchored thioredoxin-like protein
contig_29	fig|6666666.28487.peg.2528	Protein	257768	257121	-	Endonuclease III (EC 4.2.99.18)
contig_29	fig|6666666.28487.peg.2529	Protein	258024	258320	+	hypothetical protein
contig_29	fig|6666666.28487.peg.2530	Protein	258446	259120	+	cAMP-binding proteins - catabolite gene activator and

						regulatory subunit of cAMP-dependent protein kinases
contig_29	fig|6666666.28487.peg.2531	Protein	259931	259137	-	FIG146518: Zn-dependent hydrolases, including glyoxylases
contig_29	fig|6666666.28487.peg.2532	Protein	260399	259941	-	FIG137598: hypothetical protein
contig_29	fig|6666666.28487.peg.2533	Protein	260557	260396	-	FIG011121: hypothetical protein
contig_29	fig|6666666.28487.peg.2534	Protein	260632	261675	+	Arsenical pump-driving ATPase (EC 3.6.3.16)
contig_29	fig|6666666.28487.peg.2535	Protein	261706	262833	+	Arsenical pump-driving ATPase (EC 3.6.3.16)
contig_29	fig|6666666.28487.peg.2536	Protein	263257	262961	-	WhiB-type transcription regulator
contig_29	fig|6666666.28487.peg.2537	Protein	263652	266078	+	Multimodular transpeptidase-transglycosylase (EC 2.4.1.129) (EC 3.4.-.-)
contig_29	fig|6666666.28487.peg.2538	Protein	266125	267087	+	putative secreted protein
contig_29	fig|6666666.28487.peg.2539	Protein	267126	268184	+	Cysteine synthase (EC 2.5.1.47)
contig_29	fig|6666666.28487.rna.12	RNA	268427	268500	+	tRNA-Pro-CGG
contig_29	fig|6666666.28487.peg.2540	Protein	269615	268725	-	hypothetical protein
contig_29	fig|6666666.28487.peg.2541	Protein	269737	269612	-	hypothetical protein
contig_29	fig|6666666.28487.peg.2542	Protein	269718	270905	+	hypothetical protein
contig_29	fig|6666666.28487.peg.2543	Protein	270926	271645	+	hypothetical protein
contig_29	fig|6666666.28487.peg.2544	Protein	272420	271632	-	hypothetical protein
contig_29	fig|6666666.28487.peg.2545	Protein	272456	272935	+	hypothetical protein
contig_29	fig|6666666.28487.peg.2546	Protein	274496	272946	-	"phi-Carotenoid synthase" (EC 1.3.-.- and EC 2.1.1-)
contig_29	fig|6666666.28487.peg.2547	Protein	275262	274489	-	similar to membrane protein

contig_29	fig|6666666.28487.peg.2548	Protein	275389	276465	+	Glycosyl transferase, family 2
contig_29	fig|6666666.28487.peg.2549	Protein	276462	277964	+	Phytoene dehydrogenase (EC 1.14.99.-)
contig_29	fig|6666666.28487.peg.2550	Protein	278598	277915	-	CDP-alcohol phosphatidyltransferase
contig_29	fig|6666666.28487.peg.2551	Protein	278632	279426	+	Putative glycosyl transferase
contig_29	fig|6666666.28487.peg.2552	Protein	279494	281137	+	N-6 DNA methylase
contig_29	fig|6666666.28487.peg.2553	Protein	281127	282122	+	FIG00827728: hypothetical protein
contig_29	fig|6666666.28487.peg.2554	Protein	282135	283043	+	Phosphatidylglycerophosphatase B (EC 3.1.3.27)
contig_29	fig|6666666.28487.peg.2555	Protein	283173	283045	-	FIG00830927: hypothetical protein
contig_29	fig|6666666.28487.peg.2556	Protein	284171	283185	-	F420-dependent glucose-6-phosphate dehydrogenase
contig_29	fig|6666666.28487.peg.2557	Protein	284577	285548	+	putative integral membrane protein
contig_29	fig|6666666.28487.peg.2558	Protein	285550	286203	+	PROBABLE CONSERVED MEMBRANE PROTEIN
contig_29	fig|6666666.28487.peg.2559	Protein	286200	287351	+	FIG00818282: hypothetical protein
contig_29	fig|6666666.28487.peg.2560	Protein	287348	288310	+	MoxR-like ATPase
contig_29	fig|6666666.28487.peg.2561	Protein	288310	289632	+	Cell division protein DivIC (FtsB), stabilizes FtsL against RasP cleavage
contig_29	fig|6666666.28487.peg.2562	Protein	290724	289732	-	POSSIBLE CONSERVED TRANSMEMBRANE PROTEIN
contig_29	fig|6666666.28487.peg.2563	Protein	290753	291613	+	Cell division protein DivIC (FtsB), stabilizes FtsL against RasP cleavage
contig_29	fig|6666666.28487.peg.2564	Protein	293014	291635	-	Lignostilbene-alpha,beta-dioxygenase and related enzymes
contig_29	fig|6666666.28487.peg.2565	Protein	293138	293665	+	Transcriptional regulator, PadR family

contig_29	fig|6666666.28487.peg.2566	Protein	293761	294471	+	Transcriptional regulator, PadR family
contig_29	fig|6666666.28487.peg.2567	Protein	294568	295449	+	Oxidoreductase, aldo-keto reductase family
contig_29	fig|6666666.28487.peg.2568	Protein	295520	297154	+	Peptide chain release factor 3
contig_29	fig|6666666.28487.peg.2569	Protein	297648	297160	-	hypothetical protein
contig_29	fig|6666666.28487.peg.2570	Protein	298760	297648	-	DNA-binding protein
contig_29	fig|6666666.28487.peg.2571	Protein	299597	298953	-	putative serine recombinase
contig_29	fig|6666666.28487.peg.2572	Protein	301425	299917	-	Glycerol kinase (EC 2.7.1.30)
contig_29	fig|6666666.28487.peg.2573	Protein	301519	301878	+	FIG00830478: hypothetical protein
contig_29	fig|6666666.28487.peg.2574	Protein	301919	302653	+	FIG00822975: hypothetical protein
contig_29	fig|6666666.28487.peg.2575	Protein	302664	304154	+	FIG00820887: hypothetical protein
contig_29	fig|6666666.28487.peg.2576	Protein	304178	304852	+	Methyltransferase (EC 2.1.1.-)
contig_29	fig|6666666.28487.peg.2577	Protein	306020	304857	-	L-lactate dehydrogenase (EC 1.1.2.3)
contig_29	fig|6666666.28487.peg.2578	Protein	306241	307047	+	FIG00830140: hypothetical protein
contig_29	fig|6666666.28487.peg.2579	Protein	307913	307275	-	DNA-binding response regulator,
contig_29	fig|6666666.28487.peg.2580	Protein	309250	307910	-	FIG00825531: hypothetical protein
contig_29	fig|6666666.28487.peg.2581	Protein	310485	309247	-	Alcohol dehydrogenase (EC 1.1.1.1)
contig_29	fig|6666666.28487.peg.2582	Protein	312089	310569	-	VWA containing CoxE family protein
contig_29	fig|6666666.28487.peg.2583	Protein	313228	312092	-	carbon monoxide dehydrogenase D protein
contig_29	fig|6666666.28487.peg.2584	Protein	314523	313231	-	Alcohol dehydrogenase (EC 1.1.1.1)

contig_29	fig|6666666.28487.peg.2585	Protein	314879	315469	+	tetracycline repressor protein
contig_3	fig|6666666.28487.peg.2586	Protein	240	833	+	protein of unknown function DUF218
contig_3	fig|6666666.28487.peg.2587	Protein	891	1559	+	probable conserved integral membrane protein
contig_3	fig|6666666.28487.peg.2588	Protein	2829	1567	-	PE-PPE, C-terminal domain protein
contig_3	fig|6666666.28487.peg.2589	Protein	3950	3282	-	NLP/P60 family protein
contig_3	fig|6666666.28487.peg.2590	Protein	4602	4105	-	Thiol peroxidase, Tpx-type (EC 1.11.1.15)
contig_3	fig|6666666.28487.peg.2591	Protein	5328	4645	-	FIG00824686: hypothetical protein
contig_3	fig|6666666.28487.peg.2592	Protein	5429	6127	+	Thiaminase II (EC 3.5.99.2)
contig_3	fig|6666666.28487.peg.2593	Protein	6241	7506	+	GTP cyclohydrolase II homolog
contig_3	fig|6666666.28487.peg.2594	Protein	7503	8780	+	Possible pyrimidine-degrading protein DUF1688
contig_3	fig|6666666.28487.peg.2595	Protein	8780	9406	+	phosphoribosyltransferase
contig_3	fig|6666666.28487.peg.2596	Protein	9461	11761	+	Isocitrate lyase (EC 4.1.3.1), group III, Mycobacterial type ICL2
contig_3	fig|6666666.28487.peg.2597	Protein	12911	11907	-	probable transcriptional regulator, AraC family
contig_3	fig|6666666.28487.peg.2598	Protein	14129	13350	-	O-succinylbenzoate-CoA synthase
contig_3	fig|6666666.28487.peg.2599	Protein	14166	14909	+	Phosphoglucosamine mutase (EC 5.4.2.10)
contig_3	fig|6666666.28487.peg.2600	Protein	15287	14913	-	Cyanoglobin; Hemoglobin-like protein HbN
contig_3	fig|6666666.28487.peg.2601	Protein	15684	15391	-	Stress responsive alpha-beta barrel domain protein
contig_3	fig|6666666.28487.peg.2602	Protein	15764	16213	+	Transcriptional regulator, FUR family
contig_3	fig|6666666.28487.peg.2603	Protein	16250	18448	+	Catalase (EC 1.11.1.6) / Peroxidase (EC 1.11.1.7)

contig_3	fig|6666666.28487.peg.2604	Protein	19343	18690	-	POSSIBLE EXPORTED PROTEIN
contig_3	fig|6666666.28487.peg.2605	Protein	20647	19442	-	acyltransferase, putative
contig_3	fig|6666666.28487.peg.2606	Protein	20774	20971	+	hypothetical protein
contig_3	fig|6666666.28487.peg.2607	Protein	20955	22124	+	Response regulator receiver modulated serine phosphatase
contig_3	fig|6666666.28487.peg.2608	Protein	22121	23719	+	Phytochrome, two-component sensor histidine kinase (EC 2.7.3.-); cyanobacterial phytochrome 1
contig_3	fig|6666666.28487.peg.2609	Protein	23716	24156	+	Serine phosphatase RsbU, regulator of sigma subunit
contig_3	fig|6666666.28487.peg.2610	Protein	24158	25294	+	Alkaline phosphodiesterase I (EC 3.1.4.1) / Nucleotide pyrophosphatase (EC 3.6.1.9)
contig_3	fig|6666666.28487.peg.2611	Protein	25564	26175	+	POSSIBLE EXPORTED PROTEIN
contig_3	fig|6666666.28487.peg.2612	Protein	26202	26948	+	UPF0246 protein YaaA
contig_3	fig|6666666.28487.peg.2613	Protein	27456	26956	-	hypothetical protein
contig_3	fig|6666666.28487.peg.2614	Protein	27550	27798	+	PROBABLE MEMBRANE PROTEIN
contig_3	fig|6666666.28487.peg.2615	Protein	27803	28162	+	FIG00832423: hypothetical protein
contig_3	fig|6666666.28487.peg.2616	Protein	28211	29674	+	6-aminohexanoate-cyclic-dimer hydrolase
contig_3	fig|6666666.28487.peg.2617	Protein	29833	30750	+	hydrolase, alpha/beta hydrolase fold family
contig_3	fig|6666666.28487.peg.2618	Protein	30979	30755	-	FIG00821262: hypothetical protein
contig_3	fig|6666666.28487.peg.2619	Protein	32257	30989	-	Molybdopterin binding motif, CinA N-terminal domain / C-terminal domain of CinA type S
contig_3	fig|6666666.28487.peg.2620	Protein	33049	32303	-	3-alpha-(or 20-beta)-hydroxysteroid dehydrogenase (EC 1.1.1.53)

contig_3	fig|6666666.28487.peg.2621	Protein	33142	34206	+	FIG00821133: hypothetical protein
contig_3	fig|6666666.28487.peg.2622	Protein	34653	34207	-	Serine phosphatase RsbU, regulator of sigma subunit
contig_3	fig|6666666.28487.peg.2623	Protein	35091	34699	-	Anti-sigma factor antagonist
contig_3	fig|6666666.28487.peg.2624	Protein	35310	36074	+	Phosphoserine phosphatase (EC 3.1.3.3) / 1-acyl-sn-glycerol-3-phosphate acyltransferase (EC 2.3.1.51)
contig_3	fig|6666666.28487.peg.2625	Protein	36139	37269	+	2-nitropropane dioxygenase (EC 1.13.11.32)
contig_3	fig|6666666.28487.peg.2626	Protein	37557	37321	-	hypothetical protein
contig_3	fig|6666666.28487.peg.2627	Protein	37688	37521	-	hypothetical protein
contig_3	fig|6666666.28487.peg.2628	Protein	37875	38447	+	Transcriptional regulator, TetR family
contig_3	fig|6666666.28487.peg.2629	Protein	38444	40702	+	Formate dehydrogenase-O, major subunit (EC 1.2.1.2)
contig_3	fig|6666666.28487.peg.2630	Protein	41217	40699	-	hypothetical protein
contig_3	fig|6666666.28487.peg.2631	Protein	41383	42636	+	Putative cytochrome P450 125 (EC 1.14.-.-); Putative cytochrome P450 IgrA
contig_3	fig|6666666.28487.peg.2632	Protein	43976	42633	-	hypothetical protein
contig_3	fig|6666666.28487.peg.2633	Protein	44032	44676	+	transcriptional regulator, TetR family
contig_3	fig|6666666.28487.peg.2634	Protein	44977	44666	-	Possible membrane protein
contig_3	fig|6666666.28487.peg.2635	Protein	45066	45842	+	Bll5781 protein
contig_3	fig|6666666.28487.peg.2636	Protein	45903	46325	+	POSSIBLE CONSERVED LIPOPROTEIN LPPE
contig_3	fig|6666666.28487.peg.2637	Protein	47264	46470	-	Putative lipoprotein
contig_3	fig|6666666.28487.peg.2638	Protein	47997	47368	-	Integral membrane protein
contig_30	fig|6666666.28487.peg.2639	Protein	392	3250	+	Glycine dehydrogenase [decarboxylating] (glycine cleavage

						system P protein) (EC 1.4.4.2)
contig_30	fig|6666666.28487.peg.2640	Protein	3271	3993	+	Thioesterase TesA
contig_30	fig|6666666.28487.peg.2641	Protein	4003	4131	+	hypothetical protein
contig_30	fig|6666666.28487.peg.2642	Protein	4548	6329	+	Long-chain-fatty-acid--CoA ligase (EC 6.2.1.3)
contig_30	fig|6666666.28487.peg.2643	Protein	6326	11803	+	Malonyl CoA-acyl carrier protein transacylase (EC 2.3.1.39)
contig_30	fig|6666666.28487.peg.2644	Protein	11904	13634	+	Long-chain-fatty-acid--CoA ligase (EC 6.2.1.3)
contig_30	fig|6666666.28487.peg.2645	Protein	13631	18364	+	Malonyl CoA-acyl carrier protein transacylase (EC 2.3.1.39)
contig_30	fig|6666666.28487.peg.2646	Protein	18370	23808	+	Malonyl CoA-acyl carrier protein transacylase (EC 2.3.1.39)
contig_30	fig|6666666.28487.peg.2647	Protein	23808	29210	+	Phenolpthiocerol synthesis type-I polyketide synthase PpsD
contig_30	fig|6666666.28487.peg.2648	Protein	29207	33625	+	Malonyl CoA-acyl carrier protein transacylase (EC 2.3.1.39)
contig_30	fig|6666666.28487.peg.2649	Protein	33646	34653	+	Daunorubicin-DIM-transport ATP-binding protein ABC transporter DrrA
contig_30	fig|6666666.28487.peg.2650	Protein	34650	35495	+	Daunorubicin-DIM-transport integral membrane protein ABC transporter DrrB
contig_30	fig|6666666.28487.peg.2651	Protein	35492	36337	+	PROBABLE DAUNORUBICIN-DIM-TRANSPORT INTEGRAL MEMBRANE PROTEIN ABC TRANSPORTER DRRC
contig_30	fig|6666666.28487.peg.2652	Protein	36390	37646	+	POSSIBLE CONSERVED POLYKETIDE SYNTHASE ASSOCIATED PROTEIN PAPA5
contig_30	fig|6666666.28487.peg.2653	Protein	38484	37654	-	Tetracenomycin polyketide synthesis O-methyltransferase tcmP (EC 2.1.1.-)
contig_30	fig|6666666.28487.peg.2654	Protein	38583	39230	+	FIG00829405: hypothetical protein

contig_30	fig|6666666.28487.peg.2655	Protein	39240	39746	+	FIG00830829: hypothetical protein
contig_30	fig|6666666.28487.peg.2656	Protein	39739	40443	+	nitroreductase
contig_30	fig|6666666.28487.peg.2657	Protein	41786	40440	-	PE-PPE, C-terminal domain protein
contig_30	fig|6666666.28487.peg.2658	Protein	43418	42246	-	POSSIBLE OXIDOREDUCTASE
contig_30	fig|6666666.28487.peg.2659	Protein	43550	44347	+	FIG00829829: hypothetical protein
contig_30	fig|6666666.28487.peg.2660	Protein	44853	47165	+	FIG00820890: hypothetical protein
contig_30	fig|6666666.28487.peg.2661	Protein	47196	48680	+	Cyclohexanone monooxygenase (EC 1.14.13.22)
contig_30	fig|6666666.28487.peg.2662	Protein	48772	50115	+	Cytochrome P450
contig_30	fig|6666666.28487.peg.2663	Protein	50651	50112	-	Tetracenomycin polyketide synthesis O-methyltransferase tcmP (EC 2.1.1.-)
contig_31	fig|6666666.28487.peg.2664	Protein	79	204	+	hypothetical protein
contig_31	fig|6666666.28487.peg.2665	Protein	260	1822	+	Signal recognition particle, subunit Ffh SRP54 (TC 3.A.5.1.1)
contig_31	fig|6666666.28487.peg.2666	Protein	1822	2892	+	Aminopeptidase YpdF (MP-, MA-, MS-, AP-, NP- specific)
contig_31	fig|6666666.28487.peg.2667	Protein	2894	3553	+	Kynurenine formamidase, bacterial (EC 3.5.1.9)
contig_31	fig|6666666.28487.peg.2668	Protein	4454	3618	-	D-alanyl-D-alanine carboxypeptidase (EC 3.4.16.4)
contig_31	fig|6666666.28487.peg.2669	Protein	5417	4524	-	D-alanyl-D-alanine carboxypeptidase (EC 3.4.16.4)
contig_31	fig|6666666.28487.peg.2670	Protein	5475	5906	+	FIG00821456: hypothetical protein
contig_31	fig|6666666.28487.peg.2671	Protein	6402	5911	-	Bile acid 7-alpha dehydratase BaiE (EC 4.2.1.106)
contig_31	fig|6666666.28487.peg.2672	Protein	6629	7111	+	SSU ribosomal protein S16p
contig_31	fig|6666666.28487.peg.2673	Protein	7119	7361	+	KH domain RNA binding protein YlqC

contig_31	fig|6666666.28487.peg.2674	Protein	7395	7901	+	16S rRNA processing protein RimM
contig_31	fig|6666666.28487.peg.2675	Protein	7914	8606	+	tRNA (Guanine37-N1) -methyltransferase (EC 2.1.1.31)
contig_31	fig|6666666.28487.peg.2676	Protein	9529	8603	-	Putative lipoprotein lppW precursor
contig_31	fig|6666666.28487.peg.2677	Protein	9846	10187	+	LSU ribosomal protein L19p
contig_31	fig|6666666.28487.peg.2678	Protein	10236	11096	+	Signal peptidase I (EC 3.4.21.89)
contig_31	fig|6666666.28487.peg.2679	Protein	11130	11879	+	Ribonuclease HII (EC 3.1.26.4)
contig_31	fig|6666666.28487.peg.2680	Protein	11876	12181	+	Protein often found in Actinomycetes clustered with signal peptidase and/or RNaseHII
contig_31	fig|6666666.28487.peg.2681	Protein	12278	13528	+	Lipase 1 (EC 3.1.1.3)
contig_31	fig|6666666.28487.peg.2682	Protein	14035	13586	-	18 kDa antigen 2
contig_31	fig|6666666.28487.peg.2683	Protein	14271	15044	+	Hypothetical NagD-like phosphatase, Actinobacterial subfamily
contig_31	fig|6666666.28487.peg.2684	Protein	15160	16368	+	CAIB/BAIF family protein
contig_31	fig|6666666.28487.peg.2685	Protein	16365	17186	+	Acyl-CoA thioesterase II (EC 3.1.2.-)
contig_31	fig|6666666.28487.peg.2686	Protein	19644	17146	-	Long-chain-fatty-acid--CoA ligase (EC 6.2.1.3)
contig_31	fig|6666666.28487.peg.2687	Protein	20518	19685	-	Membrane protein, putative
contig_31	fig|6666666.28487.peg.2688	Protein	20657	21028	+	protein of unknown function UPF0102
contig_31	fig|6666666.28487.peg.2689	Protein	21025	22536	+	MG(2+) CHELATASE FAMILY PROTEIN / ComM-related protein
contig_31	fig|6666666.28487.peg.2690	Protein	22536	23663	+	Rossmann fold nucleotide-binding protein Smf possibly involved in DNA uptake

contig_31	fig|6666666.28487.peg.2691	Protein	23728	24582	+	Iron utilization protein
contig_31	fig|6666666.28487.peg.2692	Protein	24619	25779	+	Lactate 2-monooxygenase (EC 1.13.12.4)
contig_31	fig|6666666.28487.peg.2693	Protein	25812	26750	+	Tyrosine recombinase XerC
contig_31	fig|6666666.28487.peg.2694	Protein	27260	26775	-	Membrane proteins related to metalloendopeptidases
contig_31	fig|6666666.28487.peg.2695	Protein	27563	28378	+	SSU ribosomal protein S2p (SAe)
contig_31	fig|6666666.28487.peg.2696	Protein	28411	29226	+	Translation elongation factor Ts
contig_31	fig|6666666.28487.peg.2697	Protein	29572	31059	+	Undecaprenyl-phosphate galactosephosphotransferase (EC 2.7.8.6)
contig_31	fig|6666666.28487.peg.2698	Protein	31115	32521	+	Putative amidase amiC (EC 3.5.1.4)
contig_31	fig|6666666.28487.peg.2699	Protein	32759	32613	-	hypothetical protein
contig_31	fig|6666666.28487.peg.2700	Protein	32638	33825	+	ABC transporter, permease protein, putative
contig_31	fig|6666666.28487.peg.2701	Protein	33898	34395	+	protein of unknown function DUF214
contig_31	fig|6666666.28487.peg.2702	Protein	34400	35125	+	ABC transporter, ATP-binding protein homolog
contig_31	fig|6666666.28487.peg.2703	Protein	35151	36683	+	amino acid permease family protein, putative
contig_31	fig|6666666.28487.peg.2704	Protein	36680	38701	+	Monoamine oxidase (1.4.3.4)
contig_31	fig|6666666.28487.peg.2705	Protein	39206	38790	-	hypothetical protein
contig_31	fig|6666666.28487.peg.2706	Protein	39655	39212	-	hypothetical protein
contig_31	fig|6666666.28487.peg.2707	Protein	39982	40671	+	Uridylate kinase (EC 2.7.4.-)
contig_31	fig|6666666.28487.peg.2708	Protein	40706	41263	+	Ribosome recycling factor
contig_31	fig|6666666.28487.peg.2709	Protein	41266	42129	+	Phosphatidate cytidylyltransferase (EC 2.7.7.41)

contig_31	fig|6666666.28487.peg.2710	Protein	42193	43284	+	Ribosomal RNA large subunit methyltransferase N (EC 2.1.1.-)
contig_31	fig|6666666.28487.peg.2711	Protein	43781	43362	-	FIG172111: hypothetical protein
contig_31	fig|6666666.28487.peg.2712	Protein	45262	43778	-	Aldehyde dehydrogenase (EC 1.2.1.3)
contig_31	fig|6666666.28487.peg.2713	Protein	45848	45303	-	Transcriptional regulator, TetR family
contig_31	fig|6666666.28487.peg.2714	Protein	47072	45930	-	FIG00825607: hypothetical protein
contig_31	fig|6666666.28487.peg.2715	Protein	47154	48344	+	Hydroxyproline dehydratase putative
contig_31	fig|6666666.28487.peg.2716	Protein	50133	48409	-	TesB-like acyl-CoA thioesterase 5
contig_31	fig|6666666.28487.peg.2717	Protein	51009	50170	-	Gluconolactonase (EC 3.1.1.17)
contig_31	fig|6666666.28487.peg.2718	Protein	51103	51813	+	FIG00822996: hypothetical protein
contig_31	fig|6666666.28487.peg.2719	Protein	51813	53027	+	Nikkomycin biosynthesis protein SanQ
contig_31	fig|6666666.28487.peg.2720	Protein	53027	53995	+	alcohol dehydrogenase
contig_31	fig|6666666.28487.peg.2721	Protein	53997	54752	+	2,4-dihydroxyhept-2-ene-1,7-dioic acid aldolase (EC 4.1.2.-)
contig_31	fig|6666666.28487.peg.2722	Protein	54752	55483	+	3-oxoacyl-[acyl-carrier protein] reductase (EC 1.1.1.100)
contig_31	fig|6666666.28487.peg.2723	Protein	55492	57027	+	Cyclohexanone monooxygenase (EC 1.14.13.22)
contig_31	fig|6666666.28487.peg.2724	Protein	57024	57965	+	Probable lipase/esterase
contig_31	fig|6666666.28487.peg.2725	Protein	58439	57957	-	FIG00831752: hypothetical protein
contig_31	fig|6666666.28487.peg.2726	Protein	58529	59668	+	FIG00823256: hypothetical protein
contig_31	fig|6666666.28487.peg.2727	Protein	60697	59729	-	Luciferase family protein
contig_31	fig|6666666.28487.peg.2728	Protein	60751	61671	+	FIG00820695: hypothetical protein

contig_31	fig|6666666.28487.peg.2729	Protein	62691	61678	-	Quinone oxidoreductase (EC 1.6.5.5)
contig_31	fig|6666666.28487.peg.2730	Protein	63357	62869	-	hypothetical protein
contig_31	fig|6666666.28487.peg.2731	Protein	65219	63372	-	3-methylmercaptopropionyl-CoA dehydrogenase (DmdC)
contig_31	fig|6666666.28487.peg.2732	Protein	65205	65318	+	hypothetical protein
contig_31	fig|6666666.28487.peg.2733	Protein	66032	65325	-	FIG00822934: hypothetical protein
contig_31	fig|6666666.28487.peg.2734	Protein	66283	67713	+	Wax ester synthase/acyl-CoA:diacylglycerol acyltransferase
contig_31	fig|6666666.28487.peg.2735	Protein	67858	68436	+	FIG00830888: hypothetical protein
contig_31	fig|6666666.28487.peg.2736	Protein	68738	68484	-	POSSIBLE CONSERVED TRANSMEMBRANE PROTEIN
contig_31	fig|6666666.28487.peg.2737	Protein	68828	69700	+	FIG00825235: hypothetical protein
contig_31	fig|6666666.28487.peg.2738	Protein	71053	69701	-	Deoxyribodipyrimidine photolyase (EC 4.1.99.3)
contig_31	fig|6666666.28487.peg.2739	Protein	71869	71285	-	Cell surface lipoprotein MPT83 precursor
contig_31	fig|6666666.28487.peg.2740	Protein	72591	71911	-	Immunogenic protein Mpt70
contig_31	fig|6666666.28487.peg.2741	Protein	72736	73899	+	1-deoxy-D-xylulose 5-phosphate reductoisomerase (EC 1.1.1.267)
contig_31	fig|6666666.28487.peg.2742	Protein	73914	75152	+	Membrane-associated zinc metalloprotease
contig_31	fig|6666666.28487.peg.2743	Protein	75164	76330	+	1-hydroxy-2-methyl-2-(E)-butenyl 4-diphosphate synthase (EC 1.17.7.1)
contig_31	fig|6666666.28487.peg.2744	Protein	76437	77243	+	GCN5-related N-acetyltransferase, FIGfam019367
contig_31	fig|6666666.28487.peg.2745	Protein	77270	77947	+	FIG00823925: hypothetical protein
contig_31	fig|6666666.28487.peg.2746	Protein	78245	80020	+	Cell division protein FtsI [Peptidoglycan synthetase] (EC 2.4.1.129)

contig_31	fig|6666666.28487.peg.2747	Protein	80070	80678	+	FIG020268: hypothetical protein
contig_31	fig|6666666.28487.peg.2748	Protein	81859	80684	-	FIG00821101: hypothetical protein
contig_31	fig|6666666.28487.peg.2749	Protein	82968	81859	-	FIG00832581: hypothetical protein
contig_31	fig|6666666.28487.peg.2750	Protein	82967	83569	+	Transcriptional regulator, TetR family
contig_31	fig|6666666.28487.peg.2751	Protein	83952	83566	-	Uncharacterized protein, possibly involved in aromatic compounds catabolism
contig_31	fig|6666666.28487.peg.2752	Protein	84017	84832	+	Methionine aminopeptidase (EC 3.4.11.18)
contig_31	fig|6666666.28487.peg.2753	Protein	84950	85594	+	FIG00821571: hypothetical protein
contig_31	fig|6666666.28487.peg.2754	Protein	85668	86219	+	FIG00822818: hypothetical protein
contig_31	fig|6666666.28487.peg.2755	Protein	88026	86227	-	Cyanophycin synthase (EC 6.3.2.29)(EC 6.3.2.30)
contig_31	fig|6666666.28487.peg.2756	Protein	89762	88023	-	Asparagine synthetase [glutamine-hydrolyzing] (EC 6.3.5.4)
contig_31	fig|6666666.28487.peg.2757	Protein	91562	89994	-	Amino acid/metabolite permease in hypothetical Actinobacterial gene cluster; BAT1-like
contig_31	fig|6666666.28487.peg.2758	Protein	91769	93133	+	Glutamine synthetase family protein in hypothetical Actinobacterial gene cluster
contig_31	fig|6666666.28487.peg.2759	Protein	93120	93878	+	COG2071: predicted glutamine amidotransferases in hypothetical Actinobacterial gene cluster
contig_31	fig|6666666.28487.peg.2760	Protein	93887	95257	+	Aldehyde dehydrogenase in hypothetical Actinobacterial gene cluster
contig_31	fig|6666666.28487.peg.2761	Protein	95262	96035	+	Short-chain dehydrogenase/reductase in hypothetical Actinobacterial gene cluster
contig_31	fig|6666666.28487.peg.2762	Protein	96044	96772	+	Transcriptional regulator, GntR family, in hypothetical

						Actinobacterial gene cluster
contig_31	fig|6666666.28487.peg.2763	Protein	97068	98459	+	hypothetical protein
contig_31	fig|6666666.28487.peg.2764	Protein	98575	101466	+	Signal transduction response regulator / Disease resistance domain-containing protein
contig_31	fig|6666666.28487.peg.2765	Protein	101522	104359	+	FIG00683678: hypothetical protein
contig_31	fig|6666666.28487.peg.2766	Protein	104485	105912	+	PE-PGRS FAMILY PROTEIN
contig_31	fig|6666666.28487.peg.2767	Protein	106011	106847	+	putative methyltransferase
contig_31	fig|6666666.28487.peg.2768	Protein	106897	107217	+	regulatory protein, ArsR
contig_31	fig|6666666.28487.peg.2769	Protein	107266	108066	+	Predicted transcriptional regulator of N-Acetylglucosamine utilization, GntR family
contig_31	fig|6666666.28487.peg.2770	Protein	108148	109077	+	Choline kinase (EC 2.7.1.32)
contig_31	fig|6666666.28487.peg.2771	Protein	109074	111539	+	Sarcosine dehydrogenase (EC 1.5.99.1)
contig_31	fig|6666666.28487.peg.2772	Protein	111606	112313	+	Substrate-specific component NikM of nickel ECF transporter
contig_31	fig|6666666.28487.peg.2773	Protein	112310	112678	+	Core component NikM of nickel ECF transporter / Additional substrate-specific component NikN of nickel ECF transporter
contig_31	fig|6666666.28487.peg.2774	Protein	112681	113460	+	Transmembrane component NikQ of energizing module of nickel ECF transporter
contig_31	fig|6666666.28487.peg.2775	Protein	113457	114194	+	ATPase component NikO of energizing module of nickel ECF transporter
contig_31	fig|6666666.28487.peg.2776	Protein	115700	114186	-	Sensor protein basS/pmrB (EC 2.7.3.-)
contig_31	fig|6666666.28487.peg.2777	Protein	116509	115697	-	Two component transcriptional regulator, winged helix family

contig_31	fig|6666666.28487.peg.2778	Protein	116704	117102	+	integral membrane protein
contig_31	fig|6666666.28487.peg.2779	Protein	117223	117810	+	RNA polymerase sigma-E factor
contig_31	fig|6666666.28487.peg.2780	Protein	119345	117933	-	NADPH-dependent mycothiol reductase Mtr
contig_31	fig|6666666.28487.peg.2781	Protein	120375	119338	-	Lysophospholipase (EC 3.1.1.5)
contig_31	fig|6666666.28487.peg.2782	Protein	120514	122070	+	Malate:quinone oxidoreductase (EC 1.1.5.4)
contig_31	fig|6666666.28487.peg.2783	Protein	122087	122533	+	ElaA protein
contig_31	fig|6666666.28487.peg.2784	Protein	122530	124419	+	ChlI component of cobalt chelatase involved in B12 biosynthesis / ChlD component of cobalt chelatase involved in B12 biosynthesis
contig_31	fig|6666666.28487.peg.2785	Protein	124432	125046	+	Cob(I)alamin adenosyltransferase (EC 2.5.1.17)
contig_31	fig|6666666.28487.peg.2786	Protein	125144	126493	+	Cobyrinic acid A,C-diamide synthase
contig_31	fig|6666666.28487.peg.2787	Protein	126490	127704	+	Siroheme synthase / Precorrin-2 oxidase (EC 1.3.1.76) / Sirohydrochlorin ferrochelatase (EC 4.99.1.4) / Uroporphyrinogen-III methyltransferase (EC 2.1.1.107)
contig_31	fig|6666666.28487.peg.2788	Protein	127927	129381	+	EmrB/QacA family drug resistance transporter
contig_32	fig|6666666.28487.peg.2789	Protein	209	2671	+	hypothetical protein
contig_32	fig|6666666.28487.peg.2790	Protein	2887	3756	+	hypothetical protein
contig_32	fig|6666666.28487.peg.2791	Protein	3756	4670	+	peptidase S8 and S53 subtilisin kexin sedolisin
contig_32	fig|6666666.28487.peg.2792	Protein	4692	5393	+	cAMP-binding proteins - catabolite gene activator and regulatory subunit of cAMP-dependent protein kinases
contig_32	fig|6666666.28487.peg.2793	Protein	5611	5886	+	hypothetical protein

contig_32	fig|6666666.28487.peg.2794	Protein	6038	6814	+	thioredoxin family protein
contig_32	fig|6666666.28487.peg.2795	Protein	6933	8975	+	ABC transporter related
contig_32	fig|6666666.28487.peg.2796	Protein	9008	9853	+	TesB-like acyl-CoA thioesterase 4
contig_32	fig|6666666.28487.peg.2797	Protein	10685	9873	-	iron-chelator utilization protein
contig_32	fig|6666666.28487.peg.2798	Protein	11000	10701	-	FIG00830837: hypothetical protein
contig_32	fig|6666666.28487.peg.2799	Protein	11217	12431	+	FOG: Ankyrin repeat
contig_32	fig|6666666.28487.peg.2800	Protein	13383	12409	-	SIS domain protein
contig_32	fig|6666666.28487.peg.2801	Protein	14582	13380	-	hypothetical protein
contig_32	fig|6666666.28487.peg.2802	Protein	15894	14575	-	similar to glycosyltransferase
contig_32	fig|6666666.28487.peg.2803	Protein	15968	16792	+	Non-heme chloroperoxidase (EC 1.11.1.10)
contig_32	fig|6666666.28487.peg.2804	Protein	16912	18336	+	Partial REP13E12 repeat protein
contig_33	fig|6666666.28487.peg.2805	Protein	29	1222	+	putative cytochrome P450 hydroxylase
contig_33	fig|6666666.28487.peg.2806	Protein	1364	2599	+	EAL domain/GGDEF domain protein
contig_33	fig|6666666.28487.peg.2807	Protein	3668	2589	-	Selenocysteine lyase, CsdB
contig_33	fig|6666666.28487.peg.2808	Protein	4644	3682	-	ABC transporter ATP-binding protein
contig_33	fig|6666666.28487.peg.2809	Protein	5297	4647	-	Glutamine amidotransferases class-II
contig_33	fig|6666666.28487.peg.2810	Protein	6618	5329	-	Serine/threonine kinase
contig_33	fig|6666666.28487.peg.2811	Protein	7889	6615	-	Similar to Glutamate--cysteine ligase (EC 6.3.2.2), function unknown
contig_33	fig|6666666.28487.peg.2812	Protein	8644	8003	-	FIG00820305: hypothetical protein

contig_33	fig|6666666.28487.peg.2813	Protein	9072	8641	-	Organic hydroperoxide resistance protein
contig_33	fig|6666666.28487.peg.2814	Protein	10193	9081	-	Putative metal chaperone, involved in Zn homeostasis, GTPase of COG0523 family
contig_33	fig|6666666.28487.peg.2815	Protein	10298	10534	+	LSU ribosomal protein L28p
contig_33	fig|6666666.28487.peg.2816	Protein	10534	10698	+	LSU ribosomal protein L33p @ LSU ribosomal protein L33p, zinc-independent
contig_33	fig|6666666.28487.peg.2817	Protein	10698	11003	+	SSU ribosomal protein S14p (S29e)
contig_33	fig|6666666.28487.peg.2818	Protein	11000	11239	+	SSU ribosomal protein S18p @ SSU ribosomal protein S18p, zinc-independent
contig_33	fig|6666666.28487.peg.2819	Protein	12764	11319	-	Catalase (EC 1.11.1.6)
contig_33	fig|6666666.28487.peg.2820	Protein	13299	12859	-	hypothetical protein
contig_33	fig|6666666.28487.peg.2821	Protein	13433	14257	+	short-chain dehydrogenase
contig_33	fig|6666666.28487.peg.2822	Protein	15376	14303	-	FIG00821722: hypothetical protein
contig_33	fig|6666666.28487.peg.2823	Protein	16451	15417	-	Aspartate-semialdehyde dehydrogenase (EC 1.2.1.11)
contig_33	fig|6666666.28487.peg.2824	Protein	17720	16455	-	Aspartokinase (EC 2.7.2.4)
contig_33	fig|6666666.28487.peg.2825	Protein	17816	18418	+	nitroreductase family protein family
contig_33	fig|6666666.28487.peg.2826	Protein	18479	19339	+	hypothetical protein
contig_33	fig|6666666.28487.peg.2827	Protein	20399	19428	-	Choloylglycine hydrolase (EC 3.5.1.24)
contig_33	fig|6666666.28487.peg.2828	Protein	20576	21835	+	Ammonium transporter
contig_33	fig|6666666.28487.peg.2829	Protein	21943	23259	+	Glutamine synthetase type I (EC 6.3.1.2)
contig_33	fig|6666666.28487.peg.2830	Protein	23293	24201	+	Glutamine amidotransferase protein GlxB (EC 2.4.2.-)

contig_33	fig|6666666.28487.peg.2831	Protein	24189	24905	+	Glutamate synthase [NADPH] putative GlxC chain (EC 1.4.1.13)
contig_33	fig|6666666.28487.peg.2832	Protein	24905	26269	+	Glutamate synthase [NADPH] large chain (EC 1.4.1.13)
contig_33	fig|6666666.28487.peg.2833	Protein	26263	27471	+	Sarcosine oxidase beta subunit (EC 1.5.3.1)
contig_33	fig|6666666.28487.peg.2834	Protein	27483	28133	+	Transcriptional regulator, MerR family
contig_33	fig|6666666.28487.peg.2835	Protein	28294	30114	+	2-isopropylmalate synthase (EC 2.3.3.13)
contig_33	fig|6666666.28487.peg.2836	Protein	30176	30562	+	integral membrane protein
contig_33	fig|6666666.28487.peg.2837	Protein	31569	30568	-	DNA polymerase III epsilon subunit DnaQ (EC 2.7.7.7)
contig_33	fig|6666666.28487.peg.2838	Protein	31648	32904	+	proposed amino acid ligase found clustered with an amidotransferase
contig_33	fig|6666666.28487.peg.2839	Protein	32901	33617	+	Putative amidotransferase similar to cobyric acid synthase
contig_33	fig|6666666.28487.peg.2840	Protein	34309	33656	-	Response regulator containing a CheY-like receiver domain and an HTH DNA-binding domain
contig_33	fig|6666666.28487.peg.2841	Protein	35508	34294	-	sensor kinase, two-component system
contig_33	fig|6666666.28487.peg.2842	Protein	35651	36967	+	putative cytochrome P450 hydroxylase
contig_33	fig|6666666.28487.peg.2843	Protein	37814	36927	-	FIG00821247: hypothetical protein
contig_33	fig|6666666.28487.peg.2844	Protein	38544	37933	-	Recombination protein RecR
contig_33	fig|6666666.28487.peg.2845	Protein	38878	38546	-	FIG000557: hypothetical protein co-occurring with RecR
contig_33	fig|6666666.28487.peg.2846	Protein	39037	39729	+	probable secreted protein
contig_33	fig|6666666.28487.peg.2847	Protein	40184	39726	-	FIG00994252: hypothetical protein
contig_33	fig|6666666.28487.peg.2848	Protein	40278	41657	+	FAD/FMN-containing dehydrogenases

contig_33	fig|6666666.28487.peg.2849	Protein	41654	42976	+	Cyclopropane-fatty-acyl-phospholipid synthase (EC 2.1.1.79)
contig_33	fig|6666666.28487.peg.2850	Protein	44753	42981	-	Catalase (EC 1.11.1.6)
contig_33	fig|6666666.28487.peg.2851	Protein	45123	44722	-	hypothetical protein
contig_33	fig|6666666.28487.peg.2852	Protein	47133	45184	-	DNA polymerase III subunits gamma and tau (EC 2.7.7.7)
contig_33	fig|6666666.28487.peg.2853	Protein	48469	47171	-	Aspartate transaminase (EC 2.6.1.1)
contig_33	fig|6666666.28487.rna.13	RNA	48694	48779	+	tRNA-Ser-GGA
contig_33	fig|6666666.28487.peg.2854	Protein	48850	49620	+	PROBABLE CONSERVED TRANSMEMBRANE PROTEIN
contig_33	fig|6666666.28487.peg.2855	Protein	49673	50686	+	Probable secreted serine protease
contig_33	fig|6666666.28487.peg.2856	Protein	50736	51806	+	FIG00830617: hypothetical protein
contig_33	fig|6666666.28487.peg.2857	Protein	51816	52682	+	Permease of the drug/metabolite transporter (DMT) superfamily
contig_33	fig|6666666.28487.peg.2858	Protein	52709	53350	+	hypothetical protein
contig_33	fig|6666666.28487.peg.2859	Protein	54096	53347	-	predicted thioesterase
contig_33	fig|6666666.28487.peg.2860	Protein	54572	54093	-	FIG00820354: hypothetical protein
contig_33	fig|6666666.28487.peg.2861	Protein	55684	54632	-	ATP-dependent DNA ligase (EC 6.5.1.1)
contig_33	fig|6666666.28487.peg.2862	Protein	56730	55687	-	ATP-dependent DNA ligase (EC 6.5.1.1) LigC
contig_33	fig|6666666.28487.peg.2863	Protein	58167	56800	-	TRAP-type C4-dicarboxylate transport system, large permease component
contig_33	fig|6666666.28487.peg.2864	Protein	58682	58164	-	TRAP dicarboxylate transporter, DctQ subunit
contig_33	fig|6666666.28487.peg.2865	Protein	59758	58679	-	TRAP-type C4-dicarboxylate transport system, periplasmic component

contig_33	fig|6666666.28487.peg.2866	Protein	60557	59760	-	possible amidohydrolase, carbon-nitrogen hydrolase family protein
contig_33	fig|6666666.28487.peg.2867	Protein	61351	60554	-	putative hydroxyacid aldolase
contig_33	fig|6666666.28487.peg.2868	Protein	61431	62120	+	transcriptional regulator, GntR family protein
contig_33	fig|6666666.28487.peg.2869	Protein	63278	62181	-	Heat shock protein HtrA
contig_33	fig|6666666.28487.peg.2870	Protein	63598	64782	+	Beta-lactamase class C and other penicillin binding proteins
contig_33	fig|6666666.28487.peg.2871	Protein	64836	66257	+	Lysine decarboxylase (EC 4.1.1.18)
contig_33	fig|6666666.28487.peg.2872	Protein	66292	67356	+	ATP-dependent DNA ligase (EC 6.5.1.1) LigC
contig_33	fig|6666666.28487.peg.2873	Protein	67416	69815	+	Arylsulfatase (EC 3.1.6.1)
contig_33	fig|6666666.28487.peg.2874	Protein	69893	70768	+	FIG00825713: hypothetical protein
contig_33	fig|6666666.28487.peg.2875	Protein	70972	71619	+	metallo-beta-lactamase superfamily protein
contig_33	fig|6666666.28487.peg.2876	Protein	73312	71600	-	Propanediol dehydratase reactivation factor large subunit
contig_33	fig|6666666.28487.peg.2877	Protein	73638	73309	-	Probable cis-diol dehydratase small subunit (similar to propanediol and glycerol dehydratases)
contig_33	fig|6666666.28487.peg.2878	Protein	75887	73635	-	Probable cis-diol dehydratase large subunit (similar to propanediol and glycerol dehydratases)
contig_33	fig|6666666.28487.peg.2879	Protein	77442	75913	-	amino acid permease family protein
contig_33	fig|6666666.28487.peg.2880	Protein	77650	78930	+	Aminotransferase class-III (EC 2.6.1.40)
contig_33	fig|6666666.28487.peg.2881	Protein	78927	80483	+	Alcohol dehydrogenase (EC 1.1.1.1); Acetaldehyde dehydrogenase (EC 1.2.1.10)
contig_33	fig|6666666.28487.peg.2882	Protein	80487	81104	+	FIG00822031: hypothetical protein

contig_33	fig|6666666.28487.peg.2883	Protein	81101	81598	+	FIG00828164: hypothetical protein
contig_33	fig|6666666.28487.peg.2884	Protein	81595	81840	+	carbon dioxide concentrating mechanism protein CcmL, putative
contig_33	fig|6666666.28487.peg.2885	Protein	81907	82188	+	microcompartment protein
contig_33	fig|6666666.28487.peg.2886	Protein	82198	82851	+	hypothetical protein
contig_33	fig|6666666.28487.peg.2887	Protein	82848	83849	+	Aminoglycoside phosphotransferase
contig_33	fig|6666666.28487.peg.2888	Protein	83867	84646	+	3-oxoacyl-[acyl-carrier protein] reductase (EC 1.1.1.100)
contig_33	fig|6666666.28487.peg.2889	Protein	84699	85466	+	Predicted transcriptional regulator of N-Acetylglucosamine utilization, GntR family
contig_33	fig|6666666.28487.peg.2890	Protein	85492	86379	+	glutamyl-Q-tRNA synthetase
contig_33	fig|6666666.28487.peg.2891	Protein	86424	87890	+	amino acid ABC transporter, permease protein
contig_33	fig|6666666.28487.peg.2892	Protein	87887	88657	+	amino acid ABC transporter, ATP-binding protein (glnQ)
contig_33	fig|6666666.28487.peg.2893	Protein	89040	88669	-	Thioredoxin
contig_33	fig|6666666.28487.peg.2894	Protein	90270	89071	-	Oxidoreductase (flavoprotein)
contig_33	fig|6666666.28487.peg.2895	Protein	91662	90283	-	Metallo-beta-lactamase family protein
contig_33	fig|6666666.28487.peg.2896	Protein	91865	92449	+	Sulfur carrier protein adenylyltransferase ThiF
contig_33	fig|6666666.28487.peg.2897	Protein	92455	93330	+	FIG00829050: hypothetical protein
contig_33	fig|6666666.28487.peg.2898	Protein	93327	93596	+	hypothetical protein
contig_33	fig|6666666.28487.peg.2899	Protein	93618	94973	+	Hydroxyacylglutathione hydrolase (EC 3.1.2.6)
contig_33	fig|6666666.28487.peg.2900	Protein	94995	96356	+	Amine oxidase [flavin-containing] A (EC 1.4.3.4)
contig_33	fig|6666666.28487.peg.2901	Protein	97249	96353	-	Dehydrogenases with different specificities (related to short-

						chain alcohol dehydrogenases)
contig_33	fig|6666666.28487.peg.2902	Protein	98027	97263	-	Short-chain dehydrodenase (gene dltE)
contig_33	fig|6666666.28487.peg.2903	Protein	98109	99041	+	transcriptional regulator, LysR family protein
contig_33	fig|6666666.28487.peg.2904	Protein	100266	99031	-	biotin biosynthesis cytochrome P450 (EC 1.4.-.-)
contig_33	fig|6666666.28487.peg.2905	Protein	101623	100367	-	tRNA-guanine transglycosylase (EC 2.4.2.29)
contig_33	fig|6666666.28487.peg.2906	Protein	101647	102069	+	FIG00830586: hypothetical protein
contig_33	fig|6666666.28487.peg.2907	Protein	102144	102602	+	19 kDa lipoprotein antigen precursor LpqH
contig_33	fig|6666666.28487.peg.2908	Protein	102959	102615	-	PROBABLE CONSERVED MEMBRANE PROTEIN
contig_33	fig|6666666.28487.peg.2909	Protein	103051	103419	+	FIG00822057: hypothetical protein
contig_33	fig|6666666.28487.peg.2910	Protein	104486	103422	-	FIG00826345: hypothetical protein
contig_33	fig|6666666.28487.peg.2911	Protein	104506	105435	+	Enoyl-[acyl-carrier-protein] reductase [FMN] (EC 1.3.1.9)
contig_33	fig|6666666.28487.peg.2912	Protein	105466	105819	+	FIG00831599: hypothetical protein
contig_33	fig|6666666.28487.peg.2913	Protein	105829	106491	+	Arylesterase
contig_33	fig|6666666.28487.peg.2914	Protein	107255	106497	-	Transcriptional repressor of the fructose operon, DeoR family
contig_33	fig|6666666.28487.peg.2915	Protein	109253	107265	-	PTS system, mannitol-specific IIC component (EC 2.7.1.69) / PTS system, mannitol-specific IIB component (EC 2.7.1.69) / PTS system, mannitol-specific IIA component (EC 2.7.1.69)
contig_33	fig|6666666.28487.peg.2916	Protein	109449	110534	+	Sorbitol dehydrogenase (EC 1.1.1.14)
contig_33	fig|6666666.28487.peg.2917	Protein	110646	111830	+	hypothetical protein
contig_33	fig|6666666.28487.peg.2918	Protein	112149	113096	+	pyruvate dehydrogenase (lipoamide)(EC:1.2.4.1)
contig_33	fig|6666666.28487.peg.2919	Protein	113093	114082	+	Pyruvate dehydrogenase E1 component beta subunit (EC

						1.2.4.1)
contig_33	fig|6666666.28487.peg.2920	Protein	114079	114321	+	hypothetical protein
contig_33	fig|6666666.28487.peg.2921	Protein	114359	114586	+	hypothetical protein
contig_33	fig|6666666.28487.peg.2922	Protein	114583	115521	+	3-oxoacyl-[acyl-carrier-protein] synthase, KASIII (EC 2.3.1.41)
contig_33	fig|6666666.28487.peg.2923	Protein	115577	116239	+	Enoyl-[acyl-carrier-protein] reductase [NADPH] (EC 1.3.1.10)
contig_33	fig|6666666.28487.peg.2924	Protein	116317	116490	+	hypothetical protein
contig_33	fig|6666666.28487.rna.14	RNA	116633	116546	-	tRNA-Ser-CGA
contig_33	fig|6666666.28487.peg.2925	Protein	117148	116762	-	tRNA-specific adenosine-34 deaminase (EC 3.5.4.-)
contig_33	fig|6666666.28487.peg.2926	Protein	117758	117234	-	FIG00994171: hypothetical protein
contig_33	fig|6666666.28487.peg.2927	Protein	117832	118776	+	Arogenate dehydrogenase (EC 1.3.1.43)
contig_33	fig|6666666.28487.peg.2928	Protein	119260	118739	-	FIG00998044: hypothetical protein
contig_33	fig|6666666.28487.peg.2929	Protein	120114	119332	-	Glycine betaine ABC transport system permease protein
contig_33	fig|6666666.28487.peg.2930	Protein	120788	120111	-	L-proline glycine betaine ABC transport system permease protein ProW (TC 3.A.1.12.1)
contig_33	fig|6666666.28487.peg.2931	Protein	121905	120796	-	L-proline glycine betaine ABC transport system permease protein ProV (TC 3.A.1.12.1)
contig_33	fig|6666666.28487.peg.2932	Protein	122869	121919	-	L-proline glycine betaine binding ABC transporter protein ProX (TC 3.A.1.12.1)
contig_33	fig|6666666.28487.peg.2933	Protein	122952	123305	+	Cell division protein DivIC (FtsB), stabilizes FtsL against RasP cleavage
contig_33	fig|6666666.28487.peg.2934	Protein	124122	123310	-	Transglutaminase-like domain protein

contig_33	fig|6666666.28487.peg.2935	Protein	125252	124230	-	Acyl-CoA dehydrogenase FadE36 (EC 1.3.99.-)
contig_33	fig|6666666.28487.peg.2936	Protein	125907	125299	-	FIG00824627: hypothetical protein
contig_33	fig|6666666.28487.peg.2937	Protein	125926	126690	+	Oxidoreductase, short-chain dehydrogenase/reductase family
contig_33	fig|6666666.28487.peg.2938	Protein	126859	126698	-	protein of unknown function DUF222
contig_33	fig|6666666.28487.peg.2939	Protein	127959	126889	-	Transcriptional regulator, IclR family
contig_33	fig|6666666.28487.peg.2940	Protein	129912	128005	-	alkyl sulfatase (EC 3.1.6.-)
contig_33	fig|6666666.28487.peg.2941	Protein	130027	130530	+	Cupin 2, conserved barrel domain protein
contig_33	fig|6666666.28487.peg.2942	Protein	130608	131234	+	Conjugative transfer protein TrbL
contig_33	fig|6666666.28487.peg.2943	Protein	131231	131521	+	putative EsaT-6 like protein 12 (hypothetical alanine rich protein)
contig_33	fig|6666666.28487.peg.2944	Protein	131540	134023	+	hypothetical protein
contig_33	fig|6666666.28487.peg.2945	Protein	134921	135070	+	hypothetical protein
contig_33	fig|6666666.28487.peg.2946	Protein	135134	135331	+	hypothetical protein
contig_33	fig|6666666.28487.peg.2947	Protein	135324	136136	+	hypothetical protein
contig_33	fig|6666666.28487.peg.2948	Protein	136245	136129	-	hypothetical protein
contig_33	fig|6666666.28487.peg.2949	Protein	136270	136449	+	hypothetical protein
contig_33	fig|6666666.28487.peg.2950	Protein	136437	136874	+	hypothetical protein
contig_33	fig|6666666.28487.peg.2951	Protein	137269	137625	+	hypothetical protein
contig_33	fig|6666666.28487.peg.2952	Protein	138188	138394	+	hypothetical protein
contig_33	fig|6666666.28487.peg.2953	Protein	139321	138401	-	hypothetical protein

contig_33	fig|6666666.28487.peg.2954	Protein	140248	139406	-	dehydrogenase, putative
contig_33	fig|6666666.28487.peg.2955	Protein	140440	140778	+	Transcriptional regulator, HxlR family
contig_33	fig|6666666.28487.peg.2956	Protein	141191	140775	-	FIG172205: hypothetical protein
contig_33	fig|6666666.28487.peg.2957	Protein	141687	141184	-	Phosphoesterase
contig_33	fig|6666666.28487.peg.2958	Protein	142116	141697	-	Endoribonuclease L-PSP
contig_33	fig|6666666.28487.peg.2959	Protein	142712	142113	-	hypothetical protein
contig_33	fig|6666666.28487.peg.2960	Protein	144522	142780	-	Cytochrome c oxidase polypeptide I (EC 1.9.3.1)
contig_33	fig|6666666.28487.rna.15	RNA	144660	144588	-	tRNA-Arg-ACG
contig_33	fig|6666666.28487.rna.16	RNA	144822	144734	-	tRNA-Pseudo-GCT
contig_33	fig|6666666.28487.peg.2961	Protein	144907	145965	+	Biosynthetic Aromatic amino acid aminotransferase beta (EC 2.6.1.57)
contig_33	fig|6666666.28487.peg.2962	Protein	145962	146432	+	FIG00820948: hypothetical protein
contig_33	fig|6666666.28487.peg.2963	Protein	146463	146906	+	FIG00824694: hypothetical protein
contig_33	fig|6666666.28487.peg.2964	Protein	146923	147744	+	Enoyl-CoA hydratase
contig_33	fig|6666666.28487.peg.2965	Protein	148978	147731	-	Permeases of the major facilitator superfamily
contig_33	fig|6666666.28487.rna.17	RNA	149290	149204	-	tRNA-Ser-TGA
contig_33	fig|6666666.28487.peg.2966	Protein	149768	149301	-	Transcriptional regulator, MarR family
contig_33	fig|6666666.28487.peg.2967	Protein	149893	150867	+	Quinone oxidoreductase (EC 1.6.5.5)
contig_34	fig|6666666.28487.rna.18	RNA	94	22	-	tRNA-Arg-CCT
contig_34	fig|6666666.28487.peg.2968	Protein	154	918	+	FIG00826007: hypothetical protein

contig_34	fig|6666666.28487.peg.2969	Protein	915	1499	+	Cytochrome c oxidase polypeptide III (EC 1.9.3.1)
contig_34	fig|6666666.28487.peg.2970	Protein	1499	1768	+	FIG00829905: hypothetical protein
contig_34	fig|6666666.28487.peg.2971	Protein	2589	1765	-	putative Rif11 homolog
contig_34	fig|6666666.28487.peg.2972	Protein	2621	3817	+	Putative cytoplasmic protein
contig_34	fig|6666666.28487.peg.2973	Protein	4611	3889	-	FIG111991: hypothetical protein
contig_34	fig|6666666.28487.peg.2974	Protein	5258	4608	-	RNA polymerase sigma-70 factor
contig_34	fig|6666666.28487.peg.2975	Protein	6033	5290	-	Hypothetical protein COG3496
contig_34	fig|6666666.28487.peg.2976	Protein	7246	6155	-	D-alanyl-D-alanine carboxypeptidase (EC 3.4.16.4)
contig_34	fig|6666666.28487.peg.2977	Protein	8316	7246	-	FIG00821379: hypothetical protein
contig_34	fig|6666666.28487.peg.2978	Protein	9543	8317	-	Manganese transport protein MntH
contig_34	fig|6666666.28487.peg.2979	Protein	9877	9638	-	hypothetical protein
contig_34	fig|6666666.28487.peg.2980	Protein	10149	9901	-	FIG01277535: hypothetical protein
contig_34	fig|6666666.28487.peg.2981	Protein	10537	10292	-	hypothetical protein
contig_34	fig|6666666.28487.peg.2982	Protein	11647	10559	-	FIG00821176: hypothetical protein
contig_34	fig|6666666.28487.peg.2983	Protein	12448	11657	-	7-alpha-hydroxysteroid dehydrogenase (EC 1.1.1.159)
contig_34	fig|6666666.28487.peg.2984	Protein	13589	12639	-	Ku domain protein
contig_34	fig|6666666.28487.peg.2985	Protein	14542	13589	-	Fructokinase (EC 2.7.1.4)
contig_34	fig|6666666.28487.peg.2986	Protein	14566	16830	+	ATP-dependent DNA ligase (EC 6.5.1.1) clustered with Ku protein, LigD
contig_34	fig|6666666.28487.peg.2987	Protein	17180	17533	+	hypothetical protein

contig_35	fig|6666666.28487.peg.2988	Protein	58	1461	+	Xylulose kinase (EC 2.7.1.17)
contig_35	fig|6666666.28487.peg.2989	Protein	1478	2800	+	Sugar ABC transporter, periplasmic sugar-binding protein
contig_35	fig|6666666.28487.peg.2990	Protein	2851	3723	+	ABC-type sugar transport systems, permease components
contig_35	fig|6666666.28487.peg.2991	Protein	3720	4535	+	ABC-type sugar transport system, permease component
contig_35	fig|6666666.28487.peg.2992	Protein	4558	5616	+	Glycerol-3-phosphate ABC transporter, ATP-binding protein UgpC (TC 3.A.1.1.3)
contig_35	fig|6666666.28487.peg.2993	Protein	5645	6415	+	3-oxoacyl-[acyl-carrier protein] reductase (EC 1.1.1.100)
contig_35	fig|6666666.28487.peg.2994	Protein	6460	7281	+	FIG00826071: hypothetical protein
contig_35	fig|6666666.28487.peg.2995	Protein	7829	7353	-	Transcriptional regulator, HxlR family
contig_35	fig|6666666.28487.peg.2996	Protein	7920	9119	+	3-ketoacyl-CoA thiolase (EC 2.3.1.16) @ Acetyl-CoA acetyltransferase (EC 2.3.1.9)
contig_35	fig|6666666.28487.peg.2997	Protein	9625	9176	-	AclJ
contig_35	fig|6666666.28487.peg.2998	Protein	10620	9688	-	putative conserved transmembrane protein
contig_35	fig|6666666.28487.peg.2999	Protein	12431	10659	-	Aspartyl-tRNA synthetase (EC 6.1.1.12) @ Aspartyl-tRNA(Asn) synthetase (EC 6.1.1.23)
contig_35	fig|6666666.28487.peg.3000	Protein	12578	13087	+	FIG00823370: hypothetical protein
contig_35	fig|6666666.28487.peg.3001	Protein	14571	13084	-	Probable carboxylesterase LipT (EC 3.1.1.-)
contig_35	fig|6666666.28487.peg.3002	Protein	14649	15545	+	YpfJ protein, zinc metalloprotease superfamily
contig_35	fig|6666666.28487.peg.3003	Protein	15538	17166	+	FIG00826891: hypothetical protein
contig_35	fig|6666666.28487.peg.3004	Protein	18035	17163	-	Acyl-CoA thioesterase II (EC 3.1.2.-)
contig_35	fig|6666666.28487.peg.3005	Protein	18212	18090	-	hypothetical protein

contig_35	fig|6666666.28487.peg.3006	Protein	18145	18762	+	Transcriptional regulator, TetR family
contig_35	fig|6666666.28487.peg.3007	Protein	18882	20111	+	BarH
contig_35	fig|6666666.28487.peg.3008	Protein	20129	21265	+	Acyl-CoA dehydrogenase, short-chain specific (EC 1.3.99.2)
contig_35	fig|6666666.28487.peg.3009	Protein	21262	22536	+	FIG00822014: hypothetical protein
contig_35	fig|6666666.28487.peg.3010	Protein	23601	22567	-	Transporter
contig_35	fig|6666666.28487.peg.3011	Protein	23669	25882	+	ATP-dependent DNA helicase rep (EC 3.6.1.-)
contig_35	fig|6666666.28487.peg.3012	Protein	26267	25884	-	FIG172108: hypothetical protein
contig_35	fig|6666666.28487.peg.3013	Protein	26643	27851	+	Urea ABC transporter, substrate binding protein UrtA
contig_35	fig|6666666.28487.peg.3014	Protein	27889	28773	+	Urea ABC transporter, permease protein UrtB
contig_35	fig|6666666.28487.peg.3015	Protein	28770	29882	+	Urea ABC transporter, permease protein UrtC
contig_35	fig|6666666.28487.peg.3016	Protein	29879	30712	+	Urea ABC transporter, ATPase protein UrtD
contig_35	fig|6666666.28487.peg.3017	Protein	30712	31404	+	Urea ABC transporter, ATPase protein UrtE
contig_35	fig|6666666.28487.peg.3018	Protein	34386	31498	-	FIG00824761: hypothetical protein
contig_35	fig|6666666.28487.peg.3019	Protein	35836	34844	-	hypothetical protein
contig_35	fig|6666666.28487.peg.3020	Protein	35908	36450	+	hypothetical protein
contig_35	fig|6666666.28487.peg.3021	Protein	37366	36455	-	hypothetical protein
contig_35	fig|6666666.28487.peg.3022	Protein	38774	37512	-	Histidyl-tRNA synthetase (EC 6.1.1.21)
contig_35	fig|6666666.28487.peg.3023	Protein	39516	38767	-	Hydroxyacylglutathione hydrolase (EC 3.1.2.6)
contig_35	fig|6666666.28487.peg.3024	Protein	39565	40455	+	Peptidyl-prolyl cis-trans isomerase (EC 5.2.1.8)

contig_35	fig|6666666.28487.peg.3025	Protein	40461	41474	+	Peptidyl-prolyl cis-trans isomerase (EC 5.2.1.8)
contig_35	fig|6666666.28487.peg.3026	Protein	43870	41498	-	GTP pyrophosphokinase (EC 2.7.6.5), (p)ppGpp synthetase I
contig_35	fig|6666666.28487.peg.3027	Protein	44443	43910	-	Adenine phosphoribosyltransferase (EC 2.4.2.7)
contig_35	fig|6666666.28487.peg.3028	Protein	46089	44440	-	Oligopeptide ABC transporter, periplasmic oligopeptide-binding protein OppA (TC 3.A.1.5.1)
contig_35	fig|6666666.28487.peg.3029	Protein	47352	46096	-	Protein-export membrane protein SecF (TC 3.A.5.1.1)
contig_35	fig|6666666.28487.peg.3030	Protein	49143	47356	-	Protein-export membrane protein SecD (TC 3.A.5.1.1)
contig_35	fig|6666666.28487.peg.3031	Protein	49552	49208	-	Preprotein translocase subunit YajC (TC 3.A.5.1.1)
contig_35	fig|6666666.28487.peg.3032	Protein	49948	51291	+	Gamma-aminobutyrate:alpha-ketoglutarate aminotransferase (EC 2.6.1.19)
contig_35	fig|6666666.28487.peg.3033	Protein	51879	51550	-	hypothetical protein
contig_35	fig|6666666.28487.peg.3034	Protein	52235	55723	+	Long-chain-fatty-acid--CoA ligase (EC 6.2.1.3)
contig_35	fig|6666666.28487.peg.3035	Protein	56322	55720	-	FIG00825396: hypothetical protein
contig_35	fig|6666666.28487.peg.3036	Protein	56816	56430	-	FIG00822359: hypothetical protein
contig_35	fig|6666666.28487.peg.3037	Protein	57901	56834	-	Holliday junction DNA helicase RuvB
contig_35	fig|6666666.28487.peg.3038	Protein	58492	57905	-	Holliday junction DNA helicase RuvA
contig_35	fig|6666666.28487.peg.3039	Protein	59079	58489	-	Crossover junction endodeoxyribonuclease RuvC (EC 3.1.22.4)
contig_35	fig|6666666.28487.peg.3040	Protein	59452	59156	-	hypothetical protein
contig_35	fig|6666666.28487.peg.3041	Protein	60166	59552	-	Transcriptional regulator, TetR family
contig_35	fig|6666666.28487.peg.3042	Protein	60238	61074	+	PROBABLE OXIDOREDUCTASE

contig_35	fig|6666666.28487.peg.3043	Protein	61408	61058	-	FIG00824624: hypothetical protein
contig_35	fig|6666666.28487.peg.3044	Protein	62212	61457	-	FIG000859: hypothetical protein
contig_35	fig|6666666.28487.peg.3045	Protein	62913	62320	-	Transcriptional regulator, TetR family
contig_35	fig|6666666.28487.peg.3046	Protein	62976	64700	+	FIG00828524: hypothetical protein
contig_35	fig|6666666.28487.peg.3047	Protein	64770	65981	+	FadE25_2
contig_35	fig|6666666.28487.peg.3048	Protein	66583	65996	-	Pyridoxine biosynthesis glutamine amidotransferase, glutaminase subunit (EC 2.4.2.-)
contig_35	fig|6666666.28487.peg.3049	Protein	67428	66580	-	Acyl-CoA thioesterase II (EC 3.1.2.-)
contig_35	fig|6666666.28487.peg.3050	Protein	68377	67472	-	Pyridoxine biosynthesis glutamine amidotransferase, synthase subunit (EC 2.4.2.-)
contig_35	fig|6666666.28487.peg.3051	Protein	69486	68443	-	FIG019327: Probable conserved membrane protein
contig_35	fig|6666666.28487.peg.3052	Protein	70601	69483	-	Phosphatidylinositol alpha-mannosyltransferase (EC 2.4.1.57)
contig_35	fig|6666666.28487.peg.3053	Protein	71574	70618	-	Lauroyl/myristoyl acyltransferase involved in lipid A biosynthesis (Lauroyl/myristoyl acyltransferase)
contig_35	fig|6666666.28487.peg.3054	Protein	72239	71571	-	CDP-diacylglycerol--glycerol-3-phosphate 3-phosphatidyltransferase (EC 2.7.8.5)
contig_35	fig|6666666.28487.peg.3055	Protein	72784	72236	-	FIG049476: HIT family protein
contig_35	fig|6666666.28487.peg.3056	Protein	74820	72787	-	Threonyl-tRNA synthetase (EC 6.1.1.3)
contig_35	fig|6666666.28487.peg.3057	Protein	75364	74936	-	FIG00820131: hypothetical protein
contig_35	fig|6666666.28487.peg.3058	Protein	75990	75361	-	FIG00823977: hypothetical protein
contig_35	fig|6666666.28487.peg.3059	Protein	76863	76012	-	Aldo-keto reductase

contig_35	fig|6666666.28487.peg.3060	Protein	76901	77398	+	FIG037376: hypothetical protein
contig_35	fig|6666666.28487.peg.3061	Protein	77486	78229	+	FIG00821996: hypothetical protein
contig_35	fig|6666666.28487.peg.3062	Protein	81223	78245	-	hypothetical protein
contig_35	fig|6666666.28487.peg.3063	Protein	81174	81344	+	hypothetical protein
contig_35	fig|6666666.28487.peg.3064	Protein	81450	81599	+	hypothetical protein
contig_35	fig|6666666.28487.peg.3065	Protein	83491	81770	-	Two component system sensor histidine kinase DevS
contig_35	fig|6666666.28487.peg.3066	Protein	83596	84591	+	Dinucleotide-utilizing enzymes involved in molybdopterin and thiamine biosynthesis family 2
contig_35	fig|6666666.28487.peg.3067	Protein	84585	85406	+	Universal stress protein family
contig_35	fig|6666666.28487.peg.3068	Protein	85440	86084	+	Two component transcriptional regulatory protein DevR
contig_35	fig|6666666.28487.peg.3069	Protein	86959	86081	-	Dinucleotide-utilizing enzymes involved in molybdopterin and thiamine biosynthesis family 2
contig_35	fig|6666666.28487.peg.3070	Protein	88765	87113	-	Alpha-glucosidase (EC 3.2.1.20)
contig_35	fig|6666666.28487.peg.3071	Protein	88897	89919	+	MSM (multiple sugar metabolism) operon regulatory protein
contig_35	fig|6666666.28487.peg.3072	Protein	90045	91355	+	N-Acetyl-D-glucosamine ABC transport system, sugar-binding protein
contig_35	fig|6666666.28487.peg.3073	Protein	91352	92314	+	Maltose/maltodextrin ABC transporter, permease protein MalF
contig_35	fig|6666666.28487.peg.3074	Protein	92311	93138	+	Maltose/maltodextrin ABC transporter, permease protein MalG
contig_35	fig|6666666.28487.peg.3075	Protein	93143	94342	+	Maltose/maltodextrin transport ATP-binding protein MalK (EC 3.6.3.19)

contig_35	fig|6666666.28487.peg.3076	Protein	94511	95644	+	Chromosome segregation ATPases
contig_35	fig|6666666.28487.peg.3077	Protein	96024	95641	-	anti-anti-sigma factor
contig_35	fig|6666666.28487.peg.3078	Protein	96244	96618	+	Molybdate-binding domain of ModE
contig_35	fig|6666666.28487.peg.3079	Protein	96615	97379	+	Molybdenum ABC transporter, periplasmic molybdenum-binding protein ModA (TC 3.A.1.8.1)
contig_35	fig|6666666.28487.peg.3080	Protein	97376	98182	+	Molybdenum transport system permease protein ModB (TC 3.A.1.8.1)
contig_35	fig|6666666.28487.peg.3081	Protein	98179	99273	+	Molybdenum transport ATP-binding protein ModC (TC 3.A.1.8.1)
contig_35	fig|6666666.28487.peg.3082	Protein	101331	99337	-	Acetoacetyl-CoA synthetase (EC 6.2.1.16)
contig_35	fig|6666666.28487.peg.3083	Protein	102913	101600	-	PPE family protein
contig_35	fig|6666666.28487.peg.3084	Protein	102977	103144	+	hypothetical protein
contig_35	fig|6666666.28487.peg.3085	Protein	104314	103259	-	Ribonuclease BN (EC 3.1.-.-)
contig_35	fig|6666666.28487.peg.3086	Protein	104476	104631	+	hypothetical protein
contig_35	fig|6666666.28487.peg.3087	Protein	105326	106057	+	RNA polymerase sigma factor SigB
contig_35	fig|6666666.28487.peg.3088	Protein	106295	107068	+	RNA polymerase sigma factor SigB
contig_35	fig|6666666.28487.peg.3089	Protein	107249	108487	+	COG0438: Glycosyltransferase
contig_35	fig|6666666.28487.peg.3090	Protein	108504	109331	+	hypothetical protein
contig_35	fig|6666666.28487.peg.3091	Protein	109340	110152	+	hypothetical protein
contig_35	fig|6666666.28487.peg.3092	Protein	110205	110339	+	hypothetical protein
contig_35	fig|6666666.28487.peg.3093	Protein	110349	110789	+	Serine phosphatase RsbU, regulator of sigma subunit

contig_35	fig|6666666.28487.peg.3094	Protein	111172	111528	+	Anti-sigma F factor antagonist (spoIIAA-2); Anti-sigma B factor antagonist RsbV
contig_35	fig|6666666.28487.peg.3095	Protein	111586	111864	+	hypothetical protein
contig_35	fig|6666666.28487.peg.3096	Protein	111963	112202	+	hypothetical protein
contig_35	fig|6666666.28487.peg.3097	Protein	112635	113312	+	response regulator receiver and ANTAR domain protein
contig_35	fig|6666666.28487.peg.3098	Protein	113432	114343	+	Bll2791 protein
contig_35	fig|6666666.28487.peg.3099	Protein	114444	115208	+	putative secreted protein
contig_35	fig|6666666.28487.peg.3100	Protein	115522	118092	+	regulatory protein, LuxR
contig_35	fig|6666666.28487.peg.3101	Protein	118182	118847	+	Oxygen-insensitive NADPH nitroreductase (EC 1.-.-.-)
contig_35	fig|6666666.28487.peg.3102	Protein	119001	119609	+	RNA polymerase sigma-70 factor, family protein
contig_35	fig|6666666.28487.peg.3103	Protein	119731	120033	+	hypothetical protein
contig_35	fig|6666666.28487.peg.3104	Protein	120198	120464	+	hypothetical protein
contig_35	fig|6666666.28487.peg.3105	Protein	120516	122120	+	kumamolisin
contig_35	fig|6666666.28487.peg.3106	Protein	122152	124074	+	hypothetical protein
contig_35	fig|6666666.28487.peg.3107	Protein	124947	124075	-	Putative diguanylate cyclase (GGDEF domain) with PAS/PAC sensor and Response Regulator Receiver modulation
contig_35	fig|6666666.28487.peg.3108	Protein	125344	126108	+	hypothetical protein
contig_35	fig|6666666.28487.peg.3109	Protein	126590	126165	-	Arsenate reductase (EC 1.20.4.1)
contig_35	fig|6666666.28487.peg.3110	Protein	127279	126599	-	Arsenate reductase (EC 1.20.4.1)
contig_35	fig|6666666.28487.peg.3111	Protein	128389	127280	-	Arsenical-resistance protein ACR3

contig_35	fig|6666666.28487.peg.3112	Protein	128661	128386	-	Arsenical resistance operon repressor
contig_35	fig|6666666.28487.peg.3113	Protein	129344	128892	-	Lactoylglutathione lyase (EC 4.4.1.5)
contig_35	fig|6666666.28487.peg.3114	Protein	129447	129806	+	transcriptional regulator, ArsR family
contig_35	fig|6666666.28487.peg.3115	Protein	130258	130431	+	hypothetical protein
contig_35	fig|6666666.28487.peg.3116	Protein	131098	130454	-	Two component transcriptional regulatory protein DevR
contig_35	fig|6666666.28487.peg.3117	Protein	131287	131168	-	hypothetical protein
contig_35	fig|6666666.28487.peg.3118	Protein	131330	132685	+	Wax ester synthase/acyl-CoA:diacylglycerol acyltransferase
contig_35	fig|6666666.28487.peg.3119	Protein	137945	133554	-	Probable cation-transporting ATPase I (EC 3.6.3.-)
contig_35	fig|6666666.28487.peg.3120	Protein	138051	138926	+	Universal stress protein family
contig_35	fig|6666666.28487.peg.3121	Protein	139673	138930	-	Zinc transporter, ZIP family
contig_35	fig|6666666.28487.peg.3122	Protein	139801	140694	+	Universal stress protein family
contig_35	fig|6666666.28487.peg.3123	Protein	141399	140656	-	FIG00828423: hypothetical protein
contig_36	fig|6666666.28487.peg.3124	Protein	68	313	+	protein of unknown function DUF222
contig_36	fig|6666666.28487.peg.3125	Protein	1103	303	-	Protein tyrosine phosphatase (EC 3.1.3.48)
contig_36	fig|6666666.28487.peg.3126	Protein	2353	1103	-	Butyryl-CoA dehydrogenase (EC 1.3.99.2)
contig_36	fig|6666666.28487.peg.3127	Protein	2653	4098	+	FAD dependent oxidoreductase
contig_36	fig|6666666.28487.peg.3128	Protein	5080	4100	-	Ethanolamine operon regulatory protein
contig_36	fig|6666666.28487.peg.3129	Protein	5233	5874	+	DNA-binding response regulator, LuxR family protein
contig_36	fig|6666666.28487.peg.3130	Protein	5876	7072	+	FIG00830541: hypothetical protein

contig_36	fig|6666666.28487.peg.3131	Protein	8508	7069	-	NAD(P) transhydrogenase subunit beta (EC 1.6.1.2)
contig_36	fig|6666666.28487.peg.3132	Protein	10044	8512	-	NAD(P) transhydrogenase alpha subunit (EC 1.6.1.2)
contig_36	fig|6666666.28487.peg.3133	Protein	10235	11323	+	putative alcohol dehydrogenase
contig_36	fig|6666666.28487.peg.3134	Protein	11388	12263	+	ABC-type nitrate/sulfonate/bicarbonate transport system, permease component
contig_36	fig|6666666.28487.peg.3135	Protein	12271	13308	+	Taurine-binding periplasmic protein TauA
contig_36	fig|6666666.28487.peg.3136	Protein	13292	14071	+	ABC-type nitrate/sulfonate/bicarbonate transport system, ATPase component
contig_36	fig|6666666.28487.peg.3137	Protein	14272	15402	+	Flavodoxin reductases (ferredoxin-NADPH reductases) family 1
contig_36	fig|6666666.28487.peg.3138	Protein	15461	16621	+	POSSIBLE LINOLEOYL-CoA DESATURASE (DELTA(6)-DESATURASE)
contig_36	fig|6666666.28487.peg.3139	Protein	16717	17124	+	FIG00823577: hypothetical protein
contig_36	fig|6666666.28487.peg.3140	Protein	17234	17094	-	hypothetical protein
contig_36	fig|6666666.28487.peg.3141	Protein	17361	18032	+	Transcriptional regulator, TetR family
contig_36	fig|6666666.28487.peg.3142	Protein	18085	18525	+	FIG00820956: hypothetical protein
contig_36	fig|6666666.28487.peg.3143	Protein	19183	18533	-	Transcriptional regulator, GntR family
contig_36	fig|6666666.28487.peg.3144	Protein	19340	21019	+	Long-chain-fatty-acid--CoA ligase (EC 6.2.1.3)
contig_36	fig|6666666.28487.peg.3145	Protein	21245	22042	+	Conserved hypothetical integral membrane protein YrbE1A
contig_36	fig|6666666.28487.peg.3146	Protein	22050	22925	+	Conserved hypothetical integral membrane protein YrbE1B
contig_36	fig|6666666.28487.peg.3147	Protein	22931	24148	+	MCE-family protein Mce1A

contig_36	fig|6666666.28487.peg.3148	Protein	24145	25176	+	MCE-family protein Mce1B
contig_36	fig|6666666.28487.peg.3149	Protein	25173	26741	+	MCE-family protein Mce1C
contig_36	fig|6666666.28487.peg.3150	Protein	26755	28398	+	MCE-family protein Mce1D
contig_36	fig|6666666.28487.peg.3151	Protein	28395	29540	+	MCE-family lipoprotein LprK (MCE-family lipoprotein Mce1e)
contig_36	fig|6666666.28487.peg.3152	Protein	29544	31106	+	MCE-family protein Mce1F
contig_36	fig|6666666.28487.peg.3153	Protein	31076	31696	+	FIG033430: Probable conserved MCE associated membrane protein
contig_36	fig|6666666.28487.peg.3154	Protein	31693	32673	+	FIG033285: Conserved MCE associated transmembrane protein
contig_36	fig|6666666.28487.peg.3155	Protein	32670	33236	+	FIG034772: Probable conserved MCE associated protein
contig_36	fig|6666666.28487.peg.3156	Protein	33203	34159	+	FIG030769: Probable conserved MCE associated membrane protein
contig_36	fig|6666666.28487.peg.3157	Protein	34170	34856	+	protein of unknown function DUF1275
contig_36	fig|6666666.28487.peg.3158	Protein	34853	35614	+	Ribulosamine/erythrulosamine 3-kinase potentially involved in protein deglycation
contig_36	fig|6666666.28487.peg.3159	Protein	36760	35729	-	Possible lipoprotein LprO
contig_36	fig|6666666.28487.peg.3160	Protein	37112	37555	+	hypothetical protein
contig_36	fig|6666666.28487.peg.3161	Protein	38834	37506	-	L-sorbosone dehydrogenase
contig_36	fig|6666666.28487.peg.3162	Protein	39579	38812	-	Pirin
contig_36	fig|6666666.28487.peg.3163	Protein	39680	40126	+	Transcriptional regulator, MarR family
contig_36	fig|6666666.28487.peg.3164	Protein	41026	40130	-	RNA polymerase factor sigma-70

contig_36	fig|6666666.28487.peg.3165	Protein	41036	41161	+	hypothetical protein
contig_36	fig|6666666.28487.peg.3166	Protein	41219	42118	+	Monoglyceride lipase (EC 3.1.1.23)
contig_36	fig|6666666.28487.peg.3167	Protein	42115	43113	+	hydrolase, alpha-beta fold family, putative
contig_36	fig|6666666.28487.peg.3168	Protein	43202	43954	+	FIG00819976: hypothetical protein
contig_36	fig|6666666.28487.peg.3169	Protein	43951	44442	+	FIG00820103: hypothetical protein
contig_36	fig|6666666.28487.peg.3170	Protein	44457	44678	+	hypothetical protein
contig_36	fig|6666666.28487.peg.3171	Protein	44736	45401	+	O-methyltransferase
contig_36	fig|6666666.28487.peg.3172	Protein	45469	46047	+	Transcriptional regulator, TetR family
contig_36	fig|6666666.28487.peg.3173	Protein	46133	46984	+	Metallo-beta-lactamase superfamily protein PA0057
contig_36	fig|6666666.28487.peg.3174	Protein	47666	46977	-	Transcriptional regulator, IclR family
contig_36	fig|6666666.28487.peg.3175	Protein	47734	49068	+	oxidoreductase, FAD-binding
contig_36	fig|6666666.28487.peg.3176	Protein	49068	49859	+	FIG00820194: hypothetical protein
contig_36	fig|6666666.28487.peg.3177	Protein	49856	50260	+	FIG00823361: hypothetical protein
contig_36	fig|6666666.28487.peg.3178	Protein	50273	50413	+	hypothetical protein
contig_36	fig|6666666.28487.peg.3179	Protein	50478	50918	+	FIG00826419: hypothetical protein
contig_36	fig|6666666.28487.peg.3180	Protein	51804	50905	-	hypothetical protein
contig_36	fig|6666666.28487.peg.3181	Protein	51807	51920	+	hypothetical protein
contig_36	fig|6666666.28487.peg.3182	Protein	52106	53056	+	Agmatinase (EC 3.5.3.11)
contig_36	fig|6666666.28487.peg.3183	Protein	53053	54675	+	Acetohydroxy acid synthase

contig_36	fig|6666666.28487.peg.3184	Protein	54691	56154	+	sodium-solute symporter, putative
contig_36	fig|6666666.28487.peg.3185	Protein	56535	56170	-	hypothetical protein
contig_36	fig|6666666.28487.peg.3186	Protein	56620	57225	+	Transcriptional regulator, TetR family
contig_36	fig|6666666.28487.peg.3187	Protein	57917	57222	-	hypothetical protein
contig_36	fig|6666666.28487.peg.3188	Protein	59620	58079	-	PE-PGRS FAMILY PROTEIN
contig_36	fig|6666666.28487.peg.3189	Protein	61094	59973	-	ABC transporter ATP-binding protein
contig_36	fig|6666666.28487.peg.3190	Protein	61615	61094	-	ABC transporter ATP-binding protein
contig_36	fig|6666666.28487.peg.3191	Protein	62065	61637	-	hypothetical protein
contig_36	fig|6666666.28487.peg.3192	Protein	62757	62146	-	RhtB family transporter
contig_36	fig|6666666.28487.peg.3193	Protein	62803	63312	+	hypothetical protein
contig_36	fig|6666666.28487.peg.3194	Protein	63510	64730	+	Transcriptional regulator, IclR family
contig_36	fig|6666666.28487.peg.3195	Protein	66457	64727	-	Dihydroxy-acid dehydratase (EC 4.2.1.9)
contig_36	fig|6666666.28487.peg.3196	Protein	66504	67133	+	putative lipoprotein
contig_37	fig|6666666.28487.peg.3197	Protein	462	1226	+	Lactate-responsive regulator LldR in Actinobacteria, GntR family
contig_37	fig|6666666.28487.peg.3198	Protein	3081	1312	-	CONSERVED MEMBRANE PROTEIN
contig_37	fig|6666666.28487.peg.3199	Protein	3664	3143	-	Membrane protein 2, distant similarity to thiosulphate:quinone oxidoreductase DoxD
contig_37	fig|6666666.28487.peg.3200	Protein	5159	3708	-	carotenoid oxygenase
contig_37	fig|6666666.28487.peg.3201	Protein	5211	5894	+	Transcriptional regulator, TetR family

contig_37	fig|6666666.28487.peg.3202	Protein	7150	5891	-	FIG028963: hypothetical protein
contig_37	fig|6666666.28487.peg.3203	Protein	7864	7193	-	MCE associated membrane protein
contig_37	fig|6666666.28487.peg.3204	Protein	8559	7861	-	FIG00821219: hypothetical protein
contig_37	fig|6666666.28487.peg.3205	Protein	9983	8523	-	MCE-family protein Mce1F
contig_37	fig|6666666.28487.peg.3206	Protein	11138	9987	-	MCE-family lipoprotein LprK (MCE-family lipoprotein Mce1e)
contig_37	fig|6666666.28487.peg.3207	Protein	12517	11150	-	MCE-family protein Mce1D
contig_37	fig|6666666.28487.peg.3208	Protein	13884	12514	-	MCE-family protein Mce1C
contig_37	fig|6666666.28487.peg.3209	Protein	14912	13881	-	MCE-family protein Mce1B
contig_37	fig|6666666.28487.peg.3210	Protein	16414	14948	-	MCE-family protein Mce1A
contig_37	fig|6666666.28487.peg.3211	Protein	17272	16418	-	Conserved hypothetical integral membrane protein YrbE1B
contig_37	fig|6666666.28487.peg.3212	Protein	18036	17269	-	Conserved hypothetical integral membrane protein YrbE1A
contig_38	fig|6666666.28487.rna.19	RNA	244	3364	+	Large Subunit Ribosomal RNA; lsuRNA; LSU rRNA
contig_38	fig|6666666.28487.rna.20	RNA	3480	3594	+	5S RNA
contig_39	fig|6666666.28487.peg.3213	Protein	139	1212	+	RD1 region associated protein Rv3876
contig_39	fig|6666666.28487.peg.3214	Protein	1405	1241	-	FIG00822430: hypothetical protein
contig_39	fig|6666666.28487.peg.3215	Protein	1455	1811	+	hypothetical protein
contig_39	fig|6666666.28487.peg.3216	Protein	1949	2290	+	protein of unknown function DUF1486
contig_39	fig|6666666.28487.peg.3217	Protein	3015	2299	-	hypothetical protein
contig_39	fig|6666666.28487.peg.3218	Protein	3923	3012	-	FIG00820405: hypothetical protein

contig_39	fig|6666666.28487.peg.3219	Protein	4879	3920	-	Von Willebrand factor type A domain protein
contig_39	fig|6666666.28487.peg.3220	Protein	5343	4870	-	FIG00821533: hypothetical protein
contig_39	fig|6666666.28487.peg.3221	Protein	6193	5333	-	Cell division protein DivIC (FtsB), stabilizes FtsL against RasP cleavage
contig_39	fig|6666666.28487.peg.3222	Protein	7193	6207	-	MoxR-like ATPases
contig_39	fig|6666666.28487.peg.3223	Protein	8163	7294	-	1,4-dihydroxy-2-naphthoate octaprenyltransferase (EC 2.5.1.74)
contig_39	fig|6666666.28487.peg.3224	Protein	8206	8412	+	FIG00827146: hypothetical protein
contig_39	fig|6666666.28487.peg.3225	Protein	11372	8409	-	hypothetical protein
contig_39	fig|6666666.28487.peg.3226	Protein	11501	12277	+	5'-methylthioadenosine phosphorylase (EC 2.4.2.28)
contig_39	fig|6666666.28487.peg.3227	Protein	12283	13320	+	UDP-glucose 4-epimerase (EC 5.1.3.2)
contig_39	fig|6666666.28487.peg.3228	Protein	13346	14476	+	Enoyl-[acyl-carrier-protein] reductase [FMN] (EC 1.3.1.9)
contig_39	fig|6666666.28487.peg.3229	Protein	15574	14549	-	putative two-component system sensor kinase
contig_39	fig|6666666.28487.peg.3230	Protein	16268	15561	-	two component transcriptional regulator, winged helix family
contig_39	fig|6666666.28487.peg.3231	Protein	16370	17020	+	Glycosyltransferase (EC 2.4.1.-)
contig_39	fig|6666666.28487.peg.3232	Protein	17017	17691	+	hypothetical protein
contig_39	fig|6666666.28487.peg.3233	Protein	17691	18317	+	FIG00823617: hypothetical protein
contig_39	fig|6666666.28487.peg.3234	Protein	19771	18392	-	Putative ESX-1 secretion system component Rv3877
contig_39	fig|6666666.28487.peg.3235	Protein	20465	19863	-	Phosphohydrolase
contig_39	fig|6666666.28487.peg.3236	Protein	21602	20520	-	O-succinylbenzoic acid--CoA ligase (EC 6.2.1.26)

contig_39	fig|6666666.28487.peg.3237	Protein	21979	21659	-	FIG00995255: hypothetical protein
contig_39	fig|6666666.28487.peg.3238	Protein	22202	23443	+	Probable low-affinity inorganic phosphate transporter
contig_39	fig|6666666.28487.peg.3239	Protein	23459	23743	+	hypothetical protein
contig_39	fig|6666666.28487.peg.3240	Protein	23755	24066	+	FIG00995255: hypothetical protein
contig_39	fig|6666666.28487.peg.3241	Protein	24513	24127	-	FIG020377: hypothetical protein
contig_39	fig|6666666.28487.peg.3242	Protein	26072	24537	-	Long-chain-fatty-acid--CoA ligase (EC 6.2.1.3)
contig_39	fig|6666666.28487.peg.3243	Protein	26963	26079	-	"Oxidoreductase, short-chain dehydrogenase/reductase family"
contig_39	fig|6666666.28487.peg.3244	Protein	27961	27050	-	Naphthoate synthase (EC 4.1.3.36)
contig_39	fig|6666666.28487.peg.3245	Protein	28408	27965	-	FIG00819980: hypothetical protein
contig_39	fig|6666666.28487.peg.3246	Protein	29105	28419	-	hydrolase, haloacid dehalogenase-like family, putative
contig_39	fig|6666666.28487.peg.3247	Protein	29157	29882	+	Glycosyl transferase, group 2 family protein
contig_39	fig|6666666.28487.peg.3248	Protein	29879	30820	+	Acyl-CoA dehydrogenase/oxidase domain protein
contig_39	fig|6666666.28487.peg.3249	Protein	30817	31584	+	LmbE-like protein
contig_39	fig|6666666.28487.peg.3250	Protein	31617	32204	+	Methyltransferase type 12
contig_39	fig|6666666.28487.peg.3251	Protein	33503	32295	-	RNA polymerase sigma-70 factor, ECF subfamily
contig_39	fig|6666666.28487.peg.3252	Protein	34788	33571	-	cyanate MFS transporter
contig_39	fig|6666666.28487.peg.3253	Protein	34836	35528	+	Transcriptional regulator, GntR family
contig_39	fig|6666666.28487.peg.3254	Protein	37115	35517	-	O-succinylbenzoic acid--CoA ligase (EC 6.2.1.26)
contig_39	fig|6666666.28487.peg.3255	Protein	37640	37224	-	PhnB protein

contig_39	fig|6666666.28487.peg.3256	Protein	38023	39276	+	Transcriptional regulator, TetR family
contig_39	fig|6666666.28487.peg.3257	Protein	39631	40482	+	Conserved hypothetical integral membrane protein YrbE1A
contig_39	fig|6666666.28487.peg.3258	Protein	40485	41342	+	Conserved hypothetical integral membrane protein YrbE1B
contig_39	fig|6666666.28487.peg.3259	Protein	41342	42913	+	MCE-family protein Mce1A
contig_39	fig|6666666.28487.peg.3260	Protein	42910	43965	+	MCE-family protein Mce1B
contig_39	fig|6666666.28487.peg.3261	Protein	43958	44995	+	MCE-family protein Mce1C
contig_39	fig|6666666.28487.peg.3262	Protein	44992	46110	+	MCE-family protein Mce1D
contig_39	fig|6666666.28487.peg.3263	Protein	46107	47324	+	MCE-family lipoprotein LprK (MCE-family lipoprotein Mce1e)
contig_39	fig|6666666.28487.peg.3264	Protein	47321	48571	+	MCE-family protein Mce1F
contig_39	fig|6666666.28487.peg.3265	Protein	48590	49192	+	FIG00822942: hypothetical protein
contig_39	fig|6666666.28487.peg.3266	Protein	49198	49986	+	FIG00820444: hypothetical protein
contig_39	fig|6666666.28487.peg.3267	Protein	49989	50573	+	FIG00821281: hypothetical protein
contig_39	fig|6666666.28487.peg.3268	Protein	50652	51611	+	FIG00824696: hypothetical protein
contig_39	fig|6666666.28487.peg.3269	Protein	51619	52206	+	Transcriptional regulator, TetR family
contig_39	fig|6666666.28487.peg.3270	Protein	52338	53900	+	Long-chain-fatty-acid--CoA ligase (EC 6.2.1.3)
contig_39	fig|6666666.28487.peg.3271	Protein	54517	53903	-	SOUL heme-binding protein
contig_39	fig|6666666.28487.peg.3272	Protein	54738	55052	+	Quaternary ammonium compound-resistance protein sugE
contig_39	fig|6666666.28487.peg.3273	Protein	55055	55399	+	Quaternary ammonium compound-resistance protein sugE
contig_39	fig|6666666.28487.peg.3274	Protein	55414	56370	+	O-succinylbenzoate synthase (EC 4.2.1.113)

contig_39	fig|6666666.28487.peg.3275	Protein	56424	57386	+	Transcriptional regulator, AraC family
contig_39	fig|6666666.28487.peg.3276	Protein	57404	58144	+	ThiJ/PfpI family protein
contig_39	fig|6666666.28487.peg.3277	Protein	58991	58131	-	hypothetical protein
contig_39	fig|6666666.28487.peg.3278	Protein	59107	59889	+	2-succinyl-6-hydroxy-2,4-cyclohexadiene-1-carboxylate synthase (EC 4.2.99.20)
contig_39	fig|6666666.28487.peg.3279	Protein	59919	61529	+	2-succinyl-5-enolpyruvyl-6-hydroxy-3-cyclohexene-1-carboxylic-acid synthase (EC 2.2.1.9)
contig_39	fig|6666666.28487.peg.3280	Protein	61526	62065	+	Possible membrane protein
contig_39	fig|6666666.28487.peg.3281	Protein	62729	62043	-	Serine/threonine-protein kinase pknE (EC 2.7.11.1)
contig_39	fig|6666666.28487.peg.3282	Protein	62854	62708	-	hypothetical protein
contig_39	fig|6666666.28487.peg.3283	Protein	62864	63991	+	Glycosyltransferase
contig_39	fig|6666666.28487.peg.3284	Protein	64166	64585	+	hypothetical protein
contig_39	fig|6666666.28487.peg.3285	Protein	64582	64992	+	hypothetical protein
contig_39	fig|6666666.28487.peg.3286	Protein	65161	64997	-	hypothetical protein
contig_39	fig|6666666.28487.peg.3287	Protein	66009	65185	-	oxidoreductase, short-chain dehydrogenase-reductase family
contig_39	fig|6666666.28487.peg.3288	Protein	66112	66798	+	ubiquinone/menaquinone biosynthesis methyltransferase
contig_39	fig|6666666.28487.peg.3289	Protein	66943	68001	+	hypothetical protein
contig_39	fig|6666666.28487.peg.3290	Protein	69559	67994	-	Beta-carotene ketolase (EC 1.14.-.-)
contig_39	fig|6666666.28487.peg.3291	Protein	69638	70237	+	Transcriptional regulator, TetR family
contig_39	fig|6666666.28487.peg.3292	Protein	70746	70327	-	FIG00823862: hypothetical protein

contig_39	fig|6666666.28487.peg.3293	Protein	72094	70844	-	Possible oxidoreductase (EC 1.-.-.-)
contig_39	fig|6666666.28487.peg.3294	Protein	72134	73123	+	Trans-hexaprenyltranstransferase(EC:2.5.1.30)
contig_39	fig|6666666.28487.peg.3295	Protein	73202	74101	+	Probable protease htpX homolog (EC 3.4.24.-)
contig_39	fig|6666666.28487.peg.3296	Protein	74395	74856	+	hypothetical protein
contig_39	fig|6666666.28487.peg.3297	Protein	74890	75426	+	FIG00825056: hypothetical protein
contig_39	fig|6666666.28487.peg.3298	Protein	76455	75430	-	Glycerol-3-phosphate dehydrogenase [NAD(P)+] (EC 1.1.1.94)
contig_39	fig|6666666.28487.peg.3299	Protein	77042	76551	-	UPF0234 protein YajQ
contig_39	fig|6666666.28487.rna.21	RNA	77186	77268	+	tRNA-Tyr-GTA
contig_39	fig|6666666.28487.peg.3300	Protein	77552	78328	+	Acyl-CoA thioesterase II (EC 3.1.2.-)
contig_39	fig|6666666.28487.peg.3301	Protein	79162	78398	-	hypothetical protein
contig_39	fig|6666666.28487.peg.3302	Protein	79316	79741	+	AclJ
contig_39	fig|6666666.28487.peg.3303	Protein	81518	79749	-	FIG00310351: hypothetical protein
contig_39	fig|6666666.28487.peg.3304	Protein	81930	82088	+	hypothetical protein
contig_39	fig|6666666.28487.peg.3305	Protein	82170	83354	+	hypothetical protein
contig_39	fig|6666666.28487.peg.3306	Protein	84389	83424	-	D-3-phosphoglycerate dehydrogenase (EC 1.1.1.95)
contig_39	fig|6666666.28487.peg.3307	Protein	85740	84391	-	Glucarate dehydratase (EC 4.2.1.40)
contig_39	fig|6666666.28487.peg.3308	Protein	86393	85758	-	Transcriptional regulator, IclR family
contig_4	fig|6666666.28487.peg.3309	Protein	152	802	+	Partial REP13E12 repeat protein
contig_4	fig|6666666.28487.peg.3310	Protein	1205	816	-	FIG00823474: hypothetical protein

contig_4	fig|6666666.28487.peg.3311	Protein	2412	1234	-	FIG00823518: hypothetical protein
contig_4	fig|6666666.28487.peg.3312	Protein	2983	2453	-	Transcriptional regulator, TetR family
contig_4	fig|6666666.28487.peg.3313	Protein	3224	3024	-	hypothetical protein
contig_4	fig|6666666.28487.peg.3314	Protein	3354	4385	+	SECRETED ANTIGEN 85-B FBPB (85B) (ANTIGEN 85 COMPLEX B) (MYCOLYL TRANSFERASE 85B) (FIBRONECTIN-BINDING PROTEIN B) (EXTRACELLULAR ALPHA-ANTIGEN)
contig_4	fig|6666666.28487.peg.3315	Protein	4457	5464	+	Alcohol dehydrogenase (EC 1.1.1.1)
contig_4	fig|6666666.28487.peg.3316	Protein	6683	5472	-	Probable acyl-CoA dehydrogenase (EC 1.3.99.3)
contig_4	fig|6666666.28487.peg.3317	Protein	8112	6715	-	Butyryl-CoA dehydrogenase (EC 1.3.99.2)
contig_4	fig|6666666.28487.peg.3318	Protein	8416	8216	-	hypothetical protein
contig_4	fig|6666666.28487.peg.3319	Protein	8426	9583	+	Esterase LipC
contig_4	fig|6666666.28487.peg.3320	Protein	10431	9655	-	Histidinol-phosphatase [alternative form] (EC 3.1.3.15)
contig_4	fig|6666666.28487.peg.3321	Protein	10434	10817	+	FIG00820561: hypothetical protein
contig_4	fig|6666666.28487.peg.3322	Protein	12175	10823	-	Ferredoxin--NADP(+) reductase, actinobacterial (eukaryote-like) type (EC 1.18.1.2)
contig_4	fig|6666666.28487.peg.3323	Protein	12385	13497	+	Peptide chain release factor 2
contig_4	fig|6666666.28487.peg.3324	Protein	13501	14472	+	Potassium efflux system KefA protein / Small-conductance mechanosensitive channel
contig_4	fig|6666666.28487.peg.3325	Protein	14469	14984	+	VgrG protein
contig_4	fig|6666666.28487.peg.3326	Protein	15026	15718	+	Cell division transporter, ATP-binding protein FtsE (TC 3.A.5.1.1)

contig_4	fig|6666666.28487.peg.3327	Protein	15719	16615	+	Cell division protein FtsX
contig_4	fig|6666666.28487.peg.3328	Protein	16618	17115	+	tmRNA-binding protein SmpB
contig_4	fig|6666666.28487.peg.3329	Protein	17208	17957	+	FIG00827717: hypothetical protein
contig_4	fig|6666666.28487.peg.3330	Protein	17986	20325	+	FIG00828555: hypothetical protein
contig_4	fig|6666666.28487.peg.3331	Protein	20365	20541	+	hypothetical protein
contig_4	fig|6666666.28487.peg.3332	Protein	21678	20857	-	Integrase
contig_40	fig|6666666.28487.peg.3333	Protein	612	106	-	hypothetical protein
contig_40	fig|6666666.28487.peg.3334	Protein	1593	679	-	phage integrase family protein
contig_40	fig|6666666.28487.peg.3335	Protein	1736	2299	+	Translation elongation factor P
contig_40	fig|6666666.28487.peg.3336	Protein	2362	2853	+	Transcription termination protein NusB
contig_40	fig|6666666.28487.peg.3337	Protein	2854	3399	+	hypothetical protein
contig_40	fig|6666666.28487.peg.3338	Protein	3415	4290	+	Citrate lyase beta chain (EC 4.1.3.6)
contig_40	fig|6666666.28487.peg.3339	Protein	5550	4303	-	CONSERVED 13E12 REPEAT FAMILY PROTEIN
contig_40	fig|6666666.28487.peg.3340	Protein	6806	5601	-	Beta-lactamase class C and other penicillin binding proteins
contig_40	fig|6666666.28487.peg.3341	Protein	6919	7500	+	Uracil phosphoribosyltransferase (EC 2.4.2.9) / Pyrimidine operon regulatory protein PyrR
contig_40	fig|6666666.28487.peg.3342	Protein	7529	8476	+	Aspartate carbamoyltransferase (EC 2.1.3.2)
contig_40	fig|6666666.28487.peg.3343	Protein	8473	9762	+	Dihydroorotase (EC 3.5.2.3)
contig_40	fig|6666666.28487.peg.3344	Protein	9759	10262	+	FIG00821137: secreted protein
contig_40	fig|6666666.28487.peg.3345	Protein	10259	11386	+	Carbamoyl-phosphate synthase small chain (EC 6.3.5.5)

contig_40	fig|6666666.28487.peg.3346	Protein	11488	14826	+	Carbamoyl-phosphate synthase large chain (EC 6.3.5.5)
contig_40	fig|6666666.28487.peg.3347	Protein	14829	15641	+	Orotidine 5'-phosphate decarboxylase (EC 4.1.1.23)
contig_40	fig|6666666.28487.peg.3348	Protein	16034	16351	+	integration host factor
contig_40	fig|6666666.28487.peg.3349	Protein	16362	16973	+	Guanylate kinase (EC 2.7.4.8)
contig_40	fig|6666666.28487.peg.3350	Protein	17004	17312	+	DNA-directed RNA polymerase omega subunit (EC 2.7.7.6)
contig_40	fig|6666666.28487.peg.3351	Protein	17323	18576	+	Phosphopantothenoylcysteine decarboxylase (EC 4.1.1.36) / Phosphopantothenoylcysteine synthetase (EC 6.3.2.5)
contig_40	fig|6666666.28487.peg.3352	Protein	18748	19956	+	S-adenosylmethionine synthetase (EC 2.5.1.6)
contig_40	fig|6666666.28487.peg.3353	Protein	21511	20021	-	Cyclohexanone monooxygenase (EC 1.14.13.22)
contig_40	fig|6666666.28487.peg.3354	Protein	22477	21578	-	Esterase/lipase
contig_40	fig|6666666.28487.peg.3355	Protein	22551	23447	+	Sugar phosphate isomerases/epimerases
contig_40	fig|6666666.28487.peg.3356	Protein	23561	24472	+	Endonuclease/exonuclease/phosphatase
contig_40	fig|6666666.28487.peg.3357	Protein	24954	25757	+	glycosyltransferase
contig_40	fig|6666666.28487.peg.3358	Protein	25814	26716	+	Succinoglycan biosynthesis protein
contig_40	fig|6666666.28487.peg.3359	Protein	26716	27387	+	hypothetical protein
contig_40	fig|6666666.28487.peg.3360	Protein	27384	28208	+	probable glucan endo-1,3-beta-D-glucosidase(EC:3.2.1.39)
contig_40	fig|6666666.28487.peg.3361	Protein	28205	29485	+	Probable glycosyl transferase
contig_40	fig|6666666.28487.peg.3362	Protein	29629	31737	+	oligosaccharide repeat unit transporter
contig_40	fig|6666666.28487.peg.3363	Protein	32879	31734	-	hypothetical protein
contig_40	fig|6666666.28487.peg.3364	Protein	33033	33740	+	YhhN family membrane protein

contig_40	fig|6666666.28487.peg.3365	Protein	33737	35737	+	Helicase PriA essential for oriC/DnaA-independent DNA replication
contig_40	fig|6666666.28487.peg.3366	Protein	35743	37575	+	Putative membrane protein precursor
contig_40	fig|6666666.28487.peg.3367	Protein	37569	38105	+	lemA protein
contig_40	fig|6666666.28487.peg.3368	Protein	38125	39051	+	Methionyl-tRNA formyltransferase (EC 2.1.2.9)
contig_40	fig|6666666.28487.peg.3369	Protein	39048	40433	+	Ribosomal RNA small subunit methyltransferase B (EC 2.1.1.-)
contig_40	fig|6666666.28487.peg.3370	Protein	40443	41120	+	Ribulose-phosphate 3-epimerase (EC 5.1.3.1)
contig_40	fig|6666666.28487.peg.3371	Protein	41117	42118	+	Diaminohydroxyphosphoribosylaminopyrimidine deaminase (EC 3.5.4.26) / 5-amino-6-(5-phosphoribosylamino)uracil reductase (EC 1.1.1.193)
contig_40	fig|6666666.28487.peg.3372	Protein	42315	42467	+	hypothetical protein
contig_40	fig|6666666.28487.peg.3373	Protein	43969	42434	-	EmrB/QacA family drug resistance transporter
contig_40	fig|6666666.28487.peg.3374	Protein	44718	44014	-	Lipoprotein LprG
contig_40	fig|6666666.28487.peg.3375	Protein	46502	44823	-	Trehalose synthase (EC 5.4.99.16)
contig_40	fig|6666666.28487.peg.3376	Protein	47485	46499	-	F420-dependent glucose-6-phosphate dehydrogenase
contig_40	fig|6666666.28487.peg.3377	Protein	47522	48133	+	Riboflavin synthase eubacterial/eukaryotic (EC 2.5.1.9)
contig_40	fig|6666666.28487.peg.3378	Protein	48338	49609	+	"3,4-dihydroxy-2-butanone 4-phosphate synthase (EC 4.1.99.12) / GTP cyclohydrolase II (EC 3.5.4.25)"
contig_40	fig|6666666.28487.peg.3379	Protein	49606	50085	+	6,7-dimethyl-8-ribityllumazine synthase (EC 2.5.1.78)
contig_40	fig|6666666.28487.peg.3380	Protein	50082	50546	+	Cell division protein DivIC (FtsB), stabilizes FtsL against RasP cleavage

contig_40	fig|6666666.28487.peg.3381	Protein	52455	50527	-	Gamma-glutamyltranspeptidase (EC 2.3.2.2)
contig_40	fig|6666666.28487.peg.3382	Protein	52500	54548	+	Excinuclease ABC subunit C
contig_40	fig|6666666.28487.peg.3383	Protein	54560	55474	+	FIG000506: Predicted P-loop-containing kinase
contig_40	fig|6666666.28487.peg.3384	Protein	55471	56511	+	FIG002813: hypothetical protein
contig_40	fig|6666666.28487.peg.3385	Protein	56502	57485	+	protein of unknown function DUF199
contig_40	fig|6666666.28487.peg.3386	Protein	58126	57482	-	hypothetical protein
contig_40	fig|6666666.28487.peg.3387	Protein	59394	58123	-	hypothetical protein
contig_40	fig|6666666.28487.peg.3388	Protein	59426	60139	+	Glutamine ABC transporter, periplasmic glutamine-binding protein (TC 3.A.1.3.2)
contig_40	fig|6666666.28487.peg.3389	Protein	60233	61255	+	NAD-dependent glyceraldehyde-3-phosphate dehydrogenase (EC 1.2.1.12)
contig_40	fig|6666666.28487.peg.3390	Protein	61270	62484	+	Phosphoglycerate kinase (EC 2.7.2.3)
contig_40	fig|6666666.28487.peg.3391	Protein	62503	63288	+	Triosephosphate isomerase (EC 5.3.1.1)
contig_41	fig|6666666.28487.peg.3392	Protein	1964	3	-	protein of unknown function DUF1524 RloF
contig_41	fig|6666666.28487.peg.3393	Protein	4131	1927	-	putative DNA helicase
contig_41	fig|6666666.28487.peg.3394	Protein	10524	4144	-	Helicase, C-terminal:Type III restriction enzyme, res subunit:DEAD/DEAH box helicase, N-terminal
contig_41	fig|6666666.28487.peg.3395	Protein	11223	10528	-	hypothetical protein
contig_41	fig|6666666.28487.peg.3396	Protein	15822	11227	-	Type II restriction enzyme, methylase subunits
contig_41	fig|6666666.28487.peg.3397	Protein	19113	15826	-	putative ATP-dependent helicase
contig_41	fig|6666666.28487.peg.3398	Protein	24264	19309	-	helicase, C-terminal:DEAD/DEAH box helicase, N-terminal

contig_41	fig|6666666.28487.peg.3399	Protein	27602	24282	-	FIG00820501: hypothetical protein
contig_41	fig|6666666.28487.peg.3400	Protein	28964	28023	-	POSSIBLE MEMBRANE PROTEIN
contig_41	fig|6666666.28487.peg.3401	Protein	30070	28970	-	POSSIBLE MEMBRANE PROTEIN
contig_41	fig|6666666.28487.peg.3402	Protein	30354	30653	+	protein of unknown function DUF732
contig_41	fig|6666666.28487.peg.3403	Protein	31655	30657	-	Membrane protein, putative
contig_41	fig|6666666.28487.peg.3404	Protein	31725	32132	+	hypothetical protein
contig_41	fig|6666666.28487.peg.3405	Protein	33708	32266	-	Serine/threonine-protein kinase pknE (EC 2.7.11.1)
contig_41	fig|6666666.28487.peg.3406	Protein	35305	33920	-	Wax ester synthase/acyl-CoA:diacylglycerol acyltransferase
contig_41	fig|6666666.28487.peg.3407	Protein	36115	35453	-	Transcriptional regulator, TetR family
contig_41	fig|6666666.28487.peg.3408	Protein	36291	36716	+	membrane protein, MmpS family
contig_41	fig|6666666.28487.peg.3409	Protein	36713	39598	+	Putative membrane protein
contig_41	fig|6666666.28487.peg.3410	Protein	39595	39894	+	POSSIBLE CONSERVED SECRETED PROTEIN
contig_41	fig|6666666.28487.peg.3411	Protein	39891	40361	+	possible secreted protein
contig_41	fig|6666666.28487.peg.3412	Protein	40689	41822	+	hypothetical protein
contig_41	fig|6666666.28487.peg.3413	Protein	42523	41864	-	hypothetical protein
contig_41	fig|6666666.28487.peg.3414	Protein	43938	42634	-	hypothetical protein
contig_41	fig|6666666.28487.peg.3415	Protein	44461	46074	+	Long-chain-fatty-acid--CoA ligase (EC 6.2.1.3)
contig_41	fig|6666666.28487.peg.3416	Protein	47331	46126	-	POSSIBLE LINOLEOYL-CoA DESATURASE (DELTA(6)-DESATURASE)
contig_41	fig|6666666.28487.peg.3417	Protein	48275	47391	-	Glycosyltransferase, group I

contig_41	fig|6666666.28487.peg.3418	Protein	48307	48609	+	hypothetical protein
contig_41	fig|6666666.28487.peg.3419	Protein	49012	48809	-	Cold shock protein CspA
contig_41	fig|6666666.28487.peg.3420	Protein	49629	49135	-	hypothetical protein
contig_41	fig|6666666.28487.peg.3421	Protein	50687	49926	-	hypothetical protein
contig_41	fig|6666666.28487.peg.3422	Protein	53198	50805	-	FIG00829332: hypothetical protein
contig_41	fig|6666666.28487.peg.3423	Protein	53665	53462	-	Cold shock protein CspA
contig_41	fig|6666666.28487.peg.3424	Protein	53946	53806	-	hypothetical protein
contig_41	fig|6666666.28487.peg.3425	Protein	55404	54061	-	putative cytochrome P450 hydroxylase
contig_41	fig|6666666.28487.peg.3426	Protein	55483	57684	+	putative membrane transport protein
contig_41	fig|6666666.28487.peg.3427	Protein	57777	58148	+	Serine phosphatase RsbU, regulator of sigma subunit
contig_41	fig|6666666.28487.peg.3428	Protein	59922	58159	-	Serine/threonine-protein kinase pknD (EC 2.7.11.1)
contig_41	fig|6666666.28487.peg.3429	Protein	60440	60225	-	hypothetical protein
contig_41	fig|6666666.28487.peg.3430	Protein	60553	61239	+	Transcriptional regulator, HxlR family
contig_41	fig|6666666.28487.peg.3431	Protein	61418	63007	+	Ketoglutarate semialdehyde dehydrogenase (EC 1.2.1.26)
contig_41	fig|6666666.28487.peg.3432	Protein	63038	63967	+	5-dehydro-4-deoxyglucarate dehydratase (EC 4.2.1.41)
contig_41	fig|6666666.28487.peg.3433	Protein	63972	65231	+	mandelate racemase family protein Pfl_3283
contig_41	fig|6666666.28487.peg.3434	Protein	65272	65742	+	hypothetical protein
contig_41	fig|6666666.28487.peg.3435	Protein	66400	65735	-	Transcriptional regulator, TetR family
contig_41	fig|6666666.28487.peg.3436	Protein	66518	67195	+	hypothetical protein

contig_41	fig|6666666.28487.peg.3437	Protein	67192	68001	+	short chain dehydrogenase
contig_41	fig|6666666.28487.peg.3438	Protein	67998	69407	+	Cytochrome P450 136
contig_41	fig|6666666.28487.peg.3439	Protein	69497	70420	+	Aromatic hydrocarbon utilization transcriptional regulator CatR (LysR family)
contig_41	fig|6666666.28487.peg.3440	Protein	72019	70502	-	secreted alkaline phosphatase
contig_41	fig|6666666.28487.peg.3441	Protein	72389	72165	-	hypothetical protein
contig_41	fig|6666666.28487.peg.3442	Protein	75083	72432	-	hypothetical protein
contig_41	fig|6666666.28487.peg.3443	Protein	75495	76055	+	Transcriptional regulator, TetR family
contig_41	fig|6666666.28487.peg.3444	Protein	76231	76641	+	hypothetical protein
contig_41	fig|6666666.28487.peg.3445	Protein	76651	77292	+	RNA polymerase sigma-70 factor, family protein
contig_41	fig|6666666.28487.peg.3446	Protein	77323	78267	+	Ava_C0101 and related proteins
contig_41	fig|6666666.28487.peg.3447	Protein	78240	79880	+	Glutamate synthase [NADPH] large chain (EC 1.4.1.13)
contig_41	fig|6666666.28487.peg.3448	Protein	79877	81829	+	Pyruvate oxidase [ubiquinone, cytochrome] (EC 1.2.2.2); putative
contig_41	fig|6666666.28487.peg.3449	Protein	81862	82866	+	Bifunctional protein: zinc-containing alcohol dehydrogenase; quinone oxidoreductase (NADPH:quinone reductase) (EC 1.1.1.-); Similar to arginate lyase
contig_41	fig|6666666.28487.peg.3450	Protein	83792	82863	-	D-3-phosphoglycerate dehydrogenase (EC 1.1.1.95)
contig_41	fig|6666666.28487.peg.3451	Protein	84114	85673	+	ABC transporter, substrate binding protein
contig_41	fig|6666666.28487.peg.3452	Protein	85701	86630	+	Probable ABC transporter permease
contig_41	fig|6666666.28487.peg.3453	Protein	86642	87631	+	Dipeptide transport system permease protein DppC (TC

						3.A.1.5.2)
contig_41	fig|6666666.28487.peg.3454	Protein	87628	88689	+	Oligopeptide transport ATP-binding protein OppD (TC 3.A.1.5.1)
contig_41	fig|6666666.28487.peg.3455	Protein	88682	89698	+	Oligopeptide transport ATP-binding protein OppF (TC 3.A.1.5.1)
contig_42	fig|6666666.28487.peg.3456	Protein	628	11	-	FIG00820710: hypothetical protein
contig_42	fig|6666666.28487.peg.3457	Protein	1358	861	-	FIG00823852: hypothetical protein
contig_42	fig|6666666.28487.peg.3458	Protein	2196	1450	-	Transcriptional regulator, MerR family
contig_42	fig|6666666.28487.peg.3459	Protein	2730	2260	-	FIG00672531: hypothetical protein
contig_42	fig|6666666.28487.peg.3460	Protein	3284	2889	-	Glycine cleavage system H protein
contig_42	fig|6666666.28487.peg.3461	Protein	4073	3306	-	Division initiation protein
contig_42	fig|6666666.28487.peg.3462	Protein	4402	4070	-	FIG025307: hypothetical protein
contig_42	fig|6666666.28487.peg.3463	Protein	5303	4416	-	FIG030330: hypothetical protein
contig_42	fig|6666666.28487.peg.3464	Protein	5884	5300	-	CDP-diacylglycerol--glycerol-3-phosphate 3-phosphatidyltransferase (EC 2.7.8.5)
contig_42	fig|6666666.28487.peg.3465	Protein	8135	5895	-	Protein export cytoplasm protein SecA ATPase RNA helicase (TC 3.A.5.1.1)
contig_42	fig|6666666.28487.peg.3466	Protein	8152	8265	+	hypothetical protein
contig_42	fig|6666666.28487.peg.3467	Protein	8448	10346	+	Drugs-transport transmembrane ATP-binding protein ABC transporter
contig_42	fig|6666666.28487.peg.3468	Protein	10392	12305	+	Drugs-transport transmembrane ATP-binding protein ABC transporter

contig_42	fig|6666666.28487.peg.3469	Protein	12430	13143	+	hypothetical protein
contig_42	fig|6666666.28487.peg.3470	Protein	13143	14921	+	hypothetical protein
contig_42	fig|6666666.28487.peg.3471	Protein	15006	15173	+	hypothetical protein
contig_42	fig|6666666.28487.peg.3472	Protein	15199	15666	+	hypothetical protein
contig_42	fig|6666666.28487.peg.3473	Protein	16342	15638	-	Transcriptional regulator, TetR family
contig_42	fig|6666666.28487.peg.3474	Protein	16580	16413	-	hypothetical protein
contig_42	fig|6666666.28487.peg.3475	Protein	16557	17915	+	hypothetical protein
contig_42	fig|6666666.28487.peg.3476	Protein	19628	17886	-	hypothetical protein
contig_42	fig|6666666.28487.peg.3477	Protein	21169	19655	-	fumarate reductase/succinate dehydrogenase flavoprotein, N-terminal:FAD dependent oxidoreductase
contig_42	fig|6666666.28487.peg.3478	Protein	21690	21166	-	FIG00824419: hypothetical protein
contig_42	fig|6666666.28487.peg.3479	Protein	22431	21751	-	Transcriptional regulator, TetR family
contig_42	fig|6666666.28487.peg.3480	Protein	22536	22976	+	FIG00820982: hypothetical protein
contig_42	fig|6666666.28487.peg.3481	Protein	23650	22973	-	FIG00820470: hypothetical protein
contig_42	fig|6666666.28487.peg.3482	Protein	25222	23816	-	Oxidoreductase (EC 1.1.1.-)
contig_42	fig|6666666.28487.peg.3483	Protein	26150	25233	-	Membrane-bound C-5 sterol desaturase Erg3
contig_42	fig|6666666.28487.peg.3484	Protein	27753	26182	-	Para-nitrobenzyl esterase (EC 3.1.1.-)
contig_42	fig|6666666.28487.peg.3485	Protein	29019	27832	-	Membrane protein mosC
contig_42	fig|6666666.28487.peg.3486	Protein	29018	31231	+	4-alpha-glucanotransferase (amylomaltase) (EC 2.4.1.25)
contig_42	fig|6666666.28487.peg.3487	Protein	31337	31215	-	hypothetical protein

contig_42	fig|6666666.28487.peg.3488	Protein	31317	32438	+	L-talarate dehydratase @ Galactarate dehydratase (EC 4.2.1.42)
contig_42	fig|6666666.28487.peg.3489	Protein	32478	33161	+	Predicted D-glucarate or D-galactorate regulator, GntR family
contig_42	fig|6666666.28487.peg.3490	Protein	34697	33165	-	Tricarboxylate transport membrane protein TctA
contig_42	fig|6666666.28487.peg.3491	Protein	35248	34712	-	Tricarboxylate transport protein TctB
contig_42	fig|6666666.28487.peg.3492	Protein	36262	35252	-	Tricarboxylate transport protein TctC
contig_42	fig|6666666.28487.peg.3493	Protein	36532	37023	+	FIG00821888: hypothetical protein
contig_42	fig|6666666.28487.peg.3494	Protein	37116	38018	+	Metal-dependent hydrolase
contig_42	fig|6666666.28487.peg.3495	Protein	38069	39124	+	Vanillate O-demethylase oxidoreductase (EC 1.14.13.-)
contig_42	fig|6666666.28487.peg.3496	Protein	39156	40604	+	Serine/threonine protein kinase (EC 2.7.11.1)
contig_42	fig|6666666.28487.peg.3497	Protein	41510	41019	-	MutT-like protein
contig_42	fig|6666666.28487.peg.3498	Protein	41526	42305	+	hypothetical protein
contig_42	fig|6666666.28487.peg.3499	Protein	42398	43180	+	probable secreted protein
contig_42	fig|6666666.28487.peg.3500	Protein	43804	43202	-	FIG00825921: hypothetical protein
contig_42	fig|6666666.28487.peg.3501	Protein	44675	43866	-	FIG00831975: hypothetical protein
contig_42	fig|6666666.28487.peg.3502	Protein	44781	45215	+	FIG00829100: hypothetical protein
contig_42	fig|6666666.28487.peg.3503	Protein	46379	45195	-	putative cytochrome P450 hydroxylase
contig_42	fig|6666666.28487.peg.3504	Protein	46637	47065	+	membrane protein, MmpS family
contig_42	fig|6666666.28487.peg.3505	Protein	47062	49968	+	Putative membrane protein
contig_42	fig|6666666.28487.peg.3506	Protein	49965	50300	+	POSSIBLE CONSERVED SECRETED PROTEIN

contig_42	fig|6666666.28487.peg.3507	Protein	50381	50941	+	FIG00820691: hypothetical protein
contig_42	fig|6666666.28487.peg.3508	Protein	51409	50942	-	possible secreted protein
contig_42	fig|6666666.28487.peg.3509	Protein	51778	51503	-	FIG00825577: hypothetical protein
contig_42	fig|6666666.28487.peg.3510	Protein	53078	51885	-	Pigment protein
contig_42	fig|6666666.28487.peg.3511	Protein	54086	53097	-	hypothetical protein
contig_42	fig|6666666.28487.peg.3512	Protein	54445	55854	+	Ethanolamine permease
contig_42	fig|6666666.28487.peg.3513	Protein	55851	57260	+	Ethanolamine ammonia-lyase heavy chain (EC 4.3.1.7)
contig_42	fig|6666666.28487.peg.3514	Protein	57257	58009	+	Ethanolamine ammonia-lyase light chain (EC 4.3.1.7)
contig_42	fig|6666666.28487.peg.3515	Protein	58654	58010	-	FIG00833096: hypothetical protein
contig_42	fig|6666666.28487.peg.3516	Protein	61005	58672	-	Transcription accessory protein (S1 RNA-binding domain)
contig_42	fig|6666666.28487.peg.3517	Protein	61101	62888	+	Pyruvate oxidase (EC 1.2.3.3)
contig_42	fig|6666666.28487.peg.3518	Protein	62965	65112	+	Catalase (EC 1.11.1.6)
contig_42	fig|6666666.28487.peg.3519	Protein	65495	66334	+	putative transcriptional regulator
contig_42	fig|6666666.28487.peg.3520	Protein	66331	66480	+	hypothetical protein
contig_42	fig|6666666.28487.peg.3521	Protein	68743	66491	-	Catalase (EC 1.11.1.6) / Peroxidase (EC 1.11.1.7)
contig_42	fig|6666666.28487.peg.3522	Protein	69205	68756	-	Transcriptional regulator, FUR family
contig_42	fig|6666666.28487.peg.3523	Protein	70696	69398	-	putative membrane protein
contig_42	fig|6666666.28487.peg.3524	Protein	71894	70749	-	FIG00829802: hypothetical protein
contig_42	fig|6666666.28487.peg.3525	Protein	74296	72059	-	Isocitrate dehydrogenase [NADP] (EC 1.1.1.42); Monomeric isocitrate dehydrogenase [NADP] (EC 1.1.1.42)

contig_42	fig|6666666.28487.peg.3526	Protein	74535	75380	+	LpqJ
contig_42	fig|6666666.28487.peg.3527	Protein	75424	75762	+	Cupin 2, conserved barrel domain protein
contig_42	fig|6666666.28487.peg.3528	Protein	77163	75766	-	Aromatic amino acid transport protein AroP
contig_42	fig|6666666.28487.peg.3529	Protein	77291	78814	+	hypothetical protein
contig_42	fig|6666666.28487.peg.3530	Protein	78887	79543	+	POSSIBLE CONSERVED EXPORTED PROTEIN
contig_42	fig|6666666.28487.peg.3531	Protein	80555	79530	-	Alcohol dehydrogenase (EC 1.1.1.1)
contig_42	fig|6666666.28487.peg.3532	Protein	82518	80611	-	Alkaline phosphodiesterase I (EC 3.1.4.1) / Nucleotide pyrophosphatase (EC 3.6.1.9)
contig_42	fig|6666666.28487.peg.3533	Protein	83472	82705	-	Possible membrane protein
contig_44	fig|6666666.28487.peg.3534	Protein	54	974	+	Methyltransferase domain
contig_44	fig|6666666.28487.peg.3535	Protein	1028	2146	+	Methyltransferase (EC 2.1.1.-)
contig_44	fig|6666666.28487.peg.3536	Protein	2230	2958	+	immunogenic protein MPB64/MPT64 precursor
contig_44	fig|6666666.28487.peg.3537	Protein	4495	2948	-	Sodium/alanine symporter family protein
contig_44	fig|6666666.28487.peg.3538	Protein	4454	4579	+	hypothetical protein
contig_44	fig|6666666.28487.peg.3539	Protein	5933	4611	-	FIG00995884: hypothetical protein
contig_44	fig|6666666.28487.peg.3540	Protein	5982	6719	+	Galactoside O-acetyltransferase (EC 2.3.1.18)
contig_44	fig|6666666.28487.peg.3541	Protein	6885	8318	+	hypothetical protein
contig_44	fig|6666666.28487.peg.3542	Protein	9583	8351	-	Glycosyl transferase, group 1
contig_44	fig|6666666.28487.peg.3543	Protein	11148	9580	-	Glycogen branching enzyme, GH-57-type, archaeal (EC 2.4.1.18)

contig_44	fig|6666666.28487.peg.3544	Protein	11918	11145	-	Methyltransferase (EC 2.1.1.-)
contig_44	fig|6666666.28487.peg.3545	Protein	12127	12918	+	Electron transfer flavoprotein, beta subunit
contig_44	fig|6666666.28487.peg.3546	Protein	12958	13914	+	Electron transfer flavoprotein, alpha subunit
contig_44	fig|6666666.28487.peg.3547	Protein	14005	14964	+	Putative hemolysin
contig_44	fig|6666666.28487.peg.3548	Protein	14961	15818	+	1-acyl-sn-glycerol-3-phosphate acyltransferase (EC 2.3.1.51)
contig_44	fig|6666666.28487.peg.3549	Protein	15865	17070	+	Cysteine desulfurase (EC 2.8.1.7)
contig_44	fig|6666666.28487.peg.3550	Protein	17073	18146	+	tRNA-specific 2-thiouridylase MnmA
contig_44	fig|6666666.28487.peg.3551	Protein	18790	18143	-	Lipoprotein LpqA
contig_44	fig|6666666.28487.peg.3552	Protein	18826	19836	+	vitamin-B12 independent methionine synthase family protein
contig_44	fig|6666666.28487.peg.3553	Protein	21491	19875	-	4-coumarate--CoA ligase 1 (EC 6.2.1.12)
contig_44	fig|6666666.28487.peg.3554	Protein	21930	21529	-	Phosphoribosylglycinamide formyltransferase (EC 2.1.2.2)
contig_44	fig|6666666.28487.peg.3555	Protein	22074	24221	+	DNA ligase (EC 6.5.1.2)
contig_44	fig|6666666.28487.peg.3556	Protein	24821	24225	-	FIG01000629: hypothetical protein
contig_44	fig|6666666.28487.peg.3557	Protein	24997	25341	+	Aspartyl-tRNA(Asn) amidotransferase subunit C (EC 6.3.5.6) @ Glutamyl-tRNA(Gln) amidotransferase subunit C (EC 6.3.5.7)
contig_44	fig|6666666.28487.peg.3558	Protein	25338	26822	+	Aspartyl-tRNA(Asn) amidotransferase subunit A (EC 6.3.5.6) @ Glutamyl-tRNA(Gln) amidotransferase subunit A (EC 6.3.5.7)
contig_44	fig|6666666.28487.peg.3559	Protein	26841	27872	+	6-phosphofructokinase (EC 2.7.1.11)
contig_44	fig|6666666.28487.peg.3560	Protein	27996	29507	+	Aspartyl-tRNA(Asn) amidotransferase subunit B (EC 6.3.5.6) @ Glutamyl-tRNA(Gln) amidotransferase subunit B (EC

						6.3.5.7)
contig_44	fig|6666666.28487.peg.3561	Protein	29526	30890	+	Sensor-type histidine kinase prrB (EC 2.7.13.3)
contig_44	fig|6666666.28487.peg.3562	Protein	32122	31001	-	Glucose/sorbosone dehydrogenase, lipoprotein LppZ
contig_44	fig|6666666.28487.peg.3563	Protein	32346	33104	+	Membrane protein 2, distant similarity to thiosulphate:quinone oxidoreductase DoxD
contig_44	fig|6666666.28487.peg.3564	Protein	33506	33108	-	Low molecular weight protein antigen 6
contig_44	fig|6666666.28487.peg.3565	Protein	33753	35624	+	Acetolactate synthase large subunit (EC 2.2.1.6)
contig_44	fig|6666666.28487.peg.3566	Protein	35624	36124	+	Acetolactate synthase small subunit (EC 2.2.1.6)
contig_44	fig|6666666.28487.peg.3567	Protein	36196	37209	+	Ketol-acid reductoisomerase (EC 1.1.1.86)
contig_45	fig|6666666.28487.peg.3568	Protein	320	514	+	hypothetical protein
contig_45	fig|6666666.28487.peg.3569	Protein	3023	1326	-	hypothetical protein
contig_45	fig|6666666.28487.peg.3570	Protein	3085	3810	+	Adenylosuccinate synthetase (EC 6.3.4.4)
contig_45	fig|6666666.28487.peg.3571	Protein	3807	4424	+	FIG00822066: hypothetical protein
contig_45	fig|6666666.28487.peg.3572	Protein	4509	5171	+	FIG00825411: hypothetical protein
contig_45	fig|6666666.28487.peg.3573	Protein	5443	5252	-	putative ferredoxin
contig_45	fig|6666666.28487.peg.3574	Protein	6270	5440	-	putative cytochrome P450 hydroxylase
contig_45	fig|6666666.28487.peg.3575	Protein	8167	6224	-	Dihydroxy-acid dehydratase (EC 4.2.1.9)
contig_45	fig|6666666.28487.peg.3576	Protein	8166	8351	+	hypothetical protein
contig_45	fig|6666666.28487.peg.3577	Protein	10038	8323	-	FAD dependent oxidoreductase
contig_45	fig|6666666.28487.peg.3578	Protein	10360	10028	-	hypothetical protein

contig_45	fig|6666666.28487.peg.3579	Protein	11783	10347	-	4-aminobutyraldehyde dehydrogenase (EC 1.2.1.19)
contig_45	fig|6666666.28487.peg.3580	Protein	12305	11796	-	Transcriptional regulator, TetR family
contig_45	fig|6666666.28487.peg.3581	Protein	13994	12456	-	hypothetical protein
contig_45	fig|6666666.28487.peg.3582	Protein	15109	14105	-	4-hydroxy-2-oxovalerate aldolase (EC 4.1.3.-)
contig_45	fig|6666666.28487.peg.3583	Protein	16056	15106	-	Acetaldehyde dehydrogenase, acetylating, (EC 1.2.1.10) in gene cluster for degradation of phenols, cresols, catechol
contig_45	fig|6666666.28487.peg.3584	Protein	16526	16065	-	hypothetical protein
contig_45	fig|6666666.28487.peg.3585	Protein	16483	17271	+	hypothetical protein
contig_45	fig|6666666.28487.peg.3586	Protein	17358	18143	+	hypothetical protein
contig_45	fig|6666666.28487.peg.3587	Protein	18173	18445	+	hypothetical protein
contig_45	fig|6666666.28487.peg.3588	Protein	18951	18595	-	hypothetical protein
contig_45	fig|6666666.28487.peg.3589	Protein	19628	19155	-	hypothetical protein
contig_46	fig|6666666.28487.peg.3590	Protein	80	361	+	Allophanate hydrolase (EC 3.5.1.54)
contig_46	fig|6666666.28487.peg.3591	Protein	374	1174	+	Formamidopyrimidine-DNA glycosylase (EC 3.2.2.23)
contig_46	fig|6666666.28487.peg.3592	Protein	1224	2912	+	Na+/H+ antiporter
contig_46	fig|6666666.28487.peg.3593	Protein	3746	2916	-	FIG00823957: hypothetical protein
contig_46	fig|6666666.28487.peg.3594	Protein	3910	4539	+	hypothetical protein
contig_46	fig|6666666.28487.peg.3595	Protein	5300	4647	-	putative exported protein of unknown function
contig_46	fig|6666666.28487.rna.22	RNA	5590	5520	-	tRNA-Gly-TCC
contig_46	fig|6666666.28487.rna.23	RNA	5727	5800	+	tRNA-Pro-TGG

contig_46	fig|6666666.28487.peg.3596	Protein	5847	7289	+	Cell division trigger factor (EC 5.2.1.8)
contig_46	fig|6666666.28487.peg.3597	Protein	7404	8000	+	ATP-dependent Clp protease proteolytic subunit (EC 3.4.21.92)
contig_46	fig|6666666.28487.peg.3598	Protein	7997	8635	+	ATP-dependent Clp protease proteolytic subunit (EC 3.4.21.92)
contig_46	fig|6666666.28487.peg.3599	Protein	9082	8642	-	Pyridoxine 5'-phosphate oxidase, Rv1155
contig_46	fig|6666666.28487.peg.3600	Protein	9216	9485	+	Flagelliform silk protein (Fragment)
contig_46	fig|6666666.28487.peg.3601	Protein	9815	9675	-	hypothetical protein
contig_46	fig|6666666.28487.peg.3602	Protein	9861	11141	+	ATP-dependent Clp protease ATP-binding subunit ClpX
contig_46	fig|6666666.28487.peg.3603	Protein	11312	11148	-	hypothetical protein
contig_46	fig|6666666.28487.peg.3604	Protein	12655	11309	-	POSSIBLE GLYCOSYL TRANSFERASE
contig_46	fig|6666666.28487.peg.3605	Protein	13978	12668	-	POSSIBLE GLYCOSYL TRANSFERASE
contig_46	fig|6666666.28487.peg.3606	Protein	14920	14057	-	Formate dehydrogenase chain D (EC 1.2.1.2)
contig_46	fig|6666666.28487.peg.3607	Protein	17350	14987	-	Putative formate dehydrogenase oxidoreductase protein
contig_46	fig|6666666.28487.peg.3608	Protein	17354	17506	+	hypothetical protein
contig_46	fig|6666666.28487.peg.3609	Protein	17592	19769	+	Glutamine synthetase type III, GlnN (EC 6.3.1.2)
contig_46	fig|6666666.28487.peg.3610	Protein	20874	19873	-	Probable conserved lipoprotein LppL
contig_46	fig|6666666.28487.peg.3611	Protein	21970	21125	-	4-hydroxyphenylpyruvate dioxygenase (EC 1.13.11.27)
contig_46	fig|6666666.28487.peg.3612	Protein	23567	22074	-	Methylmalonate-semialdehyde dehydrogenase (EC 1.2.1.27)
contig_46	fig|6666666.28487.peg.3613	Protein	24607	23603	-	Myo-inositol 2-dehydrogenase (EC 1.1.1.18)

contig_46	fig|6666666.28487.peg.3614	Protein	25512	24610	-	Inosose dehydratase (EC 4.2.1.44)
contig_46	fig|6666666.28487.peg.3615	Protein	27477	25528	-	Epi-inositol hydrolase (EC 3.7.1.-)
contig_46	fig|6666666.28487.peg.3616	Protein	28357	27482	-	5-deoxy-glucuronate isomerase (EC 5.3.1.-)
contig_46	fig|6666666.28487.peg.3617	Protein	29238	28354	-	5-keto-2-deoxy-D-gluconate-6 phosphate aldolase [form 2] (EC 4.1.2.29)
contig_46	fig|6666666.28487.peg.3618	Protein	30202	29231	-	5-keto-2-deoxygluconokinase (EC 2.7.1.92)
contig_46	fig|6666666.28487.peg.3619	Protein	30369	31088	+	Transcriptional regulator, GntR family
contig_46	fig|6666666.28487.peg.3620	Protein	31949	31164	-	Hydroxypyruvate isomerase (EC 5.3.1.22)
contig_46	fig|6666666.28487.peg.3621	Protein	32143	33135	+	transcriptional regulator, LacI family
contig_46	fig|6666666.28487.peg.3622	Protein	33300	34286	+	Inositol transport system sugar-binding protein
contig_46	fig|6666666.28487.peg.3623	Protein	34286	35344	+	Inositol transport system permease protein
contig_46	fig|6666666.28487.peg.3624	Protein	35349	36173	+	Inositol transport system ATP-binding protein
contig_46	fig|6666666.28487.peg.3625	Protein	36274	36690	+	hypothetical protein
contig_46	fig|6666666.28487.peg.3626	Protein	36836	36985	+	hypothetical protein
contig_46	fig|6666666.28487.peg.3627	Protein	37979	37017	-	NAD binding oxidoreductase
contig_46	fig|6666666.28487.peg.3628	Protein	38851	37976	-	Inosose dehydratase (EC 4.2.1.44)
contig_46	fig|6666666.28487.peg.3629	Protein	39864	38848	-	Myo-inositol 2-dehydrogenase 1 (EC 1.1.1.18)
contig_46	fig|6666666.28487.peg.3630	Protein	41084	39861	-	Protein involved in biosynthesis of mitomycin antibiotics/polyketide fumonisin
contig_46	fig|6666666.28487.peg.3631	Protein	41207	42217	+	transcriptional regulator, LacI family, putative

contig_46	fig|6666666.28487.peg.3632	Protein	42546	44483	+	2-oxoglutarate oxidoreductase, alpha subunit (EC 1.2.7.3)
contig_46	fig|6666666.28487.peg.3633	Protein	44512	45594	+	2-oxoglutarate oxidoreductase, beta subunit (EC 1.2.7.3)
contig_46	fig|6666666.28487.peg.3634	Protein	45631	46251	+	Molybdopterin-guanine dinucleotide biosynthesis protein MobA
contig_46	fig|6666666.28487.peg.3635	Protein	46776	46919	+	hypothetical protein
contig_46	fig|6666666.28487.peg.3636	Protein	47264	47587	+	PROBABLE RESUSCITATION-PROMOTING FACTOR RPFE
contig_46	fig|6666666.28487.peg.3637	Protein	47828	48328	+	PROBABLE RESUSCITATION-PROMOTING FACTOR RPFE
contig_46	fig|6666666.28487.peg.3638	Protein	49503	48337	-	2-polyprenyl-6-methoxyphenol hydroxylase and related FAD-dependent oxidoreductases
contig_46	fig|6666666.28487.peg.3639	Protein	49629	50045	+	FIG00826409: hypothetical protein
contig_46	fig|6666666.28487.peg.3640	Protein	50164	50703	+	Flavodoxin reductases (ferredoxin-NADPH reductases) family 1; Vanillate O-demethylase oxidoreductase (EC 1.14.13.-)
contig_46	fig|6666666.28487.peg.3641	Protein	50857	51705	+	FIG00823441: hypothetical protein
contig_46	fig|6666666.28487.peg.3642	Protein	51987	51769	-	FIG00829073: hypothetical protein
contig_46	fig|6666666.28487.peg.3643	Protein	53370	51988	-	Ammonium transporter
contig_46	fig|6666666.28487.peg.3644	Protein	53503	55491	+	Peptidase S9 prolyl oligopeptidase
contig_46	fig|6666666.28487.peg.3645	Protein	55502	56782	+	putative membrane protein
contig_46	fig|6666666.28487.peg.3646	Protein	56827	57414	+	FIG00824513: hypothetical protein
contig_46	fig|6666666.28487.peg.3647	Protein	57468	60152	+	Valyl-tRNA synthetase (EC 6.1.1.9)
contig_46	fig|6666666.28487.peg.3648	Protein	60168	61595	+	Dihydrofolate synthase (EC 6.3.2.12) / Folylpolyglutamate

						synthase (EC 6.3.2.17)
contig_46	fig|6666666.28487.peg.3649	Protein	61592	61975	+	Possible membrane protein
contig_46	fig|6666666.28487.peg.3650	Protein	62007	62417	+	Nucleoside diphosphate kinase (EC 2.7.4.6)
contig_46	fig|6666666.28487.peg.3651	Protein	62726	65731	+	Ribonuclease E (EC 3.1.26.12)
contig_46	fig|6666666.28487.peg.3652	Protein	65887	66210	+	LSU ribosomal protein L21p
contig_46	fig|6666666.28487.peg.3653	Protein	66228	66497	+	LSU ribosomal protein L27p
contig_46	fig|6666666.28487.peg.3654	Protein	66586	68040	+	COG0536: GTP-binding protein Obg
contig_46	fig|6666666.28487.peg.3655	Protein	68037	69143	+	Glutamate 5-kinase (EC 2.7.2.11) / RNA-binding C-terminal domain PUA
contig_46	fig|6666666.28487.peg.3656	Protein	69768	69133	-	Transcriptional regulator, TetR family
contig_46	fig|6666666.28487.peg.3657	Protein	69835	71100	+	putative cytochrome P450 hydroxylase
contig_46	fig|6666666.28487.peg.3658	Protein	71929	71090	-	NAD-dependent protein deacetylase of SIR2 family
contig_46	fig|6666666.28487.peg.3659	Protein	72532	71948	-	heat shock protein Hsp20
contig_46	fig|6666666.28487.peg.3660	Protein	72578	72703	+	hypothetical protein
contig_46	fig|6666666.28487.peg.3661	Protein	72732	74774	+	NAD synthetase (EC 6.3.1.5) / Glutamine amidotransferase chain of NAD synthetase
contig_47	fig|6666666.28487.peg.3662	Protein	1535	327	-	PE-PPE, C-terminal domain protein
contig_47	fig|6666666.28487.peg.3663	Protein	2454	1789	-	Probable cutinase Rv1984c/MT2037 precursor (EC 3.1.1.74)
contig_47	fig|6666666.28487.peg.3664	Protein	4002	2515	-	Cyclohexanone monooxygenase (EC 1.14.13.22)
contig_47	fig|6666666.28487.peg.3665	Protein	5427	4165	-	PE-PPE, C-terminal domain protein

contig_47	fig|6666666.28487.peg.3666	Protein	6030	6884	+	3-oxoacyl-[acyl-carrier protein] reductase (EC 1.1.1.100)
contig_47	fig|6666666.28487.peg.3667	Protein	8054	6864	-	Alkane-1 monooxygenase (EC 1.14.15.3)
contig_47	fig|6666666.28487.peg.3668	Protein	9873	8296	-	Prostaglandin-endoperoxide synthase(EC:1.14.99.1)
contig_47	fig|6666666.28487.peg.3669	Protein	11871	9928	-	FIG00996932: hypothetical protein
contig_47	fig|6666666.28487.peg.3670	Protein	14110	11918	-	Malate synthase G (EC 2.3.3.9)
contig_47	fig|6666666.28487.peg.3671	Protein	15080	14199	-	FIG00823203: hypothetical protein
contig_47	fig|6666666.28487.peg.3672	Protein	16134	15073	-	Magnesium and cobalt efflux protein CorC
contig_47	fig|6666666.28487.peg.3673	Protein	17485	16127	-	Magnesium and cobalt efflux protein CorC
contig_47	fig|6666666.28487.peg.3674	Protein	17810	18838	+	Ferric iron ABC transporter, iron-binding protein
contig_47	fig|6666666.28487.peg.3675	Protein	18844	20394	+	Ferric iron ABC transporter, permease protein
contig_47	fig|6666666.28487.peg.3676	Protein	20387	21478	+	Ferric iron ABC transporter, ATP-binding protein
contig_47	fig|6666666.28487.peg.3677	Protein	22876	21431	-	Inosine-5'-monophosphate dehydrogenase (EC 1.1.1.205)
contig_47	fig|6666666.28487.peg.3678	Protein	24426	22978	-	6-phosphogluconate dehydrogenase, decarboxylating (EC 1.1.1.44)
contig_47	fig|6666666.28487.peg.3679	Protein	25458	24523	-	Peptidase M48, Ste24p precursor
contig_47	fig|6666666.28487.peg.3680	Protein	25889	25473	-	Transcriptional regulator, BlaI family
contig_47	fig|6666666.28487.peg.3681	Protein	26157	26933	+	FIG00821226: hypothetical protein
contig_47	fig|6666666.28487.peg.3682	Protein	27128	27532	+	Putative esterase
contig_48	fig|6666666.28487.peg.3683	Protein	2826	1459	-	FIG00825092: hypothetical protein
contig_48	fig|6666666.28487.peg.3684	Protein	3926	2829	-	FIG00824533: hypothetical protein

contig_48	fig|6666666.28487.peg.3685	Protein	4006	5322	+	4-hydroxybutyrate coenzyme A transferase
contig_48	fig|6666666.28487.peg.3686	Protein	5417	6562	+	3-ketoacyl-CoA thiolase (EC 2.3.1.16) @ Acetyl-CoA acetyltransferase (EC 2.3.1.9)
contig_48	fig|6666666.28487.peg.3687	Protein	6559	7359	+	Enoyl-CoA hydratase (EC 4.2.1.17)
contig_48	fig|6666666.28487.peg.3688	Protein	7379	8521	+	acyl-CoA dehydrogenase domain protein
contig_49	fig|6666666.28487.peg.3689	Protein	110	598	+	Ornithine decarboxylase (EC 4.1.1.17) / Arginine decarboxylase (EC 4.1.1.19)
contig_49	fig|6666666.28487.peg.3690	Protein	682	1812	+	Coenzyme F420-dependent N5,N10-methylene tetrahydromethanopterin reductase and related flavin-dependent oxidoreductases; sulfonate monooxygenase
contig_49	fig|6666666.28487.peg.3691	Protein	2032	1916	-	hypothetical protein
contig_49	fig|6666666.28487.peg.3692	Protein	2018	2869	+	antigen 34 kDa
contig_49	fig|6666666.28487.peg.3693	Protein	2930	4414	+	FIG021574: Possible membrane protein related to de Novo purine biosynthesis
contig_49	fig|6666666.28487.peg.3694	Protein	4424	5053	+	Phosphoribosylglycinamide formyltransferase (EC 2.1.2.2)
contig_49	fig|6666666.28487.peg.3695	Protein	5050	6627	+	IMP cyclohydrolase (EC 3.5.4.10) / Phosphoribosylaminoimidazolecarboxamide formyltransferase (EC 2.1.2.3)
contig_49	fig|6666666.28487.peg.3696	Protein	6640	7317	+	FIG00827084: hypothetical protein
contig_49	fig|6666666.28487.peg.3697	Protein	7371	8765	+	Magnesium chelatase, subunit ChlI (EC 6.6.1.1)
contig_49	fig|6666666.28487.peg.3698	Protein	8758	10731	+	FIG019045: long form Mg-chelase associated protein with vWA domain
contig_49	fig|6666666.28487.peg.3699	Protein	11347	10736	-	FIG00821093: hypothetical protein

contig_49	fig|6666666.28487.peg.3700	Protein	12169	11411	-	Methylglutaconyl-CoA hydratase (EC 4.2.1.18)
contig_49	fig|6666666.28487.peg.3701	Protein	13370	12210	-	Isovaleryl-CoA dehydrogenase (EC 1.3.99.10)
contig_49	fig|6666666.28487.peg.3702	Protein	15385	13367	-	Methylcrotonyl-CoA carboxylase biotin-containing subunit (EC 6.4.1.4)
contig_49	fig|6666666.28487.peg.3703	Protein	17003	15411	-	Methylcrotonyl-CoA carboxylase carboxyl transferase subunit (EC 6.4.1.4)
contig_49	fig|6666666.28487.peg.3704	Protein	18148	17000	-	Isovaleryl-CoA dehydrogenase (EC 1.3.99.10)
contig_49	fig|6666666.28487.peg.3705	Protein	19878	18145	-	expressed protein
contig_49	fig|6666666.28487.peg.3706	Protein	20161	20334	+	LSU ribosomal protein L32p
contig_49	fig|6666666.28487.peg.3707	Protein	20407	21096	+	Mycobacterial persistence regulator MprA (Two component response transcriptional regulatory protein)
contig_49	fig|6666666.28487.peg.3708	Protein	21186	22541	+	sensor histidine kinase
contig_49	fig|6666666.28487.peg.3709	Protein	23049	24125	+	Heat shock protein HtrA
contig_49	fig|6666666.28487.peg.3710	Protein	24203	24700	+	Molybdenum cofactor biosynthesis protein MoaB
contig_49	fig|6666666.28487.peg.3711	Protein	24862	25020	+	hypothetical protein
contig_5	fig|6666666.28487.peg.3712	Protein	58	354	+	prolipoprotein diacylglyceryl transferase
contig_5	fig|6666666.28487.peg.3713	Protein	744	361	-	putative hydrolase
contig_5	fig|6666666.28487.peg.3714	Protein	1273	2697	+	CONSERVED 13E12 REPEAT FAMILY PROTEIN
contig_5	fig|6666666.28487.peg.3715	Protein	2727	3176	+	FIG00822583: hypothetical protein
contig_5	fig|6666666.28487.peg.3716	Protein	3229	3579	+	CONSERVED MEMBRANE PROTEIN
contig_5	fig|6666666.28487.peg.3717	Protein	3657	8204	+	Glutamate synthase [NADPH] large chain (EC 1.4.1.13)

contig_5	fig|6666666.28487.peg.3718	Protein	8197	9705	+	Glutamate synthase [NADPH] small chain (EC 1.4.1.13)
contig_5	fig|6666666.28487.peg.3719	Protein	9817	11151	+	Pyruvate kinase (EC 2.7.1.40)
contig_5	fig|6666666.28487.peg.3720	Protein	11171	12031	+	Acyl-CoA thioesterase II (EC 3.1.2.-)
contig_5	fig|6666666.28487.peg.3721	Protein	12511	12038	-	putative integral membrane protein
contig_5	fig|6666666.28487.peg.3722	Protein	13944	12508	-	Transport ATP-binding protein CydC
contig_5	fig|6666666.28487.peg.3723	Protein	15553	13970	-	Transport ATP-binding protein CydD
contig_5	fig|6666666.28487.peg.3724	Protein	16626	15586	-	Cytochrome d ubiquinol oxidase subunit II (EC 1.10.3.-)
contig_5	fig|6666666.28487.peg.3725	Protein	18105	16636	-	Cytochrome d ubiquinol oxidase subunit I (EC 1.10.3.-)
contig_5	fig|6666666.28487.peg.3726	Protein	18751	18200	-	Possible membrane protein
contig_5	fig|6666666.28487.peg.3727	Protein	18973	19893	+	amino acid ABC transporter, amino acid-binding-permease protein (glnP)
contig_5	fig|6666666.28487.peg.3728	Protein	19909	20835	+	amino acid ABC transporter, permease protein (glnP)
contig_5	fig|6666666.28487.peg.3729	Protein	20873	21625	+	ATP-binding protein
contig_5	fig|6666666.28487.peg.3730	Protein	21622	22737	+	sensor kinase, two-component system
contig_5	fig|6666666.28487.peg.3731	Protein	22713	23366	+	putative two-component system response regulator
contig_5	fig|6666666.28487.peg.3732	Protein	23411	25243	+	hypothetical membrane protein
contig_5	fig|6666666.28487.peg.3733	Protein	25379	25789	+	Non-specific DNA-binding protein Dps / Iron-binding ferritin-like antioxidant protein / Ferroxidase (EC 1.16.3.1)
contig_5	fig|6666666.28487.peg.3734	Protein	27038	25764	-	Adenylate cyclase (EC 4.6.1.1)
contig_5	fig|6666666.28487.peg.3735	Protein	27078	27875	+	hypothetical protein

contig_5	fig|6666666.28487.peg.3736	Protein	28094	27885	-	hypothetical protein
contig_5	fig|6666666.28487.rna.24	RNA	28264	28191	-	tRNA-Leu-CAA
contig_5	fig|6666666.28487.peg.3737	Protein	28355	28996	+	response regulator
contig_5	fig|6666666.28487.peg.3738	Protein	29192	29064	-	hypothetical protein
contig_5	fig|6666666.28487.peg.3739	Protein	29210	30421	+	Branched-chain amino acid ABC transporter, amino acid-binding protein (TC 3.A.1.4.1)
contig_5	fig|6666666.28487.peg.3740	Protein	30514	31524	+	High-affinity branched-chain amino acid transport system permease protein LivH (TC 3.A.1.4.1)
contig_5	fig|6666666.28487.peg.3741	Protein	31542	32678	+	Branched-chain amino acid transport system permease protein LivM (TC 3.A.1.4.1)
contig_5	fig|6666666.28487.peg.3742	Protein	32675	33550	+	Branched-chain amino acid transport ATP-binding protein LivG (TC 3.A.1.4.1)
contig_5	fig|6666666.28487.peg.3743	Protein	33537	34277	+	Branched-chain amino acid transport ATP-binding protein LivF (TC 3.A.1.4.1)
contig_5	fig|6666666.28487.peg.3744	Protein	34539	34366	-	hypothetical protein
contig_5	fig|6666666.28487.peg.3745	Protein	35880	34681	-	Probable nonspecific lipid-transfer protein
contig_5	fig|6666666.28487.peg.3746	Protein	36311	35877	-	hypothetical protein
contig_5	fig|6666666.28487.peg.3747	Protein	36442	36828	+	hypothetical protein
contig_5	fig|6666666.28487.peg.3748	Protein	36858	37265	+	hypothetical protein
contig_5	fig|6666666.28487.peg.3749	Protein	37941	37267	-	salicylate esterase
contig_5	fig|6666666.28487.peg.3750	Protein	38070	38951	+	transcriptional regulator, LysR family

contig_5	fig|6666666.28487.peg.3751	Protein	39001	41730	+	DNA polymerase I (EC 2.7.7.7)
contig_5	fig|6666666.28487.peg.3752	Protein	42794	41727	-	putative membrane protein
contig_5	fig|6666666.28487.peg.3753	Protein	43064	44515	+	SSU ribosomal protein S1p
contig_5	fig|6666666.28487.peg.3754	Protein	44606	45787	+	Dephospho-CoA kinase (EC 2.7.1.24)
contig_5	fig|6666666.28487.peg.3755	Protein	46335	45784	-	FIG00822423: hypothetical protein
contig_5	fig|6666666.28487.peg.3756	Protein	46770	46345	-	FIG00996124: hypothetical protein
contig_5	fig|6666666.28487.peg.3757	Protein	46918	49083	+	Excinuclease ABC subunit B
contig_5	fig|6666666.28487.peg.3758	Protein	49123	50535	+	Multidrug resistance protein B
contig_5	fig|6666666.28487.rna.25	RNA	50584	50654	+	tRNA-Cys-GCA
contig_5	fig|6666666.28487.peg.3759	Protein	50825	51154	+	Ethidium bromide-methyl viologen resistance protein EmrE
contig_5	fig|6666666.28487.peg.3760	Protein	51151	51474	+	Ethidium bromide-methyl viologen resistance protein EmrE
contig_5	fig|6666666.28487.peg.3761	Protein	51643	52281	+	Probable cutinase Rv1984c/MT2037 precursor (EC 3.1.1.74)
contig_5	fig|6666666.28487.peg.3762	Protein	52449	52580	+	hypothetical protein
contig_5	fig|6666666.28487.peg.3763	Protein	52912	53355	+	Universal stress protein UspA and related nucleotide-binding proteins
contig_5	fig|6666666.28487.peg.3764	Protein	54298	53414	-	Fructose-bisphosphate aldolase class I (EC 4.1.2.13)
contig_5	fig|6666666.28487.peg.3765	Protein	54518	54333	-	metallo-beta-lactamase superfamily protein
contig_5	fig|6666666.28487.peg.3766	Protein	54534	55988	+	Recombinase
contig_5	fig|6666666.28487.peg.3767	Protein	56314	56643	+	hypothetical protein
contig_5	fig|6666666.28487.peg.3768	Protein	56636	56914	+	hypothetical protein

contig_5	fig|6666666.28487.peg.3769	Protein	56911	58020	+	DNA helicase
contig_5	fig|6666666.28487.peg.3770	Protein	58271	58552	+	hypothetical protein
contig_5	fig|6666666.28487.peg.3771	Protein	58579	58770	+	hypothetical protein
contig_5	fig|6666666.28487.peg.3772	Protein	58767	59042	+	hypothetical protein
contig_5	fig|6666666.28487.peg.3773	Protein	59361	59137	-	hypothetical protein
contig_5	fig|6666666.28487.peg.3774	Protein	59416	60873	+	phage Terminase
contig_5	fig|6666666.28487.peg.3775	Protein	60870	62174	+	Phage capsid and scaffold
contig_5	fig|6666666.28487.peg.3776	Protein	62174	62680	+	hypothetical protein
contig_5	fig|6666666.28487.peg.3777	Protein	62791	63660	+	hypothetical protein
contig_5	fig|6666666.28487.peg.3778	Protein	63663	64604	+	hypothetical protein
contig_5	fig|6666666.28487.peg.3779	Protein	65388	64711	-	metallo-beta-lactamase superfamily protein
contig_5	fig|6666666.28487.peg.3780	Protein	65520	68426	+	Excinuclease ABC subunit A
contig_5	fig|6666666.28487.peg.3781	Protein	68464	68937	+	GCN5-related N-acetyltransferase
contig_5	fig|6666666.28487.peg.3782	Protein	70133	68934	-	hypothetical protein
contig_5	fig|6666666.28487.peg.3783	Protein	70265	70765	+	Lipoprotein LpqH precursor (19 kDa lipoprotein antigen)
contig_5	fig|6666666.28487.peg.3784	Protein	70830	72470	+	FIG00820671: hypothetical protein
contig_5	fig|6666666.28487.peg.3785	Protein	72467	73663	+	FIG00828757: hypothetical protein
contig_5	fig|6666666.28487.peg.3786	Protein	74011	73667	-	hypothetical protein
contig_5	fig|6666666.28487.peg.3787	Protein	74067	75353	+	FIG00828859: hypothetical protein

contig_5	fig|6666666.28487.peg.3788	Protein	75350	76438	+	FIG00828098: hypothetical protein
contig_5	fig|6666666.28487.peg.3789	Protein	76704	76432	-	FIG00820127: hypothetical protein
contig_5	fig|6666666.28487.peg.3790	Protein	78070	76715	-	FIG00824695: hypothetical protein
contig_5	fig|6666666.28487.peg.3791	Protein	81430	78125	-	Putative membrane protein found fused to lysyl-tRNA synthetase like protein / Lysyl-tRNA synthetase (class II) related protein found fused to membrane protein
contig_5	fig|6666666.28487.peg.3792	Protein	81401	81556	+	hypothetical protein
contig_5	fig|6666666.28487.peg.3793	Protein	81852	81517	-	RecA/RadA recombinase
contig_5	fig|6666666.28487.peg.3794	Protein	82039	82692	+	Translation initiation factor 3
contig_5	fig|6666666.28487.peg.3795	Protein	82699	82893	+	LSU ribosomal protein L35p
contig_5	fig|6666666.28487.peg.3796	Protein	82939	83328	+	LSU ribosomal protein L20p
contig_5	fig|6666666.28487.peg.3797	Protein	83352	84098	+	tRNA/rRNA methyltransferase (SpoU)
contig_5	fig|6666666.28487.peg.3798	Protein	84378	84845	+	PE-PPE, C-terminal domain protein
contig_5	fig|6666666.28487.peg.3799	Protein	84909	85532	+	PE-PPE, C-terminal domain protein
contig_5	fig|6666666.28487.peg.3800	Protein	85546	85920	+	GatB/YqeY
contig_5	fig|6666666.28487.peg.3801	Protein	86784	86002	-	Oxidoreductase, short chain dehydrogenase/reductase family protein
contig_5	fig|6666666.28487.peg.3802	Protein	88051	86816	-	acyl-CoA dehydrogenase domain protein
contig_5	fig|6666666.28487.peg.3803	Protein	89051	88056	-	FIG00820092: hypothetical protein
contig_5	fig|6666666.28487.peg.3804	Protein	89256	90119	+	Purine cyclase-related protein
contig_5	fig|6666666.28487.peg.3805	Protein	90119	90898	+	transmembrane protein

contig_5	fig|6666666.28487.peg.3806	Protein	92461	90881	-	Ferredoxin-dependent glutamate synthase (EC 1.4.7.1)
contig_5	fig|6666666.28487.peg.3807	Protein	92534	93577	+	Phenylalanyl-tRNA synthetase alpha chain (EC 6.1.1.20)
contig_5	fig|6666666.28487.peg.3808	Protein	93577	96063	+	Phenylalanyl-tRNA synthetase beta chain (EC 6.1.1.20)
contig_5	fig|6666666.28487.peg.3809	Protein	96120	96959	+	hypothetical protein
contig_5	fig|6666666.28487.peg.3810	Protein	97801	96956	-	fumarylacetoacetate (FAA) hydrolase
contig_5	fig|6666666.28487.peg.3811	Protein	98620	97832	-	3-oxoacyl-[acyl-carrier protein] reductase (EC 1.1.1.100)
contig_5	fig|6666666.28487.peg.3812	Protein	99529	98630	-	Dihydrodipicolinate synthase family
contig_5	fig|6666666.28487.peg.3813	Protein	100734	99526	-	L-2-hydroxyglutarate oxidase (EC 1.1.3.15)
contig_5	fig|6666666.28487.peg.3814	Protein	101906	100731	-	L-carnitine dehydratase/bile acid-inducible protein F
contig_5	fig|6666666.28487.peg.3815	Protein	102403	101906	-	putative ammonia monooxygenase
contig_5	fig|6666666.28487.peg.3816	Protein	103965	102403	-	Tricarboxylate transport membrane protein TctA
contig_5	fig|6666666.28487.peg.3817	Protein	104500	103976	-	hypothetical protein
contig_5	fig|6666666.28487.peg.3818	Protein	105534	104554	-	hypothetical protein
contig_5	fig|6666666.28487.peg.3819	Protein	106026	105574	-	hypothetical protein
contig_5	fig|6666666.28487.peg.3820	Protein	106814	106023	-	Enoyl-CoA hydratase (EC 4.2.1.17)
contig_5	fig|6666666.28487.peg.3821	Protein	108034	106811	-	Putative L-carnitine dehydrogenase-like protein
contig_5	fig|6666666.28487.peg.3822	Protein	108188	109027	+	Transcriptional regulator, IclR family
contig_5	fig|6666666.28487.peg.3823	Protein	109094	110128	+	N-acetyl-gamma-glutamyl-phosphate reductase (EC 1.2.1.38)
contig_5	fig|6666666.28487.peg.3824	Protein	110125	111321	+	Glutamate N-acetyltransferase (EC 2.3.1.35) / N-acetylglutamate synthase (EC 2.3.1.1)

contig_5	fig|6666666.28487.peg.3825	Protein	111318	112190	+	Acetylglutamate kinase (EC 2.7.2.8)
contig_5	fig|6666666.28487.peg.3826	Protein	112187	113395	+	Acetylornithine aminotransferase (EC 2.6.1.11)
contig_5	fig|6666666.28487.peg.3827	Protein	113392	114315	+	Ornithine carbamoyltransferase (EC 2.1.3.3)
contig_5	fig|6666666.28487.peg.3828	Protein	114312	114797	+	Arginine pathway regulatory protein ArgR, repressor of arg regulon
contig_5	fig|6666666.28487.peg.3829	Protein	114825	116027	+	Argininosuccinate synthase (EC 6.3.4.5)
contig_5	fig|6666666.28487.peg.3830	Protein	116024	117436	+	Argininosuccinate lyase (EC 4.3.2.1)
contig_5	fig|6666666.28487.peg.3831	Protein	117550	118074	+	FIG00826229: hypothetical protein
contig_5	fig|6666666.28487.peg.3832	Protein	118101	118640	+	FIG00825105: hypothetical protein
contig_5	fig|6666666.28487.peg.3833	Protein	118637	119140	+	FIG00829448: hypothetical protein
contig_5	fig|6666666.28487.peg.3834	Protein	120401	119118	-	Putative membrane protein
contig_5	fig|6666666.28487.peg.3835	Protein	122209	120398	-	ABC transporter, ATP-binding protein
contig_5	fig|6666666.28487.peg.3836	Protein	122323	125301	+	Polyhydroxyalkanoic acid synthase
contig_5	fig|6666666.28487.peg.3837	Protein	125366	125902	+	FIG00827124: hypothetical protein
contig_5	fig|6666666.28487.peg.3838	Protein	125907	126113	+	UPF0434 protein YcaR
contig_5	fig|6666666.28487.peg.3839	Protein	126730	126104	-	Transcriptional regulator, TetR family
contig_5	fig|6666666.28487.peg.3840	Protein	127425	126736	-	"ABC drug efflux pump, inner membrane subunit, DrrB family"
contig_5	fig|6666666.28487.peg.3841	Protein	128270	127506	-	ABC transporter related
contig_5	fig|6666666.28487.peg.3842	Protein	128320	128940	+	DNA-3-methyladenine glycosylase II (EC 3.2.2.21)
contig_5	fig|6666666.28487.peg.3843	Protein	128959	130233	+	Tyrosyl-tRNA synthetase (EC 6.1.1.1)

contig_50	fig|6666666.28487.peg.3844	Protein	14	814	+	Mobile element protein
contig_50	fig|6666666.28487.peg.3845	Protein	1362	2021	+	putative two-component system response regulator
contig_50	fig|6666666.28487.peg.3846	Protein	2023	3459	+	hypothetical protein
contig_50	fig|6666666.28487.peg.3847	Protein	3580	4947	+	dicarboxylic acid transporter PcaT
contig_50	fig|6666666.28487.peg.3848	Protein	4976	5902	+	TRAP transporter solute receptor, TAXI family precursor
contig_50	fig|6666666.28487.peg.3849	Protein	6220	5909	-	FIG00822268: hypothetical protein
contig_50	fig|6666666.28487.peg.3850	Protein	6335	6213	-	hypothetical protein
contig_50	fig|6666666.28487.peg.3851	Protein	6434	6613	+	hypothetical protein
contig_50	fig|6666666.28487.peg.3852	Protein	6937	6680	-	FIG00825176: hypothetical protein
contig_50	fig|6666666.28487.peg.3853	Protein	8025	7003	-	Hypothetical protein PA2244 (similar to DNA topoisomerase IB, but possibly involved in glycosyl-transfer)
contig_50	fig|6666666.28487.peg.3854	Protein	9252	8104	-	FIG00825677: hypothetical protein
contig_50	fig|6666666.28487.peg.3855	Protein	9389	9904	+	flavin reductase domain protein, FMN-binding
contig_50	fig|6666666.28487.peg.3856	Protein	9901	10617	+	5-amino-6-(5-phosphoribosylamino)uracil reductase (EC 1.1.1.193) homolog
contig_50	fig|6666666.28487.peg.3857	Protein	10884	10606	-	Enoyl-[acyl-carrier-protein] reductase [NADPH] (EC 1.3.1.10)
contig_50	fig|6666666.28487.peg.3858	Protein	11442	10912	-	FIG00829970: hypothetical protein
contig_50	fig|6666666.28487.peg.3859	Protein	11550	11825	+	putative integral membrane protein
contig_50	fig|6666666.28487.peg.3860	Protein	11979	12164	+	hypothetical protein
contig_50	fig|6666666.28487.peg.3861	Protein	12926	12168	-	hypothetical protein
contig_50	fig|6666666.28487.peg.3862	Protein	13243	14913	+	Potassium-transporting ATPase A chain (EC 3.6.3.12) (TC

						3.A.3.7.1)
contig_50	fig|6666666.28487.peg.3863	Protein	14915	17071	+	Potassium-transporting ATPase B chain (EC 3.6.3.12) (TC 3.A.3.7.1)
contig_50	fig|6666666.28487.peg.3864	Protein	17073	17933	+	Potassium-transporting ATPase C chain (EC 3.6.3.12) (TC 3.A.3.7.1)
contig_50	fig|6666666.28487.peg.3865	Protein	17961	20522	+	Osmosensitive K+ channel histidine kinase KdpD (EC 2.7.3.-)
contig_50	fig|6666666.28487.peg.3866	Protein	20519	21217	+	DNA-binding response regulator KdpE
contig_50	fig|6666666.28487.peg.3867	Protein	21687	21220	-	hypothetical protein
contig_50	fig|6666666.28487.peg.3868	Protein	21832	22725	+	ABC transporter, ATP-binding protein
contig_50	fig|6666666.28487.peg.3869	Protein	22722	23426	+	PROBABLE ANTIBIOTIC-TRANSPORT INTEGRAL MEMBRANE LEUCINE AND VALINE RICH PROTEIN ABC TRANSPORTER
contig_50	fig|6666666.28487.peg.3870	Protein	23510	24178	+	PROBABLE ANTIBIOTIC-TRANSPORT INTEGRAL MEMBRANE LEUCINE AND ALANINE AND VALINE RICH PROTEIN ABC TRANSPORTER
contig_50	fig|6666666.28487.peg.3871	Protein	24277	24441	+	hypothetical protein
contig_50	fig|6666666.28487.peg.3872	Protein	25328	24528	-	Epoxide hydrolase (EC 3.3.2.9)
contig_50	fig|6666666.28487.peg.3873	Protein	25438	27309	+	Acetyl-coenzyme A synthetase (EC 6.2.1.1)
contig_50	fig|6666666.28487.peg.3874	Protein	27390	27857	+	FIG00825373: hypothetical protein
contig_50	fig|6666666.28487.peg.3875	Protein	28861	27926	-	Exopolyphosphatase (EC 3.6.1.11)
contig_50	fig|6666666.28487.peg.3876	Protein	29289	28858	-	FIG004853: possible toxin to DivIC
contig_50	fig|6666666.28487.peg.3877	Protein	30061	29342	-	Cell division protein DivIC (FtsB), stabilizes FtsL against RasP

						cleavage
contig_50	fig|6666666.28487.peg.3878	Protein	31354	30065	-	Enolase (EC 4.2.1.11)
contig_50	fig|6666666.28487.peg.3879	Protein	32158	31427	-	FIG00997322: hypothetical protein
contig_50	fig|6666666.28487.peg.3880	Protein	32336	33256	+	Ferrous iron transport permease EfeU
contig_50	fig|6666666.28487.peg.3881	Protein	33253	34416	+	Ferrous iron transport periplasmic protein EfeO, contains peptidase-M75 domain and (frequently) cupredoxin-like domain
contig_50	fig|6666666.28487.peg.3882	Protein	34397	35713	+	Ferrous iron transport peroxidase EfeB
contig_50	fig|6666666.28487.peg.3883	Protein	36842	35799	-	Nucleoside triphosphate pyrophosphohydrolase MazG (EC 3.6.1.8)
contig_50	fig|6666666.28487.peg.3884	Protein	40500	36901	-	Transcription-repair coupling factor
contig_50	fig|6666666.28487.peg.3885	Protein	40637	41875	+	hypothetical protein
contig_50	fig|6666666.28487.peg.3886	Protein	41876	42394	+	hypothetical protein
contig_50	fig|6666666.28487.peg.3887	Protein	42991	42398	-	Transcriptional regulator, TetR family
contig_50	fig|6666666.28487.peg.3888	Protein	43093	44604	+	Regulator of polyketide synthase expression
contig_50	fig|6666666.28487.peg.3889	Protein	44880	45332	+	Transcription elongation factor GreA
contig_50	fig|6666666.28487.rna.26	RNA	45385	45456	+	tRNA-Gln-TTG
contig_50	fig|6666666.28487.peg.3890	Protein	45597	47063	+	N-acetylglucosamine-1-phosphate uridyltransferase (EC 2.7.7.23) / Glucosamine-1-phosphate N-acetyltransferase (EC 2.3.1.157)
contig_50	fig|6666666.28487.peg.3891	Protein	47168	48148	+	Ribose-phosphate pyrophosphokinase (EC 2.7.6.1)

contig_50	fig|6666666.28487.peg.3892	Protein	48176	48520	+	Arsenate reductase (EC 1.20.4.1)
contig_50	fig|6666666.28487.peg.3893	Protein	48517	49167	+	lipoprotein, putative
contig_50	fig|6666666.28487.peg.3894	Protein	49242	49733	+	Short-chain dehydrogenase/reductase SDR
contig_50	fig|6666666.28487.peg.3895	Protein	49815	50123	+	Short-chain dehydrogenase/reductase SDR
contig_50	fig|6666666.28487.peg.3896	Protein	50287	50952	+	LSU ribosomal protein L25p
contig_50	fig|6666666.28487.peg.3897	Protein	50965	51543	+	Peptidyl-tRNA hydrolase (EC 3.1.1.29)
contig_50	fig|6666666.28487.peg.3898	Protein	51602	51775	+	hypothetical protein
contig_50	fig|6666666.28487.peg.3899	Protein	52208	51801	-	hypothetical protein
contig_50	fig|6666666.28487.peg.3900	Protein	53364	52336	-	Iron-sulfur cluster-binding protein
contig_50	fig|6666666.28487.peg.3901	Protein	53480	53989	+	hypothetical protein
contig_50	fig|6666666.28487.peg.3902	Protein	54840	53986	-	PROBABLE DAUNORUBICIN-DIM-TRANSPORT INTEGRAL MEMBRANE PROTEIN ABC TRANSPORTER DRRC
contig_50	fig|6666666.28487.peg.3903	Protein	55829	54837	-	putative ABC transporter ATP-binding protein
contig_50	fig|6666666.28487.peg.3904	Protein	57639	55969	-	Long-chain-fatty-acid--CoA ligase (EC 6.2.1.3)
contig_50	fig|6666666.28487.peg.3905	Protein	58829	57924	-	4-diphosphocytidyl-2-C-methyl-D-erythritol kinase (EC 2.7.1.148)
contig_50	fig|6666666.28487.peg.3906	Protein	59864	59031	-	SSU rRNA (adenine(1518)-N(6)/adenine(1519)-N(6))-dimethyltransferase (EC 2.1.1.182)
contig_50	fig|6666666.28487.peg.3907	Protein	61087	59969	-	Cell wall-binding protein
contig_50	fig|6666666.28487.peg.3908	Protein	62037	61189	-	Putative deoxyribonuclease YcfH
contig_50	fig|6666666.28487.peg.3909	Protein	62076	63611	+	Methionyl-tRNA synthetase (EC 6.1.1.10)

contig_50	fig|6666666.28487.peg.3910	Protein	63718	64911	+	NADH dehydrogenase (EC 1.6.99.3)
contig_50	fig|6666666.28487.peg.3911	Protein	64908	65804	+	RNA polymerase, sigma-24 subunit, ECF subfamily
contig_50	fig|6666666.28487.peg.3912	Protein	65812	67059	+	Para-aminobenzoate synthase, aminase component (EC 2.6.1.85)
contig_50	fig|6666666.28487.peg.3913	Protein	67802	67029	-	rRNA small subunit methyltransferase I
contig_50	fig|6666666.28487.peg.3914	Protein	67997	69478	+	CONSERVED MEMBRANE PROTEIN
contig_50	fig|6666666.28487.peg.3915	Protein	70777	69569	-	Arginine deiminase (EC 3.5.3.6)
contig_50	fig|6666666.28487.peg.3916	Protein	70824	71258	+	Redox-sensitive transcriptional activator SoxR
contig_50	fig|6666666.28487.peg.3917	Protein	71293	71901	+	Alkylated DNA repair protein AlkB
contig_50	fig|6666666.28487.peg.3918	Protein	72706	72029	-	FIG00820856: hypothetical protein
contig_50	fig|6666666.28487.peg.3919	Protein	73394	72735	-	FIG00821923: hypothetical protein
contig_50	fig|6666666.28487.peg.3920	Protein	74852	73431	-	PPE family protein
contig_50	fig|6666666.28487.peg.3921	Protein	75041	75754	+	PROBABLE CONSERVED LIPOPROTEIN LPQN
contig_50	fig|6666666.28487.peg.3922	Protein	76576	75758	-	Shikimate 5-dehydrogenase I gamma (EC 1.1.1.25)
contig_50	fig|6666666.28487.peg.3923	Protein	77574	76576	-	cyclic nucleotide-binding protein
contig_50	fig|6666666.28487.peg.3924	Protein	77735	78820	+	diguanylate cyclase
contig_50	fig|6666666.28487.peg.3925	Protein	78848	79276	+	FIG00831361: hypothetical protein
contig_50	fig|6666666.28487.peg.3926	Protein	79324	79812	+	Organic hydroperoxide resistance transcriptional regulator
contig_50	fig|6666666.28487.peg.3927	Protein	79832	80266	+	FIG00827504: hypothetical protein
contig_50	fig|6666666.28487.peg.3928	Protein	80313	81563	+	putative cytochrome P450 hydroxylase

contig_50	fig|6666666.28487.peg.3929	Protein	81789	82124	+	hypothetical protein
contig_50	fig|6666666.28487.peg.3930	Protein	82394	82137	-	conserved hypothetical protein
contig_50	fig|6666666.28487.peg.3931	Protein	82939	83229	+	hypothetical protein
contig_50	fig|6666666.28487.peg.3932	Protein	83405	83593	+	hypothetical protein
contig_50	fig|6666666.28487.peg.3933	Protein	83650	84321	+	hypothetical protein
contig_50	fig|6666666.28487.peg.3934	Protein	85758	85243	-	hypothetical protein
contig_50	fig|6666666.28487.peg.3935	Protein	86667	85780	-	hypothetical protein
contig_50	fig|6666666.28487.peg.3936	Protein	87105	86725	-	hypothetical protein
contig_50	fig|6666666.28487.peg.3937	Protein	87692	87102	-	hypothetical protein
contig_50	fig|6666666.28487.peg.3938	Protein	88180	87695	-	hypothetical protein
contig_50	fig|6666666.28487.peg.3939	Protein	89040	88177	-	hypothetical protein
contig_50	fig|6666666.28487.peg.3940	Protein	89261	89037	-	hypothetical protein
contig_50	fig|6666666.28487.peg.3941	Protein	89720	89550	-	hypothetical protein
contig_50	fig|6666666.28487.peg.3942	Protein	90190	89762	-	hypothetical protein
contig_50	fig|6666666.28487.peg.3943	Protein	90317	90460	+	hypothetical protein
contig_50	fig|6666666.28487.peg.3944	Protein	91076	90894	-	hypothetical protein
contig_50	fig|6666666.28487.peg.3945	Protein	91491	92750	+	Integrase
contig_50	fig|6666666.28487.rna.27	RNA	92857	92785	-	tRNA-Ala-CGC
contig_50	fig|6666666.28487.peg.3946	Protein	93948	92911	-	PROBABLE CONSERVED TRANSMEMBRANE PROTEIN

contig_50	fig|6666666.28487.peg.3947	Protein	94767	94102	-	Ribosomal-protein-S5p-alanine acetyltransferase
contig_50	fig|6666666.28487.peg.3948	Protein	96057	94777	-	Molybdopterin biosynthesis protein MoeA
contig_50	fig|6666666.28487.peg.3949	Protein	97091	96120	-	UTP--glucose-1-phosphate uridylyltransferase (EC 2.7.7.9)
contig_50	fig|6666666.28487.peg.3950	Protein	97157	97738	+	5-formyltetrahydrofolate cyclo-ligase (EC 6.3.3.2)
contig_50	fig|6666666.28487.peg.3951	Protein	98190	97981	-	hypothetical protein
contig_50	fig|6666666.28487.peg.3952	Protein	98245	98898	+	Heat shock protein 22.5 (Hsp22.5)
contig_50	fig|6666666.28487.peg.3953	Protein	99254	98961	-	hypothetical protein
contig_50	fig|6666666.28487.peg.3954	Protein	99634	100065	+	Large-conductance mechanosensitive channel
contig_51	fig|6666666.28487.peg.3955	Protein	1207	677	-	FIG00945083: hypothetical protein
contig_51	fig|6666666.28487.peg.3956	Protein	1329	2018	+	hypothetical protein
contig_51	fig|6666666.28487.peg.3957	Protein	2977	2015	-	Citrate lyase beta chain (EC 4.1.3.6)
contig_51	fig|6666666.28487.peg.3958	Protein	3016	4314	+	Mg/Co/Ni transporter MgtE / CBS domain
contig_51	fig|6666666.28487.peg.3959	Protein	4311	4844	+	PROBABLE MEMBRANE PROTEIN
contig_51	fig|6666666.28487.peg.3960	Protein	5132	4851	-	hypothetical protein
contig_51	fig|6666666.28487.peg.3961	Protein	5097	6311	+	POSSIBLE MEMBRANE PROTEIN
contig_51	fig|6666666.28487.peg.3962	Protein	6361	7491	+	Mrp protein homolog
contig_51	fig|6666666.28487.peg.3963	Protein	7934	7488	-	Twin-arginine translocation protein TatB
contig_51	fig|6666666.28487.peg.3964	Protein	9427	7937	-	serine protease
contig_51	fig|6666666.28487.peg.3965	Protein	9907	9509	-	FIG00820548: hypothetical protein

contig_51	fig|6666666.28487.peg.3966	Protein	10801	10001	-	RNA polymerase sigma-70 factor, ECF subfamily
contig_51	fig|6666666.28487.peg.3967	Protein	10900	11649	+	O-methyltransferase
contig_51	fig|6666666.28487.peg.3968	Protein	11904	12563	+	Transcriptional regulator, TetR family
contig_51	fig|6666666.28487.peg.3969	Protein	12556	13461	+	ABC-type multidrug transport system, ATPase component
contig_51	fig|6666666.28487.peg.3970	Protein	13458	15074	+	PROBABLE TETRONASIN-TRANSPORT INTEGRAL MEMBRANE PROTEIN ABC TRANSPORTER
contig_51	fig|6666666.28487.peg.3971	Protein	15166	15050	-	hypothetical protein
contig_51	fig|6666666.28487.peg.3972	Protein	15397	15759	+	Isoprenylcysteine carboxyl methyltransferase
contig_51	fig|6666666.28487.peg.3973	Protein	15796	16386	+	Probable transmembrane protein
contig_51	fig|6666666.28487.peg.3974	Protein	17764	16568	-	Glucose-1-phosphate adenylyltransferase (EC 2.7.7.27)
contig_51	fig|6666666.28487.peg.3975	Protein	17911	19074	+	Predicted glycogen synthase, ADP-glucose transglucosylase (EC 2.4.1.21), Actinobacterial type
contig_51	fig|6666666.28487.peg.3976	Protein	19254	19087	-	hypothetical protein
contig_51	fig|6666666.28487.peg.3977	Protein	20087	19479	-	DNA-3-methyladenine glycosylase (EC 3.2.2.20)
contig_51	fig|6666666.28487.peg.3978	Protein	20449	20084	-	FIG01000442: hypothetical protein
contig_51	fig|6666666.28487.peg.3979	Protein	21460	20501	-	Glycosyltransferases involved in cell wall biogenesis
contig_51	fig|6666666.28487.peg.3980	Protein	22332	21457	-	Non functional Dihydropteroate synthase 2
contig_51	fig|6666666.28487.peg.3981	Protein	24215	22410	-	Acetoacetyl-CoA synthetase (EC 6.2.1.16) / Long-chain-fatty-acid--CoA ligase (EC 6.2.1.3)
contig_51	fig|6666666.28487.peg.3982	Protein	24812	24249	-	Lysine decarboxylase family
contig_51	fig|6666666.28487.peg.3983	Protein	24871	26844	+	ATPase

contig_51	fig|6666666.28487.peg.3984	Protein	27044	26841	-	hypothetical protein
contig_51	fig|6666666.28487.peg.3985	Protein	27142	27894	+	hypothetical protein
contig_51	fig|6666666.28487.peg.3986	Protein	29498	27891	-	ABC transporter ATP-binding protein
contig_51	fig|6666666.28487.peg.3987	Protein	30639	29575	-	N-succinyl-L,L-diaminopimelate desuccinylase (EC 3.5.1.18)
contig_51	fig|6666666.28487.peg.3988	Protein	31886	30870	-	Branched-chain amino acid aminotransferase (EC 2.6.1.42)
contig_51	fig|6666666.28487.peg.3989	Protein	33432	31930	-	Urea carboxylase-related amino acid permease
contig_51	fig|6666666.28487.peg.3990	Protein	34446	33538	-	LysR family regulatory protein CidR
contig_51	fig|6666666.28487.peg.3991	Protein	34533	35486	+	2,3,4,5-tetrahydropyridine-2,6-dicarboxylate N-succinyltransferase (EC 2.3.1.117)
contig_51	fig|6666666.28487.peg.3992	Protein	36260	35622	-	D-alanyl-D-alanine carboxypeptidase (EC 3.4.16.4)
contig_51	fig|6666666.28487.peg.3993	Protein	37737	36319	-	Long-chain-fatty-acid--CoA ligase (EC 6.2.1.3)
contig_51	fig|6666666.28487.peg.3994	Protein	37779	38312	+	ADP-ribose pyrophosphatase (EC 3.6.1.13)
contig_51	fig|6666666.28487.peg.3995	Protein	39082	38309	-	probable taurine catabolism dioxygenase(EC:1.14.11.-)
contig_51	fig|6666666.28487.peg.3996	Protein	40107	39088	-	D-alanine--D-alanine ligase
contig_51	fig|6666666.28487.peg.3997	Protein	41464	40118	-	ATP-grasp ligase forming mycosporine-glycine, MysC
contig_51	fig|6666666.28487.peg.3998	Protein	42300	41461	-	O-methyltransferase MysB
contig_51	fig|6666666.28487.peg.3999	Protein	43520	42297	-	Demethyl 4-deoxygadusol synthase MysA
contig_51	fig|6666666.28487.peg.4000	Protein	44215	43550	-	Haloacid dehalogenase-like hydrolase
contig_51	fig|6666666.28487.peg.4001	Protein	44464	44994	+	regulatory protein, TetR
contig_51	fig|6666666.28487.peg.4002	Protein	45298	45681	+	FIG00821470: hypothetical protein

contig_51	fig|6666666.28487.peg.4003	Protein	49106	45699	-	Proline dehydrogenase (EC 1.5.99.8) (Proline oxidase) / Delta-1-pyrroline-5-carboxylate dehydrogenase (EC 1.5.1.12)
contig_51	fig|6666666.28487.peg.4004	Protein	49188	50111	+	putative transcriptional regulator, LysR family
contig_51	fig|6666666.28487.peg.4005	Protein	51232	50153	-	N-succinyl-L,L-diaminopimelate aminotransferase alternative (EC 2.6.1.17)
contig_51	fig|6666666.28487.peg.4006	Protein	51603	51280	-	4Fe-4S ferredoxin, iron-sulfur binding
contig_51	fig|6666666.28487.peg.4007	Protein	52746	51916	-	hypothetical protein
contig_51	fig|6666666.28487.peg.4008	Protein	52765	53310	+	Protein ycel precursor
contig_51	fig|6666666.28487.peg.4009	Protein	54260	53352	-	2-keto-4-pentenoate hydratase/2-oxohepta-3-ene-1,7-dioic acid hydratase (catechol pathway)
contig_51	fig|6666666.28487.peg.4010	Protein	54259	54414	+	hypothetical protein
contig_51	fig|6666666.28487.peg.4011	Protein	56990	54411	-	7,8-didemethyl-8-hydroxy-5-deazariboflavin synthase subunit 1 / 7,8-didemethyl-8-hydroxy-5-deazariboflavin synthase subunit 2
contig_51	fig|6666666.28487.peg.4012	Protein	57607	57176	-	FIG00820431: hypothetical protein
contig_51	fig|6666666.28487.peg.4013	Protein	58588	57722	-	N-acetyl-1-D-myo-inosityl-2-amino-2-deoxy-alpha-D-glucopyranoside deacetylase MshB
contig_51	fig|6666666.28487.peg.4014	Protein	58649	59896	+	Chloride channel protein
contig_51	fig|6666666.28487.peg.4015	Protein	61745	59880	-	LpqW
contig_51	fig|6666666.28487.peg.4016	Protein	63715	61781	-	GTP-binding protein TypA/BipA
contig_51	fig|6666666.28487.peg.4017	Protein	63745	63912	+	hypothetical protein
contig_51	fig|6666666.28487.peg.4018	Protein	63975	64247	+	hypothetical protein

contig_51	fig|6666666.28487.peg.4019	Protein	65363	64287	-	hypothetical protein
contig_51	fig|6666666.28487.peg.4020	Protein	65973	65575	-	mutT2 [EC:3.6.1.-]
contig_51	fig|6666666.28487.peg.4021	Protein	67339	66230	-	probable conserved membrane protein
contig_51	fig|6666666.28487.peg.4022	Protein	67605	68216	+	FIG01256903: hypothetical protein
contig_51	fig|6666666.28487.peg.4023	Protein	68276	69265	+	FIG00824206: hypothetical protein
contig_51	fig|6666666.28487.peg.4024	Protein	69496	69350	-	FIG00822951: hypothetical protein
contig_51	fig|6666666.28487.peg.4025	Protein	69547	70206	+	nitroreductase family protein
contig_51	fig|6666666.28487.peg.4026	Protein	70238	70630	+	Resolvase, N-terminal domain
contig_51	fig|6666666.28487.peg.4027	Protein	71240	70665	-	FIG00822543: hypothetical protein
contig_51	fig|6666666.28487.peg.4028	Protein	71287	72504	+	Autolysis histidine kinase LytS
contig_51	fig|6666666.28487.peg.4029	Protein	72501	73292	+	alginate biosynthesis regulatory protein AlgR (lytT)
contig_51	fig|6666666.28487.peg.4030	Protein	73345	73656	+	FIG00994049: hypothetical protein
contig_51	fig|6666666.28487.peg.4031	Protein	73653	75401	+	sodium:solute symporter, SSF
contig_51	fig|6666666.28487.peg.4032	Protein	75554	75904	+	INTEGRAL MEMBRANE PROTEIN (Rhomboid family)
contig_51	fig|6666666.28487.peg.4033	Protein	75901	77532	+	sodium:solute symporter protein
contig_51	fig|6666666.28487.peg.4034	Protein	77589	77720	+	hypothetical protein
contig_51	fig|6666666.28487.peg.4035	Protein	77692	78972	+	FIG00829297: hypothetical protein
contig_51	fig|6666666.28487.peg.4036	Protein	80708	80523	-	FIG00821392: hypothetical protein
contig_51	fig|6666666.28487.peg.4037	Protein	81190	80747	-	Pyridoxine 5'-phosphate oxidase, Rv1155

contig_51	fig|6666666.28487.peg.4038	Protein	81234	82091	+	O-methyltransferase-like protein
contig_51	fig|6666666.28487.peg.4039	Protein	82138	82959	+	omt (omt)
contig_51	fig|6666666.28487.peg.4040	Protein	83320	82982	-	Transcriptional regulator, GntR family
contig_51	fig|6666666.28487.peg.4041	Protein	83390	84100	+	NAD-dependent protein deacetylase of SIR2 family
contig_51	fig|6666666.28487.peg.4042	Protein	84811	84182	-	Methyltransferase (EC 2.1.1.-)
contig_51	fig|6666666.28487.peg.4043	Protein	85418	84792	-	Transcriptional regulator, TetR family
contig_51	fig|6666666.28487.peg.4044	Protein	87798	85480	-	Transmembrane transport protein MmpL13b
contig_51	fig|6666666.28487.peg.4045	Protein	87900	88445	+	Membrane protein 2, distant similarity to thiosulphate:quinone oxidoreductase DoxD
contig_51	fig|6666666.28487.peg.4046	Protein	88553	88684	+	FIG00821293: hypothetical protein
contig_51	fig|6666666.28487.peg.4047	Protein	89746	88754	-	Enoyl-[acyl-carrier-protein] reductase [FMN] (EC 1.3.1.9)
contig_51	fig|6666666.28487.peg.4048	Protein	90495	89743	-	3-hydroxyacyl-CoA dehydrogenase [isoleucine degradation] (EC 1.1.1.35)
contig_51	fig|6666666.28487.peg.4049	Protein	91591	90527	-	Alpha-methylacyl-CoA racemase (EC 5.1.99.4)
contig_51	fig|6666666.28487.peg.4050	Protein	91740	92561	+	Enoyl-CoA hydratase (EC 4.2.1.17)
contig_51	fig|6666666.28487.peg.4051	Protein	92671	93972	+	Tetracycline-resistance determinant tetV
contig_51	fig|6666666.28487.peg.4052	Protein	94679	93969	-	PROBABLE INTEGRAL MEMBRANE PROTEIN
contig_51	fig|6666666.28487.peg.4053	Protein	95773	94676	-	FIG00822654: hypothetical protein
contig_51	fig|6666666.28487.peg.4054	Protein	96398	95871	-	carbonic anhydrase, family 3
contig_51	fig|6666666.28487.peg.4055	Protein	96489	97442	+	FIG00831858: hypothetical protein

contig_51	fig|6666666.28487.peg.4056	Protein	97439	98038	+	Transcriptional regulator, TetR family
contig_51	fig|6666666.28487.peg.4057	Protein	98205	98942	+	Immunogenic protein MPB70 precursor
contig_52	fig|6666666.28487.rna.28	RNA	43	125	+	tRNA-Leu-TAG
contig_52	fig|6666666.28487.peg.4058	Protein	806	1216	+	hypothetical protein
contig_52	fig|6666666.28487.peg.4059	Protein	1357	1473	+	hypothetical protein
contig_52	fig|6666666.28487.peg.4060	Protein	2169	1555	-	helix-turn-helix- domain containing protein, AraC type
contig_52	fig|6666666.28487.peg.4061	Protein	2602	2351	-	FIG00829872: hypothetical protein
contig_52	fig|6666666.28487.peg.4062	Protein	2632	2757	+	hypothetical protein
contig_52	fig|6666666.28487.peg.4063	Protein	2818	3720	+	Alpha/beta hydrolase fold (EC 3.8.1.5)
contig_52	fig|6666666.28487.peg.4064	Protein	3998	3726	-	Transcriptional regulator WhiB-like WhiB6
contig_52	fig|6666666.28487.peg.4065	Protein	4103	4729	+	Transcriptional regulator, TetR family
contig_52	fig|6666666.28487.peg.4066	Protein	5524	4733	-	Transcriptional regulator, AraC family
contig_52	fig|6666666.28487.peg.4067	Protein	5626	6888	+	amidohydrolase 2
contig_52	fig|6666666.28487.peg.4068	Protein	8721	6898	-	Choline-sulfatase (EC 3.1.6.6)
contig_52	fig|6666666.28487.peg.4069	Protein	9158	8718	-	FIG00833435: hypothetical protein
contig_52	fig|6666666.28487.peg.4070	Protein	9789	9202	-	Transcriptional regulator, TetR family
contig_52	fig|6666666.28487.peg.4071	Protein	9896	10909	+	Dihydroflavonol-4-reductase (EC 1.1.1.219)
contig_52	fig|6666666.28487.peg.4072	Protein	10906	13128	+	PROBABLE ACYL-CoA DEHYDROGENASE FADE6 (EC 1.3.99.-)
contig_52	fig|6666666.28487.peg.4073	Protein	13141	14349	+	FIG00824044: hypothetical protein

contig_52	fig|6666666.28487.peg.4074	Protein	14352	14768	+	FIG00824333: hypothetical protein
contig_52	fig|6666666.28487.peg.4075	Protein	14824	15237	+	FIG00821410: hypothetical protein
contig_52	fig|6666666.28487.peg.4076	Protein	16791	15331	-	Adenylate cyclase (EC 4.6.1.1)
contig_52	fig|6666666.28487.peg.4077	Protein	17170	16868	-	FIG00820714: hypothetical protein
contig_52	fig|6666666.28487.peg.4078	Protein	18666	17167	-	Long-chain-fatty-acid--CoA ligase (EC 6.2.1.3)
contig_52	fig|6666666.28487.peg.4079	Protein	19505	18663	-	short chain dehydrogenase family protein [imported]
contig_52	fig|6666666.28487.peg.4080	Protein	19692	19549	-	hypothetical protein
contig_52	fig|6666666.28487.peg.4081	Protein	19696	21270	+	Methylmalonyl-CoA mutase (EC 5.4.99.2)
contig_52	fig|6666666.28487.peg.4082	Protein	21289	21687	+	B12 binding domain of Methylmalonyl-CoA mutase (EC 5.4.99.2)
contig_52	fig|6666666.28487.peg.4083	Protein	21773	22729	+	putative oxidoreductase
contig_52	fig|6666666.28487.peg.4084	Protein	22777	23784	+	L-carnitine dehydratase/bile acid-inducible protein F (EC 2.8.3.16)
contig_52	fig|6666666.28487.peg.4085	Protein	24101	23772	-	Probable phenylacetic acid degradation NADH oxidoreductase paaE (EC 1.-.-.-)
contig_52	fig|6666666.28487.peg.4086	Protein	24211	25632	+	4-coumarate--CoA ligase 1 (EC 6.2.1.12)
contig_52	fig|6666666.28487.peg.4087	Protein	25617	26432	+	FIG00823074: hypothetical protein
contig_52	fig|6666666.28487.peg.4088	Protein	26419	27507	+	Butyryl-CoA dehydrogenase (EC 1.3.99.2)
contig_52	fig|6666666.28487.peg.4089	Protein	27520	28275	+	Enoyl-CoA hydratase (EC 4.2.1.17)
contig_52	fig|6666666.28487.peg.4090	Protein	29175	28429	-	short-chain dehydrogenase/reductase SDR
contig_52	fig|6666666.28487.peg.4091	Protein	29936	29172	-	3-hydroxyacyl-CoA dehydrogenase

contig_52	fig|6666666.28487.peg.4092	Protein	31473	30862	-	Transcriptional regulator, TetR family
contig_52	fig|6666666.28487.peg.4093	Protein	33140	31527	-	3-oxosteroid 1-dehydrogenase (EC 1.3.99.4)
contig_52	fig|6666666.28487.peg.4094	Protein	33859	33158	-	Transcriptional regulator, IclR family
contig_52	fig|6666666.28487.peg.4095	Protein	34012	34878	+	2-hydroxy-6-oxo-6-phenylhexa-2,4-dienoate hydrolase (EC 3.7.1.-)
contig_52	fig|6666666.28487.peg.4096	Protein	34875	36596	+	3-(3-hydroxy-phenyl)propionate hydroxylase (EC 1.14.13.-)
contig_52	fig|6666666.28487.peg.4097	Protein	36778	37461	+	3-carboxyethylcatechol 2,3-dioxygenase (EC 1.13.11.16)
contig_52	fig|6666666.28487.peg.4098	Protein	37458	39263	+	3-oxosteroid 1-dehydrogenase (EC 1.3.99.4)
contig_52	fig|6666666.28487.peg.4099	Protein	39260	40579	+	F420-dependent N(5),N(10)-methylenetetrahydromethanopterin reductase (EC 1.5.99.11)
contig_52	fig|6666666.28487.peg.4100	Protein	40595	41434	+	3-alpha-hydroxysteroid dehydrogenase
contig_52	fig|6666666.28487.peg.4101	Protein	41431	42429	+	FIG00823018: hypothetical protein
contig_52	fig|6666666.28487.peg.4102	Protein	42426	43028	+	NADPH-dependent FMN reductase family protein
contig_52	fig|6666666.28487.peg.4103	Protein	43033	43863	+	2-hydroxymuconic semialdehyde hydrolase (EC 3.7.1.9)
contig_52	fig|6666666.28487.peg.4104	Protein	43856	44683	+	Short chain dehydrogenase
contig_52	fig|6666666.28487.peg.4105	Protein	44743	45396	+	Transcriptional regulator, TetR family
contig_52	fig|6666666.28487.peg.4106	Protein	45398	45592	+	Ferredoxin
contig_52	fig|6666666.28487.peg.4107	Protein	45696	46805	+	putative cytochrome P450 hydroxylase
contig_52	fig|6666666.28487.peg.4108	Protein	46877	48070	+	BarH
contig_52	fig|6666666.28487.peg.4109	Protein	48073	49320	+	FIG00826794: hypothetical protein

contig_52	fig|6666666.28487.peg.4110	Protein	49320	50462	+	Xaa-Pro dipeptidase (EC 3.4.13.9)
contig_52	fig|6666666.28487.peg.4111	Protein	50455	51414	+	Enoyl-CoA hydratase/isomerase
contig_52	fig|6666666.28487.peg.4112	Protein	51422	53830	+	CAIB-BAIF family family
contig_52	fig|6666666.28487.peg.4113	Protein	53841	54647	+	3-oxoacyl-[acyl-carrier protein] reductase (EC 1.1.1.100)
contig_52	fig|6666666.28487.peg.4114	Protein	55693	54644	-	UDP-glucose 4-epimerase
contig_52	fig|6666666.28487.peg.4115	Protein	55767	56564	+	FIG00824813: hypothetical protein
contig_52	fig|6666666.28487.peg.4116	Protein	56570	58189	+	Long-chain-fatty-acid--CoA ligase (EC 6.2.1.3)
contig_52	fig|6666666.28487.peg.4117	Protein	59630	58191	-	Diacyglycerol O-acyltransferase (EC 2.3.1.20)
contig_52	fig|6666666.28487.peg.4118	Protein	60616	59780	-	FIG00822915: hypothetical protein
contig_52	fig|6666666.28487.peg.4119	Protein	61872	60664	-	putative cytochrome P450 hydroxylase
contig_52	fig|6666666.28487.peg.4120	Protein	62757	62110	-	Transcriptional regulator, TetR family
contig_52	fig|6666666.28487.peg.4121	Protein	62988	63770	+	Enoyl-CoA hydratase (EC 4.2.1.17)
contig_52	fig|6666666.28487.peg.4122	Protein	64742	63930	-	Butyryl-CoA dehydrogenase (EC 1.3.99.2)
contig_52	fig|6666666.28487.peg.4123	Protein	65872	64745	-	Acyl-CoA dehydrogenase (EC 1.3.99.-)
contig_52	fig|6666666.28487.peg.4124	Protein	66297	65869	-	FIG00822989: hypothetical protein
contig_52	fig|6666666.28487.peg.4125	Protein	67525	66281	-	Prolidase (EC 3.4.13.9)
contig_52	fig|6666666.28487.peg.4126	Protein	67857	67528	-	hypothetical protein
contig_52	fig|6666666.28487.peg.4127	Protein	69172	67850	-	Choline monooxygenase, chloroplast precursor (EC 1.14.15.7)
contig_52	fig|6666666.28487.peg.4128	Protein	69720	69178	-	FIG00823110: hypothetical protein

contig_52	fig|6666666.28487.peg.4129	Protein	69985	71280	+	FIG00821608: hypothetical protein
contig_52	fig|6666666.28487.peg.4130	Protein	71372	72559	+	Acyl-CoA dehydrogenase, short-chain specific (EC 1.3.99.2)
contig_52	fig|6666666.28487.peg.4131	Protein	72611	73843	+	CAIB/BAIF family protein
contig_52	fig|6666666.28487.peg.4132	Protein	73836	74630	+	Enoyl-CoA hydratase (EC 4.2.1.17)
contig_52	fig|6666666.28487.peg.4133	Protein	74636	75781	+	3-ketoacyl-CoA thiolase (EC 2.3.1.16) @ Acetyl-CoA acetyltransferase (EC 2.3.1.9)
contig_52	fig|6666666.28487.peg.4134	Protein	75796	76944	+	Acyl-CoA dehydrogenase, short-chain specific (EC 1.3.99.2)
contig_52	fig|6666666.28487.peg.4135	Protein	77834	76947	-	hypothetical protein
contig_52	fig|6666666.28487.peg.4136	Protein	78066	78707	+	Transcriptional regulator, TetR family
contig_52	fig|6666666.28487.peg.4137	Protein	78704	79207	+	FIG00821309: hypothetical protein
contig_52	fig|6666666.28487.peg.4138	Protein	80136	79210	-	Quinone oxidoreductase (EC 1.6.5.5)
contig_52	fig|6666666.28487.peg.4139	Protein	81197	80178	-	probable phosphotransferase
contig_52	fig|6666666.28487.peg.4140	Protein	82492	81194	-	Acyl-CoA dehydrogenase, short-chain specific (EC 1.3.99.2)
contig_52	fig|6666666.28487.peg.4141	Protein	83057	82467	-	Transcriptional regulator, TetR family
contig_52	fig|6666666.28487.peg.4142	Protein	83275	83460	+	hypothetical protein
contig_52	fig|6666666.28487.peg.4143	Protein	83562	84836	+	cytochrome p450 107b1
contig_52	fig|6666666.28487.peg.4144	Protein	84833	85024	+	fdxD-related protein
contig_52	fig|6666666.28487.peg.4145	Protein	85059	85328	+	FIG00820944: hypothetical protein
contig_52	fig|6666666.28487.peg.4146	Protein	86215	85445	-	EstC
contig_52	fig|6666666.28487.peg.4147	Protein	87455	86220	-	putative cytochrome P450 hydroxylase

contig_52	fig|6666666.28487.peg.4148	Protein	87619	87419	-	Probable Ferredoxin FdxD
contig_52	fig|6666666.28487.peg.4149	Protein	88859	87654	-	putative cytochrome P450 hydroxylase
contig_52	fig|6666666.28487.peg.4150	Protein	89772	88981	-	6-phosphogluconate dehydrogenase, NAD-binding
contig_52	fig|6666666.28487.peg.4151	Protein	90587	89769	-	2-hydroxy-3-oxopropionate reductase (EC 1.1.1.60)
contig_52	fig|6666666.28487.peg.4152	Protein	91468	90587	-	FIG00824394: hypothetical protein
contig_52	fig|6666666.28487.peg.4153	Protein	93126	91465	-	3-ketoacyl-CoA thiolase
contig_52	fig|6666666.28487.peg.4154	Protein	93413	93108	-	fdxD-related protein
contig_52	fig|6666666.28487.peg.4155	Protein	94690	93410	-	NADH-quinone oxidoreductase chain F 2 (EC 1.6.99.5); Hypothetical FeS oxidoreductase
contig_52	fig|6666666.28487.peg.4156	Protein	94735	95199	+	FIG00824538: hypothetical protein
contig_52	fig|6666666.28487.peg.4157	Protein	96098	95220	-	FIG00820694: hypothetical protein
contig_52	fig|6666666.28487.peg.4158	Protein	96508	96113	-	Rieske (2Fe-2S) domain-containing protein
contig_52	fig|6666666.28487.peg.4159	Protein	97650	96505	-	Amidohydrolase 2
contig_52	fig|6666666.28487.peg.4160	Protein	98794	97670	-	FIG00821228: hypothetical protein
contig_52	fig|6666666.28487.peg.4161	Protein	99061	100056	+	FIG00821611: hypothetical protein
contig_52	fig|6666666.28487.peg.4162	Protein	100056	101231	+	Acyl-CoA dehydrogenase (EC 1.3.99.-)
contig_52	fig|6666666.28487.peg.4163	Protein	101811	101374	-	FIG00822960: hypothetical protein
contig_52	fig|6666666.28487.peg.4164	Protein	103308	101884	-	putative cytochrome P450 hydroxylase
contig_52	fig|6666666.28487.peg.4165	Protein	103519	103325	-	fdxD-related protein
contig_52	fig|6666666.28487.peg.4166	Protein	104392	103532	-	Oxidoreductase, short-chain dehydrogenase/reductase family

contig_52	fig|6666666.28487.peg.4167	Protein	105127	104426	-	3-oxoacyl-[acyl-carrier protein] reductase (EC 1.1.1.100)
contig_52	fig|6666666.28487.peg.4168	Protein	105412	106908	+	Aldehyde dehydrogenase (EC 1.2.1.3)
contig_52	fig|6666666.28487.peg.4169	Protein	106905	108113	+	L-carnitine dehydratase/bile acid-inducible protein F
contig_52	fig|6666666.28487.peg.4170	Protein	108137	109312	+	Hydroxymethylglutaryl-CoA synthase (EC 2.3.3.10)
contig_52	fig|6666666.28487.peg.4171	Protein	109309	110472	+	Acetyl-CoA acetyltransferase
contig_52	fig|6666666.28487.peg.4172	Protein	110462	111271	+	Conserved hypothetical integral membrane protein YrbE1A
contig_52	fig|6666666.28487.peg.4173	Protein	111279	112136	+	Conserved hypothetical integral membrane protein YrbE1B
contig_52	fig|6666666.28487.peg.4174	Protein	112146	113339	+	MCE-family protein Mce1A
contig_52	fig|6666666.28487.peg.4175	Protein	113336	114361	+	MCE-family protein Mce1B
contig_52	fig|6666666.28487.peg.4176	Protein	114361	115464	+	MCE-family protein Mce1C
contig_52	fig|6666666.28487.peg.4177	Protein	115448	116944	+	MCE-family protein Mce1D
contig_52	fig|6666666.28487.peg.4178	Protein	116941	118281	+	MCE-family lipoprotein LprK (MCE-family lipoprotein Mce1e)
contig_52	fig|6666666.28487.peg.4179	Protein	118281	119972	+	MCE-family protein Mce1F
contig_52	fig|6666666.28487.peg.4180	Protein	120520	119996	-	FIG00827886: hypothetical protein
contig_52	fig|6666666.28487.peg.4181	Protein	120599	121024	+	FIG00824025: hypothetical protein
contig_52	fig|6666666.28487.peg.4182	Protein	121472	121035	-	FIG00821635: hypothetical protein
contig_52	fig|6666666.28487.peg.4183	Protein	122805	121519	-	putative cytochrome P450 hydroxylase
contig_52	fig|6666666.28487.peg.4184	Protein	123002	123631	+	Transcriptional regulator, TetR family
contig_52	fig|6666666.28487.peg.4185	Protein	123926	123639	-	hypothetical protein

contig_52	fig|6666666.28487.peg.4186	Protein	124537	123929	-	Cytochrome c oxidase polypeptide III (EC 1.9.3.1)
contig_52	fig|6666666.28487.peg.4187	Protein	125247	124534	-	hypothetical protein
contig_52	fig|6666666.28487.peg.4188	Protein	126023	125277	-	FIG00827853: hypothetical protein
contig_52	fig|6666666.28487.peg.4189	Protein	127277	126126	-	Nonspecific lipid-transfer protein
contig_52	fig|6666666.28487.peg.4190	Protein	127572	127288	-	FIG00824941: hypothetical protein
contig_52	fig|6666666.28487.peg.4191	Protein	127631	127753	+	hypothetical protein
contig_52	fig|6666666.28487.peg.4192	Protein	128511	127792	-	FIG00660553: hypothetical protein
contig_52	fig|6666666.28487.peg.4193	Protein	128639	130234	+	Long-chain-fatty-acid--CoA ligase (EC 6.2.1.3)
contig_52	fig|6666666.28487.peg.4194	Protein	130270	131460	+	BarH
contig_52	fig|6666666.28487.peg.4195	Protein	131507	133000	+	Long-chain-fatty-acid--CoA ligase (EC 6.2.1.3)
contig_52	fig|6666666.28487.peg.4196	Protein	133153	134031	+	POSSIBLE MEMBRANE PROTEIN
contig_52	fig|6666666.28487.peg.4197	Protein	134028	134687	+	Uncharacterized protein Rv1362c/MT1407
contig_52	fig|6666666.28487.peg.4198	Protein	134714	135379	+	2-C-methyl-D-erythritol 4-phosphate cytidylyltransferase (EC 2.7.7.60)
contig_52	fig|6666666.28487.peg.4199	Protein	136061	135345	-	2-C-methyl-D-erythritol 4-phosphate cytidylyltransferase (EC 2.7.7.60)
contig_52	fig|6666666.28487.peg.4200	Protein	136191	137459	+	putative integral membrane protein
contig_52	fig|6666666.28487.peg.4201	Protein	138699	137446	-	membrane protein, putative
contig_52	fig|6666666.28487.peg.4202	Protein	139052	139792	+	Probable transcription regulator protein
contig_52	fig|6666666.28487.peg.4203	Protein	140675	150007	+	[Acyl-carrier-protein] acetyl transferase of FASI (EC 2.3.1.38) / Enoyl-[acyl-carrier-protein] reductase of FASI (EC 1.3.1.9) /

						3-hydroxypalmitoyl-[acyl-carrier-protein] dehydratase of FASI (EC 4.2.1.61) / [Acyl-carrier-protein] malonyl transferase of FASI (EC 2.3.1.39) / [Acyl-carrier-protein] palmitoyl transferase of FASI (EC 2.3.1.-) / Acyl carrier protein of FASI / 3-oxoacyl-[acyl-carrier-protein] reductase of FASI (EC 1.1.1.100) / 3-oxoacyl-[acyl-carrier-protein] synthase of FASI (EC 2.3.1.41)
contig_52	fig|6666666.28487.peg.4204	Protein	150030	150422	+	Holo-[acyl-carrier protein] synthase (EC 2.7.8.7)
contig_52	fig|6666666.28487.peg.4205	Protein	150619	151455	+	Formate efflux transporter (TC 2.A.44 family)
contig_52	fig|6666666.28487.peg.4206	Protein	151465	152814	+	Catalyzes the cleavage of p-aminobenzoyl-glutamate to p-aminobenzoate and glutamate, subunit A
contig_52	fig|6666666.28487.peg.4207	Protein	153264	152791	-	Thiol peroxidase, Bcp-type (EC 1.11.1.15)
contig_52	fig|6666666.28487.peg.4208	Protein	153430	153657	+	Cell division protein DivIC (FtsB), stabilizes FtsL against RasP cleavage
contig_52	fig|6666666.28487.peg.4209	Protein	153834	153664	-	hypothetical protein
contig_52	fig|6666666.28487.peg.4210	Protein	154208	154585	+	FIG00831553: hypothetical protein
contig_52	fig|6666666.28487.rna.29	RNA	154853	154781	-	tRNA-Lys-CTT
contig_52	fig|6666666.28487.peg.4211	Protein	155456	155301	-	hypothetical protein
contig_52	fig|6666666.28487.peg.4212	Protein	155445	156485	+	hypothetical protein
contig_52	fig|6666666.28487.peg.4213	Protein	158713	161214	+	hypothetical protein
contig_52	fig|6666666.28487.peg.4214	Protein	161929	162219	+	hypothetical protein
contig_52	fig|6666666.28487.peg.4215	Protein	162387	162911	+	hypothetical protein
contig_52	fig|6666666.28487.peg.4216	Protein	163404	163919	+	Resolvase, N-terminal domain

contig_52	fig|6666666.28487.peg.4217	Protein	164399	165808	+	hypothetical protein
contig_52	fig|6666666.28487.peg.4218	Protein	166035	165805	-	hypothetical protein
contig_52	fig|6666666.28487.rna.30	RNA	167904	167832	-	tRNA-Lys-CTT
contig_52	fig|6666666.28487.peg.4219	Protein	168063	169283	+	ErfK/YbiS/YcfS/YnhG family protein
contig_52	fig|6666666.28487.peg.4220	Protein	169414	169659	+	hypothetical protein
contig_52	fig|6666666.28487.rna.31	RNA	169754	169682	-	tRNA-His-GTG
contig_52	fig|6666666.28487.peg.4221	Protein	170420	169785	-	3'-to-5' oligoribonuclease (orn)
contig_52	fig|6666666.28487.peg.4222	Protein	170514	172088	+	ATPase
contig_52	fig|6666666.28487.peg.4223	Protein	172971	172165	-	Short-chain dehydrogenase/reductase SDR
contig_52	fig|6666666.28487.peg.4224	Protein	172997	174316	+	Permease of the major facilitator transporter superfamily
contig_52	fig|6666666.28487.peg.4225	Protein	175074	174313	-	POSSIBLE CONSERVED PROLINE RICH MEMBRANE PROTEIN
contig_52	fig|6666666.28487.peg.4226	Protein	175244	177106	+	Acylamino-acid-releasing enzyme
contig_52	fig|6666666.28487.peg.4227	Protein	177199	177402	+	FIG00823383: hypothetical protein
contig_52	fig|6666666.28487.peg.4228	Protein	177436	177591	+	FIG00825868: hypothetical protein
contig_52	fig|6666666.28487.peg.4229	Protein	178277	177597	-	Transcriptional regulator, TetR family
contig_52	fig|6666666.28487.peg.4230	Protein	178371	179921	+	Methylcrotonyl-CoA carboxylase carboxyl transferase subunit (EC 6.4.1.4)
contig_52	fig|6666666.28487.peg.4231	Protein	179924	181906	+	Methylcrotonyl-CoA carboxylase biotin-containing subunit (EC 6.4.1.4)
contig_52	fig|6666666.28487.peg.4232	Protein	181903	183063	+	Isovaleryl-CoA dehydrogenase (EC 1.3.99.10)

contig_52	fig|6666666.28487.peg.4233	Protein	183087	183563	+	Oxidase regulatory-related protein
contig_52	fig|6666666.28487.peg.4234	Protein	183560	184363	+	Hydroxymethylglutaryl-CoA lyase (EC 4.1.3.4)
contig_52	fig|6666666.28487.peg.4235	Protein	185116	184364	-	Enoyl-CoA hydratase (EC 4.2.1.17)
contig_52	fig|6666666.28487.peg.4236	Protein	185936	185121	-	hypothetical protein
contig_52	fig|6666666.28487.peg.4237	Protein	185993	187108	+	hypothetical protein
contig_52	fig|6666666.28487.peg.4238	Protein	187957	187115	-	Transcriptional regulator, IclR family
contig_52	fig|6666666.28487.peg.4239	Protein	189343	188054	-	Phosphoglycerate mutase
contig_52	fig|6666666.28487.peg.4240	Protein	190243	189479	-	Acyl-ACP thioesterase, FatA
contig_52	fig|6666666.28487.peg.4241	Protein	190849	190250	-	Uncharacterized methyltransferase Rv0089/MT0098 (EC 2.1.1.-)
contig_52	fig|6666666.28487.peg.4242	Protein	191594	190857	-	Dienelactone hydrolase family protein
contig_52	fig|6666666.28487.peg.4243	Protein	191589	191726	+	hypothetical protein
contig_52	fig|6666666.28487.peg.4244	Protein	192133	191723	-	hypothetical protein
contig_52	fig|6666666.28487.peg.4245	Protein	192205	192969	+	FIG00822710: hypothetical protein
contig_52	fig|6666666.28487.peg.4246	Protein	193031	193888	+	FIG00829740: hypothetical protein
contig_52	fig|6666666.28487.peg.4247	Protein	195268	193898	-	Glycosyl transferase, family 2
contig_52	fig|6666666.28487.peg.4248	Protein	196254	195265	-	hypothetical protein
contig_52	fig|6666666.28487.peg.4249	Protein	197042	196251	-	hypothetical protein
contig_52	fig|6666666.28487.peg.4250	Protein	197304	198368	+	hypothetical protein
contig_52	fig|6666666.28487.peg.4251	Protein	198396	199016	+	FIG00824959: hypothetical protein

contig_52	fig|6666666.28487.peg.4252	Protein	199721	199137	-	transcriptional regulator, TetR family
contig_52	fig|6666666.28487.peg.4253	Protein	199884	201095	+	putative cytochrome P450 hydroxylase
contig_52	fig|6666666.28487.peg.4254	Protein	201193	202113	+	probable oxidoreductase/Short-chain dehydrogenase
contig_52	fig|6666666.28487.peg.4255	Protein	202094	202993	+	Esterase LipW
contig_52	fig|6666666.28487.peg.4256	Protein	203340	203140	-	hypothetical protein
contig_52	fig|6666666.28487.rna.32	RNA	203720	203647	-	tRNA-Arg-TCT
contig_52	fig|6666666.28487.peg.4257	Protein	203813	205315	+	Acyltransferase, ws/dgat/mgat subfamily protein
contig_52	fig|6666666.28487.peg.4258	Protein	205312	206871	+	Phosphoserine phosphatase (EC 3.1.3.3) / 1-acyl-sn-glycerol-3-phosphate acyltransferase (EC 2.3.1.51)
contig_52	fig|6666666.28487.peg.4259	Protein	206868	209219	+	Glycerol-3-phosphate acyltransferase (EC 2.3.1.15)
contig_52	fig|6666666.28487.peg.4260	Protein	209251	211260	+	Copper resistance protein D
contig_52	fig|6666666.28487.peg.4261	Protein	211367	211531	+	hypothetical protein
contig_52	fig|6666666.28487.peg.4262	Protein	211564	212025	+	Single-strand DNA binding protein
contig_52	fig|6666666.28487.peg.4263	Protein	212138	213811	+	ABC transporter ATP-binding protein
contig_52	fig|6666666.28487.peg.4264	Protein	213930	218801	+	NAD-specific glutamate dehydrogenase (EC 1.4.1.2), large form
contig_52	fig|6666666.28487.peg.4265	Protein	218798	219217	+	FIG00995656: hypothetical protein
contig_52	fig|6666666.28487.peg.4266	Protein	219214	219897	+	FIG01001213: hypothetical protein
contig_52	fig|6666666.28487.peg.4267	Protein	220373	219963	-	Hemoglobin-like protein HbO
contig_52	fig|6666666.28487.peg.4268	Protein	223004	220425	-	FIG00833626: hypothetical protein

contig_52	fig|6666666.28487.peg.4269	Protein	224211	223075	-	Catalase (EC 1.11.1.6)
contig_52	fig|6666666.28487.peg.4270	Protein	224393	225100	+	HNH endonuclease family protein
contig_52	fig|6666666.28487.peg.4271	Protein	225126	225359	+	FIG00821364: hypothetical protein
contig_52	fig|6666666.28487.peg.4272	Protein	225346	225822	+	FIG00997368: hypothetical protein
contig_52	fig|6666666.28487.peg.4273	Protein	226179	225841	-	FIG00827048: hypothetical protein
contig_52	fig|6666666.28487.peg.4274	Protein	229022	226299	-	Membrane alanine aminopeptidase N (EC 3.4.11.2)
contig_52	fig|6666666.28487.peg.4275	Protein	229118	229738	+	FIG00820885: hypothetical protein
contig_52	fig|6666666.28487.peg.4276	Protein	230378	229740	-	hypothetical protein
contig_52	fig|6666666.28487.peg.4277	Protein	230501	230623	+	hypothetical protein
contig_52	fig|6666666.28487.peg.4278	Protein	230590	231063	+	Cytosine deaminase (EC 3.5.4.1)
contig_52	fig|6666666.28487.peg.4279	Protein	231060	232013	+	Oxidoreductase, 2OG-Fe(II) oxygenase family
contig_52	fig|6666666.28487.peg.4280	Protein	232146	232619	+	Ribose 5-phosphate isomerase B (EC 5.3.1.6) / Galactose 6-phosphate isomerase
contig_53	fig|6666666.28487.peg.4281	Protein	1899	1102	-	Diaminopimelate decarboxylase
contig_53	fig|6666666.28487.peg.4282	Protein	2362	1868	-	hypothetical protein
contig_53	fig|6666666.28487.peg.4283	Protein	2553	3386	+	Hydride transferase 1 (Fragment)
contig_53	fig|6666666.28487.peg.4284	Protein	3470	4567	+	protein of unknown function DUF1023
contig_53	fig|6666666.28487.peg.4285	Protein	4600	5160	+	conserved hypothetical protein
contig_53	fig|6666666.28487.peg.4286	Protein	6659	5157	-	Acetyl-CoA acetyltransferase
contig_53	fig|6666666.28487.peg.4287	Protein	6902	8767	+	peptidase S9, prolyl oligopeptidase active site domain protein

contig_53	fig|6666666.28487.peg.4288	Protein	9836	8934	-	Succinyl-CoA ligase [ADP-forming] alpha chain (EC 6.2.1.5)
contig_53	fig|6666666.28487.peg.4289	Protein	11014	9851	-	Succinyl-CoA ligase [ADP-forming] beta chain (EC 6.2.1.5)
contig_53	fig|6666666.28487.peg.4290	Protein	11090	11233	+	hypothetical protein
contig_53	fig|6666666.28487.peg.4291	Protein	11278	12333	+	Phage peptidoglycan binding endopeptidase
contig_53	fig|6666666.28487.peg.4292	Protein	12334	12948	+	FIG00820415: hypothetical protein
contig_53	fig|6666666.28487.peg.4293	Protein	15289	12950	-	ATP-dependent DNA helicase UvrD/PcrA
contig_53	fig|6666666.28487.peg.4294	Protein	16878	15424	-	PE-PGRS family protein
contig_53	fig|6666666.28487.peg.4295	Protein	17741	17292	-	hypothetical protein
contig_53	fig|6666666.28487.peg.4296	Protein	18883	17864	-	hypothetical protein
contig_53	fig|6666666.28487.peg.4297	Protein	20933	19377	-	PE-PGRS family protein
contig_53	fig|6666666.28487.peg.4298	Protein	21368	21246	-	hypothetical protein
contig_53	fig|6666666.28487.peg.4299	Protein	22122	22418	+	Chorismate mutase I (EC 5.4.99.5)
contig_53	fig|6666666.28487.peg.4300	Protein	24449	22434	-	putative acyltransferase
contig_53	fig|6666666.28487.peg.4301	Protein	26092	24644	-	Succinate-semialdehyde dehydrogenase [NADP+] (EC 1.2.1.16)
contig_53	fig|6666666.28487.peg.4302	Protein	26142	27791	+	Glucose-6-phosphate isomerase (EC 5.3.1.9)
contig_53	fig|6666666.28487.peg.4303	Protein	28370	27840	-	FIG00820902: hypothetical protein
contig_53	fig|6666666.28487.peg.4304	Protein	28638	28363	-	transcriptional regulator, HTH_3 family
contig_53	fig|6666666.28487.peg.4305	Protein	29529	28780	-	Oxidoreductase, short-chain dehydrogenase/reductase family (EC 1.1.1.-)

contig_53	fig|6666666.28487.peg.4306	Protein	30494	29631	-	Formamidopyrimidine-DNA glycosylase (EC 3.2.2.23)
contig_53	fig|6666666.28487.peg.4307	Protein	30883	30500	-	PROBABLE CONSERVED MEMBRANE PROTEIN
contig_53	fig|6666666.28487.peg.4308	Protein	31011	31913	+	FIG00822913: hypothetical protein
contig_53	fig|6666666.28487.peg.4309	Protein	31910	32647	+	hypothetical protein
contig_53	fig|6666666.28487.peg.4310	Protein	32714	33589	+	Hydride transferase 1 (Fragment)
contig_53	fig|6666666.28487.peg.4311	Protein	34146	35261	+	Dimethylallyltransferase (EC 2.5.1.1) / Geranyltranstransferase (farnesyldiphosphate synthase) (EC 2.5.1.10) / Farnesyltranstransferase (EC 2.5.1.29)
contig_53	fig|6666666.28487.peg.4312	Protein	35261	36772	+	Phytoene dehydrogenase (EC 1.14.99.-)
contig_53	fig|6666666.28487.peg.4313	Protein	36784	37728	+	Phytoene synthase (EC 2.5.1.32)
contig_53	fig|6666666.28487.peg.4314	Protein	37761	38075	+	Lycopene cyclase
contig_53	fig|6666666.28487.peg.4315	Protein	38072	38398	+	Lycopene cyclase
contig_53	fig|6666666.28487.peg.4316	Protein	38395	39105	+	CrtT-methyltransferase-like protein
contig_53	fig|6666666.28487.peg.4317	Protein	39102	40634	+	"phi-Carotenoid synthase" (EC 1.3.-.- and EC 2.1.1-)
contig_53	fig|6666666.28487.peg.4318	Protein	40631	41686	+	CrtV-methyltransferase-like protein
contig_53	fig|6666666.28487.peg.4319	Protein	42875	41694	-	FIG00832406: hypothetical protein
contig_53	fig|6666666.28487.peg.4320	Protein	42983	43624	+	4-hydroxy-2-oxoglutarate aldolase (EC 4.1.3.16) @ 2-dehydro-3-deoxyphosphogluconate aldolase (EC 4.1.2.14)
contig_53	fig|6666666.28487.peg.4321	Protein	43621	44853	+	MFS permease
contig_53	fig|6666666.28487.peg.4322	Protein	44882	45172	+	hypothetical protein
contig_53	fig|6666666.28487.peg.4323	Protein	45245	45727	+	hypothetical protein

contig_53	fig|6666666.28487.peg.4324	Protein	45724	46362	+	FIG00826659: hypothetical protein
contig_53	fig|6666666.28487.peg.4325	Protein	46359	46835	+	Ribonuclease HI (EC 3.1.26.4)
contig_53	fig|6666666.28487.peg.4326	Protein	46878	47225	+	Transcriptional regulator, ArsR family
contig_53	fig|6666666.28487.peg.4327	Protein	47240	47839	+	Aha1 domain protein
contig_54	fig|6666666.28487.peg.4328	Protein	202	1779	+	Glycerol-3-phosphate dehydrogenase (EC 1.1.5.3)
contig_54	fig|6666666.28487.peg.4329	Protein	1827	2636	+	Ribosomal large subunit pseudouridine synthase A (EC 4.2.1.70)
contig_54	fig|6666666.28487.peg.4330	Protein	3229	2981	-	hypothetical protein
contig_54	fig|6666666.28487.peg.4331	Protein	3213	3458	+	Extensin protein-like
contig_54	fig|6666666.28487.peg.4332	Protein	3449	4228	+	3-oxoacyl-(acyl-carrier-protein) reductase (fabG)
contig_54	fig|6666666.28487.peg.4333	Protein	4333	5487	+	FIG00822682: hypothetical protein
contig_54	fig|6666666.28487.peg.4334	Protein	6342	5563	-	Enoyl-CoA hydratase (EC 4.2.1.17)
contig_54	fig|6666666.28487.peg.4335	Protein	7035	6352	-	Transcriptional regulator, TetR family
contig_54	fig|6666666.28487.peg.4336	Protein	7105	8163	+	Flavodoxin reductases (ferredoxin-NADPH reductases) family 1
contig_54	fig|6666666.28487.peg.4337	Protein	8256	9380	+	POSSIBLE LINOLEOYL-CoA DESATURASE (DELTA(6)-DESATURASE)
contig_54	fig|6666666.28487.peg.4338	Protein	9549	9974	+	FIG00821802: hypothetical protein
contig_54	fig|6666666.28487.peg.4339	Protein	10932	10006	-	FIG00830679: hypothetical protein
contig_54	fig|6666666.28487.peg.4340	Protein	11790	11038	-	Formamidopyrimidine-DNA glycosylase (EC 3.2.2.23)
contig_54	fig|6666666.28487.peg.4341	Protein	16334	11790	-	Probable ATP-dependent helicase lhr (EC 3.6.1.-)

contig_54	fig|6666666.28487.peg.4342	Protein	16476	18632	+	FIG00823687: hypothetical protein
contig_54	fig|6666666.28487.peg.4343	Protein	19675	18662	-	FIG00831225: hypothetical protein
contig_54	fig|6666666.28487.peg.4344	Protein	20994	19672	-	Peroxidase (EC 1.11.1.7)
contig_54	fig|6666666.28487.peg.4345	Protein	21545	20991	-	hypothetical protein
contig_54	fig|6666666.28487.peg.4346	Protein	21872	22267	+	hypothetical protein
contig_54	fig|6666666.28487.peg.4347	Protein	22291	22617	+	seq ID no 1F, putative
contig_54	fig|6666666.28487.peg.4348	Protein	22637	22978	+	hypothetical protein
contig_54	fig|6666666.28487.peg.4349	Protein	23628	22981	-	Transcriptional regulator, TetR family
contig_54	fig|6666666.28487.peg.4350	Protein	23650	23820	+	hypothetical protein
contig_54	fig|6666666.28487.peg.4351	Protein	24605	23796	-	Oxidoreductase, short chain dehydrogenase/reductase family
contig_54	fig|6666666.28487.peg.4352	Protein	26266	24725	-	Aldehyde dehydrogenase B (EC 1.2.1.22)
contig_54	fig|6666666.28487.peg.4353	Protein	27489	26263	-	Uncharacterized protein Rv3292/MT3391
contig_54	fig|6666666.28487.peg.4354	Protein	27517	27996	+	Leucine-responsive regulatory protein, regulator for leucine (or lrp) regulon and high-affinity branched-chain amino acid transport system
contig_54	fig|6666666.28487.peg.4355	Protein	28115	29395	+	Probable L-lysine-epsilon aminotransferase (EC 2.6.1.36) (L-lysine aminotransferase) (Lysine 6-aminotransferase)
contig_54	fig|6666666.28487.peg.4356	Protein	29936	29373	-	FIG00829316: hypothetical protein
contig_54	fig|6666666.28487.peg.4357	Protein	29984	30412	+	hypothetical protein
contig_54	fig|6666666.28487.peg.4358	Protein	30446	30754	+	Putative uncharacterized protein usfY
contig_54	fig|6666666.28487.peg.4359	Protein	30814	31533	+	FIG00831883: hypothetical protein

contig_54	fig|6666666.28487.peg.4360	Protein	32107	31871	-	hypothetical protein
contig_54	fig|6666666.28487.peg.4361	Protein	32496	32368	-	FIG00821791: hypothetical protein
contig_54	fig|6666666.28487.peg.4362	Protein	32618	33052	+	Serine-protein kinase RsbW (EC 2.7.11.1)
contig_54	fig|6666666.28487.peg.4363	Protein	33115	33840	+	RNA polymerase sigma factor SigB
contig_54	fig|6666666.28487.peg.4364	Protein	34248	33820	-	Anti-sigma F factor antagonist (spoIIAA-2); Anti-sigma B factor antagonist RsbV
contig_54	fig|6666666.28487.peg.4365	Protein	34526	35044	+	CTP:molybdopterin cytidylyltransferase
contig_54	fig|6666666.28487.peg.4366	Protein	35358	35041	-	hypothetical protein
contig_54	fig|6666666.28487.peg.4367	Protein	37567	35765	-	Biotin carboxylase of acetyl-CoA carboxylase (EC 6.3.4.14) / Biotin carboxyl carrier protein of acetyl-CoA carboxylase
contig_54	fig|6666666.28487.peg.4368	Protein	38780	37683	-	diguanylate cyclase
contig_54	fig|6666666.28487.peg.4369	Protein	39188	38883	-	FIG00830350: hypothetical protein
contig_54	fig|6666666.28487.peg.4370	Protein	39667	39263	-	Sulfur acceptor protein SufE for iron-sulfur cluster assembly
contig_54	fig|6666666.28487.peg.4371	Protein	40566	39670	-	Thiosulfate sulfurtransferase, rhodanese (EC 2.8.1.1)
contig_54	fig|6666666.28487.peg.4372	Protein	41802	40627	-	acyltransferase family protein
contig_54	fig|6666666.28487.peg.4373	Protein	42205	42570	+	FIG00823944: hypothetical protein
contig_54	fig|6666666.28487.peg.4374	Protein	42653	43009	+	FIG00824435: hypothetical protein
contig_54	fig|6666666.28487.peg.4375	Protein	43644	43006	-	Septum formation protein Maf
contig_54	fig|6666666.28487.peg.4376	Protein	43922	43641	-	FIG00820143: hypothetical protein
contig_54	fig|6666666.28487.peg.4377	Protein	45591	43951	-	Methylcrotonyl-CoA carboxylase carboxyl transferase subunit (EC 6.4.1.4)

contig_54	fig|6666666.28487.peg.4378	Protein	46061	46489	+	hypothetical protein
contig_54	fig|6666666.28487.peg.4379	Protein	46578	48416	+	Methyl-accepting chemotaxis protein
contig_54	fig|6666666.28487.peg.4380	Protein	48379	49962	+	Tyrosine-protein kinase Wzc (EC 2.7.10.2)
contig_54	fig|6666666.28487.peg.4381	Protein	53847	54071	+	hypothetical protein
contig_54	fig|6666666.28487.peg.4382	Protein	55282	56865	+	hypothetical protein
contig_54	fig|6666666.28487.peg.4383	Protein	60184	59429	-	glycosyl transferase, group 1
contig_54	fig|6666666.28487.peg.4384	Protein	61192	60824	-	GDP-L-fucose synthetase (EC 1.1.1.271)
contig_54	fig|6666666.28487.peg.4385	Protein	62136	61795	-	GDP-mannose 4,6 dehydratase (EC 4.2.1.47)
contig_54	fig|6666666.28487.peg.4386	Protein	63578	65896	+	dTDP-glucose 4,6-dehydratase (EC 4.2.1.46)
contig_54	fig|6666666.28487.peg.4387	Protein	65927	67414	+	Undecaprenyl-phosphate galactosephosphotransferase (EC 2.7.8.6)
contig_54	fig|6666666.28487.peg.4388	Protein	67800	67408	-	protein tyrosine phosphatase
contig_54	fig|6666666.28487.peg.4389	Protein	68300	68947	+	Undecaprenyl diphosphate synthase (EC 2.5.1.31)
contig_54	fig|6666666.28487.peg.4390	Protein	68955	70346	+	Gll1135 protein
contig_54	fig|6666666.28487.peg.4391	Protein	70491	70970	+	FIG00995613: hypothetical protein
contig_54	fig|6666666.28487.peg.4392	Protein	71009	71578	+	hypothetical protein
contig_54	fig|6666666.28487.peg.4393	Protein	71970	71575	-	hypothetical protein
contig_54	fig|6666666.28487.peg.4394	Protein	72295	71954	-	integral membrane protein possibly involved in chromosome condensation
contig_54	fig|6666666.28487.peg.4395	Protein	73051	72365	-	FIG00821069: hypothetical protein
contig_54	fig|6666666.28487.peg.4396	Protein	73182	74426	+	Phosphoribosylaminoimidazole carboxylase ATPase subunit

						(EC 4.1.1.21)
contig_54	fig|6666666.28487.peg.4397	Protein	74423	74923	+	Phosphoribosylaminoimidazole carboxylase catalytic subunit (EC 4.1.1.21)
contig_54	fig|6666666.28487.peg.4398	Protein	75032	75733	+	putative GAF sensor protein
contig_54	fig|6666666.28487.peg.4399	Protein	75808	76077	+	hypothetical protein
contig_54	fig|6666666.28487.peg.4400	Protein	76577	76386	-	hypothetical protein
contig_54	fig|6666666.28487.peg.4401	Protein	76709	77878	+	Butyryl-CoA dehydrogenase (EC 1.3.99.2)
contig_54	fig|6666666.28487.peg.4402	Protein	78007	78363	+	hypothetical protein
contig_54	fig|6666666.28487.peg.4403	Protein	79151	78360	-	Biotin-protein ligase (EC 6.3.4.15)
contig_54	fig|6666666.28487.peg.4404	Protein	79280	79167	-	hypothetical protein
contig_54	fig|6666666.28487.peg.4405	Protein	79874	79743	-	hypothetical protein
contig_54	fig|6666666.28487.peg.4406	Protein	80909	80175	-	FIG00996117: hypothetical protein
contig_54	fig|6666666.28487.peg.4407	Protein	82335	80887	-	Cell envelope-associated transcriptional attenuator LytR-CpsA-Psr, subfamily A1 (as in PMID19099556)
contig_54	fig|6666666.28487.peg.4408	Protein	82522	83295	+	dTDP-4-dehydrorhamnose reductase (EC 1.1.1.133)
contig_54	fig|6666666.28487.peg.4409	Protein	83327	84205	+	dTDP-Rha:A-D-GlcNAc-diphosphoryl polyprenol, A-3-L-rhamnosyl transferase WbbL
contig_54	fig|6666666.28487.peg.4410	Protein	84205	85284	+	D-glycero-D-manno-heptose 1-phosphate guanosyltransferase
contig_54	fig|6666666.28487.peg.4411	Protein	85974	85333	-	hypothetical protein
contig_54	fig|6666666.28487.peg.4412	Protein	86851	86288	-	NTP pyrophosphohydrolases including oxidative damage

						repair enzymes
contig_54	fig|6666666.28487.peg.4413	Protein	88188	86848	-	Coenzyme F420-0:L-glutamate ligase @ Coenzyme F420-1:L-glutamate ligase / domain of unknown function
contig_54	fig|6666666.28487.peg.4414	Protein	89183	88185	-	Lactyl (2) diphospho-(5')guanosine:7,8-didemethyl-8-hydroxy-5-deazariboflavin 2-phospho-L-lactate transferase
contig_54	fig|6666666.28487.peg.4415	Protein	89475	89885	+	Sporulation regulatory protein WhiB
contig_54	fig|6666666.28487.peg.4416	Protein	90325	89903	-	FIG00822333: hypothetical protein
contig_54	fig|6666666.28487.peg.4417	Protein	90457	90885	+	FIG00820038: hypothetical protein
contig_54	fig|6666666.28487.peg.4418	Protein	91033	92418	+	Phosphomannomutase (EC 5.4.2.8)
contig_54	fig|6666666.28487.peg.4419	Protein	92415	93539	+	Putative regulator of the mannose operon, ManO
contig_54	fig|6666666.28487.peg.4420	Protein	93545	94771	+	Mannose-6-phosphate isomerase (EC 5.3.1.8)
contig_54	fig|6666666.28487.peg.4421	Protein	96071	94737	-	putative secreted protein
contig_54	fig|6666666.28487.peg.4422	Protein	96129	97610	+	Amino acid permease
contig_54	fig|6666666.28487.peg.4423	Protein	97986	98732	+	Alkane-1 monooxygenase (EC 1.14.15.3)
contig_54	fig|6666666.28487.peg.4424	Protein	98736	99206	+	Alkane-1 monooxygenase (EC 1.14.15.3)
contig_54	fig|6666666.28487.peg.4425	Protein	99203	99376	+	Rubredoxin
contig_54	fig|6666666.28487.peg.4426	Protein	99373	99555	+	Rubredoxin
contig_54	fig|6666666.28487.peg.4427	Protein	99678	100298	+	Transcriptional regulator, TetR family
contig_54	fig|6666666.28487.peg.4428	Protein	101532	100282	-	MFS transporter
contig_54	fig|6666666.28487.peg.4429	Protein	101738	103276	+	Adenosylhomocysteinase (EC 3.3.1.1)

contig_54	fig|6666666.28487.peg.4430	Protein	103296	103760	+	FIG00822233: hypothetical protein
contig_54	fig|6666666.28487.peg.4431	Protein	106189	103823	-	PE-PGRS FAMILY PROTEIN, PROBABLY TRIACYLGLYCEROL LIPASE (ESTERASE/LIPASE) (TRIGLYCERIDE LIPASE) (TRIBUTYRASE) (EC 3.1.1.3)
contig_54	fig|6666666.28487.peg.4432	Protein	106326	106958	+	Thymidylate kinase (EC 2.7.4.9)
contig_54	fig|6666666.28487.peg.4433	Protein	107054	107701	+	FIG00830068: hypothetical protein
contig_54	fig|6666666.28487.peg.4434	Protein	107789	108475	+	DNA-binding response regulator mtrA
contig_54	fig|6666666.28487.peg.4435	Protein	108510	110156	+	Sensor histidine kinase MtrB (EC 2.7.3.-)
contig_54	fig|6666666.28487.peg.4436	Protein	110153	111910	+	LpqB
contig_54	fig|6666666.28487.peg.4437	Protein	111963	112400	+	Rieske (2Fe-2S) domain protein
contig_54	fig|6666666.28487.peg.4438	Protein	113098	112859	-	hypothetical protein
contig_54	fig|6666666.28487.peg.4439	Protein	113147	113425	+	FIG00821502: hypothetical protein
contig_54	fig|6666666.28487.peg.4440	Protein	113429	114052	+	Competence protein F homolog, phosphoribosyltransferase domain; protein YhgH required for utilization of DNA as sole source of carbon and energy
contig_54	fig|6666666.28487.peg.4441	Protein	114375	115025	+	Ribosomal subunit interface protein
contig_54	fig|6666666.28487.peg.4442	Protein	115148	117988	+	Protein export cytoplasm protein SecA ATPase RNA helicase (TC 3.A.5.1.1)
contig_54	fig|6666666.28487.peg.4443	Protein	118457	117990	-	FIG00820824: hypothetical protein
contig_54	fig|6666666.28487.peg.4444	Protein	118655	120064	+	Wax ester synthase/acyl-CoA:diacylglycerol acyltransferase
contig_54	fig|6666666.28487.peg.4445	Protein	120123	121844	+	Glycine betaine transporter OpuD

contig_54	fig|6666666.28487.peg.4446	Protein	121845	122672	+	UDP-galactose-lipid carrier transferase (EC 2.-.-.-)
contig_54	fig|6666666.28487.peg.4447	Protein	123723	122713	-	2,3-dihydroxybiphenyl 1,2-dioxygenase
contig_54	fig|6666666.28487.peg.4448	Protein	124571	123723	-	2-hydroxy-6-oxo-6-phenylhexa-2,4-dienoate hydrolase (EC 3.7.1.-)
contig_54	fig|6666666.28487.peg.4449	Protein	126142	124568	-	3-(3-hydroxy-phenyl)propionate hydroxylase (EC 1.14.13.-)
contig_54	fig|6666666.28487.peg.4450	Protein	126230	126877	+	Transcriptional regulator, TetR family
contig_54	fig|6666666.28487.peg.4451	Protein	126874	127662	+	4-oxalocrotonate decarboxylase(EC:4.1.1.77)
contig_54	fig|6666666.28487.peg.4452	Protein	127881	128378	+	FIG00996471: hypothetical protein
contig_54	fig|6666666.28487.peg.4453	Protein	128477	129619	+	Flavodoxin reductases (ferredoxin-NADPH reductases) family 1
contig_54	fig|6666666.28487.peg.4454	Protein	129690	130946	+	POSSIBLE LINOLEOYL-CoA DESATURASE (DELTA(6)-DESATURASE)
contig_54	fig|6666666.28487.peg.4455	Protein	131865	131005	-	oxidoreductase of aldo/keto reductase family, subgroup 1
contig_54	fig|6666666.28487.peg.4456	Protein	132943	131963	-	Ribosome small subunit-stimulated GTPase EngC
contig_54	fig|6666666.28487.peg.4457	Protein	134190	132940	-	5-Enolpyruvylshikimate-3-phosphate synthase (EC 2.5.1.19)
contig_54	fig|6666666.28487.peg.4458	Protein	134308	135054	+	FIG00996837: hypothetical protein
contig_54	fig|6666666.28487.peg.4459	Protein	135051	136559	+	probable transferase
contig_54	fig|6666666.28487.peg.4460	Protein	137332	136547	-	methyltransferase, putative
contig_54	fig|6666666.28487.peg.4461	Protein	137358	138092	+	hypothetical protein
contig_54	fig|6666666.28487.peg.4462	Protein	138103	139671	+	Aldehyde dehydrogenase (EC 1.2.1.3)
contig_54	fig|6666666.28487.peg.4463	Protein	140566	139742	-	Short-chain dehydrogenase/reductase SDR

contig_54	fig|6666666.28487.peg.4464	Protein	141088	140597	-	Cys-tRNA(Pro) deacylase YbaK
contig_54	fig|6666666.28487.peg.4465	Protein	141231	141980	+	RNA polymerase sigma-54 factor RpoN
contig_54	fig|6666666.28487.peg.4466	Protein	141977	142288	+	Anti-sigma factor
contig_54	fig|6666666.28487.peg.4467	Protein	142623	142844	+	Biotin carboxyl carrier protein
contig_54	fig|6666666.28487.peg.4468	Protein	142883	144373	+	Signal transduction histidine kinase, subgroup 2
contig_54	fig|6666666.28487.peg.4469	Protein	144736	144482	-	WhiB-like transcription regulator
contig_54	fig|6666666.28487.peg.4470	Protein	145969	145013	-	Transcription regulator [contains diacylglycerol kinase catalytic domain]
contig_54	fig|6666666.28487.peg.4471	Protein	146037	146480	+	3-dehydroquinate dehydratase II (EC 4.2.1.10)
contig_54	fig|6666666.28487.peg.4472	Protein	146593	146435	-	hypothetical protein
contig_54	fig|6666666.28487.peg.4473	Protein	147372	146848	-	Diamine acetyltransferase (EC 2.3.1.57)
contig_54	fig|6666666.28487.peg.4474	Protein	148466	147369	-	Isochorismate synthase (EC 5.4.4.2)
contig_54	fig|6666666.28487.peg.4475	Protein	149074	148463	-	putative phosphoglycerate mutase family protein
contig_54	fig|6666666.28487.peg.4476	Protein	149168	149971	+	Chromosome (plasmid) partitioning protein ParA / Sporulation initiation inhibitor protein Soj
contig_54	fig|6666666.28487.peg.4477	Protein	151190	149979	-	Cell division protein DivIC (FtsB), stabilizes FtsL against RasP cleavage
contig_54	fig|6666666.28487.peg.4478	Protein	152699	151200	-	putative ATP-dependent RNA helicase
contig_54	fig|6666666.28487.peg.4479	Protein	153029	153670	+	FIG00821439: hypothetical protein
contig_54	fig|6666666.28487.peg.4480	Protein	154554	153739	-	putative membrane protein
contig_54	fig|6666666.28487.peg.4481	Protein	154839	155096	+	putative ATP-binding protein

contig_54	fig|6666666.28487.peg.4482	Protein	155846	155172	-	Transcriptional regulator, TetR family
contig_54	fig|6666666.28487.peg.4483	Protein	156024	155908	-	hypothetical protein
contig_54	fig|6666666.28487.peg.4484	Protein	156076	157065	+	FIG00820929: hypothetical protein
contig_54	fig|6666666.28487.peg.4485	Protein	157238	158416	+	Sulfur carrier protein adenylyltransferase ThiF
contig_54	fig|6666666.28487.peg.4486	Protein	158453	159307	+	FIG00994019: hypothetical protein
contig_54	fig|6666666.28487.peg.4487	Protein	159674	159378	-	Methylated DNA-protein cysteine methyltransferase
contig_54	fig|6666666.28487.peg.4488	Protein	160459	159677	-	possible lipase
contig_54	fig|6666666.28487.peg.4489	Protein	160538	163651	+	ATP-dependent DNA helicase SCO5183
contig_54	fig|6666666.28487.peg.4490	Protein	163726	166920	+	ATP-dependent DNA helicase SCO5184
contig_54	fig|6666666.28487.peg.4491	Protein	167312	166917	-	FIG00827160: hypothetical protein
contig_54	fig|6666666.28487.peg.4492	Protein	167386	168438	+	Potassium channel protein
contig_54	fig|6666666.28487.peg.4493	Protein	168467	169393	+	NADH pyrophosphatase (EC 3.6.1.22)
contig_54	fig|6666666.28487.peg.4494	Protein	170178	169390	-	Methionine aminopeptidase (EC 3.4.11.18)
contig_54	fig|6666666.28487.peg.4495	Protein	170265	170531	+	helix-turn-helix domain protein
contig_54	fig|6666666.28487.peg.4496	Protein	170779	170528	-	POSSIBLE GLUTAREDOXIN PROTEIN
contig_54	fig|6666666.28487.peg.4497	Protein	170853	172970	+	ATP-dependent DNA helicase UvrD/PcrA, actinomycete paralog
contig_54	fig|6666666.28487.peg.4498	Protein	173423	173686	+	WhiB-family transcriptional regulator
contig_54	fig|6666666.28487.peg.4499	Protein	175038	173716	-	Ubiquinone biosynthesis monooxygenase UbiB
contig_54	fig|6666666.28487.peg.4500	Protein	175929	175096	-	FIG00822196: hypothetical protein

contig_54	fig|6666666.28487.peg.4501	Protein	177431	176061	-	Collagen alpha 1(I) chain precursor
contig_54	fig|6666666.28487.peg.4502	Protein	177545	178567	+	Lon-like protease with PDZ domain
contig_54	fig|6666666.28487.peg.4503	Protein	178656	181676	+	INTEGRAL MEMBRANE PROTEIN (Rhomboid family)
contig_54	fig|6666666.28487.peg.4504	Protein	181689	185120	+	transcriptional regulator, AfsR/DnrI/RedD family
contig_54	fig|6666666.28487.peg.4505	Protein	185339	185956	+	hypothetical protein Rv2558
contig_54	fig|6666666.28487.peg.4506	Protein	186101	187489	+	MmcM
contig_54	fig|6666666.28487.peg.4507	Protein	187571	189526	+	Chaperone protein HtpG
contig_54	fig|6666666.28487.rna.33	RNA	189662	189735	+	tRNA-Met-CAT
contig_54	fig|6666666.28487.peg.4508	Protein	190324	189893	-	hypothetical protein
contig_54	fig|6666666.28487.peg.4509	Protein	191115	190921	-	hypothetical protein
contig_54	fig|6666666.28487.peg.4510	Protein	191601	194390	+	FIG00820985: hypothetical protein
contig_54	fig|6666666.28487.peg.4511	Protein	194657	195070	+	Transcriptional regulator, TetR family
contig_54	fig|6666666.28487.peg.4512	Protein	195067	195288	+	hypothetical protein
contig_54	fig|6666666.28487.peg.4513	Protein	196329	195457	-	Glyceraldehyde-3-phosphate ketol-isomerase (EC 5.3.1.1)
contig_54	fig|6666666.28487.peg.4514	Protein	197366	196332	-	Myo-inositol 2-dehydrogenase (EC 1.1.1.18)
contig_54	fig|6666666.28487.peg.4515	Protein	198280	197375	-	Inosose dehydratase (EC 4.2.1.44)
contig_54	fig|6666666.28487.peg.4516	Protein	199689	198277	-	Major myo-inositol transporter IolT
contig_54	fig|6666666.28487.peg.4517	Protein	199849	200841	+	Predicted transcriptional regulator of the myo-inositol catabolic operon
contig_54	fig|6666666.28487.peg.4518	Protein	201069	201374	+	putative integral membrane protein (DMT superfamily)

contig_54	fig|6666666.28487.peg.4519	Protein	201371	201727	+	hypothetical protein
contig_54	fig|6666666.28487.peg.4520	Protein	201724	202623	+	Dihydrodipicolinate synthase (EC 4.2.1.52)
contig_54	fig|6666666.28487.peg.4521	Protein	203559	202981	-	Transcriptional regulator, TetR family
contig_54	fig|6666666.28487.peg.4522	Protein	203660	204490	+	oxidoreductase, short chain dehydrogenase-reductase family
contig_54	fig|6666666.28487.peg.4523	Protein	204487	205290	+	3-oxoacyl-[acyl-carrier protein] reductase (EC 1.1.1.100)
contig_54	fig|6666666.28487.peg.4524	Protein	205287	206090	+	4-carboxymuconolactone decarboxylase (EC 4.1.1.44)
contig_54	fig|6666666.28487.peg.4525	Protein	206087	207547	+	Aldehyde dehydrogenase (EC 1.2.1.3)
contig_54	fig|6666666.28487.peg.4526	Protein	208144	207905	-	MCE-family protein Mce1A
contig_54	fig|6666666.28487.peg.4527	Protein	209202	208363	-	FIG00821662: hypothetical protein
contig_54	fig|6666666.28487.peg.4528	Protein	209988	209332	-	Transcriptional regulator, TetR family
contig_54	fig|6666666.28487.peg.4529	Protein	210072	210272	+	hypothetical protein
contig_54	fig|6666666.28487.peg.4530	Protein	210321	211607	+	putative cytochrome P450 hydroxylase
contig_54	fig|6666666.28487.peg.4531	Protein	212319	211651	-	Transcriptional regulator, TetR family
contig_54	fig|6666666.28487.peg.4532	Protein	212492	215215	+	hypothetical protein
contig_54	fig|6666666.28487.peg.4533	Protein	216164	216277	+	hypothetical protein
contig_54	fig|6666666.28487.peg.4534	Protein	217492	217659	+	hypothetical protein
contig_54	fig|6666666.28487.peg.4535	Protein	218013	218621	+	Chorismate--pyruvate lyase (EC 4.1.3.40)
contig_54	fig|6666666.28487.peg.4536	Protein	218764	220845	+	Long-chain-fatty-acid--CoA ligase FadD22 (EC 6.2.1.3)
contig_54	fig|6666666.28487.peg.4537	Protein	221605	221447	-	Mucin 2 precursor

contig_54	fig|6666666.28487.peg.4538	Protein	226259	224571	-	hypothetical protein
contig_54	fig|6666666.28487.peg.4539	Protein	226799	226632	-	hypothetical protein
contig_54	fig|6666666.28487.peg.4540	Protein	232342	232211	-	hypothetical protein
contig_55	fig|6666666.28487.peg.4541	Protein	1283	72	-	Dihydrolipoamide acetyltransferase component of pyruvate dehydrogenase complex (EC 2.3.1.12)
contig_55	fig|6666666.28487.peg.4542	Protein	3469	1283	-	Branched-chain alpha-keto acid dehydrogenase, E1 component, alpha subunit (EC 1.2.4.4) / Branched-chain alpha-keto acid dehydrogenase, E1 component, beta subunit (EC 1.2.4.4)
contig_55	fig|6666666.28487.peg.4543	Protein	3622	4146	+	conserved hypothetical protein
contig_55	fig|6666666.28487.peg.4544	Protein	4154	4813	+	Transcriptional regulator, GntR family
contig_55	fig|6666666.28487.peg.4545	Protein	4840	6216	+	Succinate-semialdehyde dehydrogenase [NAD] (EC 1.2.1.24); Succinate-semialdehyde dehydrogenase [NADP+] (EC 1.2.1.16)
contig_55	fig|6666666.28487.peg.4546	Protein	7539	6301	-	Aminopeptidase YpdF (MP-, MA-, MS-, AP-, NP- specific)
contig_55	fig|6666666.28487.peg.4547	Protein	8477	7536	-	hypothetical protein
contig_55	fig|6666666.28487.peg.4548	Protein	9311	8505	-	Branched-chain amino acid transport ATP-binding protein LivF (TC 3.A.1.4.1)
contig_55	fig|6666666.28487.peg.4549	Protein	11164	9308	-	Branched-chain amino acid transport ATP-binding protein LivG (TC 3.A.1.4.1)
contig_55	fig|6666666.28487.peg.4550	Protein	12042	11161	-	High-affinity branched-chain amino acid transport system permease protein LivH (TC 3.A.1.4.1)
contig_55	fig|6666666.28487.peg.4551	Protein	13283	12087	-	Extracellular ligand-binding receptor

contig_55	fig|6666666.28487.peg.4552	Protein	13610	14737	+	major facilitator superfamily MFS_1
contig_55	fig|6666666.28487.peg.4553	Protein	15634	14741	-	hypothetical protein
contig_55	fig|6666666.28487.peg.4554	Protein	15722	16897	+	putative aminotransferase
contig_55	fig|6666666.28487.peg.4555	Protein	17448	17008	-	FIG00822577: hypothetical protein
contig_55	fig|6666666.28487.peg.4556	Protein	18584	17508	-	Alpha-methylacyl-CoA racemase (EC 5.1.99.4)
contig_55	fig|6666666.28487.peg.4557	Protein	19066	18581	-	FIG00823411: hypothetical protein
contig_55	fig|6666666.28487.peg.4558	Protein	19138	19443	+	Isopeptidase T
contig_55	fig|6666666.28487.peg.4559	Protein	19477	19629	+	hypothetical protein
contig_55	fig|6666666.28487.peg.4560	Protein	21242	19680	-	Beta-carotene ketolase (EC 1.14.-.-)
contig_55	fig|6666666.28487.peg.4561	Protein	21968	21363	-	Beta-carotene ketolase (EC 1.14.-.-)
contig_55	fig|6666666.28487.peg.4562	Protein	22151	22879	+	FIG00831195: hypothetical protein
contig_55	fig|6666666.28487.peg.4563	Protein	23460	22876	-	Transcriptional regulator, TetR family
contig_55	fig|6666666.28487.peg.4564	Protein	24029	23472	-	Periplasmic chorismate mutase I precursor (EC 5.4.99.5)
contig_55	fig|6666666.28487.rna.34	RNA	24131	24058	-	tRNA-Phe-GAA
contig_55	fig|6666666.28487.rna.35	RNA	24248	24175	-	tRNA-Asp-GTC
contig_55	fig|6666666.28487.rna.36	RNA	24360	24288	-	tRNA-Glu-TTC
contig_55	fig|6666666.28487.rna.37	RNA	24487	24559	+	tRNA-Lys-TTT
contig_55	fig|6666666.28487.peg.4565	Protein	25308	24574	-	hypothetical protein
contig_55	fig|6666666.28487.peg.4566	Protein	25307	25420	+	hypothetical protein

contig_55	fig|6666666.28487.peg.4567	Protein	26091	25411	-	POSSIBLE CONSERVED EXPORTED PROTEIN
contig_55	fig|6666666.28487.peg.4568	Protein	27121	26225	-	serine/threonine protein kinase
contig_55	fig|6666666.28487.peg.4569	Protein	27268	27624	+	hypothetical protein
contig_55	fig|6666666.28487.peg.4570	Protein	28275	27628	-	hypothetical protein
contig_55	fig|6666666.28487.peg.4571	Protein	29286	28297	-	FIG00820092: hypothetical protein
contig_55	fig|6666666.28487.peg.4572	Protein	29388	29969	+	Transcriptional regulator, TetR family
contig_55	fig|6666666.28487.peg.4573	Protein	30076	31080	+	Fatty acid desaturase occurring in virulence cluster
contig_55	fig|6666666.28487.peg.4574	Protein	31110	32243	+	tRNA dihydrouridine synthase B (EC 1.-.-.-)
contig_55	fig|6666666.28487.peg.4575	Protein	32507	34495	+	Cell envelope-associated transcriptional attenuator LytR-CpsA-Psr, subfamily A1 (as in PMID19099556)
contig_55	fig|6666666.28487.peg.4576	Protein	34584	35252	+	Phosphate transport system regulatory protein PhoU
contig_55	fig|6666666.28487.peg.4577	Protein	36119	35343	-	Phosphate transport ATP-binding protein PstB (TC 3.A.1.7.1)
contig_55	fig|6666666.28487.peg.4578	Protein	37052	36135	-	Phosphate transport system permease protein PstA (TC 3.A.1.7.1)
contig_55	fig|6666666.28487.peg.4579	Protein	38161	37049	-	Phosphate transport system permease protein PstC (TC 3.A.1.7.1)
contig_55	fig|6666666.28487.peg.4580	Protein	39331	38198	-	Phosphate ABC transporter, periplasmic phosphate-binding protein PstS (TC 3.A.1.7.1)
contig_55	fig|6666666.28487.peg.4581	Protein	40355	39447	-	Acetyl-CoA:Cys-GlcN-Ins acetyltransferase, mycothiol synthase MshD
contig_55	fig|6666666.28487.peg.4582	Protein	41161	40352	-	Phosphate regulon transcriptional regulatory protein PhoB (SphR)

contig_55	fig|6666666.28487.peg.4583	Protein	41336	42130	+	FIG00821015: hypothetical protein
contig_55	fig|6666666.28487.peg.4584	Protein	42127	42558	+	Thioredoxin
contig_55	fig|6666666.28487.peg.4585	Protein	42768	43229	+	Possible membrane protein
contig_55	fig|6666666.28487.peg.4586	Protein	43250	44083	+	Thiosulfate sulfurtransferase, rhodanese (EC 2.8.1.1)
contig_55	fig|6666666.28487.peg.4587	Protein	44115	44387	+	protein of unknown function DUF1416
contig_55	fig|6666666.28487.peg.4588	Protein	44556	44879	+	FIG00821284: hypothetical protein
contig_55	fig|6666666.28487.peg.4589	Protein	45041	45670	+	DUF1794
contig_55	fig|6666666.28487.peg.4590	Protein	45670	45936	+	FIG00823372: hypothetical protein
contig_55	fig|6666666.28487.peg.4591	Protein	46798	45929	-	Amino acid ABC transporter, periplasmic amino acid-binding protein
contig_55	fig|6666666.28487.peg.4592	Protein	47954	47073	-	Aminodeoxychorismate lyase (EC 4.1.3.38)
contig_55	fig|6666666.28487.peg.4593	Protein	48006	49100	+	Folate-dependent protein for Fe/S cluster synthesis/repair in oxidative stress
contig_55	fig|6666666.28487.peg.4594	Protein	49248	49430	+	FIG00821405: hypothetical protein
contig_55	fig|6666666.28487.peg.4595	Protein	50543	49443	-	Phosphoribosylformylglycinamidine cyclo-ligase (EC 6.3.3.1)
contig_55	fig|6666666.28487.peg.4596	Protein	50608	51054	+	hypothetical protein
contig_55	fig|6666666.28487.peg.4597	Protein	51062	52069	+	Nucleoside-diphosphate-sugar epimerase
contig_55	fig|6666666.28487.peg.4598	Protein	53670	52126	-	Amidophosphoribosyltransferase (EC 2.4.2.14)
contig_55	fig|6666666.28487.peg.4599	Protein	54198	53806	-	FIG00820565: hypothetical protein
contig_55	fig|6666666.28487.peg.4600	Protein	54281	54694	+	FIG00829945: hypothetical protein

contig_55	fig|6666666.28487.peg.4601	Protein	55317	54691	-	3',5'-cyclic-nucleotide phosphodiesterase (EC 3.1.4.17)
contig_55	fig|6666666.28487.peg.4602	Protein	57044	55317	-	Predicted membrane protein (DUF2319)
contig_55	fig|6666666.28487.peg.4603	Protein	59352	57055	-	Phosphoribosylformylglycinamidine synthase, synthetase subunit (EC 6.3.5.3)
contig_55	fig|6666666.28487.peg.4604	Protein	59737	59396	-	glyoxalase family protein superfamily
contig_55	fig|6666666.28487.peg.4605	Protein	60949	59753	-	Aspartyl aminopeptidase
contig_55	fig|6666666.28487.peg.4606	Protein	61115	62065	+	Predicted dye-decolorizing peroxidase (DyP), encapsulated subgroup
contig_55	fig|6666666.28487.peg.4607	Protein	62065	62862	+	Encapsulating protein for a DyP-type peroxidase or ferritin-like protein oligomers
contig_55	fig|6666666.28487.peg.4608	Protein	63614	62952	-	Phosphoribosylformylglycinamidine synthase, glutamine amidotransferase subunit (EC 6.3.5.3)
contig_55	fig|6666666.28487.peg.4609	Protein	63862	63620	-	Phosphoribosylformylglycinamidine synthase, PurS subunit (EC 6.3.5.3)
contig_55	fig|6666666.28487.peg.4610	Protein	64576	63872	-	FIG00820729: hypothetical protein
contig_55	fig|6666666.28487.peg.4611	Protein	64642	65262	+	FIG00998332: hypothetical protein
contig_55	fig|6666666.28487.peg.4612	Protein	66979	65342	-	Fumarate/succinate/L-aspartate dehydrogenases
contig_55	fig|6666666.28487.peg.4613	Protein	67721	67017	-	FIG00998196: hypothetical protein
contig_55	fig|6666666.28487.peg.4614	Protein	68277	67789	-	glutathione peroxidase
contig_55	fig|6666666.28487.peg.4615	Protein	68869	68288	-	Transcriptional regulator, TetR family
contig_55	fig|6666666.28487.peg.4616	Protein	71007	68887	-	Protease II (EC 3.4.21.83)
contig_55	fig|6666666.28487.peg.4617	Protein	71897	71004	-	Phosphoribosylaminoimidazole-succinocarboxamide synthase

						(EC 6.3.2.6)
contig_55	fig|6666666.28487.peg.4618	Protein	72011	72643	+	POSSIBLE CONSERVED TRANSMEMBRANE PROTEIN
contig_55	fig|6666666.28487.peg.4619	Protein	73246	72719	-	FIG00826623: hypothetical protein
contig_55	fig|6666666.28487.peg.4620	Protein	74797	73373	-	Adenylosuccinate lyase (EC 4.3.2.2)
contig_55	fig|6666666.28487.peg.4621	Protein	74847	76568	+	FIG00823336: hypothetical protein
contig_55	fig|6666666.28487.peg.4622	Protein	78007	76565	-	Amino acid permease-associated region
contig_55	fig|6666666.28487.peg.4623	Protein	79075	78035	-	dehydrogenase/reductase
contig_55	fig|6666666.28487.peg.4624	Protein	79787	79128	-	Transcriptional regulator, TetR family
contig_55	fig|6666666.28487.peg.4625	Protein	79853	80707	+	Gamma-glutamyltranspeptidase (EC 2.3.2.2)
contig_55	fig|6666666.28487.peg.4626	Protein	81978	80704	-	Phosphoribosylamine--glycine ligase (EC 6.3.4.13)
contig_55	fig|6666666.28487.peg.4627	Protein	82094	83320	+	putative cytochrome P450 hydroxylase
contig_55	fig|6666666.28487.peg.4628	Protein	83351	83944	+	Transcriptional regulator, TetR family
contig_55	fig|6666666.28487.peg.4629	Protein	83994	85211	+	hypothetical protein
contig_55	fig|6666666.28487.peg.4630	Protein	85214	85642	+	hypothetical protein
contig_55	fig|6666666.28487.peg.4631	Protein	86956	85730	-	FIG00828709: hypothetical protein
contig_55	fig|6666666.28487.peg.4632	Protein	87389	86946	-	4-carboxymuconolactone decarboxylase (EC 4.1.1.44)
contig_55	fig|6666666.28487.peg.4633	Protein	88289	87396	-	3-hydroxyisobutyrate dehydrogenase (EC 1.1.1.31)
contig_55	fig|6666666.28487.peg.4634	Protein	89047	88286	-	3-oxoacyl-[acyl-carrier protein] reductase (EC 1.1.1.100)
contig_55	fig|6666666.28487.peg.4635	Protein	90529	89060	-	Aldehyde dehydrogenase (EC 1.2.1.3)

contig_55	fig|6666666.28487.peg.4636	Protein	90703	91299	+	Transcriptional regulator, TetR family
contig_55	fig|6666666.28487.peg.4637	Protein	91296	92504	+	putative cytochrome P450 hydroxylase
contig_55	fig|6666666.28487.peg.4638	Protein	92541	93365	+	oxidoreductase, short-chain dehydrogenase/reductase family
contig_55	fig|6666666.28487.peg.4639	Protein	93362	94717	+	Cytochrome P450 51
contig_55	fig|6666666.28487.peg.4640	Protein	94733	94939	+	Ferredoxin
contig_55	fig|6666666.28487.peg.4641	Protein	94942	95481	+	FIG00994119: hypothetical protein
contig_55	fig|6666666.28487.peg.4642	Protein	95575	96708	+	Alcohol dehydrogenase (EC 1.1.1.1)
contig_55	fig|6666666.28487.peg.4643	Protein	96800	97240	+	steroid isomerase, putative
contig_55	fig|6666666.28487.peg.4644	Protein	97263	98084	+	Short-chain dehydrogenase/reductase SDR
contig_55	fig|6666666.28487.peg.4645	Protein	99732	98086	-	AMP-dependent synthetase and ligase
contig_55	fig|6666666.28487.peg.4646	Protein	99855	100121	+	hypothetical protein
contig_55	fig|6666666.28487.peg.4647	Protein	100146	100550	+	HIT family protein
contig_55	fig|6666666.28487.peg.4648	Protein	101970	100522	-	PhoR
contig_56	fig|6666666.28487.peg.4649	Protein	173	349	+	hypothetical protein
contig_56	fig|6666666.28487.peg.4650	Protein	876	715	-	hypothetical protein
contig_56	fig|6666666.28487.peg.4651	Protein	1413	1658	+	hypothetical protein
contig_56	fig|6666666.28487.peg.4652	Protein	3141	1693	-	hypothetical protein
contig_56	fig|6666666.28487.peg.4653	Protein	3120	4925	+	hypothetical protein
contig_56	fig|6666666.28487.peg.4654	Protein	6255	4963	-	hypothetical protein

contig_56	fig|6666666.28487.peg.4655	Protein	6407	7075	+	Uracil-DNA glycosylase superfamily
contig_56	fig|6666666.28487.peg.4656	Protein	7039	7542	+	hypothetical protein
contig_56	fig|6666666.28487.peg.4657	Protein	7614	8273	+	FIG00823833: hypothetical protein
contig_56	fig|6666666.28487.peg.4658	Protein	9656	8673	-	MOSC domain containing protein
contig_56	fig|6666666.28487.peg.4659	Protein	9919	10665	+	3-oxoacyl-[acyl-carrier protein] reductase (EC 1.1.1.100)
contig_56	fig|6666666.28487.peg.4660	Protein	10802	10662	-	hypothetical protein
contig_56	fig|6666666.28487.peg.4661	Protein	10848	11615	+	Transcriptional regulator, MerR family
contig_56	fig|6666666.28487.peg.4662	Protein	13842	11566	-	Dipeptidyl peptidase IV
contig_56	fig|6666666.28487.peg.4663	Protein	14423	14040	-	putative exported protein of unknown function
contig_56	fig|6666666.28487.peg.4664	Protein	14637	14837	+	hypothetical protein
contig_56	fig|6666666.28487.peg.4665	Protein	15391	14834	-	conserved hypothetical protein
contig_56	fig|6666666.28487.peg.4666	Protein	17256	15400	-	protein of unknown function DUF1023
contig_56	fig|6666666.28487.peg.4667	Protein	17355	17624	+	FIG00314463: hypothetical protein
contig_56	fig|6666666.28487.peg.4668	Protein	18441	17644	-	2-aminoethylphosphonate ABC transporter permease protein II (TC 3.A.1.9.1)
contig_56	fig|6666666.28487.peg.4669	Protein	19312	18416	-	2-aminoethylphosphonate ABC transporter permease protein I (TC 3.A.1.9.1)
contig_56	fig|6666666.28487.peg.4670	Protein	20478	19309	-	2-aminoethylphosphonate ABC transporter ATP-binding protein (TC 3.A.1.9.1)
contig_56	fig|6666666.28487.peg.4671	Protein	21520	20480	-	Ferric iron ABC transporter, iron-binding protein
contig_56	fig|6666666.28487.peg.4672	Protein	22212	21523	-	Similar to phosphoglycolate phosphatase, clustered with

						ribosomal large subunit pseudouridine synthase C
contig_56	fig|6666666.28487.peg.4673	Protein	23324	22209	-	putative secreted oxidoreductase
contig_56	fig|6666666.28487.peg.4674	Protein	23435	23962	+	FIG00825894: hypothetical protein
contig_56	fig|6666666.28487.peg.4675	Protein	23959	24657	+	Predicted transcriptional regulator of N-Acetylglucosamine utilization, GntR family
contig_56	fig|6666666.28487.peg.4676	Protein	24695	25327	+	hypothetical protein
contig_56	fig|6666666.28487.peg.4677	Protein	25380	25991	+	FIG00829536: hypothetical protein
contig_56	fig|6666666.28487.peg.4678	Protein	26223	26011	-	hypothetical protein
contig_56	fig|6666666.28487.peg.4679	Protein	26320	26841	+	FIG00826191: hypothetical protein
contig_56	fig|6666666.28487.peg.4680	Protein	27613	26813	-	TesB-like acyl-CoA thioesterase 4
contig_56	fig|6666666.28487.peg.4681	Protein	27982	27650	-	FIG00826788: hypothetical protein
contig_56	fig|6666666.28487.peg.4682	Protein	28967	27990	-	FIG00820769: hypothetical protein
contig_56	fig|6666666.28487.peg.4683	Protein	29404	28964	-	FIG00824211: hypothetical protein
contig_56	fig|6666666.28487.peg.4684	Protein	29488	29757	+	FIG00827427: hypothetical protein
contig_56	fig|6666666.28487.peg.4685	Protein	29750	31312	+	Propionyl-CoA carboxylase beta chain (EC 6.4.1.3)
contig_56	fig|6666666.28487.peg.4686	Protein	31777	31484	-	Transcriptional regulator WhiB-like WhiB6
contig_56	fig|6666666.28487.peg.4687	Protein	31734	31847	+	hypothetical protein
contig_56	fig|6666666.28487.peg.4688	Protein	32093	32833	+	Probable forkhead-associated protein
contig_56	fig|6666666.28487.peg.4689	Protein	34054	32837	-	Antiactivator of flagellar biosynthesis FleN, an ATPase
contig_56	fig|6666666.28487.peg.4690	Protein	34471	34334	-	hypothetical protein

contig_56	fig|6666666.28487.peg.4691	Protein	34490	35347	+	RD1 region associated protein Rv3866
contig_56	fig|6666666.28487.peg.4692	Protein	35366	35890	+	RD1 region associated protein Rv3867
contig_56	fig|6666666.28487.peg.4693	Protein	35887	37611	+	AAA family ATPase, ESX-1 secretion system component Rv3868
contig_56	fig|6666666.28487.peg.4694	Protein	37615	39090	+	Putative ESX-1 secretion system component Rv3869
contig_56	fig|6666666.28487.peg.4695	Protein	39087	41318	+	FtsK/SpoIIIE family protein Rv3870, component of ESX-1 secretion system
contig_56	fig|6666666.28487.peg.4696	Protein	41315	43066	+	FtsK/SpoIIIE family protein Rv3871, component of ESX-1 secretion system
contig_56	fig|6666666.28487.peg.4697	Protein	43246	43539	+	PE family protein
contig_56	fig|6666666.28487.peg.4698	Protein	43568	44884	+	Putative ESX-1 secretion system gating protein PPE68
contig_56	fig|6666666.28487.peg.4699	Protein	45021	45302	+	hypothetical protein
contig_56	fig|6666666.28487.peg.4700	Protein	45345	45641	+	early secretory antigenic target, 6 kDa
contig_56	fig|6666666.28487.peg.4701	Protein	45734	47128	+	RD1 region associated protein Rv3876
contig_56	fig|6666666.28487.peg.4702	Protein	47215	48651	+	Putative ESX-1 secretion system component Rv3877
contig_56	fig|6666666.28487.peg.4703	Protein	48798	49421	+	RD1 region associated protein Rv3878
contig_56	fig|6666666.28487.peg.4704	Protein	49418	49726	+	hypothetical protein
contig_56	fig|6666666.28487.peg.4705	Protein	49763	49996	+	hypothetical protein
contig_56	fig|6666666.28487.peg.4706	Protein	50344	50180	-	hypothetical protein
contig_56	fig|6666666.28487.peg.4707	Protein	51360	50323	-	FIG00823379: hypothetical protein
contig_56	fig|6666666.28487.peg.4708	Protein	52248	51376	-	RD1 region associated protein Rv3879c

contig_56	fig|6666666.28487.peg.4709	Protein	52631	52852	+	hypothetical protein
contig_56	fig|6666666.28487.peg.4710	Protein	55060	54791	-	hypothetical protein
contig_56	fig|6666666.28487.peg.4711	Protein	55299	55057	-	hypothetical protein
contig_56	fig|6666666.28487.peg.4712	Protein	55787	55443	-	FIG00821130: hypothetical protein
contig_56	fig|6666666.28487.peg.4713	Protein	57415	55787	-	FIG028708: hypothetical protein
contig_56	fig|6666666.28487.peg.4714	Protein	58844	57447	-	Cell division protein DivIC (FtsB), stabilizes FtsL against RasP cleavage
contig_56	fig|6666666.28487.peg.4715	Protein	60187	58841	-	serine protease
contig_56	fig|6666666.28487.peg.4716	Protein	60627	60370	-	Phosphotransferase system, phosphocarrier protein HPr
contig_56	fig|6666666.28487.peg.4717	Protein	62645	60672	-	PTS system, fructose-specific IIA component (EC 2.7.1.69) / PTS system, fructose-specific IIB component (EC 2.7.1.69) / PTS system, fructose-specific IIC component (EC 2.7.1.69)
contig_56	fig|6666666.28487.peg.4718	Protein	63644	62667	-	1-phosphofructokinase (EC 2.7.1.56)
contig_56	fig|6666666.28487.peg.4719	Protein	64420	63641	-	Transcriptional repressor of the fructose operon, DeoR family
contig_56	fig|6666666.28487.peg.4720	Protein	64544	66229	+	Phosphoenolpyruvate-protein phosphotransferase of PTS system (EC 2.7.3.9)
contig_56	fig|6666666.28487.peg.4721	Protein	66456	67355	+	Pirin
contig_56	fig|6666666.28487.peg.4722	Protein	69493	67358	-	FIG00824951: hypothetical protein
contig_56	fig|6666666.28487.peg.4723	Protein	69530	69658	+	hypothetical protein
contig_56	fig|6666666.28487.peg.4724	Protein	70027	70590	+	Transcriptional regulator, TetR family
contig_56	fig|6666666.28487.peg.4725	Protein	70739	71668	+	FIG00823034: hypothetical protein

contig_56	fig|6666666.28487.peg.4726	Protein	71747	73153	+	Aldehyde dehydrogenase (EC 1.2.1.3)
contig_56	fig|6666666.28487.peg.4727	Protein	73196	74059	+	Short-chain dehydrogenase/reductase SDR
contig_56	fig|6666666.28487.peg.4728	Protein	74065	75027	+	Quinone oxidoreductase (EC 1.6.5.5)
contig_56	fig|6666666.28487.peg.4729	Protein	75027	75725	+	Haloacid dehalogenase, type II (EC 3.8.1.2)
contig_56	fig|6666666.28487.peg.4730	Protein	75748	76482	+	methyltransferase, UbiE/COQ5 family
contig_56	fig|6666666.28487.peg.4731	Protein	76501	76983	+	Small hydrophobic protein
contig_57	fig|6666666.28487.peg.4732	Protein	419	2251	+	hypothetical protein
contig_57	fig|6666666.28487.peg.4733	Protein	3794	2328	-	putative sugar transporter
contig_57	fig|6666666.28487.peg.4734	Protein	3993	6569	+	hypothetical protein
contig_57	fig|6666666.28487.peg.4735	Protein	6669	6803	+	hypothetical protein
contig_57	fig|6666666.28487.peg.4736	Protein	6914	9685	+	hypothetical protein
contig_57	fig|6666666.28487.peg.4737	Protein	10664	9657	-	1-aminocyclopropane-1-carboxylate deaminase (EC 3.5.99.7)
contig_57	fig|6666666.28487.peg.4738	Protein	10747	11403	+	probable transcriptional regulator YdhC
contig_57	fig|6666666.28487.peg.4739	Protein	11668	11441	-	hypothetical protein
contig_57	fig|6666666.28487.peg.4740	Protein	11687	12388	+	hypothetical protein
contig_57	fig|6666666.28487.peg.4741	Protein	12422	13243	+	Probable signal peptide protein
contig_57	fig|6666666.28487.peg.4742	Protein	13275	14864	+	Choline dehydrogenase (EC 1.1.99.1)
contig_57	fig|6666666.28487.peg.4743	Protein	15358	14816	-	hypothetical protein
contig_57	fig|6666666.28487.peg.4744	Protein	15809	16990	+	2-polyprenyl-6-methoxyphenol hydroxylase and related FAD-dependent oxidoreductases

contig_57	fig|6666666.28487.peg.4745	Protein	17001	17780	+	5-keto-D-gluconate 5-reductase (EC 1.1.1.69)
contig_57	fig|6666666.28487.peg.4746	Protein	17977	17777	-	hypothetical protein
contig_57	fig|6666666.28487.peg.4747	Protein	18132	18854	+	FIG00831532: hypothetical protein
contig_57	fig|6666666.28487.peg.4748	Protein	20946	19141	-	Proline and threonine rich protein
contig_57	fig|6666666.28487.peg.4749	Protein	21085	21543	+	Bile acid 7-alpha dehydratase BaiE (EC 4.2.1.106)
contig_57	fig|6666666.28487.peg.4750	Protein	22198	21545	-	FIG019540: hypothetical protein
contig_57	fig|6666666.28487.peg.4751	Protein	23027	22215	-	FIG00826552: hypothetical protein
contig_57	fig|6666666.28487.peg.4752	Protein	23618	23031	-	Predicted transcriptional regulators
contig_57	fig|6666666.28487.peg.4753	Protein	23683	25188	+	INTEGRAL MEMBRANE PROTEIN (Rhomboid family)
contig_57	fig|6666666.28487.peg.4754	Protein	25807	25142	-	phosphoesterase, PA-phosphatase related
contig_57	fig|6666666.28487.peg.4755	Protein	25874	26353	+	hypothetical protein
contig_57	fig|6666666.28487.peg.4756	Protein	26379	26861	+	FIG00820335: hypothetical protein
contig_57	fig|6666666.28487.peg.4757	Protein	27763	26858	-	DTDP-glucose 4,6-dehydratase (EC 4.2.1.46)
contig_57	fig|6666666.28487.peg.4758	Protein	28574	27810	-	Trans-aconitate 2-methyltransferase (EC 2.1.1.144)
contig_57	fig|6666666.28487.peg.4759	Protein	28926	28597	-	Transcriptional regulator, ArsR family
contig_57	fig|6666666.28487.peg.4760	Protein	29067	30947	+	Lead, cadmium, zinc and mercury transporting ATPase (EC 3.6.3.3) (EC 3.6.3.5); Copper-translocating P-type ATPase (EC 3.6.3.4)
contig_57	fig|6666666.28487.peg.4761	Protein	30973	32169	+	FIG00821889: hypothetical protein
contig_57	fig|6666666.28487.peg.4762	Protein	33070	32156	-	ESX-3 secretion system protein EccE3
contig_57	fig|6666666.28487.peg.4763	Protein	34428	33067	-	Type VII secretion-associated serine protease mycosin

						MycP3
contig_57	fig|6666666.28487.peg.4764	Protein	35892	34432	-	ESX-3 secretion system protein EccD3
contig_57	fig|6666666.28487.peg.4765	Protein	36746	35874	-	RD1 region associated protein Rv3866
contig_57	fig|6666666.28487.peg.4766	Protein	37043	36756	-	10 kDa antigen CFP7
contig_57	fig|6666666.28487.peg.4767	Protein	37351	37058	-	PE family protein
contig_57	fig|6666666.28487.peg.4768	Protein	38904	37348	-	PPE family protein
contig_57	fig|6666666.28487.peg.4769	Protein	39267	38956	-	PE family protein
contig_57	fig|6666666.28487.peg.4770	Protein	43301	39267	-	Type VII secretion protein EccCa
contig_57	fig|6666666.28487.peg.4771	Protein	44827	43298	-	Type VII secretion protein EccB
contig_57	fig|6666666.28487.peg.4772	Protein	46656	44824	-	AAA family ATPase, ESX-1 secretion system component Rv3868
contig_57	fig|6666666.28487.peg.4773	Protein	47683	46817	-	FIG00820338: hypothetical protein
contig_57	fig|6666666.28487.peg.4774	Protein	47783	48412	+	Transcriptional regulator, TetR family
contig_57	fig|6666666.28487.peg.4775	Protein	48981	48409	-	Glyoxalase family protein
contig_57	fig|6666666.28487.peg.4776	Protein	49107	50243	+	FIG00820646: hypothetical protein
contig_57	fig|6666666.28487.peg.4777	Protein	50240	51187	+	D-3-phosphoglycerate dehydrogenase (EC 1.1.1.95)
contig_57	fig|6666666.28487.peg.4778	Protein	51243	53441	+	Acyl-CoA dehydrogenase, short-chain specific (EC 1.3.99.2)
contig_57	fig|6666666.28487.peg.4779	Protein	54536	53454	-	diguanylate cyclase
contig_57	fig|6666666.28487.peg.4780	Protein	54832	55161	+	hypothetical protein
contig_57	fig|6666666.28487.peg.4781	Protein	55241	56503	+	hypothetical protein

contig_57	fig|6666666.28487.peg.4782	Protein	57296	56517	-	Short-chain dehydrogenase/reductase SDR
contig_57	fig|6666666.28487.peg.4783	Protein	57289	57405	+	hypothetical protein
contig_57	fig|6666666.28487.peg.4784	Protein	59099	57402	-	Long-chain-fatty-acid--CoA ligase (EC 6.2.1.3)
contig_57	fig|6666666.28487.peg.4785	Protein	59155	60393	+	ATP-dependent DNA ligase (EC 6.5.1.1)
contig_57	fig|6666666.28487.peg.4786	Protein	60541	60948	+	FIG00825806: hypothetical protein
contig_57	fig|6666666.28487.peg.4787	Protein	61085	62275	+	putative acyl-CoA dehydrogenase
contig_58	fig|6666666.28487.peg.4788	Protein	1447	2	-	GTP-binding protein EngA
contig_58	fig|6666666.28487.peg.4789	Protein	2130	1444	-	Cytidylate kinase (EC 2.7.4.14)
contig_58	fig|6666666.28487.peg.4790	Protein	2870	2127	-	Ribosomal large subunit pseudouridine synthase B (EC 4.2.1.70)
contig_58	fig|6666666.28487.peg.4791	Protein	3598	2873	-	Segregation and condensation protein B
contig_58	fig|6666666.28487.peg.4792	Protein	4421	3606	-	Segregation and condensation protein A
contig_58	fig|6666666.28487.peg.4793	Protein	5287	4418	-	Chromosome (plasmid) partitioning protein ParA / Sporulation initiation inhibitor protein Soj
contig_58	fig|6666666.28487.peg.4794	Protein	5413	6147	+	Probable catechol-o-methyltransferase (EC 2.1.1.6)
contig_58	fig|6666666.28487.peg.4795	Protein	7101	6148	-	Tyrosine recombinase XerD
contig_58	fig|6666666.28487.peg.4796	Protein	7733	7098	-	ADP-ribose pyrophosphatase (EC 3.6.1.13)
contig_58	fig|6666666.28487.peg.4797	Protein	9477	7726	-	CTP synthase (EC 6.3.4.2)
contig_58	fig|6666666.28487.peg.4798	Protein	10542	9601	-	FIG007481: hypothetical protein
contig_58	fig|6666666.28487.peg.4799	Protein	11737	10553	-	FIG005773: conserved membrane protein ML1361

contig_58	fig|6666666.28487.peg.4800	Protein	13642	11840	-	DNA repair protein RecN
contig_58	fig|6666666.28487.peg.4801	Protein	14571	13642	-	NAD kinase (EC 2.7.1.23)
contig_58	fig|6666666.28487.peg.4802	Protein	15356	14568	-	RNA binding methyltransferase FtsJ like
contig_58	fig|6666666.28487.peg.4803	Protein	15547	15380	-	FIG00820006: hypothetical protein
contig_58	fig|6666666.28487.peg.4804	Protein	16562	15549	-	4-nitrophenylphosphatase (EC 3.1.3.41)
contig_58	fig|6666666.28487.peg.4805	Protein	17388	16564	-	TPR-repeat-containing protein
contig_6	fig|6666666.28487.peg.4806	Protein	380	6	-	3-demethylubiquinone-9 3-methyltransferase
contig_6	fig|6666666.28487.peg.4807	Protein	1017	454	-	Transcriptional regulator, TetR family
contig_6	fig|6666666.28487.peg.4808	Protein	1161	2036	+	Possible peroxidase bpoB (Non-haem peroxidase) (EC 1.11.1.-)
contig_6	fig|6666666.28487.peg.4809	Protein	2039	4927	+	aminotransferase, class III
contig_6	fig|6666666.28487.peg.4810	Protein	5039	5389	+	Limonene-1,2-epoxide hydrolase (EC 3.3.2.8)
contig_6	fig|6666666.28487.peg.4811	Protein	6321	5386	-	FIG00830670: hypothetical protein
contig_6	fig|6666666.28487.peg.4812	Protein	6347	7147	+	Putative adenylate/guanylate cyclase
contig_6	fig|6666666.28487.peg.4813	Protein	7275	7114	-	hypothetical protein
contig_6	fig|6666666.28487.peg.4814	Protein	7318	7995	+	FIG022780: hypothetical protein
contig_6	fig|6666666.28487.peg.4815	Protein	8100	8717	+	FIG00825717: hypothetical protein
contig_6	fig|6666666.28487.peg.4816	Protein	9204	8767	-	protein of unknown function DUF1486
contig_6	fig|6666666.28487.peg.4817	Protein	9640	9185	-	FIG00830871: hypothetical protein
contig_6	fig|6666666.28487.peg.4818	Protein	10028	10300	+	hypothetical protein

contig_6	fig|6666666.28487.peg.4819	Protein	10320	10814	+	FIG00821929: hypothetical protein
contig_6	fig|6666666.28487.peg.4820	Protein	11045	11473	+	Rifampin ADP-ribosyl transferase
contig_6	fig|6666666.28487.peg.4821	Protein	11850	11497	-	conserved hypothetical protein
contig_6	fig|6666666.28487.peg.4822	Protein	12095	12697	+	Methyltransferase type 11
contig_6	fig|6666666.28487.peg.4823	Protein	13871	12798	-	GTP-binding and nucleic acid-binding protein YchF
contig_6	fig|6666666.28487.peg.4824	Protein	13981	15039	+	INTEGRAL MEMBRANE PROTEIN (Rhomboid family)
contig_6	fig|6666666.28487.peg.4825	Protein	16880	15117	-	Cell division protein FtsI [Peptidoglycan synthetase] (EC 2.4.1.129)
contig_6	fig|6666666.28487.peg.4826	Protein	17815	16886	-	4-hydroxy-3-methylbut-2-enyl diphosphate reductase (EC 1.17.1.2)
contig_6	fig|6666666.28487.peg.4827	Protein	17973	18539	+	FIG00997309: hypothetical protein
contig_6	fig|6666666.28487.peg.4828	Protein	18536	19780	+	Exodeoxyribonuclease VII large subunit (EC 3.1.11.6)
contig_6	fig|6666666.28487.peg.4829	Protein	19777	19989	+	Exodeoxyribonuclease VII small subunit (EC 3.1.11.6)
contig_6	fig|6666666.28487.peg.4830	Protein	20065	21177	+	PROBABLE CHOLESTEROL DEHYDROGENASE
contig_6	fig|6666666.28487.peg.4831	Protein	21223	21933	+	Beta-ketoadipate enol-lactone hydrolase (EC 3.1.1.24)
contig_6	fig|6666666.28487.peg.4832	Protein	24267	21904	-	FIG00831608: hypothetical protein
contig_6	fig|6666666.28487.peg.4833	Protein	24666	26240	+	Arylsulfatase (EC 3.1.6.1)
contig_6	fig|6666666.28487.peg.4834	Protein	26243	27142	+	FIG068086: hypothetical protein
contig_6	fig|6666666.28487.peg.4835	Protein	27156	28019	+	Sulfatase modifying factor 1 precursor (C-alpha-formyglycine-generating enzyme 1)
contig_6	fig|6666666.28487.peg.4836	Protein	28626	28024	-	FIG00831608: hypothetical protein

contig_6	fig|6666666.28487.peg.4837	Protein	30366	28657	-	FIG00831608: hypothetical protein
contig_6	fig|6666666.28487.peg.4838	Protein	30523	31140	+	Dihydrofolate reductase (EC 1.5.1.3)
contig_6	fig|6666666.28487.peg.4839	Protein	31684	31154	-	FIG00827129: hypothetical protein
contig_6	fig|6666666.28487.peg.4840	Protein	31708	32490	+	3-oxoacyl-[acyl-carrier protein] reductase (EC 1.1.1.100)
contig_6	fig|6666666.28487.peg.4841	Protein	32487	33356	+	Short chain dehydrogenase
contig_6	fig|6666666.28487.peg.4842	Protein	33409	34620	+	CONSERVED MEMBRANE PROTEIN
contig_6	fig|6666666.28487.peg.4843	Protein	35456	34638	-	Dienelactone hydrolase family protein
contig_6	fig|6666666.28487.peg.4844	Protein	36207	35470	-	FIG00820022: hypothetical protein
contig_6	fig|6666666.28487.peg.4845	Protein	36335	37288	+	Fructose-1,6-bisphosphatase, GlpX type (EC 3.1.3.11)
contig_6	fig|6666666.28487.peg.4846	Protein	37323	38756	+	Fumarate hydratase class II (EC 4.2.1.2)
contig_6	fig|6666666.28487.peg.4847	Protein	38908	39132	+	FIG00820363: hypothetical protein
contig_6	fig|6666666.28487.peg.4848	Protein	39877	39146	-	Possible glycosyl hydrolase (EC 3.-.-.-)
contig_6	fig|6666666.28487.peg.4849	Protein	41226	39874	-	Predicted ATPase related to phosphate starvation-inducible protein PhoH
contig_6	fig|6666666.28487.peg.4850	Protein	42215	41391	-	Fatty acid desaturase occurring in virulence cluster
contig_6	fig|6666666.28487.peg.4851	Protein	43814	42351	-	Serine hydroxymethyltransferase (EC 2.1.2.1)
contig_6	fig|6666666.28487.peg.4852	Protein	45100	43853	-	FIG00993983: hypothetical protein
contig_6	fig|6666666.28487.peg.4853	Protein	45161	46081	+	Pantothenate kinase (EC 2.7.1.33)
contig_6	fig|6666666.28487.peg.4854	Protein	46495	46085	-	FIG00820427: hypothetical protein
contig_6	fig|6666666.28487.peg.4855	Protein	46938	46525	-	hypothetical protein

contig_6	fig|6666666.28487.peg.4856	Protein	47442	47026	-	FIG00820427: hypothetical protein
contig_6	fig|6666666.28487.peg.4857	Protein	48870	47521	-	FIG00821548: hypothetical protein
contig_6	fig|6666666.28487.peg.4858	Protein	49663	48872	-	Undecaprenyl pyrophosphate synthetase (EC 2.5.1.31)
contig_6	fig|6666666.28487.peg.4859	Protein	49720	50463	+	COG1272: Predicted membrane protein hemolysin III homolog
contig_6	fig|6666666.28487.peg.4860	Protein	50988	50617	-	Steroid delta-isomerase (EC 5.3.3.1)
contig_6	fig|6666666.28487.peg.4861	Protein	53002	50999	-	Thymidylate kinase (EC 2.7.4.9)
contig_6	fig|6666666.28487.peg.4862	Protein	53267	52989	-	hypothetical protein
contig_6	fig|6666666.28487.peg.4863	Protein	54136	53264	-	Mycothiol S-conjugate amidase Mca
contig_6	fig|6666666.28487.peg.4864	Protein	54242	54676	+	FIG00821098: hypothetical protein
contig_6	fig|6666666.28487.peg.4865	Protein	54916	55410	+	Transcription elongation factor GreA
contig_6	fig|6666666.28487.peg.4866	Protein	55449	56120	+	Phosphoglycerate dehydrogenase and related dehydrogenases
contig_6	fig|6666666.28487.peg.4867	Protein	57328	56117	-	Cystathionine gamma-lyase (EC 4.4.1.1)
contig_6	fig|6666666.28487.peg.4868	Protein	57321	58511	+	Glutaryl-CoA dehydrogenase (EC 1.3.99.7)
contig_6	fig|6666666.28487.peg.4869	Protein	58908	58495	-	Proline-rich antigen homolog
contig_6	fig|6666666.28487.peg.4870	Protein	60024	59050	-	Uncharacterized protein Mb2590
contig_6	fig|6666666.28487.peg.4871	Protein	60328	60050	-	Proline-rich antigen homolog
contig_6	fig|6666666.28487.peg.4872	Protein	62002	60608	-	Cystathionine beta-synthase (EC 4.2.1.22)
contig_6	fig|6666666.28487.peg.4873	Protein	63133	62057	-	esterase

contig_6	fig|6666666.28487.peg.4874	Protein	63271	64302	+	CONSERVED EXPORTED PROTEIN
contig_6	fig|6666666.28487.peg.4875	Protein	64351	65568	+	3-ketoacyl-CoA thiolase (EC 2.3.1.16) @ Acetyl-CoA acetyltransferase (EC 2.3.1.9)
contig_6	fig|6666666.28487.peg.4876	Protein	65587	66786	+	Phosphoribosylglycinamide formyltransferase 2 (EC 2.1.2.-)
contig_6	fig|6666666.28487.peg.4877	Protein	67703	66861	-	PROBABLE CONSERVED TRANSMEMBRANE PROTEIN
contig_6	fig|6666666.28487.peg.4878	Protein	67898	68944	+	3-hydroxyisobutyryl-CoA hydrolase (EC 3.1.2.4)
contig_6	fig|6666666.28487.peg.4879	Protein	68941	69714	+	Enoyl-CoA hydratase [valine degradation] (EC 4.2.1.17)
contig_6	fig|6666666.28487.peg.4880	Protein	69724	71427	+	Predicted membrane protein (DUF2319)
contig_6	fig|6666666.28487.peg.4881	Protein	71845	71477	-	FIG00820003: hypothetical protein
contig_6	fig|6666666.28487.peg.4882	Protein	72366	71845	-	FIG00822063: hypothetical protein
contig_6	fig|6666666.28487.peg.4883	Protein	72769	72566	-	hypothetical protein
contig_6	fig|6666666.28487.peg.4884	Protein	72741	73016	+	PUTATIVE LIPOPROTEIN LPQV
contig_6	fig|6666666.28487.peg.4885	Protein	73375	73013	-	hypothetical protein
contig_6	fig|6666666.28487.peg.4886	Protein	73427	74392	+	UPF0028 protein YchK
contig_6	fig|6666666.28487.peg.4887	Protein	75241	74381	-	probably phosphoesterase, evidenced by COGnitor
contig_6	fig|6666666.28487.peg.4888	Protein	76378	75329	-	FIG00821199: hypothetical protein
contig_6	fig|6666666.28487.peg.4889	Protein	76545	78317	+	Serine/threonine protein kinase (EC 2.7.11.1)
contig_6	fig|6666666.28487.peg.4890	Protein	78379	79557	+	3-ketoacyl-CoA thiolase (EC 2.3.1.16)
contig_6	fig|6666666.28487.peg.4891	Protein	79729	80037	+	hypothetical protein
contig_6	fig|6666666.28487.peg.4892	Protein	81031	80108	-	putative sodium:dicarboxylate symporter

contig_6	fig|6666666.28487.peg.4893	Protein	81064	81606	+	sensor kinase citA VC0791 [imported], putative
contig_6	fig|6666666.28487.peg.4894	Protein	81603	82301	+	response regulator VC1604 [imported]
contig_6	fig|6666666.28487.peg.4895	Protein	83406	82324	-	surface antigen, putative
contig_6	fig|6666666.28487.peg.4896	Protein	84133	83861	-	anti-sigma-factor antagonist
contig_6	fig|6666666.28487.peg.4897	Protein	84535	84299	-	CsbD family protein
contig_6	fig|6666666.28487.peg.4898	Protein	84967	84629	-	Anti-sigma F factor antagonist (spoIIAA-2); Anti-sigma B factor antagonist RsbV
contig_6	fig|6666666.28487.peg.4899	Protein	85281	85631	+	protein of unknown function DUF732
contig_6	fig|6666666.28487.peg.4900	Protein	85793	86092	+	hypothetical protein
contig_6	fig|6666666.28487.peg.4901	Protein	86195	87526	+	hypothetical protein
contig_6	fig|6666666.28487.peg.4902	Protein	87694	87996	+	hypothetical protein
contig_6	fig|6666666.28487.peg.4903	Protein	88070	88309	+	FIG00832514: hypothetical protein
contig_6	fig|6666666.28487.peg.4904	Protein	89095	88322	-	PROBABLE CONSERVED TRANSMEMBRANE PROTEIN
contig_6	fig|6666666.28487.peg.4905	Protein	89489	89971	+	hypothetical protein
contig_6	fig|6666666.28487.peg.4906	Protein	90347	89979	-	integral membrane protein
contig_6	fig|6666666.28487.peg.4907	Protein	90450	91328	+	RNA polymerase, sigma-24 subunit, ECF subfamily
contig_6	fig|6666666.28487.peg.4908	Protein	92590	91310	-	Acetamidase/Formamidase family protein
contig_6	fig|6666666.28487.peg.4909	Protein	93064	92927	-	hypothetical protein
contig_6	fig|6666666.28487.peg.4910	Protein	93035	93346	+	hypothetical protein
contig_6	fig|6666666.28487.peg.4911	Protein	94154	93321	-	acetyltransferase, GNAT family family

contig_6	fig|6666666.28487.peg.4912	Protein	94251	94406	+	hypothetical protein
contig_6	fig|6666666.28487.peg.4913	Protein	94945	95241	+	protein of unknown function DUF732
contig_6	fig|6666666.28487.peg.4914	Protein	95619	95371	-	hypothetical protein
contig_6	fig|6666666.28487.peg.4915	Protein	96146	95799	-	hypothetical protein
contig_6	fig|6666666.28487.peg.4916	Protein	97687	96350	-	H(+)/Cl(-) exchange transporter ClcA
contig_6	fig|6666666.28487.peg.4917	Protein	97777	98559	+	protein of unknown function DUF427
contig_6	fig|6666666.28487.peg.4918	Protein	98822	98556	-	FIG00827997: hypothetical protein
contig_6	fig|6666666.28487.peg.4919	Protein	99134	99000	-	hypothetical protein
contig_6	fig|6666666.28487.peg.4920	Protein	99499	99131	-	hypothetical protein
contig_6	fig|6666666.28487.peg.4921	Protein	100245	99511	-	hypothetical protein
contig_6	fig|6666666.28487.peg.4922	Protein	100464	100303	-	hypothetical protein
contig_6	fig|6666666.28487.peg.4923	Protein	101341	100700	-	Horizontally Transferred TransMembrane Domain (HTTM) protein
contig_6	fig|6666666.28487.peg.4924	Protein	101595	101317	-	hypothetical protein
contig_6	fig|6666666.28487.peg.4925	Protein	102145	101606	-	hypothetical protein
contig_6	fig|6666666.28487.peg.4926	Protein	102384	102136	-	hypothetical protein
contig_6	fig|6666666.28487.peg.4927	Protein	102503	103009	+	collagen-like protein
contig_6	fig|6666666.28487.peg.4928	Protein	105132	103405	-	hypothetical protein
contig_6	fig|6666666.28487.peg.4929	Protein	105711	105262	-	Cyanate hydratase (EC 4.2.1.104)
contig_6	fig|6666666.28487.peg.4930	Protein	106571	105708	-	Nitrite transporter from formate/nitrite family

contig_6	fig|6666666.28487.peg.4931	Protein	108340	106688	-	Long-chain-fatty-acid--CoA ligase (EC 6.2.1.3)
contig_6	fig|6666666.28487.peg.4932	Protein	108768	108481	-	hypothetical protein
contig_6	fig|6666666.28487.peg.4933	Protein	109086	110264	+	PE-PPE, C-terminal domain protein
contig_6	fig|6666666.28487.peg.4934	Protein	110421	111053	+	Enoyl-[acyl-carrier-protein] reductase [NADPH] (EC 1.3.1.10)
contig_6	fig|6666666.28487.peg.4935	Protein	111170	112267	+	Ferredoxin
contig_6	fig|6666666.28487.peg.4936	Protein	112260	113111	+	2-polyprenylphenol hydroxylase and related flavodoxin oxidoreductases
contig_6	fig|6666666.28487.peg.4937	Protein	113114	113887	+	NAD-reducing hydrogenase subunit HoxY (EC 1.12.1.2)
contig_6	fig|6666666.28487.peg.4938	Protein	113884	115176	+	Cytochrome-C3 hydrogenase alpha chain
contig_6	fig|6666666.28487.peg.4939	Protein	115185	115628	+	hydrogenase maturation protease
contig_6	fig|6666666.28487.peg.4940	Protein	115785	116861	+	oxidoreductase, short-chain dehydrogenase/reductase family
contig_6	fig|6666666.28487.peg.4941	Protein	118232	116871	-	Diacyglycerol O-acyltransferase (EC 2.3.1.20)
contig_6	fig|6666666.28487.peg.4942	Protein	119217	118366	-	PROBABLE MEMBRANE PROTEIN
contig_6	fig|6666666.28487.peg.4943	Protein	119325	120032	+	transcriptional regulator, TetR family
contig_6	fig|6666666.28487.peg.4944	Protein	120195	121673	+	/ Glutamine transport system permease protein GlnP (TC 3.A.1.3.2)
contig_6	fig|6666666.28487.peg.4945	Protein	121670	122443	+	glutamine ABC transporter, ATP-binding protein
contig_6	fig|6666666.28487.peg.4946	Protein	122488	123414	+	amine oxidase, flavin-containing
contig_6	fig|6666666.28487.peg.4947	Protein	124267	123416	-	FIG00825453: hypothetical protein
contig_6	fig|6666666.28487.peg.4948	Protein	125028	124375	-	3-oxoacyl-[acyl-carrier protein] reductase (EC 1.1.1.100)

contig_6	fig|6666666.28487.peg.4949	Protein	126025	125147	-	Epoxide hydrolase (EC 3.3.2.9)
contig_6	fig|6666666.28487.peg.4950	Protein	126117	126233	+	hypothetical protein
contig_6	fig|6666666.28487.peg.4951	Protein	126257	127147	+	transcriptional regulator, LysR family
contig_6	fig|6666666.28487.peg.4952	Protein	127568	127158	-	hypothetical protein
contig_6	fig|6666666.28487.peg.4953	Protein	129234	127672	-	PPE family protein
contig_6	fig|6666666.28487.peg.4954	Protein	130984	129473	-	Partial REP13E12 repeat protein
contig_6	fig|6666666.28487.peg.4955	Protein	131905	131057	-	3-alpha-hydroxysteroid dehydrogenase (EC 1.1.1.50)
contig_6	fig|6666666.28487.peg.4956	Protein	133367	131952	-	Diacyglycerol O-acyltransferase (EC 2.3.1.20)
contig_6	fig|6666666.28487.peg.4957	Protein	133554	134726	+	FIG00821576: hypothetical protein
contig_6	fig|6666666.28487.peg.4958	Protein	135065	135340	+	hypothetical protein
contig_6	fig|6666666.28487.peg.4959	Protein	135372	136583	+	hypothetical protein
contig_6	fig|6666666.28487.peg.4960	Protein	137664	136684	-	hypothetical protein
contig_6	fig|6666666.28487.peg.4961	Protein	138400	137657	-	Hydantoin racemase (EC 5.1.99.-)
contig_6	fig|6666666.28487.peg.4962	Protein	139860	138397	-	Cytosine/purine/uracil/thiamine/allantoin permease family protein
contig_6	fig|6666666.28487.peg.4963	Protein	139879	140052	+	hypothetical protein
contig_6	fig|6666666.28487.peg.4964	Protein	140183	140049	-	hypothetical protein
contig_6	fig|6666666.28487.peg.4965	Protein	140447	141250	+	Rhamnolipids biosynthesis 3-oxoacyl-[acyl-carrier-protein] reductase RhlG (EC 1.1.1.100)
contig_6	fig|6666666.28487.peg.4966	Protein	141333	142280	+	putative DNA-binding protein

contig_6	fig|6666666.28487.peg.4967	Protein	142643	142254	-	hypothetical protein
contig_6	fig|6666666.28487.rna.38	RNA	142841	142768	-	tRNA-Leu-TAA
contig_6	fig|6666666.28487.peg.4968	Protein	144073	142862	-	Integrase
contig_6	fig|6666666.28487.peg.4969	Protein	144607	146184	+	putative ATP-binding protein
contig_6	fig|6666666.28487.peg.4970	Protein	146455	146315	-	hypothetical protein
contig_6	fig|6666666.28487.peg.4971	Protein	149721	147349	-	FIG235328: hypothetical protein
contig_6	fig|6666666.28487.peg.4972	Protein	149936	149697	-	hypothetical protein
contig_6	fig|6666666.28487.peg.4973	Protein	151674	150802	-	hypothetical protein
contig_6	fig|6666666.28487.peg.4974	Protein	152760	151834	-	hypothetical protein
contig_6	fig|6666666.28487.peg.4975	Protein	153164	154126	+	Cell division protein FtsH (EC 3.4.24.-)
contig_6	fig|6666666.28487.peg.4976	Protein	154851	155213	+	hypothetical protein
contig_6	fig|6666666.28487.peg.4977	Protein	155786	155280	-	pyridoxamine 5'-phosphate oxidase-related, FMN-binding
contig_6	fig|6666666.28487.peg.4978	Protein	157972	156002	-	Lead, cadmium, zinc and mercury transporting ATPase (EC 3.6.3.3) (EC 3.6.3.5); Copper-translocating P-type ATPase (EC 3.6.3.4)
contig_6	fig|6666666.28487.peg.4979	Protein	158519	158190	-	hypothetical protein
contig_6	fig|6666666.28487.peg.4980	Protein	158736	158590	-	YHS domain protein
contig_6	fig|6666666.28487.peg.4981	Protein	158744	158857	+	hypothetical protein
contig_6	fig|6666666.28487.peg.4982	Protein	161147	158895	-	Lead, cadmium, zinc and mercury transporting ATPase (EC 3.6.3.3) (EC 3.6.3.5); Copper-translocating P-type ATPase (EC 3.6.3.4)

contig_6	fig|6666666.28487.peg.4983	Protein	161919	161221	-	hypothetical protein
contig_6	fig|6666666.28487.peg.4984	Protein	161933	162052	+	hypothetical protein
contig_6	fig|6666666.28487.peg.4985	Protein	162404	162189	-	Copper chaperone
contig_6	fig|6666666.28487.peg.4986	Protein	162561	163382	+	Alpha/beta hydrolase fold
contig_6	fig|6666666.28487.peg.4987	Protein	163838	164248	+	Transcriptional regulator, AraC family
contig_6	fig|6666666.28487.peg.4988	Protein	165287	164646	-	quaternary amine transporter
contig_6	fig|6666666.28487.peg.4989	Protein	165815	165426	-	Mercuric resistance operon regulatory protein
contig_6	fig|6666666.28487.peg.4990	Protein	165872	167275	+	Mercuric ion reductase (EC 1.16.1.1)
contig_6	fig|6666666.28487.peg.4991	Protein	167302	167943	+	Organomercurial lyase (EC 4.99.1.2)
contig_6	fig|6666666.28487.peg.4992	Protein	167976	168266	+	hypothetical protein
contig_6	fig|6666666.28487.peg.4993	Protein	168310	168813	+	hypothetical protein
contig_6	fig|6666666.28487.peg.4994	Protein	168810	169658	+	hypothetical protein
contig_6	fig|6666666.28487.peg.4995	Protein	170226	169795	-	hypothetical protein
contig_6	fig|6666666.28487.peg.4996	Protein	170604	171020	+	Sterol desaturase
contig_6	fig|6666666.28487.peg.4997	Protein	171387	172610	+	ErfK/YbiS/YcfS/YnhG family protein
contig_6	fig|6666666.28487.peg.4998	Protein	173162	173836	+	NLP/P60 family protein
contig_6	fig|6666666.28487.peg.4999	Protein	174641	173895	-	Two-component system response regulator
contig_6	fig|6666666.28487.peg.5000	Protein	176017	174752	-	PROBABLE CONSERVED INTEGRAL MEMBRANE TRANSPORT PROTEIN
contig_6	fig|6666666.28487.peg.5001	Protein	177042	176014	-	Cysteine synthase (EC 2.5.1.47)

contig_6	fig|6666666.28487.peg.5002	Protein	177651	177235	-	PROBABLE LIPOPROTEIN LPQS
contig_6	fig|6666666.28487.peg.5003	Protein	178907	177984	-	Peptidase M48, Ste24p precursor
contig_6	fig|6666666.28487.peg.5004	Protein	179266	178904	-	Transcriptional regulator, MecI family
contig_6	fig|6666666.28487.peg.5005	Protein	179609	181012	+	NLP/P60 family protein
contig_6	fig|6666666.28487.peg.5006	Protein	181244	180990	-	hypothetical protein
contig_6	fig|6666666.28487.peg.5007	Protein	181382	182128	+	Two-component system response regulator
contig_6	fig|6666666.28487.peg.5008	Protein	182215	183309	+	Osmosensitive K+ channel histidine kinase KdpD (EC 2.7.3.-)
contig_6	fig|6666666.28487.peg.5009	Protein	183839	183327	-	putative lipoprotein
contig_6	fig|6666666.28487.peg.5010	Protein	184011	184130	+	hypothetical protein
contig_6	fig|6666666.28487.peg.5011	Protein	185205	184552	-	Membrane protein
contig_6	fig|6666666.28487.peg.5012	Protein	185784	185290	-	hypothetical protein
contig_6	fig|6666666.28487.peg.5013	Protein	186865	186287	-	Ubiquinone/menaquinone biosynthesis methyltransferase UbiE (EC 2.1.1.-)
contig_6	fig|6666666.28487.peg.5014	Protein	188642	187011	-	Ccs1/ResB-related putative cytochrome C-type biogenesis protein
contig_6	fig|6666666.28487.peg.5015	Protein	189367	188597	-	Cytochrome c-type biogenesis protein CcdA (DsbD analog)
contig_6	fig|6666666.28487.peg.5016	Protein	189604	190581	+	Copper resistance protein D
contig_6	fig|6666666.28487.peg.5017	Protein	190626	190988	+	regulatory protein, ArsR
contig_6	fig|6666666.28487.peg.5018	Protein	190981	193116	+	Lead, cadmium, zinc and mercury transporting ATPase (EC 3.6.3.3) (EC 3.6.3.5); Copper-translocating P-type ATPase (EC 3.6.3.4)

contig_6	fig|6666666.28487.peg.5019	Protein	193141	194685	+	Spermidine synthase (EC 2.5.1.16)
contig_6	fig|6666666.28487.peg.5020	Protein	195589	194717	-	FIG00825416: hypothetical protein
contig_6	fig|6666666.28487.peg.5021	Protein	196389	195694	-	Adenylate cyclase (EC 4.6.1.1)
contig_6	fig|6666666.28487.peg.5022	Protein	198252	196507	-	Cytochrome c oxidase polypeptide I (EC 1.9.3.1)
contig_6	fig|6666666.28487.peg.5023	Protein	198367	198963	+	Thiol:disulfide oxidoreductase related to ResA
contig_6	fig|6666666.28487.peg.5024	Protein	199464	198934	-	hypothetical protein
contig_6	fig|6666666.28487.peg.5025	Protein	199559	200152	+	Cytochrome c oxidase polypeptide III (EC 1.9.3.1)
contig_6	fig|6666666.28487.peg.5026	Protein	201320	200403	-	Peptidase M48, Ste24p precursor
contig_6	fig|6666666.28487.peg.5027	Protein	201694	201317	-	Transcriptional regulator, MecI family
contig_6	fig|6666666.28487.peg.5028	Protein	201981	202634	+	posibble methylase involved in ubiquinone/menaquinone biosynthesis
contig_6	fig|6666666.28487.peg.5029	Protein	203588	202668	-	Peptidase M48, Ste24p precursor
contig_6	fig|6666666.28487.peg.5030	Protein	203986	203585	-	Transcriptional regulator, MecI family
contig_6	fig|6666666.28487.peg.5031	Protein	204248	204400	+	hypothetical protein
contig_60	fig|6666666.28487.peg.5032	Protein	38	259	+	hypothetical protein
contig_60	fig|6666666.28487.peg.5033	Protein	1324	341	-	hypothetical protein
contig_60	fig|6666666.28487.peg.5034	Protein	1463	2491	+	Membrane protein, putative
contig_60	fig|6666666.28487.peg.5035	Protein	4390	2498	-	FIG00829423: hypothetical protein
contig_60	fig|6666666.28487.peg.5036	Protein	4648	4454	-	hypothetical protein
contig_60	fig|6666666.28487.peg.5037	Protein	4679	5101	+	PROBABLE CONSERVED TRANSMEMBRANE PROTEIN

contig_60	fig|6666666.28487.peg.5038	Protein	5148	5747	+	FIG00822523: hypothetical protein
contig_60	fig|6666666.28487.peg.5039	Protein	6903	5752	-	Isovaleryl-CoA dehydrogenase (EC 1.3.99.10)
contig_60	fig|6666666.28487.peg.5040	Protein	8468	6900	-	Long-chain-fatty-acid--CoA ligase (EC 6.2.1.3)
contig_60	fig|6666666.28487.peg.5041	Protein	8719	8465	-	Acyl carrier protein MbtL
contig_60	fig|6666666.28487.peg.5042	Protein	9797	8760	-	FIG00822056: hypothetical protein
contig_60	fig|6666666.28487.peg.5043	Protein	10222	9848	-	Protein CrcB homolog 2
contig_60	fig|6666666.28487.peg.5044	Protein	10608	10213	-	Protein CrcB homolog 1
contig_60	fig|6666666.28487.peg.5045	Protein	10668	12302	+	Phosphoglucomutase (EC 5.4.2.2)
contig_60	fig|6666666.28487.peg.5046	Protein	12419	13567	+	Multidrug resistance protein B
contig_60	fig|6666666.28487.rna.39	RNA	13621	13693	+	tRNA-Ala-GGC
contig_60	fig|6666666.28487.peg.5047	Protein	14180	14443	+	LSU ribosomal protein L31p
contig_60	fig|6666666.28487.peg.5048	Protein	14872	14615	-	hypothetical protein
contig_60	fig|6666666.28487.peg.5049	Protein	16046	14901	-	Putative metal chaperone, involved in Zn homeostasis, GTPase of COG0523 family
contig_60	fig|6666666.28487.peg.5050	Protein	17268	16141	-	FIG00994788: hypothetical protein
contig_60	fig|6666666.28487.peg.5051	Protein	17401	18048	+	Phosphate transport system regulatory protein PhoU
contig_60	fig|6666666.28487.peg.5052	Protein	20434	18059	-	Pyruvate-utilizing enzyme, similar to phosphoenolpyruvate synthase
contig_60	fig|6666666.28487.peg.5053	Protein	21567	20452	-	hypothetical protein
contig_60	fig|6666666.28487.peg.5054	Protein	21616	22218	+	Transcriptional regulator, TetR family

contig_61	fig|6666666.28487.peg.5055	Protein	1551	136	-	Dihydrolipoamide dehydrogenase (EC 1.8.1.4)
contig_61	fig|6666666.28487.peg.5056	Protein	1653	2096	+	FIG01122412: hypothetical protein
contig_61	fig|6666666.28487.peg.5057	Protein	3068	2181	-	FIG00823482: hypothetical protein
contig_61	fig|6666666.28487.peg.5058	Protein	6062	3201	-	Putative membrane protein mmpL5
contig_61	fig|6666666.28487.peg.5059	Protein	6295	6873	+	Transcriptional regulator, TetR family
contig_61	fig|6666666.28487.peg.5060	Protein	8147	6954	-	Possible N-acyl-L-amino acid amidohydrolase amiA1 (EC 3.5.1.-)
contig_61	fig|6666666.28487.peg.5061	Protein	10066	8126	-	Catalyzes the cleavage of p-aminobenzoyl-glutamate to p-aminobenzoate and glutamate, subunit A
contig_61	fig|6666666.28487.peg.5062	Protein	11214	10090	-	Putative transport protein
contig_61	fig|6666666.28487.peg.5063	Protein	11305	11754	+	Transcriptional regulator, MarR family
contig_61	fig|6666666.28487.peg.5064	Protein	11759	13414	+	Probable phosphomannomutase pmmB (EC 5.4.2.8)
contig_61	fig|6666666.28487.peg.5065	Protein	13987	14289	+	hypothetical protein
contig_61	fig|6666666.28487.peg.5066	Protein	14289	15377	+	FIG00831455: hypothetical protein
contig_61	fig|6666666.28487.peg.5067	Protein	16246	15374	-	luciferase family protein
contig_61	fig|6666666.28487.peg.5068	Protein	16390	17640	+	FIG026501: hypothetical protein
contig_61	fig|6666666.28487.peg.5069	Protein	18888	17800	-	Adenosine deaminase (EC 3.5.4.4)
contig_61	fig|6666666.28487.peg.5070	Protein	20217	18898	-	Thymidine phosphorylase (EC 2.4.2.4)
contig_61	fig|6666666.28487.peg.5071	Protein	20636	20214	-	Cytidine deaminase (EC 3.5.4.5)
contig_61	fig|6666666.28487.peg.5072	Protein	20884	21282	+	Succinate dehydrogenase cytochrome b-556 subunit
contig_61	fig|6666666.28487.peg.5073	Protein	21283	21756	+	Succinate dehydrogenase hydrophobic membrane anchor

						protein
contig_61	fig|6666666.28487.peg.5074	Protein	21771	23525	+	Succinate dehydrogenase flavoprotein subunit (EC 1.3.99.1)
contig_61	fig|6666666.28487.peg.5075	Protein	23566	24348	+	Succinate dehydrogenase iron-sulfur protein (EC 1.3.99.1)
contig_61	fig|6666666.28487.peg.5076	Protein	24350	25258	+	putative lipase/esterase
contig_61	fig|6666666.28487.peg.5077	Protein	26121	25255	-	putative hydrolase
contig_61	fig|6666666.28487.peg.5078	Protein	26208	26711	+	protein of unknown function DUF1470
contig_61	fig|6666666.28487.peg.5079	Protein	27882	26716	-	putative integral membrane protein
contig_61	fig|6666666.28487.peg.5080	Protein	27989	28804	+	short-chain dehydrogenase/reductase SDR
contig_61	fig|6666666.28487.peg.5081	Protein	28934	29314	+	hypothetical protein
contig_61	fig|6666666.28487.peg.5082	Protein	29431	29946	+	FIG00824105: hypothetical protein
contig_61	fig|6666666.28487.peg.5083	Protein	29990	30580	+	Transcriptional regulator, TetR family
contig_61	fig|6666666.28487.peg.5084	Protein	31537	30551	-	Dihydroflavonol-4-reductase (EC 1.1.1.219)
contig_61	fig|6666666.28487.peg.5085	Protein	31573	32742	+	NADH:flavin oxidoreductases, Old Yellow Enzyme family
contig_61	fig|6666666.28487.peg.5086	Protein	33624	32728	-	putative RNA polymerase sigma factor
contig_61	fig|6666666.28487.peg.5087	Protein	33748	35259	+	Amino acid permease
contig_61	fig|6666666.28487.peg.5088	Protein	35275	36138	+	Universal stress protein family
contig_61	fig|6666666.28487.peg.5089	Protein	37713	36238	-	Betaine aldehyde dehydrogenase (EC 1.2.1.8)
contig_61	fig|6666666.28487.peg.5090	Protein	37837	38358	+	Transcriptional regulator, AsnC family
contig_61	fig|6666666.28487.peg.5091	Protein	38444	38313	-	hypothetical protein

contig_61	fig|6666666.28487.peg.5092	Protein	38397	39743	+	Omega-amino acid--pyruvate aminotransferase (EC 2.6.1.18)
contig_61	fig|6666666.28487.peg.5093	Protein	39837	40835	+	Strictosidine synthase precursor (EC 4.3.3.2)
contig_61	fig|6666666.28487.peg.5094	Protein	41002	41301	+	hypothetical protein
contig_61	fig|6666666.28487.peg.5095	Protein	42241	41414	-	Putative transcriptional regulator
contig_61	fig|6666666.28487.peg.5096	Protein	42303	43991	+	Acetyl-coenzyme A synthetase (EC 6.2.1.1)
contig_61	fig|6666666.28487.peg.5097	Protein	43988	45604	+	Acetoacetyl-CoA synthetase (EC 6.2.1.16) / Long-chain-fatty-acid--CoA ligase (EC 6.2.1.3)
contig_61	fig|6666666.28487.peg.5098	Protein	45804	45652	-	hypothetical protein
contig_61	fig|6666666.28487.peg.5099	Protein	47127	45940	-	putative integral membrane protein
contig_61	fig|6666666.28487.peg.5100	Protein	47350	48576	+	D-alanyl-D-alanine carboxypeptidase (EC 3.4.16.4)
contig_61	fig|6666666.28487.peg.5101	Protein	49613	48573	-	putative membrane protein
contig_61	fig|6666666.28487.peg.5102	Protein	50643	49630	-	Tryptophanyl-tRNA synthetase (EC 6.1.1.2)
contig_61	fig|6666666.28487.peg.5103	Protein	51096	50677	-	Transcriptional regulator, MarR family
contig_61	fig|6666666.28487.peg.5104	Protein	51086	51916	+	FIG00823580: hypothetical protein
contig_61	fig|6666666.28487.peg.5105	Protein	52795	51983	-	Exodeoxyribonuclease III (EC 3.1.11.2)
contig_61	fig|6666666.28487.peg.5106	Protein	52866	53699	+	Alpha/beta hydrolase family
contig_61	fig|6666666.28487.peg.5107	Protein	53915	55255	+	O-acetylhomoserine sulfhydrylase (EC 2.5.1.49) / O-succinylhomoserine sulfhydrylase (EC 2.5.1.48)
contig_61	fig|6666666.28487.peg.5108	Protein	55268	56395	+	Homoserine O-acetyltransferase (EC 2.3.1.31)
contig_61	fig|6666666.28487.peg.5109	Protein	56392	57123	+	Methyltransferase (EC 2.1.1.-)

contig_61	fig|6666666.28487.peg.5110	Protein	57513	57229	-	hypothetical protein
contig_61	fig|6666666.28487.peg.5111	Protein	58361	57510	-	Methylenetetrahydrofolate dehydrogenase (NADP+) (EC 1.5.1.5) / Methenyltetrahydrofolate cyclohydrolase (EC 3.5.4.9)
contig_61	fig|6666666.28487.peg.5112	Protein	58389	59594	+	NADH:flavin oxidoreductases, Old Yellow Enzyme family
contig_61	fig|6666666.28487.peg.5113	Protein	60141	59578	-	Transcriptional regulator, TetR family
contig_61	fig|6666666.28487.peg.5114	Protein	60190	60585	+	conserved hypothetical protein
contig_61	fig|6666666.28487.peg.5115	Protein	60640	63258	+	Probable conserved transmembrane ATP-binding protein ABC transporter
contig_61	fig|6666666.28487.peg.5116	Protein	63401	63964	+	Transcriptional regulator, TetR family
contig_61	fig|6666666.28487.peg.5117	Protein	64519	63968	-	pentapeptide repeat family protein
contig_61	fig|6666666.28487.peg.5118	Protein	65093	64524	-	Putative ATP/GTP-binding protein
contig_61	fig|6666666.28487.peg.5119	Protein	65451	65074	-	FIG00995222: hypothetical protein
contig_61	fig|6666666.28487.peg.5120	Protein	65878	65459	-	FIG01121836: hypothetical protein
contig_61	fig|6666666.28487.peg.5121	Protein	68424	65875	-	putative sensor-like histidine kinase
contig_61	fig|6666666.28487.peg.5122	Protein	68775	68927	+	FIG00827895: hypothetical protein
contig_61	fig|6666666.28487.peg.5123	Protein	68969	69424	+	tRNA (cytosine34-2'-O-)-methyltransferase (EC 2.1.1.-)
contig_62	fig|6666666.28487.peg.5124	Protein	733	56	-	Enoyl-CoA hydratase (EC 4.2.1.17)
contig_62	fig|6666666.28487.peg.5125	Protein	1560	730	-	FIG00996530: hypothetical protein
contig_63	fig|6666666.28487.peg.5126	Protein	48	326	+	hypothetical protein
contig_63	fig|6666666.28487.peg.5127	Protein	837	649	-	hypothetical protein

contig_63	fig|6666666.28487.peg.5128	Protein	2577	1210	-	hypothetical protein
contig_63	fig|6666666.28487.peg.5129	Protein	3043	3987	+	hypothetical protein
contig_63	fig|6666666.28487.peg.5130	Protein	4159	10497	+	FIG00832862: hypothetical protein
contig_63	fig|6666666.28487.peg.5131	Protein	10552	10938	+	hypothetical protein
contig_63	fig|6666666.28487.peg.5132	Protein	10951	11508	+	hypothetical protein
contig_63	fig|6666666.28487.peg.5133	Protein	11803	11573	-	hypothetical protein
contig_63	fig|6666666.28487.peg.5134	Protein	11792	12316	+	Unknown protein
contig_63	fig|6666666.28487.peg.5135	Protein	12502	14553	+	hypothetical protein
contig_63	fig|6666666.28487.peg.5136	Protein	17652	14803	-	ATP-dependent RNA helicase
contig_63	fig|6666666.28487.peg.5137	Protein	17764	18702	+	hypothetical protein
contig_63	fig|6666666.28487.peg.5138	Protein	18706	21459	+	Exonuclease SbcC
contig_63	fig|6666666.28487.peg.5139	Protein	22036	22182	+	hypothetical protein
contig_63	fig|6666666.28487.peg.5140	Protein	23027	22425	-	hypothetical protein
contig_63	fig|6666666.28487.peg.5141	Protein	23217	23381	+	hypothetical protein
contig_63	fig|6666666.28487.peg.5142	Protein	24185	23541	-	hypothetical protein
contig_63	fig|6666666.28487.peg.5143	Protein	24589	24209	-	hypothetical protein
contig_63	fig|6666666.28487.peg.5144	Protein	25348	24647	-	Endonuclease/exonuclease/phosphatase
contig_63	fig|6666666.28487.peg.5145	Protein	25625	27286	+	Conserved domain protein
contig_63	fig|6666666.28487.peg.5146	Protein	28331	27327	-	hypothetical protein

contig_63	fig|6666666.28487.peg.5147	Protein	29806	28373	-	ADP-ribosylglycohydrolase
contig_63	fig|6666666.28487.peg.5148	Protein	29930	30580	+	Hypothetical Nudix-like regulator
contig_63	fig|6666666.28487.peg.5149	Protein	30901	30653	-	hypothetical protein
contig_63	fig|6666666.28487.peg.5150	Protein	31343	31176	-	hypothetical protein
contig_63	fig|6666666.28487.peg.5151	Protein	31602	32462	+	hypothetical protein
contig_63	fig|6666666.28487.peg.5152	Protein	33401	32457	-	hypothetical protein
contig_63	fig|6666666.28487.peg.5153	Protein	34489	33506	-	Bifunctional protein: zinc-containing alcohol dehydrogenase; quinone oxidoreductase (NADPH:quinone reductase) (EC 1.1.1.-); Similar to arginate lyase
contig_63	fig|6666666.28487.peg.5154	Protein	34635	35306	+	hypothetical protein
contig_63	fig|6666666.28487.peg.5155	Protein	35665	35339	-	hypothetical protein
contig_63	fig|6666666.28487.peg.5156	Protein	36060	35863	-	hypothetical protein possibly connected to lactam utilization and allophanate hydrolase
contig_63	fig|6666666.28487.peg.5157	Protein	36650	36015	-	hypothetical protein possibly connected to lactam utilization and allophanate hydrolase
contig_63	fig|6666666.28487.peg.5158	Protein	37957	36716	-	hypothetical protein
contig_63	fig|6666666.28487.peg.5159	Protein	37933	38112	+	hypothetical protein
contig_63	fig|6666666.28487.peg.5160	Protein	38228	39016	+	FIG00823058: hypothetical protein
contig_63	fig|6666666.28487.peg.5161	Protein	39013	39711	+	FIG00833620: hypothetical protein
contig_63	fig|6666666.28487.peg.5162	Protein	40072	39725	-	hypothetical protein
contig_63	fig|6666666.28487.peg.5163	Protein	40521	40270	-	hypothetical protein

contig_63	fig|6666666.28487.peg.5164	Protein	40861	40487	-	hypothetical protein
contig_63	fig|6666666.28487.peg.5165	Protein	41117	40959	-	hypothetical protein
contig_63	fig|6666666.28487.peg.5166	Protein	41459	41166	-	hypothetical protein
contig_63	fig|6666666.28487.peg.5167	Protein	41540	41659	+	hypothetical protein
contig_63	fig|6666666.28487.peg.5168	Protein	41819	42928	+	POSSIBLE LINOLEOYL-CoA DESATURASE (DELTA(6)-DESATURASE)
contig_63	fig|6666666.28487.peg.5169	Protein	43017	44246	+	Transcriptional regulator, IclR family
contig_63	fig|6666666.28487.peg.5170	Protein	44384	46102	+	Vancomycin B-type resistance protein VanW
contig_63	fig|6666666.28487.peg.5171	Protein	46595	46882	+	hypothetical protein
contig_63	fig|6666666.28487.peg.5172	Protein	48833	47553	-	hypothetical protein
contig_64	fig|6666666.28487.peg.5173	Protein	2404	41	-	FUPA25 P-type ATPase
contig_64	fig|6666666.28487.peg.5174	Protein	3058	2417	-	putative lipoprotein
contig_64	fig|6666666.28487.peg.5175	Protein	3970	3089	-	Universal stress protein family
contig_64	fig|6666666.28487.peg.5176	Protein	4785	4000	-	Universal stress protein family
contig_64	fig|6666666.28487.peg.5177	Protein	7158	4870	-	Phosphoenolpyruvate synthase (EC 2.7.9.2)
contig_64	fig|6666666.28487.peg.5178	Protein	7341	8309	+	Trehalose-6-phosphate phosphatase (EC 3.1.3.12)
contig_64	fig|6666666.28487.peg.5179	Protein	8306	10813	+	Trehalose-6-phosphate phosphatase (EC 3.1.3.12)
contig_64	fig|6666666.28487.peg.5180	Protein	12057	10879	-	hypothetical protein
contig_64	fig|6666666.28487.peg.5181	Protein	12128	12595	+	Transcriptional regulator, MarR family
contig_64	fig|6666666.28487.peg.5182	Protein	12573	14177	+	Mobile element protein

contig_64	fig|6666666.28487.peg.5183	Protein	14202	14711	+	Mobile element protein
contig_64	fig|6666666.28487.peg.5184	Protein	14718	14993	+	Mobile element protein
contig_64	fig|6666666.28487.peg.5185	Protein	15178	15041	-	hypothetical protein
contig_64	fig|6666666.28487.peg.5186	Protein	16217	15303	-	Carbamate kinase (EC 2.7.2.2)
contig_64	fig|6666666.28487.peg.5187	Protein	17231	16227	-	Ornithine carbamoyltransferase (EC 2.1.3.3)
contig_64	fig|6666666.28487.peg.5188	Protein	18485	17253	-	Arginine deiminase (EC 3.5.3.6)
contig_64	fig|6666666.28487.peg.5189	Protein	20023	18506	-	Arginine/ornithine antiporter ArcD
contig_64	fig|6666666.28487.peg.5190	Protein	20173	23043	+	ATPase, P-type (transporting), HAD superfamily, subfamily IC
contig_64	fig|6666666.28487.peg.5191	Protein	23139	23600	+	hypothetical protein
contig_64	fig|6666666.28487.peg.5192	Protein	23656	24024	+	hypothetical protein
contig_64	fig|6666666.28487.peg.5193	Protein	24112	24561	+	FIG00825439: hypothetical protein
contig_64	fig|6666666.28487.peg.5194	Protein	24587	26632	+	Protein-L-isoaspartate O-methyltransferase (EC 2.1.1.77)
contig_64	fig|6666666.28487.peg.5195	Protein	26629	27591	+	Tagatose-6-phosphate kinase (EC 2.7.1.144) / 1-phosphofructokinase (EC 2.7.1.56)
contig_64	fig|6666666.28487.peg.5196	Protein	27642	29357	+	Histidine kinase response regulator
contig_64	fig|6666666.28487.peg.5197	Protein	30005	29370	-	Transcriptional regulator, TetR family
contig_64	fig|6666666.28487.peg.5198	Protein	30333	30073	-	hypothetical protein
contig_64	fig|6666666.28487.peg.5199	Protein	31146	30535	-	FIG00832151: hypothetical protein
contig_64	fig|6666666.28487.peg.5200	Protein	31284	32303	+	Alcohol dehydrogenase zinc-binding domain protein
contig_64	fig|6666666.28487.peg.5201	Protein	32585	32328	-	hypothetical protein

contig_64	fig|6666666.28487.peg.5202	Protein	34002	32704	-	Phosphoribosyl transferase domain protein
contig_64	fig|6666666.28487.peg.5203	Protein	34811	34068	-	hypothetical protein
contig_64	fig|6666666.28487.peg.5204	Protein	35557	34808	-	hypothetical protein
contig_64	fig|6666666.28487.peg.5205	Protein	36066	35650	-	hypothetical protein
contig_64	fig|6666666.28487.peg.5206	Protein	36324	37211	+	Universal stress protein family
contig_64	fig|6666666.28487.peg.5207	Protein	37438	37208	-	hypothetical protein
contig_64	fig|6666666.28487.peg.5208	Protein	38669	37518	-	hypothetical protein
contig_64	fig|6666666.28487.peg.5209	Protein	39114	38659	-	hypothetical protein
contig_64	fig|6666666.28487.peg.5210	Protein	39277	39543	+	FIG00824345: hypothetical protein
contig_64	fig|6666666.28487.peg.5211	Protein	39540	41339	+	Acetyl-coenzyme A synthetase (EC 6.2.1.1)
contig_64	fig|6666666.28487.peg.5212	Protein	41336	42313	+	Acetoin dehydrogenase E1 component alpha-subunit (EC 1.2.4.-)
contig_64	fig|6666666.28487.peg.5213	Protein	42310	43287	+	Acetoin dehydrogenase E1 component beta-subunit (EC 1.2.4.-)
contig_64	fig|6666666.28487.peg.5214	Protein	43284	44486	+	Dihydrolipoamide acetyltransferase component (E2) of acetoin dehydrogenase complex (EC 2.3.1.-)
contig_64	fig|6666666.28487.peg.5215	Protein	44492	44746	+	Acyl carrier protein
contig_64	fig|6666666.28487.peg.5216	Protein	44961	45377	+	hypothetical protein
contig_64	fig|6666666.28487.peg.5217	Protein	45463	45741	+	conserved hypothetical protein
contig_64	fig|6666666.28487.peg.5218	Protein	45929	46909	+	Universal stress protein family
contig_64	fig|6666666.28487.peg.5219	Protein	46906	47502	+	hypothetical protein

contig_64	fig|6666666.28487.peg.5220	Protein	48048	47518	-	Hydrolases of the alpha/beta superfamily
contig_66	fig|6666666.28487.peg.5221	Protein	394	29	-	hypothetical protein
contig_66	fig|6666666.28487.peg.5222	Protein	700	419	-	hypothetical protein
contig_66	fig|6666666.28487.peg.5223	Protein	1035	757	-	membrane protein-like protein
contig_66	fig|6666666.28487.peg.5224	Protein	1380	1144	-	hypothetical protein
contig_66	fig|6666666.28487.peg.5225	Protein	2054	1485	-	hypothetical protein
contig_66	fig|6666666.28487.peg.5226	Protein	2562	2059	-	hypothetical protein
contig_66	fig|6666666.28487.peg.5227	Protein	3056	2679	-	hypothetical protein
contig_66	fig|6666666.28487.peg.5228	Protein	3228	3049	-	conserved hypothetical protein
contig_66	fig|6666666.28487.peg.5229	Protein	3552	3280	-	hypothetical protein
contig_66	fig|6666666.28487.peg.5230	Protein	3857	3549	-	hypothetical protein
contig_66	fig|6666666.28487.peg.5231	Protein	4337	3858	-	Alkaline shock protein 23
contig_66	fig|6666666.28487.peg.5232	Protein	4548	5135	+	RNA polymerase sigma-70 factor, ECF subfamily
contig_66	fig|6666666.28487.peg.5233	Protein	5123	5602	+	hypothetical protein
contig_66	fig|6666666.28487.peg.5234	Protein	5767	6615	+	CmpX
contig_66	fig|6666666.28487.peg.5235	Protein	6737	6880	+	hypothetical protein
contig_66	fig|6666666.28487.peg.5236	Protein	7369	7488	+	hypothetical protein
contig_66	fig|6666666.28487.peg.5237	Protein	8725	7619	-	Alpha-methylacyl-CoA racemase (EC 5.1.99.4)
contig_66	fig|6666666.28487.peg.5238	Protein	8999	8730	-	Butyryl-CoA dehydrogenase (EC 1.3.99.2)

contig_66	fig|6666666.28487.peg.5239	Protein	9864	9481	-	hypothetical protein
contig_66	fig|6666666.28487.peg.5240	Protein	12790	9881	-	FIG00820985: hypothetical protein
contig_66	fig|6666666.28487.peg.5241	Protein	14260	13175	-	possible Rep protein
contig_66	fig|6666666.28487.peg.5242	Protein	14816	14607	-	hypothetical protein
contig_66	fig|6666666.28487.peg.5243	Protein	15271	14924	-	hypothetical protein
contig_66	fig|6666666.28487.peg.5244	Protein	15665	15345	-	SSU ribosomal protein S7p (S5e), mitochondrial
contig_66	fig|6666666.28487.peg.5245	Protein	15964	15677	-	hypothetical protein
contig_66	fig|6666666.28487.peg.5246	Protein	16048	16275	+	hypothetical protein
contig_66	fig|6666666.28487.peg.5247	Protein	16955	16551	-	hypothetical protein
contig_66	fig|6666666.28487.peg.5248	Protein	17758	16952	-	Chromosome (plasmid) partitioning protein ParA
contig_66	fig|6666666.28487.peg.5249	Protein	18187	18044	-	hypothetical protein
contig_66	fig|6666666.28487.peg.5250	Protein	18579	18746	+	hypothetical protein
contig_66	fig|6666666.28487.peg.5251	Protein	18743	21298	+	hypothetical protein
contig_66	fig|6666666.28487.peg.5252	Protein	21475	21323	-	hypothetical protein
contig_66	fig|6666666.28487.peg.5253	Protein	21541	21885	+	Mobile element protein
contig_66	fig|6666666.28487.peg.5254	Protein	23252	22155	-	Threonine synthase (EC 4.2.3.1)
contig_66	fig|6666666.28487.peg.5255	Protein	23883	23365	-	hypothetical protein
contig_66	fig|6666666.28487.peg.5256	Protein	24451	23909	-	Anti-sigma F factor antagonist (spoIIAA-2); Anti-sigma B factor antagonist RsbV
contig_66	fig|6666666.28487.peg.5257	Protein	24778	25500	+	Transcriptional regulator, TetR family

contig_66	fig|6666666.28487.peg.5258	Protein	25617	26717	+	hypothetical protein
contig_66	fig|6666666.28487.peg.5259	Protein	28326	27109	-	hypothetical protein
contig_66	fig|6666666.28487.peg.5260	Protein	29000	28665	-	HigA protein (antitoxin to HigB)
contig_66	fig|6666666.28487.peg.5261	Protein	29341	29060	-	HigB toxin protein
contig_67	fig|6666666.28487.peg.5262	Protein	41	667	+	PROBABLE CONSERVED INTEGRAL MEMBRANE ALANINE AND LEUCINE RICH PROTEIN
contig_67	fig|6666666.28487.peg.5263	Protein	1260	664	-	Trp repressor binding protein
contig_67	fig|6666666.28487.peg.5264	Protein	2723	1293	-	Phytoene dehydrogenase and related proteins
contig_67	fig|6666666.28487.peg.5265	Protein	2756	4342	+	D-3-phosphoglycerate dehydrogenase (EC 1.1.1.95)
contig_67	fig|6666666.28487.peg.5266	Protein	4339	5349	+	3-isopropylmalate dehydrogenase (EC 1.1.1.85)
contig_67	fig|6666666.28487.peg.5267	Protein	5388	5951	+	Possible NADH-Ubiquinone/plastoquinone
contig_67	fig|6666666.28487.peg.5268	Protein	7159	5948	-	Integral membrane transport protein
contig_67	fig|6666666.28487.peg.5269	Protein	7278	8066	+	2-hydroxyhepta-2,4-diene-1,7-dioate isomerase (EC 5.3.3.-) / 5-carboxymethyl-2-oxo-hex-3- ene-1,7-dioate decarboxylase (EC 4.1.1.68)
contig_67	fig|6666666.28487.peg.5270	Protein	8078	9547	+	Glutamyl-tRNA synthetase (EC 6.1.1.17) @ Glutamyl-tRNA(Gln) synthetase (EC 6.1.1.24)
contig_67	fig|6666666.28487.rna.40	RNA	9691	9762	+	tRNA-Gln-CTG
contig_67	fig|6666666.28487.rna.41	RNA	9828	9900	+	tRNA-Glu-CTC
contig_67	fig|6666666.28487.peg.5271	Protein	10534	9980	-	Transcriptional regulator, IclR family
contig_67	fig|6666666.28487.peg.5272	Protein	10720	12162	+	3-isopropylmalate dehydratase large subunit (EC 4.2.1.33)

contig_67	fig|6666666.28487.peg.5273	Protein	12184	12777	+	3-isopropylmalate dehydratase small subunit (EC 4.2.1.33)
contig_67	fig|6666666.28487.peg.5274	Protein	12979	13632	+	DNA-binding protein HU / low-complexity, AKP-rich domain
contig_67	fig|6666666.28487.peg.5275	Protein	14622	13720	-	Possible hydrolase mutT1 (EC 3.-.-.-)
contig_67	fig|6666666.28487.peg.5276	Protein	16908	14728	-	Polyphosphate kinase (EC 2.7.4.1)
contig_67	fig|6666666.28487.peg.5277	Protein	17628	16972	-	2-phospho-L-lactate guanylyltransferase (EC 2.7.7.68)
contig_67	fig|6666666.28487.peg.5278	Protein	17673	18668	+	Glycerol-3-phosphate dehydrogenase [NAD(P)+] (EC 1.1.1.94)
contig_67	fig|6666666.28487.peg.5279	Protein	18672	19781	+	Cys/Met metabolism pyridoxal-phosphate-dependent enzymes
contig_67	fig|6666666.28487.peg.5280	Protein	19811	20914	+	D-alanine--D-alanine ligase (EC 6.3.2.4)
contig_67	fig|6666666.28487.peg.5281	Protein	21559	20978	-	Possible membrane protein
contig_67	fig|6666666.28487.peg.5282	Protein	21888	22844	+	Thiamine-monophosphate kinase (EC 2.7.4.16)
contig_67	fig|6666666.28487.peg.5283	Protein	22873	23550	+	Uracil-DNA glycosylase, family 1
contig_67	fig|6666666.28487.peg.5284	Protein	23757	23563	-	LSU ribosomal protein L28p
contig_67	fig|6666666.28487.peg.5285	Protein	23965	25626	+	Dihydroxyacetone kinase family protein
contig_67	fig|6666666.28487.peg.5286	Protein	25628	27889	+	ATP-dependent DNA helicase RecG (EC 3.6.1.-)
contig_67	fig|6666666.28487.peg.5287	Protein	27886	28593	+	hypothetical protein
contig_67	fig|6666666.28487.peg.5288	Protein	29192	29326	+	hypothetical protein
contig_67	fig|6666666.28487.peg.5289	Protein	29900	29667	-	Glycine-rich cell wall structural protein 1.8 precursor
contig_67	fig|6666666.28487.peg.5290	Protein	31083	29908	-	Putative Dyp-type peroxidase, associated with bacterial analog of Cox17 protein

contig_67	fig|6666666.28487.peg.5291	Protein	31507	31097	-	Copper metallochaperone, bacterial analog of Cox17 protein
contig_67	fig|6666666.28487.peg.5292	Protein	33011	31590	-	Uncharacterized iron-regulated membrane protein; Iron-uptake factor PiuB
contig_67	fig|6666666.28487.peg.5293	Protein	33681	33145	-	FIG00830271: hypothetical protein
contig_67	fig|6666666.28487.peg.5294	Protein	34568	33729	-	oxidoreductase of aldo/keto reductase family, subgroup 1
contig_67	fig|6666666.28487.peg.5295	Protein	34716	35831	+	lipase-esterase (lipN)
contig_67	fig|6666666.28487.peg.5296	Protein	35864	36637	+	POSSIBLE CONSERVED MEMBRANE OR SECRETED PROTEIN
contig_67	fig|6666666.28487.peg.5297	Protein	36634	37269	+	Putative ESX-1 secretion system component Rv3877
contig_67	fig|6666666.28487.peg.5298	Protein	37338	40775	+	Pyruvate carboxyl transferase (EC 6.4.1.1)
contig_67	fig|6666666.28487.peg.5299	Protein	40865	41341	+	16S rRNA (guanine(966)-N(2))-methyltransferase (EC 2.1.1.171) ## SSU rRNA m(2)G966
contig_67	fig|6666666.28487.peg.5300	Protein	41395	42777	+	putative cytochrome P450
contig_67	fig|6666666.28487.peg.5301	Protein	42845	43291	+	Phosphopantetheine adenylyltransferase (EC 2.7.7.3)
contig_67	fig|6666666.28487.peg.5302	Protein	43429	44166	+	Cell division initiation protein
contig_67	fig|6666666.28487.peg.5303	Protein	44283	44849	+	COG1399 protein, clustered with ribosomal protein L32p
contig_67	fig|6666666.28487.peg.5304	Protein	44846	45547	+	Ribonuclease III (EC 3.1.26.3)
contig_67	fig|6666666.28487.peg.5305	Protein	45540	46415	+	Formamidopyrimidine-DNA glycosylase (EC 3.2.2.23)
contig_67	fig|6666666.28487.peg.5306	Protein	46499	46924	+	FIG00997866: hypothetical protein
contig_67	fig|6666666.28487.peg.5307	Protein	46908	47192	+	Acylphosphate phosphohydrolase (EC 3.6.1.7), putative
contig_67	fig|6666666.28487.peg.5308	Protein	47219	50803	+	Chromosome partition protein smc

contig_67	fig|6666666.28487.peg.5309	Protein	51893	50889	-	Isopentenyl-diphosphate delta-isomerase, FMN-dependent (EC 5.3.3.2)
contig_67	fig|6666666.28487.peg.5310	Protein	51943	53373	+	Signal recognition particle receptor protein FtsY (=alpha subunit) (TC 3.A.5.1.1)
contig_67	fig|6666666.28487.peg.5311	Protein	53560	54933	+	Ammonium transporter
contig_67	fig|6666666.28487.peg.5312	Protein	54969	55307	+	Nitrogen regulatory protein P-II
contig_67	fig|6666666.28487.peg.5313	Protein	55389	57860	+	[Protein-PII] uridylyltransferase (EC 2.7.7.59)
contig_68	fig|6666666.28487.peg.5314	Protein	1158	40	-	Magnesium and cobalt transport protein CorA
contig_68	fig|6666666.28487.peg.5315	Protein	1303	2472	+	NADP-dependent malic enzyme (EC 1.1.1.40)
contig_68	fig|6666666.28487.peg.5316	Protein	2703	3326	+	Substrate-binding region of ABC-type glycine betaine transport system
contig_68	fig|6666666.28487.peg.5317	Protein	4245	3415	-	Short-chain type dehydrogenase/reductase
contig_68	fig|6666666.28487.peg.5318	Protein	5999	4389	-	PE-PGRS FAMILY PROTEIN
contig_68	fig|6666666.28487.peg.5319	Protein	6173	6526	+	Lipoprotein LprJ
contig_68	fig|6666666.28487.peg.5320	Protein	10393	6620	-	Dihydrolipoamide succinyltransferase component (E2) of 2-oxoglutarate dehydrogenase complex (EC 2.3.1.61) / 2-oxoglutarate dehydrogenase E1 component (EC 1.2.4.2)
contig_68	fig|6666666.28487.peg.5321	Protein	11360	10617	-	POSSIBLE MEMBRANE PROTEIN
contig_68	fig|6666666.28487.peg.5322	Protein	11520	12827	+	drug resistance transporter, Bcr-CflA family protein
contig_68	fig|6666666.28487.peg.5323	Protein	12927	14768	+	Multidrug resistance protein B
contig_68	fig|6666666.28487.peg.5324	Protein	16156	14765	-	hypothetical protein

contig_68	fig|6666666.28487.peg.5325	Protein	17533	16175	-	(S)-2-hydroxy-acid oxidase chain D (EC 1.1.3.15)
contig_68	fig|6666666.28487.peg.5326	Protein	20958	17530	-	Superfamily I DNA and RNA helicases and helicase subunits
contig_68	fig|6666666.28487.peg.5327	Protein	21635	21075	-	Putative lipoprotein lprE precursor
contig_68	fig|6666666.28487.peg.5328	Protein	21799	21632	-	hypothetical protein
contig_68	fig|6666666.28487.peg.5329	Protein	21806	23425	+	Cold-shock DEAD-box protein A
contig_68	fig|6666666.28487.peg.5330	Protein	23422	24573	+	Acyltransferase
contig_68	fig|6666666.28487.peg.5331	Protein	24644	25360	+	Uncharacterized ABC transporter ATP-binding protein Rv1348/MT1390
contig_68	fig|6666666.28487.peg.5332	Protein	25965	25345	-	Possible oxidoreductase subunit
contig_68	fig|6666666.28487.peg.5333	Protein	27206	25965	-	Cytochrome P450 130
contig_68	fig|6666666.28487.peg.5334	Protein	29665	27332	-	Pyruvate dehydrogenase E1 component (EC 1.2.4.1)
contig_68	fig|6666666.28487.peg.5335	Protein	31443	29662	-	polyketide oxidase/hydroxylase
contig_68	fig|6666666.28487.peg.5336	Protein	32154	31465	-	Biphenyl-2,3-diol 1,2-dioxygenase 3
contig_68	fig|6666666.28487.peg.5337	Protein	33122	32169	-	Fumarylacetoacetate hydrolase family protein
contig_68	fig|6666666.28487.peg.5338	Protein	34555	33233	-	hypothetical protein
contig_68	fig|6666666.28487.peg.5339	Protein	35840	34548	-	putative cytochrome P450 hydroxylase
contig_68	fig|6666666.28487.peg.5340	Protein	36646	35927	-	hypothetical protein
contig_68	fig|6666666.28487.peg.5341	Protein	36769	37563	+	short-chain dehydrogenase/reductase SDR
contig_68	fig|6666666.28487.peg.5342	Protein	37620	38609	+	NmrA family protein
contig_68	fig|6666666.28487.peg.5343	Protein	39216	38632	-	3-oxoacyl-[acyl-carrier protein] reductase (EC 1.1.1.100)

contig_68	fig|6666666.28487.peg.5344	Protein	39384	42143	+	regulatory protein, LuxR
contig_68	fig|6666666.28487.peg.5345	Protein	43630	42140	-	sensor histidine kinase
contig_68	fig|6666666.28487.peg.5346	Protein	44282	43632	-	hypothetical protein
contig_68	fig|6666666.28487.peg.5347	Protein	45015	44293	-	3-oxoacyl-[acyl-carrier-protein] reductase(EC:1.1.1.100)
contig_68	fig|6666666.28487.peg.5348	Protein	45752	45012	-	conserved secreted protein
contig_68	fig|6666666.28487.peg.5349	Protein	46162	45749	-	Cupin 2, conserved barrel domain protein
contig_68	fig|6666666.28487.peg.5350	Protein	47321	46155	-	NADH dehydrogenase (EC 1.6.99.3)
contig_68	fig|6666666.28487.peg.5351	Protein	47493	48440	+	Esterase/lipase
contig_68	fig|6666666.28487.peg.5352	Protein	48498	49508	+	NADPH-dependent methylglyoxal reductase (D-lactaldehyde dehydrogenase)
contig_68	fig|6666666.28487.peg.5353	Protein	50875	49514	-	"Glycolate oxidase, subunit GlcD"
contig_68	fig|6666666.28487.peg.5354	Protein	50949	51281	+	FIG00832000: hypothetical protein
contig_68	fig|6666666.28487.peg.5355	Protein	52516	51278	-	H+ antiporter integral membrane transport protein
contig_68	fig|6666666.28487.peg.5356	Protein	53147	52584	-	Probable serine/threonine-protein kinase pknH (EC 2.7.11.1)
contig_68	fig|6666666.28487.peg.5357	Protein	53471	54220	+	Uracil-DNA glycosylase, family 5
contig_68	fig|6666666.28487.peg.5358	Protein	54247	55296	+	bacterial luciferase family protein
contig_68	fig|6666666.28487.peg.5359	Protein	55751	55293	-	FIG029194: hypothetical protein
contig_68	fig|6666666.28487.peg.5360	Protein	56183	55755	-	Bis(5'-nucleosyl)-tetraphosphatase (asymmetrical) (EC 3.6.1.17)
contig_68	fig|6666666.28487.peg.5361	Protein	56675	56202	-	Glyoxalase family protein

contig_68	fig|6666666.28487.peg.5362	Protein	56702	57538	+	Transcriptional regulator, AraC family
contig_68	fig|6666666.28487.peg.5363	Protein	58019	57516	-	G:T/U mismatch-specific uracil/thymine DNA-glycosylase
contig_68	fig|6666666.28487.peg.5364	Protein	58044	59165	+	Adenylate cyclase (EC 4.6.1.1)
contig_69	fig|6666666.28487.peg.5365	Protein	3209	87	-	hypothetical protein
contig_69	fig|6666666.28487.peg.5366	Protein	4187	3540	-	Transcriptional regulator, TetR family
contig_69	fig|6666666.28487.peg.5367	Protein	4409	5149	+	FIG00824710: hypothetical protein
contig_69	fig|6666666.28487.peg.5368	Protein	6173	5283	-	Hydrolase
contig_69	fig|6666666.28487.peg.5369	Protein	6684	6232	-	Thioesterase family protein
contig_69	fig|6666666.28487.peg.5370	Protein	6830	6669	-	hypothetical protein
contig_69	fig|6666666.28487.peg.5371	Protein	8055	6859	-	putative cytochrome P450 hydroxylase
contig_69	fig|6666666.28487.peg.5372	Protein	8129	8725	+	Transcriptional regulator, TetR family
contig_69	fig|6666666.28487.peg.5373	Protein	8722	9771	+	F420-dependent N(5),N(10)-methylenetetrahydromethanopterin reductase (EC 1.5.99.11)
contig_69	fig|6666666.28487.peg.5374	Protein	10284	9820	-	FIG00823589: hypothetical protein
contig_69	fig|6666666.28487.peg.5375	Protein	10890	10363	-	Transcriptional regulator, TetR family
contig_69	fig|6666666.28487.peg.5376	Protein	11543	11031	-	FIG00821827: hypothetical protein
contig_69	fig|6666666.28487.peg.5377	Protein	11619	12338	+	Transcriptional regulator, TetR family
contig_69	fig|6666666.28487.peg.5378	Protein	12355	12501	+	hypothetical protein
contig_69	fig|6666666.28487.peg.5379	Protein	14022	12688	-	hypothetical protein
contig_69	fig|6666666.28487.peg.5380	Protein	14468	14151	-	hypothetical protein

contig_69	fig|6666666.28487.peg.5381	Protein	15600	14473	-	Phage-related integrase/recombinase
contig_7	fig|6666666.28487.peg.5382	Protein	148	282	+	hypothetical protein
contig_7	fig|6666666.28487.peg.5383	Protein	1299	268	-	FIG313892: hypothetical protein
contig_7	fig|6666666.28487.peg.5384	Protein	2143	1427	-	FIG00822416: hypothetical protein
contig_7	fig|6666666.28487.rna.42	RNA	2255	2327	+	tRNA-Thr-GGT
contig_7	fig|6666666.28487.rna.43	RNA	2360	2433	+	tRNA-Met-CAT
contig_7	fig|6666666.28487.peg.5385	Protein	2475	2642	+	LSU ribosomal protein L33p @ LSU ribosomal protein L33p, zinc-dependent
contig_7	fig|6666666.28487.peg.5386	Protein	2709	3188	+	FIG00995223: hypothetical protein
contig_7	fig|6666666.28487.peg.5387	Protein	3163	3603	+	MaoC domain protein dehydratase
contig_7	fig|6666666.28487.peg.5388	Protein	3614	4168	+	FIG00820537: hypothetical protein
contig_7	fig|6666666.28487.rna.44	RNA	4294	4366	+	tRNA-Trp-CCA
contig_7	fig|6666666.28487.peg.5389	Protein	4452	4904	+	Preprotein translocase subunit SecE (TC 3.A.5.1.1)
contig_7	fig|6666666.28487.peg.5390	Protein	4949	5806	+	Transcription antitermination protein NusG
contig_7	fig|6666666.28487.peg.5391	Protein	5852	6280	+	LSU ribosomal protein L11p (L12e)
contig_7	fig|6666666.28487.peg.5392	Protein	6408	7124	+	LSU ribosomal protein L1p (L10Ae)
contig_7	fig|6666666.28487.peg.5393	Protein	7787	7425	-	FIG00829968: hypothetical protein
contig_7	fig|6666666.28487.peg.5394	Protein	8029	9609	+	PE family protein
contig_7	fig|6666666.28487.peg.5395	Protein	10576	9680	-	Cyclopropane-fatty-acyl-phospholipid synthase (EC 2.1.1.79)
contig_7	fig|6666666.28487.peg.5396	Protein	11595	10711	-	Cyclopropane-fatty-acyl-phospholipid synthase (EC 2.1.1.79)

contig_7	fig|6666666.28487.peg.5397	Protein	12604	11702	-	carboxyl esterase
contig_7	fig|6666666.28487.peg.5398	Protein	13918	12605	-	Ubiquinone biosynthesis monooxygenase UbiB
contig_7	fig|6666666.28487.peg.5399	Protein	15029	14031	-	FIG00828006: hypothetical protein
contig_7	fig|6666666.28487.peg.5400	Protein	15120	15728	+	Transcriptional regulator, TetR family
contig_7	fig|6666666.28487.peg.5401	Protein	16336	15737	-	FIG00823430: hypothetical protein
contig_7	fig|6666666.28487.peg.5402	Protein	16886	16341	-	FIG00822285: hypothetical protein
contig_7	fig|6666666.28487.peg.5403	Protein	16941	17894	+	Transcriptional regulator, DeoR family
contig_7	fig|6666666.28487.peg.5404	Protein	17912	22027	+	Alpha-mannosidase (EC 3.2.1.24)
contig_7	fig|6666666.28487.peg.5405	Protein	22024	22623	+	POSSIBLE MALONYL COA-ACYL CARRIER PROTEIN TRANSACYLASE FABD2 (MCT) (EC 2.3.1.39)
contig_7	fig|6666666.28487.peg.5406	Protein	22663	23634	+	Transcriptional regulator/sugar kinase
contig_7	fig|6666666.28487.peg.5407	Protein	24765	23623	-	benABC operon transcriptional activator BenR
contig_7	fig|6666666.28487.peg.5408	Protein	24955	25503	+	hypothetical protein
contig_7	fig|6666666.28487.peg.5409	Protein	25585	26556	+	Uptake hydrogenase small subunit precursor (EC 1.12.99.6)
contig_7	fig|6666666.28487.peg.5410	Protein	26607	28214	+	Uptake hydrogenase large subunit (EC 1.12.99.6)
contig_7	fig|6666666.28487.peg.5411	Protein	28211	29038	+	Hydrogenase maturation protease (EC 3.4.24.-)
contig_7	fig|6666666.28487.peg.5412	Protein	29125	29304	+	hypothetical protein
contig_7	fig|6666666.28487.peg.5413	Protein	29309	30565	+	FOG: TPR repeat
contig_7	fig|6666666.28487.peg.5414	Protein	30562	31461	+	Nitrogen-fixing NifU, C-terminal:Rieske [2Fe-2S] region
contig_7	fig|6666666.28487.peg.5415	Protein	31440	32651	+	Transcriptional regulator

contig_7	fig|6666666.28487.peg.5416	Protein	32648	33514	+	FIG00829376: hypothetical protein
contig_7	fig|6666666.28487.peg.5417	Protein	34276	33494	-	[NiFe] hydrogenase nickel incorporation-associated protein HypB
contig_7	fig|6666666.28487.peg.5418	Protein	34626	34291	-	[NiFe] hydrogenase nickel incorporation protein HypA
contig_7	fig|6666666.28487.peg.5419	Protein	34801	37104	+	[NiFe] hydrogenase metallocenter assembly protein HypF
contig_7	fig|6666666.28487.peg.5420	Protein	37152	37370	+	[NiFe] hydrogenase metallocenter assembly protein HypC
contig_7	fig|6666666.28487.peg.5421	Protein	37367	38470	+	[NiFe] hydrogenase metallocenter assembly protein HypD
contig_7	fig|6666666.28487.peg.5422	Protein	38502	39524	+	[NiFe] hydrogenase metallocenter assembly protein HypE
contig_7	fig|6666666.28487.peg.5423	Protein	39789	40319	+	LSU ribosomal protein L10p (P0)
contig_7	fig|6666666.28487.peg.5424	Protein	40370	40762	+	LSU ribosomal protein L7/L12 (P1/P2)
contig_7	fig|6666666.28487.peg.5425	Protein	40992	42065	+	Methionine ABC transporter ATP-binding protein
contig_7	fig|6666666.28487.peg.5426	Protein	42262	44031	+	FIG00825293: hypothetical protein
contig_7	fig|6666666.28487.peg.5427	Protein	44428	47796	+	DNA-directed RNA polymerase beta subunit (EC 2.7.7.6)
contig_7	fig|6666666.28487.peg.5428	Protein	47847	51797	+	DNA-directed RNA polymerase beta' subunit (EC 2.7.7.6)
contig_7	fig|6666666.28487.peg.5429	Protein	51943	52842	+	hypothetical protein
contig_7	fig|6666666.28487.peg.5430	Protein	52872	53633	+	Endonuclease IV (EC 3.1.21.2)
contig_7	fig|6666666.28487.peg.5431	Protein	54195	56585	+	PE-PGRS FAMILY PROTEIN
contig_7	fig|6666666.28487.peg.5432	Protein	57517	56642	-	Formyltetrahydrofolate deformylase (EC 3.5.1.10)
contig_7	fig|6666666.28487.peg.5433	Protein	59239	57530	-	Glycosyl transferase, group 2 family protein
contig_7	fig|6666666.28487.peg.5434	Protein	60289	59243	-	FIG00826023: hypothetical protein

contig_7	fig|6666666.28487.peg.5435	Protein	60504	60983	+	FIG00833290: hypothetical protein
contig_7	fig|6666666.28487.peg.5436	Protein	61037	62665	+	Acyl-CoA dehydrogenase (EC 1.3.99.3)
contig_7	fig|6666666.28487.peg.5437	Protein	62665	63603	+	Enoyl-CoA hydratase
contig_7	fig|6666666.28487.peg.5438	Protein	63615	64328	+	Phenylacetic acid degradation operon negative regulatory protein PaaX
contig_7	fig|6666666.28487.peg.5439	Protein	64325	65095	+	Enoyl-CoA hydratase (EC 4.2.1.17)
contig_7	fig|6666666.28487.peg.5440	Protein	65508	65107	-	Cell division protein DivIC (FtsB), stabilizes FtsL against RasP cleavage
contig_7	fig|6666666.28487.peg.5441	Protein	65864	65511	-	Probable conserved transmembrane protein
contig_7	fig|6666666.28487.peg.5442	Protein	65977	66510	+	Possible lipoprotein LprC
contig_7	fig|6666666.28487.peg.5443	Protein	66512	67048	+	Putative lipoprotein lprB precursor
contig_7	fig|6666666.28487.peg.5444	Protein	67107	67685	+	Transcriptional regulator, TetR family
contig_7	fig|6666666.28487.peg.5445	Protein	67978	68352	+	SSU ribosomal protein S12p (S23e)
contig_7	fig|6666666.28487.peg.5446	Protein	68352	68822	+	SSU ribosomal protein S7p (S5e)
contig_7	fig|6666666.28487.peg.5447	Protein	68901	71003	+	Translation elongation factor G
contig_7	fig|6666666.28487.peg.5448	Protein	71089	72279	+	Translation elongation factor Tu
contig_7	fig|6666666.28487.peg.5449	Protein	72520	73293	+	hypothetical protein
contig_7	fig|6666666.28487.peg.5450	Protein	73377	74231	+	Membrane proteins related to metalloendopeptidases
contig_7	fig|6666666.28487.peg.5451	Protein	74271	75098	+	FIG025093: Probable membrane protein
contig_7	fig|6666666.28487.peg.5452	Protein	75141	75998	+	3-oxoacyl-[acyl-carrier protein] reductase (EC 1.1.1.100)

contig_7	fig|6666666.28487.peg.5453	Protein	77227	75995	-	Ornithine aminotransferase (EC 2.6.1.13)
contig_7	fig|6666666.28487.peg.5454	Protein	78063	77200	-	NG,NG-dimethylarginine dimethylaminohydrolase 1 (EC 3.5.3.18)
contig_7	fig|6666666.28487.peg.5455	Protein	78172	78624	+	Transcriptional regulator, AsnC family
contig_7	fig|6666666.28487.peg.5456	Protein	78681	79865	+	Ferredoxin reductase
contig_70	fig|6666666.28487.peg.5457	Protein	101	745	+	Partial REP13E12 repeat protein
contig_70	fig|6666666.28487.peg.5458	Protein	2956	746	-	Lead, cadmium, zinc and mercury transporting ATPase (EC 3.6.3.3) (EC 3.6.3.5); Copper-translocating P-type ATPase (EC 3.6.3.4)
contig_70	fig|6666666.28487.peg.5459	Protein	3114	2953	-	hypothetical protein
contig_70	fig|6666666.28487.peg.5460	Protein	3323	3120	-	Copper chaperone
contig_70	fig|6666666.28487.peg.5461	Protein	3405	4037	+	protein of unknown function DUF305
contig_70	fig|6666666.28487.peg.5462	Protein	4047	4478	+	D-tyrosyl-tRNA(Tyr) deacylase
contig_70	fig|6666666.28487.peg.5463	Protein	4578	4772	+	hypothetical protein
contig_70	fig|6666666.28487.peg.5464	Protein	5687	4800	-	DUF124 domain-containing protein
contig_70	fig|6666666.28487.peg.5465	Protein	5765	5968	+	hypothetical protein
contig_70	fig|6666666.28487.peg.5466	Protein	6034	6603	+	FIG00820425: hypothetical protein
contig_70	fig|6666666.28487.peg.5467	Protein	8750	6600	-	oxidoreductase, molybdopterin-binding
contig_70	fig|6666666.28487.peg.5468	Protein	10632	8830	-	Lipid A export ATP-binding/permease protein MsbA
contig_70	fig|6666666.28487.peg.5469	Protein	12431	10743	-	Lipid A export ATP-binding/permease protein MsbA
contig_70	fig|6666666.28487.peg.5470	Protein	12670	13209	+	Lipoprotein LprB

contig_70	fig|6666666.28487.peg.5471	Protein	13206	13751	+	Putative lipoprotein LprC precursor
contig_70	fig|6666666.28487.peg.5472	Protein	14347	13829	-	Phosphohistidine phosphatase SixA
contig_70	fig|6666666.28487.peg.5473	Protein	14471	15610	+	DNA double-strand break repair protein Mre11
contig_70	fig|6666666.28487.peg.5474	Protein	15607	18261	+	DNA double-strand break repair Rad50 ATPase
contig_70	fig|6666666.28487.peg.5475	Protein	18308	19573	+	Proline iminopeptidase (EC 3.4.11.5)
contig_70	fig|6666666.28487.peg.5476	Protein	21212	19563	-	Oligopeptide ABC transporter, periplasmic oligopeptide-binding protein OppA (TC 3.A.1.5.1)
contig_70	fig|6666666.28487.peg.5477	Protein	23113	21278	-	Oligopeptide transport system permease protein OppB (TC 3.A.1.5.1)
contig_70	fig|6666666.28487.peg.5478	Protein	24024	23110	-	Oligopeptide transport system permease protein OppC (TC 3.A.1.5.1)
contig_70	fig|6666666.28487.peg.5479	Protein	24998	24021	-	Oligopeptide transport system permease protein OppB (TC 3.A.1.5.1)
contig_70	fig|6666666.28487.peg.5480	Protein	25600	25052	-	hypothetical protein
contig_70	fig|6666666.28487.peg.5481	Protein	25731	26483	+	putative two-component system response regulator
contig_70	fig|6666666.28487.peg.5482	Protein	26542	28122	+	sensor histidine kinase
contig_70	fig|6666666.28487.peg.5483	Protein	28235	28432	+	glycosyl transferase, family 39
contig_70	fig|6666666.28487.peg.5484	Protein	28442	30025	+	putative integral membrane protein
contig_70	fig|6666666.28487.peg.5485	Protein	30055	31338	+	Glycosyl transferase
contig_70	fig|6666666.28487.peg.5486	Protein	31335	33218	+	putative integral membrane protein
contig_70	fig|6666666.28487.peg.5487	Protein	33420	34349	+	Sulfate adenylyltransferase subunit 2 (EC 2.7.7.4)

contig_70	fig|6666666.28487.peg.5488	Protein	34412	36268	+	Sulfate adenylyltransferase subunit 1 (EC 2.7.7.4) / Adenylylsulfate kinase (EC 2.7.1.25)
contig_70	fig|6666666.28487.peg.5489	Protein	36297	37028	+	CysQ protein
contig_70	fig|6666666.28487.peg.5490	Protein	37157	37705	+	Isochorismatase (EC 3.3.2.1)
contig_70	fig|6666666.28487.peg.5491	Protein	37825	38301	+	Predicted transcriptional regulator of sulfate adenylyltransferase, Rrf2 family
contig_70	fig|6666666.28487.peg.5492	Protein	38681	38298	-	hypothetical protein
contig_70	fig|6666666.28487.peg.5493	Protein	38755	39777	+	Enoyl-[acyl-carrier-protein] reductase [FMN] (EC 1.3.1.9)
contig_70	fig|6666666.28487.peg.5494	Protein	39777	41123	+	FIG00827153: hypothetical protein
contig_70	fig|6666666.28487.peg.5495	Protein	42094	41198	-	FIG00829380: hypothetical protein
contig_70	fig|6666666.28487.peg.5496	Protein	42757	42113	-	Putative two-component system response regulator
contig_70	fig|6666666.28487.peg.5497	Protein	44082	42745	-	two-component sensor histidine kinase
contig_70	fig|6666666.28487.peg.5498	Protein	44187	45383	+	C4-dicarboxylate transporter/malic acid transport protein
contig_70	fig|6666666.28487.peg.5499	Protein	45428	46309	+	FIG00829736: hypothetical protein
contig_70	fig|6666666.28487.peg.5500	Protein	46329	47009	+	FIG172199: hypothetical thioredoxin family protein
contig_70	fig|6666666.28487.peg.5501	Protein	47227	47006	-	hypothetical protein
contig_70	fig|6666666.28487.peg.5502	Protein	47569	48321	+	Flagellar hook-length control protein FliK
contig_70	fig|6666666.28487.peg.5503	Protein	48349	50859	+	FIG00823980: hypothetical protein
contig_70	fig|6666666.28487.peg.5504	Protein	51978	50872	-	Acyl-CoA dehydrogenase family protein
contig_70	fig|6666666.28487.peg.5505	Protein	53153	51996	-	FadE30
contig_70	fig|6666666.28487.peg.5506	Protein	54132	53314	-	carveol dehydrogenase ((+)-trans-carveol dehydrogenase)(

						EC:1.1.1.275)
contig_70	fig|6666666.28487.peg.5507	Protein	54995	54135	-	FIG00823697: hypothetical protein
contig_70	fig|6666666.28487.peg.5508	Protein	56250	54997	-	FIG00824606: hypothetical protein
contig_70	fig|6666666.28487.peg.5509	Protein	56732	56289	-	FIG00821499: hypothetical protein
contig_70	fig|6666666.28487.peg.5510	Protein	56806	57696	+	dehydrogenase
contig_70	fig|6666666.28487.peg.5511	Protein	58517	57702	-	probable lipoprotein YPO2292
contig_70	fig|6666666.28487.peg.5512	Protein	58743	58567	-	hypothetical protein
contig_70	fig|6666666.28487.peg.5513	Protein	58747	59508	+	probable lipoprotein YPO2292
contig_70	fig|6666666.28487.peg.5514	Protein	59632	60183	+	hypothetical protein
contig_70	fig|6666666.28487.peg.5515	Protein	60303	60566	+	hypothetical protein
contig_70	fig|6666666.28487.peg.5516	Protein	60671	61090	+	hypothetical protein
contig_70	fig|6666666.28487.peg.5517	Protein	61552	61346	-	hypothetical protein
contig_70	fig|6666666.28487.peg.5518	Protein	61712	62464	+	hypothetical protein
contig_70	fig|6666666.28487.peg.5519	Protein	62564	63178	+	hypothetical protein
contig_70	fig|6666666.28487.peg.5520	Protein	63357	64664	+	probable exported protease [EC:3.4.-.-]
contig_70	fig|6666666.28487.peg.5521	Protein	64685	65707	+	hypothetical protein
contig_70	fig|6666666.28487.rna.45	RNA	65862	65790	-	tRNA-Arg-CCG
contig_70	fig|6666666.28487.peg.5522	Protein	66072	67724	+	Arginyl-tRNA synthetase (EC 6.1.1.19)
contig_70	fig|6666666.28487.peg.5523	Protein	67721	69154	+	Diaminopimelate decarboxylase (EC 4.1.1.20)

contig_70	fig|6666666.28487.peg.5524	Protein	69151	70479	+	Homoserine dehydrogenase (EC 1.1.1.3)
contig_70	fig|6666666.28487.peg.5525	Protein	70476	71555	+	Threonine synthase (EC 4.2.3.1)
contig_70	fig|6666666.28487.peg.5526	Protein	71567	72511	+	Homoserine kinase (EC 2.7.1.39)
contig_70	fig|6666666.28487.peg.5527	Protein	72798	74801	+	Transcription termination factor Rho
contig_70	fig|6666666.28487.peg.5528	Protein	75262	74843	-	Transcriptional regulator, MarR family
contig_70	fig|6666666.28487.peg.5529	Protein	75334	75936	+	FIG00825029: hypothetical protein
contig_70	fig|6666666.28487.peg.5530	Protein	76669	76007	-	Transcriptional regulator, TetR family
contig_70	fig|6666666.28487.peg.5531	Protein	76766	78346	+	Long-chain-fatty-acid--CoA ligase (EC 6.2.1.3)
contig_70	fig|6666666.28487.peg.5532	Protein	78496	78720	+	LSU ribosomal protein L31p
contig_70	fig|6666666.28487.peg.5533	Protein	78805	79878	+	Peptide chain release factor 1
contig_70	fig|6666666.28487.peg.5534	Protein	79896	80729	+	Protein-N(5)-glutamine methyltransferase PrmC, methylates polypeptide chain release factors RF1 and RF2
contig_70	fig|6666666.28487.peg.5535	Protein	80743	81408	+	"YrdC/Sua5 family protein, required for threonylcarbamoyladenosine (t(6)A) formation in tRNA"
contig_70	fig|6666666.28487.peg.5536	Protein	81420	82637	+	Undecaprenyl-phosphate N-acetylglucosaminyl 1-phosphate transferase (EC 2.7.8.-)
contig_70	fig|6666666.28487.peg.5537	Protein	82900	83346	+	FIG048548: ATP synthase protein I2
contig_70	fig|6666666.28487.peg.5538	Protein	83357	84130	+	ATP synthase A chain (EC 3.6.3.14)
contig_70	fig|6666666.28487.peg.5539	Protein	84219	84464	+	ATP synthase C chain (EC 3.6.3.14)
contig_70	fig|6666666.28487.peg.5540	Protein	84474	84989	+	ATP synthase B chain (EC 3.6.3.14)
contig_70	fig|6666666.28487.peg.5541	Protein	84990	86327	+	ATP synthase B' chain (EC 3.6.3.14) / ATP synthase delta

						chain (EC 3.6.3.14)
contig_70	fig|6666666.28487.peg.5542	Protein	86377	88023	+	ATP synthase alpha chain (EC 3.6.3.14)
contig_70	fig|6666666.28487.peg.5543	Protein	88049	88981	+	ATP synthase gamma chain (EC 3.6.3.14)
contig_70	fig|6666666.28487.peg.5544	Protein	89007	90434	+	ATP synthase beta chain (EC 3.6.3.14)
contig_70	fig|6666666.28487.peg.5545	Protein	90498	90863	+	ATP synthase epsilon chain (EC 3.6.3.14)
contig_70	fig|6666666.28487.peg.5546	Protein	90882	91316	+	Possible membrane protein
contig_70	fig|6666666.28487.peg.5547	Protein	91931	91356	-	Cob(I)alamin adenosyltransferase PduO (EC 2.5.1.17)
contig_70	fig|6666666.28487.peg.5548	Protein	92014	93267	+	UDP-N-acetylglucosamine 1-carboxyvinyltransferase (EC 2.5.1.7)
contig_71	fig|6666666.28487.peg.5549	Protein	4172	1086	-	Putative membrane protein
contig_71	fig|6666666.28487.peg.5550	Protein	4240	5502	+	Glycosyl transferase family protein
contig_71	fig|6666666.28487.peg.5551	Protein	6805	5483	-	hypothetical protein
contig_71	fig|6666666.28487.peg.5552	Protein	8144	6807	-	hypothetical protein
contig_71	fig|6666666.28487.peg.5553	Protein	9612	8146	-	POSSIBLE CONSERVED POLYKETIDE SYNTHASE ASSOCIATED PROTEIN PAPA2
contig_71	fig|6666666.28487.peg.5554	Protein	15851	9612	-	Polyketide synthase
contig_71	fig|6666666.28487.peg.5555	Protein	16280	18874	+	AMP-dependent synthetase and ligase
contig_71	fig|6666666.28487.peg.5556	Protein	19679	18846	-	Enoyl-CoA hydratase (EC 4.2.1.17)
contig_71	fig|6666666.28487.peg.5557	Protein	20060	19698	-	Thioredoxin TrxB1
contig_71	fig|6666666.28487.peg.5558	Protein	21580	20099	-	endo-beta-1,3-glucanase

contig_71	fig|6666666.28487.peg.5559	Protein	22572	22132	-	hypothetical protein
contig_71	fig|6666666.28487.peg.5560	Protein	23257	22601	-	Short-chain dehydrogenase/reductase SDR
contig_72	fig|6666666.28487.peg.5561	Protein	55	366	+	Mobile element protein
contig_72	fig|6666666.28487.peg.5562	Protein	423	1325	+	Mobile element protein
contig_73	fig|6666666.28487.peg.5563	Protein	77	313	+	Preprotein translocase subunit SecG (TC 3.A.5.1.1)
contig_73	fig|6666666.28487.peg.5564	Protein	629	333	-	hypothetical protein
contig_73	fig|6666666.28487.peg.5565	Protein	718	3531	+	Phosphoenolpyruvate carboxylase (EC 4.1.1.31)
contig_73	fig|6666666.28487.peg.5566	Protein	3923	3552	-	FIG00820198: hypothetical protein
contig_73	fig|6666666.28487.peg.5567	Protein	4016	4618	+	hypothetical protein
contig_73	fig|6666666.28487.peg.5568	Protein	4758	5162	+	FIG00820240: hypothetical protein
contig_73	fig|6666666.28487.peg.5569	Protein	5897	5163	-	6-phosphogluconolactonase (EC 3.1.1.31), eukaryotic type
contig_73	fig|6666666.28487.peg.5570	Protein	6805	5894	-	OpcA, an allosteric effector of glucose-6-phosphate dehydrogenase, actinobacterial
contig_73	fig|6666666.28487.peg.5571	Protein	8364	6802	-	Glucose-6-phosphate 1-dehydrogenase (EC 1.1.1.49)
contig_73	fig|6666666.28487.peg.5572	Protein	9495	8380	-	Transaldolase (EC 2.2.1.2)
contig_73	fig|6666666.28487.peg.5573	Protein	11601	9511	-	Transketolase (EC 2.2.1.1)
contig_73	fig|6666666.28487.peg.5574	Protein	11801	12742	+	Heme O synthase, protoheme IX farnesyltransferase (EC 2.5.1.-) COX10-CtaB
contig_73	fig|6666666.28487.peg.5575	Protein	12745	13689	+	FIG00827769: hypothetical protein
contig_73	fig|6666666.28487.peg.5576	Protein	13749	15047	+	Proton/glutamate symporter

contig_73	fig|6666666.28487.peg.5577	Protein	16498	15044	-	Quinone oxidoreductase (EC 1.6.5.5)
contig_73	fig|6666666.28487.peg.5578	Protein	17863	16928	-	Cytochrome oxidase assembly protein
contig_73	fig|6666666.28487.peg.5579	Protein	18498	17905	-	Transcriptional regulator, TetR family
contig_73	fig|6666666.28487.peg.5580	Protein	19980	19210	-	ABC-type multidrug transport system, permease component
contig_73	fig|6666666.28487.peg.5581	Protein	20870	19977	-	ABC-type multidrug transport system, ATPase component
contig_73	fig|6666666.28487.peg.5582	Protein	22588	20912	-	POSSIBLE CONSERVED INTEGRAL MEMBRANE PROTEIN
contig_73	fig|6666666.28487.peg.5583	Protein	22708	23415	+	Iron-sulfur cluster regulator SufR
contig_73	fig|6666666.28487.peg.5584	Protein	23487	24926	+	Iron-sulfur cluster assembly protein SufB
contig_73	fig|6666666.28487.peg.5585	Protein	24929	26113	+	Iron-sulfur cluster assembly protein SufD
contig_73	fig|6666666.28487.peg.5586	Protein	26103	26885	+	Iron-sulfur cluster assembly ATPase protein SufC
contig_73	fig|6666666.28487.peg.5587	Protein	26887	28149	+	Cysteine desulfurase (EC 2.8.1.7), SufS subfamily
contig_73	fig|6666666.28487.peg.5588	Protein	28149	28610	+	Putative iron-sulfur cluster assembly scaffold protein for SUF system, SufE2
contig_73	fig|6666666.28487.peg.5589	Protein	28610	28993	+	PaaD-like protein (DUF59) involved in Fe-S cluster assembly
contig_73	fig|6666666.28487.peg.5590	Protein	29897	28983	-	putative DNA-binding protein
contig_74	fig|6666666.28487.peg.5591	Protein	102	878	+	hypothetical protein
contig_74	fig|6666666.28487.peg.5592	Protein	997	1686	+	Putative oxidoreductase
contig_74	fig|6666666.28487.peg.5593	Protein	1820	2629	+	Long-chain-fatty-acid--CoA ligase (EC 6.2.1.3)
contig_74	fig|6666666.28487.peg.5594	Protein	2633	4279	+	Long-chain-fatty-acid--CoA ligase (EC 6.2.1.3)

contig_74	fig|6666666.28487.peg.5595	Protein	4272	5552	+	Lipase (EC 3.1.1.3)
contig_74	fig|6666666.28487.peg.5596	Protein	6993	5602	-	Diacylglycerol O-acyltransferase (EC 2.3.1.20)
contig_74	fig|6666666.28487.peg.5597	Protein	8275	7115	-	Esterase LipC
contig_74	fig|6666666.28487.peg.5598	Protein	8505	10682	+	Acyl-CoA dehydrogenase, short-chain specific (EC 1.3.99.2)
contig_74	fig|6666666.28487.peg.5599	Protein	10679	11632	+	Esterase LipW
contig_74	fig|6666666.28487.peg.5600	Protein	12903	11629	-	FIG00821918: hypothetical protein
contig_74	fig|6666666.28487.peg.5601	Protein	14003	12897	-	FIG00820062: hypothetical protein
contig_74	fig|6666666.28487.peg.5602	Protein	15032	14028	-	FIG024080: hypothetical protein
contig_74	fig|6666666.28487.peg.5603	Protein	15006	15131	+	hypothetical protein
contig_74	fig|6666666.28487.peg.5604	Protein	15109	16266	+	Butyryl-CoA dehydrogenase(EC:1.3.99.2)
contig_74	fig|6666666.28487.peg.5605	Protein	16266	16988	+	transcriptional regulator, GntR family
contig_74	fig|6666666.28487.peg.5606	Protein	17812	16958	-	helix-turn-helix domain protein
contig_74	fig|6666666.28487.peg.5607	Protein	17892	18626	+	oxidoreductase alr2142 [imported]
contig_74	fig|6666666.28487.peg.5608	Protein	19506	18628	-	Enoyl-CoA hydratase [valine degradation] (EC 4.2.1.17)
contig_74	fig|6666666.28487.peg.5609	Protein	21023	19503	-	Long-chain-fatty-acid--CoA ligase (EC 6.2.1.3)
contig_74	fig|6666666.28487.peg.5610	Protein	21119	21766	+	transcriptional regulator, TetR family
contig_74	fig|6666666.28487.peg.5611	Protein	21850	23415	+	hypothetical protein
contig_74	fig|6666666.28487.peg.5612	Protein	23412	24233	+	putative oxidoreductase, short-chain dehydrogenase/reductase family
contig_74	fig|6666666.28487.peg.5613	Protein	26144	24315	-	Phosphoenolpyruvate carboxykinase [GTP] (EC 4.1.1.32)

contig_74	fig|6666666.28487.peg.5614	Protein	27450	26308	-	FIG033897: hypothetical protein
contig_74	fig|6666666.28487.peg.5615	Protein	28484	27504	-	FIG032248: hypothetical protein
contig_74	fig|6666666.28487.peg.5616	Protein	28652	29389	+	tRNA (guanine46-N7-)-methyltransferase (EC 2.1.1.33)
contig_74	fig|6666666.28487.peg.5617	Protein	29386	30111	+	FIG01001556: hypothetical protein
contig_74	fig|6666666.28487.peg.5618	Protein	30174	33488	+	Transmembrane transport protein MmpL5
contig_74	fig|6666666.28487.peg.5619	Protein	33858	33508	-	putative dioxygenase
contig_74	fig|6666666.28487.peg.5620	Protein	35019	33865	-	Possible membrane protein
contig_74	fig|6666666.28487.peg.5621	Protein	35097	36272	+	Possible membrane protein
contig_74	fig|6666666.28487.peg.5622	Protein	36282	37535	+	hypothetical protein
contig_74	fig|6666666.28487.peg.5623	Protein	37522	37899	+	PROBABLE CONSERVED TRANSMEMBRANE PROTEIN
contig_74	fig|6666666.28487.peg.5624	Protein	39133	37901	-	sensor histidine kinase
contig_74	fig|6666666.28487.peg.5625	Protein	39972	39238	-	DNA-binding response regulator TrcR
contig_74	fig|6666666.28487.peg.5626	Protein	40394	39993	-	seq ID no 1F, putative
contig_74	fig|6666666.28487.peg.5627	Protein	40945	40535	-	POSSIBLE EXPORTED PROTEIN
contig_74	fig|6666666.28487.peg.5628	Protein	41235	44159	+	Transmembrane transport protein MmpL13
contig_74	fig|6666666.28487.peg.5629	Protein	44252	44671	+	FIG00821048: hypothetical protein
contig_74	fig|6666666.28487.peg.5630	Protein	44675	45346	+	hypothetical protein
contig_75	fig|6666666.28487.peg.5631	Protein	901	182	-	FIG00994821: hypothetical protein
contig_75	fig|6666666.28487.peg.5632	Protein	1047	1418	+	FIG00945047: hypothetical protein
contig_75	fig|6666666.28487.peg.5633	Protein	1415	2128	+	PROBABLE CONSERVED INTEGRAL MEMBRANE

						ALANINE AND LEUCINE RICH PROTEIN
contig_75	fig|6666666.28487.peg.5634	Protein	2801	2136	-	Trk system potassium uptake protein TrkA
contig_75	fig|6666666.28487.peg.5635	Protein	3555	2884	-	Trk system potassium uptake protein TrkA
contig_75	fig|6666666.28487.peg.5636	Protein	3656	5650	+	PROBABLE CONSERVED INTEGRAL MEMBRANE ALANINE AND VALINE AND LEUCINE RICH PROTEIN
contig_75	fig|6666666.28487.peg.5637	Protein	5647	6849	+	23S rRNA (Uracil-5-) -methyltransferase RumA (EC 2.1.1.-)
contig_75	fig|6666666.28487.peg.5638	Protein	6862	8244	+	Na+/H+ antiporter NhaA type
contig_75	fig|6666666.28487.peg.5639	Protein	8273	10186	+	1-deoxy-D-xylulose 5-phosphate synthase (EC 2.2.1.7)
contig_75	fig|6666666.28487.peg.5640	Protein	10232	11374	+	Lipase 1 (EC 3.1.1.3)
contig_75	fig|6666666.28487.peg.5641	Protein	12667	11396	-	Ribonuclease D (EC 3.1.26.3)
contig_75	fig|6666666.28487.peg.5642	Protein	13254	12673	-	Uncharacterized protein Q1 colocalized with Q
contig_75	fig|6666666.28487.peg.5643	Protein	13340	14404	+	Uroporphyrinogen III decarboxylase (EC 4.1.1.37)
contig_75	fig|6666666.28487.peg.5644	Protein	14479	15756	+	Protoporphyrinogen IX oxidase, aerobic, HemY (EC 1.3.3.4)
contig_75	fig|6666666.28487.peg.5645	Protein	15783	16487	+	Hemoprotein HemQ, essential component of heme biosynthetic pathway in Gram-positive bacteria
contig_75	fig|6666666.28487.peg.5646	Protein	16688	19033	+	diguanylate cyclase/phosphodiesterase
contig_75	fig|6666666.28487.peg.5647	Protein	19395	18985	-	Peptide methionine sulfoxide reductase MsrB (EC 1.8.4.12)
contig_75	fig|6666666.28487.peg.5648	Protein	20670	19405	-	Putative membrane protein Q2 colocalized with Q
contig_75	fig|6666666.28487.peg.5649	Protein	22244	20706	-	FIG00995896: hypothetical protein
contig_75	fig|6666666.28487.peg.5650	Protein	23075	22278	-	5-amino-6-(5-phosphoribosylamino)uracil reductase (EC 1.1.1.193) homolog

contig_75	fig|6666666.28487.peg.5651	Protein	23110	24159	+	putative ATP/GTP-binding integral membrane protein
contig_75	fig|6666666.28487.peg.5652	Protein	24671	24135	-	FIG00820775: hypothetical protein
contig_75	fig|6666666.28487.peg.5653	Protein	25159	24656	-	Putative alanine and valine rich exported protein
contig_75	fig|6666666.28487.peg.5654	Protein	25624	25199	-	pyridoxamine 5'-phosphate oxidase-related, FMN-binding
contig_75	fig|6666666.28487.peg.5655	Protein	26260	25661	-	Methylated-DNA--protein-cysteine methyltransferase (EC 2.1.1.63)
contig_75	fig|6666666.28487.peg.5656	Protein	26730	26257	-	RNA polymerase sigma-54 factor RpoN
contig_75	fig|6666666.28487.peg.5657	Protein	27496	26774	-	Clp N terminal domain protein
contig_75	fig|6666666.28487.peg.5658	Protein	28058	27546	-	FIG00820526: hypothetical protein
contig_75	fig|6666666.28487.peg.5659	Protein	28126	28455	+	hypothetical protein
contig_75	fig|6666666.28487.peg.5660	Protein	28871	28461	-	FIG00831320: hypothetical protein
contig_75	fig|6666666.28487.peg.5661	Protein	29339	29139	-	hypothetical protein
contig_75	fig|6666666.28487.peg.5662	Protein	29767	29468	-	FIG00824595: hypothetical protein
contig_76	fig|6666666.28487.peg.5663	Protein	916	2118	+	Phage capsid and scaffold
contig_76	fig|6666666.28487.peg.5664	Protein	2115	2462	+	hypothetical protein
contig_76	fig|6666666.28487.peg.5665	Protein	2646	3167	+	hypothetical protein
contig_76	fig|6666666.28487.peg.5666	Protein	3274	4149	+	phage major capsid protein, HK97
contig_76	fig|6666666.28487.peg.5667	Protein	4152	4466	+	hypothetical protein
contig_76	fig|6666666.28487.peg.5668	Protein	4678	4812	+	hypothetical protein
contig_76	fig|6666666.28487.peg.5669	Protein	5179	5418	+	hypothetical protein

contig_76	fig|6666666.28487.peg.5670	Protein	5627	6352	+	hypothetical protein
contig_76	fig|6666666.28487.peg.5671	Protein	7013	6402	-	Dihydrofolate reductase (EC 1.5.1.3)
contig_76	fig|6666666.28487.peg.5672	Protein	7077	8567	+	FIG00994218: hypothetical protein
contig_76	fig|6666666.28487.peg.5673	Protein	8647	9246	+	hypothetical protein
contig_76	fig|6666666.28487.peg.5674	Protein	9258	10625	+	Invasion protein IbeA
contig_76	fig|6666666.28487.peg.5675	Protein	11761	10634	-	Ferric iron ABC transporter, iron-binding protein
contig_76	fig|6666666.28487.peg.5676	Protein	12795	11845	-	serine esterase, cutinase family
contig_76	fig|6666666.28487.peg.5677	Protein	13929	12832	-	Oxalate decarboxylase (EC 4.1.1.2)
contig_76	fig|6666666.28487.peg.5678	Protein	14360	15028	+	conserved hypothetical protein
contig_76	fig|6666666.28487.peg.5679	Protein	15441	15034	-	Transcriptional regulator, AsnC family
contig_76	fig|6666666.28487.peg.5680	Protein	15650	19093	+	Indolepyruvate ferredoxin oxidoreductase, alpha and beta subunits
contig_76	fig|6666666.28487.peg.5681	Protein	19770	19090	-	Serine esterase, cutinase family
contig_76	fig|6666666.28487.peg.5682	Protein	20483	19938	-	hypothetical protein
contig_76	fig|6666666.28487.peg.5683	Protein	21286	20498	-	Streptomycin 6-kinase (EC 2.7.1.72) (Streptidine kinase) (Streptomycin 6-phosphotransferase) (APH(6))
contig_76	fig|6666666.28487.peg.5684	Protein	22522	21362	-	Limonene 1,2-monooxygenase
contig_76	fig|6666666.28487.peg.5685	Protein	22623	23963	+	Amino acid transporter
contig_76	fig|6666666.28487.peg.5686	Protein	24206	24742	+	hypothetical protein
contig_76	fig|6666666.28487.peg.5687	Protein	24757	25122	+	anti-sigma-factor antagonist

contig_76	fig|6666666.28487.peg.5688	Protein	25119	28880	+	Serine phosphatase RsbU, regulator of sigma subunit
contig_76	fig|6666666.28487.peg.5689	Protein	28882	29289	+	Serine phosphatase RsbU, regulator of sigma subunit
contig_76	fig|6666666.28487.peg.5690	Protein	29289	29600	+	Anti-sigma F factor antagonist (spoIIAA-2); Anti-sigma B factor antagonist RsbV
contig_76	fig|6666666.28487.peg.5691	Protein	30577	29597	-	ATP-dependent DNA ligase (EC 6.5.1.1)
contig_76	fig|6666666.28487.peg.5692	Protein	30680	32731	+	TRAP-type uncharacterized transport system, fused permease component
contig_76	fig|6666666.28487.peg.5693	Protein	32728	33768	+	TRAP transporter solute receptor, TAXI family precursor
contig_76	fig|6666666.28487.peg.5694	Protein	33945	34298	+	FIG00827264: hypothetical protein
contig_76	fig|6666666.28487.peg.5695	Protein	34452	37304	+	Putative membrane protein
contig_76	fig|6666666.28487.peg.5696	Protein	37986	37288	-	putative hydrolase
contig_77	fig|6666666.28487.peg.5697	Protein	54	1214	+	metallophosphoesterase
contig_77	fig|6666666.28487.peg.5698	Protein	1234	2079	+	Acyl-CoA thioesterase II (EC 3.1.2.-)
contig_77	fig|6666666.28487.peg.5699	Protein	2534	2100	-	FIG00827012: hypothetical protein
contig_77	fig|6666666.28487.peg.5700	Protein	3335	2544	-	acyltransferase family protein
contig_77	fig|6666666.28487.peg.5701	Protein	3445	5958	+	putative helicase
contig_77	fig|6666666.28487.peg.5702	Protein	5983	6672	+	hypothetical protein
contig_77	fig|6666666.28487.peg.5703	Protein	6818	6958	+	FIG00826683: hypothetical protein
contig_77	fig|6666666.28487.peg.5704	Protein	7790	6960	-	putative N5, N10-methylenetetrahydromethanopterin reductase-related protein
contig_77	fig|6666666.28487.peg.5705	Protein	8223	7837	-	hypothetical protein

contig_77	fig|6666666.28487.peg.5706	Protein	8408	8755	+	Possible membrane protein
contig_77	fig|6666666.28487.peg.5707	Protein	8770	9057	+	protein of unknown function DUF202
contig_77	fig|6666666.28487.peg.5708	Protein	10191	9052	-	putative RNA polymerase sigma factor
contig_78	fig|6666666.28487.peg.5709	Protein	1032	163	-	Enoyl-CoA hydratase (EC 4.2.1.17)
contig_78	fig|6666666.28487.peg.5710	Protein	1245	1081	-	FIG00827406: hypothetical protein
contig_78	fig|6666666.28487.peg.5711	Protein	2119	1616	-	Isoprenylcysteine carboxyl methyltransferase
contig_78	fig|6666666.28487.peg.5712	Protein	3230	2130	-	Chalcone synthase (EC 2.3.1.74)
contig_78	fig|6666666.28487.peg.5713	Protein	3804	3941	+	hypothetical protein
contig_79	fig|6666666.28487.peg.5714	Protein	774	1466	+	integrase
contig_79	fig|6666666.28487.peg.5715	Protein	1655	1858	+	hypothetical protein
contig_79	fig|6666666.28487.peg.5716	Protein	1986	3431	+	hypothetical protein
contig_79	fig|6666666.28487.peg.5717	Protein	4065	3472	-	Resolvase, N-terminal domain
contig_79	fig|6666666.28487.peg.5718	Protein	6577	4679	-	DNA helicase
contig_79	fig|6666666.28487.peg.5719	Protein	9371	8022	-	hypothetical protein
contig_79	fig|6666666.28487.rna.46	RNA	9793	9722	-	tRNA-Val-GAC
contig_79	fig|6666666.28487.rna.47	RNA	9878	9808	-	tRNA-Cys-GCA
contig_79	fig|6666666.28487.rna.48	RNA	9978	9906	-	tRNA-Gly-GCC
contig_79	fig|6666666.28487.peg.5720	Protein	10373	10074	-	hypothetical protein
contig_79	fig|6666666.28487.peg.5721	Protein	10372	10530	+	hypothetical protein

contig_79	fig|6666666.28487.rna.49	RNA	10612	10683	+	tRNA-Val-CAC
contig_79	fig|6666666.28487.peg.5722	Protein	12016	10808	-	FIG01028981: hypothetical protein
contig_79	fig|6666666.28487.peg.5723	Protein	13181	13026	-	hypothetical protein
contig_79	fig|6666666.28487.peg.5724	Protein	13207	13692	+	putative lipoprotein
contig_79	fig|6666666.28487.peg.5725	Protein	14881	13706	-	Osmosensitive K+ channel histidine kinase KdpD (EC 2.7.3.-)
contig_79	fig|6666666.28487.peg.5726	Protein	15624	14878	-	Two-component system response regulator
contig_79	fig|6666666.28487.peg.5727	Protein	15764	15913	+	hypothetical protein
contig_79	fig|6666666.28487.peg.5728	Protein	16576	15998	-	NLP/P60 family protein
contig_79	fig|6666666.28487.peg.5729	Protein	17642	17854	+	Copper chaperone
contig_79	fig|6666666.28487.peg.5730	Protein	17851	18087	+	hypothetical protein
contig_79	fig|6666666.28487.peg.5731	Protein	18176	20293	+	Lead, cadmium, zinc and mercury transporting ATPase (EC 3.6.3.3) (EC 3.6.3.5); Copper-translocating P-type ATPase (EC 3.6.3.4)
contig_79	fig|6666666.28487.peg.5732	Protein	21059	20328	-	hypothetical protein
contig_79	fig|6666666.28487.peg.5733	Protein	22460	21183	-	diguanylate cyclase with PAS/PAC sensor
contig_79	fig|6666666.28487.peg.5734	Protein	23006	23851	+	Universal stress protein family
contig_79	fig|6666666.28487.peg.5735	Protein	24556	23852	-	hypothetical protein
contig_79	fig|6666666.28487.peg.5736	Protein	25109	24675	-	14 kDa antigen (16 kDa antigen) (HSP 16.3)
contig_79	fig|6666666.28487.peg.5737	Protein	25355	25996	+	hypothetical protein
contig_79	fig|6666666.28487.peg.5738	Protein	26043	26831	+	Ribosomal subunit interface protein

contig_79	fig|6666666.28487.peg.5739	Protein	26966	27856	+	Universal stress protein family
contig_79	fig|6666666.28487.peg.5740	Protein	28347	27847	-	GCN5-related N-acetyltransferase
contig_79	fig|6666666.28487.peg.5741	Protein	28520	29110	+	PROBABLE CONSERVED TRANSMEMBRANE PROTEIN
contig_79	fig|6666666.28487.peg.5742	Protein	29151	29882	+	Universal stress protein family
contig_79	fig|6666666.28487.peg.5743	Protein	30502	29846	-	Two component transcriptional regulatory protein DevR
contig_79	fig|6666666.28487.peg.5744	Protein	31331	30507	-	Universal stress protein family
contig_79	fig|6666666.28487.peg.5745	Protein	32345	31362	-	Conserved hypothetical protein Acg
contig_79	fig|6666666.28487.peg.5746	Protein	32516	33970	+	Uncharacterized protein Mb2027c
contig_8	fig|6666666.28487.peg.5747	Protein	573	1664	+	Homogentisate 1,2-dioxygenase (EC 1.13.11.5)
contig_8	fig|6666666.28487.peg.5748	Protein	1665	2861	+	FIG00822874: hypothetical protein
contig_8	fig|6666666.28487.peg.5749	Protein	2825	4063	+	L-carnitine dehydratase/bile acid-inducible protein F (EC 2.8.3.16)
contig_8	fig|6666666.28487.peg.5750	Protein	4060	4965	+	Hydroxymethylglutaryl-CoA lyase (EC 4.1.3.4)
contig_8	fig|6666666.28487.peg.5751	Protein	4972	5592	+	Transcriptional regulator, TetR family
contig_8	fig|6666666.28487.peg.5752	Protein	5617	6855	+	Isovaleryl-CoA dehydrogenase (EC 1.3.99.10)
contig_8	fig|6666666.28487.peg.5753	Protein	6892	7890	+	FIG00823333: hypothetical protein
contig_8	fig|6666666.28487.peg.5754	Protein	7887	8225	+	FIG00825230: hypothetical protein
contig_8	fig|6666666.28487.peg.5755	Protein	8222	9094	+	putative hydrolase
contig_8	fig|6666666.28487.peg.5756	Protein	9736	9101	-	Methyltransferase (EC 2.1.1.-) colocalized with Q
contig_8	fig|6666666.28487.peg.5757	Protein	9779	10153	+	FIG00821985: hypothetical protein

contig_8	fig|6666666.28487.peg.5758	Protein	10223	10642	+	Probable response regulator
contig_8	fig|6666666.28487.peg.5759	Protein	10809	11183	+	NADH ubiquinone oxidoreductase chain A (EC 1.6.5.3)
contig_8	fig|6666666.28487.peg.5760	Protein	11174	11728	+	NADH-ubiquinone oxidoreductase chain B (EC 1.6.5.3)
contig_8	fig|6666666.28487.peg.5761	Protein	11725	12417	+	NADH-ubiquinone oxidoreductase chain C (EC 1.6.5.3)
contig_8	fig|6666666.28487.peg.5762	Protein	12417	13769	+	NADH-ubiquinone oxidoreductase chain D (EC 1.6.5.3)
contig_8	fig|6666666.28487.peg.5763	Protein	13777	14658	+	NADH-ubiquinone oxidoreductase chain E (EC 1.6.5.3)
contig_8	fig|6666666.28487.peg.5764	Protein	14655	15971	+	NADH-ubiquinone oxidoreductase chain F (EC 1.6.5.3)
contig_8	fig|6666666.28487.peg.5765	Protein	15968	18352	+	NADH-ubiquinone oxidoreductase chain G (EC 1.6.5.3)
contig_8	fig|6666666.28487.peg.5766	Protein	18349	19581	+	NADH-ubiquinone oxidoreductase chain H (EC 1.6.5.3)
contig_8	fig|6666666.28487.peg.5767	Protein	19574	20146	+	NADH-ubiquinone oxidoreductase chain I (EC 1.6.5.3)
contig_8	fig|6666666.28487.peg.5768	Protein	20143	20877	+	NADH-ubiquinone oxidoreductase chain J (EC 1.6.5.3)
contig_8	fig|6666666.28487.peg.5769	Protein	20874	21173	+	NADH-ubiquinone oxidoreductase chain K (EC 1.6.5.3)
contig_8	fig|6666666.28487.peg.5770	Protein	21190	23058	+	NADH-ubiquinone oxidoreductase chain L (EC 1.6.5.3)
contig_8	fig|6666666.28487.peg.5771	Protein	23052	24632	+	NADH-ubiquinone oxidoreductase chain M (EC 1.6.5.3)
contig_8	fig|6666666.28487.peg.5772	Protein	24629	26200	+	NADH-ubiquinone oxidoreductase chain N (EC 1.6.5.3)
contig_8	fig|6666666.28487.peg.5773	Protein	26213	26998	+	Enoyl-CoA hydratase (EC 4.2.1.17)
contig_8	fig|6666666.28487.peg.5774	Protein	27665	27165	-	Transcriptional regulator, TetR family
contig_8	fig|6666666.28487.peg.5775	Protein	27834	28970	+	Phosphotransferase
contig_8	fig|6666666.28487.peg.5776	Protein	28970	30085	+	Putative uncharacterized protein BCG_3193

contig_8	fig|6666666.28487.peg.5777	Protein	31638	30088	-	monooxygenase, flavin-binding family
contig_8	fig|6666666.28487.peg.5778	Protein	32659	31679	-	PROBABLE OXIDOREDUCTASE
contig_8	fig|6666666.28487.peg.5779	Protein	32747	34090	+	Amine oxidase [flavin-containing] A (EC 1.4.3.4)
contig_8	fig|6666666.28487.peg.5780	Protein	35160	34066	-	Non-heme haloperoxidase Hpx
contig_8	fig|6666666.28487.peg.5781	Protein	35277	36251	+	PROBABLE NADPH QUINONE OXIDOREDUCTASE FADB4 (NADPH:QUINONE REDUCTASE) (ZETA-CRYSTALLIN) (EC 1.6.5.5)
contig_8	fig|6666666.28487.peg.5782	Protein	36248	39094	+	FIG00826446: hypothetical protein
contig_8	fig|6666666.28487.peg.5783	Protein	39873	39091	-	3-oxoacyl-[acyl-carrier protein] reductase (EC 1.1.1.100)
contig_8	fig|6666666.28487.peg.5784	Protein	40211	39870	-	Cyclohexanone monooxygenase (EC 1.14.13.22)
contig_8	fig|6666666.28487.peg.5785	Protein	41115	40237	-	Peroxidase BpoA
contig_8	fig|6666666.28487.peg.5786	Protein	41163	41969	+	Glucose 1-dehydrogenase (EC 1.1.1.47)
contig_8	fig|6666666.28487.peg.5787	Protein	42295	41933	-	FIG00828220: hypothetical protein
contig_8	fig|6666666.28487.peg.5788	Protein	43051	42398	-	Nicotinamidase (EC 3.5.1.19)
contig_8	fig|6666666.28487.peg.5789	Protein	43304	44416	+	Osmosensitive K+ channel histidine kinase KdpD (EC 2.7.3.-)
contig_8	fig|6666666.28487.peg.5790	Protein	44571	46514	+	Probable monooxygenase Y4ID (EC 1.14.13.-)
contig_8	fig|6666666.28487.peg.5791	Protein	46516	47541	+	Threonine dehydrogenase and related Zn-dependent dehydrogenases
contig_8	fig|6666666.28487.peg.5792	Protein	47994	47635	-	hypothetical protein
contig_8	fig|6666666.28487.peg.5793	Protein	48038	48799	+	putative transcriptional regulator
contig_8	fig|6666666.28487.peg.5794	Protein	50173	48875	-	PPE family protein

contig_8	fig|6666666.28487.peg.5795	Protein	53056	50801	-	Phytochrome, two-component sensor histidine kinase (EC 2.7.3.-)
contig_8	fig|6666666.28487.peg.5796	Protein	53078	53269	+	hypothetical protein
contig_8	fig|6666666.28487.peg.5797	Protein	53886	53242	-	Porin
contig_8	fig|6666666.28487.peg.5798	Protein	54437	54183	-	Anti-sigma F factor antagonist (spoIIAA-2); Anti-sigma B factor antagonist RsbV
contig_8	fig|6666666.28487.peg.5799	Protein	57025	54497	-	Serine phosphatase RsbU, regulator of sigma subunit
contig_8	fig|6666666.28487.peg.5800	Protein	57904	57542	-	transcriptional regulator, ArsR family
contig_8	fig|6666666.28487.peg.5801	Protein	59791	58109	-	putative transporter
contig_8	fig|6666666.28487.peg.5802	Protein	60576	59797	-	hypothetical protein
contig_8	fig|6666666.28487.peg.5803	Protein	61499	60654	-	ABC transporter, ATP-binding protein
contig_8	fig|6666666.28487.peg.5804	Protein	67030	61496	-	Malonyl CoA-acyl carrier protein transacylase (EC 2.3.1.39)
contig_8	fig|6666666.28487.peg.5805	Protein	68814	67045	-	Halogenase
contig_8	fig|6666666.28487.peg.5806	Protein	70329	68998	-	Succinate-semialdehyde dehydrogenase [NAD] (EC 1.2.1.24); Succinate-semialdehyde dehydrogenase [NADP+] (EC 1.2.1.16)
contig_8	fig|6666666.28487.peg.5807	Protein	70778	70341	-	zinc/iron permease
contig_8	fig|6666666.28487.peg.5808	Protein	70988	71845	+	hypothetical protein
contig_8	fig|6666666.28487.peg.5809	Protein	72144	71884	-	hypothetical protein
contig_8	fig|6666666.28487.peg.5810	Protein	72022	73587	+	membrane protein, putative
contig_8	fig|6666666.28487.peg.5811	Protein	73584	73790	+	hypothetical protein

contig_8	fig|6666666.28487.peg.5812	Protein	74327	74163	-	hypothetical protein
contig_80	fig|6666666.28487.peg.5813	Protein	99	1490	+	PROBABLE TRANSPOSASE
contig_81	fig|6666666.28487.peg.5814	Protein	736	38	-	Urease accessory protein UreF
contig_81	fig|6666666.28487.peg.5815	Protein	1196	717	-	hypothetical protein
contig_81	fig|6666666.28487.peg.5816	Protein	2023	1193	-	Urease accessory protein UreD
contig_81	fig|6666666.28487.peg.5817	Protein	2640	2020	-	Urease accessory protein UreG
contig_81	fig|6666666.28487.peg.5818	Protein	4369	2654	-	Urease alpha subunit (EC 3.5.1.5)
contig_81	fig|6666666.28487.peg.5819	Protein	5075	4371	-	urease, beta subunit
contig_81	fig|6666666.28487.peg.5820	Protein	6278	5079	-	Urea ABC transporter, urea binding protein
contig_81	fig|6666666.28487.peg.5821	Protein	6277	7704	+	response regulator receiver protein
contig_81	fig|6666666.28487.peg.5822	Protein	8131	8556	+	endoribonuclease L-psp family protein
contig_81	fig|6666666.28487.peg.5823	Protein	8585	9703	+	NADH:flavin oxidoreductase/NADH oxidase
contig_81	fig|6666666.28487.peg.5824	Protein	10287	9700	-	Transcriptional regulator, TetR family
contig_81	fig|6666666.28487.peg.5825	Protein	10393	11106	+	Putative oxidoreductase
contig_81	fig|6666666.28487.peg.5826	Protein	11171	11359	+	FIG00833209: hypothetical protein
contig_81	fig|6666666.28487.peg.5827	Protein	11562	12773	+	3-ketoacyl-CoA thiolase [isoleucine degradation] (EC 2.3.1.16)
contig_81	fig|6666666.28487.peg.5828	Protein	12788	14935	+	Enoyl-CoA hydratase [isoleucine degradation] (EC 4.2.1.17) / 3-hydroxyacyl-CoA dehydrogenase (EC 1.1.1.35) / 3-hydroxybutyryl-CoA epimerase (EC 5.1.2.3)
contig_81	fig|6666666.28487.peg.5829	Protein	15071	14907	-	hypothetical protein

contig_81	fig|6666666.28487.peg.5830	Protein	15398	16360	+	Transcriptional regulator, AraC family
contig_81	fig|6666666.28487.peg.5831	Protein	16402	17898	+	Long-chain-fatty-acid--CoA ligase (EC 6.2.1.3)
contig_81	fig|6666666.28487.peg.5832	Protein	18324	17899	-	FIG00828686: hypothetical protein
contig_81	fig|6666666.28487.peg.5833	Protein	18409	19401	+	Luciferase family protein
contig_81	fig|6666666.28487.peg.5834	Protein	19774	19415	-	conserved hypothetical protein; putative Glyoxalase domain
contig_81	fig|6666666.28487.peg.5835	Protein	22618	22481	-	hypothetical protein
contig_82	fig|6666666.28487.peg.5836	Protein	286	735	+	hypothetical protein
contig_82	fig|6666666.28487.peg.5837	Protein	1667	1485	-	hypothetical protein
contig_82	fig|6666666.28487.peg.5838	Protein	2075	2389	+	FIG036251: Hypothetical protein
contig_82	fig|6666666.28487.peg.5839	Protein	2386	2910	+	FIG00820930: hypothetical protein
contig_82	fig|6666666.28487.peg.5840	Protein	3729	4637	+	hypothetical protein
contig_82	fig|6666666.28487.peg.5841	Protein	4679	5449	+	Transcriptional regulator, AraC family
contig_82	fig|6666666.28487.peg.5842	Protein	5818	6240	+	hypothetical protein
contig_82	fig|6666666.28487.peg.5843	Protein	6312	6770	+	hypothetical protein
contig_83	fig|6666666.28487.peg.5844	Protein	173	505	+	Lipoprotein LprD
contig_83	fig|6666666.28487.peg.5845	Protein	524	868	+	CONSERVED MEMBRANE PROTEIN
contig_83	fig|6666666.28487.peg.5846	Protein	909	2171	+	Integral membrane acyltransferase
contig_83	fig|6666666.28487.peg.5847	Protein	3426	2644	-	Ribonuclease PH (EC 2.7.7.56)
contig_83	fig|6666666.28487.peg.5848	Protein	4220	3486	-	Metal-dependent hydrolases of the beta-lactamase superfamily III

contig_83	fig|6666666.28487.peg.5849	Protein	5148	4318	-	Glutamate racemase (EC 5.1.1.3)
contig_83	fig|6666666.28487.peg.5850	Protein	5762	5145	-	Rhomboid membrane family protein
contig_83	fig|6666666.28487.peg.5851	Protein	6793	5819	-	Cysteine synthase B (EC 2.5.1.47)
contig_83	fig|6666666.28487.peg.5852	Protein	7070	6804	-	9.5 kDa culture filtrate antigen Cfp10A
contig_83	fig|6666666.28487.peg.5853	Protein	7503	7090	-	Mec+
contig_83	fig|6666666.28487.peg.5854	Protein	8536	7514	-	possible hydrolase
contig_84	fig|6666666.28487.peg.5855	Protein	117	929	+	Putative competence protein ComEA
contig_84	fig|6666666.28487.peg.5856	Protein	935	2398	+	DNA internalization-related competence protein ComEC/Rec2
contig_84	fig|6666666.28487.peg.5857	Protein	2401	3351	+	DNA polymerase III delta subunit (EC 2.7.7.7)
contig_84	fig|6666666.28487.peg.5858	Protein	3669	3409	-	SSU ribosomal protein S20p
contig_84	fig|6666666.28487.peg.5859	Protein	3874	5508	+	Protein containing domains DUF404, DUF407
contig_84	fig|6666666.28487.peg.5860	Protein	5523	6497	+	Protein containing domains DUF403
contig_84	fig|6666666.28487.peg.5861	Protein	6498	7337	+	Protein containing transglutaminase-like domain, putative cysteine protease
contig_84	fig|6666666.28487.peg.5862	Protein	8191	7334	-	metallo-beta-lactamase family protein
contig_84	fig|6666666.28487.peg.5863	Protein	9258	8251	-	Aldo-keto reductase
contig_84	fig|6666666.28487.peg.5864	Protein	9376	9804	+	putative signal-transduction protein with CBS domains
contig_84	fig|6666666.28487.peg.5865	Protein	9804	10412	+	puromycin N-acetyltransferase, putative
contig_84	fig|6666666.28487.peg.5866	Protein	11006	10440	-	RNA 3'-terminal phosphate cyclase (EC 6.5.1.4)
contig_84	fig|6666666.28487.peg.5867	Protein	11152	11003	-	hypothetical protein

contig_84	fig|6666666.28487.peg.5868	Protein	11120	13006	+	Translation elongation factor LepA
contig_84	fig|6666666.28487.peg.5869	Protein	13045	13713	+	conserved hypothetical protein
contig_84	fig|6666666.28487.peg.5870	Protein	14152	13757	-	hypothetical protein
contig_84	fig|6666666.28487.peg.5871	Protein	15145	14192	-	Membrane protein, TerC family
contig_84	fig|6666666.28487.peg.5872	Protein	15733	15182	-	Inosine-5'-monophosphate dehydrogenase (EC 1.1.1.205)
contig_84	fig|6666666.28487.peg.5873	Protein	15890	17413	+	NADH dehydrogenase, subunit 5
contig_84	fig|6666666.28487.peg.5874	Protein	17410	19968	+	Hypothetical transmembrane protein coupled to NADH-ubiquinone oxidoreductase chain 5 homolog
contig_84	fig|6666666.28487.peg.5875	Protein	19965	20285	+	hypothetical protein
contig_84	fig|6666666.28487.peg.5876	Protein	20312	20899	+	Carbonic anhydrase (EC 4.2.1.1)
contig_84	fig|6666666.28487.peg.5877	Protein	20965	21213	+	hypothetical protein
contig_84	fig|6666666.28487.peg.5878	Protein	21444	22187	+	PROBABLE CONSERVED LIPOPROTEIN LPPR
contig_84	fig|6666666.28487.peg.5879	Protein	22232	22942	+	Serine/threonine protein kinase (EC 2.7.11.1)
contig_84	fig|6666666.28487.peg.5880	Protein	25021	23015	-	Glucoamylase (EC 3.2.1.3)
contig_84	fig|6666666.28487.peg.5881	Protein	25107	25319	+	Cell division protein DivIC (FtsB), stabilizes FtsL against RasP cleavage
contig_85	fig|6666666.28487.peg.5882	Protein	83	1363	+	EAL domain:GAF
contig_85	fig|6666666.28487.peg.5883	Protein	1996	3009	+	PE-PPE, C-terminal domain protein
contig_85	fig|6666666.28487.peg.5884	Protein	3265	4515	+	putative cytochrome P450 hydroxylase
contig_85	fig|6666666.28487.peg.5885	Protein	4508	5128	+	Transcriptional regulator, TetR family
contig_86	fig|6666666.28487.peg.5886	Protein	96	5987	+	ATP-dependent exoDNAse (exonuclease V) alpha subunit -

						helicase superfamily I member-like protein
contig_86	fig|6666666.28487.peg.5887	Protein	6414	6073	-	hypothetical protein
contig_86	fig|6666666.28487.peg.5888	Protein	7226	6411	-	D12 class N6 adenine-specific DNA methyltransferase
contig_86	fig|6666666.28487.peg.5889	Protein	8014	7241	-	hypothetical protein
contig_86	fig|6666666.28487.peg.5890	Protein	8475	8014	-	hypothetical protein
contig_86	fig|6666666.28487.peg.5891	Protein	8560	8850	+	hypothetical protein
contig_86	fig|6666666.28487.peg.5892	Protein	9296	8802	-	hypothetical protein
contig_87	fig|6666666.28487.peg.5893	Protein	98	865	+	membrane protein, putative
contig_87	fig|6666666.28487.peg.5894	Protein	896	1021	+	FIG00820358: hypothetical protein
contig_87	fig|6666666.28487.peg.5895	Protein	1076	2002	+	Sulfate transporter, CysZ-type
contig_87	fig|6666666.28487.peg.5896	Protein	3472	1982	-	FIG00825370: hypothetical protein
contig_87	fig|6666666.28487.rna.50	RNA	3574	3647	+	tRNA-Pro-GGG
contig_88	fig|6666666.28487.peg.5897	Protein	809	48	-	Methyltransferase type 11
contig_88	fig|6666666.28487.peg.5898	Protein	1692	802	-	5,10-methylenetetrahydrofolate reductase (EC 1.5.1.20)
contig_88	fig|6666666.28487.peg.5899	Protein	2573	1884	-	putative conserved transmembrane protein
contig_88	fig|6666666.28487.peg.5900	Protein	3166	2570	-	hypothetical protein
contig_88	fig|6666666.28487.peg.5901	Protein	3207	5204	+	Metallopeptidase
contig_88	fig|6666666.28487.peg.5902	Protein	5683	5243	-	putative thiol-disulphide oxidoreductase DCC
contig_88	fig|6666666.28487.peg.5903	Protein	6164	5760	-	Possible membrane protein

contig_88	fig|6666666.28487.peg.5904	Protein	7333	6170	-	POSSIBLE CONSERVED SECRETED PROTEIN
contig_88	fig|6666666.28487.peg.5905	Protein	8337	7384	-	Probable methanol dehydrogenase transcriptional regulatory protein MoxR3
contig_88	fig|6666666.28487.peg.5906	Protein	8816	8334	-	FIG00997183: hypothetical protein
contig_88	fig|6666666.28487.peg.5907	Protein	9748	8813	-	FIG00995849: hypothetical protein
contig_88	fig|6666666.28487.peg.5908	Protein	10821	9811	-	putative lipoprotein
contig_88	fig|6666666.28487.peg.5909	Protein	11001	11297	+	Conserved protein
contig_88	fig|6666666.28487.peg.5910	Protein	11741	11319	-	hypothetical protein
contig_88	fig|6666666.28487.peg.5911	Protein	11933	12559	+	hypothetical protein
contig_88	fig|6666666.28487.peg.5912	Protein	12793	12575	-	FIG00820970: hypothetical protein
contig_89	fig|6666666.28487.peg.5913	Protein	211	456	+	hypothetical protein
contig_89	fig|6666666.28487.peg.5914	Protein	504	863	+	FIG00825156: hypothetical protein
contig_89	fig|6666666.28487.peg.5915	Protein	925	1767	+	putative DNA-binding protein
contig_89	fig|6666666.28487.peg.5916	Protein	2468	1782	-	Response regulator receiver and ANTAR domain protein
contig_89	fig|6666666.28487.peg.5917	Protein	2660	3877	+	3-ketoacyl-CoA thiolase (EC 2.3.1.16)
contig_89	fig|6666666.28487.peg.5918	Protein	3874	5394	+	Lignostilbene-alpha,beta-dioxygenase and related enzymes
contig_89	fig|6666666.28487.peg.5919	Protein	5391	6290	+	Transcriptional regulator, HxlR family
contig_89	fig|6666666.28487.peg.5920	Protein	6287	6844	+	Isochorismatase (EC 3.3.2.1)
contig_89	fig|6666666.28487.peg.5921	Protein	7509	6901	-	GCN5-related N-acetyltransferase
contig_9	fig|6666666.28487.peg.5922	Protein	577	74	-	Methylated-DNA--protein-cysteine methyltransferase (EC

						2.1.1.63)
contig_9	fig|6666666.28487.peg.5923	Protein	2064	577	-	Methylated-DNA--protein-cysteine methyltransferase (EC 2.1.1.63)
contig_9	fig|6666666.28487.peg.5924	Protein	2166	2822	+	Transcriptional regulator, TetR family
contig_9	fig|6666666.28487.peg.5925	Protein	2791	3528	+	PROBABLE CONSERVED INTEGRAL MEMBRANE ALANINE AND LEUCINE RICH PROTEIN
contig_9	fig|6666666.28487.peg.5926	Protein	5128	3515	-	Adenylate cyclase (EC 4.6.1.1)
contig_9	fig|6666666.28487.peg.5927	Protein	5176	5847	+	FIG00996186: hypothetical protein
contig_9	fig|6666666.28487.peg.5928	Protein	6112	5981	-	hypothetical protein
contig_9	fig|6666666.28487.peg.5929	Protein	6610	6137	-	Methylmalonyl-CoA epimerase (EC 5.1.99.1); Ethylmalonyl-CoA epimerase
contig_9	fig|6666666.28487.peg.5930	Protein	6714	7895	+	3-ketoacyl-CoA thiolase (EC 2.3.1.16) @ Acetyl-CoA acetyltransferase (EC 2.3.1.9)
contig_9	fig|6666666.28487.peg.5931	Protein	7958	8332	+	inner membrane protein
contig_9	fig|6666666.28487.peg.5932	Protein	8463	9242	+	COG3118: Thioredoxin domain-containing protein EC-YbbN
contig_9	fig|6666666.28487.peg.5933	Protein	11465	9252	-	1,4-alpha-glucan (glycogen) branching enzyme, GH-13-type (EC 2.4.1.18)
contig_9	fig|6666666.28487.peg.5934	Protein	13560	11470	-	Putative glucanase glgE (EC 3.2.1.-)
contig_9	fig|6666666.28487.peg.5935	Protein	13819	16449	+	Glycogen phosphorylase (EC 2.4.1.1)
contig_9	fig|6666666.28487.peg.5936	Protein	17969	16542	-	POSSIBLE LIPOPROTEIN PEPTIDASE LPQM
contig_9	fig|6666666.28487.peg.5937	Protein	17851	18018	+	hypothetical protein

contig_9	fig|6666666.28487.peg.5938	Protein	20097	18118	-	DinG family ATP-dependent helicase YoaA
contig_9	fig|6666666.28487.peg.5939	Protein	21459	20104	-	Nicotinate phosphoribosyltransferase (EC 2.4.2.11)
contig_9	fig|6666666.28487.peg.5940	Protein	21512	21814	+	ATP-dependent Clp protease adaptor protein ClpS
contig_9	fig|6666666.28487.peg.5941	Protein	21822	22406	+	Transcriptional regulatory protein
contig_90	fig|6666666.28487.peg.5942	Protein	348	13	-	Mobile element protein
contig_90	fig|6666666.28487.peg.5943	Protein	3352	641	-	hypothetical protein
contig_90	fig|6666666.28487.peg.5944	Protein	3516	3358	-	hypothetical protein
contig_90	fig|6666666.28487.peg.5945	Protein	7358	3996	-	hypothetical protein
contig_90	fig|6666666.28487.peg.5946	Protein	7575	8201	+	Possible membrane protein
contig_90	fig|6666666.28487.peg.5947	Protein	8522	8319	-	hypothetical protein
contig_91	fig|6666666.28487.peg.5948	Protein	871	32	-	Mobile element protein
contig_91	fig|6666666.28487.peg.5949	Protein	1293	949	-	Mobile element protein
contig_92	fig|6666666.28487.peg.5950	Protein	57	689	+	13E12 repeat family protein
contig_93	fig|6666666.28487.peg.5951	Protein	178	975	+	FIG00829768: hypothetical protein
contig_93	fig|6666666.28487.peg.5952	Protein	1314	2339	+	NLP/P60 family protein
contig_93	fig|6666666.28487.peg.5953	Protein	2450	3721	+	ErfK/YbiS/YcfS/YnhG family protein
contig_93	fig|6666666.28487.peg.5954	Protein	4283	3729	-	NLP/P60 family protein
contig_94	fig|6666666.28487.peg.5955	Protein	644	153	-	hypothetical protein
contig_94	fig|6666666.28487.peg.5956	Protein	2076	655	-	Transcriptional regulator, GntR family domain / Aspartate aminotransferase (EC 2.6.1.1)

contig_94	fig|6666666.28487.peg.5957	Protein	2120	2581	+	4-carboxymuconolactone decarboxylase domain/alkylhydroperoxidase AhpD family core domain protein
contig_94	fig|6666666.28487.peg.5958	Protein	2597	3358	+	FIG00821910: hypothetical protein
contig_94	fig|6666666.28487.peg.5959	Protein	3669	3355	-	FIG00822152: hypothetical protein
contig_94	fig|6666666.28487.peg.5960	Protein	3749	4192	+	FIG00828620: hypothetical protein
contig_94	fig|6666666.28487.peg.5961	Protein	5026	4196	-	O-antigen export system permease protein RfbD
contig_94	fig|6666666.28487.peg.5962	Protein	5939	5040	-	Putative glycosyl transferase
contig_94	fig|6666666.28487.peg.5963	Protein	6751	5936	-	O-antigen export system, ATP-binding protein
contig_94	fig|6666666.28487.peg.5964	Protein	7283	6759	-	FIG00822472: hypothetical protein
contig_94	fig|6666666.28487.peg.5965	Protein	7657	8853	+	Cysteine desulfurase (EC 2.8.1.7)
contig_95	fig|6666666.28487.peg.5966	Protein	58	702	+	FIG00820187: hypothetical protein
contig_95	fig|6666666.28487.peg.5967	Protein	702	1847	+	FadE30
contig_95	fig|6666666.28487.peg.5968	Protein	1844	2836	+	Acyl-CoA dehydrogenase, short-chain specific (EC 1.3.99.2)
contig_95	fig|6666666.28487.peg.5969	Protein	3643	2840	-	3-oxoacyl-[acyl-carrier protein] reductase (EC 1.1.1.100)
contig_95	fig|6666666.28487.peg.5970	Protein	3726	4790	+	FIG00823642: hypothetical protein
contig_95	fig|6666666.28487.peg.5971	Protein	4965	5267	+	hypothetical protein
contig_95	fig|6666666.28487.peg.5972	Protein	6227	5388	-	Hypothetical protein DUF194, DegV family
contig_95	fig|6666666.28487.peg.5973	Protein	6983	6237	-	FIG00999365: hypothetical protein
contig_95	fig|6666666.28487.peg.5974	Protein	7650	6973	-	Phosphoglycerate mutase (EC 5.4.2.1)

contig_95	fig|6666666.28487.peg.5975	Protein	8024	7647	-	Iojap protein
contig_95	fig|6666666.28487.peg.5976	Protein	8662	8048	-	Nicotinate-nucleotide adenylyltransferase (EC 2.7.7.18)
contig_95	fig|6666666.28487.peg.5977	Protein	9446	8742	-	Transcriptional regulator, GntR family
contig_95	fig|6666666.28487.peg.5978	Protein	9616	11097	+	Urea carboxylase-related amino acid permease
contig_95	fig|6666666.28487.peg.5979	Protein	11094	12542	+	Glycerol kinase (EC 2.7.1.30)
contig_95	fig|6666666.28487.peg.5980	Protein	12539	13912	+	Possible Glycerol-3-phosphate dehydrogenase (EC 1.1.5.3)
contig_95	fig|6666666.28487.peg.5981	Protein	13909	15153	+	Sarcosine oxidase alpha subunit (EC 1.5.3.1)
contig_95	fig|6666666.28487.peg.5982	Protein	15605	15240	-	FIG00827264: hypothetical protein
contig_95	fig|6666666.28487.peg.5983	Protein	16020	16235	+	hypothetical protein
contig_95	fig|6666666.28487.peg.5984	Protein	16765	16241	-	hypothetical protein
contig_95	fig|6666666.28487.peg.5985	Protein	17627	16779	-	FIG00823882: hypothetical protein
contig_95	fig|6666666.28487.peg.5986	Protein	18850	17660	-	Cupin 4 family protein
contig_95	fig|6666666.28487.peg.5987	Protein	19079	18891	-	hypothetical protein
contig_95	fig|6666666.28487.peg.5988	Protein	20569	19208	-	VWA containing CoxE family protein
contig_95	fig|6666666.28487.peg.5989	Protein	21516	20641	-	MoxR-like ATPases
contig_95	fig|6666666.28487.peg.5990	Protein	22779	21532	-	Gamma-glutamyl phosphate reductase (EC 1.2.1.41)
contig_95	fig|6666666.28487.peg.5991	Protein	22884	23735	+	Ribokinase (EC 2.7.1.15)
contig_96	fig|6666666.28487.peg.5992	Protein	12	479	+	Mobile element protein
contig_97	fig|6666666.28487.peg.5993	Protein	376	56	-	hypothetical protein

contig_98	fig|6666666.28487.peg.5994	Protein	79	1302	+	Mobile element protein
contig_98	fig|6666666.28487.peg.5995	Protein	1299	2102	+	Mobile element protein
